# Subnational mapping of under-5 and neonatal mortality trends in India: the Global Burden of Disease Study 2000–17

**DOI:** 10.1016/S0140-6736(20)30471-2

**Published:** 2020-05-23

**Authors:** India State-Level Disease Burden Initiative Child Mortality Collaborators, Rakhi Dandona, G Anil Kumar, Nathaniel J Henry, Vasna Joshua, Siddarth Ramji, Subodh S Gupta, Deepti Agrawal, Rashmi Kumar, Rakesh Lodha, Matthews Mathai, Nicholas J Kassebaum, Anamika Pandey, Haidong Wang, Anju Sinha, Rajkumar Hemalatha, Rizwan S Abdulkader, Vivek Agarwal, Sandra Albert, Atanu Biswas, Roy Burstein, Joy K Chakma, D J Christopher, Michael Collison, A P Dash, Sagnik Dey, Daniel Dicker, William Gardner, Scott D Glenn, Mahaveer J Golechha, Yihua He, Suparna G Jerath, Rajni Kant, Anita Kar, Ajay K Khera, Sanjay Kinra, Parvaiz A Koul, Varsha Krish, Rinu P Krishnankutty, Anura V Kurpad, Hmwe H Kyu, Avula Laxmaiah, Jagadish Mahanta, P A Mahesh, Ridhima Malhotra, Raja S Mamidi, Helena Manguerra, Joseph L Mathew, Manu R Mathur, Ravi Mehrotra, Satinath Mukhopadhyay, G V S Murthy, Parul Mutreja, Balakrishna Nagalla, Grant Nguyen, Anu M Oommen, Ashalata Pati, Sanghamitra Pati, Samantha Perkins, Sanjay Prakash, Manorama Purwar, Rajesh Sagar, Mari J Sankar, Deepika S Saraf, D K Shukla, Sharvari R Shukla, Narinder P Singh, V Sreenivas, Babasaheb Tandale, Kavumpurathu R Thankappan, Manjari Tripathi, Suryakant Tripathi, Srikanth Tripathy, Christopher Troeger, Chris M Varghese, Santosh Varughese, Stefanie Watson, Geetika Yadav, Sanjay Zodpey, K Srinath Reddy, G S Toteja, Mohsen Naghavi, Stephen S Lim, Theo Vos, Hendrik J Bekedam, Soumya Swaminathan, Christopher J L Murray, Simon I Hay, R S Sharma, Lalit Dandona

## Abstract

**Background:**

India has made substantial progress in improving child survival over the past few decades, but a comprehensive understanding of child mortality trends at disaggregated geographical levels is not available. We present a detailed analysis of subnational trends of child mortality to inform efforts aimed at meeting the India National Health Policy (NHP) and Sustainable Development Goal (SDG) targets for child mortality.

**Methods:**

We assessed the under-5 mortality rate (U5MR) and neonatal mortality rate (NMR) from 2000 to 2017 in 5 × 5 km grids across India, and for the districts and states of India, using all accessible data from various sources including surveys with subnational geographical information. The 31 states and groups of union territories were categorised into three groups using their Socio-demographic Index (SDI) level, calculated as part of the Global Burden of Diseases, Injuries, and Risk Factors Study on the basis of per-capita income, mean education, and total fertility rate in women younger than 25 years. Inequality between districts within the states was assessed using the coefficient of variation. We projected U5MR and NMR for the states and districts up to 2025 and 2030 on the basis of the trends from 2000 to 2017 and compared these projections with the NHP 2025 and SDG 2030 targets for U5MR (23 deaths and 25 deaths per 1000 livebirths, respectively) and NMR (16 deaths and 12 deaths per 1000 livebirths, respectively). We assessed the causes of child death and the contribution of risk factors to child deaths at the state level.

**Findings:**

U5MR in India decreased from 83·1 (95% uncertainty interval [UI] 76·7–90·1) in 2000 to 42·4 (36·5–50·0) per 1000 livebirths in 2017, and NMR from 38·0 (34·2–41·6) to 23·5 (20·1–27·8) per 1000 livebirths. U5MR varied 5·7 times between the states of India and 10·5 times between the 723 districts of India in 2017, whereas NMR varied 4·5 times and 8·0 times, respectively. In the low SDI states, 275 (88%) districts had a U5MR of 40 or more per 1000 livebirths and 291 (93%) districts had an NMR of 20 or more per 1000 livebirths in 2017. The annual rate of change from 2010 to 2017 varied among the districts from a 9·02% (95% UI 6·30–11·63) reduction to no significant change for U5MR and from an 8·05% (95% UI 5·34–10·74) reduction to no significant change for NMR. Inequality between districts within the states increased from 2000 to 2017 in 23 of the 31 states for U5MR and in 24 states for NMR, with the largest increases in Odisha and Assam among the low SDI states. If the trends observed up to 2017 were to continue, India would meet the SDG 2030 U5MR target but not the SDG 2030 NMR target or either of the NHP 2025 targets. To reach the SDG 2030 targets individually, 246 (34%) districts for U5MR and 430 (59%) districts for NMR would need a higher rate of improvement than they had up to 2017. For all major causes of under-5 death in India, the death rate decreased between 2000 and 2017, with the highest decline for infectious diseases, intermediate decline for neonatal disorders, and the smallest decline for congenital birth defects, although the magnitude of decline varied widely between the states. Child and maternal malnutrition was the predominant risk factor, to which 68·2% (65·8–70·7) of under-5 deaths and 83·0% (80·6–85·0) of neonatal deaths in India could be attributed in 2017; 10·8% (9·1–12·4) of under-5 deaths could be attributed to unsafe water and sanitation and 8·8% (7·0–10·3) to air pollution.

**Interpretation:**

India has made gains in child survival, but there are substantial variations between the states in the magnitude and rate of decline in mortality, and even higher variations between the districts of India. Inequality between districts within states has increased for the majority of the states. The district-level trends presented here can provide crucial guidance for targeted efforts needed in India to reduce child mortality to meet the Indian and global child survival targets. District-level mortality trends along with state-level trends in causes of under-5 and neonatal death and the risk factors in this Article provide a comprehensive reference for further planning of child mortality reduction in India.

**Funding:**

Bill & Melinda Gates Foundation; and Indian Council of Medical Research, Department of Health Research, Ministry of Health and Family Welfare, Government of India.

**Research in context**

**Evidence before this study**

India has the largest proportion of under-5 deaths globally. We searched PubMed on Aug 4, 2019, for published literature on child mortality rates and mapping, causes of death, and risk factors in India, as well as Google for reports in the public domain, and references in these papers and reports, using the search terms “cause of death”, “child mortality”, “child mortality targets”, “death”, “district-level”, “epidemiology”, “geospatial”, “geospatial mapping”, “India”, “inequality”, “infant mortality rate”, “neonatal mortality rate”, “risk factors”, “sustainable development goals”, “trends”, “under-5 deaths”, and “under-5 mortality rate”, without language or publication date restrictions. We found that many previous studies have reported child mortality at the national level and state level, but very few studies have reported district-level variation in child mortality. One study reported estimation of child mortality rates at the district level in relation to the Sustainable Development Goal (SDG) 2030 targets, using a single data source. A comprehensive understanding of the variations in child mortality between the districts of India, the trends over time in relation to the India National Health Policy and SDG targets, and the causes of death and risk factors, using all accessible data sources from India in the same framework, has not been reported.

**Added value of this study**

This study uses all accessible data sources to produce fine-grid estimates of under-5 and neonatal mortality rates across India, and provides trends for every district of India from 2000 to 2017. It estimates inequality between districts within states for under-5 and neonatal mortality rates against the improving overall rates. The findings include a comparison of trends in each district with the child mortality targets set by India's National Health Policy for 2025 as well as the SDG 2030 targets. This report suggests an approach combining the different levels of both mortality rates and the rate of decline to identity priority districts in each state that could be useful for policy makers. Using the standardised approach of the Global Burden of Diseases, Injuries, and Risk Factors Study, this study analyses trends in the major causes of child mortality and risk factors in every state of India in the same framework, which provides insights into the variations in child mortality rates among the states.

**Implications of all the available evidence**

To our knowledge, this study reports the most comprehensive understanding of child mortality trends across the districts of India so far. The findings highlight that a large proportion of districts need acceleration in the reduction of child mortality to reach Indian and global targets. The specific trends for each district are a useful reference for further efforts by the government and other stakeholders for decentralised health planning aimed at improving child survival in India, especially in areas that have persistently high child mortality rates and low rates of mortality reduction.

## Introduction

A remarkable decline has occurred over the past several decades in under-5 mortality across countries worldwide, reflecting the national and global commitment and investment to improve child survival.^1^, ^2^ With growing evidence globally that national mortality estimates obscure variations at subnational levels at which health programme planning and implementation occur, a better understanding is needed about where the largest gaps in child survival are at more disaggregated geographical levels to achieve the child survival targets of 25 deaths per 1000 livebirths for under-5 mortality rate (U5MR) and 12 deaths per 1000 livebirths for neonatal mortality rate (NMR) set by the UN under the Sustainable Development Goals (SDGs) by 2030.^3^, ^4^, ^5^, ^6^, ^7^, ^8^

India had the largest proportion, about a fifth, of the 5·4 million under-5 deaths globally in 2017.^9^ Therefore, reducing child mortality in India is vital not only for India but also to further reduce global child mortality. India's National Health Policy (NHP) 2017 set a target of 23 deaths per 1000 livebirths for under-5 mortality and 16 deaths per 1000 livebirths for neonatal mortality by 2025, and the government has also set a target of fewer than ten neonatal deaths per 1000 livebirths by 2030 under the India Newborn Action Plan.^10^, ^11^ Achievement of these targets would be facilitated by deeper insights into the trends of U5MR and NMR at smaller geographical subnational levels. Some understanding of the subnational distribution of U5MR and NMR in India is available from previous reports.^12^, ^13^, ^14^, ^15^ These studies have used data from various sources, including national household surveys, censuses, and the Sample Registration System (SRS). One study also attempted estimation of child mortality at the district level in relation to the SDG targets, using only one round of the National Family Health Survey data for this analysis.^16^ Two studies over the past few years have reported causes of child death at the state level.^17^, ^18^ However, there has been no comprehensive consolidation of the trends in child mortality in all districts of India using all accessible data sources over a long period of time that also relates the district-level trends with NHP 2025 and SDG 2030 targets.

In this Article, we report geospatial analysis of U5MR and NMR in India at the state and district levels from 2000 to 2017, using all accessible data sources, and relate these trends to the NHP 2025 and SDG 2030 targets. We also report trends in major causes of child death in relation to child mortality trends, and the contribution of risk factors to child deaths at the state level, which could inform further planning to improve child survival across India.

## Methods

### Overview

The analysis and findings presented in this Article were produced by the India State-Level Disease Burden Initiative as part of the Global Burden of Diseases, Injuries, and Risk Factors Study (GBD) 2017. The work of this initiative has been approved by the Health Ministry Screening Committee of the Indian Council of Medical Research and the ethics committee of the Public Health Foundation of India. Detailed description of the metrics, data sources, and statistical modelling for child mortality at various geographical levels down to the 5 × 5 km grids, and causes of death and risk factors, have been reported elsewhere.^1^, ^4^, ^7^, ^19^, ^20^, ^21^ The methods relevant for this Article are summarised here and described further in the appendix (pp 3–67).

### Estimation and mapping of child mortality

The main data sources for the estimation of child mortality in India in GBD 2017 were the SRS, vital registration system, censuses, and large national household surveys including the National Family Health Surveys, District Level Household Surveys, and Annual Health Surveys (appendix pp 68–82).^5^ GBD used complete birth history data from household surveys when available, and summary birth history data when complete birth history was not available, for the estimation of child mortality indicators. Complete birth history data provide the month and year of birth and death of the children, reported retrospectively by mothers. These data were used to generate U5MR for each year.^1^, ^7^ By contrast, summary birth histories provide data on the number of livebirths for each woman and the number of their surviving children at the time of data collection without any specific information on the timing of births or deaths. Summary birth history data were prepared using validated indirect methods to estimate child mortality rates.^1^, ^7^, ^19^, ^22^ Using all accessible data, GBD estimated time trends of child mortality indicators.

A spatiotemporal Gaussian process regression (ST-GPR) was used to estimate trends of child mortality indicators at the state level. This included covariates such as lag-distributed income per capita and average years of schooling for women aged 15–49 years. This model also included random effects for each data source as well as fixed effects for each data source type in India to adjust for the reference sources. A weighted polynomial regression function was fitted to systematically estimate the residual variability by borrowing strength across time, age, and space patterns using the spatiotemporal component of ST-GPR. The time adjustment parameter borrowed strength from neighbouring timepoints because exposure in a given year is highly correlated with exposure in the previous year, but less so further back in time. Similarly, the age adjustment parameter borrowed strength from data in neighbouring age groups and the space adjustment parameter borrowed strength across the hierarchy of geographical locations. This process substantially improved predictive accuracy by smoothening the residuals. Finally, the output from the non-linear mixed-effect model and the space and time smoothing are used as the prior for the Gaussian process regression.

Separate models were run for estimating the probability of death for each sex across different age groups, which included early neonatal (0–6 days), late neonatal (7–27 days), post neonatal (28–364 days), infant (<1 year), and younger than 5 years. Additionally, a stepwise process was undertaken to scale the mortality estimates for each of the most detailed age groupings to the aggregate age groups; this process ensured that the most detailed information is used when aggregating early neonatal and late neonatal mortality to calculate overall neonatal mortality. A modelling process was used that integrated multiple data inputs and borrowed information across age, time, and location to produce the best possible estimates of the time trends of the mortality indicators at the state level. Each data source was first scrutinised for data quality and then assessed for completeness of death registration for all age groups. The point estimates of child mortality indictors were then generated with both direct and indirect estimation methods using complete birth history and summary birth history data. Finally, ST-GPR modelling was used to estimate trends of child mortality indicators at the state level. This estimation process is described in detail in the appendix (pp 3–16).

The 5 × 5 km grid-level estimation process of child mortality included assessment of the probability and number of neonatal, infant, and under-5 deaths. These were computed for each year from 2000 to 2017 at a spatial resolution of a 0·042° × 0·042° grid cell over the globe, which is 5 × 5 km at the equator.^4^, ^7^, ^23^ Complete and summary birth history data were extracted from large-scale national household surveys (appendix pp 18–21). To estimate mortality probabilities and assign them to specific time periods, an indirect, discrete-time, generalised additive hazard model with covariates available from summary birth history data was fitted. The details of this method are published elsewhere.^4^, ^7^, ^22^ All extracted data for the estimation at 5 × 5 km grids were geo-referenced to either global positioning system (GPS) location points or, in the absence of GPS coordinates, to the smallest possible administrative units (polygons), most of which were districts. Administrative unit data were converted to points spread across the corresponding administrative division according to a resampling algorithm that accounted for population distribution. The combined dataset consisting of geo-referenced points and converted points provided the number of deaths and sample size for a particular location by age group and time period. Boundary information for administrative units in India for the year 2018 was obtained as shapefiles from ML Infomap. Mortality estimates were generated using a statistical model that was continuous in space, and prediction was done discretely on grid cells over India at a spatial resolution corresponding to approximately 5 × 5 km, and reported at the district, state, and country levels by applying a state-level scaling factor to harmonise the continuous mapping results with the GBD state-level and national-level estimates.^1^

### Projection of mortality rates up to 2030

Trends of U5MR and NMR from 1990 to 2017 were used to project these rates from 2018 to 2030 for India as part of GBD 2017, giving greater weight to more recent annual rates of change.^24^ To project mortality rates at 5 × 5 km grids up to 2030, the annual rate of change from 2000 to 2017 was applied to obtain the estimates for subsequent years, using a projection methodology that has been used previously for such geospatial analysis.^4^ Across 1000 draws, a logit-transformed annual rate of change from 2000 to 2017 was calculated at each pixel for U5MR and NMR, which was then applied to the final 2017 pixel estimates to generate the projected estimates up to 2030. Population-weighted aggregations of mortality at the district and state levels were calculated from the pixel draws, which were then harmonised with the national-level GBD projected rates by applying the relevant scaling factor.^25^ These methods are described in the appendix (pp 29–30) and elsewhere.^23^

### Estimation of causes of death

We used population-representative verbal autopsy cause of death data from various sources from 1980 onwards, which included the Registrar General of India's SRS and Survey of Causes of Death, Indian Council of Medical Research study, and several state-specific verbal autopsy studies covering child deaths, as well as the Medical Certification of Cause of Death data (appendix pp 31–33, 68–82). After the year 2000, the main source was the SRS, which provided data on 455 460 deaths, including 71 032 under-5 deaths, from 2004 to 2013 covering every state of India; state-specific verbal autopsy studies data were also included in the estimation.

Causes of death were estimated on the basis of the GBD cause list across the age groups, using the Causes of Death Ensemble model and other models. The quality and comparability of the cause of death data were assessed and enhanced through multiple steps using statistical models, which have been reported elsewhere.^20^ Briefly, the completeness of death records by cause was assessed using statistical models and these deaths were mapped to the International Classification of Diseases versions 9 and 10 codes to enable a consistent classification of the causes of death. Deaths that could not be specified to underlying causes were assigned to causes by redistributing them using regression models. This process reassigns deaths that were originally coded to health states unlikely to be an underlying cause of death (eg, birthing problem, respiratory distress, and abdominal pain), as well as deaths that were originally coded to non-specific causes of death. The CoDCorrect algorithm was then used to adjust the predicted estimated deaths for each cause to match with the estimated count of total all-cause mortality.^20^ Details of the cause of death estimation relevant for under-5 and neonatal deaths are provided in the appendix (pp 31–45) and available elsewhere.^20^ Causes of death were estimated at the state level.

### Estimation of child deaths attributable to risk factors

The GBD comparative risk assessment framework was used to estimate exposure to risk factors related to under-5 and neonatal deaths and their attributable mortality burden.^21^ Exposure data for risk factors with a categorical or continuous distribution were collated from all available data sources that could be accessed, including survey and other data. The estimates for exposure to each risk factor were based on the age-sex distribution of the exposure, which borrowed strength over space and time in models using covariates. The relative risk of disease outcomes was estimated for each risk exposure–disease outcome pair that had sufficient evidence of a causal relation in the global literature, including randomised controlled trials, prospective cohorts, and case-control studies. Using these exposure and relative risk estimates, the population attributable fractions for diseases from each risk factor were estimated by location, age, sex, and year. A description of the exposure and attributable disease burden estimation for the major risk factors associated with under-5 and neonatal deaths is provided in the appendix (pp 46–67) and published elsewhere.^21^

### Analysis presented in this paper

We report trends of U5MR and NMR from 2000 to 2017 for the 723 districts of India, aggregated from 5 × 5 km grid estimates across India, and for the 31 geographical units of India, including 29 states, the union territory of Delhi, and union territories other than Delhi. The state of Telangana was created in 2014 from a larger state; for trends before 2014, estimates were based on the districts that now constitute this state, using geolocated data to arrive at estimate for these districts. Jammu and Kashmir was divided into two union territories in August, 2019; as we are reporting findings up to 2017, we report findings for the undivided state of Jammu and Kashmir. We categorised the states into three groups on the basis of their Socio-demographic Index (SDI) as calculated in GBD 2017: low SDI (≤0·53), middle SDI (0·54–0·60), and high SDI (>0·60; appendix p 83).^26^, ^27^ SDI is a composite indicator of development status, which ranges from 0 to 1, and is a geometric mean of the values of the indices of lag-distributed per-capita income, mean education for those aged 15 years or older, and total fertility rate in women younger than 25 years.^26^ Since infant mortality rate does not have an SDG target, we report detailed trends only for U5MR and NMR in this Article, with estimates of infant mortality rates in 2000, 2010, and 2017 for the states and districts presented in the appendix (pp 84–97).

We report the rates of change for U5MR and NMR for the states and districts, highlighting the more recent changes from 2010 to 2017. We report inequality in U5MR and NMR between districts within each state using the coefficient of variation (CV), which is a simple metric of the relative spread of the two mortality rate distributions, defined as the ratio of the SD to the mean and expressed as a percentage. We also assessed how the CV of U5MR and NMR changed over time in each state. In states with 20 districts or more, we identified districts that need higher priority for child mortality reduction based on 3 × 3 grouping of districts in tertiles of U5MR and NMR in 2017 and the tertiles of their annual rate of reduction from 2010 to 2017 for the distribution within the states and the distribution across India. We compared the projected U5MR and NMR with the NHP 2025 targets (23 under-5 deaths and 16 neonatal deaths per 1000 livebirths) and the SDG 2030 targets (25 under-5 deaths and 12 neonatal deaths per 1000 livebirths).^8^, ^11^

We assessed trends of the major causes of under-5 and neonatal deaths from 2000 to 2017 at the state level, reporting specific causes which contributed 3% or more of the deaths. We assessed variations in the cause factions, death rate, and percentage change in death rate from 2000 to 2017 between the states. We assessed the Pearson correlation coefficient between under-5 cause-specific death rates with the SDI of the states in 2017, using their continuous distributions. We report trends of cause-fractions for neonatal deaths and highlight key differences between early and late neonatal deaths. Finally, we report the proportion of under-5 and neonatal deaths attributable to major risk factors and their trends from 2000 to 2017 at the state level and for the SDI state groups.

All estimates are reported with 95% uncertainty intervals (UIs) where relevant, which were based on 1000 draws for each estimate, with the mean taken as the point estimate and the 2·5th and 97·5th percentiles comprising the 95% UI (appendix p 30). Statistically significant change was defined as change for which the 95% UIs did not overlap zero.

### Role of the funding source

Some of the contributors to this Article work with the Indian Council of Medical Research. The Bill & Melinda Gates Foundation had no role in the study design, data collection, data analysis, data interpretation, or writing of this paper. The corresponding author had full access to all of the data in the study, and had final responsibility for the decision to submit for publication.

## Results

There were 1·04 million (95% UI 0·98–1·10) under-5 deaths in India in 2017, of which 0·57 million (0·54–0·61) were neonatal deaths, down from 2·24 million (2·16–2·32) under-5 deaths including 1·02 million (0·97–1·08) neonatal deaths in 2000. U5MR in India decreased by 49% between 2000 and 2017, from 83·1 (95% UI 76·7–90·1) to 42·4 (36·5–50·0) per 1000 livebirths, and NMR decreased by 38% during this period, from 38·0 (34·2–41·6) to 23·5 (20·1–27·8) per 1000 livebirths (table). Point estimates of the annual rate of change for both U5MR and NMR showed greater reduction during 2010–17 than during 2000–10, although the 95% UIs overlapped and the difference was not significant (table).

**Table 1:** U5MR and NMR in the states of India in 2000, 2010, and 2017

	**U5MR per 1000 livebirths**						**NMR per 1000 livebirths**						**Ratio of NMR to U5MR, 2017**
	2000	2010	2017	Annual rate of change, 2000–10	Annual rate of change, 2010–17	Annual rate of change, 2000–17	2000	2010	2017	Annual rate of change, 2000–10	Annual rate of change, 2010–17	Annual rate of change, 2000–17	
**India (1380 million)**	**83·1 (76·7 to 90·1)**	**58·6 (53·0 to 65·0)**	**42·4 (36·5 to 50·0)**	**−3·43% (−3·71 to −3·17)**	**−4·56% (−6·18 to −2·85)**	**−3·90% (−4·52 to −3·17)**	**38·0 (34·2 to 41·6)**	**29·9 (26·5 to 33·4)**	**23·5 (20·1 to 27·8)**	**−2·38% (−2·71 to −2·08)**	**−3·44% (−5·10 to −1·63)**	**−2·82% (−3·50 to −2·07)**	**0·55**
**Low SDI states (675 million)**													
Bihar	88·8 (81·5 to 96·2)	60·3 (54·8 to 66·1)	43·8 (37·9 to 51·1)	−1·95% (−2·60 to −1·31)	−4·51% (−7·10 to −1·73)	−4·09% (−5·23 to −2·97)	36·5 (31·1 to 41·9)	28·9 (25·2 to 33·0)	23·4 (20·0 to 27·8)	−2·28% (−3·01 to −1·52)	−3·04% (−5·77 to −0·09)	−2·60% (−3·80 to −1·46)	0·53
Madhya Pradesh	110·5 (101·4 to 120·6)	75·4 (68·4 to 82·7)	50·7 (43·9 to 59·2)	−3·75% (−4·22 to −3·24)	−5·56% (−8·07 to −3·21)	−4·50% (−5·56 to −3·46)	48·3 (41·4 to 54·8)	36·8 (31·2 to 42·6)	26·9 (23·0 to 31·7)	−2·69% (−3·25 to −2·09)	−4·44% (−6·96 to −1·98)	−3·42% (−4·55 to −2·39)	0·53
Jharkhand	87·2 (78·4 to 96·0)	60·1 (54·2 to 66·7)	43·9 (37·9 to 51·2)	−3·66% (−4·32 to −3·01)	−4·44% (−6·97 to −1·73)	−3·99% (−5·00 to −2·89)	37·5 (32·4 to 43·0)	28·7 (23·6 to 33·8)	22·9 (19·8 to 26·7)	−2·64% (−3·37 to −1·89)	−3·26% (−5·94 to −0·37)	−2·90% (−3·98 to −1·76)	0·52
Uttar Pradesh	112·8 (101·8 to 125·0)	81·8 (74·3 to 89·7)	59·7 (51·8 to 69·7)	−3·16% (−3·61 to −2·74)	−4·43% (−6·60 to −2·14)	−3·69% (−4·56 to −2·73)	48·2 (41·1 to 55·8)	39·4 (34·4 to 44·5)	31·7 (27·3 to 37·2)	−1·99% (−2·49 to −1·50)	−3·10% (−5·46 to −0·66)	−2·45% (−3·40 to −1·42)	0·53
Rajasthan	90·5 (81·0 to 100·7)	64·9 (59·1 to 71·0)	48·3 (41·8 to 56·4)	−2·85% (−3·73 to −2·00)	−4·19% (−6·53 to −1·84)	−3·65% (−4·59 to −2·69)	40·5 (34·8 to 46·4)	33·2 (29·1 to 37·5)	26·5 (22·8 to 31·0)	−1·97% (−2·60 to −1·30)	−3·21% (−5·70 to −0·92)	−2·49% (−3·51 to −1·49)	0·55
Chhattisgarh	94·7 (85·4 to 104·3)	68·8 (60·8 to 77·8)	47·7 (41·3 to 55·7)	−3·80% (−4·45 to −3·10)	−5·13% (−7·84 to −2·42)	−3·97% (−5·09 to −2·81)	48·2 (42·1 to 55·0)	39·5 (32·9 to 46·3)	29·2 (25·3 to 34·0)	−1·97% (−2·84 to −1·13)	−4·26% (−7·06 to −1·37)	−2·92% (−4·07 to −1·69)	0·61
Odisha	99·1 (90·2 to 108·1)	68·3 (60·6 to 77·6)	49·1 (42·4 to 57·2)	−5·75% (−6·58 to −4·94)	−4·66% (−7·37 to −2·12)	−4·07% (−5·17 to −3·00)	45·3 (39·9 to 51·1)	33·2 (27·7 to 39·0)	25·5 (21·8 to 30·2)	−3·06% (−3·84 to −2·23)	−3·78% (−6·66 to −1·09)	−3·36% (−4·50 to −2·21)	0·52
Assam	87·6 (79·6 to 96·1)	71·9 (63·7 to 81·4)	54·9 (47·5 to 64·0)	−5·82% (−7·02 to −4·46)	−3·83% (−6·53 to −1·11)	−2·73% (−3·81 to −1·66)	43·5 (38·1 to 49·3)	36·4 (30·6 to 42·5)	29·5 (25·1 to 35·0)	−1·77% (−2·54 to −1·00)	−3·00% (−5·87 to −0·38)	−2·28% (−3·40 to −1·13)	0·54
**Middle SDI states (387 million)**													
Andhra Pradesh	81·8 (75·1 to 88·9)	55·3 (44·4 to 67·3)	36·1 (25·9 to 50·8)	−1·69% (−2·52 to −0·84)	−5·97% (−8·77 to −2·83)	−4·73% (−5·99 to −3·46)	37·1 (31·0 to 43·1)	28·4 (22·2 to 35·7)	20·1 (14·2 to 28·3)	−2·64% (−3·84 to −1·48)	−4·90% (−7·86 to −1·61)	−3·58% (−4·91 to −2·25)	0·56
West Bengal	65·2 (59·3 to 71·5)	42·7 (35·1 to 51·0)	29·2 (25·2 to 34·2)	−4·15% (−4·93 to −3·40)	−5·35% (−8·03 to −2·59)	−4·65% (−5·80 to −3·52)	31·9 (28·0 to 36·2)	23·8 (19·0 to 29·2)	17·8 (15·3 to 20·8)	−2·86% (−3·71 to −2·02)	−4·17% (−6·97 to −1·38)	−3·41% (−4·63 to −2·20)	0·61
Tripura	61·2 (55·5 to 67·4)	44·9 (36·8 to 54·8)	34·0 (29·3 to 39·8)	−4·62% (−5·89 to −3·41)	−3·97% (−7·29 to −0·47)	−3·43% (−4·89 to −2·09)	29·9 (25·7 to 34·4)	23·3 (18·1 to 29·6)	18·7 (16·3 to 22·0)	−2·46% (−3·53 to −1·36)	−3·15% (−6·61 to 0·37)	−2·75% (−4·30 to −1·39)	0·55
Arunachal Pradesh	73·0 (65·9 to 80·7)	40·1 (34·8 to 46·1)	27·3 (23·6 to 32·0)	−3·84% (−4·91 to −2·79)	−5·39% (−8·74 to −2·17)	−5·64% (−7·15 to −4·22)	28·7 (24·2 to 33·4)	19·2 (15·8 to 22·9)	14·4 (12·4 to 16·9)	−3·92% (−5·25 to −2·45)	−4·08% (−7·35 to −0·81)	−3·99% (−5·54 to −2·49)	0·53
Meghalaya	66·0 (60·0 to 72·6)	49·2 (42·8 to 56·3)	39·1 (33·7 to 45·6)	−2·90% (−3·67 to −2·16)	−3·29% (−6·20 to −0·29)	−3·06% (−4·27 to −1·84)	24·9 (20·6 to 29·7)	21·5 (17·1 to 26·0)	18·8 (16·1 to 22·1)	−1·43% (−2·28 to −0·59)	−1·98% (−5·01 to 1·21)	−1·66% (−2·88 to −0·36)	0·48
Karnataka	64·9 (59·2 to 70·9)	44·5 (39·1 to 50·4)	31·7 (27·4 to 37·1)	−3·69% (−4·72 to −2·67)	−4·81% (−8·18 to −1·29)	−4·16% (−5·56 to −2·87)	33·0 (29·4 to 36·9)	24·2 (20·5 to 28·3)	18·3 (15·8 to 21·5)	−3·05% (−4·22 to −1·94)	−3·96% (−7·40 to −0·31)	−3·43% (−4·87 to −2·06)	0·58
Telangana	73·0 (65·2 to 80·6)	45·5 (36·3 to 56·2)	28·6 (20·6 to 40·3)	−5·13% (−6·27 to −3·93)	−6·47% (−9·71 to −3·16)	−5·39% (−6·72 to −3·99)	34·4 (29·0 to 39·8)	25·2 (19·1 to 32·5)	17·6 (12·5 to 24·9)	−3·07% (−4·40 to −1·73)	−5·07% (−8·40 to −1·55)	−3·90% (−5·32 to −2·39)	0·62
Gujarat	72·7 (66·0 to 79·9)	49·9 (44·2 to 56·8)	38·1 (33·0 to 44·5)	−3·70% (−4·73 to −2·69)	−3·81% (−6·79 to −0·78)	−3·75% (−4·97 to −2·53)	36·5 (32·2 to 41·0)	28·0 (23·7 to 32·5)	23·1 (19·9 to 27·1)	−2·62% (−3·70 to −1·56)	−2·76% (−5·81 to 0·29)	−2·68% (−3·92 to −1·41)	0·61
Manipur	47·3 (42·5 to 52·3)	31·4 (27·4 to 36·1)	24·9 (21·5 to 29·3)	−4·01% (−5·18 to −2·82)	−3·33% (−6·67 to −0·06)	−3·73% (−5·18 to −2·34)	21·9 (18·5 to 25·5)	16·5 (13·8 to 19·7)	13·8 (12·0 to 16·0)	−2·79% (−4·05 to −1·46)	−2·62% (−6·02 to 0·62)	−2·72% (−4·21 to −1·24)	0·55
Jammu and Kashmir*	57·9 (52·4 to 63·5)	43·5 (38·2 to 49·5)	33·4 (28·9 to 39·0)	−2·81% (−3·97 to −1·63)	−3·75% (−6·64 to −0·96)	−3·20% (−4·48 to −2·09)	27·8 (24·5 to 31·6)	24·0 (20·4 to 27·7)	19·7 (17·0 to 23·0)	−1·46% (−2·77 to −0·08)	−2·85% (−5·72 to −0·03)	−2·04% (−3·37 to −0·80)	0·59
Haryana	73·2 (66·5 to 80·5)	54·6 (48·4 to 62·2)	39·3 (34·0 to 46·0)	−2·88% (−3·62 to −2·12)	−4·63% (−7·21 to −2·04)	−3·61% (−4·73 to −2·57)	30·1 (26·2 to 34·3)	25·6 (21·7 to 30·5)	20·0 (17·2 to 23·5)	−1·60% (−2·41 to −0·77)	−3·52% (−6·18 to −0·76)	−2·40% (−3·53 to −1·33)	0·51
**High SDI states (318 million)**													
Uttarakhand	58·1 (52·1 to 64·5)	43·0 (37·8 to 49·1)	30·3 (26·2 to 35·5)	−2·97% (−3·79 to −2·12)	−4·90% (−7·47 to −2·25)	−3·77% (−4·86 to −2·70)	27·0 (23·4 to 30·6)	22·0 (17·8 to 26·4)	17·1 (14·8 to 20·0)	−2·00% (−2·89 to −1·05)	−3·64% (−6·25 to −0·82)	−2·68% (−3·79 to −1·57)	0·56
Tamil Nadu	51·4 (47·0 to 56·4)	30·3 (25·2 to 36·4)	19·6 (16·9 to 23·0)	−3·47% (−4·72 to −2·20)	−6·13% (−9·90 to −2·31)	−5·55% (−7·06 to −3·93)	25·4 (22·6 to 28·6)	16·8 (13·7 to 20·4)	11·3 (9·7 to 13·3)	−4·08% (−5·41 to −2·89)	−5·52% (−9·37 to −1·80)	−4·68% (−6·33 to −2·96)	0·58
Mizoram	44·0 (39·6 to 48·8)	44·1 (38·5 to 50·5)	36·3 (31·4 to 42·5)	0·02% (−1·14 to 1·12)	−2·81% (−6·10 to 0·54)	−1·16% (−2·55 to 0·24)	19·9 (17·0 to 23·1)	21·1 (17·4 to 24·9)	18·1 (15·6 to 21·3)	0·59% (−0·62 to 1·82)	−2·20% (−5·62 to 1·25)	−0·57% (−2·08 to 0·84)	0·50
Maharashtra	54·0 (49·2 to 59·3)	36·7 (30·4 to 44·0)	26·1 (22·5 to 30·6)	−3·78% (−4·71 to −2·84)	−4·83% (−7·88 to −1·92)	−4·22% (−5·38 to −3·01)	31·1 (27·5 to 34·8)	22·6 (18·6 to 27·0)	17·0 (14·7 to 20·0)	−3·14% (−4·12 to −2·10)	−4·00% (−7·02 to −0·98)	−3·50% (−4·75 to −2·27)	0·65
Punjab	55·6 (50·5 to 61·0)	41·6 (36·6 to 47·4)	29·7 (25·6 to 34·6)	−3·66% (−4·39 to −2·89)	−4·77% (−7·36 to −2·09)	−3·65% (−4·76 to −2·55)	26·3 (23·2 to 29·7)	21·4 (18·2 to 25·0)	16·4 (14·1 to 19·2)	−2·04% (−2·99 to −1·09)	−3·78% (−6·50 to −0·98)	−2·76% (−3·95 to −1·63)	0·55
Sikkim	47·5 (42·3 to 52·8)	33·4 (28·7 to 38·4)	21·9 (18·8 to 25·7)	−3·26% (−3·82 to −2·72)	−5·90% (−8·83 to −2·78)	−4·48% (−5·75 to −3·13)	22·5 (18·7 to 26·8)	17·2 (13·2 to 21·4)	12·1 (10·5 to 14·0)	−2·66% (−4·00 to −1·32)	−4·99% (−7·98 to −1·73)	−3·63% (−4·99 to −2·18)	0·55
Nagaland	60·4 (54·2 to 66·9)	41·8 (36·4 to 47·8)	34·8 (30·0 to 40·6)	−3·62% (−4·65 to −2·50)	−2·66% (−5·78 to 0·33)	−3·23% (−4·54 to −1·97)	25·1 (20·4 to 30·7)	17·9 (14·0 to 22·5)	15·8 (13·7 to 18·6)	−3·30% (−4·51 to −2·01)	−1·84% (−5·13 to 1·26)	−2·71% (−4·09 to −1·36)	0·45
Himachal Pradesh	52·2 (47·1 to 57·6)	40·6 (35·6 to 46·3)	31·4 (27·1 to 36·6)	−2·47% (−3·69 to −1·28)	−3·69% (−6·46 to −0·99)	−2·98% (−4·28 to −1·78)	27·2 (24·0 to 30·6)	22·8 (19·0 to 26·9)	18·6 (16·1 to 21·7)	−1·78% (−3·07 to −0·50)	−2·92% (−5·81 to −0·12)	−2·25% (−3·61 to −1·03)	0·59
Union Territories other than Delhi	35·2 (29·7 to 41·4)	29·7 (24·5 to 36·1)	22·9 (19·7 to 26·9)	−3·05% (−4·01 to −2·00)	−3·68% (−5·77 to −1·53)	−2·52% (−3·45 to −1·49)	19·7 (16·4 to 23·3)	17·7 (14·0 to 21·8)	14·1 (12·2 to 16·7)	−1·07% (−1·99 to −0·14)	−3·15% (−5·28 to −0·90)	−1·93% (−2·89 to −0·88)	0·62
Kerala	19·9 (17·5 to 22·6)	12·9 (11·1 to 15·0)	10·4 (9·0 to 12·2)	−4·26% (−5·81 to −2·64)	−3·10% (−7·16 to 1·36)	−3·79% (−5·71 to −1·92)	12·9 (11·3 to 14·7)	8·7 (7·3 to 10·2)	7·1 (6·1 to 8·4)	−3·95% (−5·65 to −2·22)	−2·81% (−6·97 to 1·76)	−3·49% (−5·34 to −1·59)	0·68
Delhi	57·5 (52·0 to 63·2)	31·8 (26·6 to 38·3)	25·1 (21·7 to 29·4)	−3·15% (−3·93 to −2·43)	−3·38% (−6·12 to −0·58)	−4·78% (−5·97 to −3·70)	31·1 (26·4 to 35·7)	19·5 (15·8 to 23·9)	16·1 (13·9 to 18·8)	−4·55% (−5·44 to −3·64)	−2·79% (−5·63 to 0·05)	−3·83% (−5·06 to −2·73)	0·64
Goa	35·2 (30·8 to 40·4)	24·2 (20·2 to 28·5)	18·8 (13·5 to 26·6)	−3·69% (−5·54 to −1·82)	−3·66% (−8·05 to 0·51)	−3·69% (−5·58 to −1·76)	19·5 (16·8 to 22·6)	13·9 (11·1 to 17·0)	11·0 (7·8 to 15·6)	−3·31% (−5·34 to −1·26)	−3·50% (−7·90 to 0·83)	−3·40% (−5·43 to −1·39)	0·58

Data in parentheses are 95% uncertainty intervals; the population of each state SDI group in 2017 is shown in parentheses. States are listed in increasing order of SDI in 2017. U5MR=under-5 mortality rate. NMR=neonatal mortality rate. SDI=Socio-demographic Index.

* The state of Jammu and Kashmir was divided into two union territories in August, 2019; as we are reporting findings up to 2017, we report findings for the undivided state of Jammu and Kashmir.

A significant inverse correlation was observed between the SDI of states and U5MR (*r*= −0·77; p<0·0001) and NMR (*r* = −0·76; p<0·0001) in 2017. U5MR varied 5·7 times between the states, ranging from 10·4 (95% UI 9·0 to 12·2) in Kerala to 59·7 (51·8 to 69·7) in Uttar Pradesh, and NMR varied 4·5 times, ranging from 7·1 (6·1 to 8·4) in Kerala to 31·7 (27·3 to 37·2) in Uttar Pradesh (table). The annual rate of change from 2010 to 2017 for U5MR ranged among the states from a 2·66% (95% UI −0·33 to 5·78) reduction in Nagaland to a 6·47% (3·16 to 9·71) reduction in Telangana, and for NMR ranged from a 1·84% (−1·26 to 5·13) reduction in Nagaland to a 5·52% (1·80 to 9·37) reduction in Tamil Nadu (table; appendix p 98). Among the low SDI states, Assam had the lowest annual rate of reduction from 2010 to 2017 and Madhya Pradesh the highest (table). In the middle SDI states, Andhra Pradesh and Tripura had a similar U5MR in 2017, but quite different annual rates of reduction from 2010 to 2017 (5·97% and 3·97%, respectively; table). Likewise, among the high SDI states, Tamil Nadu had an annual rate of reduction of 6·13% and Goa of 3·66% during 2010–17, with both having a similar U5MR in 2017. The annual rate of reduction of NMR was lower than that of U5MR in all states during 2010–17, but varied considerably between the states (table; appendix p 98). The ratio of NMR to U5MR reduction was lowest in Meghalaya (0·60), Bihar (0·67), Nagaland (0·69), and Uttar Pradesh (0·70), and highest in the high SDI states of Goa (0·96), Kerala (0·91), and Tamil Nadu (0·90). The number of under-5 and neonatal deaths in 2017 was highest in the state of Uttar Pradesh, followed by Bihar (appendix p 99).

If the U5MR trends estimated up to 2017 were to continue, the projected U5MR for India would be 29·8 (95% UI 25·1–36·0) per 1000 livebirths in 2025, which would be higher than the NHP 2025 target of 23, and 23·2 (18·9–29·0) in 2030, which would be lower than the SDG 2030 target of 25 (appendix p 100). 17 of the 31 states would need a higher rate of improvement than they had up to 2017 to individually achieve the NHP U5MR target, and seven of the 31 states would need a higher rate of improvement to individually achieve the SDG U5MR target (appendix p 100). If the NMR trends up to 2017 were to continue, the projected NMR for India would be 17·6 (14·7–21·4) and 14·7 (11·8–18·4) per 1000 livebirths in 2025 and 2030, respectively (appendix p 100), both higher than the respective NHP (16 per 1000 livebirths) and SDG (12 per 1000 livebirths) targets. To reach the NHP and SDG targets for NMR individually, 12 and 15 of the 31 states, respectively, would need a rate of improvement higher than they had up to 2017 (appendix p 100).

**Figure 1:**
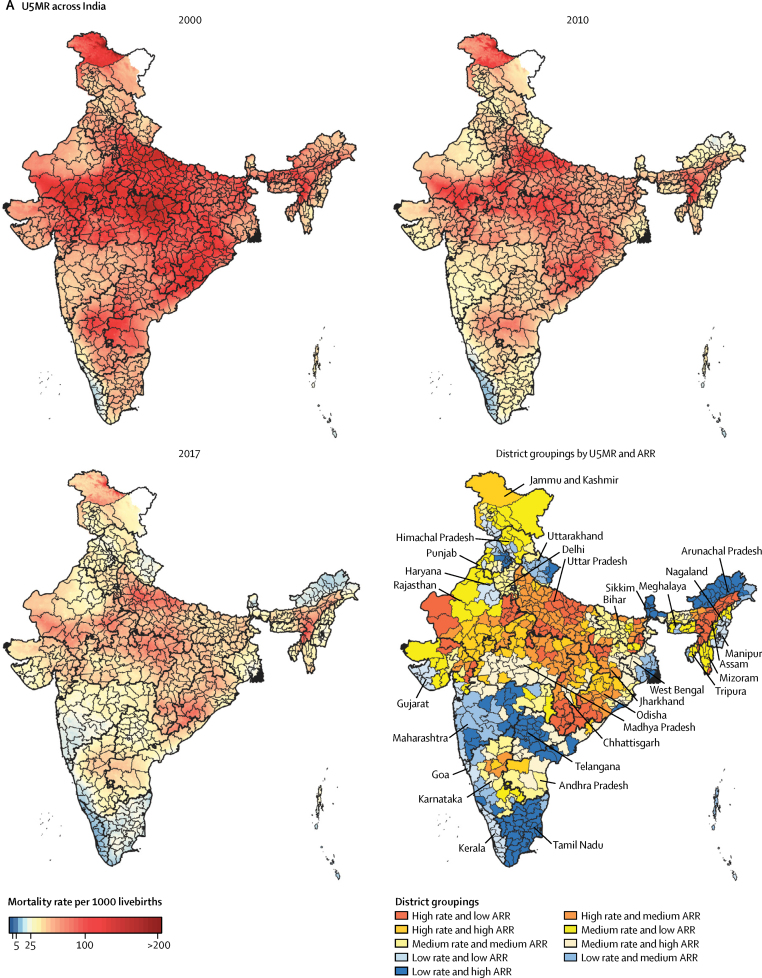

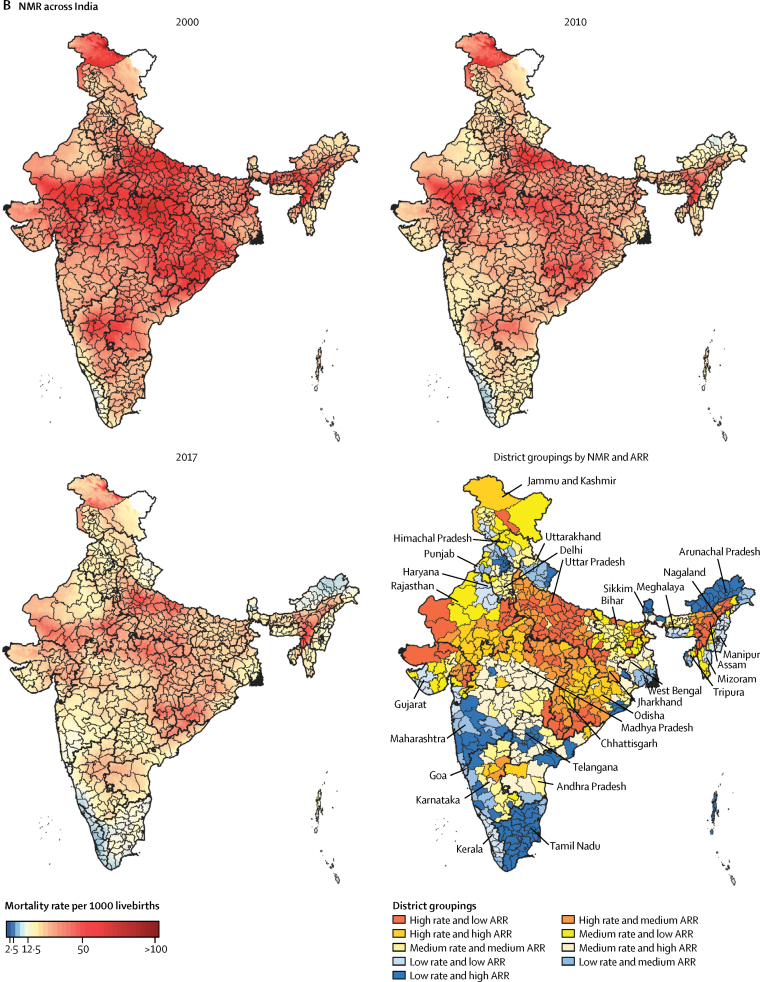
U5MR (A) and NMR (B) mapping in India U5MR and NMR in the years 2000, 2010, and 2017, and district groupings of U5MR and NMR according to district-level rates in 2017 against the ARR from 2010 to 2017. High, medium, and low groupings are based on tertiles of U5MR, NMR, and ARR. The tertile cutoffs for U5MR were 30·9 and 45·2 and for U5MR ARR 4·15% and 5·12%. The tertile cutoffs for NMR were 17·6 and 24·6 and for NMR ARR 3·04% and 4·11%. U5MR=under-5 mortality rate. NMR=neonatal mortality rate. ARR=annual rate of reduction.

At the district level, U5MR varied 10·5 times between the 723 districts of India in 2017, ranging from 8·4 (95% UI 5·6–12·1) to 87·9 (72·2–106·4) per 1000 livebirths (figure 1; appendix pp 101–109). U5MR was 40 or more per 1000 livebirths in 275 (88%) of the 312 districts in the low SDI states, in 41 (18%) of the 233 districts in the middle SDI states, and in four (2%) of the 176 districts in the high SDI states (appendix pp 101–109). The district-level annual rate of reduction in U5MR from 2010 to 2017 ranged from 9·02% (6·30–11·63) to no significant change (appendix pp 101–109). The median annual rate of reduction of U5MR from 2010 to 2017 was 4·56% (IQR 4·09–5·22) in districts in the low SDI states, 4·87% (3·77–5·74) in the middle SDI states, and 4·63% (3·42–5·42) in the high SDI states (appendix pp 101–109).

NMR varied 8·0 times between the districts in India in 2017, with a range from 5·8 (95% UI 3·9–8·2) to 46·2 (37·5–56·6) per 1000 livebirths (figure 1; appendix pp 109–118). The districts with the highest and lowest NMR coincided with the districts with the highest and lowest U5MR (appendix pp 101–118). NMR was 20 or more in 291 (93%) of the 312 districts in the low SDI states, in 81 (35%) of the 233 districts in the middle SDI states, and in 23 (13%) of the 176 districts in the high SDI states (appendix pp 109–118). The district-level annual rate of reduction in NMR from 2010 to 2017 ranged from 8·05% (95% UI 5·34–10·74) to no significant change (appendix pp 109–118). The median annual rate of reduction of NMR from 2010 to 2017 was 3·32% (IQR 2·18–4·13) in districts in the low SDI states, 3·78% (2·75–4·55) in the middle SDI states, and 3·66% (2·83–4·61) in the high SDI states.

**Figure 2:**
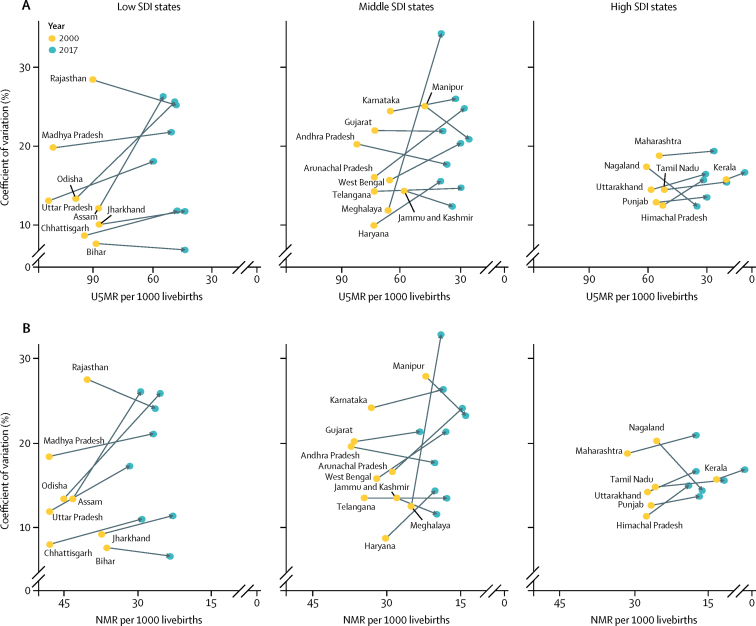
Coefficient of variation for U5MR (A) and NMR (B) between districts within the states of India, 2000 and 2017 Higher coefficient of variation indicates higher inequality between the districts. Data are shown for states with more than ten districts. U5MR=under-5 mortality rate. NMR=neonatal mortality rate. SDI=Socio-demographic Index.

Inequality between the districts within the states varied widely in 2017 for both U5MR and NMR, with the CV varying 10·7 times for U5MR and 13·2 times for NMR (figure 2; appendix p 119). Inequality increased from 2000 to 2017 for U5MR in 23 states and for NMR in 24 states, with generally a similar pattern for the two mortality indicators (figure 2; appendix p 119). Among the low SDI states, the highest increases in CV were in Assam and Odisha, and among the middle SDI states, the highest increases in CV were in the small northeastern states of Meghalaya and Arunachal Pradesh and in Haryana (appendix p 119). The time trends for inequality among the states were quite variable, even within the same SDI state groups (figure 2).

**Figure 3:**
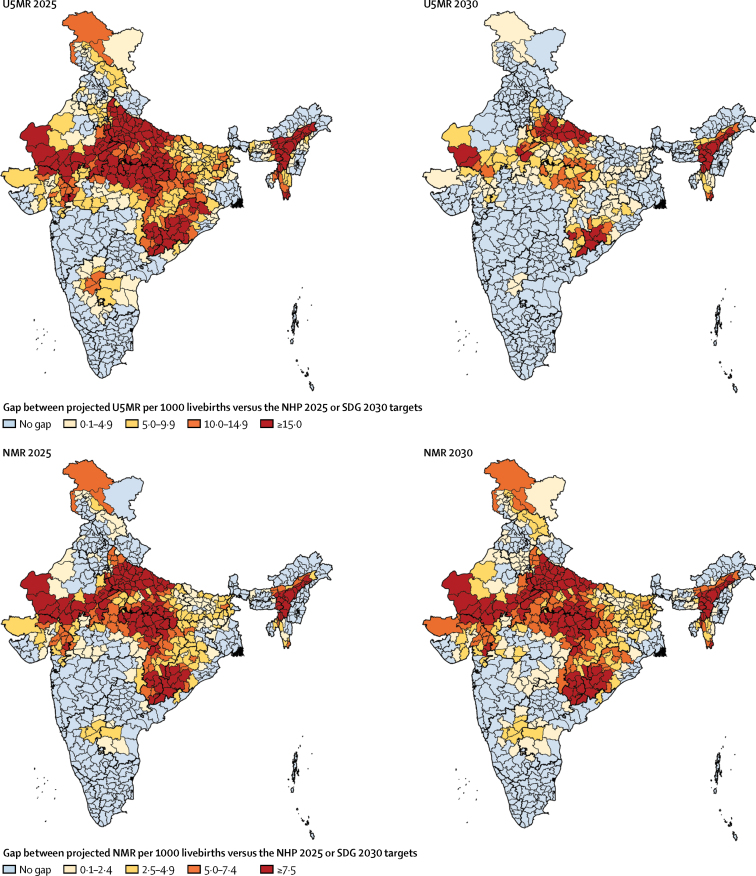
Gap between the projected U5MR and NMR in the districts of India in 2025 and 2030 based on trends from 2000 to 2017 versus the NHP 2025 targets and SDG 2030 targets U5MR=under-5 mortality rate. NMR=neonatal mortality rate. NHP=National Health Policy. SDG=Sustainable Development Goals.

Of the 723 districts in India, 455 (63%) would need a rate of improvement higher than they had up to 2017 to individually meet the U5MR NHP 2025 target and 246 (34%) to meet the SDG 2030 target (figure 3; appendix pp 120–133). 353 (49%) districts would need a higher rate of improvement than they had up to 2017 to individually meet the NMR NHP 2025 target and 430 (59%) districts would need a higher rate of improvement to reach the SDG 2030 NMR target (figure 3); this proportion was 91%, 47%, and 21% in the low, middle, and high SDI states, respectively (appendix pp 120–133).

Identification of priority districts based on tertiles of the state distribution of district-level U5MR and NMR in 2017 and tertiles of their annual rate of reduction from 2010 to 2017 is described in the appendix for 18 states with 20 or more districts (pp 134–153). As an example, in Uttar Pradesh, which is the most populous state of India and had the highest NMR in 2017, NMR ranged from 22·8 (95% UI 17·9 to 28·7) to 46·2 (37·5 to 56·6) per 1000 livebirths in 2017 among the districts and the annual rate of reduction from 2010 to 2017 ranged from 1·64% (−1·16 to 4·84) to 4·46% (1·35 to 7·59; appendix p 111). A cluster of eight districts in the north-central part, a cluster of two districts in the south, and one district in the southwest corner of the state fell into the worst category of high NMR and low rate of reduction, which would need the highest priority (appendix pp 134–136). The highest priority districts for U5MR in Uttar Pradesh with this approach were similar for NMR, but one more district in the south also fell into this category. Examination of the trends in the districts of Uttar Pradesh with respect to the nationwide district-level distribution of NMR in 2017 and the annual rate of reduction from 2010 to 2017, however, revealed that 68 (91%) of the 75 districts in this state were in the high tertile for NMR and only three (4%) districts were in the high tertile for the annual rate of reduction, resulting in 36 (48%) districts falling into the worst category of high NMR and low annual rate of reduction (figure 1; appendix pp 134–135). Considering all low SDI states together, 208 (67%) of the total 312 districts fell into the high nationwide tertile for NMR and 213 (68%) districts for U5MR.

**Figure 4:**
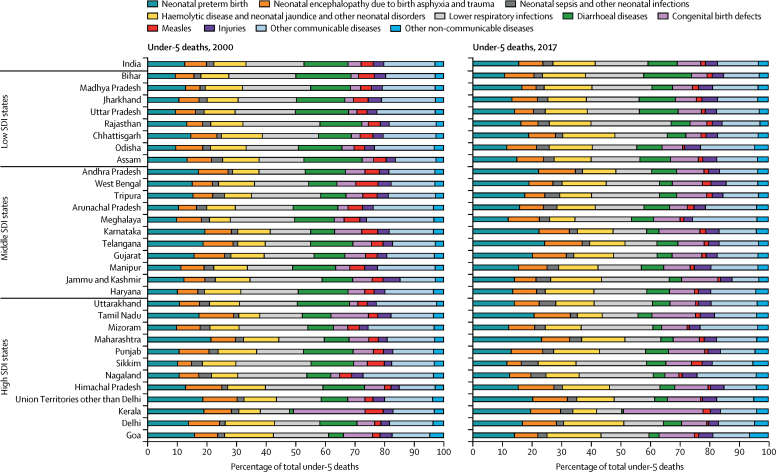

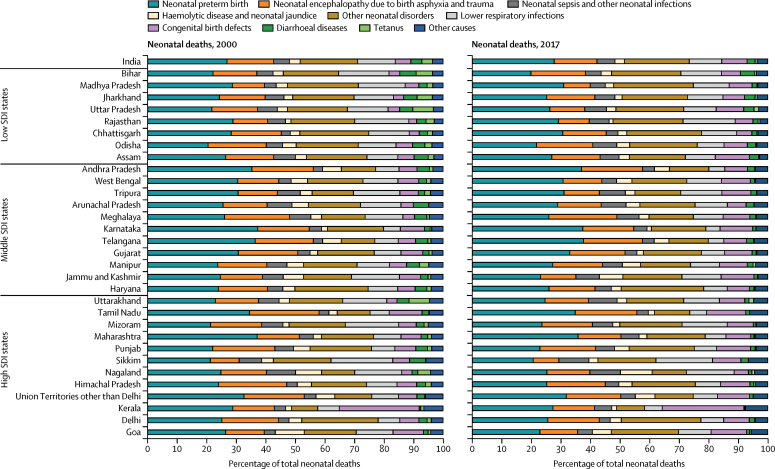
Major causes of under-5 and neonatal death in the states of India, 2000 and 2017 Specific causes shown are those that contributed 3% or more of the deaths in 2000 or 2017. Haemolytic disease and neonatal jaundice contributed more than 3% of neonatal deaths but less than 3% of under-5 deaths, and so was added to the other neonatal disorders category for under-5 deaths. For under-5 deaths, the remaining causes were aggregated under injuries, other communicable diseases, and other non-communicable diseases. For neonatal deaths, all of the remaining causes were aggregated under other causes as their proportion was very low. SDI=Socio-demographic Index.

The causes of under-5 deaths in India in 2017 were lower respiratory infections (17·9%), neonatal preterm birth (15·6%), haemolytic disease and neonatal jaundice and other neonatal disorders (14·3%), diarrhoeal diseases (9·9%), neonatal encephalopathy due to birth asphyxia and trauma (8·1%), congenital birth defects (8·0%), injuries (4·1%), neonatal sepsis and other neonatal infections (3·5%), measles (1·6%), other communicable diseases (13·8%), and other non-communicable diseases (3·4%; figure 4; appendix p 154). In 2017, the cause-fractions of under-5 deaths were higher for lower respiratory infections and diarrhoeal diseases but lower for preterm birth, neonatal encephalopathy due to birth asphyxia and trauma, and congenital birth defects in the low SDI state group than in the middle and high SDI groups (appendix p 154). However, there were wide variations for the cause-fractions in the states within the SDI groups as well. The death rate for most causes of under-5 death in 2017 had a significant inverse correlation with increasing levels of SDI, except for congenital birth defects, other communicable disease, and other non-communicable disease. This inverse correlation was the strongest for measles (*r*=−0·76; p<0·0001) and diarrhoeal diseases (*r* =−0·72; p<0·0001; appendix p 155). The U5MR reduced for all categories of causes in India as a whole from 2000 to 2017, with the highest reduction in death rate for infectious diseases, intermediate reduction for neonatal disorders, and smallest reduction for congenital birth defects (appendix pp 156–157). For the specific causes presented, the percentage reduction was highest for measles (81·9%, 95% UI 76·9–86·2), followed by diarrhoeal diseases (68·7%, 60·7–75·5), and lower respiratory infections (57·2%, 51·6–61·7), and least for congenital birth defects (15·1%, 3·9–32·2; appendix pp 156–157). However, there were wide variations between the states, even within the same SDI group (appendix pp 156–157).

The causes of neonatal death in India in 2017 were neonatal preterm birth (27·7%), neonatal encephalopathy due to birth asphyxia and trauma (14·5%), lower respiratory infections (11·0%), congenital birth defects (8·6%), neonatal sepsis and other neonatal infections (6·1%), haemolytic disease and neonatal jaundice (3·2%), diarrhoeal diseases (2·7%), tetanus (0·7%), other neonatal disorders (22·0%), and other causes (3·5%; figure 4; appendix p 158). As for under-5 deaths, the cause-fractions for neonatal deaths also varied considerably between the states (figure 4; appendix p 158). In India in 2017, 79·5% of neonatal deaths were in the early neonatal period of 0–6 days, and this varied from 69·6% to 84·9% between the states (appendix p 159). The proportion of deaths due to preterm birth, neonatal encephalopathy due to birth asphyxia and trauma, and other neonatal disorders was higher in the early than in the late neonatal period (appendix p 160).

**Figure 5:**
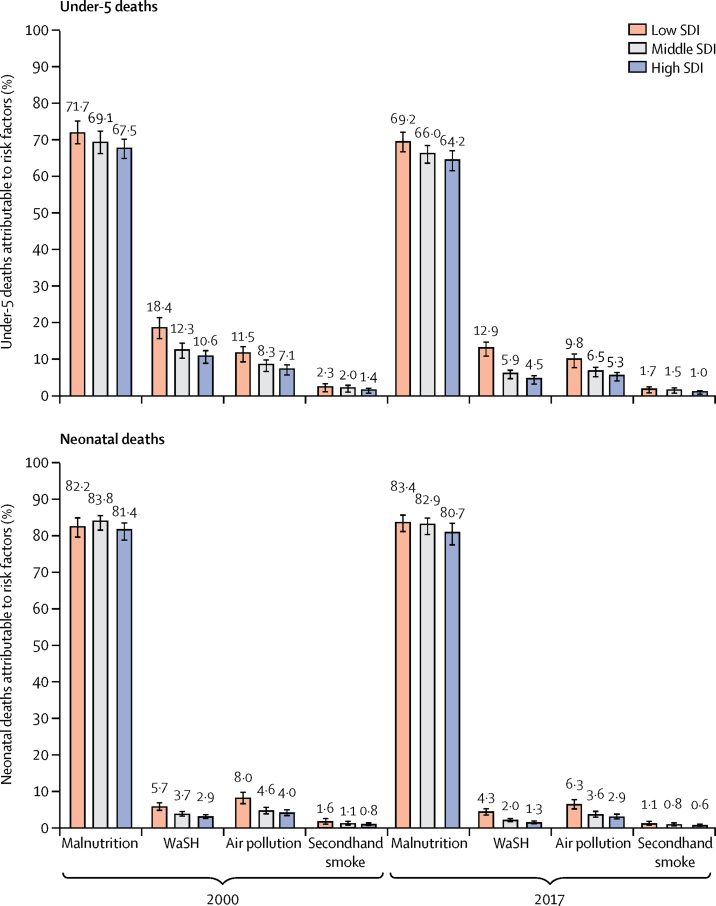
Risk factors for under-5 and neonatal death by SDI state groups in India, 2000 and 2017 Error bars show 95% uncertainty intervals. SDI=Socio-demographic Index. WaSH=water, sanitation, and handwashing.

The dominant risk factor for under-5 death was child and maternal malnutrition, to which 68·2% (95% UI 65·8–70·7) of all child deaths in India could be attributed in 2017, with the largest contributor to this being low birthweight and short gestation (45·9%, 95% UI 44·4–47·5) followed by child growth failure (21·4%, 19·5–23·2). 10·8% (9·1–12·4) of under-5 deaths could be attributed to unsafe water (8·0%, 5·2–9·9) and sanitation (4·8%, 3·8–5·9) and 8·8% (7·0–10·3) to air pollution (appendix p 161). The point estimates for the proportion of under-5 deaths attributable to child and maternal malnutrition, unsafe water and sanitation, and air pollution were highest for the low SDI state group, and the difference with the other SDI state groups was most prominent for unsafe water and sanitation and air pollution (figure 5). The proportion of under-5 deaths attributable to child and maternal malnutrition ranged from 62·1% (57·2–67·0) to 72·7% (69·0–76·0) in the low SDI state group, 59·1% (53·1–65·7) to 67·9% (64·4–71·1) in the middle SDI state group, and 50·8% (46·0–56·6) to 68·5% (63·5–72·5) in the high SDI state group. The proportion of under-5 deaths attributable to unsafe water and sanitation varied between the states from 1·2% (0·8–1·6) to 14·2% (11·4–16·9), and for air pollution from 2·2% (1·6–2·9) to 13·6% (10·5–16·3; appendix p 161). For neonatal deaths, child and maternal malnutrition was the predominant risk factor to which 83·0% (80·6–85·0) of deaths could be attributed, almost all of which were due to low birthweight and short gestation (82·8%, 77·6–88·4; appendix p 162). Estimates for the contribution of risk factors to neonatal deaths were higher in the low than in the middle and high SDI state groups, as for under-5 (figure 5; appendix p 162).

## Discussion

India has made major progress in child mortality, with U5MR dropping by 49% and NMR by 38% from 2000 to 2017. Despite the overall notable gains in child survival, there are wide variations in U5MR and NMR and their rates of improvement between the states and even more so between the districts of India. The U5MR and NMR estimates for all districts of India in this Article, based on comprehensive geospatial analysis using all accessible data sources, highlight the enduring disparities in child survival across the country. These findings underscore the need for tracking local granular patterns in child survival in order to achieve the Indian and SDG targets for child mortality.

Quantification of child mortality levels and trends at the district level provides important insights into child health inequalities within states and across the country. The variations of child mortality rates among the districts of India are dramatic, with U5MR varying by ten times and NMR by eight times between the 723 districts of India in 2017. The highest district-level U5MR and NMR in India in 2017 were comparable to the highest rate globally among some countries in sub-Saharan Africa.^28^ Although U5MR and NMR decreased in almost all districts of India between 2000 and 2017, inequality in these rates, as measured by the CV, increased between districts in a large proportion of the states. This highlights the need to identify districts that continue to have high mortality rates and low rates of reduction. It is useful to note that the distribution of the district-level rate of decline for U5MR and NMR was not significantly different between the three SDI state groups, and that a large proportion of the districts in most of the low SDI states fall in the high tertile group of U5MR and NMR for the nationwide distribution.

If the child mortality trends observed up to 2017 were to continue, India would achieve the SDG 2030 U5MR target of 25 per 1000 livebirths, but not the SDG NMR target of 12 per 1000 livebirths, nor either of the NHP 2025 targets for U5MR and NMR. Interestingly, the India target for U5MR is stricter than the SDG target, but the India NMR target is less strict than the SDG target. The government has also set a target of fewer than ten deaths for NMR by 2030 under the India Newborn Action Plan, which is more ambitious. The majority of the districts in the low SDI states would need acceleration of mortality reduction rate to meet the NHP 2025 and SDG 2030 targets for child mortality individually, as well as some districts in the other states.

Importantly, in 2017, the under-5 death cause-fractions for lower respiratory infections and diarrhoeal diseases were higher in the low SDI state group than in the middle and high SDI state groups, whereas those for preterm birth, neonatal encephalopathy due to birth asphyxia and trauma, and congenital birth defects were lower in this group, but the death rate for all causes was higher in the low SDI state group than in the other SDI state groups. The cause-fractions in combination with the trends in cause-specific death rate decline as described for each state in this Article can guide the relative effort needed to deal with particular causes of under-5 deaths in each state. The under-5 cause-specific death rate for all major causes reduced in India from 2000 to 2017, with the highest decline for infectious diseases, followed by neonatal disorders, and least for congenital birth defects. There were wide variations between the states in the rate of decline for different causes. A previous analysis using SRS data has reported an increase in the death rate from prematurity and low birthweight in India from 2000 to 2015.^17^ We, however, estimated a decrease in the death rate from preterm birth in India from 2000 to 2017, as did another recent report.^18^ This difference is probably due to different analytical approaches, with the previous study using the SRS data directly and our study using all relevant accessible data sources on mortality, causes of death, and risk factors in India including SRS and other sources in a single framework; this approach leads to an integrated analysis that enables balancing of incongruous trends that might be seen with isolated analysis of single data sources. Furthermore, prevalence of low birthweight in India has been declining modestly.^17^ This reduction, along with the generally improving health care in India, would be expected to lead to a decline in the death rate due to preterm birth rather than an increase. In any case, the cause of death reporting system in India needs to be strengthened so that it is based predominantly on medically certified causes of death instead of being based mostly on verbal autopsy data as is currently the case.

The relatively lower decline in NMR compared with U5MR points to the need for more focus on neonatal causes of death, particularly in the early neonatal period, which accounted for nearly 80% of neonatal deaths in India in 2017. A recent study from the Indian state of Bihar, with one of the highest burden of neonatal deaths, has highlighted that the causes of death and their determinants at 0–2 days are different from those between 3–7 days, and that the distribution in the latter is similar to those in deaths at 8–27 days.^29^ Given this emerging evidence, it could be useful to monitor neonatal mortality at 0–2 and 3–7 days separately to enable more effective programming to reduce neonatal mortality.

The inclusion of risk factor analysis for child mortality in this Article provides a useful, broader perspective on how to address child mortality. Malnutrition, by far, outweighs all of the other risk factors for child mortality in every state of India, with low birthweight and short gestation and child growth failure the largest components of this risk factor.^30^ We have recently discussed the burden of malnutrition across the states of India in relation to the National Nutrition Mission, which is a major recent initiative that aims to coordinate and boost a variety of programmes and activities aimed at improving the nutritional status of children and women.^30^, ^31^, ^32^ Low birthweight needs particular attention to reduce child mortality, as more than a fifth of children born in India have low birthweight and its rate of reduction over time has been modest. We report district-level trends of child growth failure in a companion paper, which provides further nuanced insights for policy and programmatic actions aimed at reducing this aspect of child malnutrition.^33^ It should be possible to reduce child mortality substantially with more effective improvements in maternal and child malnutrition across India, which is being attempted by the National Nutrition Mission.^31^, ^32^ The other risk factors to which under-5 deaths could be attributed in India are unsafe water and sanitation, air pollution, and secondhand smoke from tobacco use. There is interaction between malnutrition and unsafe water and sanitation, and the substantial recent improvements in sanitation in India through the Swachh Bharat Mission are expected to also contribute to the reduction of malnutrition.^30^, ^34^

In 2005, India embarked on the National Rural Health Mission to strengthen the public health system, and since then, has launched several initiatives to improve newborn and child health.^35^ The Integrated Child Development Scheme to address malnutrition has been in place since 1975 and was gradually expanded to cover the entire country.^36^ A major review undertaken about a decade ago concluded that although these programmes and relevant policies included useful interventions across the life-cycle and service-delivery continuum, the coverage of interventions was modest and several inadequacies were highlighted.^37^ Furthermore, a global review of maternal, newborn, and child health interventions revealed that the coverage of interventions had increased slowly until 2011, except for malaria interventions, and predicted that substantial reductions in child deaths were possible only if the efforts to achieve intervention coverage are intensified.^38^ Although the coverage of maternal and child health interventions has increased in India, wide subnational variations and inequity based on socioeconomic indicators persist.^39^, ^40^, ^41^, ^42^, ^43^ Reducing the geographical inequities in child mortality will require addressing the geographical inequities in maternal and child health interventions and in the broader social determinants of health at the district level.^12^, ^15^, ^44^ Several cost-effective interventions across the continuum of maternal, newborn, and child health care have been identified that could address child mortality, which include adequate antenatal care, management of labour and delivery, care of preterm births, and treatment of serious infectious diseases and acute malnutrition.^45^, ^46^, ^47^, ^48^, ^49^, ^50^, ^51^ Many of these interventions are part of the India National Newborn Action Plan and Ayushman Bharat, but would need to address the human resources, infrastructure, governance, information, and monitoring of bottlenecks in delivery of these interventions.^10^, ^52^ Only a few high-impact solutions are currently available for preterm birth prevention, and further programmatic action is needed to improve survival and reduce disability in these babies, which is feasible through antenatal steroids and kangaroo mother care.^53^ The new WHO guidelines on antibiotic management of neonatal infections based on the results of the Simplified Antibiotic Therapy Trial could further encourage community treatment and reduce mortality from neonatal infections.^54^, ^55^

There is increasing global evidence that poor-quality health care is a major driver of excess mortality across conditions, including neonatal mortality.^56^ Poor-quality health care across the continuum of care from pregnancy to delivery has also been reported from India.^29^, ^57^, ^58^, ^59^, ^60^, ^61^, ^62^, ^63^, ^64^, ^65^, ^66^, ^67^, ^68^, ^69^ For example, the Janani Suraksha Yojana programme in India, the world's largest demand-side financial incentive programme that provided cash incentives for women to deliver in health facilities, is reported to have significantly increased coverage of facility births but with variable improvements in maternal and newborn survival, as many births occur in facilities that do not have sufficiently skilled staff to address maternal and newborn complications.^56^, ^70^, ^71^, ^72^ For India to continue to reduce child mortality rapidly, it is necessary that the continued priority and expansion of child health interventions distinctly adds quality to quantity.^72^ Some effort in this regard is being made under the LaQshya Initiative to improve quality of care during delivery and immediate post-partum period in public sector facilities.^73^

The limitations of child mortality mapping and the estimation of causes of death and risk factors are described elsewhere.^1^, ^7^ The modelling approaches for fine-grid mapping were limited by the absence of high-resolution spatial data on the full universe of potential covariates of child mortality. A large amount of polygon data were included in our spatially continuous models for which we resampled polygon data to points. This could have introduced over-smoothing, although these effects are probably minimal given their agreement with other subnational mortality models. Also, the majority of polygon data were at the district level and, as we report mortality estimates mainly for districts, this might not be a major limitation, although the uncertainty range of our estimates for the rate of change in recent years were relatively high. Future availability of more geo-referenced data across the surveys and censuses would strengthen the estimation and monitoring of child mortality at the sub-state level. Another limitation is that we mapped mortality for both sexes together, which masks differences between boys and girls. A major limitation for the cause of death assessment in India is an incomplete medically certified cause of death system that covers only a small proportion of the deaths and has variable coverage across the states. Improvements in the medically certified cause of death system and availability of geo-referenced cause of death data at the population level would enable a more robust and granular understanding of the distribution of child death causes in India. Inclusion of cause of death data in the Maternal and Child Health Tracking System that is being used in India would also be useful. Regarding the estimation of malnutrition, the major risk factor for child death, data on low birthweight are relatively weak in India although data on child growth failure are quite extensive.^30^ A major strength of the findings in this Article is the estimation of granular child mortality trends across all districts of the country combined with estimation of causes of death and risk factors at the state level, using all accessible data sources from India in a single framework. Substantial inputs from leading child health experts in India on the analysis and interpretation of findings is another clear strength of this study.

Sustaining and expanding the gains achieved in child survival is a major global agenda, in which India has an important role to play. Regular surveillance of U5MR and NMR and availability of cause of death data at the district level will be crucial for understanding whether policies to counteract inequalities in child mortality are being successful. Our results on district-level variations in the magnitude of U5MR and NMR and in their rate of decline, the estimation of additional effort needed in each district to reach the Indian and SDG targets for U5MR and NMR, along with the distribution of causes of death and risk factors in each state probably provide the most comprehensive and consolidated understanding so far of child mortality trends across India. These findings offer valuable information that could guide further efforts of the Indian and state governments and other stakeholders to improve child survival.
