# Supplementary material for: Subnational mapping of under-5 and neonatal mortality trends in India: the Global Burden of Disease Study 2000–17
Source: Lancet. 2020 May 23;395(10237):1640–58. doi: 10.1016/S0140-6736(20)30471-2 (PMC7262604; doi:10.1016/S0140-6736(20)30471-2)
Supplement: Supplementary appendix [file mmc1.pdf]

THE LANCET

Supplementary appendix

This appendix formed part of the original submission and has been peer reviewed. We post it as supplied by the authors.

Supplement to: India State-Level Disease Burden Initiative Child Mortality Collaborators. Subnational mapping of under-5 and neonatal mortality trends in India: the Global Burden of Disease Study 2000–17. *Lancet* 2020; published online May 12. https://doi.org/10.1016/S0140-6736(20)30471-2.

Subnational mapping of under-5 and neonatal mortality trends in India: the Global Burden of Disease Study 2000–2017

India State-Level Disease Burden Initiative Child Mortality Collaborators

Web Appendix

Correspondence to: Prof. Lalit Dandona, lalit.dandona@icmr.gov.in

Table of contents

1. Child mortality estimation methods 3
2. Cause of death estimation methods 31
3. Risk factors estimation methods 46
4. Data inputs for child mortality, cause of death, risk factors and covariates for India 68
5. Grouping of the states of India based on SDI, 2017 83
6. IMR in the states and districts of India in 2000, 2010 and 2017 84
7. District-level U5MR and NMR against the annual rate of change in the three SDI state groups 98
8. Number of under-5 and neonatal deaths in the states of India, 2017 99
9. Projected U5MR and NMR for the states of India in 2025 and 2030 based on trends from 2000 to 2017 100
10. U5MR and NMR in the districts of India in 2000, 2010 and 2017 101
11. Coefficient of variation for U5MR and NMR between districts within the states of India, 2000 and 2017 119
12. Projected U5MR and NMR for the districts of India in 2025 and 2030 based on trends from 2000 to 2017 120
13. Identification of priority districts in the states based on tertile groupings of the district-level rates of child mortality in 2017 against the annual rate of their reduction from 2010 to 2017 134
14. Causes of under-5 death in the states of India, 2000 and 2017 154
15. Correlation between SDI of the states and cause-specific under-5 death rates, 2017 155
16. Cause-specific under-5 death rates in the states of India, 2000 and 2017 156
17. Causes of neonatal death in the states of India, 2000 and 2017 158
18. Proportion of neonatal deaths in early and late neonatal periods in the states of India, 2017 159
19. Causes of early and late neonatal death in the states of India, 2017 160
20. Risk factors for under-5 death in the states of India, 2017 161
21. Risk factors for neonatal death in the states of India, 2017 162

1. Child mortality estimation methods

The materials presented here are adapted from the following source:

- GBD 2017 Mortality Collaborators. Global, regional, and national age-sex-specific mortality and life expectancy, 1950–2017: a systematic analysis for the Global Burden of Disease Study 2017. *Lancet* 2018; 392: 1684–735.
- Local Burden of Disease Under-five Mortality Collaborators. Mapping 123 million neonatal, infant, and child deaths between 2000 and 2017. *Nature* 2019; 574, 353–358.
- GBD 2017 SDG Collaborators. Measuring progress from 1990 to 2017 and projecting attainment to 2030 of the health-related Sustainable Development Goals for 195 countries and territories: a systematic analysis for the Global Burden of Disease Study 2017. *Lancet* 2018; 392: 2091–138.

A. GBD data and analysis framework

The overview of data inputs and analysis framework for GBD is shown in the following flowchart:

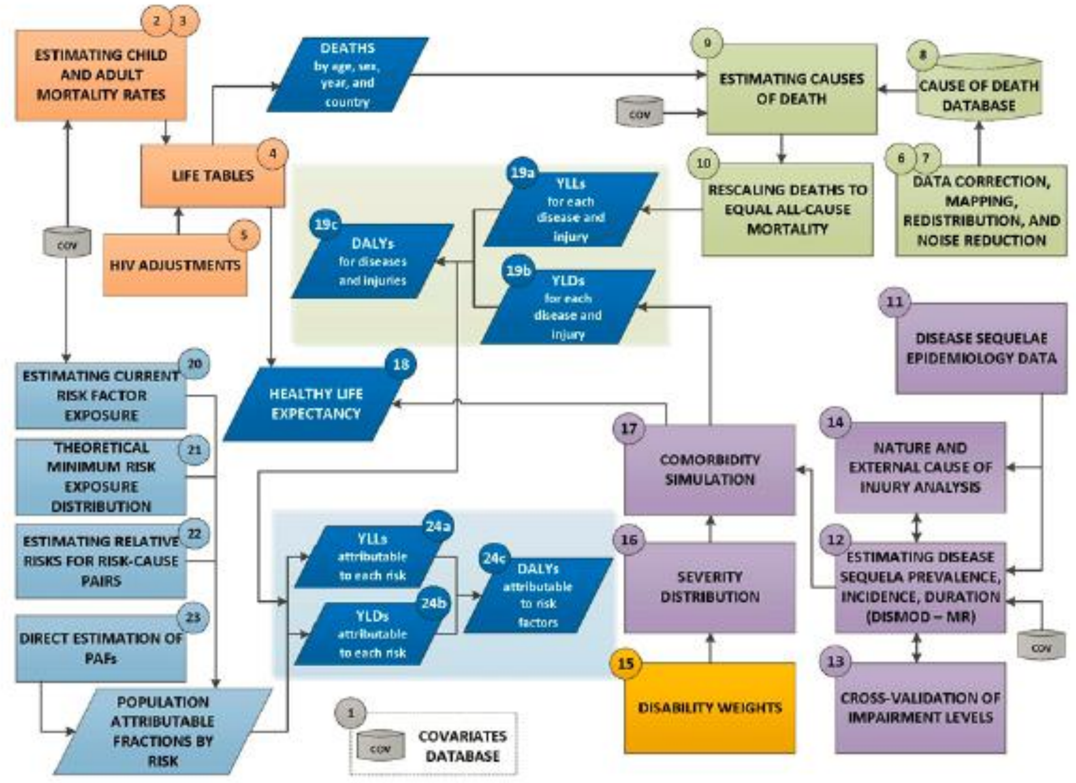

YLLs is years of life lost. YLDs is years lived with disability. DALYs is disability-adjusted life-years. PAFs is population attributable fractions.

Rectangular boxes represent analytical steps, cylinders represent databases, and parallelograms represent intermediate and final results.

The flowchart above illustrates the flow of the key components of the GBD estimation process, including:

1. Incorporation of appropriate covariates (step 1)
2. All-cause mortality estimation (steps 2-5): the data come from sources such as censuses, surveys and vital registrations (VR). The all-cause mortality estimation process (steps 2-4) can be divided into four distinct but interconnected areas: child mortality and adult mortality between ages 15 and 60, estimation of a complete set of age-specific death rates, estimation of HIV mortality and final estimates of age-specific mortality including HIV and fatal discontinuities (also known as mortality shocks; step 5).
3. Causes of death estimation (steps 6-9): cause of death data are derived from VR, verbal autopsy studies, mortality surveillance, and for selected causes, police records, crime reports and data collection systems for deaths due to conflict and natural disasters (step 7). Extensive data corrections and redistributions of ill-defined causes are made to correct for measurement bias between data sources. Cause of death ensemble modelling (CODEm), an ensemble model, is a systematized approach to analysing cause of death data for all but a few causes (step 9). CODEm explores a wide range of modelling approaches and varying predictive covariates to find an ensemble of best-performing models based on statistical tests. To do so, 30% of the data are withheld from each model and the model fit is evaluated by how well it covers the data that were left out. By repeating this process many times over the best performing models are selected. As all results in GBD are estimated over 1,000 times to propagate all sources of uncertainty, we end up with an ensemble of up to 100 or more different types of models and covariates that are selected among the 1,000 runs.
4. Rescaling deaths to equal all-cause mortality (step 10): as all these estimates are made separately for each disease and injury, the sum of these could exceed or fall below the all-cause mortality estimated from the demographic analyses of steps 2 to 5. Therefore, all deaths by age, sex, geography, year and cause are rescaled to match the all-cause death estimates (this process is called CoD Correct).
5. Estimation of disease sequelae prevalence, incidence, and duration (steps 11-12): population surveys, cohort studies, administrative records of hospitalisations and other health service encounters, disease registries, notifications, surveillance systems are the main data sources for non-fatal estimation (step 11). Extensive corrections of data to deal with measurement bias arising from study design or case definitions are applied. DisMod-MR 2.1 is the main analytical tool for non-fatal estimation (step 12). It is a Bayesian meta-regression software program that uses a lognormal model. The meta-regression component allows corrections for known sources of measurement error. Its core function is to make estimates of prevalence and incidence of disease that are consistent with data on mortality risk and remission (defined in GBD as the ‘cure rate’). For a select number of causes that do not fit well in the three state model (alive without disease, prevalent case of disease and death) of DisMod-MR 2.1, alternative modelling strategies are used.
6. Cross-validation of impairment levels (step 13): for a number of impairments in GBD terminology, such as anaemia, heart failure, hearing and vision loss, the total levels of prevalence and incidence were first estimated and then it was ensured that all sequelae of diseases that lead to this impairment add up to the total.
7. Analysis of the nature and external cause of injury is done separately (step 14).
8. Assignment of severity distributions for the main disabling conditions (step 15): in GBD terminology sequelae are the disabling consequences for which we make estimates. All sequelae are defined to be mutually exclusive and collectively exhaustive. Many diseases have sequelae with a gradation by severity such as mild, moderate and severe dementia. Often the epidemiological data on severity distribution is sparse. Therefore, we first model the epidemiology of all cases of disease and then apply a severity distribution from the sparser data.
9. Assignment of disability weights for health states (step 16): each sequela is matched with a health state or combination of health states for which we have a disability which quantifies the relative severity. Disability weights were derived from population and internet surveys of over 60,000 respondents answering pair-wise comparison question of random combinations of health states. Each pair of health states was described with brief lay descriptions highlighting the main symptoms and impairments. Respondents were asked to nominate the ‘healthier’ of each presented pair. Analytical methods exist to formalise the intuition that if the majority of respondents nominate one health state in a pair as the healthier these lie farther apart on a severity scale than pairs assigned similar proportions as the healthier. In order to anchor estimates on a 0-1 scale of severity, a subset of respondents was asked additional population health equivalence questions on a selection of health states. These questions ask for a choice of the greater amount of health produce by two health programs; one that prevented sudden death in 1,000 persons and another that prevented the onset of a GBD health state for the rest of 2,000, 5,000 or 10,000 persons’ lives.

10. Simulation of comorbidity (step 17): the last step of non-fatal estimation is a microsimulation (‘COMO’) to deal with comorbidity. For every age, sex, geography and year, 40,000 hypothetical persons are generated who have none, one or more of the GBD sequelae. In those with multiple sequelae their combined level of disability is estimated multiplicatively. That means we assume the disability from having two health states is less than the sum of the corresponding disability weights. This avoids assigning disability greater than one to any individual which would indicate that person is worse off than being dead.
11. Estimation of healthy life expectancy (step 18): health life expectancy is estimated from the life tables generated in step 4 and the all-cause YLD rates from step 19b.
12. Computation of YLLs, YLDs, and DALYs from diseases and injuries with uncertainty (steps 19a-19c): YLLs (step 19a) are estimated as the product of counts of death by ages, sex, geography, year and cause and a normative life expectancy at the age of the death. The GBD standard life expectancy used as this norm is a compilation of the lowest observed mortality rates by age in all mortality data collections of populations greater than 5 million. The standard life table reflects a life expectancy at birth of 86.59 years. YLDs are the output from COMO (step 19b). DALYs are the simple addition of YLLs and YLDs (step 19c).
13. Risk factor estimation (steps 20-24): GBD 2017 also makes estimates for individual and combined risk factors. This involves estimation of risk factor exposure (step 20); the formulation of a minimum level of exposure to each risk that is associated with the least amount of health loss (step 21); derivation of relative risks of disease outcomes for each pair of a risk factor and a disease or injury for which there is judged to be sufficient evidence of a causal relationship (step 22); and the estimation of population attributable fractions of disease caused by each risk factor. For a few risk-outcome pairs it is hard to define exposure and a corresponding risk while directly observed proportions of disease are available, such as for the proportion of HIV/AIDS due to unsafe sex or injecting drug use (step 23). For combinations of risks how much of the risk is mediated through other risks (step 24) was assessed. For instance, all of the effect of high salt intake is mediated through elevated blood pressure and part of the risk of increased body mass index is through elevated blood pressure, cholesterol or fasting plasma glucose.
14. Computation of YLLs, YLDs, and DALYs attributable to risk factors (steps 25a-25c): YLLs, YLDs and DALYs attributable to each risk factor are generated by multiplying population attributable fractions with disease estimates (steps 25a-c).

B. Child mortality estimation at state level

B.1. Mortality from vital registration

All available data were included from VR systems as inputs in GBD all-cause mortality estimation process. To achieve this, a number of multi-country VR sources were utilised, including the WHO Mortality Database, the Human Mortality Database, United Nations Demographic Yearbooks, and Organisation for Economic Co-operation and Development databases. These multi-country sources were regularly updated in GBD systems when new data were added. Beyond multi-country sources, for all ongoing national VR systems all data sources were catalogued from each system where possible.

Some countries that do not have well-performing VR systems implement sample registration systems that are incomplete by design. These data were used, paying close attention to the proper weighting of sampled data and consistency with other representative sources. The data were extracted systematically from the Sample Registration System (SRS) Statistical Report series published by the Registrar General of India. Census data were systematically extracted from Demographic Yearbook series, Integrated Public Use Microdata Series, and statistical reports from the national statistical bureaus.

B.2. Under-5 populations and live births

For GBD 2017, live births were produced as part of the population and fertility estimation.[1]

B.2.1. Complete birth histories microdata

Complete birth histories (CBHs), the preferred method for data collection on child mortality in the absence of VR, rely on administering surveys to mothers. The questionnaires ask about all living and deceased children, including date of birth, survival status, and date of death. These modules were included in many routine survey series, including the World

Fertility Surveys, Demographic and Health Surveys (DHS), Multiple Indicator Cluster Surveys (MICS), and many national survey programs. When available, this microdata were downloaded and the individual-level survey responses as opposed to tabulated results were used.

B.2.2. Complete birth histories tabulated data

In some instances, tabulated records from reports became available before survey microdata, and those data points were incorporated into the database of 5q0 data as well. However, as microdata became available, point estimates were updated from processed microdata rather than the tabulated report estimates.

B.2.3. Summary birth histories microdata

Summary birth history (SBH) questionnaires are a shorter alternative to complete birth histories. Instead of asking in detail about each child, summary birth histories simply ask mothers how many children they have given birth to and how many of the children have died. The questionnaires are shorter and can be more easily attached to other surveys. Often, censuses and MICS surveys contain summary birth histories. For GBD, all available SBH data with microdata were compiled, which enabled us to apply the updated SBH method to produce a more accurate and timely assessment of U5MR.[2]

B.2.4. Summary birth histories tabulated data

In cases where there was no access to the microdata on SBH modules from surveys and censuses, the reported estimates of U5MR were utilized from survey or census reports and outliered the first two data points based on mothers aged 15-19 and 20-24.

B.2.5. Under-5 age-sex patterns from vital registration/sample registration survey/disease surveillance points

VR systems were the primary source of data for the under-5 age pattern of mortality in high-income countries. Often, these data were classified into several age groups: early neonatal (0-6 days), late neonatal (7-27 days), post-neonatal (28-364 days), and 1 to 4 years. Some country-years of data had other age groupings with less specificity, with the early and late neonatal age groups combined, or all of the under-1 age groups combined. SRS also provided data for the age-sex patterns of under-5 mortality in several countries including India.

B.2.6. Under-5 age-sex patterns from complete birth history

In many countries without VR systems, CBH surveys were used to obtain age-sex patterns of mortality in under-5 age groups. For all CBH microdata sources, direct estimation methods were applied to obtain probabilities of death for each of the under-5 age groups. Within each survey, where each observation is a child recalled by a mother, observations were grouped into 5-year groups in time to provide a data point of probability of death for each of the under-5 age-sex groups. Recall was cut off 15 years before the survey, limiting data points estimated from the survey prior to 15 years. All of these estimates were then put in the database of estimates for the age-sex pattern of under-5 mortality.

B.3. Vital registration prioritisation

The continual evaluation of VR data sources led to the development of a general hierarchy of preferred VR sources. When considering which of the multiple sources are to be used for a given location-year, WHO data was first preferred to be used from GBD cause-specific mortality estimation, followed by unadjusted WHO data, Human Mortality Database (HMD) data, and UN Demographic Yearbook data. There were exceptions to this hierarchy where there was reason to believe that there were quality issues with a certain source. Single-country VR sources were evaluated based on consistency with other data sources and also VR system documentation.

.

B.4. Identification of vital registration under-enumeration for bias correction

The approach to estimating the completeness of VR systems for deaths under-5 was the similar to that of the previous GBD iterations. Most biased VR is corrected in the mixed effects non-linear model outline.

B.5 Biennial 5q0 estimates

B.5.1. Computation of complete birth history for 5q0

Microdata (individual-level survey data) from CBH yielded direct calculation of death numbers and probabilities of death in the under-5 age group. Observations were grouped into two-year intervals such that biennial estimates of 5q0 were obtained from these survey data. In GBD 2017, surveys were unpooled for GBD analysis.[3] Instead of grouping observations from all DHS complete birth history questionnaires from a country into one full set of observations and all MICS observations from multiple survey years into another full set of observations, each survey were analysed separately by location. This allowed for a greater ability to address known data quality issues in specific surveys. To compensate for the decreased sample size and to generate greater stability in the unpooled data points, two-year estimates of under-5 mortality were created, pooling observations over two-year periods instead of single years. The years of recalled CBH data were restricted to two-year intervals with over 10,000 person-months of data as generated by months contributed by each child.

B.5.2. Processing of tabular complete birth history

In some instances, microdata from surveys were not available. If survey reports could be obtained but the microdata were not available to do calculations to obtain 5q0, report data point estimates were used. These estimates were added directly to the under-5 mortality database.

B.5.3. Summary birth history time series method

Summary birth history method from microdata

An SBH method was used to provide more accurate and timely estimates of U5MR from microdata on SBH from surveys and censuses.[2]

Analysis of summary birth history from tabular data

When only tabular data were available for the numbers of children ever born and number of children that have died by mother's age, the Maternal Age Cohort model were applied.[2]

B.6. 5q0 data synthesis, model running, and bias correction

Data synthesis using ST-GPR and bias correction

The child mortality estimation methodology was applied as elsewhere.[3] Based on the under-5 mortality data synthesis model for GBD 2010,[4,5] 2013,[3] 2015,[6] and 2016,[7] data bias adjustment were incorporated into the modelling process. Specifically, a fixed effect for source type were included across all locations to detect systematic differences in the level of child mortality, controlling for covariates for one source type versus another. The groups of sources used to make this adjustment are listed below. In addition, a random effect was included for each country-source. By choosing a reference source (country-by-country or using the mean of a set of sources, a country-by-country basis was adjusted for the problem of compositional bias created by substantial source-specific non-sampling error. Reference sources were not adjusted, even if multiple sources were used as reference. Once the systematic difference in sources were removed, estimating false trends due to partial overlap of sources with different levels of non-sampling variance was avoided. The combination of non-linear mixed effects model, spatiotemporal regression, and GPR to synthesise raw child mortality data were then applied after data bias adjustment to obtain consistent time series estimates of mortality with 95% uncertainty intervals for every country.

The following types of data source were used in child mortality bias correction:

- CBH - Demographic and Health Survey
- CBH – World Fertility Survey
- CBH – Multiple Indicator Cluster Survey
- CBH – Census
- CBH – Other survey series
- SBH – Demographic and Health Survey
- SBH – Multiple Indicator Cluster Survey
- SBH – Other survey series
- SBH – Census
- SBH – World Fertility Survey
- VR/Sample Registration/Surveillance – complete
- VR/Sample Registration/Surveillance – incomplete
- Household Death Recall – Other survey series
- Household Death Recall – Census
- Household Death Recall - incomplete VR/Sample Registration/Surveillance

Each data sources were scrutinized for the data quality and were excluded if they greater than 10% of the records were missing for dates of birth and death (for complete birth histories) or for children ever born and died (for summary birth histories). Additionally, surveys were screened for unrealistic geographic or time trends compared to other surveys in nearby country-years as well as non-standard sampling methodologies. Based on these screening procedures, data sources were included in the first stage prediction.

Mixed effect non-linear model and the bias adjustment for raw U5MR sources

In this stage, a non-linear mixed effects regression was used to estimate data bias and provide first stage predictions.

The nonlinear mixed effects regression model is

$$ {}_5m_{0cys} = \exp[(\beta_1 + \gamma_{1c}) * \log(LDI_{cy}) + (\beta_2 + \gamma_{2c}) * education_{cy} + \gamma_c + \gamma_{cs} + \alpha_t] + \beta_3 * HIV_{cy} + \varepsilon_{cys} $$

cc is country, yy is year, ss is source, is source type
${}_5m_0$ is under-5 mortality rate
LDI is lag-distributed income per capita
education is mean years of education for women of reproductive age (15-49 years)
HIV is death rate due to HIV in age groups 0-4
γ is a random effect
α is a fixed effect on source type across countries
βi is a fixed covariate coefficient
ε is the residual

Each source was categorised into one of the above source type across all countries. The HIV death rates used in this regression are the GBD 2017 HIV estimates. For each country, expert opinion was relied on to choose a source, or combination of sources, which were believed to be the least biased. If a country had a VR system which were deemed to be complete, this was the reference source. If a country did not have a complete VR system, but had DHS estimates from CBHs, these were used as the reference source. If a country had neither of these types of data, or DHS estimates were deemed unreliable, the surveys conducted after 1950 were assigned (in combination) as the reference. Incomplete VR data were not included. Additionally, in many countries alternate surveys were chosen as the reference. For accurate estimation, it was important to have local knowledge on specific data sources' accuracy. All-cause mortality experts drew from their familiarity with data quality to help us to choose the reference category. Each data source had an associated random effect as well as a source

type fixed effect. The values of these random and fixed effects for the reference sources were deemed to be the true deviation from unbiased mortality level. In countries with multiple high-quality sources, the mean of the random and fixed effects from these sources was taken as this true deviation. All other sources were adjusted by including these reference values for the random and fixed effects values instead of those estimated for each individual source.

$$adjusted_5m_{0,cys} = \exp[(\beta_1 + \gamma_{1c}) * \log(LDI_{cy}) + (\beta_2 + \gamma_{2c}) * education_{cy} + \gamma_c + \gamma_{ref,c} + \alpha_{ref,c}] + (\beta_3 + \gamma_{3c}) * HIV_{cy} + \varepsilon_{cys}$$

The exception to this correction was incomplete VR data, which was adjusted upwards using a five year rolling mean of the difference between incomplete VR and a Loess of the already adjusted survey data.

B.6.1. Spatiotemporal smoothing

The spatiotemporal stage smooths the residuals between the predicted time series of 5q0 and the adjusted raw data over time and across countries in the same GBD region. The predicted time series for this was smoother obtained from the equation below; no random effects or survey type fixed effects were included.

$$predicted_5m_{0,cy} = \exp[\beta_1 * \log(LDI_{cy}) + \beta_2 * education_{cy} + \alpha_{intercept}] + \beta_3 * HIV_{cy}$$

The residuals were first found between the predicted time series, above, and the adjusted points. A combination of smoothing functions was then applied to these residuals. For each country-year, the data points in this region were based on their proximity to this country-year in space and time. 99% of the weight to in-country residuals, and 1% of the weight to out-of-country residuals were given. Additionally, a modified tricubic window were used, as specified below, to give more weight to points closer in time, and less weight to points further in time weighted all.

$$w_t = \left(1 - \left(\frac{|r_t - r_{est}|}{1 + argmax_t|r_t - r_{est}|}\right)^{\lambda}\right)^3$$

Where
r_t is the year of interest
r_{est} is the year of the residual being weighted
$argmax_t|r_t - r_{est}|$ is the maximum distance between the year of interest and a residual within the region.
λ is the weighting function that dictates how quickly the weights fall off as the distance in time increases.

A larger λ implies that the assigned weights will diminish slowly with time, while a smaller λ allows the weights to diminish more rapidly with time. λ values were chosen using the parameter selection process described below. One estimate of the smoothed residuals were then created using a linear fit to this weighted data, similar to a Loess fit. Additionally, a second estimate of the smoothed residuals were created by calculating the weighted average of this data. These two estimates were then combined for a final estimate of the smoothed residuals. In data-dense countries, more weight was given to the local linear fit; in data sparse countries, more weight was given to the weighted average. The equation for this is as follows.

$$final\ smoothed\ residual = k * linear\ estimate + (1 - k) * weighted\ average$$

Where

$$k = \frac{number\ of\ in\ country\ data\ points}{number\ of\ in\ country\ data\ points\ +\ number\ of\ country\ years\ with\ no\ data}$$

Finally, the smoothed residuals were added back to the predictions from above; this smoothed approximation to the adjusted data was used as the prior for GPR, described below.

B.6.2. Gaussian process regression

The output of the space-time smoothing step was used as a prior for GPR, which produced a final time series of point estimates, as well as confidence bounds. Parameters for GPR were chosen through cross validation as described below.

The model for GPR was

$$\mu_t = f(t) + S_t$$

$$f(t) \sim GP(M, C)$$

Where

μ_t is the true $\log_{10}(5q0)$ at time t

$f(t)$ is the baseline mortality risk

S_t is excess mortality due to fatal discontinuities estimated independently of $f(t)$

M is the mean for the Gaussian process

C is the covariance for the Gaussian process

A prior distribution for (t) from the spatiotemporal regression were specified, and a likelihood function that describes the data generation process; the specified prior distributions and likelihood function are described below. Markov Chain Monte Carlo (MCMC)[8] were then used to approximate the posterior distribution of $f(t)$, which also incorporated information from the observed empirical estimates of adult mortality. An MCMC chain of length 5,000 was produced; the first 3,000 samples were discarded and the remaining 2000 were thinned by a factor of 2 for a total of 1,000 simulations retained. The reported best estimates and uncertainty intervals were generated from the mean and the 2.5th and 97.5th percentiles of the 1,000 samples, respectively.

B.6.3. Priors

The prior distribution of $f(t)$ can be described in terms of the mean prior—the prior for MM—and the covariance prior—the prior for CC. The second stage predictions were utilised as the mean prior and used a Matern covariance function to describe the covariance prior. The parameters of the Matern covariance function were selected through cross-validation and were location-specific. This covariance function incorporated three parameters: the amplitude, which controlled the amount by which realisations of the Gaussian process distribution can deviate from the mean function, the scale, which controlled the distance over which the function was correlated, and the degree of differentiability, which influenced the smoothness of the samples from the Gaussian process. These parameters were selected in the parameter selection process described below.

B.6.4. Likelihood

The likelihood describes the probability of observing the data given a particular set of parameters. As shown in the equation below, a normal model was used for describing the probability of observing a particular value of log(5q0)

$$log_{10}({}_5q_{0_t}) \sim Normal(f(t), V_t)$$

Where *f(t)* is the mean and V_t is the data variance

Data variance was calculated for each empirical observation of 5q0 and incorporated both sampling and non-sampling variation. The method for calculating the data variance depended on the type of data:

1. For estimates derived from complete VR data, it was assumed that there was no non-sampling variance and included only sampling variance as computed from a binomial model. N was set equal to the national population aged 0 to 5 years and p equal to the mortality rate, ${}_5m_0$. The variance of ${}_5m_0$ was calculated from $(1-p)/N$ and then transformed this to the variance of $\log_{10}$(5q0) using the delta method.[9]
2. For estimates derived from incomplete VR data, it was required to include not only sampling variance but also the non-sampling variance that arises from uncertainty in the completeness estimate. For these data, the total data variance was given by the sum of the sampling variance (calculated as for complete VR data) and the variance of the completeness estimate.
3. For estimates derived from CBHs, 1,000 simulations of 5q0 were generated, converted these estimates into $\log_{10}$ space, and calculated the sampling variance from these 1,000 simulations.
4. For estimates derived from SBHs the standard error from the mean residuals were used.
5. For estimates not covered under the above four calculations, the missing data variance was determined as the maximum standard error from non-VR points in the country. If the data variance was still missing it was calculated as the maximum standard error from non-VR data in the GBD region.
6. For each source type the within-source-type variance of the source-specific random effect were calculated. This additional non-sampling variance was then converted to log10 space and added to the variance as calculated above for all data points not classified as complete VR.

B.6.5. Hyperparameter selection for under-5 mortality rate ST-GPR

Hyperparameters were selected based on a newly-created data density score for a given location. The data density score was calculated for each location based on the number of deaths from VR sources as well as the number of unique CBH and SBH available. The data density score was computed using the following steps:

1. Complete VR score calculation: this component of data density was computed based on the number of deaths from an unbiased VR sources in the location. Using the death counts, the number of deaths were capped at 500 for each year and then divided that number by 500. The result was a score for each year between 0 and 1 where 1 represents a complete VR system with at least 500 registered deaths. To get the final complete VR score for a location, the score was added up for each year across the full time series. The result was a complete VR score between 0 and 68 (the range of GBD full estimation time series was 1950-2017).
2. Incomplete VR score calculation: this component of data density is computed in the same manner as the complete VR score using biased VR instead of unbiased VR.
3. Total CBH sources is simply a count of the unique complete birth histories for a location.
4. Total SBH sources is simply a count of the unique summary birth histories for a location.

Once the intermediate calculations were completed, the following formula was used to compute the final data density:

$$data\_density = complete\_vr\_deaths' + (0.5 \times incomplete\_vr\_deaths) + (2 \times cbh\_sources) + (0.25 * sbh\_sources)$$

Once the data density for a location was calculated, hyperpameters were assigned:

Data Density	Zeta	Lambda	Scale
0 to 9	0.7	0.7	15
10 to 19	0.7	0.5	15
20 to 29	0.8	0.4	15
30 to 49	0.9	0.3	10
50 plus	0.99	0.2	5

B.7. Identification and removal of outliers

There were several important quality-control steps in reviewing child mortality data and estimates. First, data points from years in which fatal discontinuities occurred were outliered, unless they were VR data points with sufficient information that the fatal discontinuities could simply be subtracted out of the VR data. The intent was to capture the underlying mortality risk rather than large stochastic variations. These fatal discontinuities were then added on in a later step. Secondly, data sources with quality concerns were outliered. GBD extensive collaborator network allowed for review of sources, and collaborators raised concerns over known issues with data sources about which they had expert knowledge.

B.8. Raking of subnational estimates to national level

The estimation process for 5q0 does not enforce consistency between subnational estimates and national estimates. To ensure consistency throughout the GBD hierarchy, the subnational estimates were rescaled to the national level via population-weighting to calculate an implied national estimate from the subnational estimates. This created a scalar of the national-level estimate from GPR to the aggregated subnational estimates, which was then multiplied by all of the subnational estimates to obtain the scaled estimates. In most cases, national-level estimates were considered to be more reliable, so this strategy of subnational scaling were chosen. In locations with high-quality VR data, this scaling has a minimal effect, but the effect can be greater in locations with more subnational units and variable equality data.

B.9. Review of estimates for quality

Estimates of 5q0 from the spatio-temporal Gaussion process regression (ST-GPR) were reviewed in comparison to UNICEF estimates from their 2015 revision and GBD 2015 results.[10] Any differences were traced to either changes in available data or changes induced by changes in hyperparameters or input covariates. Revisions were made through this review process and through expert consultation with the GBD mortality collaborator network.

B.10. U5MR with HIV

The 5q0 ST-GPR process generated U5MR for all GBD 2017 locations that was inclusive of the impact of all causes of death excluding fatal discontinuities, which were added in a separate step.

B.11. Under-5 age and sex pattern model estimation

The process used to break down under-5 mortality into age-and sex-specific groups has been previously described.[7] The current process was largely similar but was modified to improve the accuracy of predictions for countries affected by HIV/AIDS. Neonatal mortality tends to be overestimated if the all-cause U5MR is used as the only predictor.[11] A multi-stage modelling process were used to generate sex-specific estimates of early neonatal (days 0 to 6), late neonatal (days 7 to 27), post-neonatal (the remainder of the first year), under-1, and childhood (ages 1 to 4) mortality. First, the ratio of male to female 5q0 was estimated, then age- and sex-specific mortality estimates were generated using this ratio. To fit models to obtain estimates data from VR, sample VR, and CBHs were converted to mortality risks for specific age groups. Sources had differing levels of age specificity and at minimum included infant (composed of early neonatal, late neonatal, and

postneonatal) and child mortality, but could include all 4 smaller age groups. The sex-specific model was fit to the data, followed by the age-specific model.

The sex model first predicted the ratio of male 5q0 to female 5q0 in rescaled logit space for each country i in region j in year t. The data were ordered by observed 5q0, and categorized into 20 evenly sized bins. The ratio data were rescaled between 0 and 1 to between 0.8 and 1.5. Then the model was fit to the data as described in the equation below.

$$logit\left(\frac{Male\ {}_5q_0}{Female\ {}_5q_0}\right)_{jit} = \beta + \gamma_{{}_5q_0\ bin} + \gamma_j + \gamma_i + \varepsilon_{jit}$$

The ratio was predicted by nested location and region random effects γi and γj, a random effect on the 5q0 bin, and an intercept term, β. A Loess regression was then used to smooth the estimated $\gamma 5q0$ bin on 5q0, creating a continuous $\gamma' 5q0$ bin. Then, the equation below was used to predict the ratio of male to female 5q0:

$$logit\left(\frac{Male\ {}_5q_0}{Female\ {}_5q_0}\right)_{jit} = \hat{\beta} + \gamma'_{{}_5q_0\ bin}\left({}_5q_{0_{jit}}\right) + \hat{\gamma}_j + \hat{\gamma}_i$$

The predicted ratios were unscaled, inverse logited, and used as the first stage prediction for ST-GPR as used for the estimation of U5MR. ST-GPR hyperparameters were chosen using the same method outlined for U5MR. The male and female 5q0 values were found using the system of equations that includes the prediction from the ST-GPR described above and the equation below, where γ_{birth} is the sex-ratio at birth.

$${}_5q_0 = \left(\frac{1}{1 + r_{birth}}\right) * \left(female_{{}_5q_0}\right) + \left(\frac{r_{birth}}{1 + r_{birth}}\right) * \left(male_{{}_5q_0}\right)$$

Age-specific models were then fit for each age group on sex-specific data. A separate model was fit for each age group, yielding five models for each sex: early neonatal, late neonatal, post-neonatal, infant, and child. The log of the probability that an under-5 death occurs in a given age group conditioned on surviving to that age group was modeled instead of the mortality risk, simplifying the scaling process and restricting risks to be between 0 and 1. Because evidence suggests HIV has differential effects on different under-5 age groups,[12,13] the crude death rates from HIV/AIDS in the under-5 age group were included in the model for age groups after neonatal, given our assumption that all HIV deaths that occur in the first year of a child's life occur in the post-neonatal stage (after 28 days). The literature on HIV in these age groups is still unclear, but seems to indicate higher mortality in the post-neonatal stage. There is no clear evidence to guide alternative methods of age-splitting under-1 deaths due to HIV.[13,14] The crude death rate due to HIV were used from the GBD 2017 model. The inclusion of this covariate improves both the fit and prediction of the model in countries with high HIV prevalence. The maternal education covariate that is also used in the 5q0 first-stage model and the completeness of the source-specific 5q0 estimate were included for the data-point used in the regression for that 1 to 4 age group only. This completeness measure was calculated by taking the source-specific 5q0 point estimate and dividing by the final 5q0 estimate from GPR. The functional forms of the model are below, where age y included late-neonatal, post-neonatal, and under-1 children.

$$log(Pr(death\ at\ age\ enn|u5\ death)_{jit}) = \beta_1 + \gamma_{{}_5q_0\ bin} + \gamma_j + \gamma_i + \varepsilon_{jit}$$

$$log(Pr(death\ at\ age\ y|u5\ death)_{jit}) = \beta_1 + \beta_2 * HIV_{it} + \gamma_{{}_5q_0\ bin} + \gamma_j + \gamma_i + \varepsilon_{jit}$$

$$log(Pr(death\ in\ age\ 1-4\ |u5\ death)_{jit}) = \beta_1 + \beta_2 * HIV_{it} + \beta_3 * Mat.Ed._{it} + \beta_4 * Completeness_{sit} + \gamma_{{}_5q_0\ bin} + \gamma_j + \gamma_i + \varepsilon_{jit}$$

Similar to the sex model, the sex-specific age prediction used 5q0 bins and smoothed the random effect on the bin using 5q0. The prediction equation for the age group 1 to 4 is shown below.

$$log(Pr(death\ in\ age\ 1-4|u5\ death)_{jit}) = \hat{\beta}_1 + \hat{\beta}_2 * HIV_{it} + \hat{\beta}_3 * Mat.Ed._{it} + \hat{\beta}_4 * 1 + \hat{\gamma}'_{_5q_0\ bin}({}_5q_{0_{jit}}) + \hat{\gamma}_j + \hat{\gamma}_i$$

Where

y is age

j is region

t is time

$\hat{\gamma}_i$ is nested random effects on country

$\hat{\gamma}_j$ is nested random effects on region

$\hat{\beta}_1$ is an intercept term

$\hat{\gamma}'_{_5q_0\ bin}({}_5q_{0_{jit}})$ is a smoothed random effect on 5q0 bin

$\hat{\beta}_2$ is a coefficient on the under-5 crude death rate from HIV

$\hat{\beta}_3$ is a coefficient on maternal education

$\hat{\beta}_4$ is a coefficient on completeness

Note that for prediction, the completeness coefficient was multiplied by 1 instead of a source-specific completeness, as we sought to predict based on a hypothetically complete source. Similar to the sex ratio model discussed above, these predictions were used as the first stage prediction for ST-GPR that produced sex and age specific probabilities of death. Once each of these predictions was made by age group, they were rescaled such that the probabilities of death in the early neonatal, late neonatal, post-neonatal, and 1 to 4-year age groups aggregated to the 5q0 estimates from the under-5 model.

B.12. Identification and removal of outliers

There were several criteria for removing outliers for the under-5 age-sex pattern model. For the sex model, non-VR (survey) data points from high-quality VR locations (as determined by the GBD VR quality rating system) were outliered to ensure the model followed the highest quality data. Additionally, unoutliered sex ratio data were adjusted to be between 0.8 and 1.5. For the age model, the following outliering criteria were used: VR data that were considered incomplete were marked as outliers. To be considered incomplete, the 9-year rolling average of the VR data 5q0 value was compared to the 9-year rolling average of the 5q0 estimates. Then, for a given data-year, the value of 5q0 in the raw

$$q_{enn} = \Pr(death\ in\ enn \mid u5\ death) * 5q0$$

$$q_{lnn} = \Pr(death\ in\ lnn \mid u5\ death) * 5q0/(1 - q_{enn})$$

The older age groups were also calculated in this manner, yielding probabilities of death in each of the under-5 age-sex groups.

B.13. Under-5 death number estimation

Assigning under-5 deaths to GBD age-sex groups

To estimate the number of under-5 deaths, an estimation process that aged birth cohorts was ran through estimated probabilities of death. This process separated yearly birth numbers for each location into week-sized cohorts and aged each of these cohorts through mortality estimates in week-long steps to estimate the number of person-years and deaths in each of the early neonatal, late neonatal, post neonatal, and 1-4 years age groups.

Completeness data synthesis

Once a full series of under-5 completeness estimates were obtained for each country source the Model were fit described in the equation:

$$log_{10}(c_{i,s,t}^{adult}) = \alpha + \beta_1 \times log_{10}(c_{i,s,t}^{child}) + \gamma_1^{SR} + \gamma_2^{SR} \times log_{10}(c_{i,s,t}^{child}) + \gamma_1^{R} + \gamma_2^{R} * log_{10}(c_{i,s,t}^{child}) + \eta_{i,s} + \xi_{i,s,t}$$

Where

i is country

s is source

t is time

R is region

SR is super-region

$c_{i,s,t}^{adult}$ is completeness of adult deaths registration

$c_{i,s,t}^{child}$ is completeness of child deaths registration

γ terms are random effects

$\eta_{i,s}$ is a random effect at the country and source level

$\xi_{i,s,t}$ is an error term

This model related adult completeness to under-5 completeness and included super-region and random effects to allow for differences in both the average level of adult completeness and the relationship between child and adult completeness at these levels. The country-source random effect captured the fundamental difference in level of completeness between different data sources.

A $\log_{10}$ transformation was employed to make over- and under-completeness symmetric (e.g., 50% complete and 200% complete were symmetric around 0 when $\log_{10}$ transformed) and to simplify calculation of the variance of completeness estimates in log10 space, which was needed for the adult mortality estimation process. For each country-source $log_{10}(c_{i,s,t}^{adult})$ was predicted from coefficients estimated in the model above and child completeness. Not every country can be used to fit this model, as death distribution methods cannot be applied in some cases due to lack of appropriate census data. However, because the coefficients used for prediction were at the region and super-region level, predictions from this model could be generated for all countries where estimates of child completeness were available. The same relationship between adult and child completeness was not believed to exist for registration-based sources as for recall based sources, so the above model was applied only to registration-based sources (primarily VR data but also sample registration systems). For sources that only included household death recall, an arbitrary value of 1 was set for the first stage values instead of making predictions from completeness from child age groups for the aforementioned reason. However, it should be noted that this set value by no means reflected the true completeness of adult age groups in the household death recall sources and it changed once the spatial-temporal regression was applied. In the second stage, the residuals from the first stage were calculated and applied spatial-temporal smoothing to these residuals. The predicted residuals were then added back onto the first-stage predictions, generating the second-stage predictions. Spatial-temporal smoothing was carried out in the same way as in the adult mortality estimation process with three modifications:

1. The λ and ζ parameters were set to 2.0 and 0.95.
2. Only the fixed effect local regression variant was used.
3. The residuals were not held constant out of sample.

The registration-based sources and the non-registration-based sources were handled separately in this step. To do this, the variance was approximated based on the median absolute deviation (MAD) compared to the second-stage estimates. The variances were calculated at the regional level, and did so separately for registration-based sources and other sources. Then, for each country-source-year, 10,000 simulations were generated from a normal distribution with mean equal to the second-stage prediction

for that year and variance. For non-registration-based sources, that both under-and over-reporting were believed to be possible (over-reporting might have occurred due to telescoping of events outside of the recall period into the recall period), and so for these sources the 10,000 simulations were exponentiated and the mean was calculated, which served as the final prediction for completeness. For registration-based sources only underreporting was possible, so for these sources any simulations above 1 were first truncated to 1. The 10,000 simulations were then exponentiated to find the truncated mean, which served as the final prediction for completeness. In both cases, before exponentiating the simulations, the variance of the simulations was calculated and used as the variance of the completeness estimates in the adult mortality estimation process. For countries in which males and females were believed had differential completeness, the above process was carried out separately by sex. For a small number of data points completeness could not be estimated using the procedure described above due to a lack of appropriate census data; the original growth balance method was the only viable option.[12] In previous papers, a selected number of data points derived from household recall of deaths were included to which the Brass growth balance method had been applied. GBD simulation studies suggested that this method was extremely imprecise, so these points were excluded from the analysis.

References:

1. GBD 2017 Population and Fertility Collaborators. Population and fertility by age and sex for 195 countries and territories 1950-2017: a systematic analysis for the GBD 2017. *Lancet* 2018; 392: 1995–2051.
2. Rajaratnam JK, Tran LN, Lopez AD, Murray CJL. Measuring under-five mortality: validation of new low-cost methods. *PLOS Med* 2010; 7: e1000253.
3. Wang H, Liddell CA, Coates MM, *et al.* Global, regional, and national levels of neonatal, infant, and under-5 mortality during 1990–2013: a systematic analysis for the Global Burden of Disease Study 2013. *Lancet* 2014; 384: 957–79.
4. Murray CJ, Ezzati M, Flaxman AD, *et al.* GBD 2010: design, definitions, and metrics. *Lancet* 2012; 380: 2063–6.
5. Wang H, Dwyer-Lindgren L, Lofgren KT, *et al.* Age-specific and sex-specific mortality in 187 countries, 1970–2010: a systematic analysis for the Global Burden of Disease Study 2010. *Lancet* 2012; 380: 2071–94.
6. Wang H, Bhutta ZA, Coates MM, *et al.* Global, regional, national, and selected subnational levels of stillbirths, neonatal, infant, and under-5 mortality, 1980–2015: a systematic analysis for the Global Burden of Disease Study 2015. *Lancet* 2016; 388: 1725–74.
7. Wang H, Abajobir AA, Abate KH, *et al.* Global, regional, and national under-5 mortality, adult mortality, age-specific mortality, and life expectancy, 1970–2016: a systematic analysis for the Global Burden of Disease Study 2016. *Lancet* 2017; 390: 1084–150.
8. Gelman A, editor. Bayesian data analysis, 2nd ed. Boca Raton, Fla: Chapman & Hall/CRC, 2004.
9. Gompertz B. On one uniform law of mortality from birth to extreme old age, and on the law of sickness. *J Inst Actuar Assur Mag* 1871; 16: 329–44.
10. The UN Inter-agency Group for child mortality estimation. Child mortality estimates. http://www.childmortality.org/ (accessed May 20, 2017).
11. Bradshaw D, Dorrington R. Child mortality in South Africa - we have lost touch. *S Afr Med J* 2007; 97: 582.66
12. Kerber KJ, Lawn JE, Johnson LF, *et al.* South African child deaths 1990-2011: have HIV services reversed the trend enough to meet Millennium Development Goal 4? *AIDS Lond Engl* 2013; 27: 2637–48.
13. Kim H-Y, Kasonde P, Mwiya M, *et al.* Pregnancy loss and role of infant HIV status on perinatal mortality among HIV-infected women. *BMC Pediatr* 2012; 12: 138.
14. Brocklehurst P, French R. The association between maternal HIV infection and perinatal outcome: a systematic review of the literature and meta-analysis. *Br J Obstet Gynaecol* 1998; 105: 836–48.

C. Geospatial mapping methods for child mortality

The analytical process generally followed that of previous work to map under-5 mortality probabilities,[1] diarrhoea prevalence,[2] child growth failure,[3] and educational attainment[4] with several key exceptions and methodological advancements. The aim of this analysis was to produce joint estimates, with uncertainty, of the probability of death for children aged 0-28 days (neonates), children under-1 (infants), and children under-5, as well as estimates of the numbers of deaths for these age groups, at the subnational level in India for each year from 2000 to 2017. Estimates were generated using a statistical model that was continuous in space, and prediction was done at a grid-cell resolution of approximately 5x5 km at the equator (0.042 decimal degrees), and reported at the first and second administrative level, as well at the country level. The term mortality *probability* was used to describe the number of deaths per 1000 live births. This is the quantity that was modeled and is discussed in the paper. In standard demographic notation, mortality probability for under-5 children is referred to as 5q0. Often, it is colloquially acceptable to use the term mortality rate for this measure. These have technically different definitions; mortality rate refers to deaths per person-years lived. Since both mortality probabilities and rates are being used in this analytical processing, for the purpose of clarity these terms would not be used interchangeably in describing the methodology.

The individual records were extracted from data sources. Records were gathered either in the form of SBHs or CBHs. SBH data are at the woman level, while CBH data are at the child level. Data preparation differed for these two types: sample sizes and number of death events over time and age could be tabulated directly for CBH data, while SBH data were prepared in accordance with indirect methods developed and validated previously.[5] Seven household surveys of summary birth histories and complete birth histories in India were included for the pixel level mortality estimation. Each data sources were scrutinized for the data quality and were screened for unrealistic geographic or time trends compared to other surveys in nearby country-years as well as non-standard sampling methodologies. Based on these screening procedures, data sources were included. Surveys were screened for high incompleteness because a survey with a high non-response rate may indicate that the respondents were not representative of the overall survey population. All data observations were geo-referenced to either global positioning system (GPS) locations (points) or areal units. Areal data were converted to pseudo-points and weighted based on spatial population distributions.

The values from each of 10 geospatial covariates were extracted at each data point. Geospatial covariates are spatial data which are represented at the 5x5 km grid-cell resolution. Covariates typically have global spatial coverage and values that vary each year.

In order to synthesize information across various sources, and to make consistent estimates across space and time, discrete hazards geostatistical models were fit to binomial data. The model explicitly accounted for variation across age bin, time, and space through inclusion of both fixed and random effects. Indicator variables for each age bin were included to form a discrete baseline mortality hazard function. Baseline hazard functions are allowed to vary in space and time in response to changing covariate values, as well a linear effect on a secular time trend. A Gaussian random effect was included across countries to account for larger-scale variations due to political or institutional effects. The Gaussian random effects were also included for each data source to account for source-specific biases. Finally, a Gaussian process random effect were included with a covariance matrix structured to account for correlation across age, time, and physical space. As such, estimates at a specific age, time, or place benefit from drawing predictive strength from data points which are nearby in any of these dimensions. The priors were assigned to all model parameters and performed maximum a posteriori (MAP) inference using Template Model Builder[6] (TMB) software in R version 3.4.

From the fitted model parameters, predictive posterior mortality estimates were produced for each age group at each 5x5 km grid cell for each year 2000 through 2017. These estimates with grid-cell-level population data were also supplemented in order to estimate the number of deaths occurring in each age group at each location in time. It was ensured that at the national level, aggregated estimates for each age group and year are calibrated such that they equal estimates in the GBD study.

The following sections provide detailed description on the analytical process.

C.1. Modelling strategy

Standard demographic notation used *n*: length of age bin; *x:* starting age of age bin; *d*: number of deaths; *q*; probability of death; *a*: average time lived in age bin by those who died in the age bin.

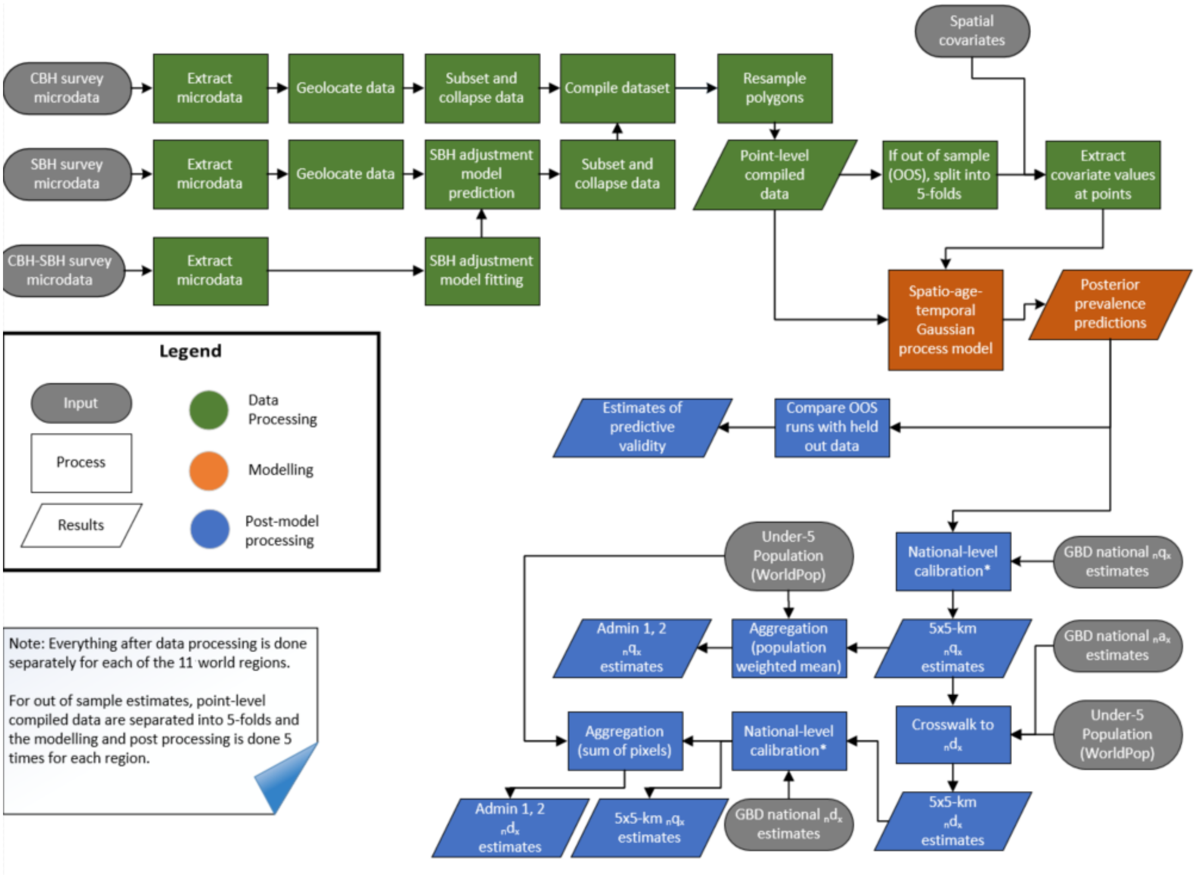

Data sources

The data sources used for the geospatial modelling of child mortality are described in the table below. In India, there were four identified national surveys consisting of SBH and three national surveys consisting of CBH data including National Family Health Survey, District Level Health Survey, and Annual Health Survey as listed below which were included in the analytical process.

Year	Source	Children Born	Polygons	Points	Type
1998-1999	International Institute for Population Sciences (IIPS), Ministry of Health and Family Welfare, Government of India, ICF. India National Family Health Survey Data (NFHS-2) 1998-1999 Mumbai, India: IIPS.	268,879	26	0	CBH
2002-2004	International Institute for Population Sciences (IIPS), Ministry of Health and Family Welfare, Government of India. India District Level Household Survey Data (DLHS-2) 2002-2004. Mumbai, India: IIPS.	1,391,228	585	0	SBH
2005-2006	International Institute for Population Sciences (IIPS), Ministry of Health and Family Welfare, Government of India, ICF. India National Family Health Survey Data (NFHS-3) 2005-2006: Mumbai, India: IIPS.	256,782	29	0	CBH
2007-2008	International Institute for Population Sciences (IIPS), Ministry of Health and Family Welfare, Government of India. India District Level Household and Facility Survey Data (DLHS-3) 2007-2008. Mumbai, India: IIPS.	1,818,042	590	0	SBH
2012-2013	International Institute for Population Sciences (IIPS), Ministry of Health and Family Welfare, Government of India. India District Level Household and Facility Survey Data (DLHS-4) 2012-2013: State Reports. Mumbai, India: IIPS.	259,034	275	0	SBH
2010-2013	Office of the Registrar General & Census Commissioner, Ministry of Home Affairs, Government of India. India Annual Health Survey Data 2010-2013. New Delhi, India: Office of the Registrar General & Census Commissioner.	31,956,497	277	0	SBH

2015-2016	International Institute for Population Sciences (IIPS), Ministry of Health and Family Welfare, Government of India, ICF. India National Family Health Survey Data (NFHS-4) 2015-2016: Mumbai, India: IIPS.	1,315,617	82	28,387	CBH

CBH=Complete birth history. SBH= Summary birth history.

The surveys that have both complete and summary birth histories were only used since complete birth histories are more detailed and to avoid using the same data twice. For each data source a data quality report was produced. Data sources were excluded based on screening for missingness and unexpected trends in several variables. Sources were excluded due to missingness greater than 10% in date of birth or death and children ever born or died, unrealistic geographic trends compared to other surveys in nearby country-years, inability to match the microdata to geographic locations, or non-standard methodology.

Each individual child record was associated with a cluster, a group of neighbouring households or a 'village' that acts as a primary sampling unit. Some surveys included geographical coordinates or precise place names for each cluster within that survey. In the absence of geographical coordinates for each cluster, data were assigned to the smallest available administrative areal unit in the survey, either state or district, while correcting for the survey sample design. Boundary information for these administrative units were obtained as shapefiles from ML Infomap (https://www.mlinfomap.com/).

Survey reports that were manually extracted at an aggregated level, most often at the first administrative (Admin 1) or second administrative (Admin 2) level, were appended to the collapsed data, and all data attributed to a polygon were resampled to points. After examining diagnostic plots, a small number of surveys were dropped for exhibiting implausible trends. The final cleaned and vetted data were used as input data for the modelling.

Data preparation

CBH capture detailed vital event histories of children, as reported retrospectively by their mothers. CBH surveys include information about the month and year of birth, as well as the age of death if applicable for each child born to responding mothers. Data on life and mortality experiences from CBH sources can be tabulated directly into discrete period and age bins, thus allowing for age- and period-specific mortality estimation, known as the synthetic cohort method.[8–10] Data tabulated for a specific period and age are referred as period-age-binned.

For this study, 18 annual period bins from 2000 to 2017, and seven age bins were used. Age bins were defined as follows: Neonatal (live birth–1 month), Post-neonatal 1 (1 month–6 months), Post-neonatal 2 (6 months–1 year), 1 year olds (1 year–2 years), 2 year olds (2 years–3 years), 3 year olds (3 years–4 years), 4 year olds (4 years–5 years). Thus, one child can supply information toward up to seven age bins. Each child entering a period-age bin counts toward the sample size of that age bin (unless the child entered the age bin more recently than the length of the age bin, in which case they were censored). If a child died within a period-age bin, that death is recorded as occurring in that bin. The ratio of deaths within to number entering can thus be considered an estimate of the probability of death in that age bin, conditional on entering the age bin. This is the same as the lifetable quantity q_a for age bin a.[11]

For survey clusters, deaths and number were tabulated directly. For areal units, survey weights were used, if provided, to estimate a mortality probability for each component period-age bin, which was then multiplied by the sample size of the period-bin within the areal unit to get an estimate of numbers of deaths.

In an SBH, a respondent (typically the mother), is asked about how many live births she has had (children ever born, CEB), and how many of her children have died (CD). There is no additional information given about the timing of births or deaths, and as such, an additional inferential step is needed in order to estimate mortality probabilities and assign them to specific time period. This step is broadly referred to as indirect estimation.[11]

Summary birth histories were prepared in accordance with the methods detailed in the paper by Burstein and colleagues.[5] The methods are briefly described here.

A discrete time generalized additive hazard model was trained using individual-level data from DHSs. The model was fit on CBH data but using covariates also available from SBH data (mother's age, CEB, CD/CEB, and national-level covariates). SBH data were then applied to the fitted model to make predictions of discrete hazard curves for all hypothetical children potentially ever born to mothers reporting in SBH. Discrete hazard curves were set up to have breaks at the same seven age bins used for tabulation of CBH data. Each of these hypothetical children is given a weight based on their probability of birth (given their mother's age, total fertility, and region of residence). Hazard curves were turned into age-period-specific estimates of mortality by taking weighted means across all hypothetical children living in a survey cluster or administrative area. In addition, weights were summed over these area-age-bins to approximate sample sizes.

To account for the fact that SBH data arose from modelled indirect estimates, a three-step simulation procedure were used to calculate an effective sample size for each SBH data point based on uncertainty from predictions and out-of-sample uncertainty as calculated by Burstein and colleagues.[5]

The age-specific indirect estimation method described previously were applied to all SBH datasets used in the paper, to produce an estimate of mortality probability ($\hat{p}$) and sample size ($\widehat{N}$) for each area-age-period bin from each survey (notated as $s, a, t,$ and k, respectively). The smallest available geography from each survey, typically a cluster or a second administrative or first administrative unit were referred to as areas. The administrative unit data were converted to points spread across the corresponding administrative division according to a resampling algorithm that accounted for population distribution. The combined dataset consisting of geo-referenced points and converted points provided the number of deaths and sample size for a particular location by age and time period. Boundary information for administrative units for the year 2018 was obtained as shapefiles from the ML Infomap. Sample sizes represented estimates of the number of children expected to enter each area-age-period bin, estimates of $\widehat{N}$ were deterministic using above described method, but $\hat{p}$ arose from a statistical model, and thus uncertainty in fitted model parameters was propagated into estimates of $\hat{p}$. For each $\hat{p}_{k,a,s,t}$ 1000 predictive draws were extracted from the model by simulating from a multivariate Gaussian distribution using the mean vector and variance-covariance matrix of all estimated model parameters. Furthermore, modelled estimates could deviate from the truth for various reasons not captured in model uncertainty. This added variance can be estimated by comparing model estimates to out of sample empirical validation data. This procedure was done as part of the subnational validation where they performed leave-one (survey)-out validation for each of the 243 DHSs used to train the complete model. To account for this additional error within each age bin, the error with the relative residual error terms (cv) for each of the seven age bins were first estimated. The value cv_a for each of the seven age-bins was calculated across all surveys as $SD\big(logit(\hat{p}_{k,a,t}) - logit(p^{oos}_{k,a,t})\big)/mean(logit(\hat{p}_{k,a,t}))$, where $\hat{p}_{k,a,t}$ is the model estimate for age bin a from survey k and $p^{oos}_{k,at}$ is the empirical estimate for the same survey held out of sample (i.e., the validation data).

In the first step, this out of sample residual error into each estimate of $\hat{p}_{k,a,s,t}$ via simulation were incorporated. This was done for each draw of each area-age-bin estimate, in logit space to constrain estimates from 0 to 1, by simulating: $logit(\hat{p}^{inflated}_{k,a,s,t}) \sim Normal(logit(\hat{p}_{k,a,s,t}), logit(\hat{p}_{k,a,s,t}) * cv_a)$.

In the second step numbers of deaths for each area-age-period bin for each of the 1000 draws were simulated: $\hat{Y}^{inflated}_{k,a,s,t} \sim Binomial(\hat{p}^{inflated}_{k,a,s,t}, \widehat{N}^{inflated}_{k,a,s,t})$.

In the third step, the effective sample size were calculated for each area-age-period bin by assuming that $\hat{Y}^{inflated}_{k,a,s,t}$ arose from a beta-binomial distribution. The beta-binomial distribution was chosen because it assumes a fixed sample size ($\widehat{N}$), but a variable probability, accounting for model and residual uncertainty there was incorporation into $\hat{p}^{inflated}_{k,a,s,t}$ (and thus $\hat{Y}^{inflated}_{k,a,s,t}$) via simulation in the first two steps. In the geostatistical model, a binomial data likelihood was used, so the interest was in finding an

effective sample size for a binomial distribution with the same variance as a beta-binomial distribution for each area-age-period observation. Across the draws for each $\hat{Y}_{k,a,s,t}^{inflated}$ the beta-binomial parameters $\hat{\alpha}_{k,a,s,t}$ and $\hat{\beta}_{k,a,s,t}$ using method of moments were first calculated. the variance equation for a beta-binomial were then used to calculate the variance as $Var(\hat{Y}_{k,a,s,t}^{inflated}/\hat{N}_{k,a,s,t}) = \frac{\hat{\alpha}_{k,a,s,t}\hat{\beta}_{k,a,s,t}(\hat{\alpha}_{k,a,s,t}+\hat{\beta}_{k,a,s,t}+\hat{N}_{k,a,s,t})}{(\hat{\alpha}_{k,a,s,t}+\hat{\beta}_{k,a,s,t})^2(\hat{\alpha}_{k,a,s,t}+\hat{\beta}_{k,a,s,t}+1)\hat{N}_{k,a,s,t}}$. The question was then asked: if this data had been observed from a true binomial distribution, what would the effective sample size, $N_{bin_{k,a,s,t}}$, have been to see as much variation in $\hat{p}_{k,a,s,t} = \hat{Y}_{k,a,s,t}^{inflated}/\hat{N}_{k,a,s,t}$ as was seen under the beta-binomial setting? To answer this question and to solve for $N_{bin_{k,a,s,t}}$, the variance of an estimator for the probability of success of a binomial distribution, $\frac{\hat{p}_{k,a,s,t}(1-\hat{p}_{k,a,s,t})}{N_{bin_{k,a,s,t}}}$, was set equal to the beta-binomial variance shown above. By setting $\frac{\hat{\alpha}_{k,a,s,t}\hat{\beta}_{k,a,s,t}(\hat{\alpha}_{k,a,s,t}+\hat{\beta}_{k,a,s,t}+\hat{N}_{k,a,s,t})}{(\hat{\alpha}_{k,a,s,t}+\hat{\beta}_{k,a,s,t})^2(\hat{\alpha}_{k,a,s,t}+\hat{\beta}_{k,a,s,t}+1)\hat{N}_{k,a,s,t}} = \frac{\hat{p}_{k,a,s,t}(1-\hat{p}_{k,a,s,t})}{N_{bin_{k,a,s,t}}}$ and rearranging the equations, the equation for the effective sample size for each binomial SBH observation were arrived at (suppressing k, a, s, t subscripts for simplicity): $N_{bin} = N_{eff} = \frac{\hat{p}(1-\hat{p})(\hat{\alpha}+\hat{\beta})^2(\hat{\alpha}+\hat{\beta}+1)\hat{N}}{\hat{\alpha}\beta(\hat{\alpha}+\hat{\beta}+\hat{N})}$.

Via this procedure, 31% of SBH observations had an N_{eff} that was larger than $\hat{N}$. This was due to practical constraints of having small mortality probabilities and small sample sizes, which can lead to unstable simulation estimates. For these observations, it was set to $N_{eff} = \hat{N}$. Since this was predominantly a problem of small sample sizes, this only affected 1.7% of the data weighted by sample size. The sum of all $\hat{N}$ across all SBH data was 210 million, and the sum of all N_{eff} was 123 million. Thus, accounting for model variance and out of sample residuals reduced total SBH sample size by 41%**.**

C.2. Covariates used for mapping

Description of layers and justification

Geospatial covariates were used to help improve predictions in places without observed data. The covariates that had known associations with child mortality were chosen. These included socio-demographic and environmental measures. The covariates representing health outcomes which directly contribute to mortality were also included.

The following socio-demographic and environmental covariates were included. Travel time to nearest inhabited area of 50,000 or more, which serves as a proxy for remoteness from services;[12] intensity of lights at night, which serves as a measure of electricity consumption and economic development;[13,14] mean years of educational attainment by women of reproductive age;[15,16] the mass per cubic meter of air of particles with a diameter less than 2.5 micrometers ($PM_{2.5}$);[17] ratio of children under-5 to number of women of reproductive age;[18,19] the total population; and urbanicity.[20]

The following health-related covariates were also included: proportion of children aged 12-23 months who have received the third dose of diphtheria-pertussis-tetanus vaccine,[21] which also serves as a proxy for routine health service utilization in children;[22,23] incidence rate of *Plasmodium falciparum* malaria in children under-5;[24,25] and prevalence of stunting in children under-5.[26–28]

Spatial and temporal standardisation

Raw data from spatial covariates came in varying spatial and temporal resolutions. All covariates were prepared to a standardized format and aligned to a 5x5 km global annual grid. If raw input resolution was finer than 5x5 km, the raster was resampled by taking either a neighborhood average or sum.

If covariates were reported in time intervals coarser than year it was interpolated between years. Several covariates were not available out to 2017, and for these the time series were completed by carrying forward data available for the most recent year. Travel time to cities was synoptic and thus did not vary

over time. For this covariate no temporal adjustments were made and it was used as non-temporally changing in the model. Covariates prevalence of diarrhoea, coverage of DPT3 vaccine in children under-5, average years of education for women of reproductive age, and prevalence of stunting in children under-5 were all modeled estimates.

Covariates used for child mortality mapping

Covariate	Source	Paper Citation	Dataset Citation	Processing
access2 (Travel time to nearest settlement)	Oxford	Weiss DJ, Nelson A, Gibson HS, Temperley W, Peedell S, Lieber A, et al. A global map of travel time to cities to assess inequalities in accessibility in 2015. Nature. 2018 18; 553: 333–6.	Available at: https://map.ox.ac.uk/research-project/accessibility_to_cities/	Raster resampled using bilinear interpolation to standard 5 km spatial resolution.
dmspntl (Nighttime lights)	NOAA DMSP satellite program (derived)		Available at: https://www.ngdc.noaa.gov/eog/dmsp/downloadV4composites.html	Rasters resampled using bilinear interpolation to standard 5 km spatial resolution.
fertility	WorldPop (derived)	Lloyd CT, Sorichetta A, Tatem AJ. High resolution global gridded data for use in population studies. Scientific Data. 2017; 4: 170001.	Available at: http://www.worldpop.org.uk/data/get_data/. (Accessed: 25th July 2017)	fertility = (M04 + F04) / WOCBA<br>where:<br>M04 = males, 0-4 years old<br>F04 = females, 0-4 years old<br>WOCBA = women of child bearing age (females 15-49 years old) - from WorldPop 5-year rasters were interpolated using an exponential growth rate to produce annual rasters. 1 km raw data were aggregated to 5 km spatial resolution, preserving total population sums. Once converted to fertility, those areas with a population of zero were filled with the nearest neighboring fertility value where the original population was > 0.
ghslurbanicity (Urbanicity)	European Commission/ GHS	Pesaresi M, Ehrlich D, Ferri S, Florczyk A.J, Freire S, Halkiaet M, et al. Operating procedure for the production of the Global Human Settlement Layer from Landsat data of the epochs 1975, 1990, 2000, and 2014; JRC Technical Report EUR 27741 EN; 2016.	Available at: http://ghsl.jrc.ec.europa.eu/data.php	Rasters resampled using bilinear interpolation to standard 5 km spatial resolution. For intervening data years, the closest available data year was used.
ihmepm25 (ambient air pollution, particulate matter 2.5)	IHME GBD		Data integration model available at: https://arxiv.org/abs/1609.00141	Rasters resampled using bilinear interpolation to standard 5 km spatial resolution.
map_pf_incidence (Malaria incidence)	The Malaria Atlas Project	Gething PW, Casey DC, Weiss DJ, Bisanzio D, Bhatt S, Cameron E, et al. Mapping Plasmodium falciparum Mortality in Africa between 1990 and 2015. New England Journal of Medicine. 2016; 375: 2435–45.	Available at: http://www.map.ox.ac.uk/	Rasters resampled using bilinear interpolation to standard 5 km spatial resolution.
worldpop (Population)	WorldPop	Lloyd CT, Sorichetta A, Tatem AJ. High resolution global gridded data for use in population studies. Scientific Data. 2017; 4: 170001.	World Pop. Get data. Available at: http://www.worldpop.org.uk/data/get_data/. (Accessed: 25th July 2017)	5-year rasters were interpolated using an exponential growth rate to produce annual rasters. 1 km raw data were aggregated to 5 km spatial resolution, preserving total population sums.

Gridded population data

The WorldPop (http://www.worldpop.org.uk/) were used as a source for all gridded population data used throughout this analysis, including as a covariate in modelling and for taking population weighted averages at areal units. Except for use as a model covariate and for polygon resampling, where gridded total population was used, gridded population for the under-5 age band were used. WorldPop provides gridded population estimates at the 1x1 km spatial resolution and at five-year intervals, so the raster were resampled by taking a zonal sum to reach the 5x5 km resolution for analysis and results were used. Interpolation between years were done using an exponential growth rate.

C.3. Statistical model

Geostatistical model

For each modelling region, a discrete hazards model was assumed, with a baseline hazard function varying across the seven age bins. Age bins are (in months) were NN: (0-1), PNN1: (1-6), PNN2: (6-12), 1yr: (12-24), 2yr: (24-36), 3yr: (36-48), 4yr: (48-60).

Each child recorded in CBH data is counted as entering a period-age bin (an age band within a calendar year), and a death event for a given child is assigned if they died within a period-age bin. The number of children entering into, N, and dying within, Y, for each period-age bin from each point location in each survey k within each country c in the data were counted. Likewise, these same variables are estimated indirectly for each point in SBH data.

The number of deaths for children in age band a in year t at point location s was assumed to follow a binomial distribution:

$$Y_{a,s,t} \sim Binomal(N_{a,s,t}, p_{a,s,t})$$

Where $p_{a,s,t}$ can be interpreted as the probability of death in the age bin, conditional on survival to that age bin for a particular space-time location. Using a generalised linear regression modelling framework, a logit link function is used to relate p to a linear combination of effects:

$$Logit(p_{a,s,t}) = \beta^0 + \sum_{a=2}^{7} I_a \beta_a^1 + \beta^2 X_{s,t} + \beta^3 t + \nu_{c[s]} + \nu_{k[s]} + Z_{a,s,t}$$

The first term β^0 is an intercept, representing the mean for the first age band when all covariates equal zero, while $I_a \beta_a^1$ are fixed effects for each age band, representing the mean overall hazard deviation for each age band from the intercept. $\beta^2 X_{s,t}$ are the effects of geospatial covariates. All geospatial covariates were centered and scaled by subtracting their mean and dividing by their standard deviations. $\beta^3 t$ is an overall linear temporal effect to account for broad secular trends. Each ν term represent uncorrelated random effects: $\nu_{c[s]} \sim Normal(0, \sigma_c^2)$ is a country-level random effect applied to all locations, s, within a country; $\nu_{k[s]} \sim Normal(0, \sigma_k^2)$ is a data source-level random effect for the survey k from which the data at location s was observed. Survey-level random effects were used to account for systematic variation or biases across sources and were included in model fitting but not in prediction from fitted models.

The term $Z_{a,s,t} \sim Gaussian\ Process(0, \boldsymbol{K})$ is a correlated random effect across age, space, and time and is modeled as a four-dimensional mean zero Gaussian process with covariance matrix $\boldsymbol{K}$. This term accounts for structured residual correlation across these spatial-age-temporal dimensions that are not accounted for by any of the other model's fixed or random effects. This structure was chosen because the hazard for each age group is expected to vary in space and time, and such spatiotemporal correlation are likely to be similar across ages. $\boldsymbol{K}$ is constructed as a separable process across age, space, and time: $\boldsymbol{K} = \boldsymbol{\Sigma_a} \otimes \boldsymbol{\Sigma_t} \otimes \boldsymbol{\Sigma_s}$. The continuous spatial component is modeled with a stationary isotropic Matérn covariance function: $\text{cov}_s(d_s) = \frac{2^{1-\nu}}{\tau\Gamma(\nu)} \left(\sqrt{2\nu}\frac{d_s}{\kappa}\right)^{\nu} K_\nu \left(\sqrt{2\nu}\frac{d_s}{\kappa}\right)$, where K_ν is the modified Bessel function of the second kind. The Matérn function has three hyperparameters: κ, τ, and ν; the parameter ν is fixed

at 1 and κ and τ are fitted in the model. The overall amplitude of the process is determined by the marginal variance, $1/\tau > 0$ while the distance required between two spatial locations, $d_s = |s_i - s_j| > 0$, before their correlation drops below any specific threshold is governed by the scaling parameter, $\kappa > 0$. The age and temporal effects were each assumed to be discrete auto-regressive order 1 (AR1) processes where the discrete steps are taken annually in time and across the seven age groups. The AR1 covariance functions are each defined by single correlation parameters: ρ_a and ρ_t for age and time, respectively.

The following priors for model parameters are specified:

$$\log(\kappa) \sim Normal(0,1)$$

$$\log(\tau) \sim Normal(0,1)$$

$$\log\left(\frac{1+\rho_a}{1-\rho_a}\right) \sim Normal(1,1)$$

$$\log\left(\frac{1+\rho_t}{1-\rho_t}\right) \sim Normal(1,1)$$

$$\beta^{0,1,2,3} \sim Normal(0,9)$$

$$\log(\sigma_c) \sim Normal(-4,4)$$

$$\log(\sigma_k) \sim Normal(-4,4)$$

The transformations on priors were used in order to perform optimization on a likelihood and maintain constrained parameters. Log-transforms were used to constrain certain parameters $(\kappa, \tau, \sigma_c, \sigma_k)$ positive. Transformations on the ρ parameters constrained values between -1 and 1 and centered at zero. Priors on all fixed effects were weakly informative, since covariates were centered and scaled. Priors for the standard deviation of Gaussian random effects (σ_c, σ_k) were structured such that they had a small mean and a long tail because these effects were thought would be minimal considering other model components, but did not want to completely constrain their size. The models were fit using TMB[6] package in R version 3.4.

MAP inference were used, using a maximum likelihood estimation with an augmented optimisation objective (log-likelihood function) which incorporated prior distributions for all model parameters. TMB uses automatic differentiation to find the Laplace approximation to the marginal log-likelihood with respect to the hyperparameters of any random effects specified in the model. A non-linear optimisation routine is then used to maximise the marginal log-likelihood to derive point estimates for all model parameters. The $Z_{a,s,t}$ random effects were fitted using the stochastic partial differential equations[29] approximation to Gaussian process residuals. A finite elements mesh was constructed for the SPDE approximation based on a polygon boundary defining the spatial limits of the modelling region. The mesh had a minimum edge length of 100 km over land. Finally, a generalised delta-method was used to approximate the joint precision matrix of all model parameters.

Using the joint precision matrix and point estimates, 1000 draws were generated from all model parameters using a multivariate-normal approximation. These model parameter draws were used to predict corresponding draws of mortality probabilities across all age groups for each grid cell in each year. In other words, for each age bin in each year 1000 surfaces of mortality probability estimates were estimated, each surface corresponding to one draw from the posterior parameter estimates. Within each surface, or "candidate map", the correlation structure across space-age-time is maintained.[30]

This model was fit across a geographic region that included India. This allowed for the model to draw predictive power from data observations in neighboring countries in addition to India.

Polygon resampling

All data to GPS coordinates (latitude and longitude) were matched wherever possible. These precisely located data were referred to as ‘point data’. In cases where point data were not available, data points were matched to the smallest possible areal unit (also referred to as polygons). In most cases these polygons represent administrative sub-divisions. Since the geostatistical model were fit, it requires point data, re-sampled polygon data into pseudo-point data. This approach has been used before in mapping studies of child mortality,[1] child growth failure,[3] education,[4] and diarrhoea burden.[2]

The approach to producing pseudo-points proceeded as follows for each polygon-level observation: 10,000 locations with weights proportional were sampled to the underlying population (as measured by WorldPop) at 1x1 km spatial resolution. The k-means clustering was used to derive a reduced set of points, with k set to 1 per 1000 grid cells. Each of these resulting clusters then served as pseudo-point data. Each pseudo-point was assigned a weight proportional to the number of sampled locations contained in it via k-means clustering. The observed mortality probability for the polygon as a whole was assigned to each point, and the sample size for each point was taken as the sample size for the polygon multiplied by the weight. The sample sizes for all pseudo-points derived from a polygon thus sum to equal the sample size of the polygon as a whole.

This resampling process converts observations at the administrative unit (or polygon) level into multiple point observations located within the original polygon. The step is undertaken because the geostatistical model described below requires input survey data that has been geolocated to points. This geostatistical model, originally described in Lindgren et al. (2011),[29] generates continuous pixel-level estimates of a process based on discrete point observations.

C.4. Model results

Coefficient values for each model region are given in below figures. These are exponentiated coefficients from a model with a logit link and as such should be interpreted as odds ratios. Geospatial covariates enter the model as centered and scaled by the mean and standard deviations, as such coefficients can be interpreted as the odds ratio for a one standard deviation increase in covariate value. The horizontal black line on the figure indicates an odds ratio of 1, or no effect on mortality. Any effect size below the line can be interpreted as protective, and effects above the line are associated with increased mortality. It is emphasized that, due to high correlation and issues of circularity in production of covariate surfaces, drawing inference on effect sizes or directions is not recommended.

Posterior values for all model hyper-parameters are shown in below figure. Variation in country random effects was substantial in the South Asia modeling region, indicating that there were significant country-level intercept shifts that were not accounted for by spatial covariates alone. Variation in country random effects was larger than variation in the data source-specific random effects. Data-source-specific random effects account for systematic bias across sources and were not included in predictions. The posterior estimates for the four parameters guiding the Gaussian Process random effect were shown. The ρ parameter for age was always above 0.75 and the ρ parameter for year was always above 0.98, indicating high correlation across age bins, and very high correlation across annual time steps. This was expected given the way that data were prepped, where a single data source contributes a time trend of information for all the different age bins.

All fixed effect parameter estimates are exponentiated. Geospatial covariates enter the model as centered and scaled by their standard deviations. Additional fixed effects not included in this plot are the overall intercept and age-bin-specific intercepts.

Fitted estimates of covariate coefficients in the South Asia modeling region

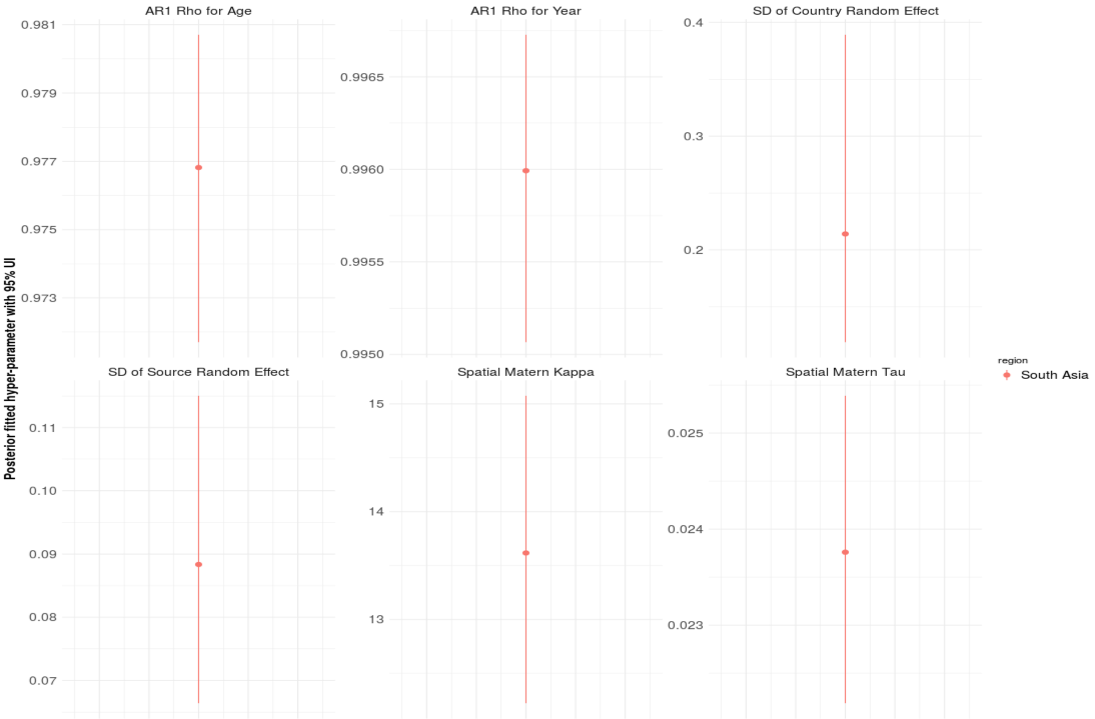

Posterior hyper-parameter fits for the South Asia model region

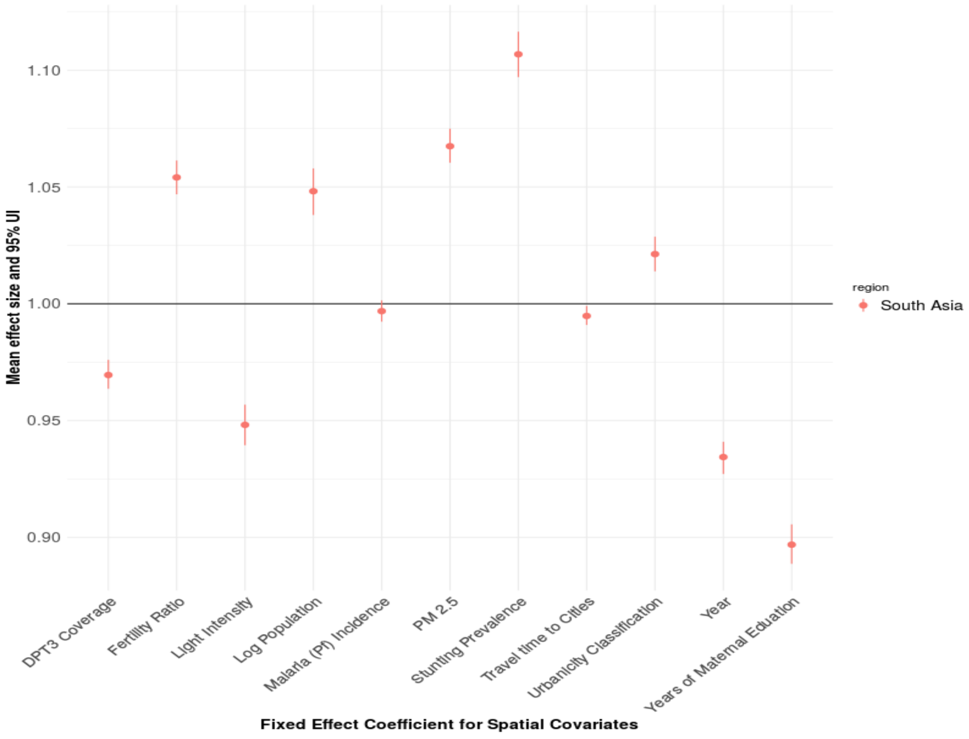

C.5. Post-estimation

Combining age groups

For reporting in this paper, for presenting results, the focus was for three age bands of mortality: neonatal, infant, and under-5. Neonatal is taken as the first age band predicted from the model, and infant and under-5 are derived from a combination of age bands using the synthetic cohort approach to estimating period-specific mortality probabilities. All aggregation across ages was done at the draw level for each space-time grid cell. For each space-time grid cell (gc) and posterior predictive draw (m) of estimated mortality probability $\hat{p}^{m,gc}$, infant mortality ($1\hat{q}0^{m,gc}$) is calculated as $1 - \prod_{a=1}^{3}(1 - \hat{p}_a^{m,gc})$, and under-5 mortality ($5\hat{q}0^{m,gc}$) is calculated as $1 - \prod_{a=1}^{7}(1 - \hat{p}_a^{m,gc})$. Since these calculations are done at the grid-cell-draw level, correlation structure within each draw is maintained by this processing.

Calculating numbers of deaths

In order to produce estimates of death counts, each grid-cell-draw estimate of mortality probability, q, were first converted, predicted directly from the model (and aggregated into the three age bins) into estimates of yearly mortality rates, m. For infants and under-5s the following formula[11] was used

$$m = 1/(-n + a + n/q)$$

where n is the width of the age band (1 and 5 for infant and under-5, respectively), and a is the average number of years lived by those who died within the age band. For a we used the country-year-specific estimate produced as part of the GBD study.[7] m in the neonatal band were calculated as $-12 * log(1 - q)$.[11] For provisional estimates of deaths, m at each age-space-time-draw were multiplied by high-resolution population estimates for under-5s available from the WorldPop project. WorldPop does not publish estimates of neonatal or infant population, so within-country relative spatial distributions of populations were assumed in those age bins equivalent to those seen across the broader under-5 populations. Furthermore, WorldPop does not publish uncertainty around their grid-cell-level population estimates, so it was not possible to propagate population count uncertainty into these estimates of death counts. Finally, grid-cell-level death counts were scaled by national death count estimates available from the GBD.

C.6. Summarising results and aggregating to administrative subdivisions

Summary maps for probabilities of death and death counts were produced by taking summary statistics across draws at either the grid cell or aggregated levels. For example, mean grid-cell-level maps were derived by taking the mean across all draws in each age-time grid cell, while uncertainty intervals were derived from the 2.5th and 97.5th percentiles at each age-time grid cell.

Spatially aggregated estimates, such as those at the country and first and second administrative areal unit were made by taking population weighted averages using WorldPop gridded population data of the value of interest for each draw across each areal unit, such that draw-level estimates were produced for each areal unit. Grid cells were assigned to an area based on the location of their respective centroids. These were subsequently summarised across draws. In places where borders intersected grid cells, death counts in each grid cell were split based on the share of land area in each adjacent area. This approach was used to produce aggregated estimates for the districts and states of India.

C.7. Validation

Five-fold cross-validation were utilised to assess and compare model performance with respect to estimating local trends of age-specific mortality. Each fold was created by combining complete surveys into subsets of ~20% of data sources from the input data. Holding out entire surveys at a time served as a comparable approximation to the type of missingness in our data, essentially helping understand how this model estimates of mortality probabilities compared to empirical estimates of mortality probability from a new survey which did not inform the model.

To address this, each estimate were aggregated from each survey to the country level as well as the first and second administrative level for each year of available data. At each level of aggregation, the weighted mean of empirical and estimated mortality probabilities were taken based on sample sizes of data points. Estimates were aggregated at the draw level, and then means were taken at the aggregated level across draws.

C.8. Calibration with the state-level and national-level estimates

For India, a calibration of these estimates was then performed to the state and national estimates from the GBD 2017 study.[7] This allowed us to take advantage of the state-level and national-level information such as vital registration data which are used as part of the GBD estimation process but vital registration and sample registration system data were not incorporated into geospatial estimation, as completeness of these sources may vary locally in space. To calibrate between GBD estimates and geospatial estimates, each grid cell was assigned to a state based on the location of the grid cell centroid. The mean estimates were then generated for each state-age-year. For each state-age-year estimate, a scaling factor was generated which is a multiplicative constant defined as the ratio between the population-weighted mean of the raw 5x5 km gridded estimates when aggregated to the state level and the state level GBD estimates of child mortality. These scaling factors allow the 5x5 km mortality estimates to leverage additional data that has been incorporated in state-level estimation, such as data from the sample registration system and vital registration system. For all 5x5 km estimates in the years 2000-2017, the results were harmonized to state-level estimates from GBD 2017. These scaling factors were applied to each grid-cell draw, ensuring that aggregated mean estimates from our model were identical to the mean estimate from the GBD 2017 study for the states of India. This approach led to calibration with the GBD 2017 national estimate for India as well. For projected estimates in 2030, the 5x5 km gridded estimates were harmonised to national-level estimates from the GBD 2017 SDG projections as state-level estimates were not projected to 2030 as part of the GBD 2017 SDG projections.

References

1. Golding N, *et al.* Mapping under-5 and neonatal mortality in Africa, 2000–15: a baseline analysis for the Sustainable Development Goals. *Lancet* 2017*;* 390, 2171–2182.
2. Reiner R C, *et al.* Variation in childhood diarrheal morbidity and mortality in Africa, 2000–2015. *N. Engl. J. Med* 2018*;* 379, 1128–1138.
3. Osgood-Zimmerman A, *et al.* Mapping child growth failure in Africa between 2000 and 2015. *Nature* 2018; 555, 41–47.
4. Graetz N, *et al.* Mapping local variation in educational attainment across Africa. *Nature 2018;* 555, 48–53.
5. Burstein R, Wang H, Jr R CR, Hay SI Development and validation of a new method for indirect estimation of neonatal, infant, and child mortality trends using summary birth histories. *PLOS Med* 2018*;* 15, e1002687.
6. Kristensen, K. *et al.* TMB: Template Model Builder: A general random effect tool inspired by 'ADMB*'*. 2018. https://rdrr.io/cran/TMB/.
7. GBD 2017 Mortality Collaborators. Global, regional, and national age-sex-specific mortality and life expectancy, 1950–2017: a systematic analysis for the Global Burden of Disease Study 2017. *Lancet* 2018*;* 392, 1684–1735.
8. Ahmad OB, Lopez AD, Inoue M. The decline in child mortality: a reappraisal. *Bull. World Health Organ 2000;* 78, 1175–1191.
9. Somoza JL. Illustrative analysis of infant and child mortality in Colombia; draft for comments. 1980; published online April. https://repositorio.cepal.org//handle/11362/32357.
10. Infant and child mortality: levels, trends and demographic differentials. *POPLINE.* https://www.popline.org/node/399179. (Accessed: 21st January 2019)
11. Preston SH, Heuveline P, Guillot M. Demography: measuring and modeling population processes. Malden, MA: Blackwell Publishers, 2001.
12. Karra M, Fink G, Canning D. Facility distance and child mortality: a multi-country study of health facility access, service utilization, and child health outcomes. *Int. J. Epidemiol* 2017*;* 46, 817–826.
13. Wang L. Determinants of child mortality in LDCs: Empirical findings from demographic and health surveys. *Health Policy 2003;* 65, 277–299.

14. Proville J, Zavala-Araiza D, Wagner G. Night-time lights: A global, long term look at links to socio-economic trends. *PLoS ONE* 2017*;* 12.
15. Browne AW, Barrett, H. R. female education in Sub-Saharan Africa: The key to development? *Comp. Educ* 1991*;* 27, 275–285.
16. Gakidou E, Cowling K, Lozano R, Murray CJ. Increased educational attainment and its effect on child mortality in 175 countries between 1970 and 2009: a systematic analysis. *Lancet.* 2010*;* 376, 959–974.
17. Heft-Neal S, Burney J, Bendavid E, Burke M. Robust relationship between air quality and infant mortality in Africa. *Nature.* 2018*;* 559, 254–258.
18. Sah RK. The effects of child mortality changes on fertility choice and parental welfare. *J. Polit. Econ. 1991;* 99, 582–606.
19. Coale AJ Demographic transition. In: Social economics. London: Palgrave Macmillan, 1989.
20. Eckert S, Kohler S. Urbanization and health in developing countries: a systematic review. *World Health Popul 2014;* 15, 7–20.
21. Akachi Y, Steenland M, Fink G. Associations between key intervention coverage and child mortality: an analysis of 241 sub-national regions of sub-Saharan Africa. *Int. J. Epidemiol.* 2017; published online Dec 21.
22. Ehreth J. The global value of vaccination. *Vaccine. 2003;* 21, 596–600.
23. Madhi SA, Levine, OS, Hajjeh R, Mansoor OD, Cherian T. Vaccines to prevent pneumonia and improve child survival. *Bull. World Health Organ.* 2008*;* 86, 365–372.
24. Akachi Y, Atun R. Effect of investment in malaria control on child mortality in sub-Saharan Africa in 2002-2008. *PloS One.* 2011*;* 6, e21309.
25. Eisele TP, *et al.* Estimates of child deaths prevented from malaria prevention scale-up in Africa 2001-2010. *Malar. J* 2012; 11, 93.
26. GBD 2017 Risk Factors Collaborators. Global, regional, and national comparative risk assessment of 84 behavioural, environmental and occupational, and metabolic risks or clusters of risks for 195 countries and territories, 1990–2017: a systematic analysis for the Global Burden of Disease Study 2017. *Lancet* 2018; 392: 1923–94.
27. Black RE, *et al.* Maternal and child undernutrition: global and regional exposures and health consequences. *Lancet* 2008*;* 371, 243–260.
28. Pelletier DL, Frongillo EA. Changes in child survival are strongly associated with changes in malnutrition in developing countries. *J. Nutr* 2003; 133, 107–119.
29. Lindgren F, Rue H, Lindström J. An explicit link between Gaussian fields and Gaussian Markov random fields: the stochastic partial differential equation approach. *R. Stat. Soc* 2011; 73, 423–498.
30. Patil AP, Gething PW, Piel FB, Hay SI. Bayesian geostatistics in health cartography: the perspective of malaria. *Trends Parasitol* 2011; 27, 246–253.
31. Foreman KJ, Marquez N, Dolgert A, Fukutaki K, Fullman N, McGaughey M, Brown JC. Forecasting life expectancy, years of life lost, and all-cause and cause-specific mortality for 250 causes of death: reference and alternative scenarios for 2016–40 for 195 countries and territories. *Lancet* 2018; 392:2052-2090.

D. Projection of under-5 and neonatal mortality rate at 2030

D1. Projections at the national level

GBD 2017 produced projections for the health-related Sustainable Development Goal (SDG) indicators up to 2030 for all countries based on past trends of outcome and covariates, using a new advanced modelling framework (Lancet 2018; 392: 2091–138). The steps used to produce projections for the under-5 mortality are as follows.

The annual change from the previous year was first calculated from 1990 to 2017 using the logit of the rate for each year. The weight for each year was calculated using the following formula. Then annual rate of change was used to project the rate up to 2030 for India as part of GBD, giving higher weight to the more recent annual rate of change to project from 2018 to 2030.

$$weight_{year} = (t - 1990 + 1)^{\omega}$$

Where ω is the weight function, the value of which denotes how much higher impact recent years would have compared with the past years when calculating the annual rate of change for the projection. To determine the appropriate value of ω for each indicator, an out-of-sample predictive validity test was done using data from 1990 to 2007 to predicted values for the years from 2008 to 2017. Assuming a range of values, in the increments of 0.25, from 0 to 10 for ω, the best predicted value for the period 2008 to 2017 was tested for each indicator. The final value for the weight function (ω) specific to each indicator for projection was chosen that minimised the root mean squared error in the 2008–2017 projections based on the 1990–2007 data. Using this approach, the weight function computed for under-5 mortality rate and neonatal mortality rate was 2. The inverse of the weighted logit mean of the annualised rate of change from 1991 to 2017 was then applied to the years 2018 onward to estimate the rate of child mortality indicators up to 2030.

D2. Projections at the subnational level

To estimate mortality rates in the year 2030 at 5x5 km grids, a simple projection methodology that has been used previously for such geospatial analysis was performed using estimated annual rates of change (AROCs) from 2000 to 2017 to obtain the estimates for subsequent years.[1]

A logit-transformed annualised rate of change were calculated from 2000 to 2017 at each pixel 'i' for each of the age-specific mortality indicators (U5MR and NMR) 'm' across each of the 1000 draws 'd' to generate the projected estimates up to 2030,

$$AROC_{a,i,d}^{logit} = \frac{logit\left(Q_{a.i.d,2017}\right) - logit(Q_{a,i,d,2000})}{2017 - 2000}$$

The raw projections to 2030 were then calculated by applying the annualised rate of change estimates to 2017 estimates for each draw using the following projection calculation:

$$Q_{a,i,d,2025} = logit^{-1}\left(logit(Q_{a,i,d,2017}) + AROC_{a,i,d}^{logit} * (2025 - 2017)\right)$$

The population-weighted aggregates of mortality from these pixel-level projections were created at the district- and state-levels for draws using WorldPop estimates for population in each pixel.[3] The draw-level projections for 2025 and 2030 were then summarized using the mean, 2.5th percentile, and 97.5th percentiles of the draw estimates. The mean estimates were harmonised at the national level to estimates produced by GBD by applying the relevant raking factor.[2]

References

1. Osgood-Zimmerman A, Millear AI, Stubbs RW, Shields C, Pickering BV, Earl L, & Browne AJ. Mapping child growth failure in Africa between 2000 and 2015. *Nature* 2018; 555, 41.
2. Foreman KJ, Marquez N, Dolgert A, Fukutaki K, Fullman N, McGaughey M, Brown JC. Forecasting life expectancy, years of life lost, and all-cause and cause-specific mortality for 250 causes of death: reference and alternative scenarios for 2016–40 for 195 countries and territories. *Lancet* 2018; 392, 2052-2090.
3. Lloyd CT, Sorichetta A, Tatem AJ. High resolution global gridded data for use in population studies. Scientific Data 2017; 4: 170001.

E. Uncertainty intervals

Point estimates for each quantity of interest were derived from the mean of the draws, while 95% uncertainty intervals (UIs) were derived from the 2.5th and 97.5th percentiles of the 1,000 draw level values. Uncertainty in the estimation is attributable to sample size variability within data sources, different availability of data by age, sex, year, or location, and cause-specific model specifications. The UIs were determined for components of cause-specific estimation based on 1,000 draws from the posterior distribution of cause specific mortality by age, sex, and location for each year included in the GBD 2017 analysis. With this approach, uncertainty could be quantified and propagated into the final quantities of interest.

2. Cause of death estimation methods

The materials presented here are adapted from the following source:

- GBD 2017 Causes of Death Collaborators. Global, regional, and national age-sex-specific mortality for 282 causes of death in 195 countries and territories, 1980–2017: a systematic analysis for the Global Burden of Disease Study 2017. *Lancet* 2018; 392: 1736–88.

A. List of ICD codes mapped to the GBD cause of death list for under-5 and neonates

The codes used by GBD Study 2017 from the 9th and 10th revisions of the International Statistical Classification of Diseases and Related Health Problems (ICD) for under-5 deaths are listed below:

Cause	ICD 10	ICD 9
Neonatal preterm birth	P05-P07.39, P22-P22.9, P25-P28.9, P61.2, P77-P77.9	764-765.9, 769-770, 770.2-770.9, 776.6, 777.5-777.53
Neonatal encephalopathy due to birth asphyxia and trauma	P02-P03.9, P10-P21.9, P24-P24.9, P52-P52.9, P90-P91.9	761.7-763.9, 767.0-768.9, 768.2-768.9, 772.1-772.2, 779.0-779.2
Neonatal sepsis and other neonatal infections	K29.0-K29.00, K29.1-K29.20, P36-P36.9, P38-P39.9, P77-P78.1	535.0-535.00, 535.3-535.30, 771, 771.4-771.89, 777.5-777.7
Hemolytic disease and other neonatal jaundice	P55-P59.9	773-774.9
Other neonatal disorders	P00-P01.9, P04-P05.9, P07-P07.18, P08-P09, P19-P19.9, P29-P29.9, P50-P54.9, P60-P61.1, P61.3-P61.9, P70-P72.9, P74-P74.9, P75.0-P76.9, P78-P78.9, P80-P81.9, P83-P84, P92-P92.9, P94-P94.9, P96, P96.3-P96.9, P99.9	655.3-655.63, 760-761.6, 764-765.20, 766-766.9, 771.9-772.9, 775-775.1, 775.4-776.5, 776.7-777.4, 777.6-779, 779.3-779.5, 779.7-779.9
Lower respiratory infections	A48.1, A70, B96.0-B97.6, J09-J22.9, J85.1, P23-P23.9, U04-U04.9, Z25.1	079.82, 466-470.0, 480-484, 484.1-490.9, 510-513.9, 513.0-513.9, 770.0, V01.82, V04.7, V04.81, V12.61
Diarrhoeal diseases	A00-A00.9, A02-A09, Z22.1, Z23.0	001-001.9, 003.8-009.9, V01.0, V01.83, V02.0, V02.2-V02.3, V03.0, V74.0
Measles	B05-B05.9, Z24.4	055-055.9, 484.0, V04.2, V73.2
Tetanus	A33-A35.0, Z23.5	037-037.9, 771.3, V03.7
Congenital birth defects	P96.0, Q00-Q07.9, Q10-Q15.9, Q17-Q18.9, Q20-Q28.9, Q30-Q45.9, Q50-Q56.4, Q63.3, Q64-Q64.19, Q65-Q87.89, Q89-Q89.8, Q90-Q93.9, Q95-Q99.9, Z13.7-Z13.79, Z14-Z15.89, Z82.7-Z82.79	237.7-237.79, 740-744, 744.00-753, 753.5, 754-759.9, V13.6-V13.69, V18.61, V18.9, V19.5, V19.7-V19.8, V82.3
Injuries	V01-Y87.2	E000-E999

B. Cause of death estimation

Cause of death estimation modelling methods for the under-5 and neonates deaths due to the major causes (neonatal disorders, lower respiratory infection, diarrhoeal diseases, measles, tetanus, congenital birth defects, and injuries) are described below. Details about the other cause of deaths are available in the GBD 2017 cause of death capstone paper (Lancet 2018; 392: 1736–88).

Data

The data sources used for estimation of cause of child deaths in India are listed in the data sources table on pp 69-83.

The Sample Registration System (SRS) in India is operated by the Office of the Registrar General of India working under the Ministry of Home Affairs, Government of India.[1] Cause of death data from SRS verbal autopsy on 455,460 deaths including 71,052 under-5 deaths from the rural and urban populations of every state of India from 2004 to 2013, which included 71,032 under-5 deaths were provided by the Office of the Registrar General of India for analysis by the India State-Level Disease Burden Initiative. Using the 2001

census, 7597 geographic units, 4433 (58·4%) of which were rural, were sampled for the 2004–13 SRS to represent the population of each state and union territory of India, ultimately with a sample of 6·7 million people that was equivalent to 0·7% of India's population. The SRS cause of death data were available for 2004–06, 2007–09, and 2010–13 years groups. 2005, 2008, and 2012 were used as midpoint years for these three time periods. The field investigation consists of continuous enumeration of births and deaths in selected sample units by the enumerators and an independent survey every six months by SRS supervisors. The data obtained by these two independent functionaries are matched. The unmatched or partially matched events are re-verified in the field by a third party or jointly by the supervisor and the enumerator to get an unduplicated count of correct events. For every death occurring in the households, a verbal autopsy data was collected through a structured interview with a family member or close acquaintance of the deceased trained fieldworker by a trained field-worker. The verbal autopsy questionnaire was independently reviewed by two trained physicians to determine the probable cause of death based on the guidelines of ICD-10. In case of disagreement between the two physicians, the final ICD code was assigned by a third senior physician. The inclusion of SRS 2004–13 data in this analysis offers a comprehensive picture of causes of death in India. In the absence of a fully functional VR system, verbal autopsy can provide reasonable population level cause of death distribution. Since the cause of death data are unavailable before the year 2000 in India, the estimation of child mortality before this period are mainly driven from covariates.

Building upon the previous Model Registration System of the Office of the Registrar General of India, data on causes of death in rural areas of India were collected by the Survey of Causes of Deaths (Rural) from 1980 to 1998 in a sample of villages of selected primary health centers using the verbal autopsy method.[2] In each state the number of primary health centres selected were based on the norm of atleast on unit per million of the population. Almost all the districts within the state were covered. A paramedical field agent from the primary health centre was designated with the task of interviewing the family of the decreased, and recording the symptoms, conditions, anatomical site and duration of illness using a structured questionnaire. A checklist of the non-medical causes of death based on ICD-9 was used. The probable cause of death was ascertained by applying the structured questionnaire to the symptoms and circumstances recorded. The primary health centre statistician was designated to do a half-yearly verification of the household list and the events reported by the field agent. The correctness of the cause of death assigned by the field agent was certified by the medical officer of the primary health centre. The survey design used was reasonably valid and fulfilled the design criteria for a good verbal autopsy system.[2] This survey provides reasonable data on the cause of death due to accidents and injuries, as these symptoms can easily be recognized by a lay person.[3] In 1999, Survey of Causes of Deaths (Rural) was merged with the SRS verbal autopsy cause of death data collection, covering both rural and urban areas.

The India Cause of Death Data Set Version 1.3 available from the Institute of Health Systems contains data from the Survey of Cause of Death (Rural) for the years 1980-1998, the Indian National List dataset of diseases (INL-9), and the Medical Certification of Cause of Death (MCCD) for the year 1991 to 1996. The data from the Survey of Cause of Death (Rural) for the years 1980-1995 were used from this source for estimating road injury deaths in the states of India.

The MCCD system under the Office of the Registrar General of India has data mostly for the urban parts of the states and union territories beginning in 1980. MCCD covered only 22% of the deaths in India in 2015, with the coverage less than 20% in 15 states, 20–50% in ten states and union territories, and more than 50% in some states and union territories. Deaths reported in this data source are medically certified and are considered VR data (Lancet 2017; 390: 2437–2460).

The Indian Council of Medical Research Study on Causes of Death by Verbal Autopsy was carried out in five states of India namely, Assam, Bihar, Maharashtra, Rajasthan, and Tamil Nadu to assess probable causes of deaths in 2003.[3] A stratified multi-stage sampling design was adopted. Six-monthly survey was conducted in each state to collect information during the first and the second half of the year 2003. The reporting of deaths was supplemented by death reports from the health workers or private practitioners or prominent persons in the locality and list of deaths from the Municipal Corporation in urban area and Gram Panchayat in villages during a fixed reference period. After identifying the households, a close relative or caretaker or neighbour who attended the deceased person during the terminal phase preceding death was contacted for details information of the illness or events leading to death. The verbal autopsy method used for the data collection was exhaustive containing verbatim questions on symptoms, signs and modules. Based on the responses about the signs and symptoms preceding death, the cause of death was determined by physicians according to ICD-10 codes. For checking the completeness of deaths reported in

the survey, estimated deaths based on sampling design by age, sex and place of residence were compared with those estimated in SRS for 2003 for each of the five selected states.

In addition to the above major data sources, a number of state-specifc verbal autopsy studies were also included in the analysis. These data sources are listed on pages 69-83 of this appendix and their details are available in the respective publications.

Data were standardised and mapped according to the GBD causes of death ICD mapping method. These data were then age-sex split, and appropriate redistribution of garbage code data was performed. Data points that violated well-established age or time trends or that resulted in extremely high or low cause fractions were considered to be outliers. Outliers were identified by systematic examination of data points for all location-years.

Modelling strategy

Cause of death was estimated using CODEm developed for the GBD study. Ensemble modelling is a method where a large number of model specifications are systematically tested and reviewed based on their out-of-sample predictive validity; models that perform best are subsequently incorporated into a weighted ensemble model with the highest weights assigned to models with the best out-of-sample prediction error. A description of CODEm follows.

First, all available data are identified and gathered to be used in the modelling process. Though the data may vary in quality, they all contain some signal of the true epidemiological process.

Second, a diverse set of plausible models are developed to capture well documented associations in the estimates. Using a wide variety of individual models to create an ensemble predictive model has been shown to outperform techniques using only a single model both in cause of death estimation and in more general prediction applications.

Third, the out-of-sample predictive validity is assessed for all individual models, which are then ranked for use in the ensemble modelling stage. Finally, differently weighted combinations of individual models are evaluated to select the ensemble model with the highest out-of-sample predictive validity.

For some causes, separate models were run for different age ranges when there was reason to believe that the relation between covariates and death rates might be different in different age ranges, for example, in children compared with adults. Separate models are developed for countries with extensive, complete, and representative VR for every cause such that uncertainty can better reflect the more complete VR in these locations.

As many factors covary with a particular cause of death, a large range of plausible statistical models are developed for each cause. For the CODEm framework, four families of statistical models are developed using covariates. These are mixed effects linear models of the natural log of the death rate, mixed effects linear models of the logit of the cause fraction, ST-GPR models of the log of the death rate, and ST-GPR of the logit of the cause fraction. All plausible relationships between covariates and relevant cause are identified, and all possible permutations of selected covariates are tested in linear models where the logit cause fraction or log death rate is the response variable. Because all permutations of covariates, multicollinearity between covariates were tested and found to produce implausible signs on coefficients or unstable coefficients. All models where the sign on the coefficient is in the direction expected based on the literature and where the coefficient is statistically significant at $p < 0.05$ are retained. The covariate selection was run for both cause fractions and death rates and then both mixed effects only and ST models were created for each set of covariates.

The performance of all component models and ensembles is evaluated using out-of-sample predictive validity tests. Thirty percent of the data are excluded from the initial model fits, and half of that (15% of total) is used to evaluate and rank component models and then build ensembles. Data are held out from the analysis using the pattern of missingness for each cause in the cause of death database. Out-of-sample predictive validity testing is repeated until stable model results have been obtained. The out-of-sample performance tests include the root mean squared error of the log of the cause-specific death rate, the direction of the trend in the prediction compared to the data, and the validity of the 95% uncertainty interval

(UI). For every model, the in-sample RMSE of the log death rates and the out-of-sample performance were show in the 15% of data that not used in the model building process.

After component models are ranked on their out-of-sample predictive validity they are weighted based on their ranking and each component model contributes a portion to the final estimate. How much each sub model contributes is a function of its relative ranking as well as the value of psi chosen, which dictates that distribution of rankings.

Using the second half of the holdout data (15% of total), the differently weighted ensembles and different values of psi are tested using the same predictive validity metrics as the component models. The ensemble with the best average trend and RMSE is chosen as the final ensemble weighting scheme.

After a model weighting scheme has been chosen, each model contributes a number of draws proportional to its weight such that 1,000 draws are created. The mean of the draws is used as the final estimate for the CODEm process and 95% UI are created from the 0.025 and 0.975 quantiles of the draws. The final assessment of ensemble model performance is the validity of the UIs; ideally, the 95% UI for a model would capture 95% of the data out-of-sample. Higher coverage suggests that UIs are too large and lower than 95% suggest UIs are too narrow.

CODEm models estimated the individual cause-level mortality without taking into account the all-cause mortality. GBD uses the CoD Correct algorithm to ensure that all individual causes add up to the all-cause mortality. After generating underlying cause of death estimates and accompanying uncertainty, this algorithm combines these models into estimates that are consistent with the levels of all-cause mortality estimated for each age-sex-year-location group. Using 1000 draws from the posterior distribution of each cause and 1000 draws from the posterior distribution of the estimation of all-cause mortality, CoD Correct rescales the sum of cause specific estimates to equal the draws from the all cause distribution. Further details of CoB Correct algorithm can be found in the appendix to the GBD 2017 cause of death capstone paper (Lancet 2018; 392: 1736–88).

References

1. Office of the Registrar General & Census Commissioner, Ministry of Home Affairs, Government of India. 2019. http://www.censusindia.gov.in/vital_statistics/SRS_Statistical_Report.html (accessed Dec 27, 2019).
2. Mahapatra P, Chalapati Rao P. Cause of death reporting systems in India: a performance analysis. *Natl Med J India* 2001; 14:154-62.
3. Indian Council of Medical Research. Study on Causes of Death by Verbal Autopsy in India. New Delhi, India; 2009. https://www.icmr.nic.in/sites/default/files/reports/Contents_NCD.pdf (accessed Dec 27, 2019).

B.1. Neonatal disorders

The steps in the estimation of neonatal disorders deaths are shown in the following flowchart:

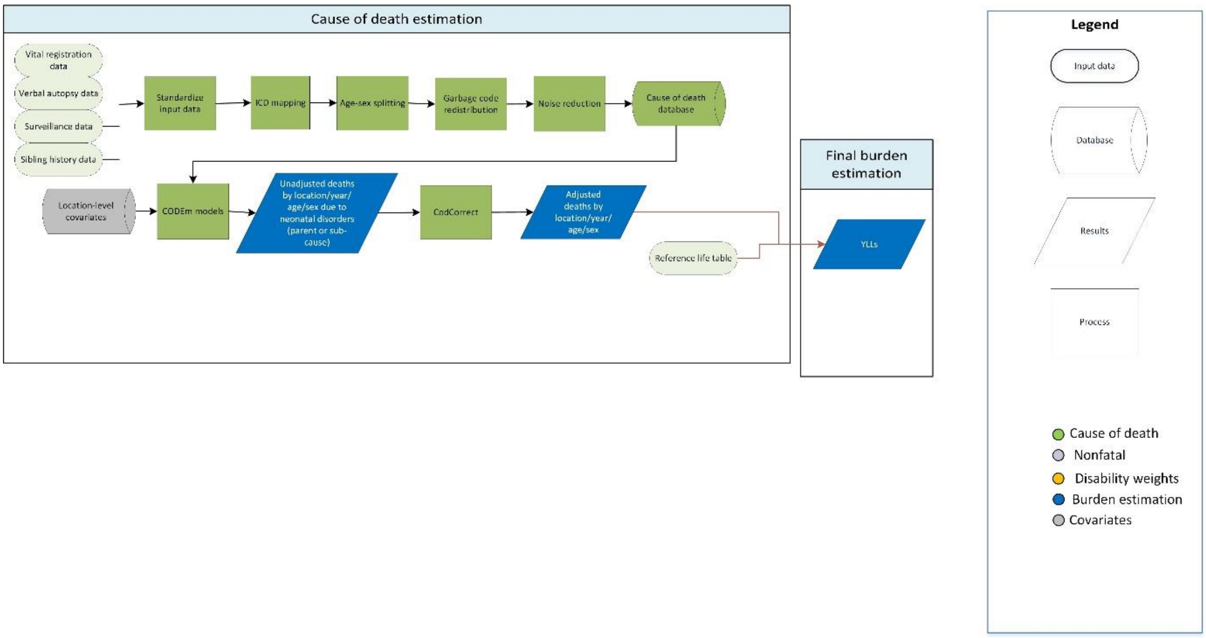

Data

Mortality for five causes are modeled within “neonatal disorders” including preterm birth complications, neonatal encephalopathy and birth trauma, neonatal sepsis and other infections, hemolytic disease and neonatal jaundice, and other neonatal disorders. An overall neonatal disorders “parent” envelope is also estimated, to which all neonatal causes are squeezed.

For the neonatal disorders envelope, preterm birth complications, neonatal encephalopathy and birth trauma, neonatal sepsis and other infections, hemolytic disease and neonatal jaundice, and other neonatal disorders, vital registration and surveillance were the majority of data sources used for GBD 2017 to estimate number of deaths from each condition. In India, only verbal autopsy was used for the estimation of deaths due to neonatal disorders. Only deaths among males and females under age 5 were modelled, in four separate age groups: early neonatal period, late neonatal period, post-neonatal period, and 1-4 years. Data points were selected as outliers if they were implausibly high, low, or significantly conflicted with established age or temporal patterns.

Modelling strategy

For GBD 2017, the standard CODEm modelling approach was used to model each of the neonatal conditions. This same method was employed in GBD 2013, 2015, and 2016. Varying levels of data quality and coding issues may still have affected the results. Validation studies suggest that verbal autopsy methods tend to be less accurate for cause of death ascertainment in the neonatal age groups.[1–4]

The following covariates were used for the estimation process of neonatal disorder:

Level	Covariate	Direction
1	Indoor air pollution (all cooking fuels)	+
	Smoking prevalence (reproductive age-standardized)	+
2	Antenatal care (4 visits) coverage (proportion)	-
	In-facility delivery (proportion)	-
	Live births 35+ (proportion)	+
	Skilled birth attendance (proportion)	-

	Health system access (capped)	-
	Healthcare access and quality index	-
	Age-standardised underweight (weight-for-age) SEV	+
3	Education (years per capita)	-
	Lag distributed income per capita (I$)	-
	Total fertility rate	+
	Socio-demographic Index	-

References

1. Anker M, Black RE, Coldham C, *et al.* A Standard verbal autopsy method for investigating causes of death in infants and children. Geneva, Switzerland: World Health Organization Department of Communicable Disease Surveillance and Response; The Johns Hopkins School of Hygiene and Public Health; The London School of Hygiene and Tropical Medicine, 1999.
2. Kalter HD, Gray RH, Black RE, Gultiano SA. Validation of postmortem interviews to ascertain selected causes of death in children. *Int J Epidemiol* 1990; 19: 380–6.
3. Quigley MA, Armstrong Schellenberg JR, Snow RW. Algorithms for verbal autopsies: a validation study in Kenyan children. *Bull World Health Organ* 1996; 74: 147–54.
4. Snow RW, Armstrong JR, Forster D, *et al.* Childhood deaths in Africa: uses and limitations of verbal autopsies. *Lancet* 1992; 340: 351–5.

B.2. Lower respiratory infections

The steps in the estimation of lower respiratory infections deaths are shown in the following flowchart:

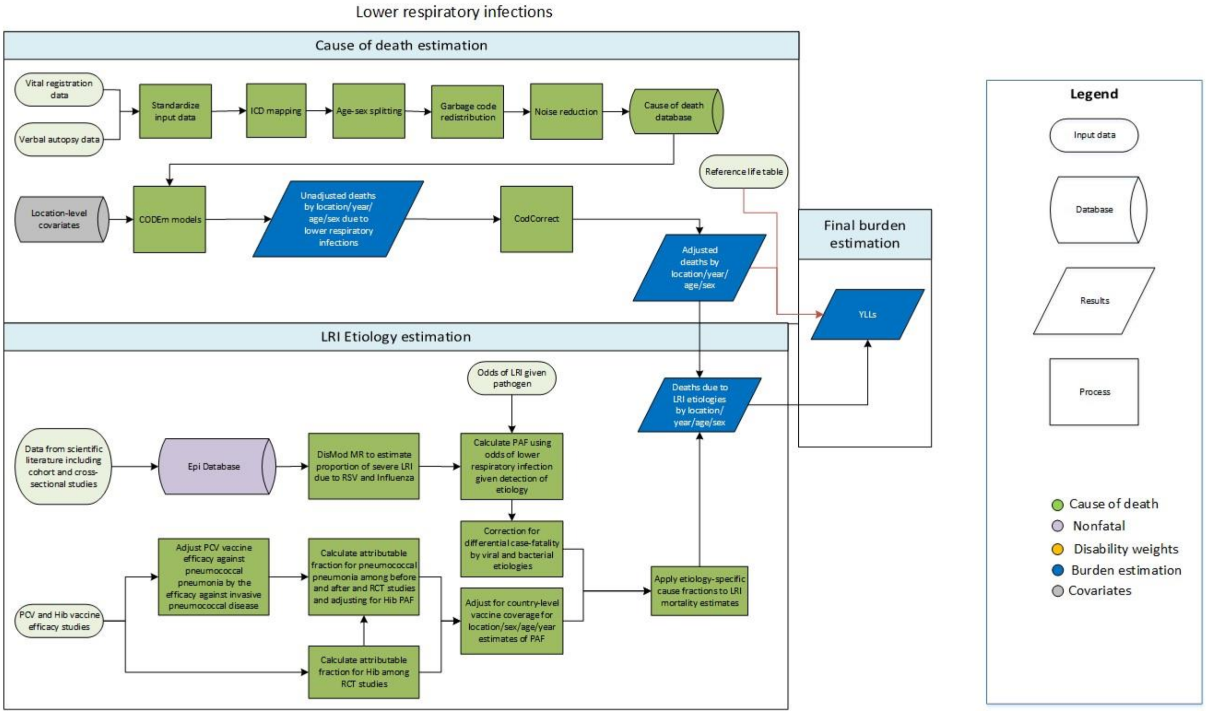

Data

Lower respiratory infection (LRI) mortality was estimated in CODEm. LRI mortality was estimated separately for males and females and for children under-5 years and older than 5 years. All available data from VR systems, surveillance systems, and verbal autopsy were used. The outliers were checked for and excluded from the data by country or region.

Modelling strategy

The country-level covariates were used to inform CODEm models (Table 1). The LRI cause of death models were evaluated using in and out of sample predictive performance. Like all models of mortality in GBD, LRI mortality models are single-cause, requiring in effect that the sum of all mortality models must be equal to the all-cause mortality envelope. The LRI mortality estimates, and other causes of mortality were corrected, by rescaling them according to the uncertainty around the cause-specific mortality rate. This process is called CoDCorrect and is essential to ensure internal consistency among causes of death.

Table 1. Covariates used in LRI mortality modelling for children under-5

Level	Covariate	Direction
1	Childhood stunting SEV	+
	Childhood underweight SEV	+
	Childhood wasted SEV	+
	Indoor air pollution	+
	Short gestation SEV	+
	Low weight gestation	+
	LRI summary exposure variable	+
	Second-hand smoking prevalence	+
	Antibiotics for LRI	-
	Hib vaccine coverage	-
	Pneumococcal conjugate vaccine coverage	-
2	Discontinued breastfeeding SEV	+
	Vitamin A deficiency	+
	Zinc deficiency	+
	DTP3 vaccine coverage	-
	Healthcare access and quality index	-
3	Outdoor air pollution ($PM_{2.5}$)	+
	Population density > $1000/km^2$	+
	Sanitation SEV	+
	Handwashing	-
	LDI per capita	-
	Maternal education	-
	Socio-demographic Index	-

The Level is the associated strength of relationship between the covariate and LRI mortality, ranked from 1 (proximally related) to 3 (distally related). The direction is the forced direction of the association between the covariate and LRI mortality.

1) $$HibPAF_{Base} = 1 - \frac{VE_{Pneumonia}}{VE_{Hib}}$$

2) $$PneumoPAF_{Base} = 1 - \frac{VE_{Pneumonia} * (1 - PAF_{Hib} * VE_{Hib\ Optimal})}{VE_{Streptococcus} * Cov_{Serotype}}$$

3) $$PAF_{Hib} = PAF_{Base} * \frac{(1 - Cov_{Hib} * VE_{Hib\ Optimal})}{(1 - PAF_{Base} * Cov_{Hib} * VE_{Hib\ Optimal})}$$

4) $$PAF_{Pneumo} = \frac{PAF_{Base} * (1 - Cov_{PCV} * VE_{PCV\ Optimal})}{(1 - PAF_{Hib} * Cov_{Hib} * VE_{Hib\ Optimal}) * \left(1 - \frac{PAF_{Base} Cov_{PCV} * VE_{PCV\ Optimal}}{(1 - PAF_{Hib} * Cov_{Hib} * VE_{Hib\ Optimal})}\right)}$$

B.3. Diarrhoeal diseases

The steps in the estimation of diarrhoeal diseases deaths are shown in the following flowchart:

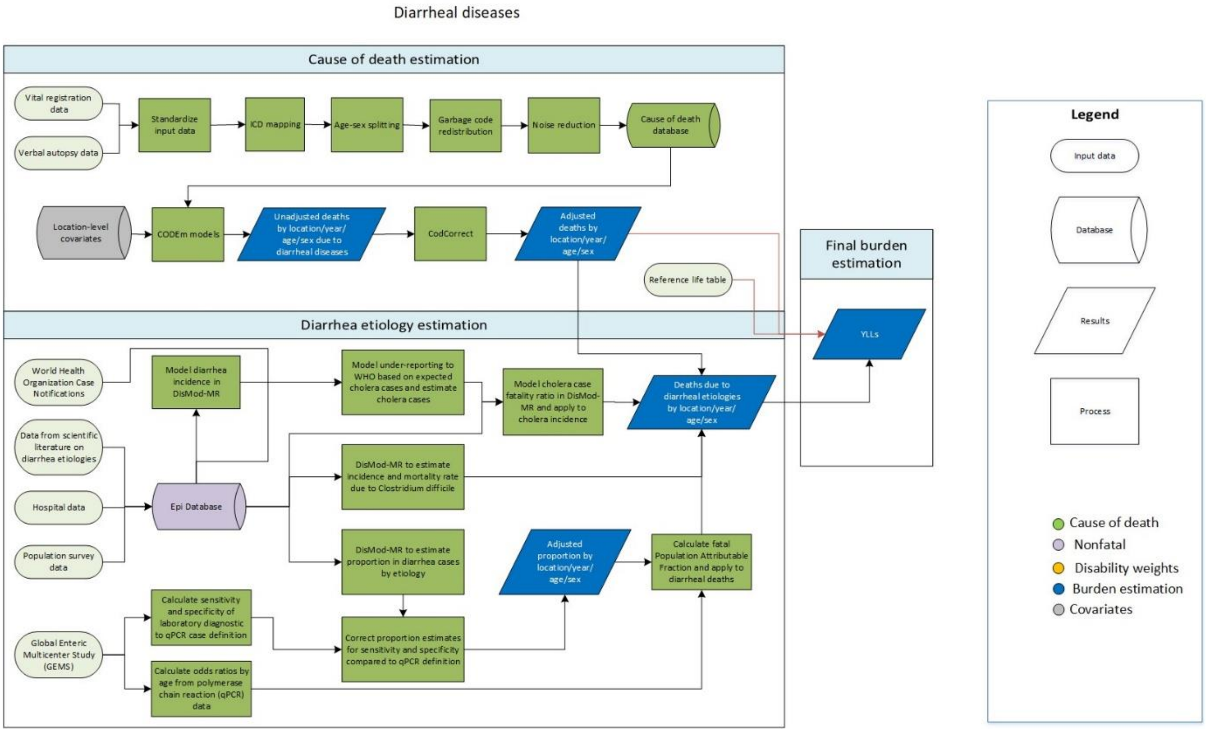

Data

All available data from VR systems, surveillance systems and verbal autopsies were used.

Modelling strategy

Diarrhoeal disease mortality was estimated in the CODEm. Diarrhoea mortality separately for males and females and for children under-5 years and older than 5 years were estimated. Country-level covariates were used to inform CODEm models (Table 1). The diarrhoeal disease cause of death models were evaluated using in and out of sample predictive performance.

Table 1. The covariates used in diarrhoea mortality modelling for the 0-4 years.

Level	Covariate	Direction
1	Diarrhoea SEV	+
	Childhood stunting SEV	+
	Sanitation SEV	+
	Water SEV	+
	Childhood underweight SEV	+
	Childhood wasting SEV	+
	Short gestation SEV	+
	Low weight gestation SEV	+
	Oral rehydration solution treatment	-
	Safe sanitation	-
	Safe water	-
2	Vitamin A deficiency	+
	Zinc deficiency	+
	Healthcare access and quality index	-
	Rotavirus vaccine	-
	Zinc treatment for diarrhoea	-
3	Breastfeeding SEV	+
	Handwashing	-
	LDI per capita	-
	Maternal education years	-
	Socio-demographic Index	-

	Population density < 150/km2	0
	Population density > 1000/km2	0

The level represents the strength of the association between the covariate and diarrhoea mortality from 1 (proximally related) to 3 (distally related). The direction indicates the positive or negative association between the covariate and diarrhoea mortality.

$$PAF = Proportion * (1 - \frac{1}{OR})$$

$$Odds\ ratio = exp(log(OR) - 1)) + 1$$

$$Proportion_{True} = \frac{(Proportion_{Observed} + Specificity - 1)}{(Sensitivity + Specificity - 1)}$$

B.4. Measles

The steps in the estimation of measles deaths are shown in the following flowchart:

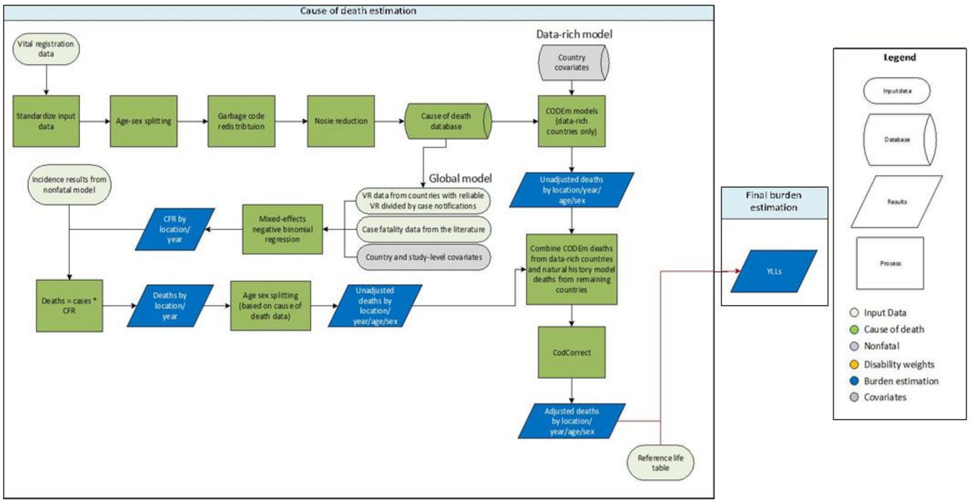

Modelling strategy

Two separate methods were used for modelling measles mortality based on the quality of available VR data. For countries with well-defined VR (ie, “data-rich”countries), a CODEm approach was used. For the remaining countries, a natural history model approach was used. For all countries, the age range was estimated for postneonatal to 59 years.

Data-rich countries

For data-rich countries, a CODEm strategy in count space was used to model VR data through time using the following country covariates:

Level	Covariate	Direction
1	Measles-containing vaccination dose one (MCV1)	-
2	Healthcare access and quality (HAQ) index	-
3	Health systems access (capped)	-
	Socio-demographic Index (SDI)	-
	Mean years of education per capita	-

Covariates including measles-containing vaccination dose two (MCV2) coverage were excluded due to their collinearity with MCV1. Models in count space (as opposed to rate space) had lower out-of-sample RMSE and were thus chosen as final models for these countries.

Natural history model

To inform the natural history model, data from the following sources were used: World Health Organization (WHO) case notifications from 1995 to 2017 (most recently released in June 2018); additional case notification sources identified by collaborators; VR data in countries with well-defined VR data; and case fatality data identified through systematic literature reviews. Studies were included in the literature review if they reported case fatality rate, number of deaths, and number of cases. Studies were excluded if they were conducted on non-representative samples. Measles mortality in the non-data-rich countries was modelled using a natural-history-based model.

First, measles incidence was modelled with a mixed-effects linear regression of case notifications from WHO (1995–2017) on both doses of routine measles vaccination rates (MCV1 and MCV2) and supplementary immunization activity (SIA) coverage using the following equation:

$$Y_{ij} = \beta_0 + \beta_1 MCV1_{ij} + \beta_2 MCV2_{ij} + \beta_{a3} SIA_{aij} + u_j + e_{ij} ,$$

Where Yij is the log-transformed incidence rate (in cases per 100,000 persons using WHO case notifications and GBD populations); $\beta 0$ is the fixed-effect intercept; $\beta 1$, $\beta 2$, and $\beta a3$ are the fixed-effects slopes on the log-transformed proportion of population without the MCV1 vaccine, log-transformed proportion of population without the MCV2 vaccine, and supplementary vaccination coverage (administered doses over the target population of all under-15s) lagged by a=1-5 years, respectively; uj is the super-region, region, and country-level random effects; eij is the residual; i is the year; and j is the location.

The results of this mixed effects regression model were then used to predict location-year specific incidence as a function of routine vaccine coverage and SIAs. To correct for underreporting in case notifications, the effect of a 95% attack rate was added, which was assumed to be the same across all unvaccinated populations. Uncertainty was estimated by taking 1,000 iterations of the predictions based on the variance-covariance matrix. More information on this part of the natural history model can be found in the non-fatal methods appendix for this round of the GBD.

Second, measles case fatality ratio was modelled using a mixed effects negative binomial regression using the Socio-demographic Index (SDI) as a country covariate and three indicators (hospital-based or not; outbreak or not; and rural or urban/mixed) as study-level covariates, with country random effects:

$$Y_{ij} = \beta_0 + \beta_1 SDI_{ij} + \beta_2 hospital_{ij} + \beta_3 outbreak_{ij} + \beta_3 rural_{ij} + u_j + e_{ij} ,$$

Where Yij is the number of deaths (using measles cases as the offset term); $\beta 0$ is the fixed-effect intercept; $\beta 1$, $\beta 2$, $\beta 3$, and $\beta 4$ are the fixed-effects slopes on the Socio-demographic Index (SDI) and hospital, outbreak, and rurality study-level covariates; uj is country-level random effects; eij is the residual; i is the year; and j is the location. Uncertainty was estimated by taking 1,000 iterations of the predictions based on the variance-covariance matrix and uncertainty in country random effects. The fit of the model was evaluated using diagnostic plots of predicted versus observed values.

Finally, estimated deaths were calculated at the 1000-draw level from the two sets of custom model predictions as:

$$deaths = incident\ cases * CFR .$$

The overall number of deaths were estimated and then an age-sex distribution was assigned based on the global level age- and sex-specific patterns found in the cause of death data.

B.5. Tetanus

The steps in the estimation of tetanus deaths are shown in the following flowchart:

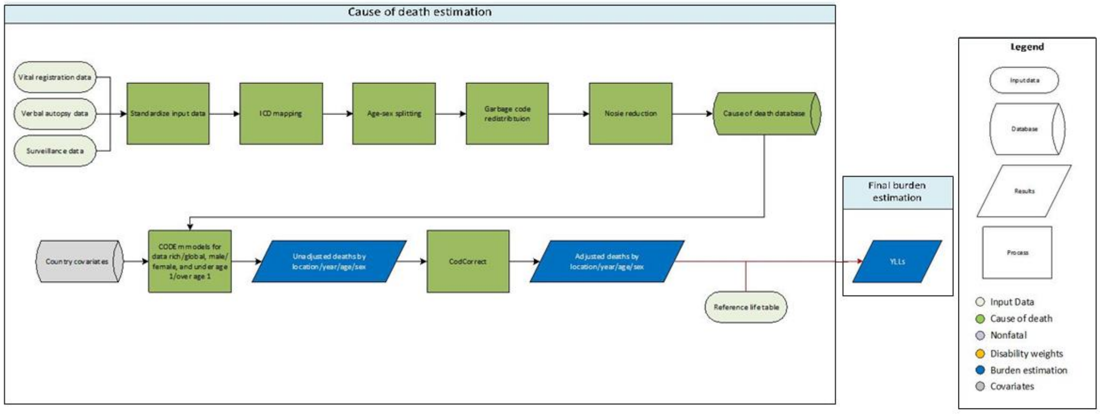

Data

Mortality data from VR, verbal autopsy, and surveillance sources were used in tetanus cause of death models. Data were excluded if they largely conflicted with the majority of data from other studies conducted either in the same or different countries in the same region with similar socio-demographic characteristics.

Modelling strategy

A count-space CODEm strategy was used. Separate models were run by age (under-1 year and 1-95+ years of age), sex (male and female), and data quality (data-rich and global). The following covariates for the under-1 models were used:

Level	Covariate	Direction
1	Diphtheria-tetanus-pertussis third-dose vaccination coverage (DTP3)	-
	Tetanus toxoid coverage	-
2	In-facility deliveries (proportion)	-
	Skilled birth attendance (proportion	-
	Health systems access (capped)	-
	Healthcare access and quality index (HAQ)	-
3	Lagged-distributed income (LDI)	-
	Socio-demographic Index (SDI)	-
	Mean years of education per capita	-

and the following covariates for the 1+ models:

Level	Covariate	Direction
1	Diphtheria-tetanus-pertussis third-dose vaccination coverage (DTP3)	-
2	Health systems access (capped)	-
	Healthcare access and quality index (HAQ)	-
3	Sanitation (proportion)	-
	Lagged-distributed income (LDI)	-
	Socio-demographic Index (SDI)	-
	Mean years of education per capita	-

Models in count space had lower out-of-sample RMSE than rate-space models and were thus chosen as final models for these data-rich countries.

B.6. Congenital birth defects

The steps in the estimation of congenital birth defects deaths are shown in the following flowchart:

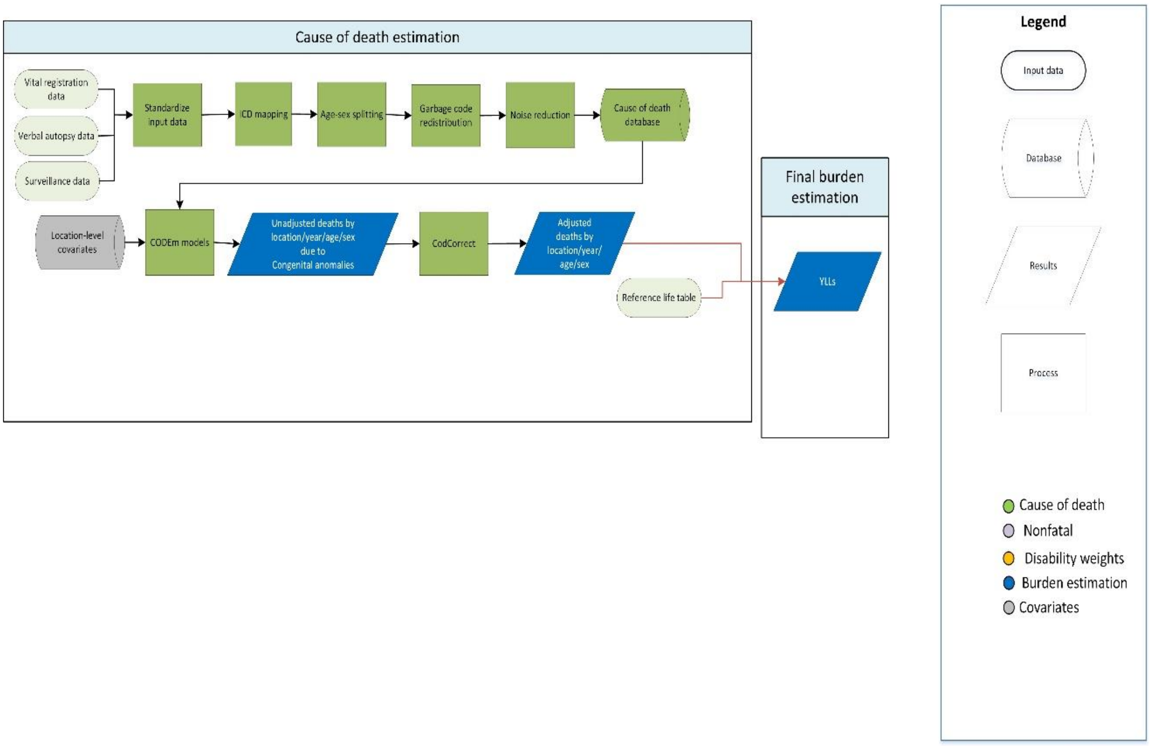

Congenital birth defects consist of neural tube defects, congenital heart anomalies, orofacial clefts, Down syndrome, Turner syndrome, Klinefelter syndrome, other chromosomal disorders, congenital musculoskeletal anomalies, urogenital congenital anomalies, digestive congenital anomalies, and other congenital birth defects.

Data

Input data for estimating mortality due to congenital anomalies was centrally extracted, processed, and stored in the CoD database. VR was the dominant data type, followed by VA and surveillance. Those CoD data sources that specified the sub-cause of birth defect were included in estimation of both the parent congenital anomalies model as well as in sub-type-specific models.

The majority of VA data were outliered in those over 5 years old as the age patterns were unreliable and led to poor model performance in the under-5 age groups. Some data sources from the parent model that had only a subset of sub-causes were specified and excluded (eg, congenital heart disease, neural tube defects, and other congenital anomalies) The sum of the sub-causes that clearly represented systematic underreporting of one of the sub-causes were also excluded.

Systematic underreporting was suspected when sex- and age-specific rates were more than an order of magnitude lower than neighboring or comparable locations. Data sources for those locations were still included by default for sub-cause-specific models because under-reporting of the total was not assumed to necessarily be associated with under-reporting of all of the component conditions.

Modelling strategy

All types of congenital anomalies were estimated using CODEm for GBD 2017, as was done for previous iterations of the GBD study. Specific causes included neural tube defects, congenital heart anomalies, orofacial clefts, Down syndrome, other chromosomal anomalies, congenital musculoskeletal anomalies, urogenital congenital anomalies, digestive congenital anomalies, and other congenital birth defects. No mortality was assumed from either Klinefelter syndrome or Turner syndrome, for which only non-fatal

outcomes were modelled. For GBD 2017, congenital anomalies were modelled as a cause of death for ages 0-69 years only, assuming that all mortality from congenital conditions occurs before age 70 years of age.

The following covarites were selected for CODEm model of overall congenital birth defects.

Covariate	Transformation	Level	Direction
Maternal alcohol consumption during pregnancy (proportion)	None	1	Positive
In-facility delivery (proportion)	None	1	Negative
Live births 35+ (proportion)	None	1	Positive
Folic acid unadjusted (ug)	None	1	Negative
Legality of abortion	None	2	Negative
Antenatal care (1 visit) coverage (proportion)	None	2	Not specified
Smoking prevalence (reproductive-age-standardised)	None	2	Positive
Antenatal care (4 visits) coverage (proportion)	None	2	Negative
Healthcare access and quality index	None	2	Negative
Education (years per capita)	None	2	Negative
Alcohol (litres per capita)	None	3	Positive
Fruits unadjusted (g)	None	3	Positive
Outdoor air pollution ($PM_{2.5}$)	None	3	Positive
Indoor air pollution (all cooking fuels)	None	3	Positive
Socio-demographic Index	None	3	Negative
Vegetables unadjusted (g)	None	3	Positive

B.7. Injuries

The steps in the estimation of injuries deaths are shown in the following flowchart:

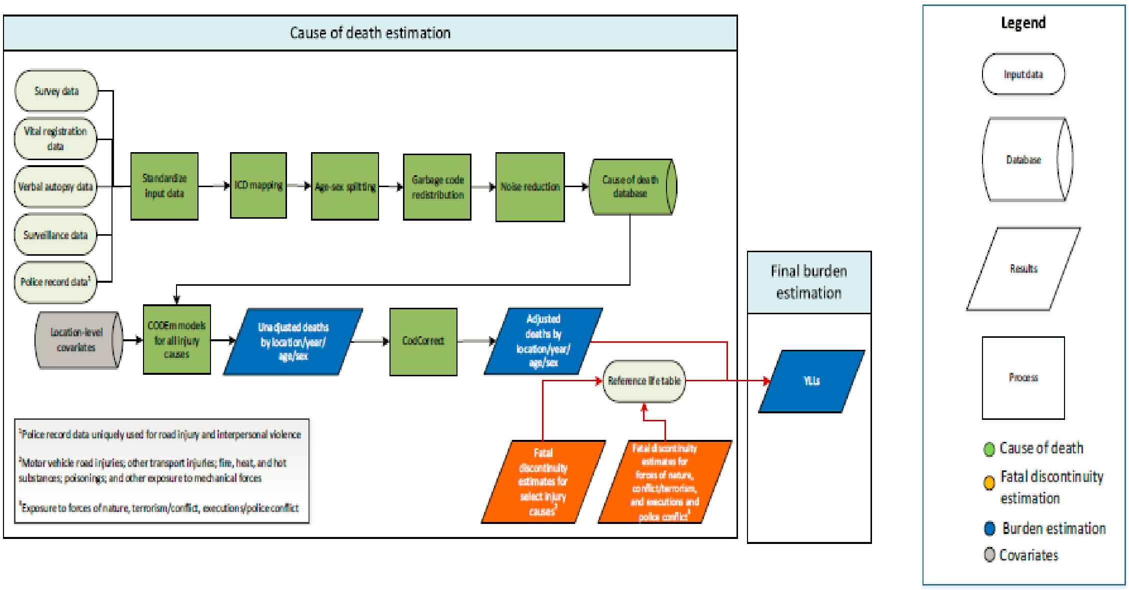

Data

In GBD 2017, injury mortality was estimated from VR, verbal autopsy, mortality surveillance, censuses, surveys, and police record data. Police and crime reports were data sources uniquely used for the estimation of deaths from road traffic injury and interpersonal violence. The police data were collected from published studies, national agencies, and institutional surveys such as the United Nations Crime Trends Survey and the WHO Global Status Report on Road Safety Survey.

For countries with VR data, police records were not used, except if the recorded number of road injury and interpersonal violence deaths from police records exceeded that in the VR. Infrequently, data points were marked as outliers. Outlier criteria excluded data points that [1] were implausibly high or low relative to global or regional patterns,[2] substantially conflicted with established age or temporal patterns, or [3] significantly conflicted with other data sources conducted from the same locations or locations with similar characteristics (ie, Socio-demographic Index).

Modelling strategy

Fatal discontinuity was estimated for ten injury causes modelled in CODEm. These causes included “Road injuries”, “Motor vehicle road injuries”, “Other transport injuries”, “Fire, heat, and hot substances”, “Poisonings” “Environmental exposure to heat and cold “, “Other unintentional injuries”, “Interpersonal violence”, “Other exposure to mechanical forces”, and “Executions and police conflict”. Final fatal discontinuity estimations for these causes were merged with CODEm results post-CoDCorrect to produce final cause of death results.

Preparation of data

The preparation of cause of death data includes age splitting, age-sex splitting, smoothing, and outlier detection. These steps are described in detail by Naghavi and colleagues and Lozano and colleagues.[1,2,3] The concept of “garbage codes” and redistribution of these codes was proposed in GBD 1990.[4] Garbage codes are causes of death that should not be identified as specific underlying causes of death but have been entered as the underlying cause of death on death certificates. A classic example of these types of codes in injuries chapters are “Exposure to unspecified factor” (X59 in ICD-10 and E887 in ICD-9) and all undetermined intent codes (Y10-Y34 in ICD-10 and E980-E988 in ICD 9). Other examples of garbage codes in injuries are the coding of an injury death to intermediate codes like septicaemia or peritonitis or as an ill-defined and unknown cause of mortality (R99). Approximately 2% of total deaths in countries with VR data are assigned to these three injury garbage code categories.

Splitting into sublevel causes

In countries with non-detail ICD code data, cause-of-injury categories were proportionally split into sublevel cause-of-injury categories. The sublevel cause-of-injury causes were created in the CoDCorrect process. One of the countries with non-detail ICD code data is South Africa, and in GBD 2013 the proportions of sublevel cause-of-injury were based on vital registration data. For GBD iterations of 2015, 2016, and 2017, the proportions were based on post-mortem investigation of injury deaths that are described elsewhere.[5]

Limitations and model assumptions

The police data for road injuries and interpersonal violence were added to help predict level and age patterns in countries with sparse or absent cause of death data even though it is known that from countries with near complete VR data, the police records tend to underestimate the true level of deaths. However, police data estimates were applied in instances where reported deaths were higher than VR numbers.

References

1. Lozano R, Naghavi M, Foreman K, *et al.* Global and regional mortality from 235 causes of death for 20 age groups in 1990 and 2010: a systematic analysis for the Global Burden of Disease Study 2010. *Lancet* 2012; 380: 2095–128.
2. Global, regional, and national age–sex specific all-cause and cause-specific mortality for 240 causes of death, 1990–2013: a systematic analysis for the Global Burden of Disease Study 2013. *Lancet* 2015; 385: 117–71.
3. Global, regional, and national life expectancy, all-cause mortality, and cause-specific mortality for 249 causes of death, 1980-2015: a systematic analysis for the Global Burden of Disease Study 2015. *Lancet* 2016; 388: 1459-1544.
4. Murray CJL, Lopez AD, Harvard School of Public Health, World Health Organization, World Bank. The global burden of disease: a comprehensive assessment of mortality and disability from diseases, injuries, and risk factors in 1990 and projected to 2020. Cambridge, MA: Published by the Harvard School of

Public Health on behalf of the World Health Organization and the World Bank: Distributed by Harvard University Press, 1996.
5. Matzopoulos R, Prinsloo M, Wyk VP, Gwebushe N, Mathews S, et al. Injury-related mortality in South Africa: a retrospective descriptive study of postmortem investigations. *Bull World Health Organ* 2015; 93: 303–13.

3. Risk factors estimation methods

The materials presented here are adapted from the following source:

- GBD 2017 Risk Factors Collaborators. Global, regional, and national comparative risk assessment of 84 behavioural, environmental and occupational, and metabolic risks or clusters of risks for 195 countries and territories, 1990–2017: a systematic analysis for the Global Burden of Disease Study 2017. *Lancet* 2018; 392: 1923–94.
- India State-Level Disease Burden Initiative Malnutrition Collaborators. The burden of child and maternal malnutrition and trends in its indicators in the states of India: the Global Burden of Disease Study 1990–2017. *Lancet Child & Adolescent Health* 2019; 3:855–70.
- India State-Level Disease Burden Initiative Air Pollution Collaborators. The impact of air pollution on deaths, disease burden, and life expectancy across the states of India: the Global Burden of Disease Study 2017. *Lancet Planetary Health* 2019; 3: e26-39.

The analytical approach used in GBD 2017 for comparative risk assessment to estimate population attributable fractions for risk factors is shown in the following flowchart:

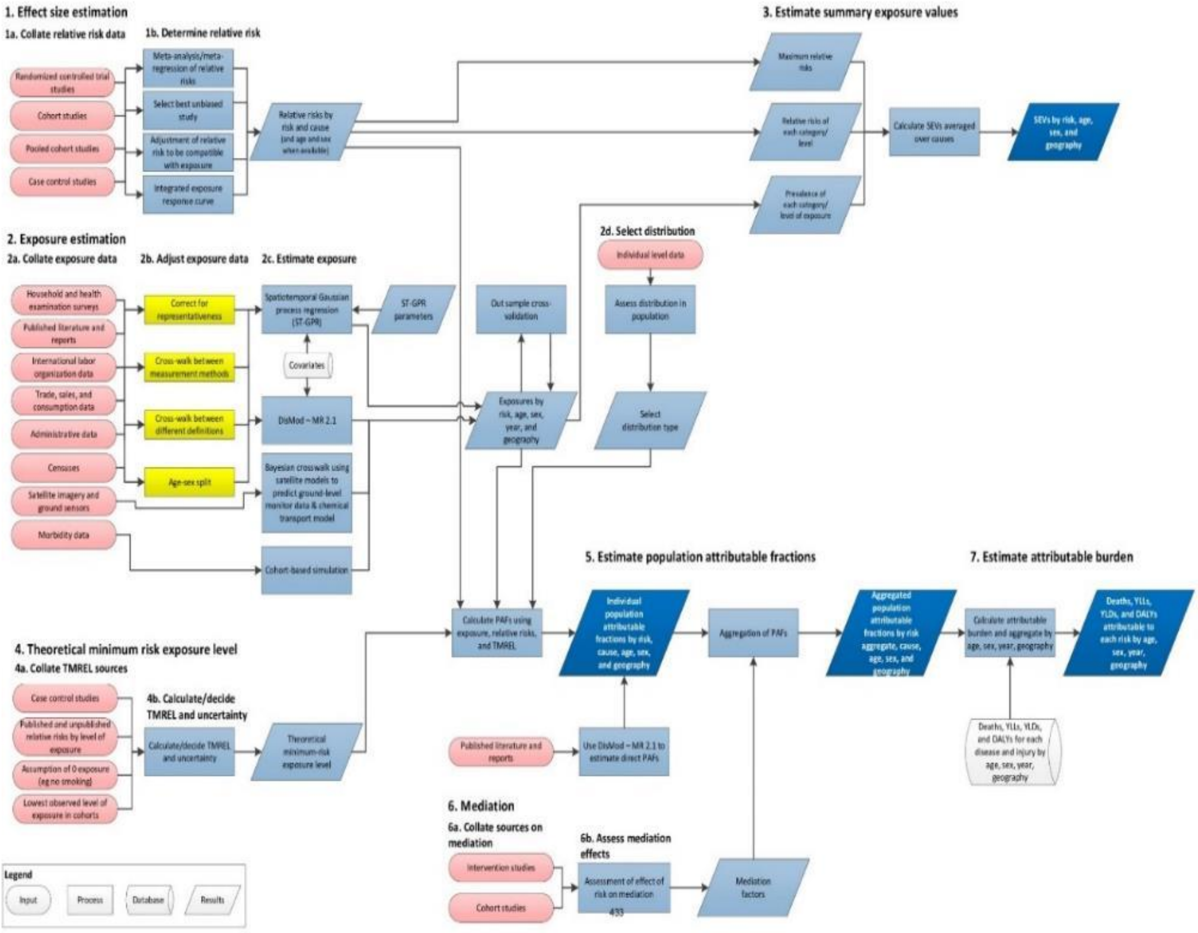

GBD is Global Burden of Disease. SEV is summary exposure value. TMREL is theoretical minimum-risk exposure level. PAF is population attributable fraction. YLL is years of life lost. YLD is years lived with disability. DALY is disability adjusted life-years. Ovals represent data inputs, rectangular boxes represent analytical steps, cylinders represent databases, and parallelograms represent intermediate and final results.

The details of the major risk factor related to under-5 deaths and neonatal deaths, i.e. child and maternal malnutrition, water, sanitation, and handwashing (WaSH), air pollution and second-hand smoke are described below. Description of other risk factors can be found in the GBD 2017 risk factor paper (Lancet 2018; 392: 1923-45).

A. Child and maternal malnutrition

The components of child and maternal malnutrition in GBD include low birth weight and short gestation, child growth failure, suboptimal breastfeeding, and micronutrient deficiencies. Low birth weight and short gestation includes low birth weight for gestation and short gestation for birth weight, child growth failure includes child stunting, child wasting, and child underweight, suboptimal breastfeeding includes non-exclusive breastfeeding and discontinued breastfeeding, and micronutrient deficiencies include iron, vitamin A, and zinc deficiencies. We describe in detail the methods used to estimate the two malnutrition indicators that are reported in this paper, i.e. low birth weight and short gestation and child growth failure.

A.1. Low birth weight and short gestation

Low birth weight for gestation and short gestation for birth weight are separate risk factors, however the exposures and relative risks for both were estimated jointly through the low birth weight and short gestation parent risk factor. The meaning of “low birth weight” and “short gestation” in GBD have subtle definitional differences compared to other usages of “low birth weight” and “short gestation” in the literature. The term “low birth weight” has historically been used to refer to birth weight less than 2,500 grams. However, because the goal of the GBD risk factors analysis was to quantify the entirety of attributable burden due to each risk factor, the GBD definition of “low birth weight” therefore refers to all birth weight below the theoretical minimum-risk exposure level (TMREL) for birth weight. Likewise, new-borns were typically classified into gestational age categories of “extremely preterm” (<28 weeks of gestation), “very preterm” (28-<32 weeks of gestation), and “moderate to late preterm” (32-<37 weeks of gestation). “Short gestation” refers to gestational age below the gestational age TMREL. Exposures and relative risks for the GBD low birth weight and short gestation risk factors were categorised into different combinations of joint 500-gram birth weight and 2-week gestational age. The lowest risk overall 500-gram/2-week bin was the overall TMREL. The univariate TMRELs vary with gestational age and birth weight. The lowest risk gestational age varies by birth weight category and the lowest risk birth weight vary with gestational age category. The latter were used to quantify univariate attributable risk. Under this framework, all attributable burden under the joint TMREL were referred to jointly as burden of low birth weight and short gestation. All attributable burden to birth weights under the TMREL for each gestational age category were, on aggregate, “low birth weight”, and all attributable burden to gestational ages under the TMREL for each birth weight category were, on aggregate, “short gestation.” Each combination of 500-grams and 2-weeks was associated with a relative risk for mortality by neonatal period (early and late neonatal) and by the causes, and relative to the joint TMREL.

The steps in the estimation of low birth weight and short gestation are shown in the following flowchart:

Low birth weight and Short gestation Risk Factors

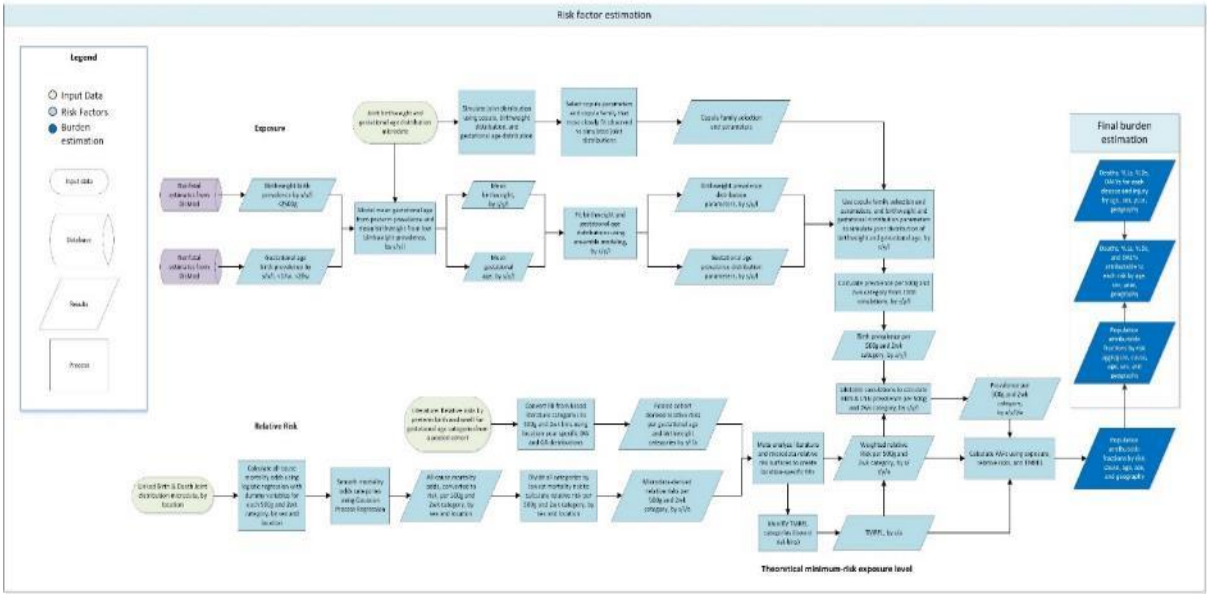

Data

To model the joint distribution of exposure of low birth weight and short gestation for each location, year, and sex estimated in GBD 2017, three types of information were used:

- Distribution of birth weight for each location, year, and sex
- Distribution of gestational age for each location, year, and sex
- Copula family and parameters, specifying correlation between gestational age and birth weight distributions

Major data inputs for India were national surveys such as National Family Health Surveys, District Level Household Surveys, and Annual Health Surveys.

Modelling strategy

To model the joint distribution of birth weight and gestational age for every location-year-sex, ensemble model methods standard to GBD risk factors, were used to create separate distributions of birth weight and gestational age for every location-year-sex. Microdata is the most ideal data source for modelling distributions; however, microdata is not widely available for birth weight and is even scarcer for gestational age. Much more readily available, and from a wider range of locations and years, is categorical prevalence data for low birth weight (<2,500 grams), extremely preterm (<28 weeks of gestation), very preterm (28-32 weeks of gestation), moderate to late preterm (32-37 weeks of gestation), and preterm birth (<37 weeks of gestation). The full distributions at birth were modelled for gestational age and birth weight for all GBD locations, estimation years, and both sexes. The gestational age and birth weight distributions were then aggregated into the categorical estimates of <28 weeks, 28-<32 weeks, 32-<37 weeks gestation, and <2,500 grams birth weight.

Ensemble model methods standard to GBD were used to model the distribution at birth of gestational age and birth weight. Gestational age ensemble distribution models used the prevalence of <37 weeks gestation, the prevalence of <28 weeks gestation, and mean gestational age per each location-year-sex as inputs into the model. Birth weight distribution models used the prevalence of <2,500 grams birth weight and mean birth weight for each location-year-sex. Prevalence of <37 weeks gestation and of <2,500 grams birth weight was estimated for all location-year-sex using spatiotemporal regression and Gaussian Process Regression (ST-GPR) modelling process standard to GBD.

Low birth weight (<2,500 grams) data were extracted from surveys, VR systems, and the literature. The missing data for low birth weight in the Demographic and Health Surveys (DHS) was imputed using Amelia package in R from the following variables: urbanicity, sex, birthweight recorded on card, birth order, maternal education, paternal education, child age, child weight, child height, mother's age at birth, mother's weight, shared toilet facility, and household water treated.

Global ensemble weights for gestational age were derived by using a 3 million sample of all available microdata to select the ensemble weights. Of the exponential, gamma, inverse gamma, Weibull, log normal, and normal distributions, the three distribution families that received the highest weights were the Weibull (87%), normal (4%), and inverse gamma (4%) distributions. Global ensemble weights for birth weight were derived using a 3 million sample of all available microdata, in addition to birth weight microdata available primarily through the DHS and Multiple Indicator Cluster Surveys (MICS). Of the exponential, gamma, inverse gamma, Weibull, log normal, and normal distributions, the three distribution families that received the highest weights were the log normal (38%), normal (32%), and Weibull (20%) distributions.

Ordinary-least square was used to model mean gestational age for all location-year-sex by regressing mean gestational age on prevalence of <37 weeks gestation per location-year. All available microdata were used to fit the model. Ordinary-least square was also used to model mean birth weight by regressing prevalence of <2,500 grams birth weight per location-year. All available joint microdata, as well as additional birth weight microdata extracted primarily through DHS and MICS, were used to fit the model. As estimates of prevalence of <37 weeks gestation and prevalence of <2,500 grams birth weight was available for all location-year-sex through ST-GPR models, mean gestational age and mean birth weight were predicted for all location-year-sex.

Copula optimisation

Copula modelling was used to model joint distributions between the birth weight and gestational age marginal distributions. In order to model the joint distribution of birth weight and gestational age from separate distributions, information is needed about the correlation between the two distributions. The Spearman correlation for each country where joint microdata was available, pooling across all years of data available, ranged from 0.25-0.49 indicating that the distributions of birth weight and gestational age are not independent. The overall Spearman correlation was 0.38, pooling across all countries in the dataset. The Copula and VineCopula packages in R were used to select the optimal copula family and copula parameters to model the joint distribution, using joint microdata from the country-year. The copula family selected from the microdata was "Survival BB8", with theta parameter set to 1.75 and delta parameter set to 1.

The joint distribution of birth weight and gestational age per location-year-sex was modelled using the global copula family and parameters selected, and the location-year-sex gestational age and birth weight distributions. The joint distribution was simulated 100 times to capture uncertainty. Each simulation consisted of 100,000 simulated joint birth weight and gestational age data points. Each joint distribution was divided into 500-gram by 2-week bins to match the categorical bins of the relative risk surface. Birth prevalence was then calculated for each 500-gram by 2-week bin.

Estimating early neonatal prevalence and late neonatal prevalence from birth cohorts

Early neonatal and late neonatal prevalence was estimated using life table approaches for each 500-gram and 2-week bin. Using the all-cause early neonatal mortality rate for each location-year-sex, births per location-year-sex-bin, and the relative risks for each location-year-sex bin in the early neonatal period, the all-cause early neonatal mortality rate was calculated for each location-year-sex bin. The early neonatal mortality rate per bin was used to calculate the number of survivors at 7 days and prevalence in the early neonatal period. Using the same process, the all-cause late neonatal mortality rate for each location-year-sex was paired with the number of survivors at 7 days and late neonatal relative risks per bin to calculate late neonatal prevalence and survivors at 28 days.

Relative risks and theoretical minimum-risk exposure level

The available data for deriving relative risk was only for all-cause mortality. The relative risk of all-cause mortality across all available sources and selected outcomes was analysed based on criteria of biologic plausibility. Some causes, most notably congenital birth defects, haemoglobinopathies, malaria, and HIV/AIDS, were excluded based on the criteria that reverse causality could not be excluded. The outcomes included in calculating the attributable burden for low birth weight and short gestation were: diarrhoeal diseases, lower respiratory infections, upper respiratory infections, otitis media, pneumococcal meningitis, H influenza type B meningitis, meningococcal meningitis, other meningitis, encephalitis, neonatal preterm birth complications, neonatal encephalopathy due to birth asphyxia and trauma, neonatal sepsis and other neonatal infections, hemolytic disease and other neonatal jaundice, other neonatal disorders, and sudden infant death syndrome.

GBD has so far attributed disease burden to low birth weight and short gestation only for the neonatal period. Further analytical work is planned to able to use the available evidence to estimate disease burden that may be attributable in the post-neonatal period as well.

For each location, data were pooled across years, and the risk of all-cause mortality at the early neonatal period and late neonatal period at joint birth weight and gestational age combinations was calculated. To calculate relative risk at each 500-gram and 2-week combination, logistic regression was first used to calculate mortality odds for each joint 2-week gestational age and 500-gram birth weight category. Mortality odds were smoothed with Gaussian process regression, with the independent distributions of mortality odds by birth weight and mortality odds by gestational age serving as priors in the regression.

A pooled country analysis[1] of mortality risk in the early neonatal period and late neonatal period by short gestational age category in developing countries including Asian countries were also converted into 500-gram and 2-week bin mortality odds surfaces. Location-specific relative risk surfaces, derived from location-specific estimates of with-condition mortality of preterm birth, were converted into 500-gram and 2-week bin mortality odds. The meta-analysed mortality odds surface for each location was smoothed using Gaussian Process Regression and then converted into mortality risk. To calculate mortality relative risks, the risk of each joint 2-week gestational age and 500-gram birth weight category were divided by the risk of mortality in the joint gestational age and birth weight category with the lowest mortality risk.

For each of the country-derived relative risk surfaces, the 500-gram and 2-week gestational age joint bin with the lowest risk was identified. This bin differed within each country dataset. To identify the universal 500-gram and 2-week gestational age category that would serve as the universal TMREL for our analysis, we chose the bins that was identified to be the TMREL in each country dataset to contribute to the universal TMREL. Therefore, the joint categories that served as our universal TMREL for the low birth weight and short gestation risk factor were "38-40 weeks of gestation and 3,500-4,000 grams", "38-40 weeks of gestation and 4,000-4,500 grams", and "40-42 weeks of gestation and 4,000-4,500 grams". As the joint TMREL, all three categories were assigned to a relative risk equal to 1.

Population attributable fraction calculations

The total population attributable fraction (PAF) for the low birth weight and short gestation joint risk factor was calculated by summing the PAF calculated from each 500-gram and 2-week category, with the lowest risk category among all the 500-gram and 2-week categories serving as the TMREL. The equation for calculating PAF for each 500-gram and 2-week category is:

$$PAF_{joasgt} = \frac{\sum_{x=1}^{u} RR_{joast}(x)P_{jasgt}(x) - RR_{joasg}(TMRE_{jas})}{\sum_{x=1}^{u} RR_{joas}(x)P_{jasgt}(x)}$$

To calculate the overall PAF for the short gestation for birth weight risk factor, PAF was once again calculated for each joint 500-gram and 2-week category. Unlike the joint PAF calculation, which used only one TMREL for all 500-gram and 2-week categories, the joint 500-gram and 2-week category with the lowest risk for each 500-gram birth weight grouping served as the

TMREL for that 500-gram birth weight grouping. In the relative risk surface figures, a birth weight grouping was one "column" of the birth weight and gestational age matrix.

The overall PAF for the short gestation for birth weight risk factor was then calculated for all the joint 500-gram and 2-week categories using the formula below:

$$PAF_{1..i} = 1 - \prod_{i=1}^{n}(1 - PAF_i)$$

The same methodology was applied to calculate the total PAF for the low birth weight for gestation risk factor, using 2-week gestational age categories (each "row" of the matrix) instead of 500-gram birth weight categories.

After the short gestation for birth weight PAF and low birth weight for gestational age PAF were calculated, they were then scaled so that the sum of the short gestation for birth weight PAF and low birth weight for gestation PAF equal the low birth weight and short gestation parent PAF calculated for each location-year-sex-age group.

A.2. Child growth failure

Child growth failure was estimated using three indicators stunting, wasting, and underweight all of which are based on categorical definitions using the WHO 2006 growth standards for children 0-59 months. Definitions were based on z-scores from the growth standards, which were derived from an international reference population. Mild, moderate, and severe categorical prevalences were estimated for each of the three indicators.

TMREL for stunting, wasting, and underweight was assigned to be greater than or equal to -1 standard deviation (SD) of the WHO 2006 standard height-for-age, weight-for-height, and weight-for-age curves, respectively.

The steps in the estimation of child growth failure are shown in the following flowchart:

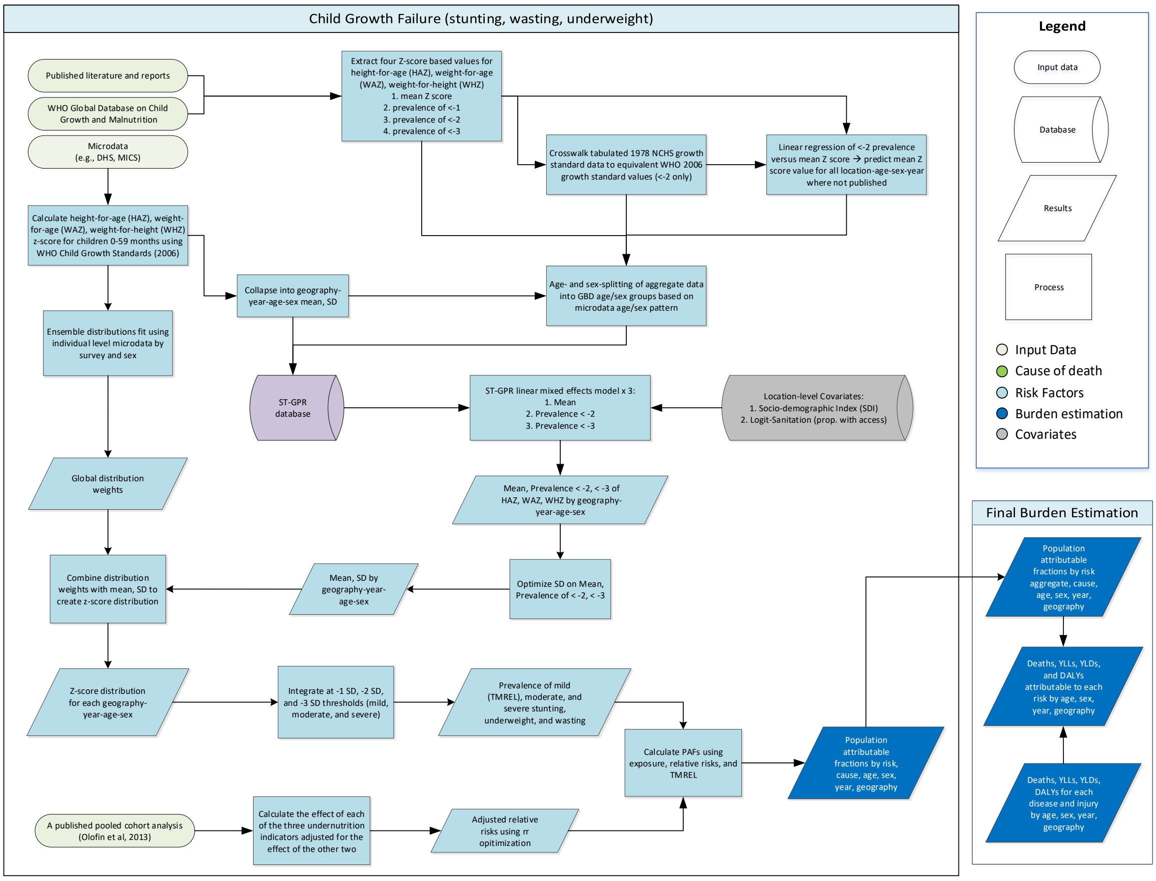

Data

The three main inputs for the GBD child growth failure models were: microdata from population-based surveys including anthropometric surveys, tabulated data from reports and published literature, and the WHO Global Database on Child Growth and Malnutrition.[2] Population surveys include a variety of multi-country and country-specific survey series such as DHS, MICS, and Living Standards Measurement Surveys, as well as other one-time country-specific surveys. This microdata contains information about each individual child's age (from which age in weeks and age in months are calculated), as well as height and/or weight. From that information, a height-for-age z-score (HAZ), weight-for-height z-score (WHZ), and weight-for-age z-score (WAZ) were calculated using the WHO 2006 Child Growth Standards and the LMS method.[3]

All available data from the WHO Global Database on Child Growth and Malnutrition were extracted – majority of which was from published studies. Four metrics that were sought from all sources with tabulated data were: mean z-score, prevalence <-1 z-score (mild), prevalence <-2 z-score (moderate), and prevalence <-3 z-score (severe). All data for each metric was extracted for each of stunting (height-for-age z-score; HAZ), wasting (weight-for-height z-score; WHZ), and underweight (weight-for-age z-score; WAZ).

To maximise internal-consistency and comprehensiveness of the modelling dataset, three data transformations were performed. Firstly, any data that were reported using the National Center for Health Statistics (NCHS) 1978 growth standards were crosswalked to corresponding values on the WHO 2006 Growth Standards curves based on a study that evaluated growth standard concordance.[4] Crosswalks from 1978 to 2006 growth standards were performed only on <-2 z-score (i.e. moderate) prevalence data as that was where the concordance was most consistent. Secondly, for any study that lacked a measure of mean z-score for any of stunting, wasting, or underweight, a mean value was predicted for that study based on an ordinary-least squares regression of mean z-score versus <-2 z-score prevalence for that metric from all sources where both were available. Thirdly, any data that were presented as both sexes combined for 0-59 months, the age- and sex-pattern were used from all data sources that included that detail to split these data into corresponding age-and sex-specific data.

The major data sources from India were national surveys including National Family Health Surveys, District Level Household Surveys, Annual Health Survey, National Nutrition Monitoring Bureau Diet and Nutritional Surveys, and Rapid Survey on Children.

Modelling strategy

The following three-step modelling process was used to estimate stunting, wasting, and underweight.

First, all microdata was fit using an ensemble modelling process. A series of 12 individual distributions (normal, log normal, log logistic, exponential, gamma, mirror gamma, inverse gamma, gumbel, mirror gumbel, Weibull, inverse Weibull, and beta) were fit to the entire set of microdata (approximately 2.5 million individual z-scores) at the individual survey level. A weighting algorithm combined each distribution to find the optimal combination of these distributions for each survey, minimising the absolute prediction error across the entire distribution. Ensemble weights for each survey were then averaged across all surveys to produce a single set of global weights of the ensemble distributions. Weights were different for each sex, but invariant across geography, time, and age group. All component distributions that were used to derive weights were parameterised using "method of moments," meaning that each corresponding probability density function could be described as a function of the mean and variance of the quantity of interest.

Second, models were developed for mean z-scores and prevalence of moderate and severe growth failure. Individual level microdata were collapsed to calculate three metrics: mean z-score, moderate prevalence, and severe prevalence. These data were combined with that derived from literature, GHDx review, and the WHO Global Database on Child Growth and Malnutrition. For those sources where moderate prevalence was reported without a corresponding mean, a predicted mean was calculated using an ordinary-least square regression from those sources where both

metrics were present. Each of the three metrics were then modelled using ST-GPR, generating estimates for each location, year, age group, and sex.

Third, estimates of mean and prevalence (moderate and severe) were combined with ensemble weights in an optimisation framework in order to derive the variance that would best correspond to the predicted mean and prevalence. This variance was then paired with the mean, and using the method of moments equation for each of the component distributions of the ensemble, probability density function of the distribution of z-scores were calculated for each location, year, age group, and sex. Probability density functions were integrated to determine the prevalence between -1 and -2 z-scores (mild), between -2 and -3 z-scores (moderate), and below -3 z-scores (severe). These were categorical exposures used for subsequent attributable risk analysis.

All models were run with the complete dataset. Data plausibility inspection began with examination of time-trends in stunting. If a given datum was judged to have led to a change in the prevalence of moderate stunting in 1-4 year olds of 50% or greater in 5 years or fewer, and was inconsistent with data prior to and after that year (a change considered implausible), the offending datum were outliered and the model was re-run. The results of moderate stunting, wasting, and underweight were further visually-inspected in parallel to look for location-year-age-sex where the results were not internally-consistent (e.g. underweight rapidly increasing, and stunting and wasting decreasing). This inspection revealed very few inconsistent data.

Relative risks were derived from a pooled cohort analysis.[5] The final list of outcomes paired with child growth failure risks included lower respiratory infections, diarrhoea, measles, and protein energy malnutrition as shown in the below table.

There is a high degree of correlation between stunting, wasting, and underweight. Failing to account for their covariance and assuming independence would significantly overestimate the total burden. A method developed by Olofin and colleagues was used to adjust observed relative risks by simulating the joint distribution of the three indicators using the distribution of each indicator and covariance between indicators in the countries included in the meta-analysis (extracted from DHS micro-data).[6] Based on the analysis done by McDonald and colleagues, it was assumed that there is an interaction between the three indicators, and the interaction terms were extracted from the corresponding analysis. The adjusted relative risks were calculated by minimising the error between observed crude relative risks (from meta-analysis) and expected crude relative risks derived from adjusted relative risks.

The relative risks were adjusted using an optimisation algorithm that takes into account covariance between the three child growth failure indicators. Of historical note, upper respiratory infections and otitis media were previously included as outcomes, based on the “analogy” causal criterion, assuming there is similar pathway as lower respiratory infections outcome. However, closer review did not find sufficient evidence to support their inclusion and they were excluded. 100% of protein energy malnutrition were attributed to childhood wasting and underweight but not stunting. The final list of relative risk outcomes paired with child growth failure risks included lower respiratory infections, diarrhoea, measles, and protein energy malnutrition as shown in the below table.

Adjusted RRs for each risk-outcome pair for child growth failure

Outcome	**Stunting**	**Wasting**	**Underweight**
Diarrhoea	<-1: 1.111 (1.023-1.273)<br><-2: 1.222 (1.067-1.5)<br><-3: 1.851 (1.28-2.699)	<-1: 6.601 (2.158-11.243)<br><-2: 23.261 (9.02-35.845)<br><-3: 105.759 (42.198-157.813)	<-1: 1.088 (1.046-1.134)<br><-2: 1.23 (1.163-1.314)<br><-3: 2.332 (2.076-2.802)
Lower respiratory infections	<-1: 1.125 (0.998-1.655)<br><-2: 1.318 (1.014-2.165)<br><-3: 2.355 (1.15-5.114)	<-1: 5.941 (1.972-11.992)<br><-2: 20.455 (70.84-37.929)<br><-3: 47.67 (15.923-94.874)	<-1: 1.145 (1.044-1.364)<br><-2: 1.365 (1.215-1.755)<br><-3: 2.593 (1.908-4.39)
Measles	<-1: 1.103 (0.861-1.719)<br><-2: 1.540 (1.029-3.222)<br><-3: 2.487 (1.129-6.528)	<-1: 1.833 (0.569-8.965)<br><-2: 8.477 (1.33-42.777)<br><-3: 37.936 (5.088-199.126)	<-1: 0.995 (0.5-1.726)<br><-2: 2.458 (1.26-5.118)<br><-3: 5.668 (1.767-12.414)
Protein-energy malnutrition	0% PAF	100% PAF	100% PAF

<-1, <-2, and <-3 refer to categories of stunting, wasting, and underweight that are less than -1, -2, and -3 standard deviations of the median in the WHO 2006 standard curve.

B. Water, sanitation, and handwashing

The components of water, sanitation, and handwashing (WaSH) in GBD include unsafe water, unsafe sanitation, unsafe hygiene. The methods used to estimate the two WaSH components that are reported in this paper, i.e. unsafe water and unsafe sanitation are decribed here in detail.

B.1. Unsafe water

The steps in the estimation of unsafe water are shown in the following flowchart:

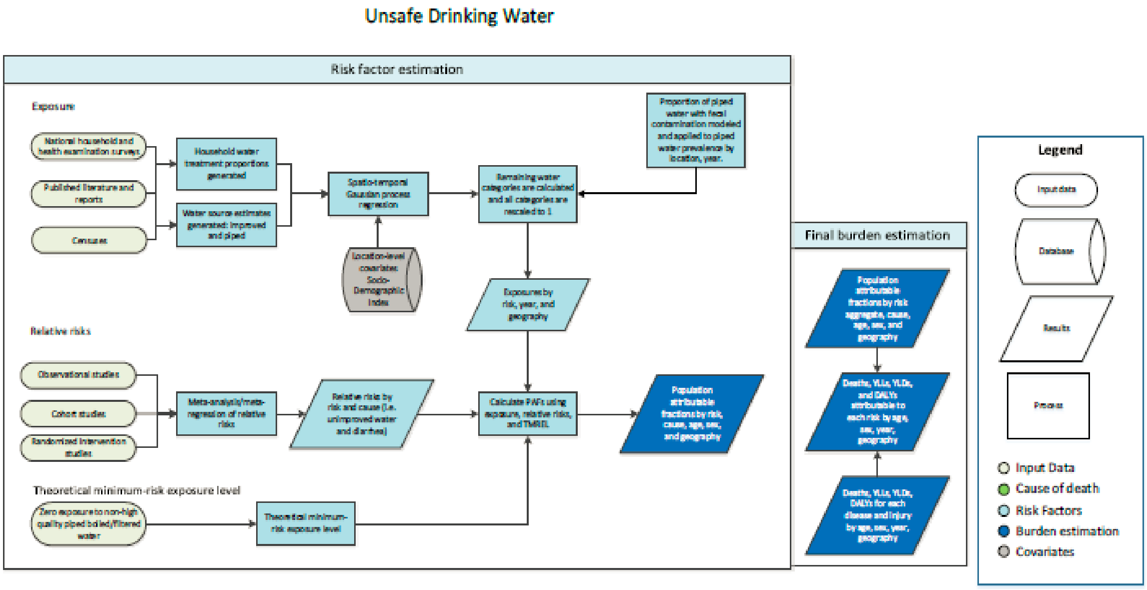

For GBD 2017, exposure to unsafe water was defined based on reported primary water source used by the household and use of household water treatment (HWT) to improve the quality of drinking water before consumption. Water sources were defined as "improved" based on the JMP designation,[1] which includes piped water as improved water, and households with access to piped water connection to the house, yard, or plot were defined as having access to piped water supply. Solar treatment, chlorine treatment, boiling, or the use of filters were all established as effective point-of-use household water treatments based on effect sizes calculated from network meta-analysis.

Data

There are many data sources on unsafe water in India including national health surveys such as the National Family Health Survey, the District Level Household Survey, Annual Health Survey, and nationwide surveys of the National Sample Survey Organisation as well as other published epidemiological studies. For each survey, household sample weights were multiplied by the number of household members to produce a weighting scheme that estimates proportion of individuals, not proportion of households, exposed to a given indicator. Survey data were then tabulated to the two water source and two water treatment categories of interest for each location.

Modelling strategy

Water source data is modelled using an ordinal framework, with two distinct models: prevalence of piped water and proportion of improved water (excluding piped) within the non-piped population. Both models produce results for each unique location, year combination. This ordinal framework allows us to estimate the category with the most data (piped water prevalence) and leverage that estimate to anchor the estimates for improved and unimproved water categories. The results of the improved proportion model are multiplied by the piped water prevalence to calculate improved water prevalence. The sum of improved and piped water prevalence are subtracted from 1 to yield unimproved water prevalence. HWT categories are estimated in a similar ordinal framework, by modelling prevalence of individuals using no water treatment methods and proportions of households that boil/filter water within the population of

households that engage in treatment methods. The prevalence of individuals that boil/filter drinking water is calculated by multiplying the proportion that boil/filter modelled previously times prevalence of any water treatment (estimated by subtracting prevalence of no treatment from 1).

The prevalence of individuals that treat their water using solar/chlorine methods was estimated by subtracting the sum of prevalence of no treatment estimates and prevalence of filter/boil treatment from 1. By year and location, each of the above categories are modelled using a 3-step modelling scheme of mixed effect linear regression followed by spatio-temporal Gaussian process regression (ST-GPR), which produces full time series estimates for each GBD 2017 location. Socio-demographic index (SDI), a composite metric combining education per capita, income per capita, and fertility, was set as a fixed effect in the linear regression since it proved to be a significant predictor. Random effects were set at GBD 2017 region and super-region levels to fit the models but were not used in the predictions.

The process of vetting and validating models was accomplished primarily through an examination of ST-GPR scatter plots by GBD 2017 location from 1990-2017. Any unfitting data points were re-inspected for error at the level of extraction and survey implementation, and subsequently excluded from analysis if deemed appropriate. In addition to SDI, a number of different potential fixed effects were considered, including lag-distributed income and urbanicity, but SDI proved to be the strongest predictor of the unsafe water categories. Uncertainty in the estimates was initially formed based on standard deviation by survey, then propagated through ST-GPR modelling by means of confidence intervals around each data point that reflect the point-estimate specific variance.

Once models are vetted, full time series outputs from ST-GPR modelling are then converted from proportion to prevalence by year and geography and then rescaled to form 9 mutually exclusive categories that sum up to 1. The table below provides the final result of this rescaling.

**Category**	**Definition**
Unimproved, no HWT	Proportion of individuals that primarily use unimproved source, and *do not* use any HWT to purify their drinking water.
Unimproved, chlorine/solar	Proportion of individuals that primarily use unimproved source, and solar or chlorine treatment to purify their drinking water.
Unimproved, boil/filter	Proportion of individuals that primarily use unimproved source, and boil or filter to purify their drinking water.
Improved water except piped, no HWT	Proportion of individuals that primarily use improved sources other than piped water supply, and *do not* use any HWT to purify their drinking water.
Improved water except piped, chlorine/solar	Proportion of individuals that primarily use improved sources other than piped water supply, and use solar or chlorine treatment to purify their drinking water.
Improved water except piped, boil/filter	Proportion of individuals that primarily use improved sources other than piped water supply, and boil/filter their drinking water.
Basic piped water, no HWT	Proportion of individuals that primarily use basic piped water supply, and *do not* use any HWT to purify their drinking water
Basic piped water, chlorine/solar	Proportion of individuals that primarily use basic piped water supply, and *use* solar or chlorine water treatment to purify their drinking water.
Basic piped water, boil/filter	Proportion of individuals that primarily use basic piped water supply, and boil or filter to purify their drinking water
High-quality (HQ) piped water, boil/filter	Proportion of individuals that primarily use basic piped water supply, and boil or filter to purify their drinking water

The microbiological quality of piped water sources were modelled primarily using data a review.[1] that measured proportion of piped water sources contaminated with fecal indicators. The value generated was used from this model to split the prevalence of piped water into basic piped water and high quality piped water by location, year, age, and sex.

A substantial limitation in our analysis is the paucity of data on HWT and piped water quality. The inclusion of more location-specific data on water treatment utilisation at the household level can greatly improve our estimates in future iterations.

Theoretical minimum-risk exposure level

The theoretical minimum-risk exposure level for unsafe water is defined as all households have access to high quality piped water that has been boiled or filtered before drinking.

Relative risks

For GBD 2017, unsafe water was paired with one outcome-diarrhoeal diseases-given evidence provided by relative risk studies. A meta-analysis[3] provided the bulk of the relative risk evidence for the relationship between unsafe water and diarrhoeal diseases. This meta-analysis was updated through a literature review that searched for related intervention studies post-2014 conducted in PubMed. Relative risk values for water-source interventions and point-of-use treatment interventions were calculated using network meta-analysis approach so as to include studies that differ in control groups within the same analysis. This analysis produced distinct relative risks for each water source and water treatment category. The combined effect of a source intervention and point-of-use intervention was assumed to be multiplicative in order to match GBD 2017 exposure definitions.

References

1. World Health Organization (WHO), United Nations Children's Fund (UNICEF). Progress on drinking water, sanitation and hygiene: 2017 update and SDG baselines. Geneva: WHO, UNICEF, 2017. https://washdata.org/report/jmp-2017-report-final.
2. Bain, R., Cronk, R., Wright, J., Yang, H., Slaymaker, T., & Bartram, J. Fecal contamination of drinking-water in low- and middle-income countries: a systematic review and meta-analysis. *PLoS Medicine* 2014, 11.
3. Wolf, Jennyfer, Annette Prüss-Ustün, Oliver Cumming, Jamie Bartram, et al. Systematic review: assessing the impact of drinking water and sanitation on diarrhoeal disease in low- and middle-income settings: systematic review and meta-regression. *Trop Med Int Health Tropical Medicine & International Health* 2014; 19.8: 928-42.

B.2. Unsafe sanitation

The steps in the estimation of unsafe sanitation are shown in the following flowchart:

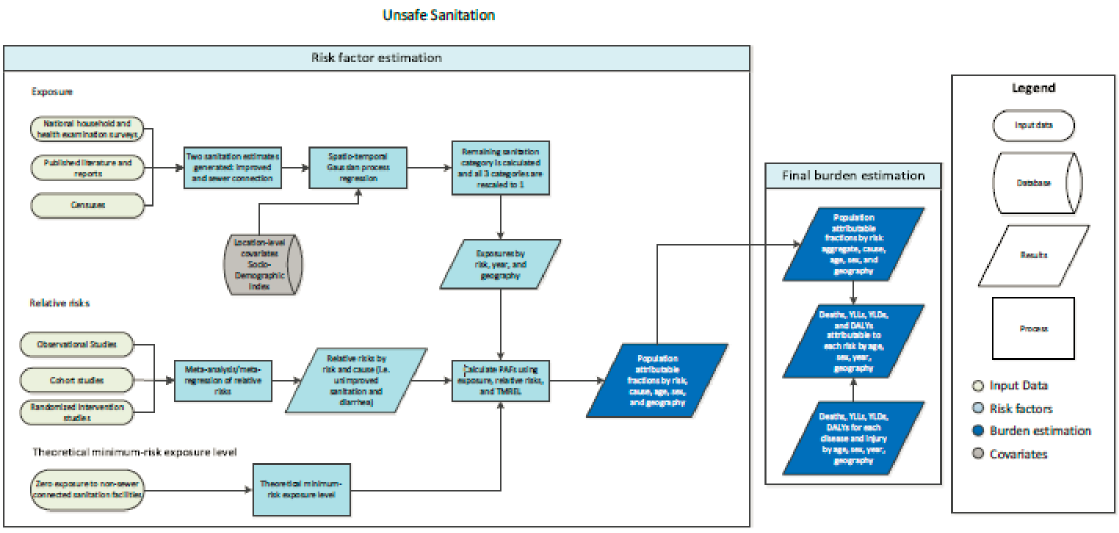

Exposure

Exposure to unsafe sanitation is defined based on the primary toilet type used by households. Improved facilities are defined as such based on JMP designation (WHO). Sewer connection toilets included flush toilets or any toilet with connection to the sewer or septic tank.

Data

There are many data sources on unsafe water in India including national health surveys such as the National Family Health Survey, the District Level Household Survey, Annual Health Survey, and nationwide surveys of the National Sample Survey Organisation as well as other published epidemiological studies. For each survey, household sample weights were multiplied by the number of

household members to produce a weighting scheme that estimates proportion of individuals, not proportion of households, exposed to a given indicator. Survey data were then tabulated to the two sanitation categories, sewer connection and improved sanitation for each location.

Modelling startegy

A change made for GBD 2017 was to model sanitation categories in an ordinal framework instead of independent models. Two distinct indicators were estimated: the prevalence of individuals using sewer connection or septic tank facilities and the proportion of individuals with improved sanitation within the population not connected to sewer or septic tank. This ordinal framework allows us to estimate the category with the most data (sewer connection/septic tank prevalence) and leverage that estimate to anchor the estimates for improved and unimproved sanitation categories. The results of the improved proportion model is multiplied by the sewer connection/septic tank prevalence to calculate improved sanitation prevalence. The sum of improved and sewer connection/septic tank prevalence are subtracted from 1 to yield unimproved sanitation prevalence.

The two indicators were modelled using a 3-step modelling scheme of mixed effect linear regression followed by ST-GPR, which produced full time series estimates for each GBD 2017 location. SDI, a composite metric combining education per capita, income per capita, and fertility, was set as a fixed effect in the linear regression since it proved to be a significant predictor. Random effects were set at GBD 2017 region and super region levels to fit the models but were not used in the predictions.

The process of vetting and validating models was accomplished primarily through an examination of ST-GPR scatter plots by GBD 2017 location from 1990-2017. Any unfitting data points were re-inspected for error at the level of extraction and survey implementation, and subsequently excluded from analysis if deemed appropriate. In addition to SDI, a number of different potential fixed effects were considered, including lag-distributed income and urbanicity, but SDI proved to be the strongest predictor of unsafe sanitation in terms of magnitude of the coefficient. Uncertainty in the estimates was initially constructed based on standard deviation around each survey mean, then propagated through ST-GPR modelling by incorporating the variance of each data point in the Gaussian process regression step. A data point with high variance, for example, would contribute relatively less influence to the model than a data point with lower variance.

Once models are vetted, full time series outputs from ST-GPR modeling are then converted from proportion to prevalence by year and geography and then rescaled to form three mutually exclusive categories that sum up to 1. The table below provides the final result of this rescaling.

Category	Definition
Unimproved sanitation	Proportion of individuals that use unimproved sanitation facilities.
Improved sanitation	Proportion of individuals with access to improved sanitation facilities, excluding sewer connection or septic tank.
Sanitation facilities with sewer connection or septic tank	Proportion of individuals with access to toilet facilities with sewer connection or septic tank.

Theoretical minimum-risk exposure level

The theoretical minimum-risk exposure level for unsafe sanitation was defined as all individuals have access to a sanitation facility with sewer connection.

Relative risks

For GBD 2017, unsafe sanitation was only paired with one outcome, diarrhoeal diseases. A meta-analysis by Wolf et al. 2014 provides the bulk of the relative risk evidence for the relationship between unsafe sanitation and diarrhoeal diseases. This meta-analysis was updated through a literature review that searched for related intervention studies post-2014 conducted in PubMed. Search terms used were identical to those provided by Wolf et al. 2014.

References

1. World Health Organization (WHO), United Nations Children's Fund (UNICEF). Progress on drinking water, sanitation and hygiene: 2017 update and SDG baselines. Geneva: WHO, UNICEF, 2017. https://washdata.org/report/jmp-2017-report-final.Wolf, Jennyfer, Annette Prüss-Ustün, Olive
2. Cumming, Jamie Bartram, et al. Systematic review: assessing the impact of drinking water and sanitation on diarrhoeal disease in low- and middle-income settings: systematic review and meta-regression. *Trop Med Int Health Tropical Medicine & International Health* 2014; 19.8: 928-42.

C. Air pollution

Air pollution in GBD consists mainly of ambient air pollution and household air pollution. The exposure to these and the disease burden caused by them are estimated separately in GBD.

C.1. Ambient particulate matter pollution

Exposure to ambient particulate matter pollution was defined as the population-weighted annual average mass concentration of particles with an aerodynamic diameter less than 2.5 micrometres ($PM_{2.5}$) in a cubic meter of air at a spatial resolution of 0.1°x0.1° over the globe, which is approximately 11x11-km at the equator. This measurement is reported in $\mu g/m^3$. These estimates were based on multiple satellite-based aerosol optical depth data globally combined with a chemical transport model, and calibration of these with $PM_{2.5}$ data from the ground-level monitoring stations.

For the purpose of attributing disease burden to ambient particulate matter pollution, the theoretical minimum-risk exposure level (TMREL) was defined as population-weighted mean between 2.4 and 5.9 $\mu g/m^3$, bounded by the minimum and fifth percentiles of exposure distributions from outdoor air pollution (OAP) cohort studies. This uniform distribution represents the uncertainty regarding adverse effects of low-level exposure. To include the uncertainty in the TMREL, we took a random draw from the uniform distribution of the interval between 2.4 and 5.9 µg/m3 each time the population attributable burden was calculated. TMREL was defined as a uniform distribution rather than a fixed value in order to represent the uncertainty regarding the level at which the scientific evidence was consistent with adverse effects of exposure. The specific OAP cohort studies selected for this averaging were based on the criteria that their fifth percentiles were less than that of the American Cancer Society Cancer Prevention II (CPSII) cohort's fifth percentile of 8.2 based on Turner et al.[1]

The steps in the estimation of disease burden attributable to ambient particulate matter pollution are shown in the following flowchart:

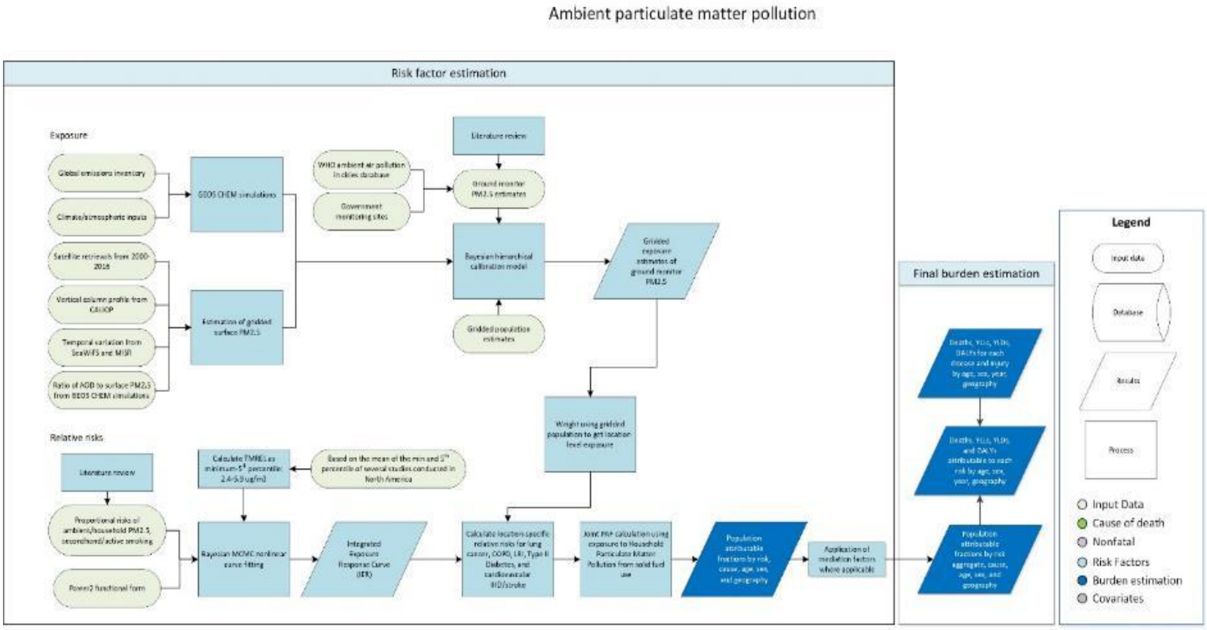

Data

The estimates of ambient $PM_{2.5}$ exposures in India were based on multiple satellite-based aerosol optical depth data combined with a chemical transport model, and calibration of these with $PM_{2.5}$ data from ground-level monitoring stations.

$PM_{2.5}$ ground measurements: More recent monitoring data from new locations were used in GBD 2017. Monitor-specific measurements (rather than city averages as reported in the WHO Air Pollution in Cities database) were used, resulting in measurements of concentrations of PM_{10} and $PM_{2.5}$ from approximately 10,000 ground monitors from 113 countries. For locations measuring only PM_{10}, $PM_{2.5}$ measurements were estimated from PM_{10}. This was performed using a locally derived conversion factor ($PM_{2.5}/PM_{10}$ ratio, for stations where measurements were available for the same year) that was estimated using population-weighted averages of location-specific conversion factors for the country or state. If country-level conversion factors were not available, the average of country-level conversion factors within a region were used. Additional information related to the ground measurements was also included where available, including monitor geo coordinates and monitor site type. Estimates in GBD 2017 included a substantially increased number of ground monitoring sites from India, which included data from 185 ground monitors for $PM_{2.5}$ and 184 monitors for PM_{10}.

Satellite-based estimates: These estimates were available at 0.1º×0.1º resolution (~11x11-km resolution at the equator) which combines aerosol optical depth retrievals from multiple satellites with the GEOS Chem chemical transport model and land use information.[2] The model to calibrate satellite-based estimates to these measurements varied smoothly over space and time in regions with many measurements.

Population data: A comprehensive set of population data on a high-resolution grid was obtained from the Gridded Population of the World (GPW v4r10) database. These data were provided on a 0.0417×0.0417º resolution. Aggregation to each 0.1×0.1º grid cell comprised of summing the central 3×3 population cells. As this resulted in a resolution higher than necessary, it was repeated four times, each offset by one cell in a North, South, East and West direction. The average of the resulting five quantities was used as the estimated population for each grid cell.

Chemical transport model simulations: Estimates of the sum of particulate sulphate, nitrate, ammonium and organic carbon and the compositional concentrations of mineral dust simulated using the GEOS Chem chemical transport model, and a measure combining elevation and the distance to the nearest urban land surface were available from 2000 to 2016 for each 0.1×0.1º grid cell.[2]

Modelling strategy

Annual mean exposure to $PM_{2.5}$ was estimated in 5-year intervals from 1990 onward, at 0·1×0·1° resolution using estimates from satellites combined with a chemical transport model, surface measurements, and geographical data. We aggregated gridded exposure concentrations to national-level population-weighted means using the corresponding grid cell population value. National-level population-weighted mean concentrations and the 95% uncertainty interval (95% UI) around this mean were estimated by sampling 1000 draws of each grid cell value and its uncertainty distribution.

An updated version of the Data Integration Model for Air Quality (DIMAQ) was used for ambient particulate matter pollution modelling.[2,3] The coefficients in the calibration model were estimated for each country or state. Where data were insufficient within a country or state, information was `borrowed' from a higher aggregation (region) and if enough information was still not available from an even higher level (super region). Individual country or state level estimates were therefore based on a combination of information from the state, country, its region and super-region. This was implemented within a Bayesian Hierarchical Modelling (BHM) framework. BHMs provide an extremely useful and flexible framework in which to model complex relationships and dependencies in data. Uncertainty can also be propagated through the model allowing uncertainty arising from different components, both data sources and models, to be incorporated within estimates of uncertainty associated with the final estimates. The results of the modelling comprise a posterior distribution for each grid cell, rather than just a single point estimate, allowing a variety of summaries to be calculated. The primary outputs here were the median and 95% credible intervals for each grid cell.

The GBD 2017 model (DIMAQ-2) was updated to also include within country variation in calibrations. The model used for GBD 2017, DIMAQ-2, provides a number of substantial improvements over the initial formulation of DIMAQ. In DIMAQ, ground measurements from different years were all assumed to have been made in the primary year of interest (i.e. 2014 for GBD 2015 before extrapolation) and then regressed against values from other inputs (e.g. satellites etc.) made in that year. In the presence of changes over time therefore, and particularly in areas where no recent measurements were available, there was the possibility of mismatches between the ground measurements and other variables. In DIMAQ-2, ground measurements were matched with other inputs over time, and the possibility of the global level coefficients being allowed to vary over time, subject to smoothing that was induced by a second-order random walk process. In addition, the manner in which spatial variation can be incorporated within the model was developed: where there was sufficient data, the calibration equations can now vary (smoothly) both within and between countries, achieved by allowing the coefficients to follow (smooth) Gaussian processes. Within a geographic location where there was insufficient data, in order to produce accurate equations, information was borrowed from lower down the hierarchy and was supplemented with information from the wider region.

Due to both the complexity of the models and the size of the data, notably the number of spatial predictions that were required, recently developed techniques that perform 'approximate' Bayesian inference based on Integrated Nested Laplace Approximations (INLA) were used.[4] Computation was performed using the R interface to the INLA computational engine. Fitting the models and performing predictions for each of the 1.4 million grid cells required the use of a high performance computing cluster making use of high memory nodes.

Model development and comparison was performed using within- and out-of-sample assessment. In the evaluation, cross validation was performed using 25 combinations of training (80%) and validation (20%) datasets. Validation sets were obtained by taking a stratified random sample, using sampling probabilities based on the cross-tabulation of $PM_{2.5}$ categories (0-24.9, 25-49.9, 50-74.9, 75-99.9, 100+ $\mu g/m^3$) and super-regions, resulting in them having the same distribution of $PM_{2.5}$ concentrations and super-regions as the overall set of sites. The following metrics were calculated for each training/evaluation set combination: for model fit - R^2 and deviance information criteria (DIC, a measure of model fit for Bayesian models); for predictive accuracy - RMSE and population weighted root mean squared error.

All modelling was performed on the log-scale. The choice of which variables were included in the model was made based on their contribution to model fit and predictive ability. The following is a list of variables and model structures that were considered in developing the model:

Variable	Model structure
Continuous explanatory variables	(SAT) Estimate of $PM_{2.5}$ (in µgm-3) for 2014 from satellite remote sensing on the log scale.
	(CTM) Estimate of $PM_{2.5}$ (in µgm-3) for 2010 from the TM5 chemical transport model on the log-scale.
	(POP) Estimate of population for 2014 on the log-scale.
	(SNAOC) Estimate of the sum of sulphate, nitrate, ammonium and organic carbon simulated using the GEOS Chem chemical transport model.
	(DST) Estimate of compositional concentrations of mineral dust simulated using the GEOS Chem chemical transport model.
	(EDxDU) The log of the elevation difference between the elevation at the ground measurement location and the mean elevation within the GEOS Chem simulation grid cell multiplied by the inverse distance to the nearest urban land surface.
Discrete explanatory variables	(LOC) Binary variable indicating whether exact location of ground measurement is known.
	(TYPE) Binary variable indicating whether exact type of ground monitor is known.
	(CONV) Binary variable indicating whether ground measurement is $PM_{2.5}$ or converted from PM_{10}.
Random effects	Grid cell random effects on the intercept to allow for multiple ground monitors in a grid cell.
	State-country-region-super-region hierarchical random effects for the intercept.
	State-country-region-super-region hierarchical random effects for the coefficient associated with SAT.
	State-country-region-super-region hierarchical random effects for the coefficient associated with the difference between estimates from CTM and SAT.
	State-country-region-super-region hierarchical random effects for the coefficient associated with POP.
	State/country level random effects for population uses a neighbourhood structure allowing specific borrowing of information from neighbouring geographies.

	Within a region, country or state level effects of SAT and the difference between SAT AND CTM were assumed to be independent and identically distributed.
	Within a super-region, region level random effects were assumed to be independent and identically distributed.
	Super-region random effects were assumed to be independent and identically distributed.
Interactions	Interactions between the binary variables and the effects of SAT and CTM.

The final model contained the following variables: SAT, POP, SNAOC, DST, EDxDU, LOC, TYPE, and CONV, together with interactions between SAT and each of LOC, TYPE and CONV. The model structure contained grid cell random effects on the intercept to allow for multiple ground monitors in a grid cell, state-country-region-super-region hierarchical random effects for intercepts and SAT and state/country level random effects for population using a neighbourhood structure allowing specific borrowing of information from neighbouring countries together with region-super-region hierarchical random effects for POP.

Satellite estimates, populations and quantities estimated using the GEOS-Chem model were available for 1990, 1995, 2000, 2005, 2010 to 2017. Population estimates for 2000, 2005, 2010, 2015 and 2020 were available from GPW version 4r10. For 1990 and 1995 data were extracted from GPW version 4r10.[3] As with populations for 2015, values for each cell for 2011 to 2017 were obtained by interpolation using natural splines with knots placed at 2000, 2005, 2010, 2015 and 2020.

These were used as inputs to DIMAQ, enabling estimates of exposures to be obtained for each of these years respectively. For 2017, estimates of exposures were obtained from predictions from locally-varying regression models.[5] For each cell a model was fit to the values within that cell over time, with a constraint placed on the rate of change between 2016 and 2017 to avoid unrealistic and/or unjustified extrapolation of trends. Measures of uncertainty were obtained by repeating the procedure for the limits of the 95% intervals, again on a cell-by-cell basis.

The burden attributable to $PM_{2.5}$ for ischaemic heart disease (IHD), stroke, lung cancer, chronic obstructive pulmonary disease (COPD), and acute lower respiratory infections (LRI) were estimated for all-ages. The GBD 2017 type 2 diabetes was added as a relative risk outcome. These were also the pollutant-outcome pairs used to estimate the ambient particulate matter pollution attributable burden. The results from all cohort studies published so far were used that reported cause-specific relative risk (RR) estimates based on measured or modelled $PM_{2.5}$ and that adjusted for potential confounding due to other major risk factors such as tobacco smoking using data for each study participant.

A recently published work assembled the evidence for the relationship between particulate matter and diabetes to generate integrated exposure-response function (IER) curves and attributable burden estimates based on methodologies similar to those of the GBD.[6] When generating the IER for type 2 diabetes, all eight of the studies summarized by Bowe et al. were included in addition to the other cohorts. The resulting attributable burden estimates were remarkably similar to GBD 2017 results.

Integrated exposure-response function

IERs were developed for each cause of death to estimate the RR of mortality over the entire global range of ambient annual mean $PM_{2.5}$ concentrations using risk estimates from studies of ambient particulate matter pollution, household air pollution (HAP), and second-hand smoke exposure and active smoking. IERs assign concentrations of $PM_{2.5}$ to each type of exposure on an equivalent µg/m³ basis assuming that risk was determined by the 24-h $PM_{2.5}$ inhaled dose regardless of the exposure source. The IER was created to ascertain the shape of the dose response curve for a variety of health outcomes across a wide range of exposure to $PM_{2.5}$. The IER model was fit by integrating RR information from studies of OAP, second hand tobacco smoke, HAP, and active smoking. Because OAP studies are often performed at the lower end of the OAP range, incorporating other exposures to particulate matter enables RR estimation across the global range of exposure. These methods have been described in detail elsewhere.[7,8] Notable changes for GBD 2017 included the added OAP cohorts, the inclusion of HAP cohorts, and updated literature reviews for active smoking studies.

All published and unpublished cohorts of long-term exposure to ambient $PM_{2.5}$ and incidence or mortality due to IHD, stroke, COPD, lung cancer, and LRIs were considered. Newly published cohorts of long-term exposure to ambient $PM_{2.5}$ and incidence or mortality due to IHD, stroke, COPD, lung cancer, and LRI were added. One notable addition was the China male cohort which included mortality due to

IHD, stroke, COPD, and lung cancer.[9] This study represented a higher exposure range than most of the previously incorporated studies with 5th and 95th percentile of 15.5 and 77.1 micrograms/m^3. In GBD 2017, type 2 diabetes which was included as a new relative risk outcome and was estimated by including all cohorts which measured long-term $PM_{2.5}$ exposure and diabetes incidence or mortality due to diabetes.

All the available cohort studies of HAP and any of the related measured outcomes were included, along with those with binary exposure data (presence or absence of solid-fuel use for cooking). To incorporate cohort studies with binary exposure data, the $PM_{2.5}$ mapping function to obtain a $PM_{2.5}$ level attributed to solid fuel use for cooking for the location-year of the study (ExpHAP) was used. The OAP exposure model was used to obtain an OAP $PM_{2.5}$ level for the location-year (ExpOAP). The study RR was used to inform the curve on the range of ExpOAP to (ExpOAP + ExpHAP).

Updated systematic reviews of literature for studies examining cigarettes smoked per day and the six IER outcomes related to particulate matter were used for the high exposure range of the curve.

The IER has the mathematical form:

$$IER(\beta,z)=1+\alpha\times(1-e^{-\beta(z-z_{cf})_{+}^{\gamma}})$$

where z is the level of $PM_{2.5}$ and z_{cf} is the TMREL, below which no additional risk is assumed, with

$$(z-z_{cf})_{+}=(z-z_{cf})$$

If z is greater than z_{cf} and zero otherwise. Here, $1 + \alpha$ is the maximum risk, β is the ratio of the IER at low to high concentrations, and γ is the power of $PM_{2.5}$ concentration. Epidemiological evidence suggests that the RRs for IHD and stroke decline with age. The particulate matter source-specific RR were modified for both IHD and stroke mortality and applied this age modification to the RRs, fitting the IER model for each age group separately. Observed RRs were related to the IER within a Bayesian framework using the STAN fitting algorithm. Given the true values of the four parameters (α, β, γ, z_{cf}), the logarithm of each study's were assumed that observed RR was normally distributed, with mean defined by the IER and variance given by the square of the observed SE of the study-specific log-relative risk estimate plus an additional variance term for each of the four sources on $PM_{2.5}$ exposure (OAP, second-hand smoke, HAP, and active smoking).

It is important to recognize the inherent limitations of the IER approach. The use of various sources to construct a risk curve assumes an equitoxicity of particles, consistent with evaluations by US EPA and WHO. However, current evidence suggests there are differences in health impact by source, size, and chemical composition. This is seen when comparing studies of ambient and household particulate matter. As this body of evidence grows, this strategy will be continually re-examined for the integrated exposure-response curve. For now, the IER is a practical solution to fill gaps in the literature where there were no sufficient evidence such as household air pollution exposures and ambient in highly polluted areas.

The exposure concentrations used for both second-hand smoking and active smoking data points when fitting the IER were contrasted with the TMREL and do not take into account ambient particulate matter pollution. In future iterations of fitting the curve, the alternate approaches will be tested, including a similar approach to HAP, allowing each data point to inform the curve on the range of ExpOAP to (ExpOAP + ExpAS/SHS).

Relative risk and proportional population-attributable fraction (PAF) approach

For GBD 2017, a new approach was developed to use the IER for obtaining PAFs for both OAP and HAP. Previously relative risks for both exposures were obtained from the IER as a function of exposure and relative to the same TMREL. Were you to reduce one of these risk factors, however, the other would remain. In GBD 2017, RRs were estimated from the output of the IER curve. Everyone is exposed to some level of OAP, but only a proportion of the population in each location-year use solid cooking fuel and were exposed to HAP. For the proportion of the population not exposed to HAP the RR was obtained using RROAP = IER (z = ExpOAP), and the PAF was calculated at each grid-cell. These PAFs were population weighted and aggregated up to each location.

For the proportion of the population exposed to both OAP and HAP, a joint RR from the IER by RROAP+HAP = IER (z = ExpOAP+ExpHAP) were calculated. This joint RR was used to calculate a joint PAF for each 0.1x0.1 degree grid cell. For each grid cell the joint PAF was proportioned based on the proportion of exposure due to OAP and HAP, respectively. Thus the PAF of both the exposure were mutually exclusive of each other in GBD 2017. The table below indicates the equations used to calculate proportional PAFs:

PAF	Population not exposed to HAP	Population exposed to HAP
OAP	PAFOAP	(ExpOAP/(ExpOAP+ExpHAP))*PAFOAP+ HAP
HAP	0	(ExpHAP/(ExpOAP+ExpHAP))*PAFOAP+ HAP

A 1000 predicted values of the IER for each $PM_{2.5}$ concentration were calculated based on the posterior distributions of (α, β, γ) and the prespecified uniform distribution of TMREL to characterise uncertainty in the estimates of the IER. The mean of the 1000 IER predictions at each concentration was used as the central estimate, with uncertainty defined by 95% UIs.

C.2. Household air pollution

Exposure to HAP from solid fuels in GBD analysis was defined as the proportion of households using solid cooking fuels. The definition of solid fuel in this analysis includes coal, wood, charcoal, dung, and agricultural residues. The household exposure to solid fuels was converted to average $PM_{2.5}$ exposures from solid fuel use for different household members based on studies measuring 24-hour kitchen and living area $PM_{2.5}$ concentrations in households, and estimating this for men, women and children.

For cataract where the RR were extracted based on direct epidemiological evidence, TMREL was defined such that no households would report using solid fuel as their primary cooking fuel. For the other health outcomes that utilized evidence based on IER, TMREL was defined as a uniform distribution between 2.4 and 5.9 ug/m^3. To include the uncertainty in the TMREL, a random draw from the uniform distribution of the interval between 2.4 and 5.9 µg/m^3 each time the population attributable burden was calculated were taken.

The steps in the estimation of disease burden attributable to household air pollution are shown in the following flowchart:

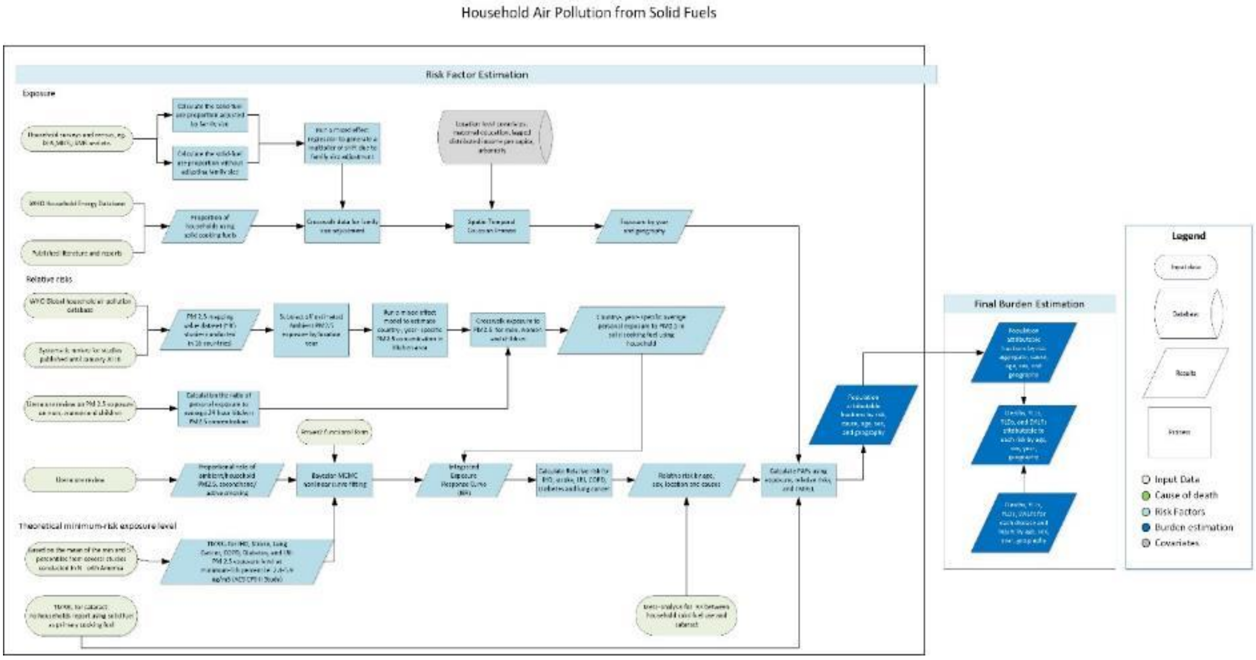

Data

There are many data sources on HAP from solid fuel use in India include national health surveys such as the National Family Health Survey and the District Level Household Survey, nationwide surveys of the National Sample Survey Organisation, and the Census of India, as well as other published and unpublished epidemiological studies.

Globally, data were extracted from the standard multi-country survey series such as Demographic and Health Surveys, Living Standards Measurement Surveys, Multiple Indicator Cluster Surveys, and World Health Surveys, as well as country-specific survey series. To fill the gaps of data in surveys and censuses, we also downloaded and updated HAP estimates from WHO Energy Database and extracted from literature through systematic review. Each nationally or sub-nationally representative data point provided an estimate for the percentage of households using solid cooking fuels. Estimates for the usage of solid fuels for non-cooking purpose were excluded, i.e. heating and primary fuels for lighting. The HAP database, with estimates from 1980 to 2017 contained about 680 studies form 150 countries.

In GBD 2017, the model was updated to estimate the individual exposure to $PM_{2.5}$ over and above ambient levels due to the use of solid cooking fuel. This was done by subtracting off the estimated ambient level $PM_{2.5}$ for the location-year of each study in the database before inputting them into the model. By doing this we derive at independent estimates for $PM_{2.5}$ exposure due to ambient and household solid fuel use. The average $PM_{2.5}$ exposures from solid fuel use for different household members were derived from studies measuring 24-hour kitchen and living area $PM_{2.5}$ concentrations in households, and estimating this for men, women and children separately.

These exposures were cross-walked to men, women, and children by generating the ratio of each group's mean exposure to the overall mean personal exposure. The resulting location, year, sex, and age specific $PM_{2.5}$ exposure values were used as inputs in the IER and attributable burden calculation process.

Modelling strategy

HAP was modelled at household level using a three-step modelling strategy that uses linear regression, spatiotemporal regression and Gaussian Process Regression (ST-GPR). The first step was a mixed-effect linear regression of logit-transformed proportion of households using solid cooking fuels. The linear model contains maternal education, proportion of population living in urban areas, and lagged-distributed income as covariates and has nested random effect by GBD region, and GBD super region respectively. Description of the full ST-GPR process is available in the GBD 2017 risk factors capstone paper (Lancet 2018; 392: 1923–94).

A variety of combinations of socioeconomic and environmental covariates in different transformation format were tested by running mixed-effect models with exposure data. The final list of covariates included in the exposure model were maternal education, proportion of population living in urban area, and lagged-distributed income since they proved to be the strongest predictors.

Generally, the disease-outcomes paired with HAP include LRI, stroke, IHD, COPD, lung cancer, and cataract. For GBD 2017, type 2 diabetes was included as a new outcome of HAP. The RRs of all outcomes, with the exception of cataracts, were generated by using IER, for which a new approach was adopted in GBD 2017, as described above in the section on ambient particulate matter pollution modelling. The RR for cataracts were extracted from a meta-analysis and was 2.47 with 95% (1.61, 3.73).[10] GBD currently only estimates cataracts as an outcome for females.

In order to use the IER curve, the exposure to particulate matter with diameter of less than 2.5 micrometres ($PM_{2.5}$) must be estimated. A mapping model relying on a database of almost 90 studies which measures $PM_{2.5}$ exposure in households using solid cooking fuel was utilised. Using socio-demographic index and study-level factors as covariates, the exposure was predicted for all location-years.

References

1. Turner MC, Jerrett M, Pope CA III, et al. Long-term ozone exposure and mortality in a large prospective study. *Am J Respir Crit Care Med* 2016; 193: 1134–42.

2. Shaddick G, Thomas M, Amini H, et al. Data integration for the assessment of population exposure to ambient air pollution for global burden of disease assessment. *Environ Sci Technol* 2018; 52: 9069–78.
3. Shaddick G, Thomas ML, Jobling A, et al. Data integration model for air quality: a hierarchical approach to the global estimation of exposures to ambient air pollution. *J R Stat Soc Ser C Appl Stat* 2018; 67: 231–53.
4. Rue H, Martino S, Chopin N. Approximate Bayesian inference for latent Gaussian models by using integrated nested Laplace approximations. *J R Stat Soc Ser B Stat Methedol* 2009; 71:319–92.
5. Cleveland WS, Devlin SJ. Locally weighted regression: an approach to regression analysis by local fitting. *J Am Stat Assoc* 1988; 83: 596–610.
6. Bowe B, Xie Y, Li T, Yan Y, Xian H, Al-Aly Z. The 2016 global and national burden of diabetes mellitus attributable to PM2.5 air pollution. *Lancet Planet Health* 2018; 2: e301–12.
7. Cohen AJ, Brauer M, Burnett R, et al. Estimates and 25-year trends of the global burden of disease attributable to ambient air pollution: an analysis of data from the Global Burden of Diseases Study 2015. *Lancet* 2017; 389: 1907–18.
8. Burnett RT, Pope CA III, Ezzati M, et al. An integrated risk function for estimating the global burden of disease attributable to ambient fine particulate matter exposure. *Environ Health Perspect* 2014; 122: 397–403.
9. Yin P, Brauer M, Cohen A, et al. Long-term fine particulate matter exposure and nonaccidental and cause-specific mortality in a large national cohort of Chinese men. *Environ Health Perspect* 2017; 125: 117002.
10. Smith KR, Bruce N, Balakrishnan K, *et al.* Millions dead: how do we know and what does it mean? Methods used in the comparative risk assessment of household air pollution. *Annu Rev Public Health* 2014; 35: 185–206.

D. Second-hand smoke

The steps in the estimation of second-hand smoke are shown in the following flowchart:

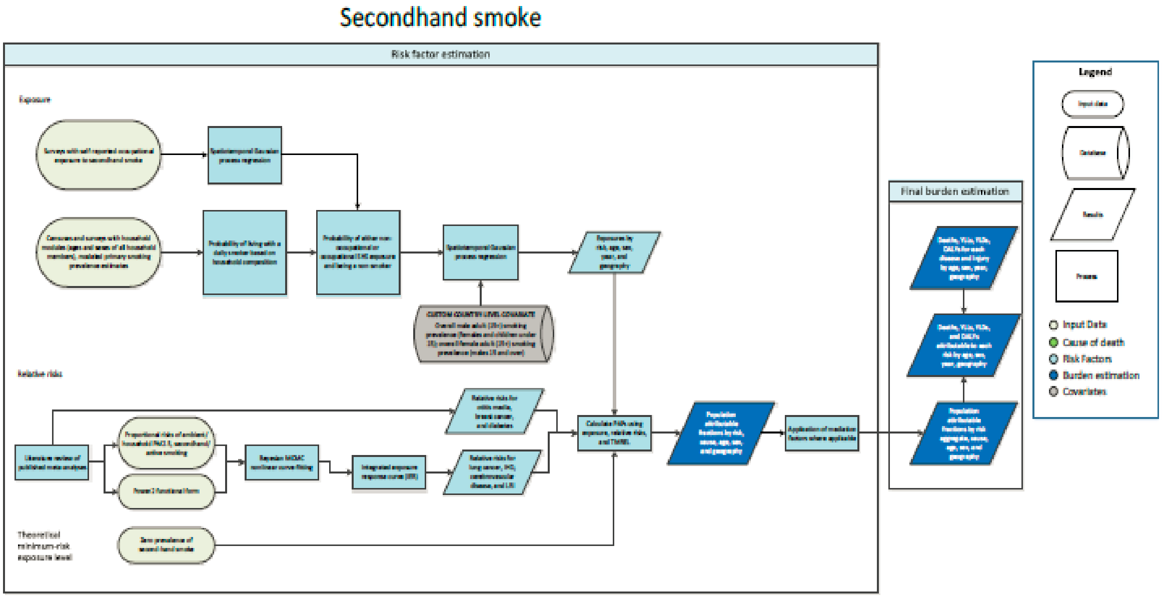

Exposure

Second-hand smoke exposure is defined as current exposure to second-hand tobacco smoke at home, at work, or in other public places. The household composition as a proxy for non-occupational second-hand smoke exposure and make the assumption that all persons living with a daily smoker are exposed to tobacco smoke were used. Surveys to estimate the proportion of individuals exposed to second-hand smoke at work were used. Non-smokers were the only one considered to be exposed to second-hand smoke. Non-smokers are defined as all persons who are not daily smokers. Ex-smokers and occasional smokers are considered non-smokers in this analysis. Exposure is evaluated for both children and adults.

Data

To calculate the proportion of non-smokers who live with at least one smoker, unit record data on household composition, which included the ages and sexes of all persons living in the same household were used. The major data sources for second hand smoke in India include national health surveys such as the National Family Health Survey, and the Global Adult Tobacco Surveys, as well as other published and unpublished epidemiological studies.

To calculate the proportion of individuals exposed to second-hand smoke at work, by age and sex, cross-sectional surveys that ask respondents about self-reported occupational second-hand smoke exposure were used. The other sources include Eurobarometer Surveys, and WHO STEPS Surveys.

Modelling strategy

The probability that each person is living with a smoker and is also a non-smoker themselves using set theory was estimated. First, household composition data were used at the individual level to capture the ages and sexes of each person in the household. Second, surveys with both household composition data and tobacco use questions were analysed and the distribution of household size, mean age of the household members, and the age distribution determined that not to be significantly different between households with and without a self-reported smoker. Since it was not found that household composition varied between smokers and non-smokers, the GBD 2017 primary smoking prevalence model was then used to calculate the probability that each household member is a smoker. Next, the probability of the union of sets on each individual household member was used to calculate the overall probability that at least one of the other household members was a smoker. Occupational exposure by modelling prevalence of current exposure to second-hand smoke at work, by age, sex, location, and year were incorporated, using ST-GPR. In order to avoid double counting the probability that an individual is exposed through either non-occupational exposure or occupational exposure were calculated, given their age, sex, and household composition. Finally, this probability of exposure was multiplied by the probability that the individual is not a smoker themselves (i.e. 1 minus primary smoking prevalence for that person's location, year, age, and sex). These individual-level probabilities were then collapsed to produce average probabilities of exposure by location, year, age, and sex.

These probabilities were modelled in the GBD ST-GPR framework, which generates exposure estimates from a mixed effects hierarchical linear model plus weighted residuals smoothed across time, space, and age. The linear model formula was fit separately by sex using restricted maximum likelihood in R.

The sex-specific overall smoking prevalence for adults (age 15 and older) were used as a country-level covariate in the model. The overall male adult daily smoking prevalence was used as the covariate for females of all ages and for males under age 15. The overall female adult daily smoking prevalence was use as the covariate for males age 15 and older. This was a modelling change from GBD 2015, in which the male age-standardised smoking prevalence was used for the adult female and children under-15 model, and the female age-standardised smoking prevalence for the adult male model.

All input data points from the probability calculation had a measure of uncertainty (variance and sample size) coming from the uncertainty of the primary smoking prevalence model and the sample size from the unit record data going into the modelling process. Geographic random effects were used in model fitting but were not used in prediction.

Theoretical minimum-risk exposure level

The theoretical minimum-risk exposure level for second-hand smoke is zero exposure among non-smokers, meaning that non-smokers would not live with any primary smokers.

Relative risks

For children ages 0-14, the burden of otitis media attributable to second-hand smoke exposure were estimated. For all ages, the burden of lower respiratory infections (LRI), and for adults greater or equal to 25 years of age the burden of lung cancer, chronic obstructive pulmonary disease (COPD), ischemic heart disease, and cerebrovascular disease attributable to secondhand smoke exposure, breast cancer, and type 2 diabetes were estimated.

For lung cancer, ischemic heart disease, cerebrovascular disease, and LRI, country-specific relative risks created using integrated exposure response curves (IER) were used for $PM_{2.5}$ air pollution. The relative risks for otitis media, breast cancer, and diabetes are derived from published meta-analyses. The standard GBD population attributable fraction (PAF) equation to estimate burden were used based onexposure and relative risks.

4. Data inputs for child mortality, cause of death, risk factors and covariates for India

Abel R, Rajaratnam J, Gnanasekaran VJ, Jayaraman P. Prevalence of anaemia and iron deficiency in three trimesters in rural Vellore district, South India. Trop Doct. 2001; 31:86-9.

Abel R, Rajaratnam J, Kalaimani A, Kirubakaran S. Can iron status be improved in each of the three trimesters? A community-based study. Eur J Clin Nutr. 2000; 54:490-3.

Abel R, Sampathkumar V. Tamil Nadu nutritional survey comparing children aged 0-3 years with the NCHS/CDC reference population. Indian J Pediatr. 1998; 65:565-72.

Agarwal D, Pandey C, Agarwal K. Vitamin A administration and preschool child mortality. Nutr Res. 1995; 15:669–80. as it appears in Imdad A, Herzer K, Mayo-Wilson E, Yakoob MY, Bhutta ZA. Vitamin A supplementation for preventing morbidity and mortality in children from 6 months to 5 years of age. Cochrane Database Syst Rev. 2010; CD008524.

Agarwal DK, Bhardwaj B, Singla PN, Tripathi AM, Agarwal KN. Etiology of maternal and early childhood deficiency anemia. Indian J Pediatr. 1986; 53:389-95.

Ahankari A, Bapat S, Myles P, Fogarty A, Tata L. Factors associated with preterm delivery and low birth weight: a study from rural Maharashtra, India. F1000Res. 2017; 6:72.

Allam RR, Murhekar MV, Bhatnagar T, Uthappa CK, Chava N, Rewari BB, Venkatesh S, Mehendale S. Survival probability and predictors of mortality and retention in care among patients enrolled for first-line antiretroviral therapy, Andhra Pradesh, India, 2008-2011. Trans R Soc Trop Med Hyg. 2014; 108: 198–205

Alvarez-Uria G, Naik PK, Pakam R, Midde M. Factors associated with attrition, mortality, and loss to follow up after antiretroviral therapy initiation: data from an HIV cohort study in India. Glob Health Action 2013; 6: 21682.

Anand K, Kant S, Kumar G, Kapoor SK. "Development" is not essential to reduce infant mortality rate in India: experience from the Ballabgarh project. J Epidemiol Community Health. 2000; 54: 247-53

Anand K, Patro BK, Paul E, Kapoor SK. Management of Sick Children by Health Workers in Ballabgarh: Lessons for Implementation of IMCI in India. J Trop Pediatr. 2004; 50: 41-7.

Anderegg N, Johnson LF, Zaniewski E, Althoff KN, Balestre E, Law M, Nash D, Shepherd BE, Yiannoutsos CT, Egger M, IeDEA, MeSH consortia. All-cause mortality in HIV-positive adults starting combination antiretroviral therapy: correcting for loss to follow-up. AIDS. 2017;31: S31–40

Arya S, Chellani H, Pandey J. Evaluation of safety of oral vitamin "A" megadose co-administered with measles vaccination. Indian Pediatr. 2000; 37:1341–7. as it appears in Imdad A, Herzer K, Mayo-Wilson E, Yakoob MY, Bhutta ZA. Vitamin A supplementation for preventing morbidity and mortality in children from 6 months to 5 years of age. Cochrane Database Syst Rev. 2010; CD008524.

Atkinson RW, Carey IM, Kent AJ, van Staa TP, Anderson HR, Cook DG. Long-term exposure to outdoor air pollution and the incidence of chronic obstructive pulmonary disease in a national English cohort. Occup Environ Med 2015; 72: 42–8.

Atkinson RW, Cohen A, Mehta S, Anderson HR. Systematic review and meta-analysis of epidemiological time-series studies on outdoor air pollution and health in Asia. Air Qual Atmos Health 2012; 5: 383–391.

Awasthi S, Das R, Verma T, Vir S. Anemia and undernutrition among preschool children in Uttar Pradesh, India. Indian Pediatr. 2003; 40:985-90.

Awasthi S, Pande VK. Cause-specific mortality in under fives in the urban slums of Lucknow, north India. J Trop Pediatr. 1998; 44:358-61.

Awasthi S, Peto R, Read S, Clark S, Pande V, Bundy D, DEVTA (Deworming and Enhanced Vitamin A) team. Vitamin A supplementation every 6 months with retinol in 1 million pre-school children in north India: DEVTA, a cluster-randomised trial. Lancet. 2013; 381:1469-77.

Badrinath S, Sharma T, Biswas J, Srinivas V. A case control study of senile cataract in a hospital based population. Indian J Ophthalmol 1996; 44: 213–217.

Bahl R, Frost C, Kirkwood BR, Edmond K, Martines J, Bhandari N, Arthur P. Infant feeding patterns and risks of death and hospitalization in the first half of infancy: multicentre cohort study. Bull World Health Organ. 2005; 83:418-26.

Bains K, Mann SK. Physical Fitness in Relation to Energy and Iron Status of Female College Students. Food Nutr Bull. 2000; 21:305–10.

Baker KK, O'Reilly CE, Levine MM, Kotloff KL, Nataro JP, Ayers TL, et al. Sanitation and hygiene-specific risk factors for moderate-to-severe diarrhea in young children in the Global Enteric Multicenter Study, 2007-2011: case-control study. PLoS Med. 2016; 13: e1002010

Balakrishnan K, Sambandam S, Ramaswamy P, Mehta S, Smith KR. Exposure assessment for respirable particulates associated with household fuel use in rural districts of Andhra Pradesh, India. J Expo Anal Environ Epidemiol 2004; 14: S14-25.

Balakrishnan K, Sankar S, Parikh J, et al. Daily average exposures to respirable particulate matter from combustion of biomass fuels in rural households of southern India. Environ Health Perspect 2002; 110: 1069-75.

Bamji MS, V S Murthy PV, Williams L, Vardhana Rao MV. Maternal nutritional status & practices & perinatal, neonatal mortality in rural Andhra Pradesh, India. Indian J Med Res. 2008; 127:44-51.

Bang AT, Bang RA, Baitule SB, Reddy MH, Deshmukh MD. Effect of home-based neonatal care and management of sepsis on neonatal mortality: field trial in rural India. Lancet. 1999; 354:1955-61.

Bang AT, Bang RA, Reddy HM, Deshmukh MD, Baitule SB. Reduced incidence of neonatal morbidities: effect of home-based neonatal care in rural Gadchiroli, India. J Perinatol. 2005; 25 (Suppl 1): S51-61.

Bang AT, Bang RA, Tale O, Sontakke P, Solanki J, Wargantiwar R, Kelzarkar P. Reduction in pneumonia mortality and total childhood mortality by means of community-based intervention trial in Gadchiroli, India. Lancet. 1990; 336: 201-6.

Bapat U, Alcock G, More NS, Das S, Joshi W, Osrin D. Stillbirths and newborn deaths in slum settlements in Mumbai, India: a prospective verbal autopsy study. BMC Pregnancy Childbirth. 2012; 12.

Baqui AH, Darmstadt GL, Williams EK, Kumar V, Kiran TU, Panwar D, Srivastava VK, Ahuja R, Black RE, Santosham M. Rates, timing and causes of neonatal deaths in rural India: implications for neonatal health programmes. Bull World Health Organ. 2006; 84: 706-13.

Baruah J, Kusre G, Bora R. Pattern of Gross Congenital Malformations in a Tertiary Referral Hospital in Northeast India. Indian J Pediatr. 2015; 82:917–22.

Basavanagowdappa H, Guruswamy M, Ravi MD, Mahesh PA. JSS Hospital inpatient data 2014-2017. JSS Hospital, Mysuru, Karnataka, India.

Beelen R, Hoek G, van den Brandt PA, et al. Long-term effects of traffic-related air pollution on mortality in a Dutch cohort (NLCS-AIR study). Environ Health Perspect 2008; 116: 196–202.

Beelen R, Stafoggia M, Raaschou-Nielsen O, et al. Long-term exposure to air pollution and cardiovascular mortality: an analysis of 22 European cohorts. Epidemiology 2014; 25: 368–78.

Behera D. An analysis of effect of common domestic fuels on respiratory function. Indian J Chest Dis Allied Sci 1997; 39:235–243.

Benara SK, Singh P. Validity of causes of infant death by verbal autopsy. Indian Pediatr. 1999; 66: 647-50.

Bhandari N, Bahl R, Taneja S, Martines J, Bhan MK. Pathways to infant mortality in urban slums of Delhi, India: implications for improving the quality of community- and hospital-based programmes. J Health Popul Nutr. 2002; 20: 148-55

Bhandari N, Mazumder S, Taneja S, Sommerfelt H, Strand TA. Effect of implementation of Integrated Management of Neonatal and Childhood Illness (IMNCI) programme on neonatal and infant mortality: cluster randomised controlled trial. BMJ. 2012; 344: e1634.

Bharati P, Ghosh R, Gupta R. Socioeconomic Condition and Anaemia among the Mahishya Population of Southern West Bengal, India. Malays J Nutr. 2004; 10:23-30.

Bhargava M, Iyer PU, Kumar R, Ramji S, Kapani V, Bhargava SK. Relationship of maternal serum ferritin with foetal serum ferritin, birth weight and gestation. J Trop Pediatr. 1991; 37:149-52.

Bhargava SK, Singh KK, Saxena BN, India Council of Medical Research (ICMR). ICMR Task Force National Collaborative Study on Identification of High Risk Families, Mothers and Outcome of their Off-springs with particular reference to the problem of maternal nutrition, low birth weight, perinatal and infant morbidity and mortality in rural and urban slum communities. Summary, conclusions and recommendations. 1991; 28:1473-80.

Biran A, Schmidt WP, Wright R, Jones T, Seshadri M, Isaac P, et al. The effect of a soap promotion and hygiene education campaign on handwashing behaviour in rural India: a cluster randomised trial. Trop Med Int Health. 2009; 14: 1303-14

Biswas R, Biswas AB, Manna B, Bhattacharya SK, Dey R, Sarkar S. Effect of vitamin A supplementation on diarrhoea and acute respiratory tract infection in children. A double blind placebo controlled trial in a Calcutta slum community. Eur J Epidemiol. 1994; 10: 57–61. as it appears in Imdad A, Herzer K, Mayo-Wilson E, Yakoob MY, Bhutta ZA. Vitamin A supplementation for preventing morbidity and mortality in children from 6 months to 5 years of age. Cochrane Database Syst Rev. 2010; CD008524.

Blencowe H, Cousens S, Jassir FB, Say L, Chou D, Mathers C, Hogan D, Shiekh S, Qureshi ZU, You D, Lawn JE; Lancet Stillbirth Epidemiology Investigator Group. National, regional, and worldwide estimates of stillbirth rates in 2015, with trends from 2000: a systematic analysis. Lancet Glob Health. 2016; 4:e98-108.

Boisson S, Stevenson M, Shapiro L, Kumar V, Singh LP, Ward D, Clasen T. Effect of household-based drinking water chlorination on diarrhoea among children under five in Orissa, India: a double-blind randomised placebo-controlled trial. PLoS Med. 2013; 10: e1001497

Bonu S, Rani M, Jha P, Peters DH, Nguyen SN. Household tobacco and alcohol use, and child health: an exploratory study from India. Health Policy 2004; 70: 67–83.

Brauer M, Hoek G, Van Vliet P, et al. Air pollution from traffic and the development of respiratory infections and asthmatic and allergic symptoms in children. Am J Respir Crit Care Med 2002; 166: 1092–8.

Brinkhof MWG, Dabis F, Myer L, Bangsberg DR, Boulle A, Nash D, Schechter M, Laurent C, Keiser O, May M, Sprinz E, Egger M, Anglaret X, ART-LINC, IeDEA. Early loss of HIV-infected patients on potent antiretroviral therapy programmes in lower-income countries. Bull World Health Organ. 2008; 86: 559-67

Burnett RT, Pope CA, Ezzati M, et al. An integrated risk function for estimating the global burden of disease attributable to ambient fine particulate matter exposure. Environ Health Perspect 2014; 122: 397–403.

Carey IM, Atkinson RW, Kent AJ, van Staa T, Cook DG, Anderson HR. Mortality associations with long-term exposure to outdoor air pollution in a national English cohort. Am J Respir Crit Care Med 2013; 187: 1226–33.

Center for Vaccine Development, University of Maryland, Centers for Disease Control and Prevention, Department of Medical Microbiology and Immunology, Göteborg University, International Vaccine Institute, National Institute of Cholera and Enteric Diseases, Perry Point Cooperative Studies Program Coordinating Center, U.S. Department of Veterans Affairs, School of Medicine, University of Virginia, University of Chile. India - Kolkata Global Enteric Multicenter Study 2007-2011. Baltimore, MD, United States: Center for Vaccine Development, University of Maryland.

Central Pollution Control Board (CPCB), Ministry of Environment, Forest and Climate Change, Government of India. India - National Air Quality Monitoring Programme Data 2010. New Delhi, India: CPCB.

Central Pollution Control Board (CPCB), Ministry of Environment, Forest and Climate Change, Government of India. India - National Air Quality Monitoring Programme Data 2011. New Delhi, India: CPCB.

Central Pollution Control Board (CPCB), Ministry of Environment, Forest and Climate Change, Government of India. India - National Air Quality Monitoring Programme Data 2012. New Delhi, India: CPCB.

Central Pollution Control Board (CPCB), Ministry of Environment, Forest and Climate Change, Government of India. India - National Air Quality Monitoring Programme Data 2013. New Delhi, India: CPCB.
Central Pollution Control Board (CPCB), Ministry of Environment, Forest and Climate Change, Government of India. India - National Air Quality Monitoring Programme Data 2014. New Delhi, India: CPCB.
Central Pollution Control Board (CPCB), Ministry of Environment, Forest and Climate Change, Government of India. India - National Air Quality Monitoring Programme Data 2015. New Delhi, India: CPCB.
Central Pollution Control Board (CPCB), Ministry of Environment, Forest and Climate Change, Government of India. India - National Air Quality Monitoring Programme Data 2016. New Delhi, India: CPCB.
Centre for Research on the Epidemiology of Disasters (CRED), Catholic University of Leuven. EM-DAT: The OFDA/CRED International Disaster Database. Brussels, Belgium: Catholic University of Leuven.
Chakraborty B, Bhattacharya AR. Xerophthalmia in under-five children in West Bengal. Indian Pediatr. 1987; 24:600-2.
Chen Y, Ebenstein A, Greenstone M, Hongbin L. Evidence on the impact of sustained exposure to air pollution on life expectancy from China's Huai River policy. Proc Natl Acad Sci 2013; 110: 12936–12941
Chiplonkar S, Agte V, Mengale S. Relative importance of micronutrient deficiencies in iron deficiency anemia. Nutr Res. 2003; 23:1355-67.
Chowdhury S, Kumar R, Ganguly NK, Kumar L, Walia BNS. Effect of vitamin A supplementation on childhood morbidity and mortality. Indian J Med Sci. 2002; 56:259-64. as it appears in Imdad A, Herzer K, Mayo-Wilson E, Yakoob MY, Bhutta ZA. Vitamin A supplementation for preventing morbidity and mortality in children from 6 months to 5 years of age. Cochrane Database Syst Rev. 2010; CD008524.
Christian Medical College - Vellore, Fogarty International Center, National Institutes of Health, Foundation for the National Institutes of Health. India - Vellore Malnutrition and Enteric Disease (MAL-ED) Study 2009-2014. [Data shared for this analysis]
Cohen AJ, Brauer M, Burnett R, et al. Estimates and 25-year trends of the global burden of disease attributable to ambient air pollution: an analysis of data from the Global Burden of Diseases Study 2015. Lancet 2017; 389: 1907–18.
Dandona R, Rewari BB, Kumar GA, Tanwar S, Kumar SGP, Vishnumolakala VS, Duber HC, Gakidou E, Dandona L. Survival outcomes for first-line antiretroviral therapy in India's ART program. BMC Infect Dis. 2016; 16: 555
Das S, Bapat U, More NS, Alcock G, Fernandez A, Osrin D. Nutritional status of young children in Mumbai slums: a follow-up anthropometric study. Open Nutr J. 2012; 11:100.
Department of Economics & Statistics, Government of Kerala. India-Kerala Annual vital statistics perport-2014. Thiruvananthapuram, India: Vital statistics division, Department of Economics & Statistics, Government of Kerala; 2016.
Department of Economics & Statistics, Government of Kerala. India-Kerala Annual vital statistics perport-2015. Thiruvananthapuram, India: Vital statistics division, Department of Economics & Statistics, Government of Kerala; 2017.
Department of Women and Child Development, Ministry of Human Resource Development, Government of India, United Nations Children's Fund (UNICEF) - India Country Office. India Summary Report on the Multiple Indicator Cluster Survey 2000. New Delhi, India: UNICEF India Country Office; 2000.
Department of Women and Child Development, Ministry of Human Resource Development, Government of India. India Nutrition Profile Survey 1995-1996. New Delhi, India: Ministry of Human Resource Development.
Desai, Sonalde, Reeve Vanneman, National Council of Applied Economic Research, University of Michigan. India Human Development Survey 2005. Ann Arbor, Michigan: Inter-University Consortium for Political and Social Research.
Desai, Sonalde, Reeve Vanneman, National Council of Applied Economic Research, University of Michigan. India Human Development Survey II, 2011-12. Ann Arbor, Michigan: Inter-University Consortium for Political and Social Research.
Deshmukh V, Lahariya C, Krishnamurthy S, Das MK, Pandey RM, Arora NK. Taken to Health Care Provider or Not, Under-Five Children Die of Preventable Causes: Findings from Cross-Sectional Survey and Social Autopsy in Rural India. Indian J Community Med. 2016; 41:108–19.
Dherani M, Pope D, Mascarenhas M, Smith KR, Weber M, Bruce N. Indoor air pollution from unprocessed solid fuel use and pneumonia risk in children aged under five years: a systematic review and meta-analysis. Bull World Health Organ 2008; 86: 390-398C.
Directorate of Economics & Statistics and Office of Chief Registrar (Births & Deaths), Government of National Capital Territory of Delhi. Report on Medical Certification of Cause of Deaths in Delhi-2011. New Delhi, India.
Directorate of Economics & Statistics and Office of Chief Registrar (Births & Deaths), Government of National Capital Territory of Delhi. Report on Medical Certification of Cause of Deaths in Delhi-2011. New Delhi, India: Directorate of Economics & Statistics and Office of Chief Registrar (Births & Deaths); 2012.
Directorate of Economics & Statistics and Office of Chief Registrar (Births & Deaths), Government of National Capital Territory of Delhi. Report on Medical Certification of Cause of Deaths in Delhi-2012. New Delhi, India: Directorate of Economics & Statistics and Office of Chief Registrar (Births & Deaths); 2013.
Directorate of Economics & Statistics and Office of Chief Registrar (Births & Deaths), Government of National Capital Territory of Delhi. Report on Medical Certification of Cause of Deaths in Delhi-2013. New Delhi, India: Directorate of Economics & Statistics and Office of Chief Registrar (Births & Deaths); 2014.
Directorate of Economics & Statistics and Office of Chief Registrar (Births & Deaths), Government of National Capital Territory of Delhi. Report on Medical Certification of Cause of Deaths in Delhi-2014. New Delhi, India: Directorate of Economics & Statistics and Office of Chief Registrar (Births & Deaths); 2016.
Directorate of Economics & Statistics and Office of Chief Registrar (Births & Deaths), Government of National Capital Territory of Delhi. Report on Medical Certification of Cause of Deaths in Delhi-2015. New Delhi, India: Directorate of Economics & Statistics and Office of Chief Registrar (Births & Deaths); 2016.

Directorate of Economics & Statistics and Office of Chief Registrar (Births & Deaths), Government of National Capital Territory of Delhi. Report on Medical Certification of Cause of Deaths in Delhi-2016. New Delhi, India: Directorate of Economics & Statistics and Office of Chief Registrar (Births & Deaths); 2017.

Directorate of Economics & Statistics, Government of Chhattisgarh. India- Chhattisgarh Medical Certification of Cause of Death Report 2014. Raipur, India: Directorate of Economics and Statistics, Government of Chhattisgarh.

Directorate of Economics & Statistics, Government of Chhattisgarh. India- Chhattisgarh Medical Certification of Cause of Death Report 2015. Raipur, India: Directorate of Economics and Statistics, Government of Chhattisgarh.

Directorate of Economics & Statistics, Government of Jharkhand. India- Jharkhand Medical Certification of Cause of Death Report 2014-2015. Ranchi, India: Directorate of Economics and Statistics, Government of Jharkhand.

Directorate of Economics & Statistics, Government of Karnataka, India- Karnataka Medical Certification of Cause of Death Report 2014. Bangalore India: Directorate of Economics and Statistics, Government of Karnataka,

Directorate of Economics & Statistics, Government of Karnataka, India- Karnataka Medical Certification of Cause of Death Report 2015. Bangalore India: Directorate of Economics and Statistics, Government of Karnataka,

Directorate of Economics and Statistics and Office of Chief Registrar Births and Deaths (Delhi Territory, India). India - Delhi Medical Certification of Cause of Death Report 2013

Dogra V, Khanna R, Jain A, Kumar AMV, Shewade HD, Majumdar SS. Neonatal mortality in India's rural poor: Findings of a household survey and verbal autopsy study in Rajasthan, Bihar and Odisha. J Trop Pediatr. 2015; 61: 210–4.

Earth Observation Group - Defense Meteorological Satellite Progam, Boulder. Version 4 DMSP-OLS Nighttime Lights Time Series. https://www.ngdc.noaa.gov/eog/dmsp/downloadV4composites.html (accessed July, 2017).

Embassy of the United States - New Delhi, United States Environmental Protection Agency. United States Mission India Now Cast Air Quality Data 2014. New Delhi, India: Embassy of the United States.

Fakhir S, Srivastava I, Ahmad P, Hasan SS. Prevalence of xerophthalmia in pre-school children in an urban slum. Indian Pediatr. 1993; 30:668-70.

Fan Y, Ajay A. What prevents diarrhoea? The impacts of water supply, toilets, and hand-washing in rural India. J Dev Eff. 2011; 3: 340-70

FAO Supply Utilization Accounts 1961-2013. Personal Correspondence with Dr. Josef Schmidhuber, 2016. [Data shared for this analysis]

Fatmi Z, Coggon D. Coronary heart disease and household air pollution from use of solid fuel: a systematic review. Br Med Bull 2016; 118: 91–109.

Feldon K, Bahl S, Bhatnagar P, Wenger J. Severe vitamin A deficiency in India during pulse polio immunization. Indian J Med Res. 2005; 122:265-7.

Food and Agriculture Organization of the United Nations (FAO). FAOSTAT Food Balance Sheets, October 2014. Rome, Italy: FAO.

Ford Foundation, Nutrition Foundation of India. India Profiles of Undernutrition and Underdevelopment: Studies of Poor Communities in Seven Regions of the Country. New Delhi, India: Nutrition Foundation of India. (Scientific Report Series No.8).

Gan WQ, FitzGerald JM, Carlsten C, Sadatsafavi M, Brauer M. Associations of ambient air pollution with chronic obstructive pulmonary disease hospitalization and mortality. Am J Respir Crit Care Med 2013; 187: 721-7.

Genser B, Strina A, Santos LA, et al. Impact of a city-wide sanitation intervention in a large urban centre on social, environmental and behavioural determinants of childhood diarrhoea: analysis of two cohort studies. Int J Epidemiol 2008; 37:831–40.

George K, Prasad J, Singh D, Minz S, Albert DS, Muliyil J, Joseph KS, Jayaraman J, Kramer MS. Perinatal outcomes in a South Asian setting with high rates of low birth weight. BMC Pregnancy Childbirth. 2009 Feb; 9:5.

Gething PW, Casey DC, Weiss DJ, Bisanzio D, Bhatt S, Cameron E, et al. Mapping Plasmodium falciparum Mortality in Africa between 1990 and 2015. New England Journal of Medicine. 2016;375: 2435-45.

Ghosh SK, Yadav RS, Das BS, Sharma VP. Influence of nutritional and haemoglobin status on malaria infection in children. Indian J Pediatr. 1995; 62:321-6.

Ghosh R, Sharma AK. Determinants of tetanus and sepsis among the last neonatal deaths at household level in a peri-urban area of India. Postgrad Med J. 2011; 87: 257-63.

Global Burden of Disease Collaborative Network. Global Health Spending 1995-2014. Seattle, United States: Institute for Health Metrics and Evaluation, 2017

Global Burden of Disease Health Financing Collaborator Network, Institute for Health Metrics and Evaluation (IHME). Global Development Assistance for Health, Government, Prepaid Private, and Out-of-Pocket Health Spending 1995-2014. Seattle, United States: IHME; 2017.

Gomber S, Kumar S, Rusia U, Gupta P, Agarwal KN, Sharma S. Prevalence & etiology of nutritional anaemias in early childhood in an urban slum. Indian J Med Res. 1998; 107: 269-73.

Goudar SS, Goco N, Somannavar MS, Vernekar SS, Mallapur AA, Moore JL, Wallace DD, Sloan NL, Patel A, Hibberd PL, Koso-Thomas M, McClure EM, Goldenberg RL. Institutional deliveries and perinatal and neonatal mortality in Southern and Central India. Reprod Health. 2015; 12: S13.

Government of Orissa, Micronutritent Initiative, United Nations Children's Fund, World Health Organization.Orissa Impact of Vitamin A Supplementation Delivered with Oral Polio Vaccine as Part of Immunization Campaign [Draft final report]. Bhubheshwar, India: Government of Orissa; 2001.

Gupta D, Boffetta P, Gaborieau V, Jindal S. Risk factors of lung cancer in Chandigarh, India. Ind J Med Res. 2001; 113:142–150.

Gupta V, Shukla K. Epidemiology of anaemia in pre-school children from a rural and a slum community of Varanasi. Indian J Prev Soc Med. 1985; 85-9.

Health Management Information System (HMIS), National Health Mission, Ministry of Health and Family Welfare, Government of India. India Performance Related to Immunisation 2007-2009. New Delhi, India: Ministry of Health and Family Welfare.

Health Management Information System (HMIS), National Health Mission, Ministry of Health and Family Welfare, Government of India. India Performance Related to Immunisation 2008-2010. New Delhi, India: Ministry of Health and Family Welfare.

Health Management Information System (HMIS), National Health Mission, Ministry of Health and Family Welfare, Government of India. India Performance Related to Immunisation 2009-2011. New Delhi, India: Ministry of Health and Family Welfare.

Health Management Information System (HMIS), National Health Mission, Ministry of Health and Family Welfare, Government of India. India Performance Related to Immunisation 2010-2012. New Delhi, India: Ministry of Health and Family Welfare.

Health Management Information System (HMIS), National Health Mission, Ministry of Health and Family Welfare, Government of India. India Performance Related to Immunisation 2011-2013. New Delhi, India: Ministry of Health and Family Welfare.

Health Management Information System (HMIS), National Health Mission, Ministry of Health and Family Welfare, Government of India. India Performance Related to Immunisation 2012-2014. New Delhi, India: Ministry of Health and Family Welfare.

Health Management Information System (HMIS), National Health Mission, Ministry of Health and Family Welfare, Government of India. India Performance Related to Immunisation 2013-2015. New Delhi, India: Ministry of Health and Family Welfare.

Health Management Information System (HMIS), National Health Mission, Ministry of Health and Family Welfare, Government of India. India Performance Related to Immunisation 2014-2016. New Delhi, India: Ministry of Health and Family Welfare.

Health Management Information System (HMIS), National Health Mission, Ministry of Health and Family Welfare, Government of India. India Performance Related to Immunisation 2015-2017. New Delhi, India: Ministry of Health and Family Welfare.

Hebert JR, Gupta PC, Bhonsle RB, Sinor PN, Mehta H, Mehta FS. Development and testing of a quantitative food frequency questionnaire for use in Gujarat, India. Public Health Nutr. 1999; 2:39–50.

Hertz-Picciotto I, Baker RJ, Yap P-S, et al. Early childhood lower respiratory illness and air pollution. Environ Health Perspect 2007; 115: 1510–8.

Hirve S, Ganatra B. A prospective cohort study on the survival experience of under five children in rural western India. Indian Pediatr. 1997; 34:995-1001.

Hoek G, Krishnan RM, Beelen R, et al. Long-term air pollution exposure and cardio- respiratory mortality: a review. Environ Health 2013; 12: 43.

Honda T, Pun VC, Manjourides J, Suh H. Associations between long-term exposure to air pollution, glycosylated hemoglobin and diabetes. Int J Hyg Environ Health 2017; 220: 1124–32.

Horta BL, Victora CG, Organization WH. Short-term effects of breastfeeding: a systematic review on the benefits of breastfeeding on diarrhoea and pneumonia mortality. World Health Organization; 2013 https://apps.who.int/iris/handle/10665/95585 (accessed April 15, 2019)

Huang F, Pan B, Wu J, Chen E, Chen L. Relationship between exposure to PM2.5 and lung cancer incidence and mortality: a meta-analysis. Oncotarget 2017; 8: 43322–31.

Hystad P, Demers PA, Johnson KC, Carpiano RM, Brauer M. Long-term residential exposure to air pollution and lung cancer risk. Epidemiology 2013; 24: 762–72.

Imdad A, Herzer K, Mayo-Wilson E, Yakoob MY, Bhutta ZA. Vitamin A supplementation for preventing morbidity and mortality in children from 6 months to 5 years of age. Cochrane Database Syst Rev. 2010; CD008524.

India Neonatal Preterm Birth Complications Data 1995 as provided by the Global Burden of Disease 2010 Neonatal Conditions Expert Group. [Data shared for this analysis]

Indian Council of Medical Research (ICMR). India Study on Causes of Death by Verbal Autopsy 2003. New Delhi, India: ICMR. [Data shared for this analysis]

Indian Institute of Health Management Research, International Institute of Population Sciences, Ministry of Health and Family Welfare, Government of India, Performance Monitoring and Accountability 2020 (PMA2020) Project, Bill & Melinda Gates Institute for Population and Reproductive Health, Johns Hopkins Bloomberg School of Public Health. PMA 2016 Rajasthan Round 1 Survey Data as part of PMA 2020 Project. Baltimore, United States: PMA 2020.

INDEPTH. Africa, Asia, Oceania - INDEPTH Network Cause-Specific Mortality - Release 2014. Accra, Ghana: INDEPTH, 2014.

Institute of Health Systems. India Cause of Death Dataset Version 1.3 1980-1998. Hyderabad, India: Institute of Health Systems; 2002.

International Institute for Population Sciences (IIPS), Ministry of Health and Family Welfare, Government of India, Macro International. India National Family Health Survey Data (NFHS-1) 1992-1993. Mumbai, India: IIPS.

International Institute for Population Sciences (IIPS), Ministry of Health and Family Welfare, Government of India, Macro International. India National Family Health Survey (NFHS-1) 1992-1993: National Report. Mumbai, India: IIPS.

International Institute for Population Sciences (IIPS), Ministry of Health and Family Welfare, Government of India, ICF. India National Family Health Survey Data (NFHS-2) 1998-1999 Mumbai, India: IIPS.

International Institute for Population Sciences (IIPS), Ministry of Health and Family Welfare, Government of India, ICF. India National Family Health Survey Data (NFHS-3) 2005-2006: Mumbai, India: IIPS.

International Institute for Population Sciences (IIPS), Ministry of Health and Family Welfare, Government of India, Macro International. India National Family Health Survey (NFHS-3) 2005-2006: National and State Reports. Mumbai, India: IIPS.

International Institute for Population Sciences (IIPS), Ministry of Health and Family Welfare, Government of India, ICF. Indi a National Family Health Survey data (NFHS-4) 2015-2016. Mumbai, India: IIPS.

International Institute for Population Sciences (IIPS), Ministry of Health and Family Welfare, Government of India, ICF. India National Family Health Survey (NFHS-4) 2015-2016: National and State Reports. Mumbai, India: IIPS.

International Institute for Population Sciences (IIPS), Ministry of Health and Family Welfare, Government of India, ICF. India National Family Health Survey (NFHS-4) 2015-2016: National and State-level Factsheets. Mumbai, India: IIPS.

International Institute for Population Sciences (IIPS), Ministry of Health and Family Welfare, Government of India. India District Level Household Survey Data (DLHS-1) 1998-1999. Mumbai, India: IIPS.

International Institute for Population Sciences (IIPS), Ministry of Health and Family Welfare, Government of India. India District Level Household Survey (DLHS-1) 1998-1999: National Report. Mumbai, India: IIPS.

International Institute for Population Sciences (IIPS), Ministry of Health and Family Welfare, Government of India. India District Level Household Survey Data (DLHS-2) 2002-2004. Mumbai, India: IIPS.

International Institute for Population Sciences (IIPS), Ministry of Health and Family Welfare, Government of India. India District Level Household Survey (DLHS-2) 2002-2004: National and State Reports. Mumbai, India: IIPS.

International Institute for Population Sciences (IIPS), Ministry of Health and Family Welfare, Government of India. India District Level Household and Facility Survey Data (DLHS-3) 2007-2008. Mumbai, India: IIPS.

International Institute for Population Sciences (IIPS), Ministry of Health and Family Welfare, Government of India. India District Level Household and Facility Survey (DLHS-3) 2007-2008: National and State Reports. Mumbai, India: IIPS.

International Institute for Population Sciences (IIPS), Ministry of Health and Family Welfare, Government of India. India District Level Household and Facility Survey Data (DLHS-4) 2012-2013. Mumbai, India: IIPS.

International Institute for Population Sciences (IIPS), Ministry of Health and Family Welfare, Government of India. India District Level Household and Facility Survey (DLHS-4) 2012-2013: State Reports. Mumbai, India: IIPS.

International Institute for Population Sciences, World Health Organization. India World Health Survey 2003.

International Social Survey Programme (ISSP), GESIS - Leibniz Institute of Social Sciences. ISSP: Family and Changing Gender Roles IV, ZA5900 (Version 4.0.0). Mannheim, Germany: GESIS - Leibniz Institute of Social Sciences; 2012. https://dbk.gesis.org/dbksearch/sdesc2.asp?no=5900&db=e&doi=10.4232/1.12661

Jain DL, Sarathi V, Upadhye D, Gulhane R, Nadkarni AH, Ghosh K, Colah RB. Newborn screening shows a high incidence of sickle cell anemia in Central India. Hemoglobin. 2012; 36:316–22.

Jain S, Chopra H, Garg SK, Bhatnagar M, Singh JV. Anemia in children: early iron supplementation. Indian J Pediatr. 2000; 67:19-21.

Jain Y, Bansal M, Tiwari R, Kasar PK. Causes of Neonatal Mortality: A community based study using verbal autopsy tool. Natl J Community Med. 2013; 4.

Jet Propulsion Laboratory, National Aeronautics and Space Administration. Aerosol Optical Depth data from Multi-angle Imaging Spectro Radiometer. Available from: https://www-misr.jpl.nasa.gov/getData/accessData/

Jobarteh K, Shiraishi RW, Malimane I, Samo Gudo P, Decroo T, Auld AF, Macome V, Couto A. Community ART support groups in Mozambique: The potential of patients as partners in care. PLoS One. 2016;11: e0166444

Joshi R, Cardona M, Iyengar S, Sukumar A, Raju CR, Raju KR, Raju K, Reddy KS, Lopez A, Neal B. Chronic diseases now a leading cause of death in rural India – mortality data from the Andhra Pradesh Rural Health Initiative. Int J Epidemiol. 2006; 35: 1522-9

Kameswaran C, Bhatia BD, Bhat BV, Oumachigui A. Perinatal mortality: a hospital based study. Indian Pediatr. 1993; 30:997-1001.

Kapoor D, Agarwal KN, Sharma S, Kela K, Kaur I. Iron status of children aged 9-36 months in an urban slum Integrated Child Development Services project in Delhi. Indian Pediatr. 2002; 39:136-44.

Kapoor RK, Srivastava AK, Misra PK, Sharma B, Thakur S, Srivastava KI, Singh GK. Perinatal mortality in urban slums in Lucknow. Indian Pediatr. 1996; 33: 19-23.

Karr C, Lumley T, Schreuder A, et al. Effects of subchronic and chronic exposure to ambient air pollutants on infant bronchiolitis. Am J Epidemiol 2007; 165: 553–60.

Karr CJ, Rudra CB, Miller KA, et al. Infant exposure to fine particulate matter and traffic and risk of hospitalization for RSV bronchiolitis in a region with lower ambient air pollution. Environ Res 2009; 109: 321–7.

Katanoda K, Sobue T, Satoh H, et al. An association between long-term exposure to ambient air pollution and mortality from lung cancer and respiratory diseases in Japan. J Epidemiol 2011; 21: 132–43.

Katz J, Lee AC, Kozuki N, et al. Mortality risk in preterm and small-for-gestational-age infants in low-income and middle-income countries: a pooled country analysis. Lancet 2013; 382: 417–25.

Kaushik SL, Parmar VR, Grover N, Kaushik R. Neonatal mortality rate: relationship to birth weight and gestational age. Indian J Pediatr. 1998; 65:429-33.

Khandait DW, Ambadekar NN, Zodpey SP, Vasudeo ND. Maternal age as a risk factor for stillbirth. Indian J Public Health. 2000; 44: 28-30.

Khanna R, Kumar A, Vaghela JF, Sreenivas V, Puliyel JM. Community based retrospective study of sex in infant mortality in India. BMJ. 2003; 327: 126.

King George’s Medical University. King George’s Medical University pediatric inpatient data 2017. King George’s Medical University, Lucknow, Uttar Pradesh, India.

Kodkany BS, Derman RJ, Honnungar NV, Tyagi NK, Goudar SS, Mastiholi SC, Moore JL, McClure EM, Sloan N, Goldenberg RL. Establishment of a Maternal Newborn Health Registry in the Belgaum district of Karnataka, India. Reprod Health. 2015; 12 Suppl 2: S3.

Kumar S, Vollmer S. Does access to improved sanitation reduce childhood diarrhea in rural India? Health Econ. 2013; 22: 410-27

Kumar V, Datta N, Wadhwa SS, Singhi S. Morbidity and mortality in diarrhea in rural Haryana Indian. Indian J Pediatr. 1985; 52:455-61.

Kumar R, Kapoor SK, Krishnan A. Performance of cause-specific childhood mortality surveillance by health workers using a short verbal autopsy tool. Southeast Asia J Public Health. 2012; 1: 151-158.

Kumar V, Garg BS. Global health and infant mortality: application of verbal autopsy tool to categorize infant deaths, ascertain their causes and identify the gaps in health management information system in India. Int J Curr Res Rev. 2014; 5: 87-94.

Kurmi OP, Arya PH, Lam K-BH, Sorahan T, Ayres JG. Lung cancer risk and solid fuel smoke exposure: a systematic review and meta-analysis. Eur Respir J 2012; 40: 1228–37.

Lepeule J, Laden F, Dockery D, Schwartz J. Chronic exposure to fine particles and mortality: an extended follow-up of the Harvard six cities study from 1974 to 2009. Environ Health Perspect 2012; 120: 965–70.

Lipsett MJ, Ostro BD, Reynolds P, et al. Long-term exposure to air pollution and cardiorespiratory disease in the California teachers study cohort. Am J Respir Crit Care Med 2011; 184: 828–35.

Lloyd CT, Sorichetta A, Tatem AJ. High resolution global gridded data for use in population studies. Scientific Data. 2017;4: 170001.

MacIntyre EA., Gehring U, Molter A, et al. Air pollution and respiratory infections during early childhood: an analysis of 10 European birth cohorts within the ESCAPE project. Environ Health Perspect 2014; 122: 107-13.

Mahapatra A, Geddam JJ, Marai N, Murmu B, Mallick G, Bulliyya G, Acharya AS, Satyanarayana K. Nutritional status of preschool children in the drought affected Kalahandi district of Orissa. Indian J Med Res. 2000; 111: 90-4.

Mathad V, Metgud C, Mallapur MD. Nutritional status of under-fives in rural area of South India. Indian J Med Sci. 2011; 65:151-6.

Max Planck Institute for Demographic Research, Vienna Institute of Demography. Human Fertility Collection - Total Fertility Rate and Mean Age at Birth. Rostock, Germany: Max Planck Institute for Demographic Research.

McClure EM, Pasha O, Goudar SS, Chomba E, Garces A, Tshefu A, Althabe F, Esamai F, Patel A, Wright LL, Moore J, Kodkany BS, Belizan JM, Saleem S, Derman RJ, Carlo WA, Hambidge KM, Buekens P, Liechty EA, Bose C, Koso-Thomas M, Jobe AH, Goldenberg RL; Global Network Investigators. Epidemiology of stillbirth in low-middle income countries: A Global Network Study. Acta Obstet Gynecol Scand. 2011; 90:1379-85.

Mebrahtu TF, Feltbower RG, Greenwood DC, Parslow RC. Childhood body mass index and wheezing disorders: a systematic review and meta-analysis. Pediatr Allergy Immunol 2015; 26: 62–72.

Mehrotra SK, Mathur JS, Maheshwari BB. Epidemiological aspects of nutritional anaemia in children below five years. Indian J Pediatr. 1976; 43:132-5.

Mehta S, Shin H, Burnett R, North T, Cohen AJ. Ambient particulate air pollution and acute lower respiratory infections: a systematic review and implications for estimating the global burden of disease. Air Qual Atmos Health 2013; 6: 69–83.

Meshram II, Arlappa N, Balakrishna N, Laxmaiah A, Mallikarjun Rao K, Gal Reddy C, Ravindranath M, Sharad Kumar S, Brahmam GNV. Prevalence and determinants of undernutrition and its trends among pre-school tribal children of Maharashtra State, India. J Trop Pediatr. 2012; 58:125-32.

Meshram II, Arlappa N, Balakrishna N, Mallikharjuna Rao K, Laxmaiah A, Brahmam GN. Trends in the prevalence of undernutrition, nutrient & food intake and predictors of undernutrition among under five year tribal children in India. Asia Pac J Clin Nutr. 2012; 21: 568-76.

Meshram II, Balakrishna N, Arlappa N, Rao KM, Laxmaiah A, Brahmam GNV. Prevalence of undernutrition, its determinants, and seasonal variation among tribal preschool children of Odisha state, India. Asia Pac J Public Health. 2014; 26:470-80.

Meshram II, Kodavanti MR, Chitty GR, Manchala R, Kumar S, Kakani SK, Kodavalla V, Avula L, Ginnela Narsimhachary Veera B. Influence of feeding practices and associated factors on the nutritional status of infants in rural areas of Madhya Pradesh state, India. Asia Pac J Public Health. 2015; 27:NP1345-61.

Meshram II, Laxmaiah A, Venkaiah K, Brahmam GNV. Impact of feeding and breastfeeding practices on the nutritional status of infants in a district of Andhra Pradesh, India. Natl Med J India. 2012; 25:2016.

Ministry of Health and Family Welfare, Government of India, International Institute for Population Sciences, Centers for Disease Control and Prevention (CDC), Johns Hopkins Bloomberg School of Public Health, Research Triangle Institute, Inc., World Health Organization. India Global Adult Tobacco Survey 2009-2010. Atlanta, United States: CDC.

Ministry of Health and Family Welfare, Government of India, United Nations Children's Fund. India Coverage Evaluation Survey 2005.

Ministry of Health and Family Welfare, Government of India, United Nations Children's Fund. India Coverage Evaluation Survey 2007.

Ministry of Health and Family Welfare, Government of India, United Nations Children's Fund. India Coverage Evaluation Survey Data 2009-2010. [Data shared for this analysis]

Ministry of Statistics and Programme Implementation, Government of India. Common Property Resources, Sanitation & Hygiene Services, National Sample Survey Round 54, January - June 1998. New Delhi, India: Ministry of Statistics and Programme Implementation.

Ministry of Statistics and Programme Implementation, Government of India. Consumer Expenditure Survey, National Sample Survey Round 38, January-December 1983. New Delhi, India: Ministry of Statistics and Programme Implementation.

Ministry of Statistics and Programme Implementation, Government of India. Consumer Expenditure Survey, National Sample Survey Round 47, July - December 1991. New Delhi, India: Ministry of Statistics and Programme Implementation.

Ministry of Statistics and Programme Implementation, Government of India. Consumer Expenditure Survey, National Sample Survey Round 52, July 1995-June 1996. New Delhi, India: Ministry of Statistics and Programme Implementation.

Ministry of Statistics and Programme Implementation, Government of India. Consumer Expenditure Survey, India National Sample Survey Round 55, July 1999-June 2000. New Delhi, India: Ministry of Statistics and Programme Implementation.

Ministry of Statistics and Programme Implementation, Government of India. Consumer Expenditure Survey, National Sample Survey Round 60, January-June 2004. New Delhi, India: Ministry of Statistics and Programme Implementation.

Ministry of Statistics and Programme Implementation, Government of India. Consumer Expenditure Survey, National Sample Survey Round 64, July 2007- June 2008. New Delhi, India: Ministry of Statistics and Programme Implementation.

Ministry of Statistics and Programme Implementation, Government of India. Consumer Expenditure Survey, National Sample Survey Round 66, July 2009-June 2010. New Delhi, India: Ministry of Statistics and Programme Implementation.

Ministry of Statistics and Programme Implementation, Government of India. Consumer Expenditure Survey, National Sample Survey Round 68, July 2011- June 2012. New Delhi, India: Ministry of Statistics and Programme Implementation.

Ministry of Statistics and Programme Implementation, Government of India. Employment and Unemployment Survey, National Sample Survey Round 43, July 1987- June 1988. New Delhi, India: Ministry of Statistics and Programme Implementation.

Ministry of Statistics and Programme Implementation, Government of India. Employment and Unemployment Survey, National Sample Survey Round 50, July 1993- June 1994. New Delhi, India: Ministry of Statistics and Programme Implementation.

Ministry of Statistics and Programme Implementation, Government of India. Employment and Unemployment Survey, National Sample Survey Round 61, July 2004- June 2005. New Delhi, India: Ministry of Statistics and Programme Implementation.

Ministry of Statistics and Programme Implementation, Government of India. Employment and Unemployment Survey, National Sample Survey Round 64, July 2007- June 2008. New Delhi, India: Ministry of Statistics and Programme Implementation.

Ministry of Statistics and Programme Implementation, Government of India. Employment and Unemployment Survey, National Sample Survey Round 66, July 2009-June 2010. New Delhi, India: Ministry of Statistics and Programme Implementation.

Ministry of Statistics and Programme Implementation, Government of India. Employment and Unemployment Survey, National Sample Survey Round 68, July 2011- June 2012. New Delhi, India: Ministry of Statistics and Programme Implementation.

Ministry of Statistics and Programme Implementation, Government of India. Household expenditure on services and durable goods survey, National Sample Survey Round 72, July 2014 - June 2015. New Delhi, India: Ministry of Statistics and Programme Implementation.

Ministry of Statistics and Programme Implementation, Government of India. India State Series: Gross State Domestic Product at Factor Cost by Industry of Origin Tables 1980-81 to 1993-94. New Delhi, India: Ministry of Statistics and Programme Implementation.

Ministry of Statistics and Programme Implementation, Government of India. India State Series: Gross State Domestic Product at Factor Cost by Industry of Origin Tables 1993-94 to 2004-05. New Delhi, India: Ministry of Statistics and Programme Implementation.

Ministry of Statistics and Programme Implementation, Government of India. India State Series: Gross State Domestic Product at Factor Cost by Industry of Origin Tables 1999-2000 to 2009-10. New Delhi, India: Ministry of Statistics and Programme Implementation.

Ministry of Statistics and Programme Implementation, Government of India. India State Series: Gross State Domestic Product at Factor Cost by Industry of Origin Tables 2004-05 to 2013-14. New Delhi, India: Ministry of Statistics and Programme Implementation.

Ministry of Statistics and Programme Implementation, Government of India. Participation & Expenditure in Education Survey, National Sample Survey Round 64, July 2007- June 2008. New Delhi, India: Ministry of Statistics and Programme Implementation.

Ministry of Statistics and Programme Implementation, Government of India. Participation in Education Survey, National Sample Survey Round 52, July 1995-June 1996. New Delhi, India: Ministry of Statistics and Programme Implementation.

Ministry of Statistics and Programme Implementation, Government of India. Survey on Healthcare, National Sample Survey Round 52, July 1995-June 1996. New Delhi, India: Ministry of Statistics and Programme Implementation.

Ministry of Statistics and Programme Implementation, Government of India. Survey on Literacy and Culture, National Sample Survey Round 47, July - December 1991. New Delhi, India: Ministry of Statistics and Programme Implementation.

Ministry of Statistics and Programme Implementation, Government of India. Survey on Participation in Education, National Sample Survey Round 42, July 1986- June 1987. New Delhi, India: Ministry of Statistics and Programme Implementation.

Ministry of Women and Child Development, Government of India, United Nations Children's Fund. India Rapid Survey on Children 2013-2014. New Delhi, India: Ministry of Women and Child Development.

Mishra V, Retherford RD. Cooking smoke increases the risk of acute respiratory infection in children. Nat Fam Health Surv Bull.1997; 1–4.

Mishra V, Smith KR, Retherford RD. Effects of cooking smoke and environmental tobacco smoke on acute respiratory infections in young Indian children. Pop Environ. 2005; 26: 375–396.

Mitter SS, Vedanthan R, Islami F, et al. Household fuel use and cardiovascular disease mortality: Golestan cohort study. Circulation 2016; 133: 2360–9.

Mony PK, Varghese B, Thomas T. Estimation of perinatal mortality rate for institutional births in Rajasthan state, India, using capture-recapture technique. BMJ Open. 2015; 5:e005966.

Mukherjee B, Dutta A, Roychoudhury S, Ray MR. Chronic inhalation of biomass smoke is associated with DNA damage in airway cells: involvement of particulate pollutants and benzene. J Appl Toxicol 2013; 33: 281-289.
Næss Ø, Nafstad P, Aamodt G, Claussen B, Rosland P. Relation between concentration of air pollution and cause-specific mortality: four-year exposures to nitrogen dioxide and particulate matter pollutants in 470 neighbourhoods in Oslo, Norway. Am J Epidemiol 2007; 165: 435–43.
Nallam NR, Paul I, Gnanamani G. Anemia and hypoalbuminia as an adjunct to soil-transmitted helminthiasis among slum school children in Visakhapatnam, South India. Asia Pac J Clin Nutr. 1998; 7:164–9.
National Aeronautics and Space Administration (NASA), Center for International Earth Science Information Network, Columbia University. Gridded Population of the World, Version 4 (GPWv4): Population Count Adjusted to Match 2015 Revision of UN WPP Country Totals. Palisades, United States: NASA SEDAC; 2016. Available from: http://sedac.ciesin.columbia.edu/data/set/gpw-v4-population-count-adjusted-to-2015-unwpp-country-totals.
National Aeronautics and Space Administration. Aerosol Optical Depth data from Aerosol Robotic Network. Available from: https://aeronet.gsfc.nasa.gov/new_web/data.html
National Aeronautics and Space Administration. Aerosol Optical Depth data from Moderate Resolution Imaging Spectroradiometer-Dark Target Aerosol Retrieval Algorithm. Available from: https://darktarget.gsfc.nasa.gov/algorithm
National Aeronautics and Space Administration. Aerosol Optical Depth data from Moderate Resolution Imaging Spectroradiometer-Multi-Angle Implementation of Atmospheric Correction. Available from: https://ladsweb.modaps.eosdis.nasa.gov/api/v1/productGroupPage/name=maiac
National Aeronautics and Space Administration. Aerosol Optical Depth data from Moderate Resolution Imaging Spectroradiometer. Available from: https://modis.gsfc.nasa.gov/data/
National Aeronautics and Space Administration. SeaWiFS Deep Blue Aerosol Optical Depth and Angstrom Exponent Monthly Level 3 Data Gridded at 1.0 Degrees. Available from: https://data.nasa.gov/Earth-Science/SeaWiFS-Deep-Blue-Aerosol-Optical- Depth-and-Angstr/h7mm-kenx
National Nutrition Monitoring Bureau, National Institute of Nutrition (NIN), Indian Council of Medical Research. India Micronutrient Deficiency Survey Data 2003-2006. [Data shared for this analysis]
National Nutrition Monitoring Bureau, National Institute of Nutrition (NIN), Indian Council of Medical Research. India Micronutrient Deficiency Survey Report 2003-2006. Hyderabad, India: NIN.
National Nutrition Monitoring Bureau, National Institute of Nutrition (NIN), Indian Council of Medical Research. India Rural First Repeat Survey of Diet and Nutritional Status Data 1988-1990. [Data shared for this analysis]
National Nutrition Monitoring Bureau, National Institute of Nutrition (NIN), Indian Council of Medical Research. India Rural First Repeat Survey of Diet and Nutritional Status Report 1988-1990. Hyderabad, India: NIN.
National Nutrition Monitoring Bureau, National Institute of Nutrition (NIN), Indian Council of Medical Research. India Rural Second Repeat Survey of Diet and Nutritional Status Data 1996-1997. [Data shared for this analysis]
National Nutrition Monitoring Bureau, National Institute of Nutrition (NIN), Indian Council of Medical Research. India Rural Second Repeat Survey of Diet and Nutritional Status Report 1996-1997. Hyderabad, India: NIN.
National Nutrition Monitoring Bureau, National Institute of Nutrition (NIN), Indian Council of Medical Research. India Rural Survey of Diet and Nutritional Status Data 1994-1995. [Data shared for this analysis]
National Nutrition Monitoring Bureau, National Institute of Nutrition (NIN), Indian Council of Medical Research. India Rural Survey of Diet and Nutritional Status Report 1994-1995. Hyderabad, India: NIN.
National Nutrition Monitoring Bureau, National Institute of Nutrition (NIN), Indian Council of Medical Research. India Rural Survey of Diet and Nutritional Status Data 2000-2001. [Data shared for this analysis]
National Nutrition Monitoring Bureau, National Institute of Nutrition (NIN), Indian Council of Medical Research. India Rural Survey of Diet and Nutritional Status Report 2000-2001. Hyderabad, India: NIN.
National Nutrition Monitoring Bureau, National Institute of Nutrition (NIN), Indian Council of Medical Research. India Rural Survey of Diet and Nutritional Status Data 2004-2006. [Data shared for this analysis]
National Nutrition Monitoring Bureau, National Institute of Nutrition (NIN), Indian Council of Medical Research. India Rural Survey of Diet and Nutritional Status Report 2004-2006. Hyderabad, India: NIN.
National Nutrition Monitoring Bureau, National Institute of Nutrition (NIN), Indian Council of Medical Research. India Rural Third Repeat Survey of Diet and Nutritional Status Data 2011-2012. [Data shared for this analysis]
National Nutrition Monitoring Bureau, National Institute of Nutrition (NIN), Indian Council of Medical Research. India Rural Third Repeat Survey of Diet and Nutritional Status Report 2011-2012. Hyderabad, India: NIN.
National Nutrition Monitoring Bureau, National Institute of Nutrition (NIN), Indian Council of Medical Research. India Survey of Diet and Nutritional Status Data 1990-1992. [Data shared for this analysis]
National Nutrition Monitoring Bureau, National Institute of Nutrition (NIN), Indian Council of Medical Research. India Survey of Diet and Nutritional Status Report 1990-1992. Hyderabad, India: NIN.
National Nutrition Monitoring Bureau, National Institute of Nutrition (NIN), Indian Council of Medical Research. India Survey of Diet and Nutritional Status Data 1991-1992. [Data shared for this analysis]
National Nutrition Monitoring Bureau, National Institute of Nutrition (NIN), Indian Council of Medical Research. India Survey of Diet and Nutritional Status Report 1991-1992. Hyderabad, India: NIN.
National Nutrition Monitoring Bureau, National Institute of Nutrition (NIN), Indian Council of Medical Research. India Tribal First Repeat Survey of Diet and Nutritional Status Data 1998-1999. [Data shared for this analysis]

National Nutrition Monitoring Bureau, National Institute of Nutrition (NIN), Indian Council of Medical Research. India Tribal First Repeat Survey of Diet and Nutritional Status Report 1998-1999. Hyderabad, India: NIN.
National Nutrition Monitoring Bureau, National Institute of Nutrition (NIN), Indian Council of Medical Research. India Tribal Second Repeat Survey of Diet and Nutritional Status Data 2007-2008. [Data shared for this analysis]
National Nutrition Monitoring Bureau, National Institute of Nutrition (NIN), Indian Council of Medical Research. India Tribal Second Repeat Survey of Diet and Nutritional Status Report 2007-2008. Hyderabad, India: NIN.
National Nutrition Monitoring Bureau, National Institute of Nutrition (NIN), Indian Council of Medical Research. India Urban Slums Survey of Diet and Nutritional Status Data 1993-1994. [Data shared for this analysis]
National Nutrition Monitoring Bureau, National Institute of Nutrition (NIN), Indian Council of Medical Research. India Urban Slums Survey of Diet and Nutritional Status Report 1993-1994. Hyderabad, India: NIN.
National Nutrition Monitoring Bureau, National Institute of Nutrition, Indian Council of Medical Research. India National Nutrition Monitoring Bureau Eight States Pooled Data 1991-1992. [Data shared for this analysis]
Nazareth Hospital. Inpatient and Discharge Data 2014. Shillong, Meghalaya: Nazareth Hospital. [Data shared for this analysis]
Newby DE, Mannucci PM, Tell GS, et al. Expert position paper on air pollution and cardiovascular disease. Eur Heart J 2015; 36:83–93b.
Office of the Registrar General & Census Commissioner, Ministry of Home Affairs, Government of India. India Annual Health Survey Data 2010-2011. New Delhi, India: Office of the Registrar General & Census Commissioner.
Office of the Registrar General & Census Commissioner, Ministry of Home Affairs, Government of India. India Annual Health Survey Data 2010-2013. New Delhi, India: Office of the Registrar General & Census Commissioner.
Office of the Registrar General & Census Commissioner, Ministry of Home Affairs, Government of India. India Annual Health Survey Report 2010-2013. New Delhi, India: Office of the Registrar General & Census Commissioner.
Office of the Registrar General & Census Commissioner, Ministry of Home Affairs, Government of India. India Annual Health Survey Data 2011-2012. New Delhi, India: Office of the Registrar General & Census Commissioner.
Office of the Registrar General & Census Commissioner, Ministry of Home Affairs, Government of India. India Annual Health Survey Data 2012-2013. New Delhi, India: Office of the Registrar General & Census Commissioner.
Office of the Registrar General & Census Commissioner, Ministry of Home Affairs, Government of India. India Census 1981. New Delhi, India: Office of the Registrar General & Census Commissioner.
Office of the Registrar General & Census Commissioner, Ministry of Home Affairs, Government of India. India Medical Certification of Cause of Death Report 1997. New Delhi, India: Office of the Registrar General & Census Commissioner.
Office of the Registrar General & Census Commissioner, Ministry of Home Affairs, Government of India. India Medical Certification of Cause of Death Report 2005. New Delhi, India: Office of the Registrar General & Census Commissioner.
Office of the Registrar General & Census Commissioner, Ministry of Home Affairs, Government of India. India Medical Certification of Cause of Death Report 2006. New Delhi, India: Office of the Registrar General & Census Commissioner.
Office of the Registrar General & Census Commissioner, Ministry of Home Affairs, Government of India. India Medical Certification of Cause of Death Report 2008. New Delhi, India: Office of the Registrar General & Census Commissioner.
Office of the Registrar General & Census Commissioner, Ministry of Home Affairs, Government of India. India Medical Certification of Cause of Death Report 2009. New Delhi, India: Office of the Registrar General & Census Commissioner; 2014.
Office of the Registrar General & Census Commissioner, Ministry of Home Affairs, Government of India. India Medical Certification of Cause of Death Report 2010. New Delhi, India: Office of the Registrar General & Census Commissioner; 2014.
Office of the Registrar General & Census Commissioner, Ministry of Home Affairs, Government of India. India Medical Certification of Cause of Death Report 2011. New Delhi, India: Office of the Registrar General & Census Commissioner.
Office of the Registrar General & Census Commissioner, Ministry of Home Affairs, Government of India. India Medical Certification of Cause of Death Report 2012. New Delhi, India: Office of the Registrar General & Census Commissioner; 2015.
Office of the Registrar General & Census Commissioner, Ministry of Home Affairs, Government of India. India Medical Certification of Cause of Death State-Level Tabulations 1990. New Delhi, India: Office of the Registrar General & Census Commissioner.
Office of the Registrar General & Census Commissioner, Ministry of Home Affairs, Government of India. India Medical Certification of Cause of Death State-Level Tabulations 1991. New Delhi, India: Office of the Registrar General & Census Commissioner.
Office of the Registrar General & Census Commissioner, Ministry of Home Affairs, Government of India. India Medical Certification of Cause of Death State-Level Tabulations 1992. New Delhi, India: Office of the Registrar General & Census Commissioner.
Office of the Registrar General & Census Commissioner, Ministry of Home Affairs, Government of India. India Medical Certification of Cause of Death State-Level Tabulations 1993. New Delhi, India: Office of the Registrar General & Census Commissioner.
Office of the Registrar General & Census Commissioner, Ministry of Home Affairs, Government of India. India Medical Certification of Cause of Death State-Level Tabulations 1994. New Delhi, India: Office of the Registrar General & Census Commissioner.
Office of the Registrar General & Census Commissioner, Ministry of Home Affairs, Government of India. India Medical Certification of Cause of Death State-Level Tabulations 1995. New Delhi, India: Office of the Registrar General & Census Commissioner.
Office of the Registrar General & Census Commissioner, Ministry of Home Affairs, Government of India. India Medical Certification of Cause of Death State-Level Tabulations 1996. New Delhi, India: Office of the Registrar General & Census Commissioner.
Office of the Registrar General & Census Commissioner, Ministry of Home Affairs, Government of India. India Medical Certification of Cause of Death State-Level Tabulations 1998. New Delhi, India: Office of the Registrar General & Census Commissioner.

Office of the Registrar General & Census Commissioner, Ministry of Home Affairs, Government of India. India Medical Certification of Cause of Death State-Level Tabulations 1999. New Delhi, India: Office of the Registrar General & Census Commissioner.
Office of the Registrar General & Census Commissioner, Ministry of Home Affairs, Government of India. India Medical Certification of Cause of Death State-Level Tabulations 2000. New Delhi, India: Office of the Registrar General & Census Commissioner.
Office of the Registrar General & Census Commissioner, Ministry of Home Affairs, Government of India. India Medical Certification of Cause of Death State-Level Tabulations 2001. New Delhi, India: Office of the Registrar General & Census Commissioner.
Office of the Registrar General & Census Commissioner, Ministry of Home Affairs, Government of India. India Medical Certification of Cause of Death State-Level Tabulations 2002. New Delhi, India: Office of the Registrar General & Census Commissioner.
Office of the Registrar General & Census Commissioner, Ministry of Home Affairs, Government of India. India Medical Certification of Cause of Death State-Level Tabulations 2003. New Delhi, India: Office of the Registrar General & Census Commissioner.
Office of the Registrar General & Census Commissioner, Ministry of Home Affairs, Government of India. India Medical Certification of Cause of Death State-Level Tabulations 2004. New Delhi, India: Office of the Registrar General & Census Commissioner.
Office of the Registrar General & Census Commissioner, Ministry of Home Affairs, Government of India. India Population and Housing Census 1991. New Delhi, India: Office of the Registrar General & Census Commissioner.
Office of the Registrar General & Census Commissioner, Ministry of Home Affairs, Government of India. India Population and Housing Census 2001. New Delhi, India: Office of the Registrar General & Census Commissioner.
Office of the Registrar General & Census Commissioner, Ministry of Home Affairs, Government of India. India Population and Housing Census 2011. New Delhi, India: Office of the Registrar General & Census Commissioner; 2012.
Office of the Registrar General & Census Commissioner, Ministry of Home Affairs, Government of India. India Sample Registration System Age-and Sex-Specific Death Rates 1983-1995. New Delhi, India: Office of the Registrar General & Census Commissioner.
Office of the Registrar General & Census Commissioner, Ministry of Home Affairs, Government of India. India Sample Registration System Age-Specific Death Rates 1970-2006. New Delhi, India: Office of the Registrar General & Census Commissioner.
Office of the Registrar General & Census Commissioner, Ministry of Home Affairs, Government of India. India SRS Bulletin 2010. New Delhi, India: Office of the Registrar General & Census Commissioner; 2011.
Office of the Registrar General & Census Commissioner, Ministry of Home Affairs, Government of India. India SRS Compendium of Fertility and Mortality Indicators 1971-1997. New Delhi, India: Office of the Registrar General & Census Commissioner.
Office of the Registrar General & Census Commissioner, Ministry of Home Affairs, Government of India. India SRS Statistical Report 1995. New Delhi, India: Office of the Registrar General & Census Commissioner.
Office of the Registrar General & Census Commissioner, Ministry of Home Affairs, Government of India. India SRS Statistical Report 1996. New Delhi, India: Office of the Registrar General & Census Commissioner; 1998.
Office of the Registrar General & Census Commissioner, Ministry of Home Affairs, Government of India. India SRS Statistical Report 1997. New Delhi, India: Office of the Registrar General & Census Commissioner; 2001.
Office of the Registrar General & Census Commissioner, Ministry of Home Affairs, Government of India. India SRS Statistical Report 1998. New Delhi, India: Office of the Registrar General & Census Commissioner; 2000.
Office of the Registrar General & Census Commissioner, Ministry of Home Affairs, Government of India. India SRS Statistical Report 1999. New Delhi, India: Office of the Registrar General & Census Commissioner.
Office of the Registrar General & Census Commissioner, Ministry of Home Affairs, Government of India. India SRS Statistical Report 2000. New Delhi, India: Office of the Registrar General & Census Commissioner.
Office of the Registrar General & Census Commissioner, Ministry of Home Affairs, Government of India. India SRS Statistical Report 2001. New Delhi, India: Office of the Registrar General & Census Commissioner; 2005.
Office of the Registrar General & Census Commissioner, Ministry of Home Affairs, Government of India. India SRS Statistical Report 2002. New Delhi, India: Office of the Registrar General & Census Commissioner; 2005.
Office of the Registrar General & Census Commissioner, Ministry of Home Affairs, Government of India. India SRS Statistical Report 2003. New Delhi, India: Office of the Registrar General and Census Commissioner; 2005.
Office of the Registrar General & Census Commissioner, Ministry of Home Affairs, Government of India. India SRS Statistical Report 2004. New Delhi, India: Office of the Registrar General and Census Commissioner; 2006.
Office of the Registrar General & Census Commissioner, Ministry of Home Affairs, Government of India. India SRS Statistical Report 2005. New Delhi, India: Office of the Registrar General and Census Commissioner; 2006.
Office of the Registrar General & Census Commissioner, Ministry of Home Affairs, Government of India. India SRS Statistical Report 2006. New Delhi, India: Office of the Registrar General and Census Commissioner; 2008.
Office of the Registrar General & Census Commissioner, Ministry of Home Affairs, Government of India. India SRS Statistical Report 2007. New Delhi, India: Office of the Registrar General and Census Commissioner; 2008.
Office of the Registrar General & Census Commissioner, Ministry of Home Affairs, Government of India. India SRS Statistical Report 2008. New Delhi, India: Office of the Registrar General and Census Commissioner; 2010.
Office of the Registrar General & Census Commissioner, Ministry of Home Affairs, Government of India. India SRS Statistical Report 2009. New Delhi, India: Office of the Registrar General and Census Commissioner.
Office of the Registrar General & Census Commissioner, Ministry of Home Affairs, Government of India. India SRS Statistical Report 2010. New Delhi, India: Office of the Registrar General and Census Commissioner; 2012.
Office of the Registrar General & Census Commissioner, Ministry of Home Affairs, Government of India. India SRS Statistical Report 2011. New Delhi, India: Office of the Registrar General and Census Commissioner; 2013.

Office of the Registrar General & Census Commissioner, Ministry of Home Affairs, Government of India. India SRS Statistical Report 2012. New Delhi, India: Office of the Registrar General and Census Commissioner; 2013.
Office of the Registrar General & Census Commissioner, Ministry of Home Affairs, Government of India. India SRS Statistical Report 2013. New Delhi, India: Office of the Registrar General and Census Commissioner; 2014.
Office of the Registrar General & Census Commissioner, Ministry of Home Affairs, Government of India. India SRS Statistical Report 2014. New Delhi, India: Office of the Registrar General and Census Commissioner.
Office of the Registrar General & Census Commissioner, Ministry of Home Affairs, Government of India. India SRS Statistical Report 2015. New Delhi, India: Office of the Registrar General and Census Commissioner.
Office of the Registrar General & Census Commissioner, Ministry of Home Affairs, Government of India. India SRS Statistical Report 2016. New Delhi, India: Office of the Registrar General and Census Commissioner.
Office of the Registrar General & Census Commissioner, Ministry of Home Affairs, Government of India. India SRS Verbal Autopsy 2004-2006. New Delhi, India: Office of the Registrar General and Census Commissioner. [Data shared for this analysis]
Office of the Registrar General & Census Commissioner, Ministry of Home Affairs, Government of India. India SRS Verbal Autopsy Report 2004-2006. New Delhi, India: Office of the Registrar General and Census Commissioner.
Office of the Registrar General & Census Commissioner, Ministry of Home Affairs, Government of India. India SRS Verbal Autopsy 2007-2009. New Delhi, India: Office of the Registrar General and Census Commissioner. [Data shared for this analysis]
Office of the Registrar General & Census Commissioner, Ministry of Home Affairs, Government of India. India SRS Verbal Autopsy Report 2007-2009. New Delhi, India: Office of the Registrar General and Census Commissioner.
Office of the Registrar General & Census Commissioner, Ministry of Home Affairs, Government of India. India SRS Verbal Autopsy 2010-2013. New Delhi, India: Office of the Registrar General and Census Commissioner. [Data shared for this analysis]
Office of the Registrar General & Census Commissioner, Ministry of Home Affairs, Government of India. India SRS Verbal Autopsy Report 2010-2013. New Delhi, India: Office of the Registrar General and Census Commissioner.
Office of the Registrar General & Census Commissioner, Ministry of Home Affairs, Government of India. India Vital Statistics 1980. New Delhi, India: Office of the Registrar General and Census Commissioner.
Office of the Registrar General & Census Commissioner, Ministry of Home Affairs, Government of India. India Vital Statistics 1981. New Delhi, India: Office of the Registrar General and Census Commissioner.
Office of the Registrar General & Census Commissioner, Ministry of Home Affairs, Government of India. India Vital Statistics 1982. New Delhi, India: Office of the Registrar General and Census Commissioner.
Office of the Registrar General & Census Commissioner, Ministry of Home Affairs, Government of India. India Vital Statistics 1983. New Delhi, India: Office of the Registrar General and Census Commissioner.
Office of the Registrar General & Census Commissioner, Ministry of Home Affairs, Government of India. India Vital Statistics 1984. New Delhi, India: Office of the Registrar General and Census Commissioner.
Office of the Registrar General & Census Commissioner, Ministry of Home Affairs, Government of India. India Vital Statistics 1986. New Delhi, India: Office of the Registrar General and Census Commissioner.
Office of the Registrar General & Census Commissioner, Ministry of Home Affairs, Government of India. India Vital Statistics 1987. New Delhi, India: Office of the Registrar General and Census Commissioner.
Office of the Registrar General & Census Commissioner, Ministry of Home Affairs, Government of India. India Vital Statistics 1989. New Delhi, India: Office of the Registrar General and Census Commissioner.
Office of the Registrar General & Census Commissioner, Ministry of Home Affairs, Government of India. India Vital Statistics 2009. New Delhi, India: Office of the Registrar General and Census Commissioner; 2013.
Office of the Registrar General & Census Commissioner, Ministry of Home Affairs, Government of India. India Vital Statistics 2010. New Delhi, India: Office of the Registrar General and Census Commissioner; 2013.
Office of the Registrar General & Census Commissioner, Ministry of Home Affairs, Government of India. India Vital Statistics 2011. New Delhi, India: Office of the Registrar General and Census Commissioner; 2014.
Office of the Registrar General & Census Commissioner, Ministry of Home Affairs, Government of India. India Vital Statistics 2012. New Delhi, India: Office of the Registrar General and Census Commissioner; 2015.
Office of the Registrar General & Census Commissioner, Ministry of Home Affairs, Government of India. India Vital Statistics 2013. New Delhi, India: Office of the Registrar General and Census Commissioner; 2015.
Office of the Registrar General & Census Commissioner, Ministry of Home Affairs, Government of India. India Vital Statistics 2014. New Delhi, India: Office of the Registrar General and Census Commissioner.
Office of the Registrar General & Census Commissioner, Ministry of Home Affairs, Government of India. India Vital Statistics 2015. New Delhi, India: Office of the Registrar General and Census Commissioner; 2017.
Office of the Registrar General and Census Commissioner, Ministry of Health and Family Welfare, Government of India, National Institute of Health and Family Welfare, Nutrition Foundation of India, National Institute of Nutrition, Indian Council of Medical Research. India Clinical, Anthropometric and Bio-chemical (CAB) Survey Data 2014 [Biomarker Component of Annual Health Survey]. New Delhi, India: Office of the Registrar General and Census Commissioner.
Office of the Registrar General and Census Commissioner, Ministry of Health and Family Welfare, Government of India, National Institute of Health and Family Welfare, Nutrition Foundation of India, National Institute of Nutrition, Indian Council of Medical Research. India Clinical, Anthropometric and Bio-chemical (CAB) Survey Report 2014 [Biomarker Component of Annual Health Survey]. New Delhi, India: Office of the Registrar General and Census Commissioner.

Olofin I, McDonald CM, Ezzati M, Flaxman S, Black RE, Fawzi WW, Caulfield LE, Danaei G; Nutrition Impact Model Study (anthropometry cohort pooling). Associations of suboptimal growth with all-cause and cause-specific mortality in children under five years: a pooled analysis of ten prospective studies. PLoS One. 2013; 8:e64636.

Panigrahi S, Patra PK, Khodiar PK. Neonatal screening of sickle cell anemia: a preliminary report. Indian J Pediatr. 2012; 79:747–50.

Parashar A, Gupta A, Chauhan T, Barall D, Kumar D, Kumar A, Kumar S, Sharma GA, Kumari S. Verbal Autopsy to Determine the Timing and Causes of Infant Deaths in the Northern State of India. Curr Pediatr Res. 2017; 21(1): 5

Parikha J, Balakrishnanb K, Laxmia V, Biswasa H. Exposure from cooking with biofuels: pollution monitoring and analysis for rural Tamil Nadu, India. Energy. 2001; 26: 949–62.

Parker JD, Kravets N, Vaidyanathan A. Particulate matter air pollution exposure and heart disease mortality risks by race and ethnicity in the United States. Circulation 2018; 137: 1688–97.

Pasricha S-R, Black J, Muthayya S, Shet A, Bhat V, Nagaraj S, Prashanth NS, Sudarshan H, Biggs B-A, Shet AS. Determinants of anemia among young children in rural India. Pediatrics. 2010; 126:e140-9.

Pesaresi M, Ehrlich D, Ferri S, Florczyk A.J, Freire S, Halkiaet M, et al. Operating procedure for the production of the Global Human Settlement Layer from Landsat data of the epochs 1975, 1990, 2000, and 2014; JRC Technical Report EUR 27741 EN; 2016.

Pinault L, Tjepkema M, Crouse DL, et al. Risk estimates of mortality attributed to low concentrations of ambient fine particulate matter in the Canadian community health survey cohort. Environ Health 2016; 15: 18.

Prasad KN, Rao RS, Sujatha A. Birth weight pattern in Karnataka. Indian Pediatr. 1994; 31: 836-9.

Prinja S, Thakur JS, Bhatia SS. Pilot testing of WHO child growth standards in Chandigarh: implications for India's child health programmes. Bull World Health Organ. 2009; 87: 116-22.

Public Health Foundation of India. Cause of Death Estimation Study in Bihar 2011-2014. New Delhi, India: Public Health Foundation of India. [Data shared for this analysis]

Qureshi K. Domestic smoke pollution and prevalence of chronic bronchitis/asthma in a rural area of Kashmir. Indian J Chest Diseases Allied Sci 1994; 36: 61–72.

Raaschou-Nielsen O, Andersen ZJ, Beelen R, et al. Air pollution and lung cancer incidence in 17 European cohorts: prospective analyses from the European Study of Cohorts for Air Pollution Effects (ESCAPE). Lancet Oncol 2013; 14: 813–22.

Raaschou-Nielsen O, Beelen R, Wang M, et al. Particulate matter air pollution components and risk for lung cancer. Environ Int 2016; 87: 66–73.

Rahmathullah L, Tielsch JM, Thulasiraj RD, Katz J, Coles C, Devi S, John R, Prakash K, Sadanand AV, Edwin N, Kamaraj C. Impact of supplementing newborn infants with vitamin A on early infant mortality: community based randomised trial in southern India. BMJ. 2003; 327:254.

Rahmathullah L, Underwood BA, Thulasiraj RD, Milton RC, Ramaswamy K, Rahmathullah R, Babu G. Reduced mortality among children in southern India receiving a small weekly dose of vitamin A. N Engl J Med. 1990; 323:929-35.

Rai B, Pal R, Kar S, Tsering DC. Solar disinfection improves drinking water quality to prevent diarrhea in under-five children in Sikkim, India. J Glob Infect Dis. 2010; 2: 221-5

Rai S, Mahapatra B, Sircar S, Raj PY, Venkatesh S, Shaukat M, Rewari BB. Adherence to antiretroviral therapy and its effect on survival of HIV-infected individuals in Jharkhand, India. PLoS One. 2013; 8: e66860

Rai SK, Kant S, Srivastava R, Gupta P, Misra P, Pandav CS, Singh AK. Causes of and contributors to infant mortality in a rural community of North India: evidence from verbal and social autopsy. BMJ Open. 2017; 7(8): e012856.

Ramakrishnan U, Latham MC, Abel R, Frongillo EA Jr. Vitamin A supplementation and morbidity among preschool children in south India. Am J Clin Nutr. 1995; 61:1295-303.

Randomised trial to assess benefits and safety of vitamin A supplementation linked to immunisation in early infancy. WHO/CHD Immunisation-Linked Vitamin A Supplementation Study Group.Lancet. 1998; 352: 1257-63.

Rao KV, Radhaiah G, Raju SV. Association of growth status and the prevalence of anaemia in preschool children. Indian J Med Res. 1980;71: 237-46.

Reddaiah VP, Kapoor SK. Socio-biological factors in underfive deaths in a rural area. Indian J Pediatr. 1992; 59 567-71.

Rose A. Solar disinfection of water for diarrhoeal prevention in southern India. Arch Dis Child Fetal Neonatal Ed. 2006; 91: 139-41

Rusia U, Madan N, Agarwal N, Sikka M, Sood SK. Effect of maternal iron deficiency anaemia on foetal outcome. Indian J Pathol Microbiol. 1995; 38:273-9.

Sachdev HPS, Osmond C, Fall CHD, Lakshmy R, Ramji S, Dey Biswas SK, Prabhakaran D, Tandon N, Reddy KS, Barker DJP, Bhargava SK. Predicting adult metabolic syndrome from childhood body mass index: follow-up of the New Delhi birth cohort. Arch Dis Child. 2009; 94:768-74.

Sachdeva S, Alam S, Beig FK, Khan Z, Khalique N. Determinants of vitamin A deficiency amongst children in Aligarh District, Uttar Pradesh. Indian Pediatr. 2011; 48:861-6.

Saleem S, McClure EM, Goudar SS, Patel A, Esamai F, Garces A, et al. A prospective study of maternal, fetal and neonatal deaths in low- and middle-income countries. Bull World Health Organ. 2014 Aug 1;92: 605–12.

Sapkota A, Gajalakshmi V, Jetly D, Roychowdhury S, Dikshit R. Indoor air pollution from solid fuels and risk of hypopharyngeal/laryngeal and lung cancers: A multicentric case-control study from India. Int J Epidemiol 2008; 37: 321–328.

Saxena N, Nayar D, Kapil U. Prevalence of underweight, stunting and wasting. Indian Pediatr. 1997; 34:627-31.
Schultz MG, Schröder S, Lyapina O, et al. Tropospheric Ozone Assessment Report: database and metrics data of global surface ozone observations. Elem Sci Anth 2017; 5: 58.
Scott BE, Curtis V, Rabie T. What motivates handwashing in Kerala? A re-analysis of the formative research data. Mumbai, India: Indian Market Research Bureau; 2003.
Scott W, Mathew NT. A Development Monitoring Service at the Local Level: Monitoring Change in Kerala: The First Five Years. Geneva, Switzerland: United Nations Research Institute for Social Development; 1985.
Sehgal A, Telang S, Passah SM, Jyothi MC. Maternal and Neonatal Profile and Immediate outcome in extremely low birth weight babies in Delhi. Trop Doct. 2004; 34:165-8.
Sethi V, Goindi G, Kapil U. Prevalence of anemia amongst primary school age children (6-11 years) in National Capital Territory of Delhi. Indian J Pediatr. 2003; 70:519-20.
Shaddick G, Thomas ML, Jobling A, Brauer M, van Donkelaar A, Burnett R, et al. Data integration model for air quality: a hierarchical approach to the global estimation of exposures to ambient air pollution;2016. http://arxiv.org/abs/1609.00141.
Shah SN, Bakash A, Rauf A, Muzzafar A, Zuthshi ML. Incidence of iron deficiency anaemia in rural population of Kashmir. Indian J Public Health. 1982; 26:144-54.
Shah BD, Dwivedi LK. Causes of neonatal deaths among tribal women in Gujarat, India. Popul Res Policy Rev. 2011; 30: 517-36.
Shah MS, Khalique N, Khan Z. Determinants of childhood mortality. Indian J Prev Soc Med. 2011; 42: 118-22.
Shikha B, Harsh S, Narayan G. Infant deaths' audit: Contextual factors contributing to Infant deaths in tribal district-Valsad, Gujarat (India). J Res Med Den Sci. 2015; 3: 171-5.
Sidhu S, Kumari K, Uppal M. Prevalence of anemia in schedule caste preschool children of Punjab. Indian J Med Sci. 2002; 56:218-21.
Sidhu S. Incidence of anaemia among scheduled caste pre-school children of Punjab. Indian J Matern Child Health. 1996; 7:76-7.
Singhal PK, Mathur GP, Mathur S, Singh YD. Mortality patterns in under six children in I.C.D.S. urban slum. Indian Pediatr. 1986; 23: 617-22.
Singhal PK, Mathur GP, Mathur S, Singh YD. Neonatal morbidity and mortality in ICDS urban Slums. Indian Pediatr. 1990; 27: 485-8.
Singla PN, Agarwal KN. Studies on normal hemoglobin and hematocrit values in healthy children based on hematinic supplementation. Indian Pediatr. 1981; 18:821-5.
Smith KR, Bruce N, Balakrishnan K, et al. Millions dead: how do we know and what does it mean? Methods used in the comparative risk assessment of household air pollution. Annu Rev Public Health 2014; 35: 185–206.
Smith KR, McCracken JP, Weber MW, et al. Effect of reduction in household air pollution on childhood pneumonia in Guatemala (RESPIRE): a randomised controlled trial. Lancet 2011; 378: 1717–26.
Spector JM, Agrawal P, Kodkany B, Lipsitz S, Lashoher A, Dziekan G, Bahl R, Merialdi M, Mathai M, Lemer C, Gawande A. Improving Quality of Care for Maternal and Newborn Health: Prospective Pilot Study of the WHO Safe Childbirth Checklist Program. PLoS One. 2012; 7:e35151.
State Bureau of Health Intelligence & Vital Statistics, Directorate of Health Services, Department of Health & Family Welfare, Government of Odisha. India - Odisha Medical Certification of Cause of Death Data 2009. Odisha, India: Department of Health & Family Welfare, Government of Odisha.
State Bureau of Health Intelligence & Vital Statistics, Directorate of Health Services, Department of Health & Family Welfare, Government of Odisha. India - Odisha Medical Certification of Cause of Death Data 2010. Odisha, India: Department of Health & Family Welfare, Government of Odisha
State Bureau of Health Intelligence & Vital Statistics, Directorate of Health Services, Department of Health & Family Welfare, Government of Odisha. India - Odisha Medical Certification of Cause of Death Data 2011. Odisha, India: Department of Health & Family Welfare, Government of Odisha.
State Bureau of Health Intelligence & Vital Statistics, Directorate of Health Services, Department of Health & Family Welfare, Government of Odisha. India - Odisha Medical Certification of Cause of Death Data 2012. Odisha, India: Department of Health & Family Welfare, Government of Odisha.
State Bureau of Health Intelligence & Vital Statistics, Directorate of Health Services, Department of Health & Family Welfare, Government of Odisha. India - Odisha Medical Certification of Cause of Death Data 2013. Odisha, India: Department of Health & Family Welfare, Government of Odisha.
Straughn HK, Goldenberg RL, Tolosa JE, Daly S, de Codes J, Festin MR, Limpongsanurak S, Lumbiganon P, Paul VK, Peedicayil A, Purwar M, Sabogal JC, Shenoy S. Birthweight-specific neonatal mortality in developing countries and obstetric practices. Int J Gynaecol Obstet. 2003; 80:71-8.
Swain S, Singh S, Bhatia BD, Pandey S, Krishna M. Maternal hemoglobin and serum albumin and fetal growth. Indian Pediatr. 1994; 31: 777-82.
Swaminathan M, Apte S, Someswara Rao K. Nutrition of the people of Ankola Taluk N. Kanara. Indian J Med Res. 1960; 762-74.
Talsania NJ, Lala MK. Evaluation of antenatal risk scoring in a preterm birth prevention and perinatal loss. Indian J Matern Child Health. 1994; 5:5-9.

Thurston GD, Ahn J, Cromar KR, et al. Ambient particulate matter air pollution exposure and mortality in the NIH-AARP diet and health cohort. Environ Health Perspect 2016; 124: 484–90.
Tielsch JM, Rahmathullah L, Thulasiraj RD, Katz J, Coles C, Sheeladevi S, John R, Prakash K. Newborn vitamin A dosing reduces the case fatality but not incidence of common childhood morbidities in South India. J Nutr. 2007; 137:2470-4.
Tiwari S, Hopke PK, Pipal AS, Srivastava AK, Bishta DS, Tiwari S, Singh AK, Sonie VK, Attrie SD. Intra-urban variability of particulate matter (PM2.5 and PM10) and its relationship with optical properties of aerosols over Delhi, India. Atmos Res. 2015; 166: 223-32.
Tseng E, Ho W-C, Lin M-H, Cheng T-J, Chen P-C, Lin H-H. Chronic exposure to particulate matter and risk of cardiovascular mortality: cohort study from Taiwan. BMC Public Health 2015; 15: 936.
Turner MC, Jerrett M, Pope CA, et al. Long-term ozone exposure and mortality in a large prospective study. Am J Respir Crit Care Med 2016; 193: 1134–42.
Uberoi IS, DeSweemer C, Taylor CE. A study of anemia among rural Punjabi children. Indian J Med Res. 1972; 60:793-9.
United Nations, Department of Economic and Social Affairs Statistics Division, Demographic and Social Statistics Branch. Estimates of mid-year population and vital statistics summary: 1950-1964. United Nations: Demographic Yearbook, Historical supplement; 1997.
Upadhyay RP, Rai SK, Krishnan A. Using three delays model to understand the social factors responsible for neonatal deaths in rural Haryana, India. J Trop Pediatr. 2013; 59:100-5.
Upadhyaya S, Shettyb S, Kumarc SS, Dongred A, Deshmukhe P. Institutionalizing district level infant death review: an experience from southern India. Southeast Asia J Public Health. 2012; 1: 446-56.
Vaid A, Mammen A, Primrose B, Kang G. Infant mortality in an urban slum. Indian J Pediatr. 2007; 74: 449-53
Venkatarao T, Ramakrishnan R, Nair NG, Radhakrishnan S, Sundaramoorthy L, Koya PK, Kumar SK. Effect of vitamin A supplementation to mother and infant on morbidity in infancy. Indian Pediatr. 1996; 33:279-86.
Verma M, Chhatwal J, Kaur G. Prevalence of anemia among urban school children of Punjab. Indian Pediatr. 1998; 35:1181-6.
Vijayaraghavan K, Rao DH. Diet & nutrition situation in rural India. Indian J Med Res. 1998; 108:243-53.
Vogel JP, Souza JP, Mori R, Morisaki N, Lumbiganon P, Laopaiboon M, Ortiz-Panozo E, Hernandez B, Pérez-Cuevas R, Roy M, Mittal S, Cecatti JG, Tunçalp Ö, Gülmezoglu AM; WHO Multicountry Survey on Maternal and Newborn Health Research Network. Maternal complications and perinatal mortality: findings of the World Health Organization Multicountry Survey on Maternal and Newborn Health. BJOG. 2014; 121: 76-88.
Weichenthal S, Hoppin JA, Reeves F. Obesity and cardiovascular health effects of fine particulate air pollution. Obesity (Silver Spring) 2014; 22: 1580–9.
Weichenthal S, Pinault LL, Burnett RT. Impact of oxidant gases on the relationship between outdoor fine particulate air pollution and nonaccidental, cardiovascular, and respiratory mortality. Sci Rep 2017; 7: 16401.
Weiss DJ, Nelson A, Gibson HS, Temperley W, Peedell S, Lieber A, et al. A global map of travel time to cities to assess inequalities in accessibility in 2015. Nature. 2018 18;553: 333–6.
World Bank. Survey of Living Conditions 1997-1998, Uttar Pradesh and Bihar. Washington D.C., United States: World Bank.
World Bank. World Development Indicators - Vitamin A Supplementation Coverage Rate. Washington D.C., United States: World Bank.
World Health Organization (WHO), United Nations Children's Fund (UNICEF). WHO and UNICEF Reported Administrative Data for Immunization Coverage Time Series. Available from: http://www.who.int/immunization/monitoring_surveillance/data/en/
World Health Organization (WHO). Global Database on Child Growth and Malnutrition - Historical. Geneva, Switzerland: WHO.
World Health Organization (WHO). WHO Global Database on Anemia, Nutrition Landscape Information System. Geneva, Switzerland: WHO.
World Health Organization (WHO). WHO Global Database on Child Growth and Malnutrition. Geneva, Switzerland: WHO.
World Health Organization (WHO). WHO Global Database on Vitamin A Deficiency. Geneva, Switzerland: WHO.
World Health Organization (WHO). WHO Global Survey on Maternal and Perinatal Health 2004-2008. Geneva, Switzerland: WHO.
World Health Organization (WHO). WHO Household Energy Database. Geneva, Switzerland: WHO; 2010.
World Health Organization (WHO). WHO Urban Ambient Air Pollution Database Draft 2016. Geneva, Switzerland: WHO.
Yu K, Qiu G, Chan K-H, et al. Association of solid fuel use with risk of cardiovascular and all-cause mortality in rural China. JAMA 2018; 319: 1351–61.

5. Grouping of the states of India based on SDI, 2017

State group (population in 2017)	States of India*	SDI in 2017
**Low SDI states (675 million)**	Bihar	0.43
	Madhya Pradesh	0.49
	Jharkhand	0.49
	Uttar Pradesh	0.49
	Rajasthan	0.49
	Chhattisgarh	0.51
	Odisha	0.52
	Assam	0.53
**Middle SDI states (387 million)**	Andhra Pradesh	0.54
	West Bengal	0.54
	Tripura	0.54
	Arunachal Pradesh	0.56
	Meghalaya	0.56
	Karnataka	0.57
	Telangana	0.58
	Gujarat	0.58
	Manipur	0.59
	Jammu and Kashmir†	0.59
	Haryana	0.60
**High SDI states (318 million)**	Uttarakhand	0.61
	Tamil Nadu	0.62
	Mizoram	0.62
	Maharashtra	0.62
	Punjab	0.62
	Sikkim	0.63
	Nagaland	0.63
	Himachal Pradesh	0.63
	UTs other than Delhi	0.65
	Kerala	0.66
	Delhi	0.72
	Goa	0.74

SDI=Socio-demographic Index. UTs=Union territories.
SDI as computed by GBD in 2017 as described elsewhere (*Lancet* 2018; 392: 1995-2051).
*The states are listed in increasing order of SDI in 2017.
†The state of Jammu and Kashmir was divided into two union territories in August 2019; as we are reporting findings up to 2017, we report findings for the undivided state of Jammu and Kashmir.

6. IMR in the states and districts of India in 2000, 2010 and 2017

States*/Districts	IMR (95% uncertainty interval)				
	2000	2010	2017	Annual rate of reduction, 2000-2010 (%)	Annual rate of reduction, 2010-2017 (%)
**Bihar**	**58.3 (50.0-66.4)**	**44.5 (39.4-49.7)**	**35.0 (29.8-41.6)**	**-2.71**	**-3.44**
Araria	59.1 (53.1-65.4)	45.2 (40.3-50.5)	36.5 (28.5-45.9)	-2.64	-3.01
Arwal	56.0 (49.7-62.5)	43.9 (38.8-48.9)	35.1 (27.8-44.0)	-2.40	-3.16
Aurangabad	58.4 (52.2-64.8)	46.6 (41.5-51.9)	37.2 (29.7-46.9)	-2.24	-3.16
Banka	58.4 (52.6-65.1)	46.3 (41.5-51.9)	36.8 (29.2-46.8)	-2.29	-3.23
Begusarai	54.4 (49.3-59.4)	42.1 (37.8-46.4)	33.0 (26.1-40.9)	-2.54	-3.43
Bhagalpur	58.6 (52.9-64.8)	45.4 (40.8-50.8)	36.0 (28.3-45.9)	-2.52	-3.25
Bhojpur	54.8 (49.6-59.9)	42.5 (38.4-47.1)	33.5 (26.9-41.8)	-2.50	-3.34
Buxar	55.9 (50.8-61.3)	43.2 (39.2-47.5)	34.6 (27.6-43.3)	-2.55	-3.11
Darbhanga	60.1 (54.6-65.7)	44.3 (39.8-48.9)	34.3 (27.4-43.0)	-3.00	-3.57
East Champaran	61.4 (55.5-67.6)	44.8 (40.2-49.9)	35.6 (27.9-44.6)	-3.10	-3.25
Gaya	56.8 (50.9-62.7)	46.1 (41.2-50.9)	36.2 (29.0-45.5)	-2.06	-3.40
Gopalganj	55.5 (50.2-60.7)	41.2 (37.1-45.8)	32.2 (25.2-40.4)	-2.93	-3.45
Jamui	55.7 (50.7-61.4)	45.0 (40.8-50.3)	35.6 (28.6-44.5)	-2.11	-3.30
Jehanabad	55.4 (49.7-61.1)	43.6 (39.0-48.2)	34.6 (27.7-43.3)	-2.36	-3.28
Kaimur	59.3 (53.9-65.7)	45.5 (41.1-50.3)	37.5 (29.6-47.2)	-2.62	-2.71
Katihar	67.2 (60.2-74.5)	52.4 (46.6-58.7)	40.7 (32.1-51.8)	-2.45	-3.54
Khagaria	54.6 (49.1-60.1)	41.6 (37.2-46.4)	33.8 (26.7-42.5)	-2.69	-2.93
Kishanganj	60.1 (53.5-67.3)	46.3 (41.1-52.4)	36.7 (28.5-46.1)	-2.57	-3.29
Lakhisarai	53.3 (47.4-58.9)	42.3 (37.5-47.7)	33.8 (26.7-42.8)	-2.29	-3.16
Madhepura	57.4 (51.5-63.6)	43.8 (39.0-49.0)	35.6 (28.0-44.9)	-2.67	-2.93
Madhubani	70.1 (62.8-77.2)	49.5 (43.9-54.8)	38.3 (30.4-48.6)	-3.42	-3.59
Munger	53.9 (48.9-59.1)	41.0 (36.9-45.5)	32.5 (25.8-41.0)	-2.70	-3.25
Muzaffarpur	57.4 (51.8-63.0)	43.0 (38.6-48.1)	32.9 (26.1-41.5)	-2.85	-3.75
Nalanda	54.3 (49.2-59.8)	43.6 (39.2-48.3)	33.9 (27.1-42.7)	-2.18	-3.53
Nawada	54.8 (49.5-60.5)	45.1 (40.6-50.0)	35.1 (28.2-44.3)	-1.94	-3.51
Patna	51.6 (46.9-56.0)	39.6 (35.9-43.5)	30.3 (24.5-37.8)	-2.61	-3.75
Purnia	62.9 (56.3-69.5)	49.6 (44.3-55.5)	39.6 (31.5-49.7)	-2.35	-3.14
Rohtas	58.0 (52.4-63.7)	45.2 (40.7-49.8)	35.9 (28.6-44.9)	-2.47	-3.24
Saharsa	55.2 (49.1-61.4)	42.1 (37.1-47.3)	34.0 (26.5-43.0)	-2.69	-2.99
Samastipur	56.1 (51.3-61.0)	43.0 (38.9-47.4)	33.2 (26.5-41.3)	-2.61	-3.63
Saran	53.3 (47.8-58.6)	40.7 (36.6-45.5)	31.2 (24.6-39.3)	-2.66	-3.71
Sheikhpura	54.0 (48.2-60.0)	44.2 (39.2-50.0)	34.5 (27.3-44.0)	-1.99	-3.47
Sheohar	65.6 (59.2-72.2)	47.8 (42.8-53.4)	37.1 (29.3-47.0)	-3.11	-3.55
Sitamarhi	72.8 (65.5-80.2)	51.9 (46.2-57.9)	40.9 (32.3-51.6)	-3.32	-3.37
Siwan	54.7 (49.7-60.1)	41.3 (37.3-45.9)	31.8 (25.0-39.6)	-2.78	-3.66
Supaul	60.7 (54.5-67.3)	44.4 (39.4-49.4)	35.4 (27.8-44.8)	-3.07	-3.20
Vaishali	54.9 (49.7-59.7)	42.3 (38.4-47.1)	31.9 (25.4-39.7)	-2.58	-3.95
West Champaran	60.2 (53.8-67.1)	43.5 (38.7-49.3)	34.9 (27.5-44.3)	-3.20	-3.08
**Madhya Pradesh**	**80.6 (70.7-91.3)**	**59.5 (52.7-66.8)**	**41.9 (35.8-49.6)**	**-3.03**	**-5.00**
Agar Malwa	83.4 (74.9-92.3)	68.7 (61.1-76.8)	42.1 (32.8-53.3)	-1.93	-6.75
Alirajpur	75.7 (67.7-84.3)	65.6 (58.6-73.5)	44.7 (34.4-56.5)	-1.42	-5.35
Anuppur	81.7 (73.6-90.4)	61.4 (54.6-69.0)	45.9 (35.5-57.3)	-2.82	-4.06
Ashoknagar	87.6 (78.9-97.1)	67.4 (60.3-75.0)	46.9 (36.3-58.6)	-2.58	-5.07
Balaghat	72.5 (65.4-80.9)	53.1 (47.3-59.9)	37.2 (28.9-47.6)	-3.07	-4.94
Barwani	62.8 (55.4-70.4)	53.6 (46.9-60.6)	36.9 (28.2-47.1)	-1.57	-5.19
Betul	61.8 (54.5-70.2)	44.8 (38.9-51.1)	30.3 (22.9-39.0)	-3.18	-5.41
Bhind	77.1 (70.2-84.2)	59.4 (54.0-65.1)	43.5 (34.4-54.6)	-2.57	-4.34
Bhopal	57.7 (50.6-65.4)	41.2 (35.7-47.1)	28.1 (21.3-36.6)	-3.31	-5.33
Burhanpur	63.2 (55.7-71.3)	52.0 (45.9-58.9)	36.1 (27.3-46.5)	-1.92	-5.10
Chhatarpur	108.3 (97.5-119.9)	75.5 (67.6-83.4)	56.1 (44.5-69.2)	-3.54	-4.16
Chhindwara	62.6 (55.4-70.1)	43.7 (38.3-49.3)	30.7 (23.4-39.5)	-3.53	-4.93
Damoh	111.2 (100.9-122.9)	70.3 (62.3-78.7)	57.2 (45.0-71.4)	-4.48	-2.90

Datia	81.3 (73.3-89.2)	56.9 (51.5-62.7)	43.1 (33.9-53.6)	-3.51	-3.90
Dewas	64.5 (58.1-71.5)	46.9 (41.9-52.9)	30.9 (23.5-38.9)	-3.13	-5.78
Dhar	65.5 (58.4-73.0)	51.0 (45.0-57.0)	34.8 (26.8-44.2)	-2.48	-5.32
Dindori	95.3 (86.6-104.7)	69.6 (62.5-77.8)	53.1 (41.4-66.7)	-3.09	-3.78
Guna	86.1 (77.2-95.6)	70.6 (62.8-79.0)	45.8 (35.8-57.9)	-1.97	-6.00
Gwalior	76.3 (69.4-83.5)	57.6 (52.3-63.5)	43.4 (34.2-53.9)	-2.77	-3.98
Harda	74.5 (65.7-84.2)	54.0 (47.1-61.8)	37.1 (27.7-48.2)	-3.18	-5.21
Hoshangabad	67.2 (60.7-74.3)	46.5 (41.4-52.1)	31.7 (24.3-40.3)	-3.63	-5.33
Indore	55.2 (49.4-61.3)	38.1 (33.9-42.9)	25.5 (19.7-32.4)	-3.64	-5.56
Jabalpur	93.8 (84.5-104.9)	58.8 (52.3-66.4)	45.1 (34.8-56.9)	-4.56	-3.71
Jhabua	77.5 (69.2-86.7)	64.4 (57.2-72.5)	40.8 (31.2-51.3)	-1.83	-6.33
Katni	110.3 (100.7-121.1)	73.1 (65.9-81.4)	57.6 (45.1-71.5)	-4.03	-3.34
Khandwa	73.5 (65.7-82.0)	55.7 (49.6-62.7)	38.9 (29.5-49.6)	-2.73	-4.99
Khargone	68.3 (60.3-75.8)	53.4 (47.1-59.9)	37.1 (28.5-47.1)	-2.43	-5.07
Mandla	89.4 (80.9-98.9)	62.2 (55.7-69.6)	46.3 (35.8-58.5)	-3.57	-4.12
Mandsaur	80.8 (73.2-88.8)	68.9 (61.7-76.6)	39.5 (31.2-49.5)	-1.58	-7.66
Morena	79.0 (71.5-87.0)	66.8 (60.2-73.7)	47.9 (38.0-59.7)	-1.67	-4.64
Narsinghpur	79.0 (70.9-87.5)	52.5 (46.5-58.8)	37.9 (29.3-48.1)	-4.01	-4.53
Neemuch	83.5 (76.0-91.5)	73.2 (65.8-81.1)	40.0 (32.1-50.0)	-1.30	-8.27
Niwari	90.3 (81.0-99.8)	61.0 (54.6-68.1)	45.1 (35.2-56.5)	-3.85	-4.22
Panna	118.2 (105.9-130.6)	81.3 (72.6-91.4)	61.8 (48.4-77.0)	-3.67	-3.86
Raisen	73.8 (66.4-81.5)	52.2 (46.2-58.4)	37.0 (28.2-46.6)	-3.41	-4.78
Rajgarh	81.6 (73.7-89.9)	67.1 (60.3-74.7)	42.0 (32.9-52.9)	-1.94	-6.46
Ratlam	68.5 (61.6-75.7)	54.6 (48.6-60.7)	32.9 (25.9-41.6)	-2.24	-6.99
Rewa	99.3 (90.0-108.3)	72.6 (65.6-80.0)	51.9 (40.5-65.2)	-3.08	-4.70
Sagar	97.7 (87.7-108.9)	67.5 (59.5-75.6)	49.8 (38.3-62.5)	-3.63	-4.26
Satna	106.1 (96.5-116.2)	73.8 (66.2-82.1)	53.6 (42.3-67.2)	-3.57	-4.45
Sehore	65.6 (58.8-72.8)	48.0 (42.7-53.5)	32.2 (24.6-40.9)	-3.07	-5.55
Seoni	72.2 (64.4-80.6)	49.1 (43.6-55.4)	35.5 (27.3-45.6)	-3.77	-4.53
Shahdol	95.0 (85.7-104.9)	69.6 (62.3-77.8)	54.0 (42.0-68.1)	-3.06	-3.55
Shajapur	66.2 (59.4-72.9)	51.7 (46.1-57.8)	33.2 (25.8-41.8)	-2.44	-6.12
Sheopur	100.8 (91.9-110.2)	88.1 (79.4-97.3)	59.5 (47.2-73.9)	-1.34	-5.45
Shivpuri	89.8 (81.5-98.5)	67.2 (60.7-74.0)	48.4 (38.3-59.9)	-2.85	-4.58
Sidhi	97.3 (87.3-108.0)	72.9 (65.1-81.6)	54.8 (42.8-69.0)	-2.85	-3.99
Singrauli	91.6 (80.8-103.6)	71.0 (62.0-80.8)	52.2 (40.5-65.9)	-2.52	-4.30
Tikamgarh	102.7 (93.3-112.7)	72.5 (65.2-80.1)	53.8 (42.7-66.5)	-3.42	-4.19
Ujjain	59.4 (53.1-66.4)	44.6 (39.4-50.5)	28.7 (22.2-36.4)	-2.84	-6.10
Umaria	107.3 (96.8-118.5)	75.4 (67.2-85.2)	59.0 (45.7-74.9)	-3.47	-3.43
Vidisha	84.2 (74.3-94.5)	65.9 (57.2-75.5)	46.1 (35.4-58.6)	-2.42	-4.99
**Jharkhand**	**59.9 (52.8-67.9)**	**44.5 (40.6-48.9)**	**34.3 (29.8-40.1)**	**-2.97**	**-3.73**
Bokaro	57.5 (51.7-63.2)	43.2 (39.0-47.6)	31.0 (24.9-38.8)	-2.83	-4.62
Chatra	60.2 (54.4-66.1)	44.9 (40.7-49.6)	37.1 (29.7-46.2)	-2.90	-2.69
Deoghar	58.8 (53.2-64.4)	44.0 (39.7-48.7)	34.9 (28.0-43.5)	-2.85	-3.28
Dhanbad	58.5 (52.6-64.7)	43.9 (39.3-48.6)	32.6 (25.9-40.7)	-2.84	-4.17
Dumka	61.5 (55.4-67.9)	43.1 (38.5-48.1)	35.5 (28.4-44.2)	-3.49	-2.74
East Singhbhum	47.3 (41.8-53.0)	34.7 (30.6-39.2)	24.9 (19.4-31.9)	-3.04	-4.60
Garhwa	62.3 (56.0-69.9)	46.5 (41.4-52.1)	39.1 (31.0-49.6)	-2.88	-2.45
Giridih	56.3 (50.9-62.2)	43.1 (39.0-47.8)	33.5 (26.8-41.8)	-2.64	-3.53
Godda	61.0 (55.1-67.6)	44.4 (39.6-49.7)	36.7 (29.2-46.0)	-3.13	-2.67
Gumla	72.4 (65.4-79.8)	54.5 (49.2-60.7)	40.9 (32.7-50.6)	-2.79	-4.03
Hazaribagh	57.2 (51.9-62.8)	41.6 (37.7-46.3)	32.2 (25.7-39.8)	-3.13	-3.62
Jamtara	62.7 (55.6-70.0)	46.5 (41.2-52.5)	37.3 (29.1-46.7)	-2.94	-3.13
Khunti	69.0 (61.4-77.1)	52.3 (46.4-58.2)	37.2 (29.3-46.8)	-2.72	-4.75
Koderma	52.8 (47.8-58.2)	39.1 (35.4-43.3)	31.8 (25.5-39.7)	-2.96	-2.93
Latehar	66.3 (59.8-73.2)	51.3 (46.0-56.8)	40.6 (32.6-50.6)	-2.55	-3.27
Lohardaga	70.7 (63.1-79.1)	53.8 (47.7-60.3)	40.0 (31.7-50.3)	-2.70	-4.16
Pakur	61.2 (55.2-67.4)	42.4 (37.9-47.3)	35.5 (28.5-44.2)	-3.60	-2.50
Palamu	62.6 (56.7-69.1)	47.3 (42.7-52.4)	38.9 (31.4-48.9)	-2.77	-2.75

Ramgarh	57.7 (52.0-63.6)	42.0 (37.9-46.7)	29.6 (23.7-37.0)	-3.13	-4.87
Ranchi	62.8 (56.2-69.7)	46.3 (41.5-51.2)	32.8 (26.0-41.2)	-3.00	-4.82
Sahebganj	62.9 (57.0-69.0)	44.8 (40.4-49.9)	36.7 (29.1-46.1)	-3.34	-2.80
Saraikela Kharsawan	56.5 (50.7-63.0)	42.3 (37.5-47.3)	30.0 (23.5-38.0)	-2.86	-4.79
Simdega	72.0 (65.1-79.7)	52.2 (46.8-58.1)	40.1 (32.3-50.0)	-3.16	-3.69
West Singhbhum	66.3 (60.0-73.7)	51.7 (46.6-57.3)	38.5 (30.6-48.2)	-2.46	-4.13
**Uttar Pradesh**	**82.7 (71.7-94.6)**	**65.8 (59.1-73.1)**	**51.5 (44.5-60.5)**	**-2.28**	**-3.50**
Agra	76.2 (69.8-83.1)	65.2 (59.0-70.8)	49.8 (39.9-60.9)	-1.55	-3.78
Aligarh	82.6 (75.4-89.6)	73.2 (66.6-80.0)	57.6 (46.6-70.7)	-1.19	-3.38
Allahabad	95.0 (86.1-104.0)	70.5 (63.4-77.8)	56.1 (43.5-71.1)	-2.94	-3.20
Ambedkar Nagar	70.4 (63.7-77.2)	49.8 (44.6-55.6)	39.5 (31.4-49.6)	-3.41	-3.24
Amethi	85.3 (76.4-95.5)	63.1 (55.8-70.6)	51.8 (41.4-66.5)	-2.97	-2.79
Amroha	79.4 (71.2-87.8)	72.5 (64.4-80.2)	54.6 (44.1-67.5)	-0.91	-3.98
Auraiya	80.1 (73.0-87.0)	60.8 (55.2-67.2)	47.5 (38.1-58.8)	-2.71	-3.47
Azamgarh	71.5 (65.0-77.8)	54.1 (49.2-59.7)	40.0 (31.6-49.5)	-2.73	-4.22
Baghpat	66.7 (59.8-73.6)	54.4 (48.8-60.7)	42.5 (34.1-52.3)	-2.02	-3.45
Bahraich	102.6 (94.0-112.9)	82.7 (75.2-90.8)	69.6 (56.6-86.4)	-2.14	-2.43
Ballia	65.4 (59.9-71.5)	51.2 (46.6-56.3)	37.1 (29.4-46.4)	-2.42	-4.51
Balrampur	102.2 (93.3-112.9)	77.4 (70.0-85.7)	64.8 (52.0-81.9)	-2.74	-2.52
Banda	93.4 (84.8-102.4)	68.1 (61.1-75.4)	55.0 (43.3-68.7)	-3.11	-3.01
Barabanki	87.3 (79.7-95.8)	69.3 (62.8-76.6)	56.4 (45.3-71.1)	-2.28	-2.89
Bareilly	89.9 (81.3-98.5)	81.0 (73.2-89.1)	61.4 (50.0-75.1)	-1.03	-3.89
Basti	81.3 (74.9-88.4)	57.3 (52.1-62.8)	47.4 (37.9-60.2)	-3.45	-2.66
Bhadohi	89.3 (81.3-97.6)	67.4 (60.8-74.2)	52.2 (41.3-65.3)	-2.77	-3.58
Bijnor	71.4 (65.0-78.5)	63.3 (56.7-69.9)	47.6 (38.7-58.3)	-1.19	-3.99
Budaun	99.4 (90.1-108.1)	96.3 (87.0-105.1)	75.9 (62.1-92.3)	-0.31	-3.34
Bulandshahr	80.6 (73.8-87.6)	71.2 (64.1-77.7)	55.2 (44.7-67.8)	-1.23	-3.58
Chandauli	76.1 (69.7-83.5)	59.0 (53.6-65.0)	44.6 (35.4-55.9)	-2.50	-3.94
Chitrakoot	98.4 (89.5-108.1)	72.3 (64.5-80.0)	59.2 (46.3-74.4)	-3.03	-2.82
Deoria	67.7 (61.8-73.7)	50.0 (45.3-54.9)	36.0 (28.4-45.0)	-2.99	-4.56
Etah	94.4 (85.6-103.2)	82.2 (74.4-90.1)	64.9 (52.5-80.5)	-1.38	-3.31
Etawah	84.7 (77.4-92.2)	66.1 (60.1-72.5)	51.0 (40.8-63.1)	-2.44	-3.65
Faizabad	82.6 (75.1-90.5)	59.7 (53.6-66.5)	48.8 (39.1-61.7)	-3.18	-2.84
Farrukhabad	93.4 (84.9-101.8)	81.5 (73.7-89.4)	64.3 (53.1-79.4)	-1.35	-3.32
Fatehpur	87.1 (79.1-95.8)	64.4 (58.0-71.8)	50.9 (40.4-63.7)	-2.97	-3.31
Firozabad	89.3 (81.2-97.6)	75.6 (68.3-82.7)	59.2 (47.3-73.2)	-1.65	-3.44
Gautam Buddha Nagar	61.0 (55.7-66.4)	51.2 (46.2-56.1)	39.1 (31.7-48.2)	-1.74	-3.76
Ghaziabad	60.5 (54.8-66.4)	50.1 (45.1-55.6)	38.2 (30.7-47.3)	-1.86	-3.80
Ghazipur	72.4 (65.5-79.7)	57.0 (51.4-63.0)	42.2 (33.2-53.0)	-2.37	-4.20
Gonda	94.7 (86.8-104.1)	72.5 (65.8-79.9)	60.8 (48.6-77.2)	-2.63	-2.48
Gorakhpur	70.5 (64.4-77.7)	51.7 (46.5-57.2)	38.3 (29.7-48.6)	-3.06	-4.20
Hamirpur	82.5 (74.7-90.6)	56.9 (51.3-63.0)	46.1 (36.4-57.2)	-3.64	-2.96
Hapur	76.0 (69.3-82.9)	65.2 (58.6-71.4)	49.4 (39.8-61.4)	-1.52	-3.90
Hardoi	93.8 (85.6-102.3)	79.9 (72.6-88.0)	64.1 (52.6-79.1)	-1.59	-3.11
Hathras	86.4 (79.2-93.9)	72.9 (66.2-79.7)	57.5 (46.3-70.7)	-1.69	-3.33
Jalaun	74.4 (67.0-82.5)	52.0 (46.4-58.3)	43.3 (33.8-54.5)	-3.52	-2.57
Jaunpur	81.2 (74.0-88.2)	61.3 (55.6-67.2)	47.2 (37.6-58.7)	-2.77	-3.66
Jhansi	82.5 (74.7-90.7)	55.4 (50.0-61.2)	47.9 (37.8-60.1)	-3.90	-2.05
Kannauj	86.3 (78.3-94.8)	70.4 (63.3-78.6)	55.2 (44.9-68.7)	-2.02	-3.43
Kanpur Dehat	78.1 (71.1-85.5)	55.9 (50.8-61.6)	44.2 (35.3-55.1)	-3.29	-3.30
Kanpur Nagar	73.6 (66.4-81.1)	52.8 (47.8-59.0)	39.8 (31.5-49.5)	-3.26	-3.96
Kasganj	95.7 (87.4-104.4)	89.8 (81.5-98.3)	71.3 (57.9-88.1)	-0.64	-3.24
Kaushambi	96.1 (87.6-104.3)	73.5 (66.4-80.9)	59.3 (46.9-74.8)	-2.65	-3.01
Khushinagar	71.7 (65.1-79.2)	54.1 (48.8-60.7)	39.7 (30.9-50.5)	-2.78	-4.33
Lakhimpur Kheri	107.0 (96.4-117.5)	80.6 (72.5-89.9)	64.6 (52.3-80.6)	-2.79	-3.11
Lalitpur	92.2 (83.1-101.7)	64.6 (58.0-71.1)	55.9 (43.8-69.3)	-3.50	-2.03
Lucknow	75.3 (68.2-83.3)	55.8 (50.0-62.1)	42.9 (34.3-54.0)	-2.95	-3.68
Maharajganj	80.2 (72.8-88.5)	58.0 (52.0-64.6)	44.7 (34.7-56.9)	-3.20	-3.65

Mahoba	92.3 (83.4-101.3)	62.9 (56.5-69.3)	52.5 (41.5-65.0)	-3.77	-2.55
Mainpuri	90.9 (82.6-99.0)	76.0 (68.8-83.4)	58.4 (47.0-72.1)	-1.77	-3.70
Mathura	75.7 (69.1-83.0)	65.7 (59.2-71.6)	51.4 (41.2-63.4)	-1.41	-3.43
Mau	69.7 (63.7-76.0)	53.0 (48.1-58.6)	37.7 (29.8-47.3)	-2.71	-4.75
Meerut	71.0 (64.5-77.8)	58.1 (52.3-64.3)	45.1 (36.7-55.6)	-1.98	-3.57
Mirzapur	86.9 (79.2-95.0)	67.0 (61.0-73.3)	53.7 (42.8-67.0)	-2.56	-3.12
Moradabad	80.8 (72.8-89.4)	76.9 (68.6-84.8)	56.3 (45.5-69.6)	-0.49	-4.35
Muzaffarnagar	72.3 (65.9-78.9)	60.3 (53.9-66.8)	46.6 (38.0-57.1)	-1.79	-3.61
Pilibhit	87.0 (78.6-95.5)	70.0 (63.0-76.9)	52.9 (43.4-65.0)	-2.16	-3.91
Pratapgarh	89.1 (81.2-97.0)	65.1 (59.0-71.6)	52.0 (41.1-65.7)	-3.08	-3.16
Rae Bareli	82.8 (75.4-90.7)	61.0 (55.0-67.8)	49.2 (39.4-62.0)	-3.01	-3.01
Rampur	79.1 (71.6-86.9)	73.2 (65.8-80.2)	54.1 (43.9-66.7)	-0.78	-4.24
Saharanpur	66.7 (60.1-74.4)	57.6 (50.9-64.6)	44.5 (35.5-55.1)	-1.45	-3.61
Sambhal	90.0 (81.5-99.0)	87.6 (78.7-96.1)	67.6 (54.6-83.5)	-0.27	-3.63
Sant Kabir Nagar	76.1 (69.7-83.0)	54.8 (49.7-60.4)	43.2 (34.0-54.8)	-3.23	-3.34
Shahjahanpur	102.2 (92.8-111.7)	87.3 (79.1-95.6)	67.8 (55.7-83.3)	-1.56	-3.54
Shamli	70.5 (63.9-77.3)	58.9 (52.9-65.7)	45.4 (36.6-56.2)	-1.77	-3.65
Shravasti	103.9 (94.5-114.9)	83.3 (75.2-91.5)	70.2 (56.7-88.0)	-2.19	-2.40
Siddharth Nagar	92.8 (85.6-101.7)	65.8 (59.7-72.6)	53.9 (43.3-68.2)	-3.37	-2.82
Sitapur	97.9 (89.2-107.4)	79.0 (71.0-87.4)	64.1 (52.2-79.9)	-2.12	-2.93
Sonbhadra	78.6 (71.2-87.3)	61.6 (55.5-68.0)	48.7 (38.9-60.9)	-2.41	-3.28
Sultanpur	78.7 (71.0-86.0)	55.5 (50.0-61.6)	44.8 (35.9-56.5)	-3.43	-3.02
Unnao	82.9 (75.9-90.6)	62.9 (56.9-69.2)	50.4 (40.6-62.5)	-2.72	-3.12
Varanasi	77.6 (71.2-84.7)	59.5 (54.1-65.5)	43.5 (34.6-54.6)	-2.62	-4.38
**Rajasthan**	**69.0 (61.2-77.8)**	**54.4 (48.9-60.1)**	**42.0 (36.2-49.3)**	**-2.37**	**-3.69**
Ajmer	71.7 (64.1-79.1)	54.9 (48.7-60.9)	40.4 (31.9-50.4)	-2.63	-4.28
Alwar	60.3 (54.8-66.0)	47.7 (43.0-52.5)	41.0 (32.4-50.6)	-2.32	-2.16
Banswara	78.8 (70.9-86.9)	59.8 (53.4-66.3)	42.3 (33.3-53.1)	-2.72	-4.82
Baran	89.0 (81.1-97.2)	65.4 (59.1-72.3)	50.3 (40.2-62.9)	-3.04	-3.68
Barmer	80.7 (71.2-90.1)	70.3 (61.9-79.1)	56.4 (44.6-70.1)	-1.38	-3.09
Bharatpur	71.8 (65.9-78.2)	57.6 (52.3-63.0)	48.5 (38.8-59.5)	-2.18	-2.44
Bhilwara	92.0 (83.5-100.9)	73.2 (65.9-80.3)	50.4 (40.4-61.9)	-2.26	-5.21
Bikaner	43.0 (37.7-48.3)	31.1 (27.2-35.3)	31.4 (24.1-40.6)	-3.19	0.16
Bundi	92.4 (84.4-101.3)	72.0 (64.8-79.5)	52.3 (41.4-65.6)	-2.46	-4.48
Chittorgarh	100.1 (90.8-110.5)	80.9 (72.7-89.1)	53.6 (43.0-66.3)	-2.10	-5.71
Churu	40.8 (35.9-45.6)	30.1 (26.7-33.8)	26.7 (21.1-34.0)	-2.98	-1.72
Dausa	73.6 (66.2-81.5)	57.0 (50.8-63.2)	46.0 (36.7-57.2)	-2.52	-3.02
Dholpur	75.7 (68.7-83.1)	58.5 (52.8-64.5)	50.6 (40.4-62.3)	-2.54	-2.05
Dungarpur	88.5 (78.8-98.5)	71.2 (63.2-79.8)	48.1 (37.7-60.8)	-2.16	-5.44
Hanumangarh	41.8 (37.3-46.7)	29.0 (26.0-32.8)	27.5 (21.7-34.8)	-3.59	-0.75
Jaipur	52.8 (47.6-58.1)	38.5 (34.4-42.7)	31.1 (24.7-38.4)	-3.12	-2.99
Jaisalmer	57.6 (50.2-66.0)	45.2 (39.3-51.6)	44.1 (34.2-56.3)	-2.38	-0.37
Jalore	89.2 (79.5-98.9)	78.8 (69.6-88.4)	58.0 (46.2-72.4)	-1.23	-4.28
Jhalawar	89.9 (81.3-98.8)	67.0 (60.0-74.2)	48.9 (38.6-61.9)	-2.90	-4.39
Jhunjhunu	45.0 (40.1-49.9)	32.7 (29.0-36.4)	26.7 (21.0-33.7)	-3.16	-2.83
Jodhpur	52.8 (46.4-58.9)	43.9 (39.0-49.7)	35.0 (27.3-44.1)	-1.82	-3.18
Karauli	85.4 (78.5-93.2)	68.1 (61.7-74.4)	56.3 (45.5-68.8)	-2.24	-2.69
Kota	84.9 (77.5-92.8)	63.4 (57.3-70.1)	45.2 (35.8-56.5)	-2.88	-4.73
Nagaur	43.9 (38.8-49.3)	35.0 (30.9-39.4)	28.5 (22.3-35.8)	-2.24	-2.93
Pali	86.1 (77.9-94.8)	73.2 (65.7-80.6)	50.3 (40.8-62.3)	-1.61	-5.22
Pratapgarh	75.6 (67.6-84.6)	57.8 (51.3-65.2)	40.1 (31.4-50.8)	-2.65	-5.09
Rajsamand	96.0 (86.5-105.5)	79.5 (71.3-87.4)	52.3 (41.9-64.9)	-1.87	-5.80
Sawai Madhopur	96.7 (87.8-106.3)	77.7 (70.1-86.1)	61.4 (48.6-76.8)	-2.16	-3.30
Sikar	40.0 (35.4-44.7)	29.6 (26.1-33.3)	24.5 (19.3-30.8)	-2.98	-2.65
Sirohi	91.5 (81.8-101.3)	80.1 (71.7-89.1)	55.6 (44.2-69.4)	-1.32	-5.09
Sri Ganganagar	44.4 (39.3-50.0)	29.8 (26.3-33.6)	30.9 (24.1-39.4)	-3.92	0.50
Tonk	88.0 (79.9-96.7)	68.1 (61.4-75.6)	52.4 (41.2-65.2)	-2.53	-3.68
Udaipur	99.3 (89.2-109.7)	84.3 (75.8-93.3)	55.9 (44.9-70.4)	-1.62	-5.69

**Chhattisgarh**	**72.8 (64.6-81.5)**	**57.1 (50.1-65.2)**	**41.1 (35.7-47.9)**	**-2.43**	**-4.70**
Balod	70.0 (61.8-78.7)	54.0 (47.6-61.6)	39.6 (30.8-50.2)	-2.57	-4.33
Baloda Bazar	70.4 (63.8-77.3)	56.5 (50.8-63.0)	39.5 (31.3-49.3)	-2.19	-4.97
Balrampur	78.4 (70.9-86.7)	63.8 (57.4-71.3)	44.1 (35.2-54.9)	-2.03	-5.14
Bastar	69.0 (61.1-77.4)	56.4 (50.1-63.8)	43.6 (33.7-54.9)	-2.00	-3.61
Bemetara	74.3 (66.6-83.4)	56.3 (49.4-63.9)	42.6 (32.8-54.8)	-2.75	-3.90
Bijapur	60.4 (52.2-70.4)	47.8 (41.1-55.8)	40.2 (30.8-51.5)	-2.31	-2.44
Bilaspur	73.6 (66.5-81.0)	55.8 (50.0-62.2)	41.2 (32.1-51.1)	-2.73	-4.26
Dantewada	57.9 (50.3-66.5)	48.4 (42.0-56.2)	39.6 (29.9-50.7)	-1.78	-2.83
Dhamtari	67.8 (60.4-75.6)	53.5 (47.3-60.8)	37.2 (28.6-46.8)	-2.36	-5.06
Durg	66.3 (59.1-73.9)	50.4 (44.7-56.8)	35.7 (27.5-45.0)	-2.71	-4.81
Gariaband	76.3 (68.4-84.5)	61.7 (54.6-68.9)	43.5 (33.7-54.6)	-2.09	-4.88
Janjgir-Champa	74.8 (67.1-82.6)	59.8 (53.4-66.7)	39.7 (31.3-49.7)	-2.21	-5.68
Jashpur	74.3 (66.6-82.4)	59.2 (52.3-66.6)	38.9 (30.7-49.2)	-2.26	-5.81
Kabirdham	80.6 (71.6-91.6)	59.7 (51.9-68.6)	46.6 (35.1-60.6)	-2.95	-3.48
Kondagaon	84.9 (75.1-95.2)	69.8 (61.8-79.0)	53.9 (42.2-67.4)	-1.94	-3.62
Korba	72.4 (64.8-80.4)	56.9 (50.4-63.7)	39.8 (31.2-49.8)	-2.38	-4.97
Korea	76.4 (68.7-84.9)	56.4 (49.9-63.6)	43.8 (34.4-54.8)	-2.99	-3.54
Mahasamund	71.1 (64.3-78.7)	57.6 (51.7-64.3)	38.6 (30.3-48.8)	-2.09	-5.56
Mungeli	76.1 (68.1-85.6)	57.5 (50.5-64.9)	43.7 (33.8-55.8)	-2.77	-3.83
Narayanpur	81.6 (70.8-94.0)	69.4 (59.6-80.6)	56.2 (43.1-72.8)	-1.61	-2.98
North Bastar Kanker	76.0 (66.9-86.3)	61.6 (54.3-70.0)	48.1 (37.2-61.0)	-2.08	-3.46
Raigarh	73.8 (66.7-81.0)	58.7 (53.0-65.4)	38.3 (30.5-47.9)	-2.25	-5.94
Raipur	66.6 (59.3-74.2)	52.5 (46.5-59.4)	35.7 (27.9-45.1)	-2.35	-5.35
Rajnandgaon	70.2 (62.0-78.9)	54.0 (47.5-61.8)	41.2 (31.9-52.8)	-2.59	-3.79
Sukma	72.2 (62.6-83.7)	57.1 (49.3-66.7)	46.7 (35.2-60.1)	-2.32	-2.82
Surajpur	77.9 (70.5-86.2)	59.3 (52.9-66.2)	43.4 (34.5-54.6)	-2.69	-4.38
Surguja	73.9 (67.0-81.9)	57.6 (51.2-64.1)	39.7 (31.6-49.4)	-2.47	-5.19
**Odisha**	**78.0 (69.7-86.8)**	**54.1 (46.5-62.8)**	**40.1 (34.3-47.5)**	**-3.66**	**-4.29**
Angul	89.0 (80.0-98.9)	61.1 (54.3-68.6)	44.1 (34.3-56.4)	-3.69	-4.56
Balangir	82.6 (73.7-91.9)	63.1 (56.0-70.9)	47.4 (36.9-60.7)	-2.66	-4.00
Balasore	64.5 (56.6-72.5)	41.5 (36.1-47.7)	30.5 (23.3-38.8)	-4.31	-4.33
Bargarh	74.8 (67.1-83.1)	58.0 (51.7-65.1)	41.4 (32.4-52.9)	-2.52	-4.70
Bhadrak	73.2 (65.5-81.6)	43.9 (38.4-49.6)	32.7 (25.3-41.4)	-4.99	-4.09
Boudh	88.1 (78.3-98.5)	63.5 (55.8-71.7)	46.7 (36.2-59.1)	-3.22	-4.29
Cuttack	68.6 (61.2-76.4)	39.3 (34.8-44.1)	27.6 (21.5-35.0)	-5.42	-4.94
Deogarh	87.6 (78.7-97.8)	65.5 (58.9-72.9)	46.8 (36.6-58.9)	-2.87	-4.70
Dhenkanal	84.1 (75.4-93.8)	51.9 (46.1-58.2)	37.6 (29.0-47.3)	-4.71	-4.52
Gajapati	87.7 (77.3-98.2)	61.8 (53.9-70.5)	48.7 (37.1-62.2)	-3.43	-3.36
Ganjam	89.7 (78.3-101.2)	58.1 (50.3-67.2)	43.5 (33.0-55.6)	-4.25	-4.06
Jagatsinghapur	62.8 (56.0-70.7)	35.1 (30.5-40.0)	24.9 (18.8-31.9)	-5.66	-4.77
Jajapur	81.4 (73.1-90.4)	48.2 (42.6-54.1)	34.9 (27.1-44.0)	-5.11	-4.50
Jharsuguda	70.0 (62.1-78.6)	55.2 (48.9-62.2)	37.6 (29.3-47.7)	-2.35	-5.35
Kalahandi	94.2 (84.6-104.8)	73.8 (65.4-82.6)	58.3 (45.0-73.3)	-2.42	-3.30
Kandhamal	94.0 (84.0-104.8)	67.4 (59.3-76.3)	51.3 (39.8-64.7)	-3.27	-3.83
Kendrapara	70.5 (62.1-80.1)	40.6 (35.0-46.5)	29.6 (22.4-37.9)	-5.36	-4.41
Kenduajhar	84.6 (76.0-94.1)	63.6 (56.9-71.2)	44.7 (35.1-55.9)	-2.82	-4.91
Khordha	62.0 (54.3-70.1)	35.9 (31.1-40.8)	24.6 (18.7-31.2)	-5.31	-5.29
Koraput	90.0 (80.2-100.9)	70.8 (62.3-80.0)	59.3 (45.5-75.2)	-2.37	-2.51
Malkangiri	81.2 (71.2-93.0)	64.1 (55.9-73.5)	57.2 (43.5-73.6)	-2.34	-1.59
Mayurbhanj	66.8 (59.7-74.1)	51.1 (45.4-57.0)	36.3 (28.5-45.9)	-2.65	-4.75
Nabarangpur	85.2 (76.8-94.2)	68.3 (60.6-76.4)	56.8 (44.3-71.3)	-2.19	-2.60
Nayagarh	75.9 (67.9-84.8)	46.7 (41.1-52.7)	34.9 (27.0-43.8)	-4.74	-4.09
Nuapada	78.6 (70.3-86.6)	63.3 (56.4-70.4)	48.8 (37.9-61.2)	-2.14	-3.66
Puri	57.6 (50.5-65.3)	33.8 (29.1-38.9)	23.6 (17.7-30.4)	-5.20	-4.99
Rayagada	101.6 (90.8-112.8)	78.2 (69.2-87.7)	63.1 (48.4-79.9)	-2.59	-3.01
Sambalpur	76.1 (68.4-84.7)	59.0 (53.0-66.0)	40.8 (31.7-52.0)	-2.51	-5.16
Sonepur	82.7 (74.0-91.9)	61.3 (55.0-68.7)	44.3 (34.1-56.5)	-2.95	-4.55

Sundargarh	80.9 (72.3-90.6)	64.1 (57.2-71.2)	43.5 (34.6-54.3)	-2.31	-5.40
**Assam**	**64.9 (57.8-72.5)**	**53.5 (45.6-61.9)**	**42.5 (36.1-50.5)**	**-1.93**	**-3.31**
Baksa	67.4 (60.4-74.0)	47.1 (42.1-52.9)	33.2 (26.4-41.4)	-3.52	-4.88
Barpeta	59.9 (53.0-66.5)	39.3 (34.8-44.6)	29.7 (23.2-37.4)	-4.13	-3.94
Biswanath	53.4 (47.0-59.8)	47.5 (42.0-53.7)	37.0 (29.2-45.9)	-1.16	-3.52
Bongaigaon	61.5 (55.1-68.2)	38.7 (34.4-43.3)	30.0 (23.8-37.9)	-4.52	-3.58
Cachar	76.6 (68.3-85.2)	75.8 (67.5-84.9)	63.9 (50.5-80.8)	-0.10	-2.42
Charaideo	57.7 (51.2-65.3)	55.6 (49.0-63.5)	48.4 (36.6-61.8)	-0.36	-1.97
Chirang	61.2 (54.3-67.7)	38.8 (34.6-43.6)	29.5 (23.6-37.3)	-4.45	-3.84
Darrang	73.7 (65.9-82.0)	59.8 (52.9-66.8)	44.0 (35.0-54.5)	-2.07	-4.29
Dhemaji	53.2 (47.0-60.4)	45.9 (40.8-51.8)	37.5 (28.9-47.1)	-1.47	-2.82
Dhubri	72.3 (64.5-80.3)	45.7 (40.7-51.2)	35.3 (28.0-45.5)	-4.47	-3.62
Dibrugarh	53.6 (47.0-60.6)	49.1 (43.3-55.6)	41.7 (31.8-52.9)	-0.88	-2.31
Dima Hasao	73.1 (65.1-81.4)	70.1 (62.6-77.9)	61.8 (49.4-76.7)	-0.41	-1.79
Goalpara	62.2 (56.1-68.3)	38.7 (34.6-43.2)	30.7 (24.7-38.7)	-4.64	-3.23
Golaghat	59.3 (52.7-66.4)	54.5 (48.7-61.5)	45.7 (36.2-57.4)	-0.83	-2.48
Hailakandi	79.0 (70.9-87.2)	77.2 (69.2-86.2)	65.7 (51.7-83.5)	-0.24	-2.27
Hojai	75.1 (66.3-84.1)	72.0 (63.4-81.1)	58.3 (45.6-73.6)	-0.42	-2.98
Jorhat	63.6 (56.3-71.6)	58.2 (51.3-66.1)	47.3 (36.5-59.9)	-0.88	-2.93
Kamrup	60.7 (54.6-66.8)	42.1 (37.4-47.2)	31.0 (24.6-38.5)	-3.60	-4.27
Kamrup Metropolitan	55.7 (49.9-61.7)	41.0 (36.6-45.9)	29.5 (23.1-36.6)	-3.00	-4.63
Karbi Anglong	64.4 (57.1-71.9)	60.5 (53.7-67.7)	50.3 (40.1-63.0)	-0.62	-2.60
Karimganj	82.8 (74.4-91.2)	76.0 (68.0-84.5)	66.4 (52.1-84.4)	-0.85	-1.91
Kokrajhar	68.7 (61.6-76.2)	43.1 (38.3-48.2)	32.8 (26.3-41.6)	-4.55	-3.82
Lakhimpur	56.8 (50.5-63.1)	49.8 (44.1-56.0)	39.2 (30.5-48.8)	-1.31	-3.34
Majuli	63.3 (56.1-71.0)	57.1 (50.5-64.5)	45.9 (35.6-57.3)	-1.03	-3.08
Morigaon	74.4 (66.6-82.1)	64.5 (57.7-71.8)	49.5 (39.7-61.4)	-1.41	-3.72
Nagaon	69.9 (62.3-77.3)	64.7 (57.8-72.1)	50.1 (40.1-62.3)	-0.77	-3.58
Nalbari	61.2 (54.7-67.9)	41.5 (36.8-47.0)	30.0 (23.7-37.5)	-3.80	-4.56
Sivasagar	60.7 (53.8-68.5)	56.2 (49.8-63.7)	47.1 (36.3-59.6)	-0.77	-2.49
Sonitpur	60.5 (53.7-67.2)	52.4 (46.8-58.4)	38.8 (31.2-47.7)	-1.43	-4.19
South Salmara Mancachar	70.7 (63.8-78.0)	43.8 (39.2-48.9)	33.8 (26.8-42.8)	-4.67	-3.65
Tinsukia	46.0 (40.1-52.3)	42.1 (36.6-48.3)	36.0 (27.3-46.7)	-0.88	-2.20
Udalguri	71.7 (63.9-81.2)	56.7 (49.9-64.1)	40.6 (32.2-51.2)	-2.33	-4.67
West Karbi Anglong	72.6 (64.0-81.1)	68.0 (59.9-76.3)	56.2 (44.0-70.9)	-0.65	-2.70
**Andhra Pradesh**	**66.7 (59.2-74.1)**	**47.2 (38.0-57.6)**	**31.2 (22.1-44.1)**	**-3.45**	**-5.90**
Anantapur	80.7 (69.9-92.8)	52.6 (45.4-62.0)	37.0 (27.4-49.2)	-4.19	-4.89
Chittoor	60.3 (51.5-69.2)	36.6 (31.3-42.8)	27.2 (19.8-36.3)	-4.86	-4.18
East Godavari	54.9 (46.5-64.7)	39.2 (33.3-46.9)	26.7 (19.6-35.7)	-3.33	-5.31
Guntur	56.0 (47.6-66.5)	42.4 (35.0-50.7)	26.6 (19.0-35.6)	-2.75	-6.45
Krishna	57.4 (49.0-67.2)	41.3 (34.6-49.2)	25.6 (18.5-34.4)	-3.23	-6.60
Kurnool	84.8 (73.8-97.4)	63.2 (54.1-73.3)	41.3 (30.4-54.5)	-2.90	-5.89
Prakasam	58.3 (49.0-68.4)	46.2 (38.4-56.1)	30.6 (22.4-41.0)	-2.28	-5.72
Sri Potti Sriramulu Nellore	57.8 (48.0-68.8)	41.1 (33.8-49.7)	29.0 (21.0-38.9)	-3.36	-4.84
Srikakulam	85.1 (74.3-97.2)	55.9 (48.0-65.1)	34.3 (25.6-44.4)	-4.11	-6.74
Visakhapatnam	68.6 (59.2-79.3)	49.0 (41.4-57.9)	32.0 (23.8-41.8)	-3.31	-5.89
Vizianagaram	93.6 (81.8-106.8)	66.6 (56.4-77.9)	41.8 (30.8-55.3)	-3.35	-6.45
West Godavari	59.5 (50.6-69.4)	42.3 (35.2-51.8)	27.9 (20.2-37.8)	-3.34	-5.77
YSR	71.4 (60.9-82.6)	51.6 (43.6-61.1)	34.9 (25.8-45.8)	-3.19	-5.45
**West Bengal**	**51.4 (45.9-57.4)**	**36.1 (29.2-44.0)**	**25.5 (22.0-29.9)**	**-3.53**	**-4.98**
Alipurduar	55.6 (49.7-62.0)	38.3 (33.8-43.1)	25.7 (20.5-32.6)	-3.66	-5.55
Bankura	50.7 (44.9-57.8)	40.2 (35.4-45.5)	28.0 (21.9-35.6)	-2.29	-5.05
Birbhum	62.3 (55.8-68.8)	47.1 (42.0-52.4)	33.3 (26.4-41.6)	-2.75	-4.83
Cooch Behar	61.7 (55.4-68.3)	42.7 (38.0-48.0)	29.6 (23.6-37.6)	-3.61	-5.12
Dakshin Dinajpur	58.2 (51.6-65.2)	41.4 (36.6-47.0)	29.7 (23.3-38.4)	-3.35	-4.65
Darjeeling	56.4 (49.8-63.2)	41.7 (36.7-47.0)	26.0 (20.4-32.9)	-2.97	-6.54
Hooghly	44.5 (39.7-50.4)	28.5 (24.9-32.4)	21.1 (16.0-27.3)	-4.34	-4.23
Howrah	39.4 (34.4-45.4)	25.2 (21.7-29.0)	18.0 (13.4-23.6)	-4.40	-4.70

Jalpaiguri	55.5 (49.8-61.6)	41.8 (37.1-46.7)	26.9 (21.5-34.2)	-2.80	-6.09
Jhargram	51.2 (45.3-58.0)	38.2 (33.4-43.3)	27.0 (20.9-34.3)	-2.90	-4.82
Kalimpong	45.5 (40.4-50.9)	34.2 (30.0-38.7)	20.4 (16.1-25.6)	-2.83	-7.10
Kolkata	36.9 (32.1-42.5)	23.7 (20.3-27.4)	16.8 (12.5-21.9)	-4.32	-4.84
Maldah	68.2 (61.4-75.3)	51.7 (46.3-58.1)	36.0 (28.5-46.2)	-2.73	-5.03
Murshidabad	62.0 (55.3-69.3)	43.8 (38.7-49.4)	31.0 (24.2-39.3)	-3.42	-4.84
Nadia	52.6 (46.0-59.3)	34.4 (29.9-39.5)	24.9 (18.9-32.0)	-4.17	-4.49
North 24 Parganas	43.6 (38.8-49.0)	27.5 (23.8-31.2)	20.1 (15.0-26.2)	-4.50	-4.40
Paschim Burdwan	53.8 (47.3-60.8)	43.8 (38.8-49.6)	28.9 (22.6-36.5)	-2.03	-5.77
Paschim Medinipur	49.0 (43.2-55.6)	33.6 (29.2-38.4)	24.5 (18.7-31.4)	-3.72	-4.39
Purba Burdwan	50.0 (43.6-57.3)	35.0 (30.2-40.3)	25.5 (19.4-32.7)	-3.50	-4.43
Purba Medinipur	47.0 (40.8-53.4)	29.8 (25.5-34.4)	21.8 (16.4-28.2)	-4.44	-4.36
Puruliya	61.1 (54.6-67.9)	52.3 (46.5-57.9)	34.0 (27.2-42.7)	-1.54	-5.95
South 24 Parganas	46.0 (40.1-52.7)	29.9 (25.4-34.8)	21.6 (16.0-28.0)	-4.22	-4.53
Uttar Dinajpur	64.2 (57.7-71.0)	50.0 (44.8-56.0)	35.1 (27.7-44.4)	-2.47	-4.94
**Tripura**	**51.9 (46.5-58.0)**	**39.3 (31.7-48.5)**	**30.1 (25.9-35.3)**	**-2.79**	**-3.84**
Dhalai	56.9 (50.5-64.1)	45.9 (40.3-52.1)	37.5 (28.8-48.1)	-2.12	-2.85
Gomati	48.0 (42.6-53.7)	37.0 (32.6-42.2)	28.0 (21.6-35.7)	-2.56	-3.91
Khowai	57.2 (51.1-63.7)	41.0 (36.4-46.2)	31.2 (24.1-40.2)	-3.27	-3.83
North Tripura	63.2 (56.7-70.4)	53.5 (47.5-60.0)	43.9 (34.1-56.8)	-1.66	-2.77
Sepahijala	45.8 (41.0-50.9)	33.0 (29.1-37.4)	24.5 (18.9-31.2)	-3.23	-4.17
South Tripura	43.3 (37.9-49.0)	33.3 (28.5-38.6)	25.6 (19.6-33.4)	-2.58	-3.72
Unakoti	59.7 (53.1-66.9)	47.5 (42.0-53.5)	38.5 (29.7-49.7)	-2.26	-2.95
West Tripura	50.2 (45.1-55.7)	35.1 (31.1-39.7)	24.6 (19.0-31.4)	-3.52	-4.95
**Arunachal Pradesh**	**50.7 (45.1-56.8)**	**30.5 (26.4-35.5)**	**21.4 (18.4-25.1)**	**-5.08**	**-5.09**
Anjaw	43.0 (33.6-54.2)	26.4 (20.7-32.9)	19.7 (13.4-27.6)	-4.78	-4.06
Changlang	52.4 (44.6-61.0)	33.5 (28.6-38.8)	24.1 (17.9-31.5)	-4.39	-4.55
Dibang Valley	34.9 (27.0-44.5)	18.6 (14.2-24.2)	13.0 (9.0-18.4)	-6.11	-4.98
East Kameng	57.3 (48.2-67.4)	29.1 (24.0-35.0)	18.3 (14.0-23.6)	-6.55	-6.41
East Siang	45.2 (39.0-52.0)	25.4 (21.9-29.7)	17.9 (13.3-23.4)	-5.60	-4.91
Kamle	52.2 (45.3-59.4)	27.7 (24.2-31.8)	17.8 (13.7-22.6)	-6.15	-6.11
Kra Daddi	48.9 (40.9-57.5)	25.2 (21.0-30.1)	16.1 (12.0-21.0)	-6.41	-6.19
Kurung Kumey	59.8 (50.0-71.2)	28.2 (23.4-34.1)	18.6 (14.1-24.1)	-7.22	-5.78
Lohit	44.5 (37.7-51.9)	26.7 (23.0-31.0)	18.7 (13.6-24.8)	-4.99	-4.92
Longding	58.7 (50.9-67.4)	39.7 (34.2-45.8)	28.9 (21.9-37.4)	-3.84	-4.42
Lower Dibang Valley	41.2 (34.8-48.1)	23.4 (19.7-28.1)	16.2 (11.8-21.5)	-5.50	-5.12
Lower Siang	54.6 (47.8-62.8)	32.5 (28.2-37.2)	22.2 (16.8-28.4)	-5.07	-5.29
Lower Subansiri	49.5 (43.0-56.1)	26.6 (23.2-30.5)	16.5 (12.6-21.0)	-6.04	-6.58
Namsai	48.6 (41.8-55.8)	29.6 (25.6-34.0)	20.6 (15.3-26.8)	-4.86	-5.05
Pakke Kessang	52.5 (45.6-60.1)	29.7 (26.0-33.9)	17.5 (13.8-22.0)	-5.54	-7.25
Papum Pare	55.4 (48.8-61.7)	31.8 (28.3-35.7)	20.0 (15.8-24.8)	-5.41	-6.37
Shi Yomi	46.1 (40.0-52.7)	24.2 (20.9-27.9)	15.4 (11.7-20.1)	-6.26	-6.22
Siang	35.5 (29.5-42.6)	17.6 (14.3-21.3)	11.8 (8.4-16.0)	-6.79	-5.57
Tawang	57.4 (49.7-65.4)	29.0 (24.8-33.7)	17.1 (13.5-21.3)	-6.60	-7.29
Tirap	60.7 (51.8-70.6)	40.0 (33.9-46.9)	27.9 (20.8-36.6)	-4.08	-5.05
Upper Siang	34.7 (27.7-42.7)	16.9 (13.3-21.7)	11.6 (8.1-16.3)	-6.92	-5.23
Upper Subansiri	45.8 (38.5-54.1)	23.0 (18.8-27.6)	15.2 (11.2-19.9)	-6.65	-5.76
West Kameng	56.2 (49.5-63.0)	28.9 (25.3-32.6)	17.1 (13.7-21.0)	-6.43	-7.25
West Siang	38.3 (30.2-47.5)	19.0 (14.8-23.5)	12.8 (8.9-17.6)	-6.78	-5.45
**Meghalaya**	**46.5 (41.0-52.2)**	**38.0 (32.5-44.0)**	**31.9 (27.3-37.4)**	**-2.01**	**-2.52**
East Garo Hills	41.2 (37.0-45.5)	30.2 (26.7-33.7)	23.9 (19.1-30.5)	-3.07	-3.27
East Jaintia Hills	60.8 (54.3-67.5)	64.1 (57.4-71.0)	60.0 (47.5-74.6)	0.54	-0.95
East Khasi Hills	44.8 (40.2-49.4)	37.0 (33.1-41.2)	30.7 (24.2-37.9)	-1.90	-2.60
North Garo Hills	42.3 (38.0-46.7)	31.0 (27.5-34.7)	25.4 (20.1-32.0)	-3.07	-2.83
Ri Bhoi	47.1 (42.6-51.8)	43.2 (38.6-47.9)	36.1 (28.4-44.1)	-0.88	-2.53
South Garo Hills	44.2 (39.7-49.0)	30.6 (27.1-34.7)	23.2 (18.3-30.0)	-3.59	-3.89
South West Garo Hills	51.5 (45.9-57.2)	35.0 (30.8-39.6)	27.5 (21.5-35.5)	-3.79	-3.39
South West Khasi Hills	44.1 (39.5-48.9)	33.6 (29.8-37.8)	29.0 (22.6-36.4)	-2.68	-2.10

West Garo Hills	46.9 (42.3-51.7)	33.6 (30.0-37.7)	26.8 (21.1-34.4)	-3.29	-3.18
West Jaintia Hills	51.4 (46.1-56.8)	49.9 (44.8-55.5)	45.4 (35.8-56.2)	-0.28	-1.36
West Khasi Hills	42.5 (38.4-46.5)	33.2 (29.6-36.9)	28.2 (22.2-35.0)	-2.44	-2.31
**Karnataka**	**53.0 (48.0-58.7)**	**37.9 (32.7-43.8)**	**27.3 (23.4-32.1)**	**-3.35**	**-4.72**
Bagalkot	65.5 (57.5-74.4)	45.9 (39.5-52.7)	31.9 (23.5-42.2)	-3.50	-5.07
Ballari	72.9 (62.9-83.4)	55.3 (47.4-63.5)	39.5 (29.2-53.1)	-2.71	-4.70
Belagavi	47.5 (41.2-54.5)	33.8 (28.8-39.2)	23.9 (17.8-31.5)	-3.34	-4.81
Bengaluru Rural	54.2 (47.0-61.6)	38.1 (33.0-44.3)	28.5 (20.7-37.9)	-3.46	-4.05
Bengaluru Urban	45.0 (38.5-51.8)	32.7 (27.8-38.4)	23.2 (16.7-31.1)	-3.14	-4.81
Bidar	42.2 (36.3-48.2)	36.6 (31.2-42.2)	24.4 (18.5-31.3)	-1.42	-5.60
Chamarajanagar	53.6 (45.3-63.2)	38.4 (32.2-45.6)	27.4 (19.4-37.0)	-3.29	-4.69
Chikballapur	54.3 (47.0-62.6)	39.7 (34.3-46.3)	30.8 (22.3-40.5)	-3.09	-3.59
Chikkamagaluru	46.3 (39.3-53.7)	29.7 (25.0-35.6)	21.6 (15.4-29.0)	-4.36	-4.44
Chitradurga	61.8 (52.9-70.7)	42.7 (36.4-49.9)	31.9 (23.2-42.2)	-3.64	-4.07
Dakshina Kannada	24.6 (20.5-29.3)	16.3 (13.2-19.7)	11.3 (7.8-15.7)	-4.04	-5.10
Davanagere	64.7 (56.1-73.8)	44.8 (38.3-52.3)	31.2 (22.7-41.0)	-3.61	-5.03
Dharwad	62.9 (54.3-72.4)	42.3 (36.2-49.5)	30.3 (22.1-41.0)	-3.87	-4.65
Gadag	78.6 (68.8-89.5)	55.0 (47.2-63.7)	39.0 (28.6-52.5)	-3.50	-4.80
Hassan	50.5 (43.1-58.9)	32.0 (26.9-37.9)	23.2 (16.4-31.4)	-4.46	-4.50
Haveri	65.6 (56.3-76.2)	45.5 (38.3-53.4)	32.4 (23.3-43.5)	-3.61	-4.72
Kalaburgi	48.8 (41.4-56.7)	39.7 (33.6-46.2)	28.5 (21.3-37.2)	-2.04	-4.61
Kodagu	33.5 (28.6-38.7)	21.8 (18.3-25.9)	16.0 (11.1-21.5)	-4.22	-4.35
Kolar	49.8 (42.4-57.8)	35.4 (30.1-41.8)	26.7 (19.4-35.9)	-3.36	-3.92
Koppal	80.2 (70.8-90.7)	59.0 (51.3-67.3)	41.2 (30.4-54.2)	-3.02	-5.01
Mandya	56.8 (49.3-65.4)	38.0 (32.3-44.5)	27.4 (19.7-36.5)	-3.94	-4.56
Mysuru	50.6 (43.5-58.4)	34.6 (29.2-40.9)	24.0 (17.0-32.3)	-3.73	-5.08
Raichur	69.0 (60.0-78.8)	53.5 (45.5-61.5)	39.0 (29.2-50.5)	-2.51	-4.42
Ramanagara	56.1 (48.1-64.3)	38.6 (33.0-45.3)	28.3 (20.3-37.9)	-3.68	-4.30
Shivamogga	45.8 (39.5-52.7)	31.0 (26.1-36.6)	22.0 (15.8-29.4)	-3.85	-4.77
Tumakuru	58.4 (51.0-66.9)	39.8 (34.6-46.6)	30.3 (22.1-39.8)	-3.78	-3.80
Udupi	28.6 (23.5-34.7)	18.9 (15.1-23.7)	13.7 (9.5-19.0)	-4.04	-4.54
Uttara Kannada	43.2 (36.1-51.2)	29.3 (24.3-35.1)	22.3 (15.7-30.6)	-3.79	-3.83
Vijaypura	53.2 (46.4-60.6)	39.4 (33.6-45.4)	28.2 (21.0-36.4)	-2.95	-4.67
Yadgir	60.4 (52.0-69.4)	46.5 (39.6-53.9)	35.0 (26.0-45.7)	-2.57	-3.97
**Telangana**	**59.0 (52.4-66.2)**	**40.1 (31.3-50.4)**	**26.0 (18.6-36.7)**	**-3.85**	**-6.23**
Adilabad	51.6 (42.6-60.8)	42.8 (35.2-51.1)	28.5 (21.1-37.9)	-1.86	-5.62
Bhadradri Kothagudem	67.9 (56.1-82.3)	47.6 (38.4-57.4)	33.1 (24.0-43.8)	-3.50	-5.05
Hyderabad	50.1 (42.7-58.2)	32.8 (27.5-39.1)	20.3 (15.0-26.8)	-4.13	-6.65
Jagitial	54.2 (46.3-63.8)	40.4 (34.4-48.0)	25.6 (18.9-33.4)	-2.88	-6.30
Jangoan	57.6 (48.9-67.5)	37.2 (31.2-44.1)	24.1 (17.8-32.0)	-4.28	-6.03
Jayashankar Bhupalpally	57.3 (46.2-70.8)	40.9 (32.5-50.5)	28.6 (21.1-38.6)	-3.31	-4.98
Jogulamba Gadwal	82.8 (70.1-96.1)	54.2 (45.3-63.7)	36.4 (27.0-48.5)	-4.15	-5.52
Kamareddy	58.3 (48.5-69.2)	41.4 (34.0-49.9)	26.0 (19.0-34.1)	-3.36	-6.43
Karimnagar	53.6 (45.4-64.0)	36.7 (30.6-43.9)	23.5 (17.6-30.9)	-3.73	-6.18
Khammam	60.8 (49.6-73.4)	42.6 (34.5-52.2)	28.1 (20.0-38.1)	-3.50	-5.76
Kumuram Bheem Asifabad	53.5 (43.8-65.9)	42.0 (33.8-51.9)	28.4 (20.8-38.4)	-2.39	-5.45
Mahabubnagar	74.3 (63.8-86.1)	46.2 (39.1-54.8)	31.5 (23.1-41.1)	-4.65	-5.30
Mahuababad	57.1 (47.9-67.2)	39.5 (32.6-47.1)	26.3 (19.2-35.2)	-3.63	-5.64
Mancherial	54.1 (45.2-65.8)	40.8 (33.5-49.9)	26.7 (19.6-35.5)	-2.77	-5.88
Medak	62.9 (52.2-74.6)	42.0 (34.4-50.7)	26.3 (19.1-34.7)	-3.95	-6.51
Medchal Malkajgiri	53.0 (45.5-61.4)	33.8 (28.5-40.1)	20.7 (15.3-27.4)	-4.38	-6.77
Nagarkurnool	74.0 (60.1-89.6)	51.0 (40.6-63.4)	33.5 (23.9-45.8)	-3.65	-5.81
Nalgonda	61.3 (50.5-73.0)	42.4 (34.7-51.9)	27.0 (19.3-36.8)	-3.61	-6.24
Nirmal	51.6 (43.4-60.9)	42.2 (35.3-49.6)	27.4 (20.4-35.7)	-2.01	-5.97
Nizamabad	54.2 (46.1-63.6)	40.7 (34.5-47.5)	25.6 (19.1-33.2)	-2.81	-6.44
Peddapalli	54.0 (45.4-64.2)	38.4 (31.7-46.2)	24.4 (18.0-32.2)	-3.35	-6.25
Rajanna Sircilla	56.6 (47.6-66.5)	39.2 (32.7-47.0)	25.0 (18.3-32.6)	-3.62	-6.22
Rangareddy	60.5 (51.1-71.1)	37.8 (31.3-44.8)	24.2 (17.9-32.3)	-4.59	-6.16

Sangareddy	62.9 (53.9-72.3)	40.7 (34.3-47.5)	25.7 (19.3-33.4)	-4.26	-6.33
Siddipet	58.4 (49.7-67.7)	38.3 (32.3-45.4)	24.6 (18.2-32.4)	-4.14	-6.13
Suryapet	57.8 (48.5-67.8)	41.3 (33.9-49.6)	26.3 (19.3-35.3)	-3.31	-6.23
Vikarabad	69.1 (59.0-80.2)	42.4 (35.4-50.0)	28.5 (21.2-37.2)	-4.76	-5.51
Wanaparthy	78.0 (65.8-93.0)	52.1 (42.7-62.5)	34.5 (25.0-46.2)	-3.96	-5.73
Warangal Rural	54.5 (45.4-65.8)	37.3 (30.2-44.7)	24.4 (17.9-32.3)	-3.71	-5.88
Warangal Urban	51.5 (43.5-61.5)	34.8 (28.5-41.3)	22.0 (16.2-29.0)	-3.84	-6.37
Yadadri Bhuvanagiri	59.8 (49.8-70.1)	38.6 (32.1-46.5)	24.7 (18.1-33.0)	-4.28	-6.19
**Gujarat**	**56.2 (50.1-62.8)**	**41.7 (36.0-48.2)**	**33.2 (28.5-39.0)**	**-3.00**	**-3.23**
Ahmedabad	54.9 (48.6-62.9)	42.7 (36.7-49.2)	31.5 (24.3-40.6)	-2.49	-4.26
Amreli	44.6 (37.3-53.6)	30.6 (24.2-37.7)	27.1 (19.6-36.9)	-3.69	-1.74
Anand	72.4 (62.7-83.5)	55.1 (46.5-64.6)	42.0 (32.1-53.7)	-2.70	-3.82
Arvalli	74.5 (66.7-83.1)	58.9 (51.7-66.4)	41.2 (32.1-52.9)	-2.33	-4.97
Banaskantha	70.3 (61.8-79.6)	57.3 (49.9-64.8)	44.7 (35.2-55.9)	-2.03	-3.46
Bharuch	57.5 (50.4-65.6)	41.7 (35.4-48.1)	34.6 (26.3-44.5)	-3.15	-2.65
Bhavnagar	49.7 (43.3-57.5)	36.6 (30.4-43.6)	32.4 (24.6-42.4)	-3.01	-1.73
Botad	53.2 (45.5-61.5)	38.0 (31.5-44.7)	33.6 (25.5-43.0)	-3.30	-1.76
Chhotaudepur	68.7 (60.9-76.8)	51.8 (45.1-58.7)	44.0 (33.6-56.0)	-2.79	-2.30
Dahod	74.3 (65.1-84.2)	55.1 (48.4-62.4)	43.1 (33.0-54.5)	-2.95	-3.46
Dang	48.3 (42.8-54.6)	35.7 (31.0-40.9)	33.1 (25.1-42.5)	-3.00	-1.08
Devbhumi Dwarka	45.4 (35.2-57.0)	29.0 (21.9-37.7)	25.7 (17.6-36.9)	-4.39	-1.69
Gandhinagar	56.7 (49.2-65.6)	44.7 (37.5-52.3)	30.8 (22.9-40.4)	-2.34	-5.19
Gir Somnath	44.1 (36.5-53.4)	28.6 (22.4-35.8)	25.2 (18.1-34.9)	-4.23	-1.80
Jamnagar	45.3 (37.7-54.6)	29.5 (24.0-35.8)	25.3 (18.6-34.2)	-4.19	-2.18
Junagadh	44.0 (36.6-52.6)	27.2 (22.1-33.7)	22.5 (16.4-31.2)	-4.67	-2.72
Kachchh	55.0 (45.3-65.7)	38.7 (31.3-47.6)	36.1 (26.9-47.5)	-3.46	-0.98
Kheda	68.2 (60.5-77.1)	53.3 (46.3-60.4)	39.5 (31.0-50.9)	-2.43	-4.20
Mahesana	60.5 (53.8-68.6)	46.5 (40.1-53.2)	34.0 (26.3-43.6)	-2.61	-4.36
Mahisagar	72.6 (65.1-80.5)	55.6 (49.0-62.0)	40.4 (31.9-51.1)	-2.63	-4.48
Morbi	49.4 (40.3-59.8)	34.4 (27.4-43.2)	30.6 (22.2-40.7)	-3.56	-1.65
Narmada	66.2 (57.4-75.6)	48.7 (41.9-56.5)	41.7 (31.1-53.9)	-3.03	-2.17
Navsari	40.2 (34.4-46.2)	27.6 (23.3-32.3)	23.8 (17.8-31.5)	-3.67	-2.09
Panchmahal	71.1 (63.5-79.4)	54.1 (47.5-60.7)	40.5 (31.6-50.9)	-2.70	-4.04
Patan	64.2 (55.7-73.9)	49.9 (42.2-58.1)	40.4 (31.1-51.1)	-2.49	-2.95
Porbandar	44.0 (35.4-53.8)	27.7 (22.0-34.5)	22.7 (16.0-32.2)	-4.53	-2.76
Rajkot	42.9 (36.3-50.6)	28.0 (23.0-33.8)	23.5 (17.7-31.1)	-4.17	-2.46
Sabar Kantha	70.8 (63.5-79.0)	57.4 (50.3-65.5)	39.8 (31.1-50.4)	-2.07	-5.12
Surat	41.8 (35.4-48.9)	29.3 (24.5-35.3)	24.8 (18.2-33.1)	-3.48	-2.36
Surendranagar	54.1 (45.7-63.6)	39.7 (32.9-47.7)	33.9 (25.3-43.3)	-3.05	-2.22
Tapi	53.1 (46.9-59.7)	38.1 (33.2-43.5)	34.4 (26.0-44.0)	-3.25	-1.48
Vadodara	67.4 (59.2-76.4)	50.2 (43.2-57.4)	38.4 (29.7-48.6)	-2.91	-3.76
Valsad	38.9 (33.1-45.0)	27.6 (23.3-32.7)	24.7 (18.3-32.7)	-3.35	-1.57
**Manipur**	**34.5 (30.5-38.8)**	**23.9 (20.7-27.7)**	**19.3 (16.8-22.5)**	**-3.66**	**-3.02**
Bishnupur	32.9 (28.8-37.5)	23.3 (20.2-26.7)	18.7 (13.9-24.4)	-3.39	-3.09
Chandel	31.9 (27.4-36.7)	25.3 (21.7-29.2)	21.1 (15.9-27.6)	-2.29	-2.54
Churachandpur	36.0 (31.8-40.6)	26.6 (23.2-30.0)	21.3 (16.2-27.4)	-3.00	-3.11
Imphal East	31.9 (28.1-36.0)	21.8 (19.0-24.6)	17.4 (13.2-22.2)	-3.75	-3.15
Imphal West	30.9 (27.1-35.0)	21.7 (19.0-24.6)	16.6 (12.6-21.3)	-3.49	-3.72
Jiribam	65.2 (58.1-72.6)	44.5 (40.0-50.2)	33.8 (26.5-42.5)	-3.74	-3.86
Kakching	30.3 (26.2-34.7)	21.9 (18.9-25.3)	18.0 (13.4-23.5)	-3.18	-2.75
Kamjong	32.1 (27.4-37.4)	22.2 (18.8-25.8)	19.9 (14.9-25.9)	-3.65	-1.55
Kangpokpi	32.7 (28.8-36.8)	22.1 (19.3-24.9)	18.1 (13.8-23.0)	-3.85	-2.79
Noney	48.0 (42.6-53.9)	33.7 (29.9-37.9)	26.6 (20.5-33.8)	-3.50	-3.30
Pherzawl	47.4 (42.7-52.6)	32.9 (29.1-36.8)	26.4 (20.5-33.8)	-3.59	-3.08
Senapati	38.6 (34.3-43.0)	24.9 (22.2-27.7)	20.7 (16.2-26.0)	-4.29	-2.56
Tamenglong	49.9 (44.5-55.5)	32.8 (29.3-36.5)	26.5 (20.7-33.2)	-4.12	-3.02
Tengnoupal	32.0 (27.4-37.0)	23.2 (19.6-27.1)	20.0 (14.9-26.4)	-3.17	-2.10
Thoubal	30.4 (26.5-34.6)	21.3 (18.5-24.5)	17.3 (12.9-22.3)	-3.49	-2.95

Ukhrul	32.1 (28.2-36.6)	21.1 (18.5-24.0)	19.0 (14.6-24.2)	-4.11	-1.46
**Jammu & Kashmir†**	**46.9 (41.9-52.1)**	**38.9 (34.1-44.3)**	**30.5 (26.4-35.7)**	**-1.87**	**-3.47**
Anantnag	48.4 (42.6-55.1)	41.6 (36.1-47.4)	31.9 (25.1-39.7)	-1.49	-3.72
Badgam	49.1 (43.3-55.4)	41.0 (35.9-47.0)	30.6 (24.0-38.1)	-1.77	-4.11
Bandipore	55.7 (48.4-63.9)	46.2 (39.9-53.2)	35.2 (27.4-44.3)	-1.85	-3.82
Baramulla	55.8 (50.2-61.9)	48.6 (43.6-54.2)	36.4 (30.9-42.6)	-1.38	-4.03
Doda	39.5 (34.0-45.8)	37.0 (31.9-43.1)	29.9 (23.1-37.8)	-0.64	-2.99
Ganderbal	55.7 (48.3-63.8)	46.5 (40.5-53.6)	35.6 (27.9-45.0)	-1.78	-3.74
Jammu	42.3 (38.2-46.7)	34.5 (31.0-38.2)	29.2 (24.4-34.5)	-2.02	-2.33
Kargil	62.2 (54.0-71.7)	53.1 (46.0-61.4)	41.6 (32.9-51.5)	-1.56	-3.44
Kathua	43.8 (38.8-49.2)	35.1 (31.2-39.7)	28.5 (22.9-35.0)	-2.21	-2.90
Kishtwar	47.7 (41.4-55.5)	43.2 (37.3-50.0)	35.1 (27.4-44.0)	-0.97	-2.92
Kulgam	43.2 (38.5-48.9)	37.5 (32.9-42.7)	28.6 (22.7-35.5)	-1.42	-3.77
Kupwara	55.8 (49.3-63.0)	47.0 (41.7-53.2)	35.3 (29.0-42.3)	-1.70	-4.01
Leh	48.0 (38.3-59.1)	38.1 (30.4-48.1)	30.7 (22.7-40.7)	-2.29	-3.05
PoJK	59.3 (50.9-67.9)	54.0 (46.6-61.8)	39.7 (32.7-47.9)	-0.94	-4.29
Poonch	45.7 (41.3-50.5)	38.8 (34.9-43.2)	31.9 (27.1-37.1)	-1.62	-2.76
Pulwama	48.4 (42.7-55.1)	40.9 (35.7-46.6)	31.2 (24.5-38.7)	-1.67	-3.82
Rajouri	40.7 (36.0-45.3)	33.3 (29.8-37.4)	28.8 (24.2-34.3)	-2.00	-2.01
Ramban	38.7 (33.7-44.4)	35.1 (30.4-40.3)	28.2 (22.2-35.5)	-0.99	-3.05
Reasi	38.1 (33.5-43.0)	32.2 (28.3-36.7)	26.3 (20.8-32.9)	-1.69	-2.86
Samba	44.9 (40.1-50.3)	36.6 (32.7-40.9)	30.4 (25.0-36.1)	-2.01	-2.64
Shopian	45.6 (40.6-51.3)	38.9 (34.3-44.2)	29.6 (23.5-36.6)	-1.58	-3.85
Srinagar	50.2 (43.7-57.3)	41.1 (35.6-47.3)	29.9 (23.3-37.6)	-1.97	-4.44
Udhampur	39.2 (34.5-44.4)	33.4 (29.3-38.3)	26.7 (21.0-33.3)	-1.59	-3.13
**Haryana**	**58.5 (52.3-65.1)**	**47.2 (41.1-54.5)**	**34.7 (29.8-40.6)**	**-2.15**	**-4.40**
Ambala	51.1 (46.1-56.9)	39.6 (34.9-44.5)	28.3 (22.5-34.8)	-2.51	-4.70
Bhiwani	58.0 (52.0-64.3)	45.0 (40.0-50.1)	32.8 (25.8-40.8)	-2.52	-4.41
Charkhi Dadri	58.5 (51.6-66.1)	45.5 (39.8-51.5)	32.9 (25.6-41.4)	-2.48	-4.53
Faridabad	55.7 (50.4-61.0)	50.1 (45.1-55.4)	36.0 (28.7-44.6)	-1.05	-4.62
Fatehabad	59.0 (52.1-65.9)	45.1 (39.6-51.0)	35.1 (27.5-43.5)	-2.65	-3.52
Gurugram	55.5 (50.8-60.5)	48.1 (43.3-53.2)	35.7 (28.6-44.3)	-1.41	-4.18
Hisar	58.6 (52.4-64.5)	45.4 (40.4-50.5)	34.2 (27.1-42.2)	-2.51	-3.97
Jhajjar	58.9 (52.7-65.4)	46.4 (41.1-52.0)	34.7 (27.5-43.7)	-2.36	-4.05
Jind	63.8 (57.1-71.0)	49.3 (43.8-55.3)	36.1 (28.4-44.6)	-2.54	-4.36
Kaithal	62.8 (55.7-70.1)	46.9 (40.9-53.6)	34.0 (26.2-42.6)	-2.87	-4.51
Karnal	63.3 (57.0-69.7)	49.7 (44.1-55.6)	35.1 (27.7-43.5)	-2.40	-4.84
Kurukshetra	57.0 (51.4-63.1)	43.7 (38.6-49.2)	31.5 (24.9-38.9)	-2.61	-4.59
Mahendragarh	50.1 (45.0-55.7)	39.1 (34.7-43.7)	28.2 (22.3-35.4)	-2.45	-4.56
Nuh	67.4 (60.9-74.4)	63.0 (56.3-70.0)	49.8 (39.4-62.0)	-0.66	-3.31
Palwal	68.0 (61.3-75.3)	61.4 (55.1-67.9)	47.5 (38.0-59.0)	-1.02	-3.60
Panchkula	47.2 (41.8-53.2)	37.9 (32.9-43.1)	27.8 (21.8-34.6)	-2.17	-4.34
Panipat	61.5 (55.6-67.3)	48.5 (43.3-54.1)	33.8 (26.8-41.9)	-2.35	-5.03
Rewari	56.0 (50.6-61.9)	46.7 (41.6-51.9)	34.1 (27.0-42.8)	-1.81	-4.39
Rohtak	60.7 (54.2-67.3)	47.1 (42.0-52.5)	34.6 (27.5-43.7)	-2.49	-4.30
Sirsa	50.8 (44.8-57.0)	38.2 (33.8-43.4)	31.0 (24.3-39.0)	-2.82	-2.92
Sonipat	62.6 (56.9-68.7)	48.9 (44.1-54.1)	35.3 (28.0-43.5)	-2.45	-4.55
Yamunanagar	57.5 (51.7-63.7)	47.3 (41.8-53.1)	33.1 (26.2-40.9)	-1.95	-4.98
**Uttarakhand**	**45.1 (40.1-50.8)**	**35.8 (31.5-40.8)**	**26.1 (22.7-30.6)**	**-2.31**	**-4.49**
Almora	38.3 (34.6-42.2)	27.2 (24.1-30.4)	19.9 (15.8-24.5)	-3.35	-4.41
Bageshwar	40.6 (36.2-45.5)	29.3 (25.6-33.4)	20.9 (16.4-26.5)	-3.20	-4.76
Chamoli	35.0 (30.1-40.3)	26.9 (22.9-31.6)	19.3 (14.9-24.8)	-2.60	-4.59
Champawat	49.9 (44.6-55.4)	33.7 (29.9-37.8)	23.5 (18.7-29.4)	-3.86	-5.02
Dehradun	41.1 (37.1-45.7)	34.8 (30.9-39.1)	25.7 (20.6-31.9)	-1.65	-4.24
Haridwar	54.8 (49.7-60.5)	44.7 (39.7-50.0)	33.1 (26.7-40.6)	-2.01	-4.22
Nainital	41.5 (37.5-46.4)	31.5 (28.1-35.0)	23.0 (18.6-28.6)	-2.74	-4.37
Pauri Garhwal	40.6 (36.4-45.1)	33.0 (29.2-37.3)	24.1 (19.5-29.8)	-2.03	-4.39
Pithoragarh	45.0 (38.4-51.8)	31.2 (26.8-36.5)	21.4 (16.4-27.9)	-3.58	-5.26

Rudraprayag	35.4 (30.7-40.7)	29.3 (24.7-34.5)	21.2 (16.2-27.3)	-1.87	-4.51
Tehri Garhwal	38.8 (34.4-43.4)	32.7 (28.3-37.3)	24.0 (19.0-29.8)	-1.67	-4.36
Udham Singh Nagar	50.7 (45.8-56.1)	40.0 (35.8-44.2)	28.8 (23.4-35.6)	-2.35	-4.57
Uttarkashi	41.0 (36.2-46.5)	36.1 (31.1-41.2)	27.0 (21.0-33.8)	-1.26	-4.08
**Tamil Nadu**	**43.3 (39.0-48.1)**	**26.8 (22.0-32.4)**	**17.6 (15.1-20.5)**	**-4.80**	**-6.04**
Ariyalur	50.9 (44.1-58.5)	31.6 (26.8-37.4)	21.7 (15.6-29.6)	-4.66	-5.23
Chennai	30.8 (25.3-37.2)	18.0 (14.4-22.2)	12.8 (9.0-18.2)	-5.25	-4.78
Coimbatore	35.2 (29.9-41.1)	22.0 (18.4-26.2)	13.1 (9.4-18.0)	-4.59	-7.15
Cuddalore	48.2 (41.8-55.0)	28.7 (24.6-33.4)	19.6 (14.4-26.5)	-5.07	-5.30
Dharmapuri	51.3 (45.0-58.6)	33.4 (28.7-38.9)	20.4 (15.0-27.0)	-4.21	-6.80
Dindigul	45.7 (39.1-53.3)	29.5 (25.0-35.5)	18.4 (13.0-25.4)	-4.28	-6.56
Erode	49.3 (42.7-56.8)	32.0 (27.0-37.3)	19.1 (13.9-25.8)	-4.24	-7.08
Kanchipuram	38.8 (32.6-45.9)	21.4 (17.6-25.7)	15.9 (11.4-22.3)	-5.77	-4.20
Kanniyakumari	26.2 (19.6-33.6)	16.4 (12.2-20.8)	9.8 (6.1-14.5)	-4.55	-7.14
Karur	44.8 (39.0-51.7)	29.0 (24.3-34.0)	18.2 (13.2-24.4)	-4.28	-6.45
Krishnagiri	56.5 (48.6-64.4)	35.7 (30.8-41.8)	22.0 (16.0-29.0)	-4.48	-6.68
Madurai	48.9 (41.2-57.3)	31.3 (26.2-37.0)	20.1 (13.7-27.6)	-4.35	-6.17
Nagapattinam	44.2 (37.1-52.2)	26.3 (21.8-31.6)	18.2 (12.8-25.2)	-5.07	-5.07
Namakkal	43.6 (37.0-50.8)	28.4 (23.5-33.4)	17.4 (12.5-23.6)	-4.18	-6.80
Perambalur	46.7 (40.7-53.5)	29.5 (25.2-34.4)	19.6 (14.0-26.3)	-4.48	-5.70
Pudukkottai	49.2 (42.7-56.9)	29.9 (25.4-35.0)	20.1 (14.5-27.1)	-4.84	-5.53
Ramanathapuram	39.7 (31.5-48.8)	23.9 (18.7-29.1)	16.1 (10.9-22.9)	-4.94	-5.53
Salem	46.9 (40.5-53.8)	30.8 (26.0-35.9)	18.7 (13.6-25.1)	-4.12	-6.90
Sivaganga	45.9 (38.7-54.5)	27.4 (22.8-32.3)	18.3 (12.7-25.1)	-5.03	-5.60
Thanjavur	48.3 (41.6-56.0)	29.6 (25.0-35.1)	20.1 (14.3-27.8)	-4.77	-5.39
The Nilgiris	39.5 (34.0-45.3)	24.5 (20.6-28.8)	14.6 (10.6-19.8)	-4.67	-7.12
Theni	47.4 (39.3-57.4)	30.4 (24.6-36.8)	19.3 (12.9-27.3)	-4.34	-6.30
Thiruvallur	36.3 (30.5-43.0)	20.7 (16.9-25.1)	15.1 (10.8-21.2)	-5.44	-4.42
Thiruvarur	46.4 (39.2-54.5)	28.1 (23.5-33.6)	19.6 (13.9-27.3)	-4.90	-5.03
Tiruchirappalli	46.6 (40.6-53.6)	29.7 (25.3-34.6)	19.1 (13.8-25.7)	-4.41	-6.09
Tirunelveli	35.0 (29.2-42.1)	22.8 (18.2-27.5)	14.5 (9.6-20.5)	-4.21	-6.24
Tiruppur	42.3 (35.1-50.7)	26.8 (22.2-32.5)	16.0 (11.3-22.1)	-4.46	-7.06
Tiruvannamalai	46.4 (39.5-54.1)	27.9 (23.4-33.3)	19.4 (14.4-26.4)	-4.96	-5.04
Thoothukudi	38.5 (31.6-46.6)	25.2 (20.2-30.8)	16.5 (11.0-23.6)	-4.15	-5.91
Vellore	46.2 (40.1-53.1)	28.2 (24.2-32.9)	19.2 (13.9-26.2)	-4.82	-5.33
Viluppuram	47.7 (41.6-54.9)	28.7 (24.8-33.4)	19.8 (14.6-26.5)	-4.96	-5.18
Virudhunagar	47.0 (39.9-55.2)	30.5 (25.3-36.2)	19.9 (13.6-27.3)	-4.23	-5.91
**Mizoram**	**31.8 (28.5-35.6)**	**34.6 (30.0-39.7)**	**28.8 (24.8-33.9)**	**0.83**	**-2.61**
Aizawl	30.3 (27.2-33.7)	31.7 (27.9-35.4)	26.9 (20.9-34.5)	0.44	-2.29
Champhai	25.9 (22.6-29.5)	31.0 (26.7-35.5)	25.6 (19.8-33.2)	1.82	-2.72
Kolasib	33.3 (30.0-37.0)	33.3 (29.5-37.3)	27.8 (21.5-35.8)	0.00	-2.57
Lawngtlai	35.8 (31.5-40.6)	40.8 (35.7-46.6)	34.0 (26.1-43.6)	1.30	-2.57
Lunglei	33.2 (29.2-37.7)	36.7 (31.9-41.8)	29.7 (22.8-38.1)	0.99	-2.96
Mamit	39.0 (34.9-43.9)	38.4 (33.9-43.4)	32.1 (24.8-41.3)	-0.18	-2.52
Saiha	36.7 (31.5-42.9)	45.3 (38.9-52.2)	37.6 (28.7-48.5)	2.15	-2.63
Serchhip	29.9 (26.4-33.6)	32.7 (28.3-36.9)	27.0 (21.0-34.9)	0.90	-2.71
**Maharashtra**	**45.4 (40.8-50.6)**	**31.1 (25.7-37.3)**	**22.6 (19.5-26.5)**	**-3.79**	**-4.55**
Ahmednagar	43.2 (37.4-50.4)	29.5 (24.7-34.3)	21.3 (15.8-27.7)	-3.75	-4.53
Akola	48.2 (41.9-55.1)	35.0 (30.1-40.8)	24.7 (18.3-31.6)	-3.15	-4.85
Amravati	54.3 (46.6-63.6)	35.7 (30.4-42.3)	25.6 (18.9-33.3)	-4.10	-4.66
Aurangabad	45.3 (39.2-52.3)	32.6 (27.6-38.0)	23.2 (17.1-30.1)	-3.23	-4.73
Beed	43.6 (38.1-50.0)	30.6 (26.3-35.2)	22.5 (16.7-28.8)	-3.49	-4.28
Bhandara	64.0 (55.9-72.9)	39.2 (33.8-45.7)	29.4 (22.6-38.6)	-4.78	-4.03
Buldhana	49.8 (43.2-57.2)	37.3 (32.1-42.7)	26.0 (19.3-33.5)	-2.85	-5.00
Chandrapur	57.2 (49.4-65.5)	39.2 (33.1-46.1)	29.9 (22.7-39.3)	-3.71	-3.78
Dhule	52.6 (45.5-60.6)	37.7 (32.1-44.1)	26.3 (19.9-34.1)	-3.29	-4.98
Gadchiroli	65.0 (56.4-74.7)	45.3 (39.0-52.6)	35.6 (27.5-45.5)	-3.55	-3.37
Gondia	69.6 (61.5-78.8)	43.4 (38.2-49.6)	31.1 (24.0-40.4)	-4.63	-4.62

Hingoli	47.5 (41.0-54.0)	38.1 (32.7-43.8)	26.6 (19.9-35.0)	-2.16	-5.03
Jalgaon	55.1 (47.6-62.9)	39.4 (33.8-45.6)	26.8 (20.2-34.7)	-3.29	-5.37
Jalna	48.1 (41.7-55.2)	36.6 (31.3-41.9)	26.5 (19.5-34.4)	-2.71	-4.48
Kolhapur	44.0 (36.3-52.4)	24.3 (19.9-29.2)	16.9 (12.1-23.1)	-5.78	-5.05
Latur	44.6 (38.2-51.7)	29.2 (24.4-34.5)	20.7 (15.3-27.2)	-4.14	-4.79
Mumbai City	34.4 (28.1-41.3)	25.2 (20.2-31.4)	19.0 (13.1-25.9)	-3.08	-3.95
Mumbai Suburban	32.9 (27.4-38.9)	24.7 (20.1-30.1)	18.7 (13.3-25.2)	-2.82	-3.88
Nagpur	56.7 (49.6-64.7)	34.8 (30.0-40.1)	26.6 (20.2-34.8)	-4.75	-3.78
Nanded	49.9 (43.1-57.5)	37.1 (31.8-43.0)	26.3 (19.5-34.0)	-2.91	-4.79
Nandurbar	67.3 (59.4-75.3)	47.2 (41.3-53.5)	33.0 (25.2-42.0)	-3.48	-4.97
Nashik	45.7 (39.8-52.3)	31.9 (27.2-37.1)	22.9 (17.4-29.5)	-3.52	-4.62
Osmanabad	45.1 (39.3-51.3)	28.7 (24.6-33.4)	21.0 (15.5-27.4)	-4.41	-4.39
Palghar	41.6 (35.5-48.1)	29.6 (25.0-34.9)	23.2 (17.3-30.2)	-3.35	-3.41
Parbhani	50.1 (43.2-57.9)	39.3 (33.4-45.9)	27.2 (20.4-35.5)	-2.40	-5.10
Pune	38.1 (32.0-45.5)	24.8 (20.3-29.7)	18.1 (13.3-24.3)	-4.22	-4.42
Raigad	37.0 (31.4-43.7)	25.5 (20.9-30.7)	19.3 (13.7-25.9)	-3.65	-3.90
Ratnagiri	39.0 (31.0-48.0)	24.6 (19.1-30.9)	17.7 (12.1-25.2)	-4.52	-4.57
Sangli	45.4 (38.5-52.7)	25.4 (21.2-29.7)	17.8 (13.1-23.7)	-5.66	-4.96
Satara	38.6 (31.8-45.9)	24.1 (19.6-28.6)	17.1 (12.4-23.0)	-4.60	-4.78
Sindhudurg	41.3 (34.1-49.7)	22.8 (18.3-28.4)	17.4 (12.1-24.2)	-5.77	-3.81
Solapur	46.2 (39.6-53.2)	27.6 (23.3-32.5)	20.0 (14.5-26.4)	-5.00	-4.55
Thane	36.4 (31.0-42.6)	27.2 (22.5-32.8)	20.3 (14.7-26.8)	-2.87	-4.07
Wardha	54.4 (47.2-62.4)	35.0 (29.9-40.7)	27.1 (20.3-34.9)	-4.31	-3.57
Washim	45.5 (39.4-51.9)	35.5 (30.5-40.9)	24.8 (18.5-31.7)	-2.45	-4.98
Yavatmal	49.9 (42.8-57.3)	36.9 (31.4-43.0)	27.1 (20.2-35.1)	-2.97	-4.33
**Punjab**	**45.0 (40.2-50.3)**	**34.4 (29.6-40.0)**	**25.0 (21.5-29.3)**	**-2.69**	**-4.58**
Amritsar	42.8 (38.6-47.6)	31.2 (27.5-34.8)	24.8 (20.1-30.4)	-3.12	-3.20
Barnala	53.5 (47.7-59.2)	40.2 (35.6-44.9)	28.7 (22.9-35.7)	-2.81	-4.68
Bathinda	46.9 (42.0-52.0)	35.7 (31.7-40.0)	25.8 (20.4-32.0)	-2.70	-4.53
Faridkot	50.8 (45.5-57.1)	37.5 (33.1-41.9)	28.8 (22.9-36.0)	-3.01	-3.70
Fatehgarh Sahib	42.5 (37.8-47.5)	33.5 (29.4-37.9)	22.0 (17.4-27.0)	-2.36	-5.83
Fazilka	45.7 (41.1-50.8)	34.5 (30.8-38.3)	26.6 (21.3-33.0)	-2.78	-3.64
Ferozepur	56.2 (50.4-62.8)	42.1 (37.2-47.2)	32.5 (26.0-40.5)	-2.85	-3.63
Gurdaspur	38.8 (34.7-43.3)	29.3 (25.8-33.2)	23.0 (18.5-28.4)	-2.77	-3.37
Hoshiarpur	38.7 (34.6-43.3)	30.1 (26.3-34.0)	21.8 (17.2-27.1)	-2.49	-4.49
Jalandhar	42.7 (38.4-47.7)	33.0 (29.1-37.0)	24.2 (19.2-30.1)	-2.56	-4.33
Kapurthala	44.3 (39.8-49.3)	33.2 (29.3-37.2)	25.4 (20.3-31.4)	-2.84	-3.77
Ludhiana	44.5 (39.7-49.2)	36.1 (31.8-40.8)	23.9 (19.1-29.7)	-2.05	-5.72
Mansa	50.7 (44.7-56.2)	39.4 (34.5-44.5)	27.7 (21.9-34.2)	-2.48	-4.92
Moga	53.1 (46.7-60.4)	39.3 (34.2-44.3)	30.0 (23.5-37.9)	-2.96	-3.77
Pathankot	35.0 (30.9-39.5)	28.1 (24.5-32.0)	20.0 (15.7-25.0)	-2.19	-4.75
Patiala	44.7 (40.3-49.4)	34.7 (30.7-39.1)	22.5 (18.0-27.7)	-2.52	-5.97
Rupnagar	38.8 (34.9-43.5)	30.9 (27.0-35.1)	21.1 (16.7-26.0)	-2.27	-5.27
Sahibzada Ajit Singh Nagar	38.9 (34.4-44.0)	30.5 (26.5-35.0)	20.0 (15.6-25.0)	-2.41	-5.87
Sangrur	52.3 (47.0-57.6)	40.1 (35.6-44.7)	27.3 (21.7-33.7)	-2.61	-5.36
Shahid Bhagat Singh Nagar	41.5 (37.3-46.1)	33.1 (29.0-37.3)	23.0 (18.4-28.5)	-2.25	-5.02
Sri Mukhtar Sahib	44.8 (40.3-49.7)	33.2 (29.6-37.0)	25.4 (20.2-31.6)	-2.95	-3.76
Tarn Taran	50.1 (45.1-55.7)	35.8 (31.9-40.0)	29.8 (24.0-36.5)	-3.30	-2.57
**Sikkim**	**36.0 (30.9-41.4)**	**26.4 (22.4-30.8)**	**17.6 (15.3-20.3)**	**-3.11**	**-5.81**
East Sikkim	33.3 (29.1-38.3)	24.2 (21.0-28.0)	15.8 (12.3-20.0)	-3.13	-5.93
North Sikkim	42.8 (36.9-49.3)	31.1 (26.6-36.2)	21.2 (16.3-27.3)	-3.15	-5.32
South Sikkim	34.6 (30.5-39.4)	25.2 (21.8-29.1)	16.5 (12.9-20.9)	-3.12	-5.91
West Sikkim	39.6 (34.9-44.9)	29.5 (25.5-34.1)	19.8 (15.4-25.4)	-2.92	-5.50
**Nagaland**	**45.3 (39.4-51.3)**	**30.9 (26.9-35.3)**	**26.3 (22.6-30.8)**	**-3.84**	**-2.31**
Dimapur	53.1 (46.5-60.5)	34.6 (30.5-39.4)	28.3 (22.0-35.7)	-4.18	-2.83
Kiphire	31.7 (26.7-37.0)	22.4 (18.9-26.4)	22.0 (16.7-28.2)	-3.42	-0.28
Kohima	40.0 (35.3-45.4)	26.4 (23.4-29.8)	22.6 (17.7-28.2)	-4.07	-2.24
Longleng	47.6 (42.0-53.8)	33.8 (29.9-38.4)	28.5 (22.2-35.5)	-3.37	-2.43

Mokokchung	50.2 (44.2-56.5)	33.7 (29.7-38.1)	27.0 (20.8-33.9)	-3.92	-3.09
Mon	48.7 (43.1-55.1)	35.5 (31.3-40.5)	30.3 (23.5-38.5)	-3.11	-2.24
Peren	52.5 (46.6-59.1)	35.8 (31.8-40.2)	29.8 (23.4-37.3)	-3.76	-2.60
Phek	32.3 (28.0-36.9)	22.2 (19.2-25.5)	20.7 (15.9-26.1)	-3.67	-1.02
Tuensang	36.0 (30.9-41.6)	26.0 (22.2-30.0)	24.3 (18.8-30.7)	-3.22	-0.96
Wokha	50.7 (45.0-57.4)	33.8 (29.9-37.9)	27.2 (21.5-34.2)	-3.99	-3.03
Zunheboto	37.5 (32.4-42.8)	25.6 (22.1-29.3)	22.7 (17.7-28.5)	-3.74	-1.70
**Himachal Pradesh**	**45.9 (41.1-51.0)**	**35.5 (30.7-41.1)**	**27.5 (23.8-32.2)**	**-2.55**	**-3.64**
Bilaspur	40.4 (35.7-45.8)	29.6 (25.2-33.9)	23.0 (18.0-28.7)	-3.08	-3.52
Chamba	46.3 (39.6-53.4)	38.2 (32.4-44.9)	30.8 (23.4-39.4)	-1.90	-3.06
Hamirpur	38.4 (33.5-44.3)	28.4 (23.9-33.3)	21.9 (17.0-27.8)	-2.98	-3.63
Kangra	44.6 (38.7-50.7)	34.8 (29.5-40.2)	27.4 (21.1-34.7)	-2.46	-3.35
Kinnaur	53.9 (45.1-63.6)	43.6 (36.7-51.9)	32.7 (24.8-42.1)	-2.09	-4.02
Kullu	53.8 (46.8-61.4)	43.7 (37.0-50.6)	33.6 (25.9-42.6)	-2.06	-3.69
Lahul and Spiti	49.8 (42.0-58.2)	40.5 (33.6-47.7)	31.9 (23.9-41.2)	-2.06	-3.35
Mandi	44.7 (39.8-50.3)	34.7 (30.1-39.7)	26.0 (20.5-32.3)	-2.49	-4.07
Shimla	54.0 (47.1-62.0)	43.0 (36.8-50.2)	32.7 (25.4-41.2)	-2.25	-3.82
Sirmaur	48.9 (43.8-54.7)	38.8 (34.3-43.8)	29.7 (23.6-36.7)	-2.28	-3.78
Solan	41.8 (37.2-47.0)	29.8 (26.0-34.0)	23.5 (18.6-29.1)	-3.31	-3.36
Una	40.6 (35.6-46.4)	29.0 (24.8-34.0)	23.2 (18.1-29.2)	-3.31	-3.15
**UTs other than Delhi**	**29.2 (24.6-34.4)**	**25.3 (20.9-30.7)**	**19.6 (16.9-23.1)**	**-1.45**	**-3.60**
Chandigarh	30.5 (26.8-34.9)	29.1 (25.0-33.8)	20.6 (15.9-25.9)	-0.47	-4.83
Dadra & Nagar Haveli	35.8 (29.9-42.5)	34.1 (28.0-40.8)	26.0 (19.1-34.9)	-0.50	-3.77
Daman	30.7 (26.1-35.7)	28.1 (23.5-33.3)	20.5 (15.2-27.1)	-0.88	-4.41
Diu	37.2 (29.8-45.1)	30.0 (23.5-37.4)	23.8 (16.9-33.1)	-2.12	-3.23
Karaikal	31.9 (26.3-38.1)	21.3 (17.3-26.2)	19.2 (13.2-26.9)	-3.94	-1.46
Lakshadweep	14.7 (5.8-31.8)	10.4 (4.0-23.9)	9.0 (3.4-20.3)	-3.35	-2.10
Mahe	14.9 (12.3-17.8)	9.7 (7.8-11.7)	8.3 (5.7-11.6)	-4.20	-2.12
Nicobars	15.3 (6.7-31.1)	12.9 (5.4-25.8)	10.4 (3.8-22.1)	-1.67	-3.05
North & Middle Andaman	33.7 (24.3-45.4)	30.9 (21.8-42.8)	22.0 (13.0-34.8)	-0.89	-4.73
Puducherry	27.7 (23.9-31.9)	18.9 (16.1-22.3)	16.8 (12.3-22.8)	-3.75	-1.71
South Andaman	24.6 (17.3-35.3)	21.2 (14.8-29.8)	15.6 (9.1-24.8)	-1.51	-4.30
Yanam	41.6 (33.9-51.1)	27.8 (22.6-34.9)	18.7 (13.4-25.6)	-3.94	-5.48
**Kerala**	**16.5 (14.5-18.6)**	**11.2 (9.6-13.0)**	**9.1 (7.8-10.6)**	**-3.86**	**-2.97**
Alappuzha	14.7 (11.3-18.4)	10.3 (7.9-13.2)	7.9 (5.3-11.6)	-3.51	-3.65
Ernakulam	13.7 (11.3-16.4)	9.1 (7.4-11.5)	7.3 (5.1-10.5)	-3.96	-3.12
Idukki	22.1 (18.7-26.2)	15.2 (12.5-18.4)	12.7 (8.8-17.7)	-3.68	-2.55
Kannur	18.1 (15.2-21.4)	11.5 (9.4-13.9)	10.0 (6.9-13.9)	-4.44	-2.00
Kasaragod	17.5 (14.0-21.3)	11.3 (8.9-14.1)	9.4 (6.4-13.3)	-4.25	-2.71
Kollam	15.7 (12.4-19.2)	11.2 (8.8-14.0)	8.9 (5.9-12.6)	-3.31	-3.31
Kottayam	15.3 (12.2-18.4)	10.4 (8.1-13.0)	8.4 (5.8-12.1)	-3.83	-2.95
Kozhikode	17.1 (14.1-20.5)	11.5 (9.2-14.0)	9.6 (6.5-13.5)	-3.94	-2.46
Malappuram	17.9 (15.1-21.0)	12.2 (10.0-14.8)	9.9 (6.9-13.9)	-3.74	-2.95
Palakkad	17.7 (14.5-21.4)	11.9 (9.5-14.7)	9.7 (6.7-13.7)	-3.92	-2.91
Pathanamthitta	17.3 (13.8-21.0)	11.8 (9.2-14.8)	9.9 (6.7-14.2)	-3.75	-2.46
Thiruvananthapuram	15.8 (12.2-19.9)	11.5 (8.9-14.6)	8.8 (5.6-13.0)	-3.12	-3.81
Thrissur	13.6 (11.0-16.8)	9.1 (7.1-11.6)	7.3 (4.9-10.6)	-3.97	-2.99
Wayanad	22.8 (18.7-27.4)	15.3 (12.2-18.8)	12.3 (8.4-17.3)	-3.92	-3.00
**Delhi**	**46.9 (42.6-51.8)**	**26.4 (21.9-31.8)**	**21.1 (18.2-24.7)**	**-5.75**	**-3.19**
Central	48.5 (43.4-53.2)	27.3 (24.3-30.4)	22.0 (17.6-27.4)	-5.59	-3.02
East	47.2 (42.6-51.6)	26.3 (23.5-29.2)	21.2 (17.0-26.3)	-5.67	-3.06
New Delhi	47.2 (42.4-51.5)	27.4 (24.4-30.4)	22.0 (17.6-27.3)	-5.30	-3.04
North	47.4 (42.9-52.0)	26.4 (23.5-29.4)	20.8 (16.7-25.8)	-5.70	-3.32
North East	47.9 (43.4-52.5)	26.6 (23.8-29.6)	21.2 (17.0-26.3)	-5.71	-3.20
North West	47.2 (42.7-51.7)	25.8 (23.1-28.9)	20.6 (16.5-25.4)	-5.84	-3.19
Shahdara	46.5 (42.1-51.0)	27.1 (24.2-30.1)	20.8 (16.7-25.9)	-5.26	-3.74
South	47.2 (42.8-51.4)	27.0 (24.2-29.9)	21.7 (17.4-26.9)	-5.43	-3.07
South East	46.8 (42.4-51.2)	28.4 (25.4-31.4)	22.8 (18.3-28.1)	-4.90	-3.09

South West	46.4 (42.1-50.6)	26.4 (23.7-29.3)	21.3 (17.0-26.2)	-5.48	-3.05
West	45.8 (41.3-50.1)	25.7 (22.9-28.6)	20.7 (16.6-25.5)	-5.61	-3.04
**Goa**	**27.3 (23.8-31.3)**	**19.3 (15.7-23.1)**	**15.2 (10.8-21.5)**	**-3.48**	**-3.44**
North Goa	27.7 (22.4-33.4)	19.8 (15.9-24.7)	15.6 (10.8-21.9)	-3.29	-3.30
South Goa	27.1 (22.3-32.2)	18.9 (15.4-23.0)	14.8 (10.3-20.3)	-3.56	-3.44

IMR=Infant Mortality Rate. UTs=Union territories.
*The states are listed in increasing order of Socio-demographic Index in 2017.
†The state of Jammu and Kashmir was divided into two union territories in August 2019; as we are reporting findings up to 2017, we report findings for the undivided state of Jammu and Kashmir.

7. District-level U5MR and NMR against the annual rate of change in the three SDI state groups

U5MR

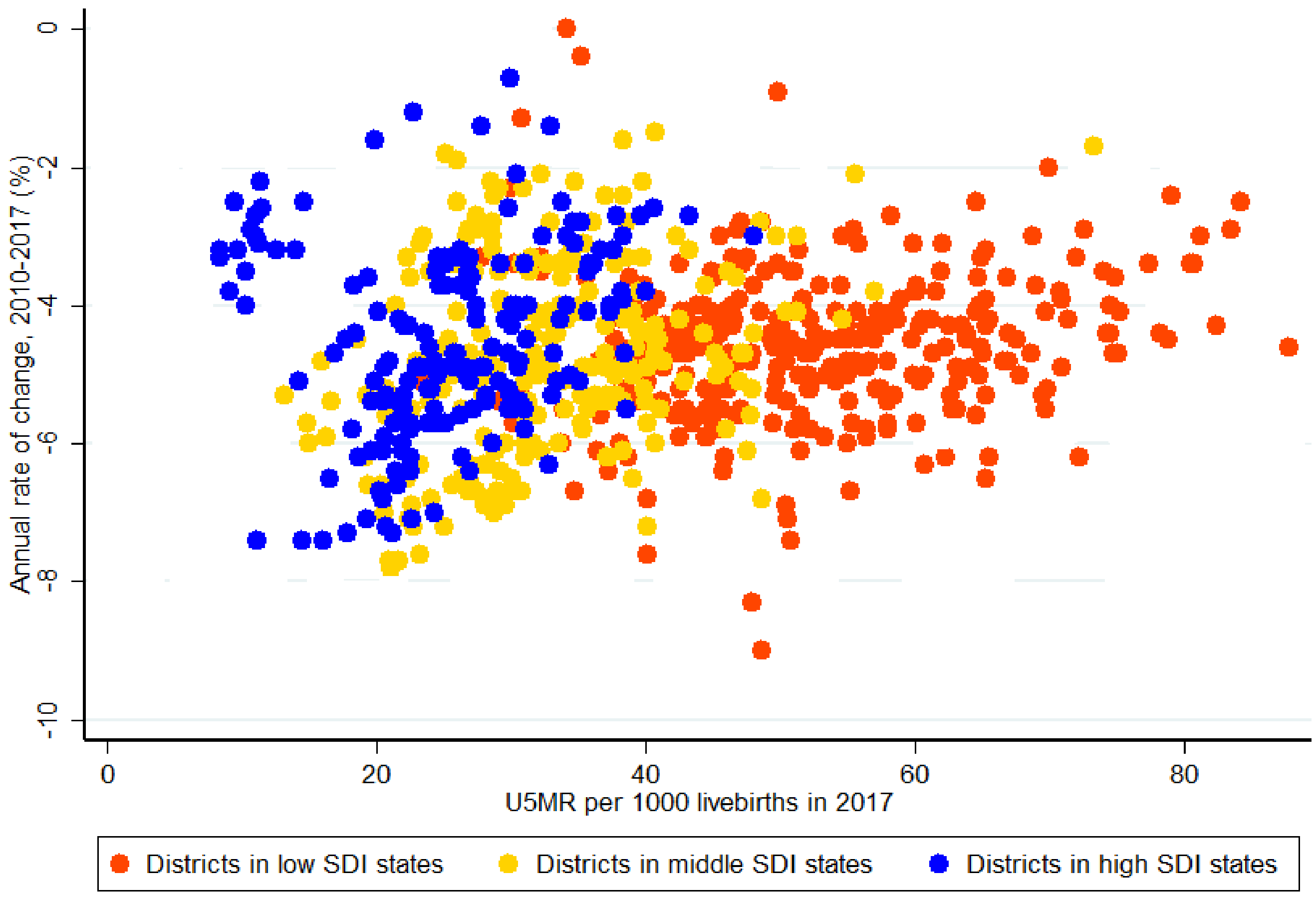

NMR

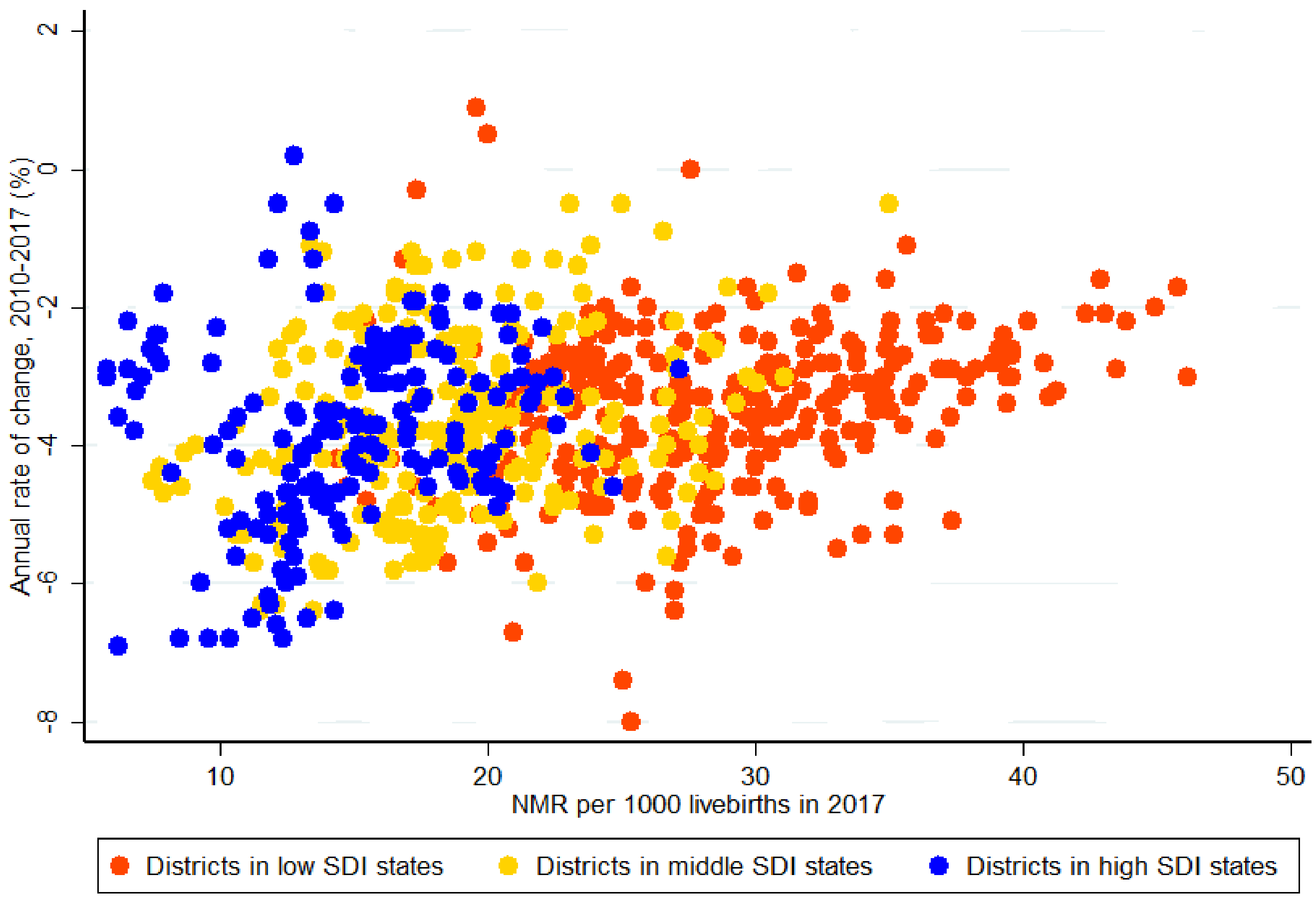

8. Number of under-5 and neonatal deaths in the states of India, 2017

States[*]	Number of under-5 deaths (95% uncertainty interval)	Number of neonatal deaths (95% uncertainty interval)
**India**	**1,035,900 (982,100-1,099,400)**	**571,500 (539,800-608,600)**
Bihar	141,500 (121,500-168,800)	75,300 (63,800-90,700)
Madhya Pradesh	80,100 (68,900-93,800)	42,200 (36,100-50,000)
Jharkhand	28,200 (23,900-33,800)	14,600 (12,100-17,500)
Uttar Pradesh	312,820 (272,260-364,400)	165,800 (142,300-196,300)
Rajasthan	82,500 (71,300-96,900)	45,300 (38,700-53,400)
Chhattisgarh	25,800 (22,400-30,100)	15,700 (13,400-18,500)
Odisha	41,000 (35,000-48,700)	21,200 (17,800-25,600)
Assam	39,200 (33,000-46,900)	21,300 (17,900-25,000)
Andhra Pradesh	21,350 (16,470-28,190)	12,600 (9,500-16,900)
West Bengal	44,600 (38,00-53,300)	27,200 (22,900-32,900)
Tripura	2,870 (2,370-3,580)	1,600 (1,300-1,980)
Arunachal Pradesh	870 (710-1060)	470 (380-580)
Meghalaya	2,990 (2,480-3,670)	1,350 (1,100-1,600)
Karnataka	28,800 (24,800-33,500)	16,900 (14,300-19,900)
Telangana	14,100 (10,800-18,500)	8,800 (6,600-11,800)
Gujarat	40,400 (35,300-46,900)	24,300 (21,100-28,300)
Manipur	1,840 (1,520-2,250)	1,040 (850-1,300)
Jammu and Kashmir[†]	7,340 (6,280-8,680)	4,400 (3,743-5,220)
Haryana	17,800 (15,400-20,800)	9,030 (7,720-10,500)
Uttarakhand	5,160 (4,400-6,010)	2,900 (2,430-3,440)
Tamil Nadu	16,900 (14,600-19,700)	9,750 (8,350-11,440)
Mizoram	870 (720-1040)	440 (360-530)
Maharashtra	44,200 (38,100-51,500)	28,400 (24,100-33,500)
Punjab	13,900 (12,100-16,200)	7,760 (6,609-9,110)
Sikkim	180 (140-230)	100 (80-130)
Nagaland	1,700 (1,370-2,150)	820 (660-1,050)
Himachal Pradesh	3,040 (2,530-3,720)	1,820 (1,510-2,210)
UTs other than Delhi	1,650 (1,380-2,000)	1,030 (850-1,270)
Kerala	4,850 (4,200-5,590)	3,430 (2,940-4,000)
Delhi	9,010 (7,570-10,900)	5,860 (4,780-7,140)
Goa	380 (280-530)	230 (170-320)

UTs=Union territories.
*The states are listed in increasing order of Socio-demographic Index in 2017.
[†]The state of Jammu and Kashmir was divided into two union territories in August 2019; as we are reporting findings up to 2017, we report findings for the undivided state of Jammu and Kashmir.

9. Projected U5MR and NMR for the states of India in 2025 and 2030 based on trends from 2000 to 2017

States*	Projected U5MR (95% uncertainty interval)		Projected NMR (95% uncertainty interval)	
	2025	2030	2025	2030
**India**	**29.8 (25.1-36.0)**	**23.2 (18.9-29.0)**	**17.6 (14.7-21.4)**	**14.7 (11.8-18.4)**
Bihar	30.9 (23.1-41.4)	23.8 (16.7-33.5)	18.2 (13.5-24.3)	15.3 (10.6-21.5)
Madhya Pradesh	34.7 (25.8-45.3)	26.2 (18.4-36.0)	19.6 (14.4-25.4)	15.8 (10.9-21.6)
Jharkhand	31.3 (23.6-41.3)	24.2 (17.4-34.0)	17.4 (13.0-23.3)	14.4 (10.2-20.4)
Uttar Pradesh	43.6 (34.2-56.2)	34.4 (25.9-46.4)	25.0 (19.5-32.3)	21.2 (15.7-28.6)
Rajasthan	35.4 (27.3-44.9)	28.0 (20.5-37.3)	20.9 (15.9-26.6)	17.7 (12.8-23.5)
Chhattisgarh	34.1 (25.0-45.4)	26.5 (18.3-37.3)	22.2 (16.3-29.5)	18.4 (12.6-26.0)
Odisha	34.9 (25.8-45.6)	27.2 (18.9-37.2)	18.8 (13.8-24.8)	15.3 (10.6-21.1)
Assam	43.8 (32.9-56.7)	36.7 (26.0-49.8)	23.8 (17.5-31.0)	20.6 (14.4-28.4)
Andhra Pradesh	24.3 (17.3-33.3)	18.2 (12.1-26.6)	14.5 (10.2-19.9)	11.6 (7.5-17.0)
West Bengal	19.7 (14.6-26.3)	14.8 (10.3-20.8)	13.0 (9.4-17.5)	10.5 (7.1-14.9)
Tripura	25.4 (17.7-35.5)	20.3 (13.0-30.3)	14.6 (10.1-20.4)	12.2 (7.8-18.3)
Arunachal Pradesh	18.6 (12.6-25.7)	13.7 (8.5-20.1)	10.8 (7.3-15.2)	8.6 (5.4-13.1)
Meghalaya	30.2 (21.9-40.7)	24.8 (16.9-35.3)	16.0 (11.5-21.7)	14.2 (9.6-20.4)
Karnataka	22.3 (15.4-31.1)	17.2 (11.0-25.6)	13.4 (9.2-18.7)	10.8 (6.9-16.2)
Telangana	18.2 (12.5-25.9)	13.2 (8.5-20.1)	12.4 (8.4-17.9)	9.8 (6.2-15.2)
Gujarat	27.8 (20.2-37.5)	21.9 (14.8-31.1)	17.9 (12.9-24.2)	15.0 (10.2-21.5)
Manipur	18.3 (12.5-25.8)	14.4 (9.1-21.6)	10.7 (7.2-15.1)	8.9 (5.6-13.5)
Jammu and Kashmir†	25.4 (18.4-33.8)	20.5 (13.9-28.7)	16.0 (11.5-21.4)	13.9 (9.3-19.6)
Haryana	28.8 (21.2-37.7)	22.8 (15.8-31.3)	15.8 (11.6-20.7)	13.5 (9.4-18.4)
Uttarakhand	22.2 (16.6-29.1)	17.4 (12.3-24.1)	13.3 (9.9-17.7)	11.1 (7.8-15.6)
Tamil Nadu	12.4 (8.3-18.0)	8.9 (5.4-14.0)	7.5 (4.8-11.1)	5.7 (3.4-9.2)
Mizoram	32.9 (22.9-45.5)	29.5 (19.1-43.5)	16.8 (11.7-23.7)	15.7 (10.2-23.6)
Maharashtra	18.3 (13.2-24.4)	14.1 (9.5-19.9)	12.4 (8.9-16.5)	10.0 (6.7-14.0)
Punjab	21.7 (16.0-28.8)	17.2 (11.8-23.9)	12.6 (9.2-16.7)	10.5 (7.2-14.7)
Sikkim	14.9 (10.5-20.6)	11.2 (7.4-16.5)	8.6 (6.0-12.1)	6.9 (4.5-10.3)
Nagaland	26.9 (19.2-36.4)	21.8 (14.6-31.4)	12.5 (8.9-17.1)	10.6 (7.0-15.3)
Himachal Pradesh	24.3 (17.3-32.6)	19.9 (13.2-28.3)	14.9 (10.6-20.0)	12.7 (8.5-18.0)
UTs other than Delhi	18.2 (14.4-23.2)	15.2 (11.4-20.4)	11.5 (9.0-14.8)	10.0 (7.5-13.4)
Kerala	7.6 (4.6-11.7)	6.0 (3.3-9.9)	5.2 (3.1-8.2)	4.2 (2.3-7.1)
Delhi	16.9 (12.4-22.8)	12.6 (8.7-17.9)	11.4 (8.3-15.3)	9.0 (6.1-12.8)
Goa	13.8 (8.1-21.2)	10.9 (5.8-18.3)	8.0 (4.6-12.8)	6.5 (3.4-11.2)

UTs=Union Territories.
*The states are listed in increasing order of Socio-demographic Index in 2017.
†The state of Jammu and Kashmir was divided into two union territories in August 2019; as we are reporting findings up to 2017, we report findings for the undivided state of Jammu and Kashmir.

10. U5MR and NMR in the districts of India in 2000, 2010 and 2017

U5MR

States*/Districts	U5MR (95% uncertainty interval)					
	2000	2010	2017	Annual rate of change, 2000-2010 (%)	Annual rate of change, 2010-2017 (%)	Annual rate of change, 2000-2017 (%)
**Bihar**	**88.8 (81.5-96.2)**	**60.3 (54.8-66.1)**	**43.8 (37.9-51.1)**	**-3.80 (-4.45 to -3.10)**	**-4.51 (-7.10 to -1.73)**	**-4.09 (-5.23 to -2.97)**
Araria	89.8 (80.9-98.8)	61.2 (55.0-68.2)	45.9 (36.1-57.4)	-3.79 (-4.75 to -2.78)	-4.09 (-6.81 to -1.22)	-3.92 (-5.17 to -2.57)
Arwal	85.1 (75.9-94.4)	59.4 (52.6-66.0)	43.8 (34.6-55.1)	-3.54 (-4.55 to -2.48)	-4.34 (-7.44 to -1.32)	-3.87 (-5.16 to -2.57)
Aurangabad	88.8 (80.0-98.3)	63.1 (56.1-70.2)	46.5 (36.9-58.5)	-3.36 (-4.35 to -2.29)	-4.33 (-7.33 to -1.33)	-3.76 (-5.04 to -2.48)
Banka	89.2 (80.8-99.4)	63.0 (56.5-70.3)	46.4 (37.0-58.8)	-3.42 (-4.41 to -2.36)	-4.34 (-7.31 to -1.38)	-3.80 (-5.12 to -2.59)
Begusarai	83.7 (76.1-91.4)	57.5 (51.7-63.2)	41.6 (33.1-51.9)	-3.69 (-4.56 to -2.75)	-4.60 (-7.45 to -1.63)	-4.07 (-5.39 to -2.82)
Bhagalpur	89.8 (81.5-98.8)	61.9 (55.6-69.0)	45.5 (36.4-57.5)	-3.65 (-4.57 to -2.66)	-4.36 (-7.22 to -1.32)	-3.95 (-5.22 to -2.65)
Bhojpur	83.4 (75.7-91.2)	57.7 (52.1-63.7)	41.9 (33.6-52.2)	-3.62 (-4.52 to -2.69)	-4.51 (-7.46 to -1.49)	-3.99 (-5.24 to -2.79)
Buxar	85.0 (77.6-93.1)	58.5 (53.1-64.3)	43.3 (34.7-53.9)	-3.66 (-4.49 to -2.78)	-4.29 (-7.19 to -1.23)	-3.92 (-5.18 to -2.67)
Darbhanga	92.0 (83.8-100.4)	60.3 (54.3-66.7)	43.2 (34.5-54.0)	-4.14 (-5.02 to -3.21)	-4.72 (-7.54 to -1.70)	-4.38 (-5.65 to -3.11)
East Champaran	92.7 (84.4-102.0)	60.4 (54.1-67.1)	44.3 (35.1-55.6)	-4.20 (-5.15 to -3.21)	-4.38 (-7.38 to -1.20)	-4.28 (-5.62 to -3.06)
Gaya	86.1 (77.7-95.2)	62.3 (55.8-68.7)	45.2 (36.1-56.4)	-3.18 (-4.06 to -2.22)	-4.53 (-7.46 to -1.62)	-3.74 (-4.98 to -2.53)
Gopalganj	84.0 (76.4-91.6)	55.6 (50.1-61.7)	40.2 (31.6-50.5)	-4.05 (-4.93 to -3.10)	-4.60 (-7.67 to -1.49)	-4.28 (-5.59 to -3.06)
Jamui	85.0 (77.4-93.4)	61.1 (55.3-67.9)	44.7 (36.0-56.1)	-3.26 (-4.11 to -2.33)	-4.42 (-7.33 to -1.57)	-3.74 (-5.03 to -2.53)
Jehanabad	84.3 (76.3-92.6)	59.1 (52.9-65.2)	43.2 (34.7-54.0)	-3.49 (-4.39 to -2.54)	-4.43 (-7.41 to -1.48)	-3.88 (-5.12 to -2.69)
Kaimur	90.4 (82.6-99.8)	61.9 (55.9-68.5)	47.0 (37.3-58.8)	-3.72 (-4.57 to -2.87)	-3.90 (-6.87 to -0.76)	-3.79 (-5.04 to -2.51)
Katihar	101.4 (91.2-111.8)	70.7 (63.1-78.9)	51.1 (40.1-64.5)	-3.56 (-4.51 to -2.54)	-4.61 (-7.42 to -1.66)	-4.00 (-5.27 to -2.73)
Khagaria	84.2 (76.0-92.5)	56.9 (51.0-63.2)	42.7 (34.0-53.8)	-3.84 (-4.82 to -2.83)	-4.07 (-6.97 to -1.00)	-3.94 (-5.25 to -2.57)
Kishanganj	89.5 (80.5-99.9)	61.8 (55.1-69.5)	45.6 (35.9-56.9)	-3.66 (-4.74 to -2.59)	-4.23 (-7.04 to -1.09)	-3.90 (-5.17 to -2.55)
Lakhisarai	81.7 (73.2-90.4)	57.6 (51.3-64.7)	42.5 (33.9-53.9)	-3.45 (-4.46 to -2.41)	-4.30 (-7.22 to -1.33)	-3.80 (-5.13 to -2.52)
Madhepura	88.3 (79.4-97.7)	59.8 (53.2-66.6)	45.0 (35.5-56.7)	-3.83 (-4.85 to -2.77)	-4.05 (-6.94 to -0.93)	-3.92 (-5.29 to -2.48)
Madhubani	106.0 (95.4-116.7)	66.7 (59.3-73.8)	47.9 (38.0-61.0)	-4.53 (-5.43 to -3.58)	-4.68 (-7.52 to -1.58)	-4.60 (-5.93 to -3.27)
Munger	82.8 (75.4-90.7)	56.0 (50.4-61.8)	41.1 (32.8-51.8)	-3.85 (-4.73 to -2.92)	-4.35 (-7.29 to -1.37)	-4.06 (-5.35 to -2.81)
Muzaffarpur	87.9 (80.0-95.9)	58.6 (52.7-65.4)	41.4 (32.8-52.0)	-3.98 (-4.95 to -2.97)	-4.90 (-7.88 to -1.75)	-4.37 (-5.69 to -3.11)
Nalanda	82.9 (75.4-90.6)	59.2 (53.6-65.5)	42.5 (34.3-53.7)	-3.32 (-4.16 to -2.42)	-4.66 (-7.37 to -1.77)	-3.88 (-5.09 to -2.71)
Nawada	83.4 (75.7-91.7)	61.1 (55.3-67.5)	44.0 (35.6-55.4)	-3.08 (-3.94 to -2.18)	-4.64 (-7.42 to -1.81)	-3.73 (-4.92 to -2.55)
Patna	78.8 (71.9-85.4)	53.8 (48.9-59.1)	38.0 (30.8-47.5)	-3.76 (-4.58 to -2.91)	-4.91 (-7.75 to -1.96)	-4.24 (-5.47 to -3.09)
Purnia	95.8 (86.3-105.4)	67.3 (60.2-75.0)	49.9 (39.7-62.5)	-3.48 (-4.47 to -2.46)	-4.22 (-7.03 to -1.26)	-3.79 (-5.07 to -2.48)
Rohtas	88.2 (80.2-97.0)	61.3 (55.2-67.5)	44.9 (35.8-55.9)	-3.58 (-4.51 to -2.64)	-4.42 (-7.42 to -1.36)	-3.93 (-5.16 to -2.65)
Saharsa	85.3 (76.0-94.8)	57.6 (50.9-64.6)	43.1 (33.7-54.4)	-3.85 (-4.95 to -2.73)	-4.13 (-7.09 to -0.97)	-3.97 (-5.37 to -2.50)
Samastipur	86.2 (78.7-93.4)	58.7 (53.2-64.7)	41.9 (33.8-52.0)	-3.77 (-4.63 to -2.86)	-4.75 (-7.56 to -1.81)	-4.18 (-5.43 to -2.97)
Saran	81.5 (73.5-89.1)	55.4 (49.7-61.7)	39.2 (31.2-48.9)	-3.79 (-4.79 to -2.81)	-4.88 (-7.84 to -1.66)	-4.25 (-5.59 to -2.97)
Sheikhpura	82.7 (73.9-91.7)	60.1 (53.6-68.0)	43.4 (34.7-55.4)	-3.14 (-4.15 to -2.13)	-4.60 (-7.51 to -1.61)	-3.75 (-5.03 to -2.47)
Sheohar	99.2 (89.7-108.9)	64.5 (57.8-71.9)	46.3 (36.5-58.4)	-4.22 (-5.19 to -3.19)	-4.65 (-7.64 to -1.46)	-4.40 (-5.76 to -3.18)
Sitamarhi	109.6 (99.2-120.1)	69.7 (62.3-77.5)	50.8 (40.2-64.1)	-4.43 (-5.39 to -3.43)	-4.48 (-7.37 to -1.26)	-4.45 (-5.77 to -3.25)
Siwan	83.3 (75.8-90.9)	56.0 (50.7-62.1)	39.8 (31.3-49.6)	-3.91 (-4.75 to -2.98)	-4.82 (-7.81 to -1.67)	-4.29 (-5.57 to -3.07)
Supaul	92.3 (82.9-102.1)	60.1 (53.4-66.9)	44.4 (35.0-56.2)	-4.20 (-5.19 to -3.19)	-4.32 (-7.09 to -1.25)	-4.25 (-5.58 to -2.84)
Vaishali	84.2 (76.6-91.4)	57.6 (52.2-63.8)	40.1 (32.0-50.0)	-3.73 (-4.61 to -2.80)	-5.08 (-7.86 to -2.02)	-4.29 (-5.53 to -3.08)
West Champaran	89.8 (80.3-100.0)	58.0 (51.5-65.3)	43.2 (33.9-54.6)	-4.28 (-5.21 to -3.33)	-4.18 (-7.24 to -1.03)	-4.24 (-5.53 to -3.05)
**Madhya Pradesh**	**110.5 (101.4-120.6)**	**75.4 (68.4-82.7)**	**50.7 (43.9-59.2)**	**-3.75 (-4.22 to -3.24)**	**-5.56 (-8.07 to -3.21)**	**-4.50 (-5.56 to -3.46)**
Agar Malwa	113.2 (101.8-124.5)	86.3 (77.1-96.1)	50.8 (39.7-64.3)	-2.66 (-3.56 to -1.75)	-7.41 (-10.34 to -4.42)	-4.65 (-6.00 to -3.31)
Alirajpur	104.7 (94.3-116.2)	84.1 (75.2-94.0)	55.0 (42.5-68.8)	-2.18 (-3.24 to -1.01)	-5.99 (-9.11 to -2.74)	-3.77 (-5.17 to -2.48)
Anuppur	111.5 (101.0-123.0)	77.1 (68.9-86.1)	55.0 (42.7-68.5)	-3.62 (-4.56 to -2.62)	-4.79 (-7.83 to -1.76)	-4.11 (-5.49 to -2.82)
Ashoknagar	119.6 (107.8-131.6)	85.2 (76.3-94.5)	56.5 (44.1-69.8)	-3.36 (-4.21 to -2.44)	-5.77 (-8.54 to -3.06)	-4.36 (-5.64 to -3.14)
Balaghat	97.9 (88.3-108.8)	66.0 (59.0-74.1)	44.3 (34.5-56.3)	-3.87 (-4.97 to -2.81)	-5.61 (-8.67 to -2.39)	-4.59 (-5.88 to -3.18)
Barwani	86.3 (76.5-96.2)	68.2 (59.9-76.6)	45.2 (34.9-57.2)	-2.33 (-3.54 to -1.11)	-5.79 (-8.94 to -2.48)	-3.78 (-5.24 to -2.43)
Betul	83.9 (74.4-94.7)	56.0 (48.9-63.7)	36.4 (27.6-46.7)	-3.96 (-5.15 to -2.75)	-6.06 (-9.44 to -2.89)	-4.83 (-6.30 to -3.40)
Bhind	106.0 (96.9-115.4)	75.2 (68.6-82.4)	52.4 (41.9-65.7)	-3.36 (-4.09 to -2.64)	-5.13 (-7.96 to -2.38)	-4.10 (-5.31 to -2.96)
Bhopal	78.7 (69.4-88.6)	51.9 (45.0-59.1)	33.8 (25.8-43.5)	-4.07 (-5.18 to -2.90)	-6.00 (-9.16 to -2.78)	-4.87 (-6.42 to -3.43)
Burhanpur	85.8 (75.2-96.4)	65.3 (57.9-73.7)	43.5 (33.2-55.7)	-2.71 (-3.93 to -1.49)	-5.71 (-9.17 to -2.47)	-3.96 (-5.38 to -2.57)
Chhatarpur	149.3 (134.7-163.2)	96.2 (86.5-105.8)	67.8 (54.3-83.3)	-4.30 (-5.09 to -3.45)	-4.98 (-7.95 to -2.17)	-4.58 (-5.80 to -3.41)
Chhindwara	85.0 (76.1-94.8)	54.7 (48.2-61.5)	36.7 (28.0-47.1)	-4.31 (-5.38 to -3.25)	-5.61 (-8.89 to -2.59)	-4.85 (-6.23 to -3.49)
Damoh	152.5 (138.6-167.9)	89.2 (79.6-99.4)	69.0 (54.1-86.0)	-5.22 (-6.14 to -4.31)	-3.66 (-6.84 to -0.56)	-4.59 (-5.91 to -3.34)
Datia	111.6 (101.4-122.3)	72.1 (65.5-79.1)	51.9 (41.1-64.3)	-4.24 (-5.06 to -3.45)	-4.69 (-7.57 to -1.77)	-4.43 (-5.69 to -3.17)
Dewas	87.6 (79.1-96.9)	58.9 (52.5-66.1)	37.3 (28.6-47.0)	-3.89 (-4.84 to -2.94)	-6.41 (-9.41 to -3.26)	-4.94 (-6.33 to -3.65)
Dhar	90.3 (80.9-99.7)	65.0 (58.0-72.5)	42.6 (32.8-53.4)	-3.24 (-4.39 to -2.17)	-5.94 (-9.23 to -2.75)	-4.37 (-5.83 to -3.04)
Dindori	130.5 (119.2-142.7)	87.8 (79.1-97.9)	63.9 (50.1-80.4)	-3.87 (-4.82 to -2.99)	-4.51 (-7.50 to -1.35)	-4.14 (-5.41 to -2.79)
Guna	117.5 (105.9-130.3)	89.2 (79.9-99.1)	55.2 (43.7-69.9)	-2.73 (-3.66 to -1.72)	-6.68 (-9.46 to -3.76)	-4.38 (-5.67 to -3.05)
Gwalior	105.2 (96.0-115.2)	73.3 (66.7-80.2)	52.4 (41.5-65.1)	-3.54 (-4.30 to -2.70)	-4.75 (-7.63 to -1.85)	-4.05 (-5.32 to -2.81)
Harda	101.2 (90.2-113.5)	67.7 (59.4-76.4)	44.6 (33.5-57.8)	-3.95 (-5.15 to -2.73)	-5.86 (-9.19 to -2.34)	-4.75 (-6.35 to -3.32)
Hoshangabad	91.6 (83.2-100.4)	58.4 (51.8-65.3)	38.1 (29.5-48.1)	-4.40 (-5.41 to -3.39)	-5.99 (-9.20 to -2.92)	-5.06 (-6.48 to -3.76)
Indore	75.4 (67.8-83.3)	48.1 (42.8-53.9)	31.0 (23.8-38.8)	-4.39 (-5.44 to -3.32)	-6.18 (-9.32 to -3.03)	-5.13 (-6.58 to -3.78)
Jabalpur	128.6 (115.9-143.0)	74.4 (66.4-83.3)	54.4 (41.9-68.3)	-5.33 (-6.39 to -4.34)	-4.45 (-7.74 to -1.30)	-4.97 (-6.32 to -3.63)
Jhabua	107.7 (96.2-120.0)	83.0 (74.0-92.3)	50.4 (38.9-63.3)	-2.58 (-3.69 to -1.48)	-6.94 (-10.10 to -3.78)	-4.41 (-5.83 to -3.06)
Katni	152.0 (139.5-166.2)	93.0 (84.0-103.7)	69.7 (54.8-86.0)	-4.80 (-5.64 to -3.91)	-4.10 (-7.16 to -1.20)	-4.52 (-5.80 to -3.24)
Khandwa	100.0 (89.7-110.9)	70.0 (62.4-78.5)	47.0 (35.7-59.9)	-3.49 (-4.57 to -2.31)	-5.62 (-9.00 to -2.14)	-4.38 (-5.89 to -3.03)
Khargone	93.5 (83.1-103.4)	67.6 (59.9-75.5)	45.1 (34.6-57.0)	-3.20 (-4.32 to -2.13)	-5.67 (-8.94 to -2.44)	-4.23 (-5.72 to -2.91)
Mandla	122.1 (111.0-134.2)	78.3 (70.3-87.4)	55.6 (43.2-69.7)	-4.39 (-5.41 to -3.47)	-4.83 (-7.83 to -1.68)	-4.58 (-5.93 to -3.21)
Mandsaur	110.4 (100.5-121.2)	87.2 (78.4-96.7)	47.9 (38.1-60.0)	-2.30 (-3.20 to -1.35)	-8.33 (-11.09 to -5.43)	-4.84 (-6.06 to -3.53)
Morena	109.2 (99.2-119.7)	85.0 (77.0-93.2)	58.0 (45.8-71.9)	-2.46 (-3.30 to -1.55)	-5.40 (-8.16 to -2.49)	-3.69 (-4.93 to -2.48)
Narsinghpur	107.9 (97.1-119.9)	66.1 (58.8-74.0)	45.6 (35.4-57.6)	-4.77 (-5.80 to -3.81)	-5.25 (-8.60 to -2.14)	-4.97 (-6.38 to -3.62)
Neemuch	114.3 (104.5-124.9)	92.9 (84.0-102.4)	48.7 (39.2-60.4)	-2.01 (-2.86 to -1.11)	-9.02 (-11.63 to -6.30)	-4.96 (-6.11 to -3.73)
Niwari	123.7 (111.3-136.5)	77.2 (69.3-85.6)	54.2 (42.5-67.7)	-4.58 (-5.53 to -3.70)	-5.12 (-8.13 to -2.15)	-4.81 (-6.10 to -3.52)
Panna	163.3 (147.4-179.6)	103.8 (93.2-116.0)	74.8 (59.0-92.9)	-4.43 (-5.30 to -3.49)	-4.66 (-7.71 to -1.74)	-4.53 (-5.75 to -3.31)
Raisen	100.7 (90.9-111.0)	65.7 (58.3-73.5)	44.6 (34.4-56.1)	-4.17 (-5.16 to -3.14)	-5.48 (-8.79 to -2.47)	-4.72 (-6.19 to -3.40)
Rajgarh	110.9 (100.2-121.5)	84.4 (75.6-94.1)	50.6 (39.8-63.7)	-2.70 (-3.59 to -1.77)	-7.10 (-9.99 to -4.07)	-4.54 (-5.88 to -3.23)
Ratlam	94.1 (84.9-103.6)	69.5 (62.2-76.8)	40.2 (31.5-50.6)	-2.98 (-3.91 to -2.04)	-7.63 (-10.69 to -4.52)	-4.93 (-6.23 to -3.61)
Rewa	138.3 (125.2-150.9)	93.2 (84.2-102.4)	63.1 (49.2-79.1)	-3.88 (-4.72 to -2.98)	-5.49 (-8.58 to -2.66)	-4.55 (-5.81 to -3.25)
Sagar	133.6 (120.5-147.5)	85.3 (75.5-95.2)	59.9 (46.3-75.3)	-4.39 (-5.32 to -3.42)	-4.99 (-8.13 to -2.10)	-4.64 (-6.04 to -3.44)
Satna	147.8 (135.1-161.7)	94.7 (85.3-105.0)	65.3 (51.3-81.4)	-4.35 (-5.18 to -3.46)	-5.26 (-8.15 to -2.47)	-4.73 (-6.04 to -3.50)
Sehore	89.2 (80.4-98.8)	60.3 (53.8-67.4)	38.7 (29.9-49.2)	-3.84 (-4.86 to -2.90)	-6.21 (-9.31 to -3.10)	-4.83 (-6.27 to -3.51)

Seoni	98.2 (88.4-109.3)	61.6 (54.8-69.4)	42.6 (32.7-54.4)	-4.56 (-5.58 to -3.55)	-5.21 (-8.33 to -2.01)	-4.83 (-6.23 to -3.47)
Shahdol	131.1 (119.3-143.9)	88.5 (79.4-98.5)	65.3 (50.9-82.1)	-3.85 (-4.76 to -2.92)	-4.33 (-7.39 to -1.22)	-4.05 (-5.43 to -2.71)
Shajapur	90.0 (81.1-98.7)	65.0 (58.2-72.4)	40.1 (31.3-50.3)	-3.20 (-4.11 to -2.31)	-6.75 (-9.70 to -3.67)	-4.68 (-6.05 to -3.40)
Sheopur	139.0 (126.8-151.1)	112.2 (101.6-123.6)	72.3 (57.8-89.9)	-2.10 (-2.92 to -1.30)	-6.20 (-8.76 to -3.48)	-3.81 (-4.96 to -2.61)
Shivpuri	123.2 (112.0-134.9)	85.3 (77.2-93.7)	58.5 (46.3-72.4)	-3.62 (-4.45 to -2.76)	-5.31 (-7.96 to -2.53)	-4.32 (-5.55 to -3.13)
Sidhi	135.4 (122.6-149.9)	93.3 (83.7-103.8)	66.6 (52.2-83.3)	-3.65 (-4.58 to -2.67)	-4.78 (-7.84 to -1.77)	-4.12 (-5.45 to -2.79)
Singrauli	127.3 (113.1-143.8)	90.6 (79.6-102.9)	63.3 (49.2-79.7)	-3.34 (-4.29 to -2.33)	-5.08 (-8.00 to -2.12)	-4.07 (-5.40 to -2.84)
Tikamgarh	140.8 (127.6-153.6)	91.9 (83.1-101.0)	64.8 (51.3-79.9)	-4.17 (-4.97 to -3.36)	-4.98 (-7.85 to -2.25)	-4.51 (-5.72 to -3.34)
Ujjain	81.0 (72.9-90.0)	56.3 (49.9-63.4)	34.8 (26.9-44.1)	-3.57 (-4.64 to -2.58)	-6.73 (-9.87 to -3.62)	-4.89 (-6.27 to -3.54)
Umaria	148.1 (134.3-163.7)	95.9 (85.8-107.7)	71.4 (55.5-90.7)	-4.25 (-5.20 to -3.30)	-4.20 (-7.25 to -0.99)	-4.24 (-5.59 to -2.89)
Vidisha	114.8 (101.8-128.5)	83.1 (72.3-94.6)	55.4 (42.6-70.1)	-3.18 (-4.19 to -2.14)	-5.69 (-8.64 to -2.74)	-4.23 (-5.59 to -2.95)
**Jharkhand**	**87.2 (78.4-96.0)**	**60.1 (54.2-66.7)**	**43.9 (37.9-51.2)**	**-3.66 (-4.32 to -3.01)**	**-4.44 (-6.97 to -1.73)**	**-3.99 (-5.00 to -2.89)**
Bokaro	83.2 (75.4-91.4)	57.8 (52.5-63.9)	39.4 (32.0-48.9)	-3.58 (-4.46 to -2.69)	-5.36 (-8.14 to -2.55)	-4.32 (-5.49 to -3.06)
Chatra	87.8 (79.9-96.2)	60.5 (54.8-66.5)	47.2 (38.0-58.5)	-3.67 (-4.50 to -2.80)	-3.44 (-6.16 to -0.56)	-3.58 (-4.71 to -2.40)
Deoghar	85.1 (77.3-93.1)	58.9 (53.3-64.7)	44.3 (35.7-55.0)	-3.60 (-4.51 to -2.73)	-4.04 (-7.01 to -1.20)	-3.79 (-4.99 to -2.59)
Dhanbad	84.2 (75.9-92.7)	58.5 (52.7-64.6)	41.3 (33.1-51.5)	-3.57 (-4.50 to -2.66)	-4.91 (-7.77 to -2.08)	-4.13 (-5.35 to -2.86)
Dumka	88.4 (80.1-97.2)	57.5 (51.4-63.8)	45.0 (36.4-56.1)	-4.22 (-5.14 to -3.32)	-3.48 (-6.38 to -0.64)	-3.92 (-5.14 to -2.71)
East Singhbhum	69.4 (61.6-77.7)	47.4 (41.8-53.4)	32.3 (25.2-41.2)	-3.75 (-4.92 to -2.58)	-5.37 (-8.51 to -2.12)	-4.43 (-5.78 to -3.06)
Garhwa	91.5 (82.5-102.0)	63.2 (56.2-70.9)	50.1 (39.7-62.8)	-3.63 (-4.65 to -2.58)	-3.32 (-6.31 to -0.33)	-3.51 (-4.70 to -2.20)
Giridih	81.4 (73.4-90.0)	57.6 (52.3-63.7)	42.5 (34.2-52.9)	-3.41 (-4.29 to -2.49)	-4.29 (-7.18 to -1.40)	-3.78 (-5.00 to -2.59)
Godda	89.0 (80.6-98.9)	59.8 (53.5-66.7)	47.0 (37.5-58.9)	-3.90 (-4.90 to -2.89)	-3.45 (-6.38 to -0.41)	-3.72 (-4.99 to -2.49)
Gumla	105.9 (96.2-116.4)	74.0 (66.9-81.7)	52.6 (42.1-64.7)	-3.52 (-4.42 to -2.57)	-4.80 (-7.62 to -1.86)	-4.05 (-5.22 to -2.81)
Hazaribagh	83.0 (75.6-91.1)	55.8 (50.5-61.9)	40.9 (32.6-50.6)	-3.87 (-4.74 to -2.99)	-4.38 (-7.10 to -1.46)	-4.08 (-5.26 to -2.89)
Jamtara	89.9 (80.0-99.8)	61.9 (55.2-69.4)	47.1 (37.0-58.6)	-3.66 (-4.69 to -2.62)	-3.91 (-6.87 to -1.03)	-3.77 (-5.05 to -2.48)
Khunti	101.4 (90.9-113.0)	71.4 (63.9-79.3)	48.1 (38.1-60.3)	-3.45 (-4.37 to -2.49)	-5.53 (-8.38 to -2.64)	-4.31 (-5.52 to -3.05)
Koderma	76.7 (69.5-84.0)	52.4 (47.5-57.7)	40.3 (32.5-50.0)	-3.72 (-4.57 to -2.88)	-3.74 (-6.67 to -0.86)	-3.73 (-4.89 to -2.57)
Latehar	97.1 (88.1-107.1)	69.5 (62.5-76.6)	52.1 (41.9-64.4)	-3.28 (-4.21 to -2.34)	-4.11 (-6.79 to -1.12)	-3.63 (-4.80 to -2.38)
Lohardaga	103.8 (92.7-115.8)	73.1 (65.1-81.3)	51.4 (40.9-64.6)	-3.44 (-4.48 to -2.36)	-4.97 (-7.79 to -1.90)	-4.08 (-5.31 to -2.76)
Pakur	88.0 (80.0-96.8)	56.6 (50.8-63.0)	45.1 (36.0-55.9)	-4.33 (-5.22 to -3.36)	-3.22 (-6.13 to -0.24)	-3.88 (-5.04 to -2.67)
Palamu	91.9 (83.5-101.3)	64.2 (58.0-70.7)	49.8 (40.2-62.6)	-3.53 (-4.45 to -2.64)	-3.60 (-6.40 to -0.72)	-3.57 (-4.76 to -2.31)
Ramgarh	84.0 (76.3-92.3)	56.6 (51.4-62.9)	37.8 (30.3-47.2)	-3.88 (-4.75 to -2.95)	-5.64 (-8.33 to -2.64)	-4.61 (-5.81 to -3.35)
Ranchi	92.0 (82.9-101.7)	62.9 (56.6-69.7)	42.2 (33.7-52.8)	-3.74 (-4.65 to -2.77)	-5.60 (-8.37 to -2.70)	-4.51 (-5.70 to -3.22)
Sahibganj	91.0 (82.9-99.5)	60.0 (54.3-66.6)	46.8 (37.2-58.2)	-4.09 (-4.95 to -3.19)	-3.56 (-6.35 to -0.57)	-3.87 (-5.09 to -2.70)
Saraikela Kharsawan	83.0 (74.5-91.6)	57.6 (51.4-64.2)	38.8 (30.4-49.1)	-3.58 (-4.59 to -2.53)	-5.55 (-8.57 to -2.43)	-4.40 (-5.68 to -3.08)
Simdega	105.0 (95.0-115.0)	70.8 (63.5-78.6)	51.5 (41.3-63.7)	-3.87 (-4.81 to -2.90)	-4.48 (-7.44 to -1.39)	-4.13 (-5.32 to -2.87)
West Singhbhum	97.1 (88.0-107.4)	70.4 (63.5-77.7)	49.8 (39.5-62.5)	-3.18 (-4.09 to -2.25)	-4.86 (-7.78 to -1.86)	-3.88 (-5.11 to -2.59)
**Uttar Pradesh**	**112.8 (101.8-125.0)**	**81.8 (74.3-89.7)**	**59.7 (51.8-69.7)**	**-3.16 (-3.61 to -2.74)**	**-4.43 (-6.60 to -2.14)**	**-3.69 (-4.56 to -2.73)**
Agra	104.7 (95.9-113.5)	81.6 (74.2-88.4)	58.0 (46.7-71.1)	-2.46 (-3.20 to -1.73)	-4.81 (-7.58 to -2.09)	-3.44 (-4.57 to -2.31)
Aligarh	113.4 (104.2-122.7)	91.4 (83.1-99.4)	67.0 (54.5-82.1)	-2.12 (-2.83 to -1.38)	-4.40 (-7.01 to -1.71)	-3.07 (-4.11 to -1.99)
Allahabad	130.5 (118.9-142.8)	88.5 (80.1-96.9)	65.6 (51.4-82.5)	-3.81 (-4.74 to -2.91)	-4.21 (-7.33 to -1.04)	-3.98 (-5.30 to -2.63)
Ambedkar Nagar	95.2 (86.8-103.8)	61.4 (55.1-68.1)	45.5 (36.3-57.4)	-4.31 (-5.20 to -3.44)	-4.22 (-7.18 to -1.09)	-4.28 (-5.47 to -3.04)
Amethi	116.4 (105.0-129.3)	78.6 (70.2-87.8)	60.1 (48.1-76.6)	-3.87 (-4.75 to -3.04)	-3.81 (-6.68 to -0.93)	-3.85 (-5.07 to -2.59)
Amroha	108.6 (98.1-119.5)	89.9 (80.4-99.0)	63.2 (51.1-78.1)	-1.90 (-2.80 to -0.98)	-4.92 (-7.51 to -2.13)	-3.16 (-4.28 to -2.06)
Auraiya	108.9 (99.7-118.2)	75.6 (68.9-83.4)	55.0 (44.7-68.2)	-3.58 (-4.41 to -2.77)	-4.48 (-7.19 to -1.78)	-3.95 (-5.12 to -2.80)
Azamgarh	97.0 (89.1-105.4)	66.9 (60.9-73.5)	46.2 (36.5-57.2)	-3.65 (-4.44 to -2.87)	-5.20 (-8.05 to -2.19)	-4.30 (-5.51 to -3.06)
Baghpat	91.4 (82.8-100.9)	67.6 (60.8-75.2)	49.4 (39.8-60.5)	-2.97 (-3.90 to -2.12)	-4.43 (-7.10 to -1.63)	-3.58 (-4.73 to -2.45)
Bahraich	138.9 (128.1-151.7)	102.3 (93.2-112.2)	80.5 (65.3-100.5)	-3.05 (-3.89 to -2.27)	-3.42 (-6.25 to -0.52)	-3.21 (-4.35 to -1.94)
Ballia	89.6 (82.1-97.5)	63.6 (57.9-69.8)	43.0 (34.1-53.6)	-3.37 (-4.15 to -2.55)	-5.51 (-8.33 to -2.46)	-4.26 (-5.49 to -3.03)
Balrampur	136.6 (125.3-149.7)	94.6 (85.8-104.5)	74.1 (59.9-93.8)	-3.58 (-4.50 to -2.68)	-3.48 (-6.40 to -0.36)	-3.54 (-4.71 to -2.24)
Banda	129.7 (118.3-141.2)	86.5 (77.9-95.3)	64.8 (51.6-80.3)	-3.99 (-4.85 to -3.18)	-4.05 (-6.97 to -1.20)	-4.02 (-5.25 to -2.81)
Barabanki	118.7 (109.1-129.7)	86.1 (78.0-94.7)	65.3 (52.6-82.1)	-3.19 (-4.08 to -2.42)	-3.91 (-6.66 to -1.01)	-3.49 (-4.68 to -2.24)
Bareilly	122.2 (111.0-133.7)	100.2 (90.7-110.2)	70.9 (58.1-86.5)	-1.95 (-2.76 to -1.12)	-4.87 (-7.48 to -2.28)	-3.17 (-4.27 to -2.08)
Basti	109.5 (101.3-118.7)	70.4 (64.3-77.1)	54.5 (43.6-69.1)	-4.33 (-5.16 to -3.56)	-3.68 (-6.58 to -0.46)	-4.07 (-5.23 to -2.78)
Bhadohi	122.4 (112.0-133.0)	84.3 (76.4-92.3)	60.8 (48.3-75.8)	-3.67 (-4.51 to -2.80)	-4.62 (-7.56 to -1.45)	-4.06 (-5.28 to -2.77)
Bijnor	97.3 (88.5-106.4)	78.2 (70.3-85.9)	55.0 (45.0-66.8)	-2.16 (-3.00 to -1.28)	-4.96 (-7.44 to -2.27)	-3.33 (-4.38 to -2.26)
Budaun	135.7 (123.3-147.4)	119.5 (108.3-130.7)	87.9 (72.2-106.4)	-1.25 (-2.00 to -0.47)	-4.34 (-6.87 to -1.69)	-2.54 (-3.63 to -1.46)
Bulandshahr	110.7 (101.4-120.0)	88.8 (80.3-96.9)	64.2 (52.5-78.8)	-2.19 (-2.90 to -1.42)	-4.59 (-7.12 to -1.90)	-3.19 (-4.24 to -2.10)
Chandauli	104.4 (95.8-114.0)	73.7 (67.0-81.0)	51.8 (41.3-64.5)	-3.43 (-4.23 to -2.58)	-4.91 (-7.78 to -1.90)	-4.04 (-5.30 to -2.74)
Chitrakoot	136.6 (124.4-149.2)	91.8 (82.5-101.3)	69.8 (54.9-87.0)	-3.90 (-4.76 to -3.04)	-3.88 (-6.82 to -1.13)	-3.89 (-5.14 to -2.65)
Deoria	91.9 (84.4-100.0)	61.7 (56.0-67.8)	41.6 (32.8-52.1)	-3.91 (-4.75 to -3.05)	-5.55 (-8.46 to -2.50)	-4.59 (-5.86 to -3.37)
Etah	128.8 (117.2-140.5)	102.1 (92.5-111.6)	75.2 (60.7-93.3)	-2.30 (-3.16 to -1.49)	-4.33 (-7.08 to -1.57)	-3.14 (-4.33 to -1.92)
Etawah	115.3 (105.9-125.3)	82.2 (74.7-89.8)	59.0 (47.8-72.7)	-3.31 (-4.07 to -2.56)	-4.72 (-7.48 to -2.00)	-3.90 (-5.07 to -2.74)
Faizabad	111.8 (102.3-122.4)	73.9 (66.8-81.7)	56.4 (45.1-71.5)	-4.18 (-5.03 to -3.38)	-3.87 (-6.78 to -0.82)	-4.05 (-5.27 to -2.76)
Farrukhabad	127.3 (116.2-138.0)	101.3 (91.8-110.7)	74.5 (60.9-91.8)	-2.26 (-3.04 to -1.50)	-4.33 (-6.89 to -1.68)	-3.12 (-4.22 to -1.99)
Fatehpur	120.6 (109.9-132.0)	81.5 (73.4-90.5)	59.8 (47.7-74.7)	-3.85 (-4.71 to -3.00)	-4.39 (-7.28 to -1.53)	-4.08 (-5.25 to -2.86)
Firozabad	122.0 (111.2-133.1)	94.1 (85.1-102.7)	68.7 (55.0-84.7)	-2.55 (-3.40 to -1.71)	-4.47 (-7.36 to -1.69)	-3.35 (-4.59 to -2.13)
Gautam Buddha Nagar	84.2 (77.2-91.2)	64.1 (57.9-70.1)	45.7 (36.9-56.2)	-2.63 (-3.42 to -1.83)	-4.71 (-7.32 to -1.98)	-3.50 (-4.63 to -2.43)
Ghaziabad	83.3 (75.9-91.2)	62.6 (56.5-69.1)	44.5 (36.0-54.8)	-2.86 (-3.69 to -2.01)	-4.78 (-7.40 to -2.05)	-3.66 (-4.78 to -2.55)
Ghazipur	98.9 (90.1-108.7)	70.7 (63.8-78.0)	48.9 (38.6-61.3)	-3.29 (-4.14 to -2.37)	-5.21 (-8.11 to -2.11)	-4.09 (-5.36 to -2.77)
Gonda	127.5 (116.8-139.8)	89.3 (80.9-97.9)	69.9 (56.4-88.3)	-3.48 (-4.34 to -2.60)	-3.49 (-6.42 to -0.27)	-3.49 (-4.68 to -2.15)
Gorakhpur	95.0 (87.0-104.2)	63.4 (57.2-70.0)	43.9 (34.3-56.1)	-3.96 (-4.89 to -3.03)	-5.21 (-8.21 to -2.05)	-4.48 (-5.80 to -3.18)
Hamirpur	113.1 (103.0-123.7)	71.4 (64.6-78.5)	53.8 (42.7-66.4)	-4.48 (-5.34 to -3.64)	-4.02 (-6.87 to -1.18)	-4.30 (-5.45 to -3.12)
Hapur	104.3 (95.6-113.7)	81.2 (73.1-88.5)	57.3 (46.7-70.4)	-2.48 (-3.26 to -1.69)	-4.91 (-7.43 to -2.26)	-3.49 (-4.59 to -2.44)
Hardoi	128.0 (117.6-139.0)	99.5 (90.5-109.7)	74.3 (61.4-92.2)	-2.48 (-3.28 to -1.72)	-4.13 (-6.68 to -1.50)	-3.17 (-4.24 to -2.05)
Hathras	118.6 (108.7-128.9)	91.0 (82.7-99.0)	66.9 (53.9-81.7)	-2.61 (-3.40 to -1.87)	-4.37 (-7.05 to -1.65)	-3.34 (-4.45 to -2.21)
Jalaun	101.3 (91.9-112.1)	64.8 (58.0-72.0)	50.3 (39.6-63.3)	-4.37 (-5.34 to -3.45)	-3.60 (-6.47 to -0.68)	-4.06 (-5.27 to -2.79)
Jaunpur	110.7 (101.6-119.9)	76.2 (69.3-83.3)	54.7 (43.9-68.2)	-3.68 (-4.45 to -2.91)	-4.68 (-7.58 to -1.70)	-4.09 (-5.23 to -2.83)
Jhansi	112.3 (102.0-123.2)	69.2 (62.6-76.3)	55.8 (44.2-69.7)	-4.74 (-5.61 to -3.96)	-3.03 (-5.93 to -0.04)	-4.04 (-5.27 to -2.81)
Kannauj	117.6 (106.9-128.8)	87.5 (79.0-97.4)	63.9 (51.9-79.3)	-2.90 (-3.85 to -2.05)	-4.47 (-7.11 to -1.75)	-3.55 (-4.67 to -2.35)
Kanpur Dehat	106.8 (97.6-116.0)	69.8 (63.6-76.9)	51.4 (41.2-64.0)	-4.14 (-5.04 to -3.31)	-4.36 (-7.02 to -1.53)	-4.23 (-5.36 to -3.02)
Kanpur Nagar	101.4 (92.2-111.3)	66.5 (60.2-73.9)	46.6 (37.1-57.5)	-4.14 (-5.06 to -3.27)	-5.01 (-7.75 to -2.23)	-4.51 (-5.64 to -3.29)
Kasganj	130.6 (119.6-141.9)	111.4 (101.4-121.5)	82.5 (67.3-101.2)	-1.57 (-2.36 to -0.76)	-4.24 (-6.94 to -1.54)	-2.68 (-3.83 to -1.50)
Kaushambi	132.6 (121.1-144.0)	92.6 (84.1-101.4)	69.5 (55.3-87.2)	-3.62 (-4.45 to -2.74)	-4.33 (-7.36 to -1.39)	-3.91 (-5.14 to -2.66)
Khushinagar	96.8 (88.2-106.2)	66.4 (60.1-74.3)	45.6 (35.6-58.0)	-3.70 (-4.64 to -2.70)	-5.28 (-8.50 to -1.98)	-4.36 (-5.68 to -3.07)
Lakhimpur Kheri	146.2 (132.3-160.1)	100.6 (90.8-111.6)	75.3 (61.1-93.7)	-3.69 (-4.58 to -2.82)	-4.12 (-6.76 to -1.47)	-3.87 (-5.01 to -2.69)
Lalitpur	125.2 (112.8-137.5)	80.5 (72.4-88.4)	65.1 (51.0-80.2)	-4.34 (-5.12 to -3.50)	-2.97 (-5.91 to -0.27)	-3.78 (-5.07 to -2.56)
Lucknow	103.1 (94.1-114.1)	69.8 (62.6-77.1)	50.0 (40.1-62.9)	-3.81 (-4.71 to -2.98)	-4.68 (-7.37 to -1.88)	-4.18 (-5.35 to -2.92)
Maharajganj	107.1 (97.5-118.2)	70.6 (63.8-78.5)	51.1 (39.7-64.7)	-4.11 (-5.08 to -3.11)	-4.55 (-7.69 to -1.23)	-4.29 (-5.64 to -2.94)

Mahoba	126.4 (114.5-137.8)	78.9 (71.0-86.8)	61.3 (48.6-75.4)	-4.64 (-5.50 to -3.83)	-3.52 (-6.40 to -0.81)	-4.18 (-5.32 to -2.99)
Mainpuri	123.8 (112.8-134.5)	94.5 (85.9-103.3)	67.6 (54.7-84.0)	-2.67 (-3.53 to -1.86)	-4.70 (-7.43 to -1.95)	-3.51 (-4.69 to -2.31)
Mathura	104.5 (95.3-113.9)	82.5 (74.5-90.0)	60.1 (48.3-73.9)	-2.34 (-3.15 to -1.52)	-4.45 (-7.27 to -1.72)	-3.22 (-4.34 to -2.05)
Mau	94.7 (86.7-103.1)	65.5 (59.6-71.9)	43.5 (34.3-54.2)	-3.62 (-4.42 to -2.79)	-5.73 (-8.57 to -2.72)	-4.49 (-5.74 to -3.23)
Meerut	97.3 (88.7-106.8)	72.2 (65.0-79.2)	52.3 (42.7-64.0)	-2.94 (-3.75 to -2.09)	-4.56 (-7.09 to -1.86)	-3.61 (-4.68 to -2.54)
Mirzapur	119.5 (109.6-130.2)	84.1 (76.5-91.8)	62.7 (50.4-77.8)	-3.47 (-4.28 to -2.66)	-4.16 (-6.98 to -1.21)	-3.75 (-4.99 to -2.46)
Moradabad	110.0 (99.7-121.0)	94.9 (84.8-104.2)	65.0 (52.7-80.3)	-1.47 (-2.38 to -0.51)	-5.31 (-7.91 to -2.54)	-3.08 (-4.19 to -1.98)
Muzaffarnagar	98.8 (90.1-107.9)	74.7 (67.3-82.3)	54.0 (44.1-65.6)	-2.76 (-3.62 to -1.90)	-4.58 (-7.15 to -1.93)	-3.51 (-4.62 to -2.44)
Pilibhit	118.2 (107.3-128.7)	86.5 (78.5-94.8)	61.2 (50.4-75.2)	-2.97 (-3.77 to -2.17)	-4.89 (-7.50 to -2.37)	-3.77 (-4.91 to -2.65)
Pratapgarh	121.9 (111.4-132.0)	81.4 (74.0-89.1)	60.6 (48.2-75.5)	-3.96 (-4.75 to -3.17)	-4.20 (-7.23 to -1.22)	-4.06 (-5.22 to -2.81)
Rae Bareli	113.7 (104.1-124.4)	76.5 (69.3-84.6)	57.5 (46.3-72.2)	-3.87 (-4.76 to -3.08)	-4.16 (-7.00 to -1.41)	-3.99 (-5.13 to -2.75)
Rampur	107.3 (98.0-117.4)	90.1 (81.3-98.6)	62.3 (50.7-76.6)	-1.72 (-2.51 to -0.79)	-5.20 (-7.75 to -2.55)	-3.17 (-4.26 to -2.07)
Saharanpur	90.8 (82.1-100.8)	71.1 (63.3-79.3)	51.5 (41.3-63.8)	-2.41 (-3.43 to -1.35)	-4.57 (-7.38 to -1.70)	-3.31 (-4.55 to -2.17)
Sambhal	122.9 (111.7-134.8)	108.5 (97.8-118.6)	78.2 (63.5-96.3)	-1.25 (-2.11 to -0.35)	-4.65 (-7.17 to -1.88)	-2.67 (-3.77 to -1.57)
Sant Kabir Nagar	102.4 (94.2-111.5)	67.3 (61.3-74.0)	49.6 (39.1-62.6)	-4.12 (-4.96 to -3.25)	-4.37 (-7.42 to -1.09)	-4.22 (-5.51 to -2.97)
Shahjahanpur	139.7 (127.2-152.2)	108.7 (98.6-119.3)	78.8 (65.0-96.1)	-2.50 (-3.27 to -1.68)	-4.55 (-7.07 to -1.95)	-3.35 (-4.39 to -2.25)
Shamli	96.3 (87.8-105.1)	73.0 (66.0-80.9)	52.6 (42.6-64.4)	-2.73 (-3.58 to -1.86)	-4.62 (-7.23 to -1.95)	-3.52 (-4.64 to -2.42)
Shravasti	139.6 (127.2-153.8)	102.3 (92.7-112.5)	80.8 (65.4-101.4)	-2.94 (-3.92 to -2.07)	-3.41 (-6.24 to -0.35)	-3.14 (-4.33 to -1.83)
Siddharth Nagar	124.0 (114.5-135.4)	80.4 (73.3-88.5)	61.6 (49.4-77.9)	-4.22 (-5.05 to -3.39)	-3.75 (-6.78 to -0.50)	-4.03 (-5.31 to -2.75)
Sitapur	133.6 (122.5-145.8)	98.4 (89.1-108.7)	74.5 (61.0-92.4)	-3.01 (-3.82 to -2.24)	-3.96 (-6.64 to -1.15)	-3.41 (-4.54 to -2.23)
Sonbhadra	108.5 (98.5-120.2)	77.4 (69.7-85.5)	57.1 (45.9-71.0)	-3.31 (-4.26 to -2.38)	-4.32 (-7.20 to -1.50)	-3.73 (-5.02 to -2.48)
Sultanpur	107.0 (96.6-116.8)	68.9 (62.3-76.2)	51.8 (41.7-65.3)	-4.34 (-5.15 to -3.54)	-4.07 (-6.96 to -1.10)	-4.23 (-5.39 to -3.02)
Unnao	113.8 (104.4-123.8)	78.9 (71.7-86.7)	58.7 (47.4-72.5)	-3.60 (-4.47 to -2.84)	-4.19 (-6.94 to -1.53)	-3.84 (-4.95 to -2.65)
Varanasi	106.3 (97.7-115.6)	74.2 (67.6-81.2)	50.5 (40.2-63.2)	-3.51 (-4.30 to -2.72)	-5.43 (-8.36 to -2.40)	-4.31 (-5.51 to -3.00)
**Rajasthan**	**90.5 (81.0-100.7)**	**64.9 (59.1-71.0)**	**48.3 (41.8-56.4)**	**-3.26 (-3.82 to -2.72)**	**-4.19 (-6.53 to -1.84)**	**-3.65 (-4.59 to -2.69)**
Ajmer	93.8 (84.2-103.0)	65.4 (58.2-72.2)	46.3 (36.7-57.9)	-3.53 (-4.54 to -2.57)	-4.87 (-7.82 to -2.11)	-4.09 (-5.32 to -2.86)
Alwar	80.1 (73.0-87.4)	57.3 (51.7-62.8)	47.1 (37.3-57.9)	-3.33 (-4.14 to -2.53)	-2.80 (-5.70 to -0.04)	-3.12 (-4.26 to -1.91)
Banswara	104.8 (94.9-115.4)	72.6 (65.0-80.2)	49.6 (39.0-62.4)	-3.61 (-4.54 to -2.70)	-5.34 (-8.32 to -2.08)	-4.33 (-5.62 to -2.94)
Baran	117.1 (106.9-127.3)	78.4 (71.1-86.0)	57.9 (46.4-72.3)	-3.94 (-4.75 to -3.11)	-4.25 (-7.00 to -1.36)	-4.07 (-5.23 to -2.87)
Barmer	104.4 (92.9-116.3)	83.1 (74.1-92.8)	64.7 (51.4-80.3)	-2.26 (-3.34 to -1.13)	-3.57 (-6.49 to -0.63)	-2.80 (-4.17 to -1.54)
Bharatpur	95.9 (88.1-104.0)	69.6 (63.3-76.0)	55.9 (44.7-68.5)	-3.18 (-3.94 to -2.40)	-3.10 (-5.90 to -0.37)	-3.15 (-4.27 to -2.00)
Bhilwara	120.6 (109.9-131.9)	87.5 (79.0-95.5)	58.0 (46.4-71.4)	-3.16 (-4.06 to -2.32)	-5.76 (-8.45 to -2.99)	-4.24 (-5.41 to -3.04)
Bikaner	54.6 (48.0-61.2)	36.0 (31.8-40.7)	35.2 (27.2-45.0)	-4.09 (-5.23 to -2.75)	-0.39 (-3.55 to 2.90)	-2.59 (-4.00 to -1.11)
Bundi	121.9 (111.7-133.0)	86.6 (78.3-95.6)	60.4 (48.0-74.9)	-3.38 (-4.25 to -2.43)	-5.13 (-8.01 to -2.33)	-4.10 (-5.29 to -2.91)
Chittorgarh	131.9 (120.2-145.4)	97.3 (87.8-106.9)	62.3 (49.8-77.3)	-3.02 (-3.84 to -2.21)	-6.18 (-8.90 to -3.40)	-4.34 (-5.49 to -3.12)
Churu	52.6 (46.6-58.7)	35.2 (31.2-39.4)	30.0 (23.8-38.4)	-3.92 (-4.99 to -2.83)	-2.30 (-5.29 to 0.78)	-3.26 (-4.61 to -1.90)
Dausa	97.6 (87.9-107.3)	68.6 (61.2-76.0)	53.0 (42.2-65.2)	-3.48 (-4.32 to -2.57)	-3.65 (-6.57 to -0.94)	-3.55 (-4.72 to -2.32)
Dholpur	100.7 (92.0-109.9)	70.5 (64.1-77.2)	58.2 (46.5-72.0)	-3.54 (-4.35 to -2.66)	-2.68 (-5.54 to 0.25)	-3.19 (-4.40 to -2.01)
Dungarpur	117.5 (105.3-130.3)	86.3 (77.0-96.5)	56.4 (44.5-71.3)	-3.04 (-4.09 to -2.06)	-5.95 (-8.82 to -2.76)	-4.25 (-5.56 to -2.86)
Hanumangarh	53.3 (47.6-59.3)	33.6 (30.1-37.9)	30.8 (24.4-38.9)	-4.53 (-5.56 to -3.42)	-1.27 (-4.20 to 1.97)	-3.20 (-4.51 to -1.89)
Jaipur	69.5 (62.7-76.2)	45.9 (41.1-50.9)	35.6 (28.6-44.1)	-4.06 (-4.94 to -3.13)	-3.62 (-6.48 to -0.94)	-3.88 (-5.11 to -2.74)
Jaisalmer	73.2 (63.7-83.3)	52.7 (45.8-59.6)	49.8 (38.5-63.4)	-3.23 (-4.51 to -1.88)	-0.85 (-4.16 to 2.70)	-2.27 (-3.81 to -0.88)
Jalore	116.2 (104.1-128.4)	93.8 (83.7-104.4)	67.0 (53.3-84.0)	-2.11 (-3.18 to -0.98)	-4.76 (-7.80 to -1.79)	-3.22 (-4.51 to -1.95)
Jhalawar	117.6 (106.4-128.7)	79.9 (71.7-88.1)	56.2 (44.4-70.6)	-3.82 (-4.65 to -2.91)	-4.88 (-7.81 to -1.90)	-4.26 (-5.53 to -2.99)
Jhunjhunu	58.7 (52.5-64.8)	38.5 (34.4-42.9)	30.3 (24.0-37.9)	-4.13 (-5.10 to -3.11)	-3.41 (-6.21 to -0.42)	-3.84 (-5.09 to -2.56)
Jodhpur	68.2 (60.5-76.2)	51.7 (45.9-57.9)	39.9 (31.1-49.7)	-2.73 (-3.81 to -1.62)	-3.71 (-6.72 to -0.58)	-3.13 (-4.48 to -1.82)
Karauli	113.4 (104.3-123.1)	82.1 (74.7-89.5)	64.9 (52.3-79.0)	-3.18 (-3.96 to -2.40)	-3.33 (-6.12 to -0.63)	-3.25 (-4.41 to -2.08)
Kota	111.9 (102.4-122.1)	76.1 (68.9-84.0)	52.1 (41.5-64.5)	-3.75 (-4.60 to -2.82)	-5.24 (-8.05 to -2.49)	-4.37 (-5.55 to -3.16)
Nagaur	57.0 (50.7-63.3)	41.3 (36.5-46.3)	32.3 (25.5-41.0)	-3.16 (-4.26 to -2.05)	-3.52 (-6.49 to -0.54)	-3.31 (-4.65 to -2.04)
Pali	112.7 (101.9-123.7)	87.4 (78.7-96.1)	58.0 (47.3-71.6)	-2.52 (-3.42 to -1.60)	-5.73 (-8.47 to -2.88)	-3.86 (-5.07 to -2.69)
Pratapgarh	100.0 (89.9-111.5)	69.8 (62.2-78.4)	46.7 (36.7-59.0)	-3.55 (-4.52 to -2.52)	-5.57 (-8.49 to -2.41)	-4.39 (-5.67 to -3.03)
Rajsamand	126.6 (114.5-138.7)	95.6 (86.2-104.5)	60.8 (48.8-75.2)	-2.75 (-3.65 to -1.88)	-6.34 (-9.07 to -3.54)	-4.25 (-5.44 to -3.01)
Sawai Madhopur	128.3 (117.0-140.5)	93.7 (84.6-103.4)	71.0 (56.5-88.4)	-3.10 (-4.01 to -2.20)	-3.94 (-6.77 to -1.16)	-3.45 (-4.69 to -2.26)
Sikar	52.2 (46.6-58.2)	35.0 (31.0-39.3)	27.8 (22.1-34.9)	-3.93 (-5.00 to -2.80)	-3.27 (-6.31 to -0.23)	-3.66 (-4.98 to -2.32)
Sirohi	120.2 (108.7-132.4)	96.1 (86.2-106.4)	64.6 (51.4-80.8)	-2.21 (-3.21 to -1.20)	-5.59 (-8.52 to -2.74)	-3.62 (-4.89 to -2.31)
Sri Ganganagar	55.5 (49.5-62.0)	34.0 (30.0-38.3)	34.1 (26.7-43.7)	-4.80 (-5.95 to -3.68)	0.01 (-3.06 to 3.21)	-2.85 (-4.22 to -1.49)
Tonk	116.2 (105.5-126.7)	81.8 (74.2-90.5)	60.4 (47.7-75.3)	-3.45 (-4.29 to -2.59)	-4.28 (-7.05 to -1.57)	-3.80 (-4.97 to -2.61)
Udaipur	131.7 (119.2-145.3)	102.0 (92.0-112.7)	65.5 (52.4-82.1)	-2.52 (-3.55 to -1.56)	-6.20 (-9.06 to -3.21)	-4.05 (-5.32 to -2.72)
**Chhattisgarh**	**94.7 (85.4-104.3)**	**68.8 (60.8-77.8)**	**47.7 (41.3-55.7)**	**-3.15 (-3.93 to -2.43)**	**-5.13 (-7.84 to -2.42)**	**-3.97 (-5.09 to -2.81)**
Balod	90.2 (80.4-100.5)	64.4 (57.0-73.2)	45.7 (35.5-58.0)	-3.31 (-4.46 to -2.14)	-4.85 (-7.95 to -1.62)	-3.95 (-5.27 to -2.58)
Baloda Bazar	91.0 (82.7-99.5)	67.6 (60.9-75.2)	45.7 (36.2-56.8)	-2.93 (-3.89 to -2.02)	-5.51 (-8.47 to -2.65)	-4.00 (-5.32 to -2.75)
Balrampur	104.9 (95.4-115.9)	78.8 (70.8-88.0)	52.2 (42.0-64.8)	-2.82 (-3.76 to -1.92)	-5.80 (-8.55 to -3.00)	-4.06 (-5.21 to -2.87)
Bastar	90.0 (80.2-100.4)	68.3 (60.8-77.0)	51.2 (39.7-64.2)	-2.71 (-3.84 to -1.63)	-4.10 (-7.12 to -1.03)	-3.29 (-4.56 to -1.95)
Bemetara	96.5 (86.7-107.8)	67.7 (59.6-76.2)	49.4 (38.2-63.1)	-3.50 (-4.57 to -2.43)	-4.46 (-7.58 to -1.36)	-3.90 (-5.28 to -2.51)
Bijapur	77.8 (67.4-90.1)	57.3 (49.7-66.3)	46.8 (36.4-60.2)	-3.02 (-4.36 to -1.77)	-2.90 (-6.05 to 0.48)	-2.98 (-4.32 to -1.54)
Bilaspur	96.0 (87.1-105.6)	67.3 (60.5-74.8)	47.8 (37.6-59.1)	-3.49 (-4.52 to -2.53)	-4.82 (-7.87 to -1.83)	-4.04 (-5.34 to -2.78)
Dantewada	75.4 (65.6-86.0)	58.6 (51.2-67.5)	46.5 (35.1-59.3)	-2.50 (-3.78 to -1.19)	-3.32 (-6.59 to 0.02)	-2.84 (-4.21 to -1.40)
Dhamtari	87.4 (77.8-97.1)	63.8 (56.7-72.3)	42.9 (33.2-54.0)	-3.09 (-4.17 to -2.01)	-5.56 (-8.62 to -2.33)	-4.12 (-5.49 to -2.70)
Durg	85.6 (76.8-95.0)	60.3 (53.6-67.8)	41.2 (31.9-52.1)	-3.46 (-4.49 to -2.39)	-5.36 (-8.47 to -2.14)	-4.25 (-5.56 to -2.81)
Gariaband	98.5 (88.5-108.2)	74.2 (65.9-82.7)	50.6 (39.5-63.1)	-2.82 (-3.85 to -1.73)	-5.43 (-8.45 to -2.40)	-3.90 (-5.20 to -2.62)
Janjgir-Champa	96.7 (87.2-106.7)	71.7 (64.2-79.9)	45.9 (36.3-57.1)	-2.95 (-3.99 to -1.93)	-6.22 (-9.04 to -3.25)	-4.31 (-5.63 to -3.02)
Jashpur	98.4 (88.1-108.7)	72.4 (64.1-81.2)	45.8 (36.3-57.6)	-3.02 (-4.12 to -1.90)	-6.43 (-9.32 to -3.44)	-4.44 (-5.66 to -3.13)
Kabirdham	105.3 (93.7-118.8)	72.2 (62.9-82.2)	54.2 (41.3-70.0)	-3.70 (-4.79 to -2.56)	-4.08 (-7.35 to -0.78)	-3.86 (-5.27 to -2.49)
Kondagaon	109.8 (97.5-122.9)	84.0 (74.4-94.9)	62.8 (49.4-79.1)	-3.13 (-4.26 to -2.03)	-5.54 (-8.52 to -2.66)	-4.14 (-5.48 to -2.84)
Korba	94.1 (84.7-104.2)	68.5 (61.2-76.4)	46.1 (36.3-57.3)	-3.75 (-4.81 to -2.69)	-4.17 (-7.21 to -1.11)	-3.93 (-5.28 to -2.66)
Korea	101.5 (91.6-112.3)	69.2 (61.5-77.4)	51.5 (40.6-64.0)	-2.82 (-3.78 to -1.84)	-6.10 (-8.98 to -3.13)	-4.19 (-5.50 to -2.93)
Mahasamund	91.7 (83.4-100.8)	68.9 (62.0-76.6)	44.6 (35.3-56.3)	-3.51 (-4.57 to -2.44)	-4.42 (-7.54 to -1.33)	-3.89 (-5.29 to -2.58)
Mungeli	99.1 (89.0-111.0)	69.3 (61.0-78.3)	50.8 (39.2-64.3)	-2.34 (-3.72 to -1.08)	-3.47 (-6.58 to -0.12)	-2.81 (-4.16 to -1.35)
Narayanpur	105.5 (91.7-121.3)	83.3 (71.7-96.7)	65.3 (50.7-84.3)	-3.00 (-3.97 to -2.03)	-6.51 (-9.40 to -3.48)	-4.47 (-5.68 to -3.19)
North Bastar Kanker	98.0 (86.4-110.5)	73.6 (65.1-83.7)	55.7 (43.6-70.9)	-2.66 (-3.78 to -1.59)	-4.14 (-7.12 to -0.97)	-3.27 (-4.49 to -1.96)
Raigarh	95.9 (87.3-105.2)	70.9 (64.1-78.7)	44.5 (35.6-55.5)	-3.09 (-4.09 to -2.04)	-5.90 (-8.85 to -2.73)	-4.26 (-5.61 to -2.87)
Raipur	86.0 (76.9-95.3)	62.8 (55.9-70.7)	41.3 (32.3-52.3)	-3.33 (-4.49 to -2.21)	-4.33 (-7.56 to -1.15)	-3.75 (-5.06 to -2.37)
Rajnandgaon	90.6 (80.8-101.1)	64.6 (57.0-73.9)	47.6 (36.9-61.0)	-2.99 (-4.19 to -1.74)	-3.32 (-6.62 to 0.06)	-3.13 (-4.51 to -1.70)
Sukma	93.6 (81.7-108.2)	69.1 (59.9-80.5)	55.0 (41.6-70.4)	-3.47 (-4.39 to -2.53)	-5.01 (-7.95 to -2.19)	-4.11 (-5.32 to -2.86)
Surajpur	103.7 (93.8-114.3)	72.9 (65.4-81.2)	51.1 (40.9-64.0)	-3.23 (-4.20 to -2.24)	-5.79 (-8.69 to -2.77)	-4.30 (-5.53 to -3.07)
Surguja	97.8 (89.1-107.7)	70.4 (62.6-78.0)	46.5 (37.3-57.8)	-2.81 (-3.96 to -1.69)	-3.95 (-7.00 to -0.85)	-3.29 (-4.60 to -1.96)
**Odisha**	**99.1 (90.2-108.1)**	**68.3 (60.6-77.6)**	**49.1 (42.4-57.2)**	**-3.66 (-4.39 to -2.89)**	**-4.66 (-7.37 to -2.12)**	**-4.07 (-5.17 to -3.00)**
Angul	113.1 (102.6-125.2)	77.1 (68.9-86.1)	53.9 (42.0-67.8)	-3.76 (-4.82 to -2.71)	-5.05 (-8.22 to -2.09)	-4.30 (-5.65 to -2.97)

Balangir	103.1 (92.0-114.2)	78.2 (69.5-87.9)	57.1 (44.7-72.9)	-2.71 (-3.77 to -1.66)	-4.46 (-7.43 to -1.27)	-3.44 (-4.73 to -2.02)
Balasore	82.8 (73.0-92.5)	52.9 (46.3-60.5)	37.7 (28.8-47.6)	-4.41 (-5.56 to -3.24)	-4.80 (-7.98 to -1.46)	-4.58 (-5.97 to -3.21)
Bargarh	93.5 (83.9-103.1)	71.8 (64.3-80.1)	49.7 (39.0-63.0)	-2.59 (-3.64 to -1.55)	-5.14 (-8.11 to -2.02)	-3.66 (-4.94 to -2.29)
Bhadrak	93.4 (84.5-103.2)	55.7 (49.3-62.6)	40.4 (31.3-51.1)	-5.07 (-6.19 to -3.95)	-4.55 (-7.75 to -1.32)	-4.86 (-6.25 to -3.51)
Boudh	110.8 (99.2-123.3)	79.3 (70.1-89.4)	56.7 (44.2-71.5)	-3.29 (-4.36 to -2.15)	-4.76 (-7.94 to -1.65)	-3.90 (-5.17 to -2.50)
Cuttack	87.4 (78.5-96.6)	49.8 (44.1-55.4)	33.9 (26.3-42.9)	-5.45 (-6.52 to -4.33)	-5.47 (-8.67 to -2.31)	-5.46 (-6.79 to -4.16)
Deogarh	111.7 (101.3-123.8)	82.8 (74.4-91.8)	57.2 (44.9-71.4)	-2.97 (-3.99 to -1.97)	-5.20 (-8.24 to -2.18)	-3.90 (-5.29 to -2.65)
Dhenkanal	107.4 (96.8-118.6)	65.9 (58.7-73.3)	46.2 (35.6-58.2)	-4.79 (-5.78 to -3.69)	-5.00 (-8.04 to -1.88)	-4.88 (-6.20 to -3.60)
Gajapati	111.3 (98.3-124.1)	78.3 (68.7-89.2)	60.0 (46.2-76.6)	-3.47 (-4.71 to -2.10)	-3.78 (-7.31 to -0.37)	-3.61 (-5.08 to -2.16)
Ganjam	113.8 (99.5-128.5)	73.5 (63.9-84.7)	53.5 (40.7-68.2)	-4.27 (-5.56 to -2.92)	-4.50 (-7.89 to -1.26)	-4.37 (-5.74 to -3.01)
Jagatsinghapur	79.9 (71.5-89.1)	44.4 (38.8-50.3)	30.7 (23.3-39.2)	-5.71 (-6.89 to -4.48)	-5.23 (-8.49 to -2.04)	-5.52 (-6.94 to -4.13)
Jajapur	103.9 (93.7-114.9)	61.2 (54.3-68.6)	43.0 (33.6-53.7)	-5.17 (-6.19 to -4.06)	-4.97 (-8.03 to -1.87)	-5.09 (-6.47 to -3.82)
Jharsuguda	88.6 (79.1-99.4)	69.1 (61.5-77.5)	45.5 (35.5-57.2)	-2.45 (-3.53 to -1.41)	-5.84 (-8.86 to -2.73)	-3.86 (-5.17 to -2.52)
Kalahandi	118.3 (106.5-130.9)	92.1 (81.8-102.7)	70.8 (55.0-88.2)	-2.47 (-3.49 to -1.39)	-3.75 (-6.97 to -0.75)	-3.00 (-4.25 to -1.68)
Kandhamal	118.7 (106.4-132.0)	84.7 (74.7-95.4)	62.6 (49.0-78.4)	-3.32 (-4.39 to -2.30)	-4.29 (-7.45 to -1.16)	-3.73 (-5.01 to -2.37)
Kendrapara	89.7 (79.9-101.2)	51.5 (44.6-58.7)	36.5 (27.9-46.9)	-5.41 (-6.67 to -4.12)	-4.85 (-8.22 to -1.49)	-5.19 (-6.66 to -3.76)
Kendujhar	108.7 (98.3-119.8)	80.9 (72.7-90.1)	55.1 (43.5-68.5)	-2.92 (-3.92 to -1.90)	-5.40 (-8.38 to -2.47)	-3.95 (-5.26 to -2.75)
Khordha	79.0 (69.9-88.5)	45.5 (39.7-51.5)	30.2 (23.2-38.2)	-5.35 (-6.55 to -4.11)	-5.67 (-8.97 to -2.45)	-5.49 (-6.87 to -4.18)
Koraput	113.7 (102.0-127.1)	89.2 (79.1-100.1)	72.6 (56.6-91.9)	-2.40 (-3.59 to -1.30)	-2.93 (-6.14 to 0.14)	-2.63 (-3.88 to -1.32)
Malkangiri	102.2 (90.1-116.5)	80.3 (70.5-91.3)	70.0 (53.8-88.9)	-2.39 (-3.64 to -1.16)	-1.98 (-5.31 to 1.31)	-2.23 (-3.68 to -0.82)
Mayurbhanj	86.3 (77.4-95.5)	65.3 (58.4-72.7)	45.0 (35.2-56.9)	-2.78 (-3.84 to -1.69)	-5.27 (-8.26 to -2.26)	-3.81 (-5.11 to -2.55)
Nabarangpur	107.0 (96.7-117.8)	85.2 (75.9-95.0)	68.8 (54.2-86.5)	-2.24 (-3.26 to -1.20)	-3.05 (-6.02 to 0.00)	-2.58 (-3.83 to -1.34)
Nayagarh	96.3 (86.4-107.5)	58.9 (52.0-66.4)	42.7 (33.3-53.4)	-4.76 (-5.83 to -3.65)	-4.54 (-7.72 to -1.46)	-4.67 (-6.01 to -3.34)
Nuapada	97.9 (88.2-107.5)	78.3 (69.9-87.3)	58.5 (45.7-73.6)	-2.19 (-3.19 to -1.12)	-4.10 (-7.17 to -1.07)	-2.99 (-4.25 to -1.64)
Puri	73.4 (64.9-82.5)	42.8 (37.1-49.6)	29.1 (22.2-37.0)	-5.23 (-6.45 to -3.98)	-5.42 (-8.84 to -2.24)	-5.31 (-6.73 to -3.95)
Rayagada	128.6 (115.2-142.0)	98.6 (87.6-110.5)	77.4 (60.3-97.1)	-2.62 (-3.77 to -1.52)	-3.44 (-6.65 to -0.26)	-2.96 (-4.31 to -1.70)
Sambalpur	96.3 (86.9-106.7)	74.0 (66.5-82.2)	49.5 (38.8-62.2)	-2.59 (-3.55 to -1.64)	-5.65 (-8.61 to -2.54)	-3.87 (-5.16 to -2.55)
Sonepur	103.6 (93.2-115.0)	76.3 (68.6-85.5)	53.4 (41.5-68.2)	-3.02 (-4.08 to -1.91)	-5.03 (-8.11 to -1.93)	-3.85 (-5.12 to -2.41)
Sundargarh	104.0 (93.4-115.5)	81.4 (73.1-89.9)	53.3 (42.6-66.4)	-2.41 (-3.44 to -1.45)	-5.93 (-8.83 to -2.86)	-3.88 (-5.12 to -2.64)
**Assam**	**87.6 (79.6-96.1)**	**71.9 (63.7-81.4)**	**54.9 (47.5-64.0)**	**-1.95 (-2.60 to -1.31)**	**-3.83 (-6.53 to -1.11)**	**-2.73 (-3.81 to -1.66)**
Baksa	90.6 (81.9-99.5)	63.0 (56.6-70.6)	42.7 (33.9-52.8)	-3.57 (-4.43 to -2.63)	-5.50 (-8.48 to -2.78)	-4.37 (-5.54 to -3.19)
Barpeta	80.7 (71.8-89.5)	52.7 (46.6-59.5)	38.1 (29.8-47.7)	-4.19 (-5.24 to -3.05)	-4.57 (-7.70 to -1.44)	-4.35 (-5.61 to -2.99)
Biswanath	73.1 (64.6-81.6)	64.7 (57.4-72.6)	48.5 (38.4-59.9)	-1.21 (-2.26 to -0.22)	-4.10 (-6.97 to -1.14)	-2.41 (-3.71 to -1.15)
Bongaigaon	82.6 (74.3-90.9)	51.7 (46.2-57.5)	38.4 (30.6-48.1)	-4.57 (-5.55 to -3.52)	-4.23 (-7.26 to -1.24)	-4.43 (-5.66 to -3.12)
Cachar	101.4 (90.3-112.6)	100.1 (89.4-112.1)	81.2 (64.7-101.9)	-0.11 (-1.16 to 0.90)	-3.00 (-6.13 to 0.28)	-1.32 (-2.70 to 0.08)
Charaideo	80.1 (71.4-90.2)	77.0 (68.6-87.0)	64.6 (49.4-81.7)	-0.36 (-1.51 to 0.89)	-2.51 (-5.94 to 0.72)	-1.26 (-2.74 to 0.13)
Chirang	82.4 (73.4-90.7)	51.9 (46.3-58.3)	37.8 (30.2-47.4)	-4.51 (-5.43 to -3.56)	-4.47 (-7.48 to -1.49)	-4.50 (-5.69 to -3.27)
Darrang	98.9 (89.2-109.6)	79.9 (71.0-89.4)	56.5 (45.2-69.3)	-2.14 (-2.99 to -1.26)	-4.89 (-7.84 to -2.15)	-3.29 (-4.47 to -2.14)
Dhemaji	73.2 (65.0-82.3)	63.0 (56.2-71.0)	49.8 (38.7-62.2)	-1.49 (-2.64 to -0.21)	-3.36 (-6.90 to -0.09)	-2.27 (-3.66 to -0.84)
Dhubri	96.3 (86.5-106.9)	60.6 (54.5-67.9)	44.9 (35.9-57.0)	-4.51 (-5.55 to -3.50)	-4.29 (-7.31 to -1.09)	-4.42 (-5.65 to -3.08)
Dibrugarh	74.3 (66.0-83.2)	67.8 (60.4-76.6)	55.5 (43.0-69.8)	-0.89 (-2.03 to 0.44)	-2.87 (-6.33 to 0.50)	-1.72 (-3.16 to -0.33)
Dima Hasao	97.4 (87.3-108.3)	93.3 (83.3-103.6)	79.1 (63.4-97.7)	-0.43 (-1.41 to 0.50)	-2.38 (-5.34 to 0.60)	-1.25 (-2.55 to 0.08)
Goalpara	83.4 (75.5-91.2)	51.5 (46.2-57.2)	39.2 (31.6-49.2)	-4.72 (-5.63 to -3.74)	-3.89 (-6.89 to -0.95)	-4.38 (-5.60 to -3.10)
Golaghat	81.1 (72.5-91.0)	74.2 (66.1-83.4)	59.9 (47.6-74.5)	-0.88 (-1.94 to 0.22)	-3.08 (-6.10 to -0.12)	-1.79 (-3.07 to -0.51)
Hailakandi	104.5 (94.2-115.1)	101.9 (91.1-113.3)	83.5 (66.2-105.5)	-0.25 (-1.23 to 0.72)	-2.86 (-6.06 to 0.48)	-1.34 (-2.71 to 0.07)
Hojai	100.8 (89.4-112.2)	96.2 (84.8-108.0)	74.9 (59.0-94.2)	-0.47 (-1.62 to 0.62)	-3.63 (-6.66 to -0.60)	-1.79 (-3.10 to -0.46)
Jorhat	86.7 (77.3-97.4)	79.1 (69.6-89.5)	61.9 (47.4-78.0)	-0.92 (-2.06 to 0.26)	-3.52 (-6.70 to -0.40)	-2.00 (-3.39 to -0.61)
Kamrup	81.6 (73.5-89.4)	56.3 (50.2-62.9)	39.8 (31.7-49.0)	-3.65 (-4.53 to -2.68)	-4.85 (-7.95 to -1.96)	-4.15 (-5.41 to -2.89)
Kamrup Metropolitan	75.3 (67.6-83.4)	54.8 (48.9-61.2)	37.7 (29.6-46.5)	-3.24 (-4.14 to -2.33)	-5.30 (-8.34 to -2.47)	-4.10 (-5.36 to -2.90)
Karbi Anglong	87.2 (77.8-97.0)	81.6 (72.7-90.6)	65.3 (52.0-81.0)	-0.63 (-1.67 to 0.37)	-3.17 (-6.07 to -0.19)	-1.68 (-3.00 to -0.43)
Karimganj	109.2 (98.7-120.2)	100.2 (89.8-111.4)	84.3 (66.5-106.6)	-0.86 (-1.82 to 0.09)	-2.53 (-5.67 to 0.86)	-1.56 (-2.89 to -0.18)
Kokrajhar	91.8 (82.9-101.8)	57.2 (51.2-64.0)	41.8 (33.5-52.7)	-4.62 (-5.68 to -3.59)	-4.45 (-7.36 to -1.39)	-4.56 (-5.77 to -3.28)
Lakhimpur	78.0 (69.6-86.5)	68.0 (60.6-76.2)	51.7 (40.4-64.2)	-1.35 (-2.42 to -0.33)	-3.91 (-7.12 to -0.68)	-2.42 (-3.78 to -1.05)
Majuli	86.5 (77.2-96.3)	77.8 (68.8-87.6)	60.2 (46.6-74.8)	-1.06 (-2.17 to 0.02)	-3.65 (-6.93 to -0.47)	-2.14 (-3.50 to -0.77)
Morigaon	99.7 (89.7-109.7)	86.1 (77.2-95.7)	63.5 (50.9-78.2)	-1.46 (-2.37 to -0.55)	-4.32 (-7.27 to -1.47)	-2.65 (-3.90 to -1.50)
Nagaon	94.0 (84.0-103.4)	86.7 (77.7-96.3)	64.6 (51.8-79.9)	-0.80 (-1.76 to 0.19)	-4.15 (-7.07 to -1.22)	-2.19 (-3.42 to -0.95)
Nalbari	82.5 (74.0-90.7)	55.7 (49.5-62.6)	38.6 (30.6-47.9)	-3.84 (-4.78 to -2.78)	-5.18 (-8.25 to -2.32)	-4.40 (-5.66 to -3.09)
Sivasagar	83.3 (74.4-93.3)	76.8 (68.4-87.0)	62.1 (48.2-78.3)	-0.80 (-1.99 to 0.35)	-3.07 (-6.43 to 0.21)	-1.74 (-3.13 to -0.33)
Sonitpur	82.0 (73.2-90.9)	70.7 (63.4-78.3)	50.4 (40.6-61.7)	-1.47 (-2.44 to -0.47)	-4.77 (-7.62 to -1.82)	-2.85 (-4.10 to -1.62)
South Salmara Mancachar	94.1 (85.1-103.5)	57.7 (51.9-64.2)	42.7 (34.2-54.4)	-4.76 (-5.77 to -3.79)	-4.16 (-7.09 to -0.78)	-4.52 (-5.77 to -3.11)
Tinsukia	64.7 (57.1-73.3)	59.0 (51.7-68.0)	48.7 (37.3-62.1)	-0.89 (-2.18 to 0.67)	-2.77 (-6.30 to 0.66)	-1.67 (-3.30 to -0.12)
Udalguri	96.4 (86.1-108.0)	75.8 (66.9-85.4)	52.2 (41.8-64.7)	-2.32 (-3.30 to -1.34)	-5.21 (-8.18 to -2.50)	-3.52 (-4.72 to -2.32)
West Karbi Anglong	97.2 (86.5-108.7)	90.7 (80.2-101.5)	72.0 (56.8-90.1)	-0.70 (-1.80 to 0.32)	-3.28 (-6.36 to -0.19)	-1.78 (-3.07 to -0.46)
**Andhra Pradesh**	**81.8 (75.1-88.9)**	**55.3 (44.4-67.3)**	**36.1 (25.9-50.8)**	**-3.84 (-4.91 to -2.79)**	**-5.97 (-8.77 to -2.83)**	**-4.73 (-5.99 to -3.46)**
Anantapur	98.6 (85.3-113.0)	61.5 (52.7-71.8)	42.9 (31.9-56.6)	-4.62 (-6.01 to -3.21)	-5.11 (-8.75 to -1.13)	-4.83 (-6.49 to -3.25)
Chittoor	73.6 (63.7-84.1)	43.0 (36.8-49.9)	31.6 (23.3-42.2)	-5.25 (-6.74 to -3.76)	-4.36 (-8.05 to -0.40)	-4.89 (-6.56 to -3.21)
East Godavari	68.1 (58.3-79.9)	46.2 (39.4-55.1)	31.0 (23.1-41.1)	-3.79 (-5.41 to -2.18)	-5.65 (-9.05 to -1.98)	-4.57 (-6.21 to -2.94)
Guntur	68.0 (58.1-80.4)	49.1 (40.9-58.5)	30.4 (22.0-40.6)	-3.21 (-4.89 to -1.49)	-6.71 (-10.18 to -3.06)	-4.67 (-6.28 to -2.96)
Krishna	70.0 (60.2-81.4)	48.1 (40.4-56.9)	29.4 (21.3-39.4)	-3.68 (-5.31 to -2.02)	-6.89 (-10.45 to -3.20)	-5.02 (-6.60 to -3.36)
Kurnool	103.5 (90.5-118.0)	73.6 (63.2-85.3)	47.6 (35.1-63.8)	-3.35 (-4.71 to -1.90)	-6.13 (-9.66 to -2.39)	-4.51 (-6.13 to -2.91)
Prakasam	70.6 (59.6-82.8)	53.5 (44.9-63.9)	35.0 (25.8-47.1)	-2.74 (-4.35 to -0.96)	-5.97 (-9.64 to -2.19)	-4.09 (-5.78 to -2.38)
Sri Potti Sriramulu Nellore	70.4 (58.7-83.5)	47.9 (39.7-57.8)	33.5 (24.6-45.1)	-3.77 (-5.42 to -1.92)	-5.06 (-8.64 to -0.96)	-4.31 (-6.01 to -2.60)
Srikakulam	106.2 (92.9-121.1)	66.6 (57.5-77.3)	40.1 (30.1-51.7)	-4.58 (-5.96 to -3.18)	-7.22 (-10.67 to -3.91)	-5.68 (-7.17 to -4.17)
Visakhapatnam	85.2 (73.8-98.2)	58.1 (49.5-68.1)	37.3 (27.9-48.4)	-3.76 (-5.26 to -2.30)	-6.23 (-9.64 to -2.77)	-4.79 (-6.36 to -3.31)
Vizianagaram	116.6 (102.2-133.2)	79.1 (68.0-91.7)	48.6 (36.0-63.7)	-3.82 (-5.28 to -2.40)	-6.80 (-10.24 to -3.37)	-5.06 (-6.60 to -3.57)
West Godavari	73.0 (62.4-85.0)	49.5 (41.5-60.0)	32.2 (23.4-43.3)	-3.80 (-5.40 to -2.20)	-6.09 (-9.47 to -2.39)	-4.76 (-6.32 to -3.03)
YSR	86.7 (74.5-99.7)	60.0 (51.1-70.4)	40.2 (29.8-53.1)	-3.63 (-5.04 to -2.01)	-5.66 (-9.44 to -2.00)	-4.48 (-6.14 to -2.91)
**West Bengal**	**65.2 (59.3-71.5)**	**42.7 (35.1-51.0)**	**29.2 (25.2-34.2)**	**-4.15 (-4.93 to -3.40)**	**-5.35 (-8.03 to -2.59)**	**-4.65 (-5.80 to -3.52)**
Alipurduar	71.0 (63.5-79.5)	45.5 (40.5-51.2)	29.6 (23.8-37.5)	-4.36 (-5.42 to -3.34)	-6.03 (-8.97 to -2.97)	-5.05 (-6.23 to -3.72)
Bankura	64.5 (57.1-73.3)	47.5 (41.7-54.0)	32.0 (24.9-40.4)	-2.99 (-4.17 to -1.97)	-5.57 (-8.50 to -2.69)	-4.06 (-5.36 to -2.74)
Birbhum	78.7 (71.1-86.8)	55.4 (49.4-61.6)	37.9 (29.9-47.0)	-3.45 (-4.42 to -2.54)	-5.34 (-8.19 to -2.45)	-4.24 (-5.53 to -2.99)
Cooch Behar	78.7 (70.8-87.4)	50.8 (45.4-56.6)	34.0 (27.4-43.3)	-4.27 (-5.32 to -3.27)	-5.53 (-8.42 to -2.42)	-4.79 (-5.97 to -3.46)
Dakshin Dinajpur	73.7 (65.6-82.2)	48.8 (43.2-55.1)	33.8 (26.5-43.1)	-4.06 (-5.02 to -3.00)	-5.12 (-8.00 to -1.91)	-4.50 (-5.75 to -3.17)
Darjeeling	71.7 (63.5-80.4)	49.1 (43.4-55.5)	29.6 (23.4-37.4)	-3.72 (-4.84 to -2.58)	-6.88 (-9.67 to -3.71)	-5.04 (-6.30 to -3.68)
Hooghly	56.2 (50.5-63.4)	33.7 (29.5-38.4)	24.1 (18.5-31.0)	-5.00 (-6.12 to -3.81)	-4.74 (-8.05 to -1.56)	-4.90 (-6.33 to -3.50)
Howrah	50.1 (44.0-57.6)	29.9 (25.8-34.3)	20.7 (15.5-27.1)	-5.03 (-6.24 to -3.75)	-5.23 (-8.69 to -1.80)	-5.12 (-6.67 to -3.66)
Jalpaiguri	70.7 (63.8-78.0)	49.4 (44.0-55.0)	30.8 (24.8-38.7)	-3.52 (-4.54 to -2.53)	-6.67 (-9.33 to -3.56)	-4.83 (-6.02 to -3.54)
Jhargram	66.1 (58.8-74.5)	45.8 (40.3-52.0)	31.3 (24.4-39.7)	-3.58 (-4.82 to -2.44)	-5.37 (-8.50 to -2.25)	-4.33 (-5.71 to -3.02)

Kalimpong	58.0 (51.9-64.8)	40.4 (35.4-45.5)	23.3 (18.7-29.1)	-3.58 (-4.64 to -2.44)	-7.61 (-10.38 to -4.45)	-5.27 (-6.50 to -3.95)
Kolkata	46.8 (41.0-53.6)	28.2 (24.3-32.5)	19.3 (14.4-25.2)	-4.97 (-6.21 to -3.68)	-5.35 (-8.89 to -1.91)	-5.13 (-6.69 to -3.67)
Maldah	86.9 (78.8-95.8)	61.1 (54.7-68.3)	41.1 (32.7-52.1)	-3.44 (-4.32 to -2.47)	-5.54 (-8.33 to -2.57)	-4.31 (-5.57 to -3.05)
Murshidabad	78.2 (70.3-86.8)	51.5 (45.5-57.7)	35.2 (27.6-44.5)	-4.09 (-5.16 to -2.97)	-5.33 (-8.41 to -2.21)	-4.60 (-5.89 to -3.29)
Nadia	66.1 (58.1-74.8)	40.3 (35.2-46.3)	28.4 (21.7-36.3)	-4.82 (-5.87 to -3.76)	-4.97 (-8.19 to -1.83)	-4.88 (-6.27 to -3.46)
North 24 Parganas	55.0 (49.2-61.5)	32.5 (28.3-36.8)	23.0 (17.3-29.8)	-5.16 (-6.30 to -4.02)	-4.76 (-8.18 to -1.39)	-5.00 (-6.52 to -3.56)
Paschim Burdwan	68.1 (60.1-76.9)	51.5 (45.7-57.7)	32.8 (25.8-41.4)	-2.75 (-3.90 to -1.63)	-6.27 (-9.20 to -3.42)	-4.22 (-5.50 to -2.86)
Paschim Medinipur	62.7 (55.7-70.5)	40.0 (35.0-45.6)	28.3 (21.8-36.3)	-4.39 (-5.54 to -3.26)	-4.92 (-8.00 to -1.79)	-4.61 (-5.99 to -3.31)
Purba Burdwan	63.0 (55.3-72.1)	41.1 (35.7-47.6)	29.0 (22.2-37.2)	-4.17 (-5.30 to -3.12)	-4.93 (-8.08 to -1.90)	-4.49 (-5.84 to -3.13)
Purba Medinipur	60.1 (52.6-68.2)	35.6 (30.6-40.8)	25.3 (19.1-32.4)	-5.09 (-6.31 to -3.87)	-4.88 (-8.34 to -1.27)	-5.01 (-6.47 to -3.64)
Puruliya	78.3 (70.5-86.6)	62.2 (55.8-68.7)	39.1 (31.2-48.6)	-2.26 (-3.18 to -1.40)	-6.51 (-9.31 to -3.69)	-4.04 (-5.22 to -2.76)
South 24 Parganas	58.5 (51.3-66.7)	35.6 (30.3-41.3)	24.9 (18.7-32.3)	-4.78 (-6.04 to -3.53)	-5.19 (-8.67 to -1.72)	-4.95 (-6.50 to -3.50)
Uttar Dinajpur	81.7 (73.6-89.8)	59.0 (53.0-65.8)	40.0 (31.6-50.4)	-3.18 (-4.09 to -2.23)	-5.43 (-8.24 to -2.28)	-4.12 (-5.35 to -2.83)
**Tripura**	**61.2 (55.5-67.4)**	**44.9 (36.8-54.8)**	**34.0 (29.3-39.8)**	**-3.05 (-4.01 to -2.00)**	**-3.97 (-7.29 to -0.47)**	**-3.43 (-4.89 to -2.09)**
Dhalai	66.9 (59.6-74.9)	52.3 (46.2-59.1)	42.3 (32.5-54.3)	-2.44 (-3.58 to -1.24)	-3.03 (-6.52 to 0.48)	-2.69 (-4.17 to -1.21)
Gomati	56.8 (50.8-63.4)	42.5 (37.6-48.4)	31.8 (24.7-40.5)	-2.84 (-3.87 to -1.72)	-4.09 (-7.52 to -0.52)	-3.36 (-4.81 to -1.97)
Khowai	67.1 (60.3-74.5)	46.7 (41.6-52.3)	35.2 (27.3-45.0)	-3.56 (-4.56 to -2.51)	-4.02 (-7.38 to -0.55)	-3.75 (-5.20 to -2.35)
North Tripura	74.2 (66.7-82.5)	61.0 (54.4-68.5)	49.7 (38.5-63.4)	-1.99 (-3.05 to -0.96)	-2.99 (-6.28 to 0.48)	-2.40 (-3.84 to -0.95)
Sepahijala	54.2 (48.7-60.1)	37.8 (33.5-42.8)	27.8 (21.5-35.4)	-3.54 (-4.52 to -2.45)	-4.43 (-7.77 to -0.86)	-3.91 (-5.35 to -2.56)
South Tripura	51.8 (45.5-58.8)	38.6 (33.1-44.3)	29.3 (22.5-37.8)	-2.87 (-4.02 to -1.65)	-3.91 (-7.47 to -0.42)	-3.31 (-4.75 to -1.85)
Unakoti	69.8 (62.6-77.8)	53.9 (47.9-60.8)	43.3 (33.4-55.6)	-2.56 (-3.61 to -1.51)	-3.15 (-6.49 to 0.32)	-2.81 (-4.23 to -1.35)
West Tripura	58.9 (53.0-65.1)	39.9 (35.6-45.2)	27.7 (21.4-35.5)	-3.81 (-4.77 to -2.75)	-5.12 (-8.56 to -1.63)	-4.36 (-5.77 to -3.02)
**Arunachal Pradesh**	**73.0 (65.9-80.7)**	**40.1 (34.8-46.1)**	**27.3 (23.6-32.0)**	**-5.82 (-7.02 to -4.46)**	**-5.39 (-8.74 to -2.17)**	**-5.64 (-7.15 to -4.22)**
Anjaw	62.3 (49.2-76.9)	34.9 (27.6-42.8)	25.4 (17.5-35.4)	-5.65 (-7.72 to -3.41)	-4.47 (-8.65 to -0.45)	-5.17 (-7.09 to -3.25)
Changlang	75.8 (64.8-88.1)	44.3 (38.2-51.3)	31.1 (23.2-40.2)	-5.36 (-6.78 to -3.74)	-5.05 (-8.60 to -1.64)	-5.24 (-6.93 to -3.67)
Dibang Valley	50.4 (39.3-64.0)	24.5 (19.1-31.6)	16.7 (11.6-23.4)	-6.95 (-8.90 to -5.00)	-5.40 (-9.37 to -1.65)	-6.32 (-8.09 to -4.54)
East Kameng	80.5 (67.8-94.8)	37.2 (30.9-44.2)	22.7 (17.5-29.2)	-7.42 (-8.63 to -6.16)	-6.91 (-9.96 to -4.00)	-7.21 (-8.51 to -5.83)
East Siang	64.3 (55.7-73.6)	33.1 (28.8-38.6)	22.7 (17.1-29.5)	-6.72 (-8.06 to -5.16)	-5.55 (-9.16 to -1.98)	-6.24 (-7.81 to -4.68)
Kamle	73.8 (64.5-83.8)	35.7 (31.4-40.9)	22.3 (17.2-28.2)	-7.02 (-8.14 to -5.79)	-6.52 (-9.80 to -3.31)	-6.82 (-8.12 to -5.41)
Kra Daddi	69.9 (58.9-81.6)	32.8 (27.6-38.9)	20.4 (15.3-26.3)	-7.29 (-8.74 to -5.84)	-6.62 (-10.00 to -3.26)	-7.02 (-8.42 to -5.47)
Kurung Kumey	84.5 (70.8-99.2)	36.3 (30.2-43.4)	23.3 (17.7-30.3)	-8.09 (-9.46 to -6.73)	-6.28 (-9.56 to -3.26)	-7.36 (-8.71 to -5.97)
Lohit	64.1 (55.0-74.1)	35.1 (30.3-40.5)	24.0 (17.8-31.3)	-5.85 (-7.39 to -4.14)	-5.35 (-9.10 to -1.63)	-5.65 (-7.37 to -4.03)
Longding	84.7 (74.3-97.0)	52.1 (45.0-59.4)	37.0 (28.3-47.3)	-4.76 (-5.99 to -3.35)	-4.89 (-8.29 to -1.58)	-4.82 (-6.30 to -3.48)
Lower Dibang Valley	59.2 (50.4-69.3)	30.7 (25.8-36.4)	20.7 (15.2-27.3)	-6.36 (-7.85 to -4.66)	-5.55 (-9.22 to -1.90)	-6.04 (-7.76 to -4.37)
Lower Siang	76.5 (67.0-87.2)	42.2 (37.0-48.1)	28.1 (21.7-35.9)	-6.66 (-7.88 to -5.32)	-6.32 (-9.87 to -2.91)	-6.53 (-7.90 to -5.06)
Lower Subansiri	69.9 (60.8-79.0)	34.1 (29.9-39.1)	20.6 (15.9-26.1)	-6.92 (-8.04 to -5.74)	-7.00 (-10.12 to -3.85)	-6.96 (-8.34 to -5.57)
Namsai	70.1 (61.2-80.2)	38.9 (34.0-44.7)	26.4 (19.9-33.9)	-5.73 (-7.13 to -4.15)	-5.49 (-9.05 to -1.97)	-5.64 (-7.29 to -4.11)
Pakke Kessang	73.4 (63.6-83.4)	37.7 (33.2-42.7)	21.7 (17.1-26.9)	-6.42 (-7.50 to -5.33)	-7.67 (-10.48 to -4.66)	-6.94 (-8.23 to -5.64)
Papum Pare	77.5 (68.7-86.4)	40.9 (36.5-45.7)	25.1 (19.7-31.0)	-6.68 (-7.67 to -5.71)	-7.17 (-10.10 to -4.23)	-6.89 (-8.17 to -5.60)
Shi Yomi	65.2 (57.1-74.3)	31.2 (27.0-35.9)	19.4 (14.8-25.0)	-7.12 (-8.35 to -5.75)	-6.63 (-10.09 to -3.33)	-6.93 (-8.38 to -5.46)
Siang	50.9 (42.4-60.2)	23.0 (19.1-27.9)	15.0 (10.9-20.2)	-7.65 (-9.28 to -6.05)	-6.00 (-9.59 to -2.40)	-6.98 (-8.63 to -5.37)
Tawang	80.1 (69.5-90.8)	36.8 (31.7-42.2)	21.1 (16.7-26.1)	-7.51 (-8.65 to -6.37)	-7.76 (-10.54 to -4.96)	-7.62 (-8.83 to -6.40)
Tirap	87.8 (75.5-101.9)	52.7 (45.4-61.1)	35.7 (26.6-46.6)	-4.99 (-6.29 to -3.49)	-5.49 (-9.13 to -1.98)	-5.20 (-6.81 to -3.74)
Upper Siang	50.0 (39.9-60.9)	22.3 (17.8-28.1)	14.9 (10.5-20.6)	-7.78 (-9.59 to -5.98)	-5.67 (-9.44 to -1.99)	-6.92 (-8.61 to -5.17)
Upper Subansiri	65.5 (55.5-76.8)	30.0 (25.0-35.6)	19.2 (14.3-25.3)	-7.55 (-8.99 to -6.05)	-6.23 (-9.68 to -2.89)	-7.01 (-8.41 to -5.48)
West Kameng	78.2 (69.5-87.0)	36.6 (32.1-41.0)	21.0 (17.0-25.6)	-7.25 (-8.26 to -6.28)	-7.70 (-10.42 to -5.00)	-7.44 (-8.63 to -6.23)
West Siang	55.0 (43.9-67.1)	24.8 (19.5-30.5)	16.3 (11.6-22.2)	-7.64 (-9.17 to -5.96)	-5.91 (-9.55 to -2.42)	-6.94 (-8.53 to -5.36)
**Meghalaya**	**66.0 (60.0-72.6)**	**49.2 (42.8-56.3)**	**39.1 (33.7-45.6)**	**-2.90 (-3.67 to -2.16)**	**-3.29 (-6.20 to -0.29)**	**-3.06 (-4.27 to -1.84)**
East Garo Hills	58.9 (52.9-64.6)	39.2 (35.0-44.0)	29.4 (23.5-37.1)	-3.92 (-4.84 to -3.00)	-3.97 (-6.99 to -0.91)	-3.94 (-5.13 to -2.58)
East Jantia Hills	85.7 (76.6-94.6)	82.6 (74.2-91.5)	73.4 (57.9-91.3)	-0.36 (-1.34 to 0.54)	-1.73 (-4.81 to 1.47)	-0.93 (-2.30 to 0.40)
East Khasi Hills	63.4 (57.3-70.1)	47.8 (42.8-53.0)	37.7 (29.9-46.4)	-2.81 (-3.72 to -1.92)	-3.37 (-6.46 to -0.34)	-3.04 (-4.30 to -1.78)
North Garo Hills	60.1 (54.1-65.8)	40.5 (36.0-45.2)	31.4 (25.1-39.4)	-3.96 (-4.87 to -3.02)	-3.53 (-6.51 to -0.45)	-3.79 (-5.02 to -2.44)
Ri Bhoi	67.1 (60.7-73.4)	56.2 (50.3-62.4)	44.5 (35.5-54.8)	-1.76 (-2.63 to -0.87)	-3.73 (-6.80 to -0.80)	-2.58 (-3.80 to -1.38)
South Garo Hills	62.7 (56.4-69.4)	39.6 (35.0-44.5)	28.4 (22.5-36.5)	-4.53 (-5.47 to -3.55)	-4.74 (-7.67 to -1.44)	-4.62 (-5.86 to -3.20)
South West Garo Hills	72.4 (64.6-80.4)	45.3 (39.9-51.1)	33.7 (26.5-43.5)	-4.51 (-5.56 to -3.38)	-3.96 (-6.93 to -0.44)	-4.29 (-5.63 to -2.77)
South West Khasi Hills	62.4 (56.0-69.1)	43.4 (38.6-48.6)	35.5 (27.8-44.3)	-3.57 (-4.51 to -2.59)	-3.10 (-6.29 to 0.08)	-3.38 (-4.69 to -2.05)
West Garo Hills	66.8 (60.2-73.5)	43.5 (38.8-48.6)	32.8 (26.1-41.8)	-4.27 (-5.25 to -3.27)	-4.06 (-6.99 to -0.80)	-4.18 (-5.42 to -2.72)
East Jaintia Hills	72.6 (65.4-80.2)	64.5 (57.9-71.4)	55.6 (44.3-68.8)	-1.14 (-2.11 to -0.27)	-2.14 (-5.15 to 0.94)	-1.56 (-2.87 to -0.29)
West Khasi Hills	60.4 (54.6-66.0)	43.0 (38.5-47.7)	34.6 (27.5-43.1)	-3.33 (-4.22 to -2.47)	-3.12 (-6.26 to 0.04)	-3.24 (-4.47 to -2.00)
**Karnataka**	**64.9 (59.2-70.9)**	**44.5 (39.1-50.4)**	**31.7 (27.4-37.1)**	**-3.69 (-4.72 to -2.67)**	**-4.81 (-8.18 to -1.29)**	**-4.16 (-5.56 to -2.87)**
Bagalkot	80.9 (71.4-91.6)	54.2 (46.8-61.5)	37.2 (27.7-48.8)	-3.93 (-5.30 to -2.55)	-5.33 (-9.17 to -1.23)	-4.51 (-6.04 to -2.81)
Ballari	89.2 (77.5-101.8)	64.9 (55.8-74.6)	45.9 (34.2-61.1)	-3.13 (-4.49 to -1.70)	-4.92 (-8.54 to -0.84)	-3.88 (-5.50 to -2.22)
Belagavi	58.6 (50.9-66.7)	39.9 (34.1-46.0)	27.9 (20.7-36.9)	-3.76 (-5.16 to -2.35)	-5.07 (-9.05 to -1.07)	-4.31 (-5.91 to -2.66)
Bengaluru Rural	65.9 (57.5-74.8)	44.7 (38.8-51.8)	33.2 (24.2-43.7)	-3.86 (-5.24 to -2.33)	-4.27 (-8.16 to -0.33)	-4.04 (-5.71 to -2.43)
Bengaluru Urban	54.7 (47.0-62.8)	38.3 (32.8-44.8)	27.0 (19.5-36.0)	-3.48 (-4.88 to -1.92)	-4.99 (-8.80 to -0.90)	-4.11 (-5.83 to -2.42)
Bidar	51.7 (45.1-58.8)	42.6 (36.6-48.8)	28.0 (21.2-35.7)	-1.91 (-3.38 to -0.47)	-5.90 (-9.42 to -2.49)	-3.58 (-5.10 to -2.10)
Chamarajanagar	64.7 (55.2-75.7)	44.8 (37.8-53.2)	31.9 (22.7-42.8)	-3.59 (-5.04 to -2.03)	-4.84 (-8.81 to -0.73)	-4.11 (-5.84 to -2.43)
Chikballapur	66.2 (57.4-75.7)	46.5 (40.2-54.3)	35.8 (26.1-46.9)	-3.44 (-4.88 to -1.96)	-3.78 (-7.55 to 0.14)	-3.58 (-5.28 to -1.96)
Chikkamagaluru	56.5 (48.2-65.3)	34.8 (29.6-41.3)	25.1 (18.1-33.5)	-4.73 (-6.24 to -3.10)	-4.65 (-8.59 to -0.40)	-4.70 (-6.42 to -3.07)
Chitradurga	75.4 (64.9-86.0)	50.0 (42.8-58.5)	37.0 (27.0-49.5)	-4.02 (-5.43 to -2.55)	-4.28 (-8.17 to -0.12)	-4.13 (-5.83 to -2.49)
Dakshina Kannada	30.1 (25.1-35.4)	19.2 (15.7-23.5)	13.2 (9.2-18.2)	-4.41 (-6.22 to -2.46)	-5.31 (-9.41 to -0.95)	-4.79 (-6.72 to -3.01)
Davanagere	79.0 (69.2-89.3)	52.6 (45.1-61.3)	36.3 (26.4-47.9)	-4.00 (-5.39 to -2.60)	-5.25 (-9.07 to -1.07)	-4.52 (-6.19 to -2.92)
Dharwad	77.1 (67.2-88.5)	49.8 (42.6-57.9)	35.3 (26.0-47.6)	-4.28 (-5.65 to -2.85)	-4.88 (-8.96 to -0.60)	-4.53 (-6.12 to -2.74)
Gadag	96.4 (84.7-108.9)	64.7 (55.9-74.4)	45.3 (33.3-60.4)	-3.91 (-5.24 to -2.49)	-5.05 (-9.09 to -0.94)	-4.38 (-6.03 to -2.56)
Hassan	61.3 (52.4-71.3)	37.5 (31.9-44.1)	27.0 (19.3-36.2)	-4.80 (-6.31 to -3.28)	-4.69 (-8.61 to -0.63)	-4.76 (-6.55 to -3.10)
Haveri	80.2 (69.9-92.2)	53.4 (45.6-62.1)	37.7 (27.3-50.4)	-4.00 (-5.44 to -2.44)	-4.96 (-8.97 to -0.78)	-4.40 (-6.02 to -2.60)
Kalaburgi	60.3 (51.7-69.4)	46.7 (39.6-54.0)	33.0 (25.0-43.1)	-2.52 (-4.01 to -1.02)	-4.90 (-8.44 to -1.20)	-3.51 (-5.22 to -1.99)
Kodagu	40.7 (34.8-47.2)	25.6 (21.6-30.2)	18.6 (13.1-25.1)	-4.55 (-6.19 to -2.85)	-4.51 (-8.52 to -0.30)	-4.54 (-6.45 to -2.75)
Kolar	60.7 (52.0-70.1)	41.6 (35.6-48.8)	31.2 (22.7-41.6)	-3.65 (-5.19 to -2.09)	-4.10 (-7.88 to -0.20)	-3.85 (-5.62 to -2.13)
Koppal	98.4 (87.6-110.4)	69.4 (60.4-78.7)	47.9 (35.7-62.7)	-3.44 (-4.73 to -2.09)	-5.24 (-9.21 to -1.32)	-4.19 (-5.77 to -2.51)
Mandya	68.7 (60.0-79.0)	44.4 (37.8-51.7)	31.8 (22.9-41.8)	-4.26 (-5.77 to -2.72)	-4.75 (-8.73 to -0.79)	-4.47 (-6.21 to -2.83)
Mysuru	61.1 (52.8-70.6)	40.4 (34.3-47.7)	27.9 (20.0-37.3)	-4.05 (-5.60 to -2.44)	-5.25 (-9.32 to -1.30)	-4.56 (-6.34 to -2.89)
Raichur	84.8 (73.9-96.5)	62.8 (54.0-71.6)	45.2 (33.9-58.7)	-2.96 (-4.31 to -1.55)	-4.68 (-8.23 to -0.80)	-3.67 (-5.19 to -2.02)
Ramanagara	67.9 (58.9-77.9)	45.1 (38.5-52.8)	33.0 (23.7-43.9)	-4.01 (-5.37 to -2.52)	-4.47 (-8.26 to -0.34)	-4.21 (-5.91 to -2.54)
Shivamogga	56.1 (48.6-64.4)	36.4 (30.9-42.8)	25.6 (18.4-34.0)	-4.23 (-5.78 to -2.60)	-4.98 (-9.06 to -0.87)	-4.55 (-6.28 to -2.86)
Tumakuru	71.0 (62.2-81.1)	46.5 (40.3-54.3)	35.2 (26.0-46.5)	-4.12 (-5.48 to -2.63)	-3.98 (-7.93 to 0.07)	-4.07 (-5.80 to -2.46)
Udupi	35.1 (28.9-42.0)	22.4 (18.0-27.8)	16.0 (11.1-22.1)	-4.41 (-6.12 to -2.59)	-4.76 (-8.95 to -0.68)	-4.56 (-6.53 to -2.76)
Uttara Kannada	52.9 (44.5-62.6)	34.5 (28.8-41.1)	26.0 (18.5-35.7)	-4.19 (-5.78 to -2.50)	-4.06 (-8.25 to 0.17)	-4.14 (-5.86 to -2.36)

Vijaypura	66.1 (57.5-75.1)	46.7 (40.1-53.4)	32.9 (24.6-42.8)	-3.42 (-4.87 to -2.09)	-4.95 (-8.52 to -1.08)	-4.06 (-5.63 to -2.44)
Yadgir	74.5 (64.5-85.3)	54.8 (47.0-62.9)	40.7 (30.5-52.8)	-3.02 (-4.41 to -1.59)	-4.25 (-7.72 to -0.54)	-3.53 (-5.16 to -1.90)
**Telangana**	**73.0 (65.2-80.6)**	**45.5 (36.3-56.2)**	**28.6 (20.6-40.3)**	**-4.62 (-5.89 to -3.41)**	**-6.47 (-9.71 to -3.16)**	**-5.39 (-6.72 to -3.99)**
Adilabad	64.3 (53.0-75.5)	48.5 (40.3-57.5)	31.4 (23.2-41.5)	-2.77 (-4.44 to -1.14)	-6.13 (-9.42 to -2.69)	-4.17 (-5.64 to -2.68)
Bhadradri Kothagudem	84.3 (70.2-100.8)	54.2 (44.0-65.2)	36.7 (26.8-48.4)	-4.29 (-5.68 to -2.80)	-5.44 (-8.71 to -1.86)	-4.77 (-6.26 to -3.22)
Hyderabad	61.9 (53.0-71.7)	37.2 (31.3-44.1)	22.4 (16.7-29.5)	-4.95 (-6.44 to -3.46)	-7.08 (-10.82 to -3.58)	-5.84 (-7.39 to -4.24)
Jagitial	66.9 (57.3-78.7)	45.6 (38.7-54.2)	28.1 (21.0-37.0)	-3.76 (-5.32 to -2.23)	-6.77 (-10.00 to -3.29)	-5.02 (-6.52 to -3.53)
Jangoan	70.9 (60.4-82.0)	42.0 (35.4-49.4)	26.4 (19.5-34.9)	-5.12 (-6.60 to -3.71)	-6.47 (-9.91 to -3.03)	-5.68 (-7.13 to -4.18)
Jayashankar Bhupalpally	70.7 (57.5-86.6)	46.2 (36.8-56.8)	31.4 (23.3-42.2)	-4.17 (-5.76 to -2.66)	-5.46 (-8.76 to -1.84)	-4.71 (-6.27 to -3.10)
Jogulamba Gadwal	103.3 (87.9-120.1)	62.2 (52.3-72.7)	40.8 (30.4-54.1)	-4.95 (-6.42 to -3.49)	-5.96 (-9.58 to -2.25)	-5.37 (-6.89 to -3.78)
Kamareddy	72.1 (60.5-85.6)	46.8 (38.6-56.1)	28.6 (20.9-37.6)	-4.23 (-5.91 to -2.63)	-6.89 (-10.44 to -3.45)	-5.34 (-6.84 to -3.81)
Karimnagar	66.1 (55.9-78.2)	41.3 (34.7-49.1)	25.7 (19.3-33.8)	-4.58 (-6.14 to -3.16)	-6.63 (-9.84 to -3.16)	-5.44 (-6.88 to -3.93)
Khammam	75.3 (61.4-90.8)	48.4 (39.4-59.2)	31.1 (22.3-42.0)	-4.33 (-5.84 to -2.71)	-6.19 (-9.61 to -2.57)	-5.11 (-6.68 to -3.48)
Kumuram Bheem Asifabad	66.3 (54.5-81.2)	47.5 (38.5-58.8)	31.1 (22.9-42.1)	-3.28 (-4.92 to -1.66)	-5.96 (-9.13 to -2.47)	-4.40 (-5.86 to -2.77)
Mahabubnagar	92.6 (79.5-107.2)	52.8 (44.8-62.5)	35.2 (26.2-45.7)	-5.47 (-6.94 to -4.05)	-5.71 (-9.30 to -2.11)	-5.58 (-7.15 to -4.01)
Mahuababad	70.4 (59.6-82.6)	44.6 (37.1-53.3)	28.9 (21.2-38.5)	-4.47 (-6.00 to -2.94)	-6.08 (-9.49 to -2.58)	-5.14 (-6.65 to -3.59)
Mancherial	66.8 (56.1-80.8)	46.1 (38.1-55.9)	29.2 (21.8-38.8)	-3.65 (-5.26 to -2.07)	-6.39 (-9.55 to -2.97)	-4.79 (-6.25 to -3.19)
Medak	77.7 (65.2-92.0)	47.5 (39.1-57.2)	28.8 (21.1-38.2)	-4.80 (-6.42 to -3.13)	-6.96 (-10.54 to -3.38)	-5.70 (-7.27 to -4.11)
Medchal Malkajgiri	65.4 (56.4-75.3)	38.3 (32.2-45.1)	22.8 (17.0-30.1)	-5.26 (-6.76 to -3.77)	-7.20 (-10.87 to -3.70)	-6.07 (-7.56 to -4.52)
Nagarkurnool	91.6 (74.9-110.5)	58.0 (46.4-71.7)	37.2 (26.6-51.0)	-4.48 (-6.03 to -2.86)	-6.22 (-9.94 to -2.51)	-5.21 (-6.86 to -3.57)
Nalgonda	75.6 (62.2-89.9)	48.0 (39.3-58.6)	29.8 (21.6-40.3)	-4.43 (-6.04 to -2.84)	-6.65 (-10.13 to -2.95)	-5.36 (-6.94 to -3.65)
Nirmal	64.1 (54.3-75.2)	47.8 (40.2-56.3)	30.1 (22.4-39.1)	-2.90 (-4.49 to -1.42)	-6.48 (-9.85 to -3.09)	-4.40 (-5.89 to -2.85)
Nizamabad	67.1 (57.2-78.4)	46.1 (39.2-53.7)	28.1 (21.1-36.3)	-3.69 (-5.27 to -2.13)	-6.91 (-10.24 to -3.55)	-5.03 (-6.49 to -3.52)
Peddapalli	66.6 (56.2-79.2)	43.3 (35.9-51.8)	26.8 (19.8-35.4)	-4.22 (-5.71 to -2.82)	-6.71 (-9.80 to -3.27)	-5.26 (-6.73 to -3.72)
Rajanna Sircilla	69.8 (59.0-82.1)	44.1 (36.9-52.5)	27.4 (20.3-35.7)	-4.48 (-6.11 to -2.90)	-6.67 (-10.02 to -3.14)	-5.40 (-6.86 to -3.89)
Rangareddy	74.9 (63.6-88.1)	42.9 (35.8-50.8)	26.8 (19.9-35.4)	-5.38 (-6.86 to -3.82)	-6.65 (-10.41 to -3.23)	-5.91 (-7.49 to -4.30)
Sangareddy	78.1 (67.4-89.1)	46.2 (39.1-53.8)	28.5 (21.2-36.9)	-5.10 (-6.54 to -3.66)	-6.76 (-10.40 to -3.38)	-5.79 (-7.29 to -4.33)
Siddipet	71.9 (61.4-83.3)	43.1 (36.3-50.9)	26.9 (20.1-35.6)	-4.98 (-6.54 to -3.46)	-6.57 (-10.07 to -3.01)	-5.64 (-7.12 to -4.13)
Suryapet	71.3 (59.9-83.4)	46.8 (38.5-56.0)	29.0 (21.6-38.5)	-4.13 (-5.71 to -2.54)	-6.65 (-10.08 to -3.06)	-5.19 (-6.80 to -3.55)
Vikarabad	86.2 (73.5-99.5)	48.5 (40.9-56.7)	31.7 (23.9-41.2)	-5.60 (-7.09 to -4.20)	-5.95 (-9.57 to -2.51)	-5.75 (-7.29 to -4.23)
Wanaparthy	97.0 (82.1-114.7)	59.5 (49.1-71.4)	38.4 (28.3-51.2)	-4.78 (-6.23 to -3.32)	-6.13 (-9.76 to -2.50)	-5.35 (-6.93 to -3.74)
Warangal Rural	67.1 (56.3-80.6)	42.1 (34.3-50.3)	26.8 (19.8-35.7)	-4.56 (-6.13 to -3.11)	-6.27 (-9.74 to -2.69)	-5.28 (-6.83 to -3.72)
Warangal Urban	63.4 (53.7-75.4)	39.2 (32.4-46.6)	24.1 (17.7-31.8)	-4.68 (-6.23 to -3.31)	-6.83 (-10.18 to -3.31)	-5.58 (-7.10 to -4.05)
Yadadri Bhuvanagiri	73.6 (61.8-86.0)	43.6 (36.3-52.0)	27.1 (19.9-36.0)	-5.11 (-6.66 to -3.61)	-6.62 (-10.16 to -3.11)	-5.74 (-7.23 to -4.11)
**Gujarat**	**72.7 (66.0-79.9)**	**49.9 (44.2-56.8)**	**38.1 (33.0-44.5)**	**-3.70 (-4.73 to -2.69)**	**-3.81 (-6.79 to -0.78)**	**-3.75 (-4.97 to -2.53)**
Ahmedabad	70.9 (62.9-80.9)	51.0 (43.9-58.7)	36.1 (28.2-45.9)	-3.24 (-4.62 to -1.93)	-4.90 (-8.08 to -1.82)	-3.93 (-5.33 to -2.55)
Amreli	56.8 (47.5-67.5)	36.3 (29.1-44.5)	30.9 (22.5-41.8)	-4.38 (-6.12 to -2.58)	-2.32 (-6.34 to 1.52)	-3.54 (-5.19 to -1.78)
Anand	93.5 (81.3-107.1)	65.8 (55.8-77.1)	48.1 (37.3-61.2)	-3.45 (-4.80 to -2.08)	-4.44 (-7.74 to -1.16)	-3.87 (-5.27 to -2.46)
Arvalli	98.0 (87.9-109.0)	71.5 (62.8-80.5)	47.8 (37.7-61.1)	-3.11 (-4.20 to -2.00)	-5.62 (-8.64 to -2.54)	-4.16 (-5.48 to -2.79)
Banaskantha	91.0 (80.7-102.9)	68.5 (60.1-77.4)	51.3 (40.3-64.0)	-2.80 (-4.05 to -1.56)	-4.11 (-7.20 to -0.91)	-3.35 (-4.72 to -1.98)
Bharuch	74.5 (65.3-84.2)	50.0 (42.9-57.8)	39.8 (30.3-50.9)	-3.90 (-5.27 to -2.52)	-3.26 (-6.65 to 0.27)	-3.64 (-5.05 to -2.15)
Bhavnagar	63.6 (55.4-73.3)	43.5 (36.5-51.9)	37.0 (28.2-48.5)	-3.73 (-5.24 to -2.25)	-2.36 (-5.89 to 1.31)	-3.18 (-4.66 to -1.66)
Botad	67.9 (58.7-77.8)	45.1 (37.5-53.0)	38.3 (29.0-48.6)	-4.02 (-5.56 to -2.59)	-2.37 (-5.89 to 1.16)	-3.35 (-4.79 to -1.83)
Chhotaudepur	90.8 (80.6-100.9)	63.2 (55.6-71.6)	51.3 (39.3-64.8)	-3.56 (-4.75 to -2.35)	-2.97 (-6.13 to 0.48)	-3.32 (-4.75 to -1.97)
Dahod	99.2 (87.5-111.4)	67.8 (59.5-76.2)	50.5 (38.9-63.6)	-3.74 (-4.90 to -2.59)	-4.12 (-7.31 to -0.69)	-3.90 (-5.31 to -2.52)
Dang	63.1 (55.8-70.7)	43.1 (37.6-49.2)	38.3 (29.0-49.0)	-3.77 (-5.13 to -2.45)	-1.63 (-5.00 to 2.09)	-2.90 (-4.38 to -1.44)
Devbhumi Dwarka	57.0 (44.2-71.1)	34.0 (25.7-44.2)	29.1 (20.3-41.3)	-5.06 (-7.26 to -2.74)	-2.30 (-6.15 to 1.84)	-3.94 (-5.81 to -1.89)
Gandhinagar	73.6 (64.3-84.8)	53.7 (45.2-63.0)	35.4 (26.6-45.9)	-3.11 (-4.56 to -1.74)	-5.80 (-9.03 to -2.57)	-4.23 (-5.72 to -2.79)
Gir Somnath	56.1 (46.5-67.2)	33.9 (26.8-41.9)	28.8 (20.8-39.5)	-4.92 (-6.89 to -2.95)	-2.39 (-6.47 to 1.67)	-3.90 (-5.70 to -1.97)
Jamnagar	57.0 (47.6-68.0)	34.6 (28.2-42.0)	28.6 (21.2-38.1)	-4.86 (-6.77 to -3.07)	-2.76 (-6.34 to 1.03)	-4.01 (-5.63 to -2.31)
Junagadh	55.7 (46.8-66.1)	32.1 (26.1-39.1)	25.5 (18.8-35.2)	-5.35 (-7.27 to -3.37)	-3.31 (-7.18 to 0.65)	-4.52 (-6.26 to -2.59)
Kachchh	69.0 (57.6-82.0)	45.2 (36.9-55.1)	40.7 (30.3-53.2)	-4.16 (-5.84 to -2.53)	-1.52 (-4.92 to 2.08)	-3.09 (-4.63 to -1.42)
Kheda	88.6 (78.9-99.7)	64.1 (55.9-72.5)	45.5 (35.7-57.5)	-3.19 (-4.46 to -2.00)	-4.84 (-8.11 to -1.68)	-3.88 (-5.25 to -2.54)
Mahesana	78.5 (70.2-88.3)	55.7 (48.3-63.6)	39.1 (30.6-49.7)	-3.38 (-4.58 to -2.16)	-4.99 (-8.07 to -1.96)	-4.05 (-5.40 to -2.71)
Mahisagar	96.1 (86.4-106.8)	67.9 (60.2-75.5)	47.0 (37.1-59.7)	-3.46 (-4.52 to -2.44)	-5.13 (-8.17 to -1.90)	-4.15 (-5.45 to -2.78)
Morbi	62.5 (51.4-75.3)	40.5 (32.5-50.2)	34.7 (25.3-45.7)	-4.26 (-6.00 to -2.67)	-2.25 (-5.69 to 1.32)	-3.44 (-5.05 to -1.91)
Narmada	86.9 (75.6-98.6)	59.0 (50.7-68.3)	48.5 (36.4-62.1)	-3.79 (-5.17 to -2.42)	-2.84 (-6.28 to 0.79)	-3.40 (-4.92 to -1.89)
Navsari	52.2 (44.9-59.6)	33.2 (28.3-39.0)	27.5 (20.5-36.3)	-4.42 (-5.96 to -2.91)	-2.71 (-6.37 to 1.11)	-3.73 (-5.33 to -2.15)
Panchmahal	93.6 (84.0-103.8)	65.7 (58.2-73.6)	47.1 (37.0-58.9)	-3.47 (-4.57 to -2.41)	-4.71 (-7.71 to -1.47)	-3.99 (-5.37 to -2.62)
Patan	82.4 (71.8-94.1)	59.3 (50.5-69.1)	46.1 (35.7-58.3)	-3.25 (-4.57 to -1.95)	-3.54 (-6.85 to -0.41)	-3.37 (-4.76 to -2.02)
Porbandar	55.4 (45.1-67.6)	32.5 (25.8-40.1)	25.8 (18.3-36.3)	-5.21 (-7.35 to -3.01)	-3.36 (-7.18 to 0.65)	-4.46 (-6.29 to -2.36)
Rajkot	54.3 (46.3-63.7)	33.0 (27.4-39.9)	26.7 (20.0-34.8)	-4.86 (-6.54 to -3.12)	-3.04 (-6.72 to 0.58)	-4.12 (-5.75 to -2.49)
Sabar Kantha	92.7 (83.5-103.1)	69.4 (61.2-78.6)	46.0 (36.2-58.3)	-2.85 (-3.90 to -1.77)	-5.77 (-8.81 to -2.75)	-4.07 (-5.40 to -2.76)
Surat	54.3 (46.1-63.2)	35.3 (29.3-42.0)	28.6 (21.1-38.2)	-4.24 (-5.84 to -2.69)	-3.00 (-6.60 to 0.86)	-3.74 (-5.34 to -2.12)
Surendranagar	69.0 (58.5-80.4)	47.0 (39.1-56.1)	38.6 (28.9-48.7)	-3.77 (-5.27 to -2.28)	-2.84 (-6.24 to 0.41)	-3.39 (-4.84 to -1.90)
Tapi	69.6 (61.5-78.0)	46.2 (40.2-52.8)	39.8 (30.4-50.7)	-4.02 (-5.32 to -2.70)	-2.15 (-5.53 to 1.39)	-3.26 (-4.77 to -1.79)
Vadodara	87.7 (77.4-98.6)	60.4 (52.1-69.2)	44.3 (34.4-56.1)	-3.67 (-4.89 to -2.38)	-4.40 (-7.71 to -0.99)	-3.98 (-5.36 to -2.63)
Valsad	50.4 (43.3-58.1)	33.2 (28.1-39.2)	28.5 (21.3-37.6)	-4.15 (-5.68 to -2.58)	-2.20 (-5.95 to 1.80)	-3.36 (-5.01 to -1.77)
**Manipur**	**47.3 (42.5-52.3)**	**31.4 (27.4-36.1)**	**24.9 (21.5-29.3)**	**-4.01 (-5.18 to -2.82)**	**-3.33 (-6.67 to -0.06)**	**-3.73 (-5.18 to -2.34)**
Bishnupur	44.9 (39.5-50.8)	30.5 (26.6-34.8)	24.0 (17.9-31.1)	-3.79 (-5.13 to -2.46)	-3.45 (-6.89 to -0.03)	-3.66 (-5.20 to -2.19)
Chandel	44.0 (38.2-50.3)	33.4 (28.7-38.5)	27.4 (20.6-35.7)	-2.73 (-4.16 to -1.32)	-2.91 (-6.41 to 0.66)	-2.81 (-4.40 to -1.32)
Churachandpur	49.1 (43.5-55.1)	34.7 (30.7-39.0)	27.3 (20.8-34.9)	-3.40 (-4.63 to -2.19)	-3.46 (-6.83 to 0.05)	-3.43 (-4.89 to -2.03)
Imphal East	44.0 (38.8-49.5)	28.8 (25.2-32.4)	22.5 (17.2-28.7)	-4.10 (-5.31 to -2.86)	-3.63 (-7.11 to -0.35)	-3.91 (-5.37 to -2.51)
Imphal West	42.4 (37.4-47.8)	28.5 (25.0-32.2)	21.4 (16.2-27.3)	-3.92 (-5.16 to -2.65)	-4.05 (-7.45 to -0.78)	-3.98 (-5.44 to -2.54)
Jiribam	87.2 (77.8-96.6)	57.3 (51.3-63.9)	42.6 (33.6-53.7)	-4.09 (-5.10 to -3.07)	-4.24 (-7.37 to -1.00)	-4.16 (-5.51 to -2.79)
Kakching	41.5 (36.1-47.3)	28.8 (25.0-33.0)	23.2 (17.3-30.3)	-3.61 (-5.07 to -2.16)	-3.13 (-6.66 to 0.39)	-3.42 (-5.01 to -1.91)
Kamjong	44.9 (38.7-52.0)	29.7 (25.3-34.2)	26.0 (19.5-34.0)	-4.06 (-5.33 to -2.74)	-1.93 (-5.33 to 1.59)	-3.20 (-4.72 to -1.68)
Kangpokpi	45.1 (39.8-50.6)	29.2 (25.6-32.8)	23.5 (17.9-29.8)	-4.35 (-5.52 to -3.14)	-2.99 (-6.39 to 0.26)	-3.80 (-5.24 to -2.39)
Noney	64.8 (58.0-72.2)	43.6 (38.7-48.9)	33.8 (26.2-42.7)	-3.90 (-5.04 to -2.80)	-3.64 (-6.86 to -0.35)	-3.80 (-5.23 to -2.40)
Pherzawl	64.0 (57.7-70.7)	42.6 (38.1-47.2)	33.5 (26.1-42.5)	-4.15 (-5.16 to -3.14)	-3.45 (-6.71 to -0.14)	-3.87 (-5.23 to -2.50)
Senapati	53.3 (47.6-59.6)	32.9 (29.4-36.7)	26.9 (20.9-33.6)	-4.73 (-5.77 to -3.59)	-2.87 (-6.08 to 0.18)	-3.97 (-5.32 to -2.63)
Tamenglong	67.7 (60.6-75.2)	42.7 (38.2-47.5)	33.8 (26.3-42.3)	-4.52 (-5.51 to -3.46)	-3.35 (-6.51 to -0.22)	-4.04 (-5.37 to -2.73)
Tengnoupal	44.3 (38.3-50.9)	30.7 (26.1-35.6)	26.0 (19.3-34.1)	-3.60 (-4.98 to -2.25)	-2.50 (-5.94 to 1.08)	-3.16 (-4.68 to -1.61)
Thoubal	41.8 (36.6-47.5)	28.1 (24.3-32.0)	22.3 (16.7-28.9)	-3.89 (-5.24 to -2.53)	-3.30 (-6.81 to 0.17)	-3.66 (-5.18 to -2.17)
Ukhrul	45.4 (40.1-51.6)	28.6 (25.1-32.4)	25.2 (19.3-31.6)	-4.53 (-5.69 to -3.31)	-1.84 (-5.13 to 1.48)	-3.44 (-4.90 to -2.04)
**Jammu & Kashmir[†]**	**57.9 (52.4-63.5)**	**43.5 (38.2-49.5)**	**33.4 (28.9-39.0)**	**-2.81 (-3.97 to -1.63)**	**-3.75 (-6.64 to -0.96)**	**-3.20 (-4.48 to -2.09)**
Anantnag	59.7 (52.8-68.1)	46.6 (40.7-53.0)	35.0 (27.7-43.4)	-2.46 (-3.79 to -1.13)	-4.07 (-7.02 to -1.14)	-3.13 (-4.45 to -1.91)
Badgam	60.2 (53.3-67.7)	45.7 (40.1-52.0)	33.4 (26.5-41.5)	-2.73 (-4.08 to -1.40)	-4.44 (-7.43 to -1.41)	-3.44 (-4.87 to -2.17)

Bandipore	68.8 (60.1-78.7)	51.8 (45.0-59.8)	38.6 (30.1-48.2)	-2.80 (-4.22 to -1.21)	-4.12 (-7.37 to -0.95)	-3.35 (-4.79 to -2.04)
Baramulla	70.4 (63.6-77.8)	55.7 (50.3-62.1)	41.0 (35.0-47.6)	-2.81 (-4.16 to -1.30)	-4.53 (-7.61 to -1.49)	-3.53 (-4.94 to -2.23)
Doda	48.9 (42.3-56.6)	41.5 (35.9-48.3)	32.7 (25.5-41.5)	-1.64 (-2.93 to -0.22)	-3.38 (-6.42 to -0.52)	-2.36 (-3.80 to -1.04)
Ganderbal	68.7 (60.1-78.4)	52.1 (45.3-59.9)	39.1 (30.8-49.5)	-2.73 (-4.14 to -1.24)	-4.07 (-7.28 to -0.93)	-3.29 (-4.73 to -1.96)
Jammu	53.7 (48.7-58.8)	39.9 (35.9-44.0)	33.0 (27.7-38.7)	-3.93 (-5.19 to -2.64)	-2.75 (-5.87 to 0.33)	-3.45 (-4.76 to -2.17)
Kargil	78.7 (68.4-90.6)	61.0 (53.2-70.3)	46.7 (37.2-57.5)	-2.55 (-4.10 to -1.07)	-3.59 (-6.81 to -0.40)	-2.99 (-4.38 to -1.69)
Kathua	54.5 (48.6-60.8)	39.6 (35.3-44.6)	31.5 (25.3-38.3)	-3.36 (-4.58 to -2.14)	-3.34 (-6.29 to -0.37)	-3.36 (-4.64 to -2.05)
Kishtwar	59.2 (51.5-69.0)	48.6 (42.1-56.2)	38.6 (30.4-48.4)	-1.95 (-3.29 to -0.63)	-3.29 (-6.20 to -0.38)	-2.51 (-3.90 to -1.28)
Kulgam	53.2 (47.2-60.2)	41.8 (37.0-47.6)	31.2 (25.0-38.8)	-2.38 (-3.64 to -1.13)	-4.10 (-7.00 to -1.27)	-3.10 (-4.40 to -1.89)
Kupwara	69.7 (61.8-78.4)	53.4 (47.5-60.2)	39.3 (32.5-46.7)	-2.93 (-4.34 to -1.42)	-4.28 (-7.52 to -1.17)	-3.49 (-4.98 to -2.15)
Leh	61.7 (49.6-75.6)	44.2 (35.5-55.3)	34.6 (25.7-45.8)	-3.34 (-5.21 to -1.54)	-3.41 (-6.85 to 0.00)	-3.37 (-4.99 to -1.72)
PoJK	80.7 (69.4-92.3)	66.1 (57.1-75.7)	47.3 (39.0-56.7)	-1.98 (-4.10 to -0.07)	-4.67 (-6.81 to -0.40)	-3.09 (-4.38 to -1.69)
Poonch	58.2 (52.8-63.9)	44.9 (40.6-49.8)	36.1 (31.0-42.0)	-3.48 (-4.82 to -2.17)	-2.75 (-5.84 to 0.23)	-3.19 (-4.52 to -1.95)
Pulwama	59.6 (52.6-67.6)	45.7 (40.0-52.1)	34.1 (26.9-42.2)	-2.62 (-3.95 to -1.31)	-4.16 (-7.04 to -1.21)	-3.26 (-4.61 to -2.02)
Rajouri	51.1 (45.3-56.6)	38.0 (34.1-42.6)	32.2 (27.1-38.2)	-3.58 (-4.85 to -2.34)	-2.14 (-5.26 to 0.81)	-2.99 (-4.32 to -1.77)
Ramban	47.7 (41.6-54.8)	39.1 (34.1-44.8)	30.8 (24.3-38.4)	-1.96 (-3.23 to -0.65)	-3.42 (-6.46 to -0.54)	-2.57 (-3.89 to -1.33)
Reasi	46.9 (41.1-52.7)	35.8 (31.6-40.7)	28.6 (22.8-35.6)	-2.65 (-3.86 to -1.41)	-3.22 (-6.23 to -0.28)	-2.89 (-4.14 to -1.67)
Samba	56.7 (51.0-63.1)	42.1 (37.8-46.9)	34.2 (28.5-40.7)	-3.61 (-4.98 to -2.25)	-3.30 (-6.48 to -0.17)	-3.49 (-4.90 to -2.13)
Shopian	56.1 (49.8-63.1)	43.4 (38.4-49.1)	32.3 (25.9-39.9)	-2.54 (-3.82 to -1.30)	-4.20 (-7.10 to -1.29)	-3.23 (-4.54 to -2.02)
Srinagar	61.6 (54.2-70.1)	45.9 (39.9-52.8)	32.7 (25.7-40.8)	-2.93 (-4.33 to -1.44)	-4.82 (-7.85 to -1.73)	-3.72 (-5.15 to -2.38)
Udhampur	48.3 (42.6-54.5)	37.2 (32.8-42.5)	29.1 (22.9-36.1)	-2.55 (-3.77 to -1.26)	-3.51 (-6.61 to -0.54)	-2.95 (-4.26 to -1.66)
**Haryana**	**73.2 (66.5-80.5)**	**54.6 (48.4-62.2)**	**39.3 (34.0-46.0)**	**-2.88 (-3.62 to -2.12)**	**-4.63 (-7.21 to -2.04)**	**-3.61 (-4.73 to -2.57)**
Ambala	63.6 (57.5-70.1)	45.6 (40.3-51.1)	32.0 (25.5-39.1)	-3.20 (-4.17 to -2.12)	-4.94 (-7.63 to -2.09)	-3.93 (-5.14 to -2.74)
Bhiwani	71.8 (64.7-79.2)	51.7 (46.0-57.2)	37.0 (29.4-45.9)	-3.24 (-4.22 to -2.26)	-4.71 (-7.39 to -1.84)	-3.85 (-5.09 to -2.65)
Charkhi Dadri	72.8 (64.1-81.5)	52.5 (45.9-59.3)	37.2 (29.2-46.6)	-3.22 (-4.37 to -2.09)	-4.87 (-7.70 to -1.77)	-3.91 (-5.25 to -2.66)
Faridabad	70.9 (64.5-77.4)	58.9 (53.0-64.8)	41.3 (33.0-50.9)	-1.81 (-2.69 to -0.96)	-4.77 (-7.45 to -1.98)	-3.04 (-4.21 to -1.90)
Fatehabad	72.6 (64.7-81.1)	51.6 (45.3-58.2)	39.5 (31.1-48.7)	-3.38 (-4.41 to -2.25)	-3.80 (-6.68 to -0.80)	-3.56 (-4.91 to -2.35)
Gurugram	70.1 (64.1-76.1)	56.3 (51.0-61.7)	40.8 (33.0-50.6)	-2.21 (-3.09 to -1.35)	-4.54 (-7.28 to -1.74)	-3.18 (-4.39 to -2.03)
Hisar	72.4 (65.1-79.6)	52.1 (46.5-57.6)	38.5 (30.8-47.4)	-3.24 (-4.16 to -2.26)	-4.23 (-6.93 to -1.40)	-3.66 (-4.92 to -2.50)
Jhajjar	73.9 (66.3-81.4)	53.9 (47.9-60.1)	39.5 (31.7-49.5)	-3.13 (-4.10 to -2.12)	-4.44 (-7.25 to -1.52)	-3.68 (-4.97 to -2.47)
Jind	79.5 (71.6-88.0)	56.9 (50.5-63.6)	40.8 (32.3-50.5)	-3.29 (-4.22 to -2.33)	-4.68 (-7.54 to -1.88)	-3.87 (-5.16 to -2.69)
Kaithal	78.3 (69.6-87.5)	54.2 (47.4-61.9)	38.5 (29.7-48.5)	-3.63 (-4.65 to -2.57)	-4.81 (-7.69 to -1.91)	-4.12 (-5.44 to -2.92)
Karnal	79.7 (72.1-87.7)	57.7 (51.4-64.3)	39.9 (31.7-49.2)	-3.22 (-4.13 to -2.26)	-5.19 (-7.96 to -2.47)	-4.04 (-5.24 to -2.87)
Kurukshetra	71.1 (64.2-78.5)	50.4 (44.7-56.5)	35.6 (28.2-44.0)	-3.33 (-4.24 to -2.37)	-4.91 (-7.62 to -2.10)	-3.99 (-5.16 to -2.82)
Mahendragarh	62.8 (56.5-69.3)	45.4 (40.5-50.7)	32.0 (25.4-39.9)	-3.17 (-4.12 to -2.17)	-4.98 (-7.75 to -2.14)	-3.92 (-5.18 to -2.67)
Nuh	85.8 (77.7-94.6)	74.2 (66.3-82.1)	57.1 (45.2-71.3)	-1.42 (-2.47 to -0.45)	-3.77 (-6.63 to -0.84)	-2.40 (-3.64 to -1.18)
Palwal	86.9 (78.4-95.7)	72.4 (65.3-79.8)	54.6 (43.4-67.6)	-1.84 (-2.81 to -0.90)	-4.16 (-6.94 to -1.29)	-2.81 (-4.01 to -1.57)
Panchkula	58.3 (51.8-65.5)	43.4 (37.6-49.3)	31.3 (24.4-38.9)	-2.86 (-4.00 to -1.69)	-4.91 (-7.70 to -1.95)	-3.71 (-4.98 to -2.48)
Panipat	77.5 (70.3-84.7)	56.4 (50.5-62.6)	38.4 (30.5-47.6)	-3.12 (-4.01 to -2.20)	-5.41 (-8.18 to -2.74)	-4.08 (-5.24 to -2.92)
Rewari	70.6 (63.8-77.5)	54.4 (48.9-60.2)	38.8 (31.0-48.5)	-2.52 (-3.41 to -1.57)	-4.85 (-7.67 to -1.97)	-3.49 (-4.77 to -2.29)
Rohtak	75.8 (68.1-83.6)	54.5 (48.7-60.5)	39.3 (31.3-49.2)	-3.25 (-4.21 to -2.25)	-4.64 (-7.39 to -1.74)	-3.82 (-5.07 to -2.61)
Sirsa	61.8 (54.7-69.4)	43.3 (38.4-49.2)	34.6 (27.2-43.5)	-3.51 (-4.55 to -2.33)	-3.16 (-6.08 to -0.07)	-3.37 (-4.72 to -2.08)
Sonipat	78.8 (71.8-86.1)	56.8 (51.3-62.8)	40.1 (32.0-49.1)	-3.23 (-4.09 to -2.35)	-4.91 (-7.59 to -2.10)	-3.93 (-5.08 to -2.77)
Yamunanagar	72.0 (65.0-79.4)	54.6 (48.3-61.2)	37.4 (29.8-45.9)	-2.73 (-3.71 to -1.64)	-5.36 (-8.09 to -2.51)	-3.83 (-5.06 to -2.60)
**Uttarakhand**	**58.1 (52.1-64.5)**	**43.0 (37.8-49.1)**	**30.3 (26.2-35.5)**	**-2.97 (-3.79 to -2.12)**	**-4.90 (-7.47 to -2.25)**	**-3.77 (-4.86 to -2.70)**
Almora	49.0 (44.5-54.1)	32.5 (29.1-36.2)	23.0 (18.5-28.3)	-3.97 (-4.96 to -2.97)	-4.89 (-7.55 to -2.12)	-4.35 (-5.50 to -3.16)
Bageshwar	51.8 (46.3-57.8)	35.0 (30.7-39.5)	24.1 (18.9-30.7)	-3.84 (-5.04 to -2.70)	-5.24 (-8.05 to -2.10)	-4.42 (-5.73 to -3.06)
Chamoli	44.7 (38.6-51.4)	32.1 (27.4-37.6)	22.4 (17.3-28.5)	-3.24 (-4.51 to -1.98)	-5.07 (-7.91 to -1.97)	-4.00 (-5.34 to -2.57)
Champawat	63.9 (57.7-71.0)	40.3 (35.9-45.1)	27.2 (21.8-34.3)	-4.52 (-5.51 to -3.52)	-5.53 (-8.32 to -2.78)	-4.94 (-6.19 to -3.67)
Dehradun	53.0 (48.0-58.8)	41.8 (37.2-46.8)	29.9 (24.0-36.8)	-2.34 (-3.38 to -1.35)	-4.72 (-7.54 to -1.76)	-3.33 (-4.56 to -2.09)
Haridwar	71.1 (64.6-78.4)	54.0 (48.2-60.2)	38.5 (31.2-47.1)	-2.73 (-3.68 to -1.80)	-4.71 (-7.47 to -1.87)	-3.55 (-4.73 to -2.43)
Nainital	53.3 (48.4-59.2)	37.7 (33.8-41.8)	26.6 (21.6-33.0)	-3.41 (-4.32 to -2.45)	-4.88 (-7.49 to -2.04)	-4.02 (-5.20 to -2.83)
Pauri Garhwal	52.4 (47.1-57.9)	39.7 (35.3-44.5)	28.0 (22.6-34.4)	-2.69 (-3.68 to -1.70)	-4.92 (-7.60 to -2.09)	-3.62 (-4.76 to -2.48)
Pithoragarh	57.2 (48.9-65.6)	37.2 (32.2-43.2)	24.7 (19.0-32.3)	-4.21 (-5.52 to -2.95)	-5.72 (-8.59 to -2.47)	-4.84 (-6.18 to -3.37)
Rudraprayag	45.4 (39.5-51.7)	35.0 (29.7-41.1)	24.6 (18.7-31.7)	-2.56 (-3.88 to -1.18)	-5.00 (-7.98 to -1.81)	-3.57 (-4.96 to -2.15)
Tehri Garhwal	49.8 (44.4-55.5)	39.2 (34.3-44.4)	27.8 (22.0-34.6)	-2.36 (-3.47 to -1.23)	-4.87 (-7.68 to -1.85)	-3.41 (-4.67 to -2.19)
Udham Singh Nagar	65.4 (59.5-72.3)	48.1 (43.2-53.0)	33.4 (27.1-41.5)	-3.13 (-4.00 to -2.21)	-5.09 (-7.70 to -2.31)	-3.95 (-5.13 to -2.81)
Uttarkashi	52.3 (46.3-58.8)	43.0 (37.2-49.0)	31.2 (24.2-39.1)	-1.92 (-3.26 to -0.65)	-4.52 (-7.40 to -1.48)	-3.00 (-4.39 to -1.65)
**Tamil Nadu**	**51.4 (47.0-56.4)**	**30.3 (25.2-36.4)**	**19.6 (16.9-23.0)**	**-5.13 (-6.27 to -3.93)**	**-6.13 (-9.90 to -2.31)**	**-5.55 (-7.06 to -3.93)**
Ariyalur	60.5 (52.4-69.5)	35.8 (30.3-42.3)	24.3 (17.5-33.0)	-5.09 (-6.60 to -3.36)	-5.52 (-9.61 to -1.42)	-5.28 (-7.17 to -3.42)
Chennai	36.9 (30.8-44.2)	20.4 (16.4-25.2)	14.3 (10.1-20.1)	-5.76 (-7.71 to -3.76)	-5.08 (-9.12 to -0.30)	-5.49 (-7.38 to -3.57)
Coimbatore	41.3 (35.3-48.2)	24.7 (20.7-29.0)	14.5 (10.6-19.9)	-5.03 (-6.56 to -3.41)	-7.41 (-11.48 to -3.05)	-6.03 (-7.78 to -4.23)
Cuddalore	57.4 (50.0-65.4)	32.5 (27.9-37.8)	21.9 (16.1-29.5)	-5.54 (-6.99 to -3.91)	-5.61 (-9.71 to -1.42)	-5.58 (-7.42 to -3.75)
Dharmapuri	60.6 (53.5-69.0)	37.5 (32.3-43.2)	22.6 (16.8-29.6)	-4.67 (-6.01 to -3.30)	-7.09 (-10.67 to -3.19)	-5.68 (-7.23 to -4.05)
Dindigul	54.0 (46.5-62.4)	33.4 (28.3-39.7)	20.5 (14.7-28.1)	-4.70 (-6.22 to -3.24)	-6.83 (-10.92 to -2.58)	-5.59 (-7.35 to -3.83)
Erode	57.9 (50.4-67.1)	35.9 (30.3-41.5)	21.2 (15.5-28.4)	-4.72 (-6.12 to -3.28)	-7.35 (-11.12 to -3.35)	-5.82 (-7.50 to -4.10)
Kanchipuram	46.4 (39.2-54.6)	24.4 (20.1-29.2)	17.7 (12.9-25.1)	-6.24 (-8.03 to -4.39)	-4.52 (-8.74 to 0.07)	-5.54 (-7.45 to -3.66)
Kanniyakumari	31.5 (24.1-40.0)	19.0 (14.3-24.0)	11.2 (7.2-16.5)	-4.95 (-7.22 to -2.53)	-7.44 (-11.91 to -2.45)	-5.99 (-8.34 to -3.74)
Karur	53.0 (46.1-60.9)	32.7 (27.6-38.0)	20.3 (14.9-27.1)	-4.71 (-6.16 to -3.24)	-6.75 (-10.74 to -2.51)	-5.56 (-7.24 to -3.80)
Krishnagiri	66.6 (58.0-75.8)	40.1 (34.5-46.7)	24.3 (17.9-31.8)	-4.97 (-6.36 to -3.61)	-7.01 (-10.67 to -3.21)	-5.82 (-7.45 to -4.20)
Madurai	57.9 (49.5-67.8)	35.5 (29.9-41.9)	22.5 (15.5-30.5)	-4.76 (-6.41 to -3.19)	-6.44 (-10.81 to -1.90)	-5.46 (-7.42 to -3.71)
Nagapattinam	52.7 (44.5-61.7)	29.9 (24.9-35.9)	20.5 (14.4-28.2)	-5.51 (-7.22 to -3.66)	-5.39 (-9.60 to -1.15)	-5.47 (-7.47 to -3.54)
Namakkal	51.4 (43.9-59.9)	32.0 (26.6-37.6)	19.3 (13.9-26.3)	-4.62 (-6.07 to -3.09)	-7.12 (-11.18 to -3.10)	-5.67 (-7.37 to -3.93)
Perambalur	55.4 (48.6-63.3)	33.4 (28.5-38.8)	21.9 (15.8-29.3)	-4.93 (-6.35 to -3.40)	-6.00 (-10.13 to -1.87)	-5.38 (-7.19 to -3.58)
Pudukkottai	58.4 (50.9-67.6)	34.0 (28.9-39.5)	22.5 (16.4-30.3)	-5.29 (-6.83 to -3.74)	-5.80 (-10.00 to -1.60)	-5.51 (-7.41 to -3.67)
Ramanathapuram	47.5 (37.7-58.2)	27.4 (21.7-33.9)	18.2 (12.4-25.7)	-5.34 (-7.35 to -3.34)	-5.81 (-10.33 to -1.21)	-5.54 (-7.68 to -3.56)
Salem	55.3 (47.9-63.6)	34.6 (29.2-40.2)	20.7 (15.3-27.6)	-4.58 (-5.93 to -3.22)	-7.17 (-11.06 to -3.21)	-5.66 (-7.35 to -4.01)
Sivaganga	54.7 (46.2-64.5)	31.2 (26.0-36.8)	20.6 (14.5-27.9)	-5.43 (-7.15 to -3.71)	-5.91 (-10.12 to -1.58)	-5.63 (-7.58 to -3.80)
Thanjavur	57.4 (49.6-66.7)	33.6 (28.4-39.6)	22.5 (16.2-30.5)	-5.20 (-6.84 to -3.42)	-5.70 (-9.82 to -1.57)	-5.41 (-7.31 to -3.53)
The Nilgiris	46.3 (40.1-53.1)	27.4 (23.3-32.1)	16.1 (11.8-21.8)	-5.17 (-6.66 to -3.54)	-7.44 (-11.44 to -3.27)	-6.12 (-7.90 to -4.36)
Theni	56.0 (46.5-67.6)	34.4 (28.1-41.4)	21.6 (14.6-30.3)	-4.76 (-6.50 to -3.08)	-6.57 (-10.95 to -1.97)	-5.52 (-7.50 to -3.63)
Thiruvallur	43.4 (36.5-51.3)	23.5 (19.3-28.5)	16.9 (12.1-23.6)	-5.98 (-7.88 to -4.08)	-4.72 (-8.77 to -0.04)	-5.47 (-7.34 to -3.61)
Thiruvarur	55.3 (47.2-64.7)	32.0 (26.7-38.0)	22.0 (15.5-30.5)	-5.35 (-7.09 to -3.51)	-5.28 (-9.60 to -1.08)	-5.33 (-7.36 to -3.37)
Thoothukudi	46.3 (38.1-55.5)	29.0 (23.2-35.4)	18.7 (12.6-26.3)	-4.55 (-6.60 to -2.60)	-6.20 (-10.62 to -1.53)	-5.25 (-7.32 to -3.27)
Tiruchirappalli	55.2 (48.1-63.3)	33.6 (28.6-38.9)	21.4 (15.6-28.3)	-4.84 (-6.26 to -3.41)	-6.36 (-10.61 to -2.25)	-5.48 (-7.24 to -3.69)
Tirunelveli	42.0 (35.3-50.1)	26.2 (21.2-31.3)	16.5 (11.0-23.2)	-4.62 (-6.50 to -2.63)	-6.52 (-10.84 to -1.78)	-5.41 (-7.54 to -3.37)
Tiruppur	49.8 (41.4-59.2)	30.1 (25.0-36.4)	17.8 (12.7-24.6)	-4.90 (-6.40 to -3.39)	-7.33 (-11.23 to -3.01)	-5.92 (-7.63 to -4.13)
Tiruvannamalai	55.1 (47.3-64.0)	31.5 (26.8-37.2)	21.6 (16.0-29.0)	-5.41 (-6.93 to -3.87)	-5.37 (-9.20 to -1.31)	-5.40 (-7.11 to -3.65)

Vellore	54.9 (47.8-62.9)	31.8 (27.6-37.0)	21.3 (15.8-28.7)	-5.29 (-6.88 to -3.73)	-5.66 (-9.46 to -1.62)	-5.45 (-7.22 to -3.65)
Viluppuram	56.7 (49.4-65.2)	32.5 (28.0-37.6)	22.1 (16.6-29.4)	-5.42 (-6.85 to -3.97)	-5.53 (-9.44 to -1.46)	-5.47 (-7.25 to -3.71)
Virudhunagar	56.0 (47.8-65.4)	34.8 (28.9-41.0)	22.5 (15.5-30.8)	-4.65 (-6.45 to -2.92)	-6.17 (-10.45 to -1.71)	-5.29 (-7.39 to -3.39)
**Mizoram**	**44.0 (39.6-48.8)**	**44.1 (38.5-50.5)**	**36.3 (31.4-42.5)**	**0.02 (-1.14 to 1.12)**	**-2.81 (-6.10 to 0.54)**	**-1.16 (-2.55 to 0.24)**
Aizawl	41.7 (37.7-46.2)	40.2 (35.7-44.7)	33.8 (26.7-43.5)	-0.37 (-1.47 to 0.74)	-2.54 (-5.76 to 0.91)	-1.27 (-2.68 to 0.17)
Champhai	36.1 (31.6-41.0)	39.8 (34.4-45.3)	32.4 (25.1-41.7)	0.99 (-0.33 to 2.26)	-2.99 (-6.28 to 0.48)	-0.68 (-2.17 to 0.83)
Kolasib	45.6 (41.1-50.4)	42.0 (37.5-46.9)	34.6 (27.1-44.4)	-0.80 (-1.82 to 0.22)	-2.82 (-6.05 to 0.60)	-1.64 (-3.04 to -0.20)
Lawngtlai	50.1 (44.1-56.6)	52.6 (46.0-59.7)	43.3 (33.5-55.9)	0.64 (-0.78 to 2.08)	-2.73 (-6.26 to 0.74)	-0.77 (-2.35 to 0.82)
Lunglei	46.2 (40.6-52.1)	47.0 (41.1-53.3)	37.6 (29.0-48.3)	0.24 (-1.07 to 1.62)	-3.19 (-6.61 to 0.40)	-1.19 (-2.68 to 0.22)
Mamit	52.7 (47.4-59.0)	48.0 (42.7-54.2)	39.7 (30.9-50.9)	-0.84 (-2.01 to 0.32)	-2.72 (-6.05 to 0.73)	-1.62 (-3.08 to -0.16)
Saiha	51.5 (44.3-59.5)	58.7 (50.6-67.3)	48.1 (36.8-61.6)	1.32 (-0.21 to 2.91)	-2.96 (-6.46 to 0.55)	-0.47 (-2.11 to 1.15)
Serchhip	41.5 (36.9-46.7)	41.8 (36.6-46.9)	34.1 (26.6-43.6)	0.10 (-1.18 to 1.30)	-2.97 (-6.29 to 0.45)	-1.18 (-2.67 to 0.27)
**Maharashtra**	**54.0 (49.2-59.3)**	**36.7 (30.4-44.0)**	**26.1 (22.5-30.6)**	**-3.78 (-4.71 to -2.84)**	**-4.83 (-7.88 to -1.92)**	**-4.22 (-5.38 to -3.01)**
Ahmednagar	51.6 (44.9-59.6)	35.1 (29.7-40.7)	24.8 (18.4-32.2)	-3.79 (-5.25 to -2.31)	-4.94 (-8.53 to -1.33)	-4.27 (-5.75 to -2.71)
Akola	57.2 (49.8-65.2)	41.1 (35.3-47.7)	28.2 (21.2-35.7)	-3.26 (-4.55 to -1.99)	-5.32 (-8.93 to -1.91)	-4.12 (-5.76 to -2.66)
Amravati	64.4 (55.6-74.9)	41.9 (35.6-49.3)	29.1 (21.5-37.7)	-4.21 (-5.52 to -2.90)	-5.14 (-8.72 to -1.85)	-4.60 (-6.16 to -3.14)
Aurangabad	54.4 (47.3-62.4)	38.9 (33.1-45.0)	27.0 (19.9-34.8)	-3.31 (-4.75 to -1.97)	-5.15 (-8.66 to -1.56)	-4.08 (-5.54 to -2.59)
Beed	52.1 (45.7-59.3)	36.2 (31.3-41.5)	26.0 (19.3-33.2)	-3.58 (-4.99 to -2.16)	-4.68 (-8.24 to -1.16)	-4.04 (-5.65 to -2.58)
Bhandara	75.3 (66.1-85.7)	45.6 (39.4-52.8)	33.2 (25.6-43.5)	-4.86 (-6.14 to -3.57)	-4.66 (-7.88 to -1.30)	-4.78 (-6.10 to -3.27)
Buldhana	59.3 (51.4-67.8)	43.9 (38.0-50.4)	29.8 (22.4-38.1)	-2.95 (-4.26 to -1.70)	-5.45 (-9.12 to -2.04)	-4.00 (-5.55 to -2.50)
Chandrapur	66.9 (57.6-76.6)	45.3 (38.4-53.2)	33.7 (25.8-44.1)	-3.82 (-5.22 to -2.36)	-4.22 (-7.52 to -0.86)	-3.99 (-5.41 to -2.44)
Dhule	63.5 (54.9-72.7)	45.2 (38.6-52.4)	30.7 (23.6-39.7)	-3.35 (-4.69 to -2.06)	-5.42 (-8.78 to -2.04)	-4.22 (-5.65 to -2.74)
Gadchiroli	75.9 (65.9-87.0)	52.3 (45.2-60.7)	40.0 (31.2-51.3)	-3.68 (-4.91 to -2.41)	-3.83 (-6.95 to -0.55)	-3.74 (-5.01 to -2.30)
Gondia	82.0 (72.7-92.0)	50.4 (44.3-57.5)	35.1 (27.2-45.3)	-4.76 (-6.02 to -3.52)	-5.06 (-8.20 to -1.74)	-4.89 (-6.19 to -3.41)
Hingoli	56.0 (48.6-63.3)	44.5 (38.4-51.1)	30.2 (22.7-39.7)	-2.28 (-3.62 to -0.96)	-5.46 (-9.07 to -1.77)	-3.61 (-5.25 to -2.05)
Jalgaon	66.1 (57.2-75.0)	46.9 (40.5-53.8)	31.0 (23.5-39.9)	-3.37 (-4.70 to -2.07)	-5.82 (-9.27 to -2.37)	-4.39 (-5.88 to -2.92)
Jalna	57.4 (50.1-65.4)	43.2 (37.0-49.4)	30.6 (22.7-39.8)	-2.80 (-4.21 to -1.51)	-4.92 (-8.45 to -1.30)	-3.68 (-5.33 to -2.18)
Kolhapur	52.2 (43.6-62.2)	28.7 (23.7-34.5)	19.6 (14.1-26.7)	-5.79 (-7.53 to -4.00)	-5.38 (-9.51 to -1.27)	-5.63 (-7.44 to -3.91)
Latur	52.9 (45.3-60.9)	34.4 (28.9-40.6)	23.8 (17.6-31.1)	-4.23 (-5.80 to -2.63)	-5.18 (-8.81 to -1.51)	-4.62 (-6.31 to -2.96)
Mumbai City	40.7 (34.0-48.8)	29.8 (23.9-36.7)	22.0 (15.5-29.9)	-3.09 (-5.14 to -0.83)	-4.32 (-8.46 to 0.04)	-3.61 (-5.37 to -1.76)
Mumbai Suburban	38.9 (32.9-45.8)	29.2 (24.0-35.4)	21.7 (15.5-29.0)	-2.84 (-4.78 to -0.68)	-4.25 (-8.32 to -0.05)	-3.43 (-5.18 to -1.60)
Nagpur	66.9 (58.7-76.1)	40.6 (35.1-46.5)	30.1 (23.1-39.1)	-4.87 (-6.16 to -3.62)	-4.27 (-7.64 to -1.00)	-4.63 (-6.06 to -3.11)
Nanded	58.5 (50.4-67.4)	43.1 (37.0-50.1)	29.8 (22.4-38.4)	-3.00 (-4.38 to -1.62)	-5.18 (-8.64 to -1.79)	-3.91 (-5.54 to -2.42)
Nandurbar	81.4 (71.8-90.6)	56.7 (49.9-64.2)	38.6 (29.8-48.6)	-3.51 (-4.75 to -2.26)	-5.48 (-8.71 to -2.16)	-4.34 (-5.71 to -2.87)
Nashik	54.8 (47.9-62.6)	38.1 (32.8-44.2)	26.7 (20.2-34.5)	-3.56 (-4.96 to -2.14)	-5.05 (-8.44 to -1.56)	-4.18 (-5.74 to -2.74)
Osmanabad	53.9 (47.0-61.3)	34.0 (29.2-39.4)	24.3 (18.3-31.5)	-4.49 (-5.94 to -3.01)	-4.77 (-8.43 to -1.24)	-4.61 (-6.29 to -3.05)
Palghar	49.4 (42.4-57.2)	35.1 (29.8-41.2)	26.9 (20.2-35.2)	-3.36 (-4.96 to -1.65)	-3.80 (-7.66 to 0.14)	-3.55 (-5.16 to -1.83)
Parbhani	59.3 (51.4-68.2)	46.0 (39.1-53.4)	31.1 (23.2-40.5)	-2.48 (-3.82 to -1.08)	-5.51 (-8.99 to -2.00)	-3.75 (-5.41 to -2.21)
Pune	45.4 (38.4-53.4)	29.4 (24.1-35.0)	21.0 (15.6-28.0)	-4.26 (-5.87 to -2.47)	-4.79 (-8.70 to -0.76)	-4.48 (-6.11 to -2.80)
Raigad	43.8 (37.7-51.6)	30.2 (24.9-36.0)	22.4 (16.1-29.8)	-3.67 (-5.51 to -1.65)	-4.26 (-8.23 to -0.08)	-3.92 (-5.72 to -2.11)
Ratnagiri	46.1 (36.9-56.2)	29.0 (22.7-36.6)	20.6 (14.2-29.0)	-4.53 (-6.42 to -2.47)	-4.91 (-9.21 to -0.64)	-4.70 (-6.54 to -2.79)
Sangli	54.2 (46.2-62.6)	30.1 (25.4-35.2)	20.7 (15.3-27.6)	-5.70 (-7.22 to -4.11)	-5.30 (-9.19 to -1.57)	-5.54 (-7.21 to -3.89)
Satara	45.9 (38.1-54.6)	28.6 (23.4-34.1)	19.9 (14.4-26.5)	-4.64 (-6.24 to -2.94)	-5.14 (-9.15 to -1.29)	-4.85 (-6.43 to -3.19)
Sindhudurg	48.6 (40.5-58.2)	26.9 (21.7-33.4)	20.1 (14.2-28.0)	-5.78 (-7.68 to -3.78)	-4.14 (-8.48 to 0.21)	-5.11 (-7.02 to -3.24)
Solapur	55.2 (47.3-63.2)	32.8 (28.0-38.2)	23.2 (16.9-30.5)	-5.06 (-6.53 to -3.59)	-4.93 (-8.72 to -1.22)	-5.01 (-6.69 to -3.48)
Thane	43.2 (37.2-50.0)	32.2 (26.7-38.5)	23.6 (17.2-31.0)	-2.90 (-4.74 to -0.94)	-4.43 (-8.37 to -0.43)	-3.54 (-5.22 to -1.80)
Wardha	64.1 (56.0-72.9)	40.8 (35.0-47.1)	30.7 (23.1-39.4)	-4.42 (-5.72 to -3.15)	-4.06 (-7.38 to -0.68)	-4.28 (-5.78 to -2.78)
Washim	53.8 (46.6-61.3)	41.5 (35.8-47.6)	28.2 (21.2-36.4)	-2.57 (-3.92 to -1.20)	-5.41 (-8.90 to -1.77)	-3.76 (-5.40 to -2.21)
Yavatmal	58.7 (50.6-67.0)	42.9 (36.6-49.5)	30.6 (23.2-39.5)	-3.07 (-4.41 to -1.74)	-4.78 (-8.13 to -1.33)	-3.78 (-5.31 to -2.32)
**Punjab**	**55.6 (50.5-61.0)**	**41.6 (36.6-47.4)**	**29.7 (25.6-34.6)**	**-2.85 (-3.73 to -2.00)**	**-4.77 (-7.36 to -2.09)**	**-3.65 (-4.76 to -2.55)**
Amritsar	51.9 (46.7-57.4)	37.1 (33.1-41.5)	29.2 (23.7-35.7)	-3.35 (-4.36 to -2.35)	-3.43 (-6.12 to -0.64)	-3.39 (-4.59 to -2.16)
Barnala	66.7 (59.7-73.7)	49.1 (43.4-54.7)	34.4 (27.5-42.6)	-3.02 (-4.05 to -2.02)	-5.01 (-7.80 to -2.20)	-3.85 (-5.12 to -2.60)
Bathinda	58.1 (52.1-64.1)	43.3 (38.5-48.4)	30.7 (24.4-38.1)	-2.91 (-3.91 to -1.91)	-4.84 (-7.64 to -1.87)	-3.71 (-4.96 to -2.48)
Faridkot	62.4 (55.8-69.7)	45.1 (40.1-50.5)	34.1 (27.1-42.5)	-3.19 (-4.22 to -2.12)	-3.98 (-6.88 to -1.09)	-3.52 (-4.77 to -2.27)
Fatehgarh Sahib	53.5 (47.9-59.6)	41.1 (36.1-46.3)	26.4 (20.9-32.3)	-2.64 (-3.66 to -1.59)	-6.19 (-8.81 to -3.35)	-4.12 (-5.32 to -2.94)
Fazilka	55.5 (49.8-61.5)	41.2 (36.9-45.6)	31.3 (25.2-38.7)	-2.96 (-3.94 to -1.96)	-3.97 (-6.80 to -1.03)	-3.38 (-4.61 to -2.13)
Ferozepur	68.5 (61.6-76.5)	50.4 (44.8-56.3)	38.3 (30.5-47.7)	-3.00 (-4.02 to -1.96)	-3.90 (-6.76 to -1.01)	-3.38 (-4.63 to -2.15)
Gurdaspur	47.1 (42.2-52.4)	34.9 (31.1-39.3)	27.1 (21.8-33.2)	-2.99 (-4.02 to -1.89)	-3.63 (-6.32 to -0.92)	-3.25 (-4.48 to -2.02)
Hoshiarpur	47.5 (42.8-53.3)	36.2 (31.6-41.1)	25.8 (20.3-32.0)	-2.70 (-3.81 to -1.57)	-4.72 (-7.46 to -1.95)	-3.54 (-4.78 to -2.35)
Jalandhar	52.7 (47.4-58.7)	39.8 (35.2-44.5)	28.7 (22.8-35.6)	-2.76 (-3.85 to -1.67)	-4.61 (-7.40 to -1.78)	-3.53 (-4.77 to -2.27)
Kapurthala	54.4 (48.8-60.3)	40.0 (35.5-44.8)	30.1 (24.0-37.2)	-3.04 (-4.13 to -2.00)	-4.03 (-6.85 to -1.25)	-3.45 (-4.68 to -2.18)
Ludhiana	55.4 (49.5-61.1)	44.0 (38.9-49.6)	28.6 (22.8-35.3)	-2.27 (-3.28 to -1.22)	-6.03 (-8.67 to -3.16)	-3.84 (-5.05 to -2.65)
Mansa	63.4 (56.3-70.5)	48.2 (42.5-54.3)	33.1 (26.2-40.9)	-2.70 (-3.73 to -1.59)	-5.28 (-8.09 to -2.29)	-3.77 (-5.12 to -2.55)
Moga	65.6 (57.7-74.4)	47.6 (41.6-54.1)	35.7 (28.0-45.3)	-3.16 (-4.39 to -1.99)	-4.06 (-7.10 to -0.95)	-3.54 (-4.88 to -2.19)
Pathankot	42.3 (37.6-47.6)	33.4 (29.1-37.9)	23.4 (18.5-29.1)	-2.26 (-3.44 to -1.11)	-4.95 (-7.76 to -2.17)	-3.38 (-4.61 to -2.14)
Patiala	56.5 (51.0-62.1)	42.6 (37.7-47.9)	27.0 (21.4-33.1)	-2.77 (-3.72 to -1.80)	-6.39 (-9.03 to -3.62)	-4.28 (-5.47 to -3.18)
Rupnagar	48.4 (43.5-54.0)	37.6 (33.1-42.7)	25.2 (20.0-31.1)	-2.45 (-3.50 to -1.26)	-5.69 (-8.43 to -2.84)	-3.80 (-5.03 to -2.57)
Sahibzada Ajit Singh Nagar	49.1 (43.6-55.3)	37.4 (32.4-42.7)	23.9 (18.8-29.8)	-2.67 (-3.80 to -1.46)	-6.28 (-9.04 to -3.37)	-4.17 (-5.44 to -2.98)
Sangrur	65.7 (59.2-71.9)	49.2 (43.7-54.6)	32.8 (26.0-40.5)	-2.85 (-3.77 to -1.93)	-5.68 (-8.37 to -2.91)	-4.03 (-5.23 to -2.83)
Shahid Bhagat Singh Nagar	51.6 (46.4-57.2)	40.2 (35.5-45.2)	27.5 (21.8-33.9)	-2.47 (-3.54 to -1.39)	-5.34 (-8.10 to -2.53)	-3.67 (-4.89 to -2.46)
Sri Mukhtar Sahib	54.9 (49.3-60.7)	40.0 (35.7-44.3)	30.0 (24.1-37.4)	-3.12 (-4.10 to -2.15)	-4.04 (-6.79 to -1.23)	-3.50 (-4.66 to -2.27)
Tarn Taran	61.1 (55.0-67.9)	42.9 (38.4-47.8)	35.2 (28.5-43.2)	-3.56 (-4.58 to -2.53)	-2.82 (-5.65 to 0.08)	-3.26 (-4.51 to -2.06)
**Sikkim**	**47.5 (42.3-52.8)**	**33.4 (28.7-38.4)**	**21.9 (18.8-25.7)**	**-3.47 (-4.72 to -2.20)**	**-5.90 (-8.83 to -2.78)**	**-4.48 (-5.75 to -3.13)**
East Sikkim	43.9 (38.6-50.3)	30.5 (26.4-35.2)	19.6 (15.4-24.8)	-3.54 (-4.80 to -2.27)	-6.05 (-9.03 to -2.89)	-4.59 (-5.89 to -3.20)
North Sikkim	56.1 (48.3-64.3)	39.1 (33.5-45.3)	26.3 (20.3-33.9)	-3.53 (-4.81 to -2.19)	-5.57 (-8.45 to -2.57)	-4.38 (-5.67 to -3.04)
South Sikkim	45.7 (40.5-51.8)	31.9 (27.7-36.5)	20.5 (16.1-25.8)	-3.53 (-4.75 to -2.24)	-6.15 (-9.12 to -3.02)	-4.62 (-5.89 to -3.25)
West Sikkim	51.9 (46.2-58.5)	37.0 (32.1-42.6)	24.6 (19.2-31.3)	-3.32 (-4.59 to -2.02)	-5.73 (-8.65 to -2.52)	-4.32 (-5.63 to -2.95)
**Nagaland**	**60.4 (54.2-66.9)**	**41.8 (36.4-47.8)**	**34.8 (30.0-40.6)**	**-3.62 (-4.65 to -2.50)**	**-2.66 (-5.78 to 0.33)**	**-3.23 (-4.54 to -1.97)**
Dimapur	69.3 (61.0-78.9)	46.0 (40.5-51.9)	36.7 (28.6-45.9)	-4.06 (-5.20 to -2.93)	-3.21 (-6.27 to -0.11)	-3.72 (-5.03 to -2.41)
Kiphire	44.0 (37.4-50.9)	31.4 (26.6-36.6)	30.0 (23.0-38.2)	-3.30 (-4.73 to -1.84)	-0.73 (-4.29 to 2.82)	-2.26 (-3.82 to -0.79)
Kohima	53.4 (47.4-60.0)	35.7 (31.7-40.1)	29.8 (23.3-37.1)	-3.92 (-4.94 to -2.73)	-2.63 (-5.81 to 0.48)	-3.39 (-4.71 to -2.05)
Longleng	63.9 (57.0-71.3)	46.1 (40.9-52.3)	37.9 (29.8-47.0)	-3.19 (-4.39 to -2.01)	-2.72 (-6.06 to 0.44)	-3.00 (-4.40 to -1.65)
Mokokchung	66.9 (59.3-74.7)	45.5 (40.2-51.3)	35.7 (27.7-44.4)	-3.80 (-5.03 to -2.64)	-3.48 (-6.72 to -0.39)	-3.67 (-5.04 to -2.32)
Mon	65.7 (58.3-73.7)	48.6 (43.0-55.0)	40.6 (31.7-51.1)	-2.89 (-4.09 to -1.70)	-2.63 (-5.99 to 0.70)	-2.79 (-4.19 to -1.44)
Peren	68.3 (60.8-76.5)	47.3 (42.0-52.7)	38.4 (30.2-48.2)	-3.64 (-4.70 to -2.55)	-2.98 (-5.95 to 0.10)	-3.37 (-4.70 to -2.11)
Phek	44.0 (38.6-50.0)	30.6 (26.7-34.9)	27.8 (21.4-34.9)	-3.52 (-4.74 to -2.21)	-1.40 (-4.75 to 2.04)	-2.66 (-4.09 to -1.30)
Tuensang	49.6 (43.0-56.8)	36.2 (31.3-41.5)	33.0 (25.6-41.4)	-3.09 (-4.42 to -1.73)	-1.37 (-4.82 to 2.05)	-2.39 (-3.94 to -0.97)
Wokha	67.3 (60.2-75.3)	45.5 (40.2-51.0)	35.8 (28.1-44.8)	-3.84 (-4.95 to -2.65)	-3.42 (-6.60 to -0.35)	-3.67 (-5.03 to -2.35)

Zunheboto	50.9 (44.4-57.8)	35.3 (30.7-40.2)	30.5 (23.5-38.0)	-3.62 (-4.86 to -2.30)	-2.13 (-5.44 to 1.23)	-3.01 (-4.46 to -1.62)
**Himachal Pradesh**	**52.2 (47.1-57.6)**	**40.6 (35.6-46.3)**	**31.4 (27.1-35.6)**	**-2.47 (-3.69 to -1.28)**	**-3.69 (-6.46 to -0.99)**	**-2.98 (-4.28 to -1.78)**
Bilaspur	46.1 (40.8-52.1)	33.9 (29.2-38.8)	26.2 (20.8-32.7)	-3.04 (-4.34 to -1.74)	-3.65 (-6.56 to -0.66)	-3.29 (-4.65 to -1.99)
Chamba	51.8 (44.5-59.7)	43.2 (36.8-50.5)	34.7 (26.4-44.2)	-1.78 (-3.15 to -0.36)	-3.10 (-6.15 to -0.13)	-2.33 (-3.75 to -1.03)
Hamirpur	43.6 (38.1-50.1)	32.4 (27.5-37.8)	24.9 (19.3-31.7)	-2.92 (-4.38 to -1.50)	-3.74 (-6.75 to -0.71)	-3.26 (-4.65 to -1.95)
Kangra	50.2 (43.7-56.9)	39.5 (33.6-45.6)	31.0 (23.8-39.1)	-2.38 (-3.76 to -0.99)	-3.43 (-6.37 to -0.58)	-2.82 (-4.23 to -1.57)
Kinnaur	61.8 (51.9-72.8)	50.1 (42.2-59.5)	37.4 (28.3-48.4)	-2.07 (-3.77 to -0.47)	-4.15 (-7.41 to -0.99)	-2.94 (-4.56 to -1.38)
Kullu	61.0 (52.9-69.7)	49.9 (42.4-57.8)	38.2 (29.4-48.2)	-2.00 (-3.46 to -0.47)	-3.80 (-6.78 to -0.88)	-2.75 (-4.25 to -1.40)
Lahul and Spiti	56.4 (47.5-65.5)	46.1 (38.2-54.0)	36.2 (27.0-46.7)	-2.04 (-3.58 to -0.40)	-3.40 (-6.47 to -0.35)	-2.60 (-4.17 to -1.23)
Mandi	50.9 (45.3-57.3)	39.7 (34.5-45.3)	29.6 (23.4-36.9)	-2.45 (-3.81 to -1.14)	-4.19 (-7.16 to -1.37)	-3.18 (-4.53 to -1.87)
Shimla	62.0 (53.8-70.8)	49.5 (42.4-57.4)	37.4 (29.3-47.0)	-2.24 (-3.59 to -0.94)	-3.97 (-6.93 to -0.96)	-2.96 (-4.35 to -1.55)
Sirmaur	57.4 (51.6-63.9)	45.3 (40.3-50.8)	34.1 (27.4-41.9)	-2.40 (-3.43 to -1.33)	-3.97 (-6.72 to -1.06)	-3.06 (-4.27 to -1.80)
Solan	48.1 (43.0-53.8)	34.4 (30.2-39.2)	26.9 (21.2-33.6)	-3.30 (-4.47 to -2.08)	-3.49 (-6.36 to -0.60)	-3.38 (-4.64 to -2.15)
Una	46.0 (40.5-52.5)	33.1 (28.3-38.8)	26.3 (20.6-33.1)	-3.26 (-4.58 to -2.00)	-3.24 (-6.19 to -0.27)	-3.26 (-4.59 to -1.97)
**UTs other than Delhi**	**35.2 (29.7-41.4)**	**29.7 (24.5-36.1)**	**22.9 (19.7-26.9)**	**-1.69 (-2.52 to -0.84)**	**-3.68 (-5.77 to -1.53)**	**-2.52 (-3.45 to -1.49)**
Chandigarh	37.2 (32.8-42.4)	33.8 (29.0-39.2)	23.4 (18.1-29.7)	-0.50 (-1.73 to 0.78)	-5.06 (-7.93 to -2.04)	-2.41 (-3.77 to -1.12)
Dadra & Nagar Haveli	43.4 (36.6-51.1)	40.3 (33.6-48.3)	30.4 (22.2-40.8)	-0.53 (-2.25 to 1.20)	-3.98 (-7.79 to -0.05)	-1.97 (-3.71 to -0.26)
Daman	37.2 (31.8-42.8)	33.3 (28.0-39.2)	24.0 (17.8-31.8)	-1.12 (-2.74 to 0.51)	-4.63 (-8.38 to -0.70)	-2.59 (-4.26 to -0.95)
Diu	45.2 (36.5-54.9)	35.2 (27.9-43.8)	27.5 (19.7-37.8)	-1.34 (-3.31 to 0.78)	-4.20 (-8.19 to -0.14)	-2.54 (-4.33 to -0.55)
Karaikal	38.3 (31.8-45.7)	25.3 (20.4-31.0)	22.8 (15.6-31.7)	-3.80 (-5.66 to -1.85)	-1.19 (-5.81 to 3.36)	-2.74 (-4.86 to -0.72)
Lakshadweep	17.7 (7.1-37.1)	12.5 (4.9-28.0)	10.8 (4.1-23.7)	-3.45 (-6.84 to -0.25)		
Mahe	18.0 (14.9-21.5)	11.1 (9.0-13.5)	9.5 (6.5-13.3)	-3.86 (-5.82 to -1.75)	-2.46 (-6.82 to 2.14)	-3.29 (-5.28 to -1.39)
Nicobars	18.5 (8.1-37.3)	15.6 (6.7-30.7)	12.6 (4.8-27.1)	-1.64 (-5.92 to 2.96)	-3.21 (-8.79 to 2.90)	-2.31 (-5.96 to 1.43)
North & Middle Andaman	39.7 (29.1-52.3)	36.1 (25.9-49.4)	25.8 (15.3-40.1)	-0.94 (-4.32 to 2.46)	-4.88 (-10.40 to 0.61)	-2.59 (-5.61 to 0.38)
Puducherry	33.4 (28.9-38.2)	22.5 (19.3-26.4)	19.9 (14.6-26.8)	-3.85 (-5.49 to -2.19)	-1.64 (-5.97 to 2.83)	-2.95 (-4.81 to -1.08)
South Andaman	29.1 (20.6-40.8)	24.9 (17.8-35.0)	18.4 (10.8-28.6)	-1.53 (-4.87 to 1.94)	-4.44 (-10.26 to 1.30)	-2.75 (-5.80 to 0.30)
Yanam	51.7 (42.3-63.4)	33.0 (26.7-41.1)	21.9 (15.7-29.8)	-4.32 (-6.08 to -2.49)	-6.09 (-9.57 to -2.28)	-5.06 (-6.73 to -3.28)
**Kerala**	**19.9 (17.5-22.6)**	**12.9 (11.1-15.0)**	**10.4 (9.0-12.2)**	**-4.26 (-5.81 to -2.64)**	**-3.10 (-7.16 to 1.36)**	**-3.79 (-5.71 to -1.92)**
Alappuzha	17.8 (13.8-22.1)	11.9 (9.2-15.2)	9.1 (6.1-13.2)	-3.95 (-6.03 to -1.74)	-3.83 (-8.32 to 1.30)	-3.91 (-6.07 to -1.62)
Ernakulam	16.6 (13.8-19.8)	10.6 (8.6-13.1)	8.4 (5.8-12.1)	-4.43 (-6.33 to -2.49)	-3.30 (-7.62 to 1.52)	-3.97 (-6.01 to -1.84)
Idukki	26.8 (22.7-31.6)	17.6 (14.6-21.0)	14.6 (10.2-20.3)	-4.13 (-5.91 to -2.31)	-2.53 (-6.97 to 2.36)	-3.48 (-5.50 to -1.48)
Kannur	21.9 (18.3-25.9)	13.2 (10.8-15.9)	11.4 (7.9-15.6)	-4.97 (-6.72 to -3.04)	-2.18 (-6.61 to 2.29)	-3.84 (-5.80 to -1.95)
Kasaragod	21.3 (17.1-25.7)	13.0 (10.3-16.2)	10.7 (7.3-15.1)	-4.78 (-6.69 to -2.70)	-2.90 (-7.36 to 1.61)	-4.02 (-6.06 to -2.10)
Kollam	19.2 (15.3-23.2)	13.1 (10.3-16.2)	10.3 (7.0-14.8)	-3.74 (-5.79 to -1.51)	-3.48 (-8.01 to 1.55)	-3.64 (-5.82 to -1.45)
Kottayam	18.6 (15.0-22.3)	12.0 (9.5-14.9)	9.7 (6.6-13.9)	-4.28 (-6.19 to -2.26)	-3.16 (-7.55 to 1.91)	-3.83 (-5.96 to -1.67)
Kozhikode	20.7 (17.0-24.7)	13.1 (10.6-16.1)	11.0 (7.5-15.3)	-4.43 (-6.26 to -2.50)	-2.66 (-7.09 to 2.00)	-3.72 (-5.72 to -1.79)
Malappuram	21.6 (18.3-25.3)	14.0 (11.6-16.9)	11.3 (8.0-15.7)	-4.22 (-5.96 to -2.38)	-3.11 (-7.40 to 1.58)	-3.77 (-5.72 to -1.91)
Palakkad	21.4 (17.6-25.7)	13.6 (11.0-16.7)	11.0 (7.6-15.6)	-4.38 (-6.22 to -2.54)	-3.00 (-7.32 to 1.84)	-3.82 (-5.75 to -1.86)
Pathanamthitta	21.0 (16.9-25.5)	13.7 (10.7-17.0)	11.5 (7.8-16.3)	-4.20 (-6.20 to -2.13)	-2.63 (-7.23 to 2.47)	-3.56 (-5.71 to -1.43)
Thiruvananthapuram	19.4 (15.2-24.2)	13.5 (10.3-17.1)	10.3 (6.6-14.9)	-3.55 (-5.82 to -1.13)	-4.01 (-8.70 to 1.34)	-3.75 (-6.16 to -1.48)
Thrissur	16.4 (13.2-20.2)	10.4 (8.2-13.1)	8.4 (5.6-12.1)	-4.44 (-6.46 to -2.54)	-3.18 (-7.43 to 1.82)	-3.93 (-5.94 to -1.86)
Wayanad	27.4 (22.6-32.6)	17.4 (14.0-21.5)	14.0 (9.5-19.4)	-4.42 (-6.15 to -2.51)	-3.16 (-7.47 to 1.26)	-3.91 (-5.87 to -2.00)
**Delhi**	**57.5 (52.0-63.2)**	**31.8 (26.6-38.3)**	**25.1 (21.7-29.4)**	**-5.75 (-6.58 to -4.94)**	**-3.38 (-6.12 to -0.58)**	**-4.78 (-5.97 to -3.70)**
Central	59.4 (53.5-65.0)	32.9 (29.4-36.5)	26.2 (21.1-32.4)	-5.75 (-6.55 to -4.94)	-3.23 (-5.99 to -0.46)	-4.72 (-5.90 to -3.64)
East	57.9 (52.5-63.1)	31.8 (28.5-35.1)	25.2 (20.3-31.1)	-5.54 (-6.36 to -4.72)	-3.68 (-6.34 to -0.90)	-4.78 (-5.94 to -3.69)
New Delhi	57.8 (52.2-63.0)	33.0 (29.6-36.5)	26.2 (21.2-32.5)	-5.46 (-6.28 to -4.64)	-3.28 (-6.03 to -0.49)	-4.57 (-5.75 to -3.50)
North	58.1 (52.5-63.2)	31.8 (28.5-35.2)	24.7 (20.0-30.6)	-5.75 (-6.58 to -4.96)	-3.31 (-6.03 to -0.52)	-4.76 (-5.93 to -3.67)
North East	58.7 (53.1-64.0)	32.0 (28.8-35.4)	25.2 (20.3-31.2)	-5.85 (-6.68 to -5.03)	-3.44 (-6.17 to -0.64)	-4.87 (-6.02 to -3.76)
North West	57.7 (52.2-62.8)	31.1 (27.9-34.5)	24.4 (19.6-30.1)	-5.99 (-6.84 to -5.18)	-3.49 (-6.21 to -0.68)	-4.97 (-6.16 to -3.87)
Shahdara	57.1 (51.9-62.3)	32.7 (29.4-36.2)	24.7 (20.0-30.7)	-5.79 (-6.63 to -4.96)	-3.69 (-6.39 to -0.91)	-4.93 (-6.07 to -3.83)
South	57.9 (52.6-62.9)	32.6 (29.3-35.9)	25.8 (20.8-31.8)	-5.58 (-6.41 to -4.78)	-3.32 (-6.03 to -0.52)	-4.66 (-5.84 to -3.58)
South East	58.4 (53.0-63.6)	34.5 (31.0-38.0)	27.0 (21.7-33.3)	-5.66 (-6.48 to -4.86)	-3.29 (-5.99 to -0.52)	-4.69 (-5.87 to -3.62)
South West	56.8 (51.7-61.5)	31.8 (28.6-35.1)	25.2 (20.3-31.0)	-5.66 (-6.47 to -4.85)	-3.27 (-6.01 to -0.49)	-4.69 (-5.89 to -3.58)
West	56.0 (50.7-60.8)	30.9 (27.8-34.2)	24.6 (19.7-30.2)	-5.77 (-6.59 to -4.96)	-3.27 (-6.04 to -0.50)	-4.75 (-5.96 to -3.64)
**Goa**	**35.2 (30.8-40.4)**	**24.2 (20.2-28.5)**	**18.8 (13.5-26.6)**	**-3.69 (-5.54 to -1.82)**	**-3.66 (-8.05 to 0.51)**	**-3.69 (-5.58 to -1.76)**
North Goa	35.6 (29.4-42.9)	24.8 (20.2-30.8)	19.4 (13.6-27.0)	-3.55 (-5.48 to -1.59)	-3.60 (-7.94 to 0.63)	-3.58 (-5.54 to -1.59)
South Goa	35.0 (29.0-41.2)	23.7 (19.4-28.9)	18.3 (13.0-25.1)	-3.82 (-5.66 to -1.96)	-3.71 (-8.10 to 0.43)	-3.78 (-5.75 to -1.89)

NMR

States[*]/Districts	NMR (95% uncertainty interval)					
	2000	2010	2017	Annual rate of change, 2000-2010 (%)	Annual rate of change, 2010-2017 (%)	Annual rate of change, 2000-2017 (%)
**Bihar**	**36.5 (31.1-41.9)**	**28.9 (25.2-33.0)**	**23.4 (20.0-27.8)**	**-2.28 (-3.01 to -1.52)**	**-3.04 (-5.77 to -0.09)**	**-2.60 (-3.80 to -1.46)**
Araria	36.6 (32.9-40.8)	29.1 (25.9-32.7)	24.1 (18.8-30.4)	-2.29 (-3.38 to -1.20)	-2.70 (-5.66 to 0.44)	-2.46 (-3.81 to -1.07)
Arwal	35.2 (31.3-39.5)	28.8 (25.4-32.2)	23.7 (18.7-29.8)	-2.00 (-3.07 to -0.85)	-2.81 (-5.98 to 0.38)	-2.34 (-3.69 to -1.00)
Aurangabad	36.6 (32.5-40.9)	30.4 (27.1-34.0)	25.1 (19.9-31.5)	-1.83 (-2.89 to -0.68)	-2.79 (-5.90 to 0.33)	-2.23 (-3.52 to -0.86)
Banka	37.0 (33.2-41.4)	30.4 (27.1-34.2)	24.8 (19.5-31.7)	-1.94 (-3.03 to -0.84)	-2.93 (-6.05 to 0.20)	-2.35 (-3.79 to -1.02)
Begusarai	34.2 (30.9-37.5)	27.5 (24.9-30.6)	22.2 (17.6-27.6)	-2.15 (-3.09 to -1.14)	-3.13 (-6.09 to 0.01)	-2.56 (-3.93 to -1.16)
Bhagalpur	37.0 (33.3-41.1)	29.7 (26.7-33.3)	24.2 (19.1-30.8)	-2.16 (-3.15 to -1.12)	-2.95 (-6.04 to 0.32)	-2.49 (-3.82 to -1.18)
Bhojpur	34.3 (31.0-37.8)	27.8 (25.0-30.8)	22.6 (17.9-28.2)	-2.09 (-3.06 to -1.07)	-2.98 (-6.01 to 0.26)	-2.46 (-3.76 to -1.22)
Buxar	34.9 (31.8-38.3)	28.1 (25.4-31.1)	23.3 (18.5-29.3)	-2.14 (-3.02 to -1.16)	-2.73 (-5.78 to 0.42)	-2.39 (-3.71 to -1.06)
Darbhanga	37.5 (33.9-41.1)	28.7 (25.7-31.9)	22.9 (18.2-28.8)	-2.61 (-3.62 to -1.62)	-3.26 (-6.18 to -0.04)	-2.88 (-4.24 to -1.53)
East Champaran	38.0 (34.2-42.2)	28.9 (25.9-32.4)	23.7 (18.6-30.2)	-2.69 (-3.74 to -1.64)	-2.90 (-6.03 to 0.40)	-2.78 (-4.17 to -1.49)
Gaya	35.8 (32.0-39.6)	30.3 (26.9-33.7)	24.5 (19.6-30.7)	-1.66 (-2.62 to -0.59)	-3.04 (-6.04 to 0.02)	-2.23 (-3.55 to -0.97)
Gopalganj	34.5 (31.2-38.0)	26.7 (24.0-29.9)	21.5 (16.9-27.3)	-2.53 (-3.55 to -1.48)	-3.09 (-6.23 to 0.20)	-2.76 (-4.12 to -1.47)
Jamui	35.3 (32.0-39.0)	29.6 (26.8-33.2)	24.1 (19.3-30.4)	-1.75 (-2.71 to -0.81)	-2.99 (-6.04 to -0.01)	-2.27 (-3.59 to -0.99)
Jehanabad	34.9 (31.3-38.7)	28.6 (25.6-31.9)	23.4 (18.7-29.3)	-1.96 (-2.94 to -0.92)	-2.92 (-5.97 to 0.12)	-2.36 (-3.69 to -1.12)
Kaimur	36.9 (33.3-40.9)	29.5 (26.6-33.0)	25.2 (19.9-31.9)	-2.20 (-3.15 to -1.19)	-2.31 (-5.34 to 0.98)	-2.25 (-3.59 to -0.90)
Katihar	42.2 (37.6-47.1)	34.1 (30.3-38.3)	27.2 (21.2-34.9)	-2.12 (-3.19 to -1.04)	-3.25 (-6.22 to -0.06)	-2.60 (-3.92 to -1.25)
Khagaria	34.3 (30.8-38.1)	27.2 (24.3-30.4)	22.7 (17.9-28.6)	-2.31 (-3.39 to -1.23)	-2.61 (-5.72 to 0.72)	-2.44 (-3.85 to -0.99)
Kishanganj	37.1 (33.0-41.8)	29.7 (26.2-33.7)	24.1 (18.7-30.7)	-2.22 (-3.41 to -1.05)	-2.89 (-5.78 to 0.52)	-2.50 (-3.82 to -1.07)
Lakhisarai	33.7 (29.9-37.5)	27.8 (24.7-31.4)	22.8 (18.1-29.2)	-1.92 (-3.04 to -0.83)	-2.83 (-5.94 to 0.31)	-2.30 (-3.69 to -0.91)
Madhepura	35.9 (32.1-40.0)	28.4 (25.2-31.9)	23.7 (18.6-30.0)	-2.30 (-3.43 to -1.17)	-2.61 (-5.71 to 0.70)	-2.43 (-3.89 to -0.93)
Madhubani	43.6 (38.9-48.4)	32.0 (28.4-35.5)	25.5 (19.9-32.5)	-3.04 (-4.04 to -2.02)	-3.26 (-6.27 to -0.04)	-3.13 (-4.51 to -1.71)
Munger	34.0 (30.9-37.5)	26.9 (24.2-29.9)	21.9 (17.3-27.7)	-2.34 (-3.33 to -1.30)	-2.91 (-6.02 to 0.21)	-2.58 (-3.91 to -1.22)
Muzaffarpur	35.8 (32.2-39.6)	27.9 (24.9-31.4)	22.0 (17.3-28.0)	-2.44 (-3.50 to -1.34)	-3.41 (-6.44 to -0.10)	-2.84 (-4.18 to -1.54)

Nalanda	34.2 (30.8-37.9)	28.6 (25.6-31.8)	22.9 (18.4-28.9)	-1.79 (-2.75 to -0.77)	-3.18 (-5.97 to -0.15)	-2.36 (-3.65 to -1.14)
Nawada	34.7 (31.1-38.4)	29.7 (26.7-32.9)	23.8 (19.0-30.3)	-1.56 (-2.52 to -0.57)	-3.18 (-6.07 to -0.17)	-2.23 (-3.51 to -0.97)
Patna	32.4 (29.3-35.4)	25.9 (23.4-28.6)	20.4 (16.4-25.6)	-2.22 (-3.15 to -1.26)	-3.41 (-6.33 to -0.37)	-2.71 (-4.02 to -1.51)
Purnia	39.4 (35.2-43.7)	32.2 (28.7-36.0)	26.4 (20.7-33.3)	-2.00 (-3.08 to -0.90)	-2.84 (-5.78 to 0.45)	-2.35 (-3.71 to -0.96)
Rohtas	36.3 (32.7-40.0)	29.5 (26.5-32.8)	24.1 (19.1-30.4)	-2.06 (-3.04 to -1.04)	-2.87 (-6.05 to 0.36)	-2.40 (-3.71 to -1.12)
Saharsa	34.5 (30.5-38.8)	27.4 (24.1-30.9)	22.8 (17.6-28.8)	-2.31 (-3.51 to -1.11)	-2.67 (-5.80 to 0.84)	-2.46 (-3.94 to -0.91)
Samastipur	35.1 (32.0-38.3)	28.0 (25.4-31.0)	22.3 (17.7-27.8)	-2.22 (-3.20 to -1.24)	-3.27 (-6.13 to -0.07)	-2.66 (-3.96 to -1.38)
Saran	33.3 (29.7-36.9)	26.5 (23.7-29.8)	21.0 (16.4-26.4)	-2.24 (-3.33 to -1.14)	-3.36 (-6.45 to -0.05)	-2.71 (-4.08 to -1.43)
Sheikhpura	34.2 (30.2-38.1)	29.1 (25.7-32.8)	23.4 (18.4-30.0)	-1.60 (-2.74 to -0.51)	-3.13 (-6.19 to 0.05)	-2.24 (-3.58 to -0.89)
Sheohar	40.7 (36.6-45.2)	31.0 (27.6-34.6)	24.7 (19.4-31.8)	-2.71 (-3.80 to -1.64)	-3.19 (-6.32 to 0.19)	-2.91 (-4.25 to -1.61)
Sitamarhi	45.2 (40.5-50.0)	33.6 (29.8-37.4)	27.2 (21.3-34.7)	-2.93 (-3.97 to -1.88)	-3.04 (-6.04 to 0.36)	-2.98 (-4.38 to -1.63)
Siwan	34.1 (30.9-37.7)	26.8 (24.1-30.0)	21.3 (16.7-26.9)	-2.38 (-3.33 to -1.32)	-3.29 (-6.34 to -0.03)	-2.76 (-4.11 to -1.47)
Supaul	37.7 (33.7-42.1)	28.7 (25.4-32.0)	23.5 (18.5-30.0)	-2.69 (-3.79 to -1.60)	-2.91 (-5.93 to 0.41)	-2.78 (-4.22 to -1.29)
Vaishali	34.4 (31.0-37.6)	27.6 (24.9-30.8)	21.4 (17.0-27.0)	-2.18 (-3.20 to -1.12)	-3.59 (-6.46 to -0.37)	-2.77 (-4.09 to -1.51)
West Champaran	37.1 (33.0-41.7)	27.9 (24.6-31.6)	23.1 (18.0-29.6)	-2.80 (-3.82 to -1.78)	-2.71 (-5.83 to 0.61)	-2.77 (-4.19 to -1.50)
**Madhya Pradesh**	**48.3 (41.4-54.8)**	**36.8 (31.2-42.6)**	**26.9 (23.0-31.7)**	**-2.69 (-3.25 to -2.09)**	**-4.44 (-6.96 to -1.98)**	**-3.42 (-4.55 to -2.39)**
Agar Malwa	50.4 (45.1-56.1)	42.7 (37.7-48.1)	27.0 (21.0-34.2)	-1.65 (-2.66 to -0.60)	-6.42 (-9.44 to -3.33)	-3.65 (-5.05 to -2.25)
Alirajpur	45.8 (40.7-51.2)	40.6 (35.9-45.9)	28.5 (21.8-36.1)	-1.22 (-2.39 to 0.08)	-5.05 (-8.23 to -1.71)	-2.82 (-4.33 to -1.38)
Anuppur	49.6 (44.4-55.4)	38.5 (34.1-43.7)	29.9 (23.1-37.6)	-2.50 (-3.56 to -1.42)	-3.63 (-6.87 to -0.34)	-2.97 (-4.39 to -1.56)
Ashoknagar	52.2 (46.7-57.9)	41.5 (36.8-46.4)	29.9 (23.1-37.4)	-2.31 (-3.28 to -1.36)	-4.64 (-7.63 to -1.78)	-3.28 (-4.64 to -1.96)
Balaghat	44.3 (39.5-49.9)	33.5 (29.8-37.9)	24.3 (18.8-31.2)	-2.77 (-3.94 to -1.67)	-4.56 (-7.79 to -1.23)	-3.52 (-4.95 to -2.04)
Barwani	38.4 (33.5-43.3)	33.6 (29.2-38.1)	23.8 (18.2-30.4)	-1.36 (-2.56 to -0.03)	-4.85 (-8.22 to -1.51)	-2.82 (-4.35 to -1.36)
Betul	37.7 (33.0-43.0)	28.1 (24.4-32.4)	19.7 (14.8-25.5)	-2.89 (-4.13 to -1.63)	-5.04 (-8.49 to -1.74)	-3.79 (-5.32 to -2.31)
Bhind	45.2 (41.0-49.6)	35.9 (32.5-39.5)	27.4 (21.6-34.6)	-2.26 (-3.05 to -1.46)	-3.88 (-6.84 to -0.98)	-2.94 (-4.20 to -1.67)
Bhopal	34.8 (30.2-39.8)	25.6 (22.1-29.5)	18.1 (13.7-23.7)	-3.00 (-4.20 to -1.78)	-4.93 (-8.33 to -1.59)	-3.80 (-5.34 to -2.37)
Burhanpur	39.1 (34.3-44.4)	33.0 (29.1-37.7)	23.5 (17.8-30.4)	-1.70 (-2.96 to -0.47)	-4.77 (-8.32 to -1.28)	-2.98 (-4.46 to -1.52)
Chhatarpur	64.1 (57.5-71.0)	46.1 (41.0-51.0)	35.6 (28.1-44.4)	-3.23 (-4.14 to -2.31)	-3.73 (-6.81 to -0.83)	-3.44 (-4.66 to -2.18)
Chhindwara	38.0 (33.3-42.7)	27.4 (23.9-31.0)	19.9 (15.2-25.7)	-3.22 (-4.34 to -2.06)	-4.54 (-8.03 to -1.44)	-3.77 (-5.14 to -2.36)
Damoh	66.6 (59.9-73.7)	43.4 (38.6-48.7)	36.7 (28.7-46.2)	-4.18 (-5.16 to -3.17)	-2.44 (-5.73 to 0.83)	-3.47 (-4.85 to -2.17)
Datia	48.0 (43.2-52.7)	34.6 (31.1-38.1)	27.2 (21.4-34.2)	-3.19 (-4.09 to -2.28)	-3.46 (-6.53 to -0.40)	-3.30 (-4.61 to -2.03)
Dewas	39.3 (35.2-43.8)	29.4 (26.2-33.3)	20.0 (15.1-25.4)	-2.87 (-3.97 to -1.78)	-5.41 (-8.61 to -2.22)	-3.93 (-5.45 to -2.57)
Dhar	39.8 (35.0-44.2)	31.7 (27.8-35.8)	22.3 (17.2-28.1)	-2.26 (-3.48 to -1.08)	-4.97 (-8.29 to -1.74)	-3.39 (-4.88 to -1.96)
Dindori	57.5 (51.7-63.3)	43.4 (38.7-48.6)	34.4 (26.5-43.7)	-2.77 (-3.81 to -1.76)	-3.35 (-6.49 to 0.01)	-3.01 (-4.34 to -1.56)
Guna	51.4 (45.9-57.4)	43.4 (38.5-49.0)	29.2 (22.5-37.1)	-1.67 (-2.72 to -0.58)	-5.60 (-8.59 to -2.59)	-3.31 (-4.70 to -1.90)
Gwalior	44.8 (40.5-49.4)	34.9 (31.6-38.4)	27.3 (21.5-34.3)	-2.47 (-3.36 to -1.53)	-3.51 (-6.61 to -0.53)	-2.90 (-4.21 to -1.63)
Harda	45.7 (39.8-51.9)	34.0 (29.5-39.0)	24.1 (17.9-31.5)	-2.92 (-4.23 to -1.61)	-4.86 (-8.39 to -1.25)	-3.73 (-5.41 to -2.23)
Hoshangabad	40.7 (36.4-45.3)	29.0 (25.7-32.6)	20.5 (15.5-26.0)	-3.33 (-4.44 to -2.20)	-4.94 (-8.37 to -1.75)	-4.00 (-5.49 to -2.68)
Indore	33.7 (29.9-37.6)	23.9 (21.1-27.1)	16.5 (12.6-20.9)	-3.39 (-4.52 to -2.27)	-5.19 (-8.45 to -2.01)	-4.14 (-5.63 to -2.73)
Jabalpur	56.4 (50.2-63.2)	36.5 (32.2-41.5)	29.1 (22.3-36.9)	-4.26 (-5.40 to -3.20)	-3.27 (-6.70 to 0.06)	-3.86 (-5.22 to -2.45)
Jhabua	46.5 (41.2-52.4)	39.6 (34.9-44.7)	25.9 (19.7-32.7)	-1.59 (-2.81 to -0.44)	-5.97 (-9.22 to -2.72)	-3.43 (-4.93 to -2.05)
Katni	65.9 (59.5-72.8)	45.1 (40.5-50.6)	37.0 (28.8-46.2)	-3.73 (-4.65 to -2.76)	-2.87 (-6.11 to 0.33)	-3.38 (-4.77 to -2.03)
Khandwa	45.2 (40.3-50.6)	35.2 (31.2-39.6)	25.4 (19.0-32.4)	-2.48 (-3.64 to -1.20)	-4.64 (-8.17 to -1.08)	-3.38 (-4.95 to -1.96)
Khargone	41.9 (37.0-46.6)	33.6 (29.5-37.8)	24.1 (18.3-30.6)	-2.21 (-3.34 to -1.02)	-4.71 (-8.13 to -1.31)	-3.25 (-4.81 to -1.90)
Mandla	54.0 (48.5-60.0)	38.8 (34.7-43.7)	30.0 (23.0-37.8)	-3.30 (-4.37 to -2.29)	-3.69 (-6.95 to -0.28)	-3.46 (-4.88 to -2.05)
Mandsaur	48.4 (43.5-53.6)	42.5 (37.7-47.3)	25.1 (19.8-31.6)	-1.29 (-2.28 to -0.21)	-7.36 (-10.27 to -4.49)	-3.84 (-5.14 to -2.51)
Morena	46.2 (41.4-51.2)	40.2 (36.3-44.7)	30.0 (23.5-37.6)	-1.35 (-2.28 to -0.33)	-4.18 (-7.15 to -1.27)	-2.53 (-3.83 to -1.27)
Narsinghpur	47.6 (42.5-52.7)	32.6 (28.8-36.7)	24.5 (18.9-31.1)	-3.69 (-4.76 to -2.64)	-4.12 (-7.52 to -0.91)	-3.87 (-5.31 to -2.50)
Neemuch	49.8 (45.0-55.1)	44.9 (40.4-49.8)	25.4 (20.3-31.9)	-0.99 (-1.94 to -0.03)	-8.05 (-10.74 to -5.34)	-3.96 (-5.18 to -2.71)
Niwari	53.5 (47.7-59.5)	37.2 (33.2-41.6)	28.6 (22.1-35.9)	-3.54 (-4.56 to -2.56)	-3.91 (-7.04 to -0.77)	-3.70 (-5.08 to -2.36)
Panna	70.1 (62.2-78.2)	49.8 (44.3-56.2)	39.4 (30.9-49.4)	-3.36 (-4.31 to -2.34)	-3.40 (-6.62 to -0.18)	-3.38 (-4.71 to -2.08)
Raisen	44.4 (39.6-49.4)	32.4 (28.6-36.4)	23.8 (18.0-30.2)	-3.10 (-4.16 to -2.00)	-4.38 (-7.77 to -1.20)	-3.63 (-5.18 to -2.31)
Rajgarh	49.2 (44.2-54.2)	41.7 (37.2-46.7)	27.0 (21.1-34.2)	-1.66 (-2.67 to -0.65)	-6.08 (-9.08 to -2.91)	-3.51 (-4.89 to -2.15)
Ratlam	41.2 (36.7-45.7)	33.7 (29.9-37.6)	21.0 (16.5-26.6)	-1.98 (-3.02 to -0.93)	-6.66 (-9.82 to -3.61)	-3.94 (-5.32 to -2.61)
Rewa	58.7 (52.8-64.5)	44.5 (39.9-49.4)	33.1 (25.7-41.6)	-2.74 (-3.75 to -1.79)	-4.23 (-7.58 to -1.22)	-3.36 (-4.69 to -2.02)
Sagar	58.5 (52.3-65.4)	41.7 (36.6-46.9)	31.9 (24.4-40.3)	-3.33 (-4.33 to -2.29)	-3.83 (-6.99 to -0.72)	-3.54 (-4.95 to -2.24)
Satna	62.6 (56.5-68.9)	45.0 (40.2-50.4)	34.1 (26.6-42.7)	-3.24 (-4.21 to -2.25)	-3.99 (-7.20 to -0.96)	-3.55 (-4.93 to -2.24)
Sehore	39.8 (35.5-44.3)	30.0 (26.4-33.7)	20.8 (15.8-26.4)	-2.79 (-3.91 to -1.74)	-5.17 (-8.54 to -1.91)	-3.78 (-5.25 to -2.40)
Seoni	43.7 (38.7-49.0)	30.7 (27.1-34.7)	23.0 (17.8-29.6)	-3.47 (-4.57 to -2.39)	-4.11 (-7.45 to -0.87)	-3.74 (-5.17 to -2.30)
Shahdol	56.9 (50.9-62.8)	43.1 (38.3-48.5)	34.8 (26.9-44.0)	-2.73 (-3.72 to -1.71)	-3.10 (-6.28 to 0.15)	-2.89 (-4.32 to -1.54)
Shajapur	40.1 (35.8-44.3)	32.2 (28.6-36.2)	21.4 (16.6-27.1)	-2.16 (-3.20 to -1.17)	-5.74 (-8.83 to -2.55)	-3.66 (-5.08 to -2.35)
Sheopur	59.2 (53.5-65.0)	53.3 (47.7-59.0)	37.4 (29.5-46.5)	-1.03 (-1.93 to -0.15)	-5.06 (-7.90 to -2.31)	-2.71 (-3.91 to -1.45)
Shivpuri	53.1 (47.8-58.6)	40.9 (36.5-45.4)	30.6 (24.1-38.1)	-2.58 (-3.49 to -1.65)	-4.13 (-7.08 to -1.20)	-3.23 (-4.51 to -1.94)
Sidhi	57.8 (51.7-64.7)	44.8 (39.8-50.5)	35.1 (27.2-44.3)	-2.51 (-3.57 to -1.42)	-3.52 (-6.79 to -0.39)	-2.93 (-4.36 to -1.56)
Singrauli	54.8 (48.2-62.0)	43.9 (38.5-50.1)	33.6 (25.9-42.6)	-2.19 (-3.22 to -1.09)	-3.85 (-7.03 to -0.67)	-2.88 (-4.29 to -1.55)
Tikamgarh	61.0 (54.9-67.3)	44.4 (39.8-49.2)	34.2 (26.9-42.3)	-3.12 (-4.04 to -2.22)	-3.77 (-6.68 to -1.00)	-3.39 (-4.70 to -2.16)
Ujjain	36.0 (31.9-40.6)	27.7 (24.3-31.5)	18.5 (14.2-23.5)	-2.56 (-3.73 to -1.42)	-5.75 (-8.97 to -2.63)	-3.89 (-5.37 to -2.47)
Umaria	64.1 (57.3-71.0)	46.6 (41.4-52.9)	37.9 (29.0-48.4)	-3.15 (-4.13 to -2.12)	-2.97 (-6.18 to 0.37)	-3.08 (-4.50 to -1.67)
Vidisha	50.5 (44.3-57.1)	40.8 (35.2-47.0)	29.5 (22.4-37.8)	-2.11 (-3.21 to -1.03)	-4.59 (-7.68 to -1.44)	-3.14 (-4.62 to -1.82)
**Jharkhand**	**37.5 (32.4-43.0)**	**28.7 (23.6-33.8)**	**22.9 (19.8-26.7)**	**-2.64 (-3.37 to -1.89)**	**-3.26 (-5.94 to -0.37)**	**-2.90 (-3.98 to -1.76)**
Bokaro	36.2 (32.5-40.0)	28.0 (25.2-30.9)	20.8 (16.7-26.1)	-2.57 (-3.49 to -1.62)	-4.22 (-7.11 to -1.12)	-3.25 (-4.48 to -1.97)
Chatra	37.6 (33.7-41.2)	29.0 (26.1-32.2)	24.8 (20.1-31.0)	-2.59 (-3.49 to -1.64)	-2.18 (-4.92 to 0.78)	-2.42 (-3.60 to -1.21)
Deoghar	37.2 (33.6-41.0)	28.6 (25.7-31.9)	23.4 (18.6-29.4)	-2.59 (-3.54 to -1.59)	-2.91 (-6.10 to 0.09)	-2.72 (-3.97 to -1.49)
Dhanbad	37.1 (33.2-41.0)	28.6 (25.6-31.6)	21.9 (17.3-27.4)	-2.58 (-3.57 to -1.57)	-3.79 (-6.74 to -0.83)	-3.09 (-4.35 to -1.81)
Dumka	39.0 (35.2-43.2)	28.1 (25.1-31.2)	23.8 (19.1-29.8)	-3.26 (-4.27 to -2.24)	-2.37 (-5.43 to 0.70)	-2.90 (-4.20 to -1.64)
East Singhbhum	29.3 (25.7-33.1)	22.1 (19.3-25.1)	16.4 (12.7-21.0)	-2.78 (-4.01 to -1.52)	-4.24 (-7.40 to -0.85)	-3.39 (-4.79 to -1.98)
Garhwa	38.4 (34.3-43.3)	29.7 (26.3-33.6)	26.0 (20.5-33.0)	-2.53 (-3.65 to -1.41)	-1.97 (-5.07 to 1.16)	-2.31 (-3.55 to -0.94)
Giridih	35.6 (32.0-39.4)	28.1 (25.3-31.4)	22.5 (18.0-28.2)	-2.36 (-3.31 to -1.40)	-3.13 (-6.10 to -0.08)	-2.68 (-3.93 to -1.46)
Godda	38.4 (34.6-43.0)	28.7 (25.6-32.4)	24.5 (19.3-31.0)	-2.87 (-3.97 to -1.81)	-2.29 (-5.40 to 0.93)	-2.64 (-4.02 to -1.31)
Gumla	44.9 (40.3-49.5)	35.0 (31.4-38.8)	27.1 (21.7-33.6)	-2.47 (-3.47 to -1.48)	-3.58 (-6.49 to -0.53)	-2.93 (-4.15 to -1.60)
Hazaribagh	35.9 (32.3-39.6)	27.0 (24.4-30.0)	21.6 (17.2-26.9)	-2.81 (-3.77 to -1.83)	-3.18 (-5.93 to -0.17)	-2.97 (-4.19 to -1.75)
Jamtara	40.0 (35.4-44.8)	30.4 (26.7-34.2)	25.0 (19.4-31.4)	-2.70 (-3.80 to -1.60)	-2.80 (-5.96 to 0.10)	-2.75 (-4.04 to -1.43)
Khunti	42.7 (37.8-48.0)	33.5 (29.8-37.4)	24.6 (19.4-31.0)	-2.41 (-3.46 to -1.35)	-4.35 (-7.27 to -1.27)	-3.22 (-4.49 to -1.90)
Koderma	33.4 (30.1-36.8)	25.5 (22.9-28.3)	21.4 (17.2-26.8)	-2.64 (-3.58 to -1.69)	-2.53 (-5.53 to 0.50)	-2.60 (-3.81 to -1.40)
Latehar	41.2 (36.9-45.4)	32.9 (29.4-36.7)	27.0 (21.6-33.6)	-2.20 (-3.19 to -1.23)	-2.84 (-5.62 to 0.31)	-2.47 (-3.70 to -1.21)
Lohardaga	43.8 (38.7-49.1)	34.4 (30.4-38.5)	26.5 (20.9-33.6)	-2.37 (-3.50 to -1.24)	-3.74 (-6.65 to -0.41)	-2.94 (-4.25 to -1.61)
Pakur	38.9 (34.8-42.9)	27.5 (24.6-30.9)	23.8 (19.0-29.9)	-3.40 (-4.36 to -2.38)	-2.12 (-5.08 to 1.05)	-2.88 (-4.10 to -1.60)
Palamu	38.7 (34.8-42.8)	30.3 (27.2-33.7)	25.9 (20.8-32.6)	-2.43 (-3.47 to -1.41)	-2.28 (-5.18 to 0.76)	-2.37 (-3.59 to -1.05)
Ramgarh	36.1 (32.4-39.9)	27.1 (24.5-30.1)	19.8 (15.7-24.7)	-2.83 (-3.78 to -1.86)	-4.46 (-7.27 to -1.28)	-3.51 (-4.72 to -2.21)

Ranchi	39.0 (34.6-43.6)	29.7 (26.6-33.0)	21.7 (17.3-27.2)	-2.69 (-3.67 to -1.70)	-4.40 (-7.26 to -1.29)	-3.41 (-4.62 to -2.10)
Sahibganj	39.7 (35.8-43.6)	29.0 (26.1-32.4)	24.5 (19.4-30.9)	-3.11 (-4.06 to -2.13)	-2.44 (-5.34 to 0.66)	-2.84 (-4.15 to -1.58)
Saraikela Kharsawan	35.0 (31.1-39.4)	27.0 (23.8-30.2)	19.8 (15.4-25.3)	-2.59 (-3.66 to -1.43)	-4.41 (-7.51 to -1.09)	-3.35 (-4.66 to -1.99)
Simdega	44.8 (40.0-49.7)	33.5 (29.9-37.4)	26.6 (21.3-33.3)	-2.86 (-3.90 to -1.80)	-3.28 (-6.34 to -0.19)	-3.04 (-4.28 to -1.73)
West Singhbhum	41.0 (36.8-45.6)	32.9 (29.5-36.5)	25.3 (20.1-31.7)	-2.19 (-3.26 to -1.17)	-3.71 (-6.77 to -0.55)	-2.82 (-4.12 to -1.49)
**Uttar Pradesh**	**48.2 (41.1-55.8)**	**39.4 (34.4-44.5)**	**31.7 (27.3-37.2)**	**-1.99 (-2.49 to -1.50)**	**-3.10 (-5.46 to -0.66)**	**-2.45 (-3.40 to -1.42)**
Agra	43.9 (40.1-48.0)	38.5 (34.7-42.1)	30.3 (24.3-37.2)	-1.29 (-2.14 to -0.44)	-3.44 (-6.40 to -0.54)	-2.18 (-3.37 to -1.01)
Aligarh	47.2 (43.0-51.5)	43.0 (39.0-47.1)	34.8 (28.0-42.7)	-0.91 (-1.77 to -0.10)	-3.03 (-5.72 to -0.20)	-1.79 (-2.94 to -0.68)
Allahabad	56.2 (50.6-61.6)	42.9 (38.2-47.5)	35.2 (27.4-44.9)	-2.66 (-3.63 to -1.64)	-2.83 (-6.21 to 0.55)	-2.73 (-4.12 to -1.29)
Ambedkar Nagar	42.1 (37.8-46.5)	30.6 (27.3-34.3)	24.9 (19.7-31.2)	-3.16 (-4.15 to -2.19)	-2.91 (-6.02 to 0.34)	-3.06 (-4.34 to -1.71)
Amethi	50.3 (44.9-56.5)	38.2 (33.8-43.0)	32.3 (25.6-41.5)	-2.72 (-3.66 to -1.78)	-2.43 (-5.46 to 0.68)	-2.61 (-3.87 to -1.31)
Amroha	45.4 (40.5-50.4)	42.5 (37.6-47.2)	32.9 (26.4-40.8)	-0.66 (-1.63 to 0.38)	-3.60 (-6.38 to -0.66)	-1.89 (-3.06 to -0.73)
Auraiya	46.7 (42.4-51.1)	36.4 (33.0-40.4)	29.3 (23.4-36.7)	-2.45 (-3.36 to -1.60)	-3.11 (-5.93 to -0.21)	-2.72 (-3.95 to -1.51)
Azamgarh	42.8 (38.7-47.0)	33.4 (30.2-36.9)	25.4 (19.9-31.6)	-2.47 (-3.37 to -1.56)	-3.91 (-6.89 to -0.79)	-3.07 (-4.37 to -1.77)
Baghpat	37.8 (33.6-42.1)	31.7 (28.3-35.5)	25.5 (20.4-31.8)	-1.75 (-2.75 to -0.75)	-3.10 (-5.91 to -0.25)	-2.31 (-3.54 to -1.13)
Bahraich	59.1 (54.1-65.2)	49.0 (44.5-54.2)	42.4 (34.5-53.1)	-1.89 (-2.79 to -1.02)	-2.09 (-4.97 to 1.02)	-1.97 (-3.18 to -0.71)
Ballia	39.3 (35.6-43.1)	31.7 (28.7-35.0)	23.5(18.5-29.5)	-2.14 (-2.99 to -1.20)	-4.24 (-7.30 to -1.02)	-3.01 (-4.31 to -1.72)
Balrampur	60.2 (54.6-66.9)	46.8 (42.1-51.8)	40.2 (32.0-51.1)	-2.46 (-3.47 to -1.48)	-2.21 (-5.27 to 1.20)	-2.36 (-3.60 to -1.01)
Banda	54.1 (48.8-59.5)	40.6 (36.3-45.2)	33.8 (26.4-42.2)	-2.84 (-3.77 to -1.93)	-2.61 (-5.79 to 0.46)	-2.75 (-4.03 to -1.46)
Barabanki	51.1 (46.3-56.6)	41.7 (37.5-46.0)	34.9 (28.1-44.2)	-2.05 (-2.95 to -1.21)	-2.56 (-5.40 to 0.48)	-2.26 (-3.49 to -0.92)
Bareilly	51.7 (46.6-56.8)	47.9 (43.3-52.8)	37.3 (30.1-45.9)	-0.75 (-1.63 to 0.19)	-3.56 (-6.34 to -0.81)	-1.92 (-3.08 to -0.72)
Basti	48.5 (44.5-53.0)	35.1 (31.7-38.6)	29.8 (23.7-37.7)	-3.19 (-4.08 to -2.30)	-2.38 (-5.46 to 0.88)	-2.86 (-4.11 to -1.53)
Bhadohi	53.1 (48.1-58.3)	41.3 (37.1-45.5)	32.9 (26.0-41.3)	-2.50 (-3.42 to -1.54)	-3.26 (-6.44 to 0.02)	-2.82 (-4.07 to -1.46)
Bijnor	40.8 (36.9-45.1)	37.2 (33.3-41.4)	28.7 (23.3-35.4)	-0.93 (-1.90 to 0.02)	-3.67 (-6.34 to -0.86)	-2.07 (-3.21 to -0.95)
Budaun	57.3 (51.8-62.8)	57.0 (51.5-62.2)	46.2 (37.5-56.6)	-0.03 (-0.83 to 0.85)	-2.99 (-5.81 to -0.15)	-1.27 (-2.43 to -0.09)
Bulandshahr	45.9 (41.6-50.1)	41.7 (37.5-45.7)	33.2 (26.8-40.9)	-0.97 (-1.83 to -0.10)	-3.24 (-5.98 to -0.44)	-1.91 (-3.02 to -0.79)
Chandauli	45.5 (41.3-50.2)	36.4 (32.9-40.3)	28.2 (22.3-35.4)	-2.22 (-3.14 to -1.31)	-3.58 (-6.62 to -0.24)	-2.79 (-4.09 to -1.47)
Chitrakoot	57.2 (51.6-63.2)	43.3 (38.5-48.3)	36.5 (28.5-46.3)	-2.74 (-3.67 to -1.77)	-2.45 (-5.66 to 0.62)	-2.62 (-3.96 to -1.32)
Deoria	40.7 (37.1-44.5)	30.9 (27.9-34.2)	22.8 (17.9-28.7)	-2.72 (-3.66 to -1.77)	-4.30 (-7.45 to -1.12)	-3.38 (-4.78 to -2.07)
Etah	54.5 (49.4-60.1)	48.7 (43.8-53.5)	39.6 (32.0-49.1)	-1.12 (-2.06 to -0.22)	-2.96 (-5.96 to -0.04)	-1.88 (-3.16 to -0.57)
Etawah	49.3 (45.0-54.0)	39.5 (35.6-43.4)	31.3 (25.1-39.1)	-2.18 (-3.02 to -1.34)	-3.36 (-6.31 to -0.44)	-2.67 (-3.88 to -1.46)
Faizabad	48.9 (44.5-53.9)	36.3 (32.7-40.4)	30.5 (24.2-38.6)	-3.05 (-3.97 to -2.14)	-2.53 (-5.59 to 0.66)	-2.84 (-4.14 to -1.49)
Farrukhabad	54.1 (49.1-59.1)	48.5 (43.7-53.3)	39.4 (32.3-48.9)	-1.08 (-1.91 to -0.25)	-2.97 (-5.71 to -0.09)	-1.87 (-2.99 to -0.70)
Fatehpur	50.5 (45.7-55.8)	38.5 (34.6-43.2)	31.3 (24.8-39.3)	-2.69 (-3.67 to -1.77)	-2.96 (-6.01 to 0.10)	-2.81 (-4.04 to -1.45)
Firozabad	51.6 (46.8-56.9)	44.9 (40.3-49.4)	36.1 (28.8-45.0)	-1.39 (-2.36 to -0.48)	-3.10 (-6.20 to -0.14)	-2.10 (-3.39 to -0.75)
Gautam Buddha Nagar	34.5 (31.3-37.7)	29.8 (26.7-32.6)	23.4 (18.8-29.0)	-1.39 (-2.29 to -0.52)	-3.35 (-6.15 to -0.47)	-2.21 (-3.44 to -1.09)
Ghaziabad	34.2 (30.7-37.6)	29.2 (26.0-32.5)	22.9 (18.4-28.6)	-1.63 (-2.59 to -0.72)	-3.44 (-6.22 to -0.59)	-2.39 (-3.59 to -1.24)
Ghazipur	43.5 (39.0-48.2)	35.2 (31.7-39.2)	26.8 (21.0-33.8)	-2.08 (-3.03 to -1.03)	-3.91 (-7.12 to -0.65)	-2.84 (-4.20 to -1.48)
Gonda	56.0 (51.0-62.2)	44.0 (39.9-48.6)	37.9 (30.2-48.0)	-2.35 (-3.31 to -1.40)	-2.17 (-5.23 to 1.19)	-2.28 (-3.53 to -0.93)
Gorakhpur	42.3 (38.4-46.7)	31.9 (28.6-35.5)	24.2 (18.7-30.9)	-2.80 (-3.81 to -1.80)	-3.96 (-7.08 to -0.65)	-3.28 (-4.66 to -1.91)
Hamirpur	48.1 (43.4-53.0)	34.1 (30.5-37.9)	28.5 (22.4-35.5)	-3.37 (-4.34 to -2.43)	-2.62 (-5.65 to 0.42)	-3.06 (-4.28 to -1.79)
Hapur	43.2 (39.1-47.1)	38.1 (34.0-41.8)	29.7 (23.9-36.8)	-1.25 (-2.14 to -0.36)	-3.58 (-6.25 to -0.80)	-2.22 (-3.37 to -1.09)
Hardoi	54.3 (49.4-59.4)	47.5 (42.9-52.3)	39.2 (32.2-48.9)	-1.31 (-2.19 to -0.47)	-2.77 (-5.48 to 0.07)	-1.92 (-3.07 to -0.70)
Hathras	49.6 (45.1-54.3)	42.9 (38.7-47.0)	34.9 (28.0-42.8)	-1.43 (-2.35 to -0.61)	-3.00 (-5.88 to -0.17)	-2.08 (-3.25 to -0.88)
Jalaun	43.6 (38.9-48.6)	31.2 (27.8-35.1)	26.8 (20.8-34.1)	-3.27 (-4.31 to -2.32)	-2.21 (-5.26 to 0.84)	-2.84 (-4.14 to -1.51)
Jaunpur	48.4 (44.0-52.9)	37.6 (34.0-41.4)	29.8 (23.7-37.1)	-2.50 (-3.32 to -1.64)	-3.35 (-6.32 to -0.23)	-2.86 (-4.04 to -1.52)
Jhansi	48.4 (43.7-53.5)	33.4 (30.0-37.1)	29.7 (23.2-37.3)	-3.66 (-4.63 to -2.78)	-1.66 (-4.74 to 1.50)	-2.85 (-4.18 to -1.57)
Kannauj	50.2 (45.2-55.5)	42.0 (37.5-47.0)	33.9 (27.3-42.7)	-1.75 (-2.73 to -0.81)	-3.10 (-5.95 to -0.14)	-2.31 (-3.52 to -1.03)
Kanpur Dehat	45.5 (41.3-50.0)	33.4 (30.3-37.0)	27.2 (21.8-34.0)	-3.01 (-3.97 to -2.17)	-2.97 (-5.79 to 0.03)	-3.00 (-4.16 to -1.71)
Kanpur Nagar	42.6 (38.3-47.0)	31.4 (28.4-35.2)	24.4 (19.4-30.5)	-2.99 (-3.99 to -2.03)	-3.60 (-6.42 to -0.65)	-3.25 (-4.47 to -1.89)
Kasganj	55.2 (50.3-60.6)	53.2 (48.0-58.2)	43.5 (35.1-54.1)	-0.36 (-1.26 to 0.51)	-2.89 (-5.78 to -0.08)	-1.41 (-2.64 to -0.16)
Kaushambi	56.3 (51.2-61.5)	44.4 (39.8-48.9)	36.9 (29.1-46.5)	-2.45 (-3.40 to -1.51)	-2.93 (-6.13 to 0.22)	-2.66 (-3.92 to -1.29)
Khushinagar	43.0 (39.0-47.6)	33.3 (29.9-37.4)	25.1 (19.5-31.6)	-2.52 (-3.57 to -1.43)	-4.06 (-7.23 to -0.70)	-3.16 (-4.50 to -1.79)
Lakhimpur Kheri	60.8 (54.6-67.1)	47.1 (42.3-52.7)	38.9 (31.4-49.1)	-2.53 (-3.49 to -1.55)	-2.77 (-5.65 to 0.03)	-2.63 (-3.83 to -1.38)
Lalitpur	54.5 (49.0-60.3)	39.2 (35.0-43.4)	34.9 (27.1-43.5)	-3.26 (-4.16 to -2.38)	-1.64 (-4.84 to 1.16)	-2.60 (-3.94 to -1.34)
Lucknow	43.8 (39.4-48.5)	33.4 (29.7-37.4)	26.5 (21.0-33.4)	-2.66 (-3.65 to -1.72)	-3.30 (-6.21 to -0.35)	-2.93 (-4.18 to -1.64)
Maharajganj	47.8 (43.2-53.2)	35.4 (31.7-39.7)	28.0 (21.8-35.6)	-2.97 (-4.03 to -1.86)	-3.32 (-6.47 to 0.18)	-3.12 (-4.47 to -1.74)
Mahoba	54.0 (48.6-59.7)	37.8 (33.8-41.9)	32.5 (25.6-40.7)	-3.55 (-4.48 to -2.62)	-2.12 (-5.21 to 0.86)	-2.97 (-4.18 to -1.69)
Mainpuri	52.7 (47.8-58.0)	45.3 (40.9-49.8)	35.8 (28.9-44.5)	-1.51 (-2.44 to -0.61)	-3.34 (-6.38 to -0.43)	-2.27 (-3.50 to -0.99)
Mathura	43.1 (39.1-47.6)	38.5 (34.6-42.2)	31.0 (24.7-38.2)	-1.14 (-2.10 to -0.24)	-3.07 (-5.98 to -0.27)	-1.95 (-3.13 to -0.76)
Mau	41.9 (38.0-45.9)	32.7 (29.6-36.2)	23.9 (18.8-30.1)	-2.42 (-3.32 to -1.53)	-4.46 (-7.59 to -1.35)	-3.27 (-4.54 to -1.93)
Meerut	40.3 (36.3-44.4)	33.9 (30.3-37.4)	27.1 (21.8-33.4)	-1.72 (-2.63 to -0.79)	-3.23 (-5.93 to -0.41)	-2.34 (-3.50 to -1.22)
Mirzapur	51.6 (47.0-56.7)	41.0 (37.2-45.0)	33.8 (26.8-42.4)	-2.28 (-3.18 to -1.36)	-2.79 (-5.86 to 0.41)	-2.50 (-3.79 to -1.14)
Moradabad	46.3 (41.5-51.4)	45.2 (40.3-50.2)	34.1 (27.4-42.0)	-0.23 (-1.22 to 0.88)	-4.02 (-6.76 to -1.10)	-1.81 (-3.00 to -0.67)
Muzaffarnagar	41.2 (37.4-45.4)	35.3 (31.4-39.4)	28.1 (22.8-34.4)	-1.53 (-2.47 to -0.61)	-3.27 (-6.02 to -0.48)	-2.26 (-3.37 to -1.16)
Pilibhit	49.8 (44.8-54.8)	41.1 (37.0-45.5)	32.0 (26.2-39.6)	-1.79 (-2.69 to -0.85)	-3.58 (-6.43 to -0.91)	-2.54 (-3.70 to -1.40)
Pratapgarh	52.6 (47.7-57.8)	39.6 (35.6-43.6)	32.5 (25.7-41.0)	-2.81 (-3.64 to -1.99)	-2.83 (-5.88 to 0.35)	-2.82 (-4.05 to -1.57)
Rae Bareli	48.3 (43.7-53.2)	36.7 (33.0-40.8)	30.5 (24.3-38.6)	-2.71 (-3.67 to -1.84)	-2.75 (-5.69 to 0.23)	-2.73 (-3.95 to -1.48)
Rampur	45.5 (41.0-50.0)	43.2 (38.8-47.7)	32.8 (26.7-40.5)	-0.50 (-1.41 to 0.52)	-3.92 (-6.68 to -1.15)	-1.92 (-3.15 to -0.79)
Saharanpur	38.3 (34.1-42.8)	33.9 (29.8-38.2)	26.9 (21.3-33.7)	-1.18 (-2.31 to -0.03)	-3.31 (-6.16 to -0.28)	-2.07 (-3.30 to -0.80)
Sambhal	51.6 (46.3-57.1)	51.6 (46.0-57.1)	41.0 (33.0-50.6)	-0.01 (-0.94 to 0.97)	-3.32 (-6.04 to -0.42)	-1.39 (-2.58 to -0.27)
Sant Kabir Nagar	45.6 (41.4-49.9)	33.7 (30.4-37.3)	27.3 (21.5-34.4)	-2.97 (-3.88 to -2.01)	-3.09 (-6.26 to 0.24)	-3.02 (-4.35 to -1.67)
Shahjahanpur	58.8 (53.1-64.6)	51.6 (46.6-56.8)	41.3 (33.9-50.9)	-1.32 (-2.19 to -0.43)	-3.20 (-6.00 to -0.46)	-2.10 (-3.26 to -0.94)
Shamli	40.2 (36.3-44.3)	34.5 (30.7-38.6)	27.4 (22.0-33.8)	-1.51 (-2.47 to -0.55)	-3.33 (-6.07 to -0.51)	-2.27 (-3.44 to -1.15)
Shravasti	60.4 (54.7-66.9)	49.7 (44.7-54.7)	43.1 (34.5-54.4)	-1.80 (-2.86 to -0.81)	-2.10 (-5.05 to 1.18)	-1.92 (-3.17 to -0.51)
Siddharth Nagar	55.0 (50.6-60.6)	40.1 (36.2-44.2)	33.6 (26.8-42.6)	-3.10 (-4.05 to -2.15)	-2.49 (-5.57 to 0.88)	-2.85 (-4.13 to -1.55)
Sitapur	56.4 (51.1-61.9)	46.8 (42.1-51.7)	39.1 (31.6-48.8)	-1.85 (-2.72 to -0.98)	-2.59 (-5.41 to 0.33)	-2.16 (-3.30 to -0.93)
Sonbhadra	46.8 (42.2-52.0)	37.8 (33.9-41.8)	30.7 (24.4-39.0)	-2.11 (-3.12 to -1.09)	-2.97 (-5.93 to 0.12)	-2.47 (-3.81 to -1.14)
Sultanpur	46.8 (42.1-51.5)	33.9 (30.4-37.7)	28.1 (22.4-35.5)	-3.19 (-4.12 to -2.31)	-2.72 (-5.79 to 0.44)	-3.00 (-4.24 to -1.70)
Unnao	48.1 (43.8-52.9)	37.5 (34.0-41.5)	31.0 (24.8-38.5)	-2.44 (-3.37 to -1.55)	-2.78 (-5.62 to 0.12)	-2.59 (-3.78 to -1.33)
Varanasi	46.3 (42.3-50.9)	36.6 (33.1-40.3)	27.4 (21.7-34.5)	-2.32 (-3.19 to -1.40)	-4.11 (-7.16 to -0.89)	-3.06 (-4.35 to -1.70)
**Rajasthan**	**40.5 (34.8-46.4)**	**33.2 (29.1-37.5)**	**26.5 (22.8-31.0)**	**-1.97 (-2.60 to -1.30)**	**-3.21 (-5.70 to -0.92)**	**-2.49 (-3.51 to -1.49)**
Ajmer	42.5 (37.7-47.2)	33.8 (29.9-37.7)	25.7 (20.2-32.0)	-2.25 (-3.33 to -1.15)	-3.90 (-6.94 to -1.05)	-2.94 (-4.25 to -1.66)
Alwar	34.4 (31.1-37.8)	28.4 (25.4-31.4)	25.4 (20.0-31.4)	-1.92 (-2.83 to -0.99)	-1.66 (-4.61 to 1.28)	-1.82 (-2.98 to -0.58)
Banswara	46.4 (41.8-51.7)	36.6 (32.5-40.9)	26.7 (20.8-33.8)	-2.36 (-3.44 to -1.35)	-4.45 (-7.51 to -1.26)	-3.23 (-4.61 to -1.83)
Baran	52.2 (47.0-57.6)	40.0 (35.9-44.6)	31.8 (25.2-40.1)	-2.64 (-3.54 to -1.68)	-3.23 (-6.10 to -0.30)	-2.88 (-4.12 to -1.59)
Barmer	47.9 (42.2-53.7)	43.0 (37.8-48.6)	35.6 (28.0-44.5)	-1.07 (-2.28 to 0.21)	-2.74 (-5.92 to 0.42)	-1.76 (-3.23 to -0.47)
Bharatpur	40.9 (37.2-44.8)	34.3 (30.9-37.5)	30.0 (24.0-36.8)	-1.76 (-2.68 to -0.89)	-1.93 (-4.81 to 0.86)	-1.83 (-3.01 to -0.63)

Bhilwara	54.5 (49.3-59.8)	45.0 (40.4-49.8)	32.0 (25.7-39.5)	-1.88 (-2.85 to -0.93)	-4.83 (-7.57 to -2.10)	-3.11 (-4.34 to -1.86)
Bikaner	25.7 (22.2-29.1)	19.2 (16.8-21.9)	20.0 (15.4-25.9)	-2.85 (-4.07 to -1.44)	0.54 (-2.83 to 3.95)	-1.48 (-3.00 to 0.03)
Bundi	54.0 (49.0-59.7)	43.9 (39.2-48.6)	32.9 (26.1-41.4)	-2.07 (-3.04 to -1.06)	-4.13 (-7.09 to -1.30)	-2.93 (-4.23 to -1.64)
Chittorgarh	59.1 (53.4-65.0)	49.6 (44.5-55.0)	34.0 (27.2-42.1)	-1.75 (-2.68 to -0.82)	-5.28 (-8.08 to -2.56)	-3.22 (-4.41 to -1.96)
Churu	24.1 (21.2-27.2)	18.5 (16.3-20.9)	16.9 (13.4-21.7)	-2.61 (-3.77 to -1.40)	-1.31 (-4.40 to 1.96)	-2.08 (-3.45 to -0.63)
Dausa	42.3 (37.9-47.1)	34.2 (30.4-38.2)	28.6(22.7-35.4)	-2.11 (-3.15 to -1.07)	-2.55 (-5.50 to 0.36)	-2.29 (-3.54 to -1.06)
Dholpur	43.6 (39.4-48.0)	35.1 (31.7-38.8)	31.6 (25.0-39.0)	-2.15 (-3.08 to -1.21)	-1.51 (-4.50 to 1.39)	-1.89 (-3.10 to -0.66)
Dungarpur	52.1 (46.3-58.3)	43.5 (38.3-48.9)	30.3 (23.7-38.4)	-1.79 (-2.93 to -0.76)	-5.08 (-7.99 to -1.93)	-3.17 (-4.56 to -1.72)
Hanumangarh	24.7 (21.9-27.8)	17.8 (15.8-20.2)	17.4 (13.7-22.1)	-3.25 (-4.33 to -2.14)	-0.32 (-3.30 to 3.05)	-2.06 (-3.41 to -0.66)
Jaipur	30.7 (27.6-34.0)	23.3 (20.8-26.0)	19.5 (15.5-24.4)	-2.72 (-3.66 to -1.72)	-2.56 (-5.67 to 0.28)	-2.66 (-3.95 to -1.49)
Jaisalmer	34.0 (29.4-39.2)	27.5 (23.8-31.6)	27.6 (21.3-35.5)	-2.08 (-3.51 to -0.60)	-0.01 (-3.23 to 3.79)	-1.24 (-2.87 to 0.27)
Jalore	53.2 (47.4-59.5)	48.5 (42.8-54.6)	36.8 (29.1-46.0)	-0.90 (-2.12 to 0.37)	-3.94 (-7.08 to -0.87)	-2.17 (-3.59 to -0.85)
Jhalawar	53.4 (48.2-59.2)	41.4 (36.9-46.2)	31.3 (24.3-39.5)	-2.54 (-3.50 to -1.53)	-3.93 (-6.92 to -0.88)	-3.12 (-4.46 to -1.79)
Jhunjhunu	26.3 (23.3-29.4)	19.9 (17.5-22.2)	16.8 (13.3-21.1)	-2.78 (-3.82 to -1.69)	-2.38 (-5.34 to 0.67)	-2.62 (-3.94 to -1.31)
Jodhpur	31.5 (27.4-35.5)	27.1 (23.9-30.7)	22.3 (17.3-28.2)	-1.47 (-2.73 to -0.23)	-2.81 (-5.91 to 0.43)	-2.03 (-3.52 to -0.59)
Karauli	49.2 (45.0-54.0)	41.0 (37.2-45.1)	35.1 (28.2-43.0)	-1.82 (-2.74 to -0.96)	-2.21 (-5.10 to 0.55)	-1.98 (-3.17 to -0.85)
Kota	49.8 (45.2-54.8)	38.8 (34.6-42.9)	28.6 (22.5-35.6)	-2.45 (-3.41 to -1.46)	-4.25 (-7.15 to -1.37)	-3.20 (-4.45 to -1.95)
Nagaur	26.2 (22.9-29.4)	21.7 (19.0-24.5)	18.2 (14.2-23.2)	-1.86 (-3.06 to -0.61)	-2.56 (-5.57 to 0.64)	-2.16 (-3.59 to -0.79)
Pali	51.2 (46.1-56.7)	45.1 (40.2-49.9)	32.0 (25.6-39.9)	-1.26 (-2.25 to -0.23)	-4.85 (-7.70 to -1.95)	-2.76 (-4.07 to -1.57)
Pratapgarh	44.6 (39.7-50.2)	35.5 (31.2-40.3)	25.4 (19.7-32.3)	-2.28 (-3.35 to -1.20)	-4.66 (-7.70 to -1.49)	-3.27 (-4.61 to -1.84)
Rajsamand	56.7 (51.0-62.9)	48.7 (43.5-54.0)	33.1 (26.4-41.0)	-1.49 (-2.46 to -0.51)	-5.46 (-8.24 to -2.64)	-3.15 (-4.39 to -1.77)
Sawai Madhopur	55.8 (50.7-61.6)	46.8 (42.0-51.9)	38.3 (30.3-47.8)	-1.75 (-2.78 to -0.78)	-2.87 (-5.87 to -0.04)	-2.22 (-3.47 to -0.98)
Sikar	23.5 (20.5-26.6)	18.1 (15.9-20.5)	15.5 (12.1-19.7)	-2.59 (-3.74 to -1.36)	-2.24 (-5.45 to 0.90)	-2.45 (-3.83 to -1.09)
Sirohi	54.4 (48.7-60.6)	49.3 (44.0-55.3)	35.2 (28.0-43.9)	-0.98 (-2.12 to 0.17)	-4.75 (-7.77 to -1.73)	-2.55 (-3.88 to -1.24)
Sri Ganganagar	26.4 (23.4-29.8)	18.3 (16.1-20.9)	19.6 (15.2-25.0)	-3.59 (-4.85 to -2.44)	0.89 (-2.23 to 4.21)	-1.77 (-3.18 to -0.33)
Tonk	51.4 (46.4-56.7)	41.4 (37.0-46.3)	33.0 (25.8-41.1)	-2.14 (-3.09 to -1.16)	-3.26 (-6.24 to -0.46)	-2.60 (-3.89 to -1.38)
Udaipur	58.4 (52.2-65.1)	51.5 (46.0-57.5)	35.2 (27.8-44.1)	-1.26 (-2.41 to -0.19)	-5.33 (-8.27 to -2.32)	-2.96 (-4.25 to -1.57)
**Chhattisgarh**	**48.2 (42.1-55.0)**	**39.5 (32.9-46.3)**	**29.2 (25.3-34.0)**	**-1.97 (-2.84 to -1.13)**	**-4.26 (-7.06 to -1.37)**	**-2.92 (-4.07 to -1.69)**
Balod	46.7 (41.0-52.9)	37.6 (32.9-43.0)	28.3 (21.9-36.1)	-2.15 (-3.38 to -0.93)	-4.03 (-7.34 to -0.72)	-2.93 (-4.33 to -1.49)
Baloda Bazar	47.0 (42.2-52.0)	39.3 (35.2-44.1)	28.3 (22.4-35.3)	-1.76 (-2.82 to -0.75)	-4.65 (-7.58 to -1.62)	-2.96 (-4.27 to -1.66)
Balrampur	51.0 (45.9-56.8)	43.6 (39.0-48.8)	31.1 (24.8-39.0)	-1.56 (-2.58 to -0.56)	-4.80 (-7.62 to -1.78)	-2.91 (-4.13 to -1.61)
Bastar	44.8 (39.4-50.4)	38.1 (33.6-43.4)	30.2 (23.3-38.1)	-1.61 (-2.85 to -0.45)	-3.33 (-6.49 to -0.07)	-2.33 (-3.71 to -0.90)
Bemetara	49.3 (43.8-55.8)	39.0 (34.3-44.4)	30.4 (23.3-39.3)	-2.31 (-3.48 to -1.16)	-3.56 (-6.76 to -0.27)	-2.83 (-4.29 to -1.37)
Bijapur	39.6 (33.8-46.1)	32.6 (27.7-38.2)	28.1 (21.5-36.2)	-1.94 (-3.37 to -0.54)	-2.15 (-5.38 to 1.45)	-2.03 (-3.42 to -0.50)
Bilaspur	48.8 (43.8-54.1)	38.7 (34.3-43.4)	29.4 (22.9-36.7)	-2.29 (-3.39 to -1.28)	-3.90 (-7.11 to -0.78)	-2.96 (-4.28 to -1.62)
Dantewada	37.6 (32.3-43.6)	32.7 (28.2-38.1)	27.4 (20.8-35.3)	-1.39 (-2.86 to -0.03)	-2.55 (-5.85 to 0.91)	-1.87 (-3.33 to -0.32)
Dhamtari	45.3 (40.0-50.7)	37.2 (32.8-42.8)	26.6 (20.5-33.6)	-1.94 (-3.14 to -0.77)	-4.75 (-7.98 to -1.45)	-3.12 (-4.51 to -1.70)
Durg	44.2 (39.0-49.4)	35.1 (30.7-39.8)	25.5 (19.5-32.3)	-2.29 (-3.42 to -1.13)	-4.51 (-7.75 to -1.16)	-3.21 (-4.61 to -1.78)
Gariaband	50.4 (44.8-56.1)	42.6 (37.6-47.9)	30.8 (24.0-39.1)	-1.69 (-2.80 to -0.52)	-4.63 (-7.80 to -1.43)	-2.92 (-4.26 to -1.55)
Janjgir-Champa	49.8 (44.4-55.3)	41.7 (37.1-46.6)	28.4 (22.1-35.8)	-1.78 (-2.85 to -0.67)	-5.36 (-8.36 to -2.23)	-3.28 (-4.63 to -1.92)
Jashpur	48.8 (43.5-54.8)	40.7 (35.8-45.8)	27.5 (21.7-34.7)	-1.80 (-2.94 to -0.68)	-5.51 (-8.57 to -2.37)	-3.35 (-4.62 to -2.00)
Kabirdham	53.1 (46.7-60.5)	41.3 (35.8-47.3)	33.2 (24.9-43.6)	-2.50 (-3.72 to -1.27)	-3.14 (-6.45 to 0.24)	-2.77 (-4.28 to -1.30)
Kondagaon	55.8 (49.0-63.7)	47.8 (42.1-54.2)	37.9 (29.4-47.5)	-1.94 (-3.18 to -0.78)	-4.64 (-7.77 to -1.48)	-3.06 (-4.46 to -1.68)
Korba	48.1 (42.5-54.0)	39.5 (34.8-44.3)	28.5 (22.1-35.6)	-2.52 (-3.63 to -1.41)	-3.13 (-6.36 to 0.07)	-2.78 (-4.19 to -1.40)
Korea	49.8 (44.4-55.7)	38.5 (33.9-43.7)	30.9 (24.0-39.0)	-1.67 (-2.64 to -0.59)	-5.28 (-8.17 to -2.11)	-3.18 (-4.51 to -1.80)
Mahasamund	47.3 (42.3-52.7)	40.0 (35.8-44.9)	27.5 (21.5-35.2)	-2.31 (-3.47 to -1.14)	-3.50 (-6.72 to -0.10)	-2.81 (-4.24 to -1.42)
Mungeli	50.4 (44.8-57.0)	39.9 (34.9-45.3)	31.2 (23.9-40.0)	-1.21 (-2.70 to 0.13)	-2.68 (-5.98 to 0.77)	-1.83 (-3.19 to -0.28)
Narayanpur	53.8 (46.2-62.6)	47.6 (40.7-55.2)	39.6 (30.2-51.0)	-1.83 (-2.88 to -0.80)	-5.65 (-8.64 to -2.52)	-3.42 (-4.68 to -2.07)
North Bastar Kanker	50.5 (44.1-57.6)	42.6 (37.2-48.5)	34.2 (26.5-43.7)	-1.54 (-2.78 to -0.38)	-3.35 (-6.59 to -0.11)	-2.29 (-3.57 to -0.88)
Raigarh	48.8 (43.8-54.0)	40.6 (36.5-45.2)	27.2 (21.8-34.1)	-1.92 (-3.03 to -0.81)	-5.05 (-8.17 to -1.81)	-3.23 (-4.65 to -1.84)
Raipur	44.4 (39.2-49.9)	36.6 (32.3-41.7)	25.6 (19.8-32.5)	-2.16 (-3.33 to -0.95)	-3.48 (-6.91 to -0.14)	-2.71 (-4.05 to -1.25)
Rajnandgaon	46.7 (40.8-52.7)	37.5 (32.9-43.3)	29.4 (22.5-37.8)	-1.94 (-3.23 to -0.59)	-2.59 (-5.89 to 0.95)	-2.21 (-3.70 to -0.72)
Sukma	46.4 (39.8-54.1)	38.1 (32.5-44.8)	31.9 (23.9-41.2)	-2.23 (-3.23 to -1.22)	-4.00 (-7.16 to -0.86)	-2.97 (-4.24 to -1.71)
Surajpur	50.8 (45.9-56.7)	40.6 (36.0-45.3)	30.6 (24.3-38.6)	-2.01 (-3.09 to -0.98)	-4.84 (-7.93 to -1.70)	-3.19 (-4.47 to -1.88)
Surguja	48.6 (43.7-54.1)	39.6 (35.1-44.4)	28.1 (22.2-34.8)	-1.67 (-2.96 to -0.51)	-3.15 (-6.47 to 0.11)	-2.29 (-3.63 to -0.81)
**Odisha**	**45.3 (39.9-51.1)**	**33.2 (27.7-39.0)**	**25.5 (21.8-30.2)**	**-3.06 (-3.84 to -2.23)**	**-3.78 (-6.66 to -1.09)**	**-3.36 (-4.50 to -2.21)**
Angul	51.9 (46.4-58.4)	37.7 (33.4-42.4)	28.2 (21.7-36.0)	-3.15 (-4.30 to -1.99)	-4.14 (-7.28 to -1.02)	-3.57 (-4.96 to -2.18)
Balangir	48.9 (43.3-54.8)	39.5 (34.9-44.6)	30.8 (23.9-39.9)	-2.10 (-3.21 to -1.00)	-3.56 (-6.69 to -0.30)	-2.71 (-4.03 to -1.17)
Balasore	37.4 (32.6-42.4)	25.3 (21.9-29.1)	19.2 (14.5-24.7)	-3.86 (-5.11 to -2.62)	-3.93 (-7.17 to -0.44)	-3.90 (-5.35 to -2.46)
Bargarh	44.5 (39.6-49.8)	36.5 (32.5-41.1)	27.0 (21.0-34.5)	-1.95 (-2.99 to -0.87)	-4.22 (-7.24 to -0.90)	-2.90 (-4.17 to -1.42)
Bhadrak	42.3 (37.6-47.6)	26.7 (23.4-30.1)	20.6 (15.7-26.3)	-4.54 (-5.71 to -3.31)	-3.69 (-7.00 to -0.29)	-4.20 (-5.69 to -2.80)
Boudh	51.9 (46.0-58.2)	39.5 (34.5-44.7)	30.1 (23.2-38.5)	-2.68 (-3.82 to -1.46)	-3.87 (-7.20 to -0.58)	-3.18 (-4.56 to -1.69)
Cuttack	39.7 (35.3-44.3)	24.0 (21.0-26.9)	17.4 (13.5-22.0)	-4.90 (-6.04 to -3.70)	-4.62 (-7.96 to -1.36)	-4.79 (-6.16 to -3.40)
Deogarh	51.2 (45.9-57.2)	40.5 (36.2-45.2)	30.0 (23.3-37.8)	-2.32 (-3.39 to -1.22)	-4.27 (-7.39 to -1.21)	-3.13 (-4.55 to -1.83)
Dhenkanal	48.8 (43.5-54.8)	31.8 (28.3-35.8)	23.8 (18.3-30.1)	-4.21 (-5.27 to -3.03)	-4.11 (-7.25 to -0.90)	-4.18 (-5.55 to -2.87)
Gajapati	50.0 (43.6-56.4)	37.2 (32.3-42.8)	30.3 (22.9-38.8)	-2.95 (-4.27 to -1.59)	-2.93 (-6.60 to 0.65)	-2.95 (-4.52 to -1.44)
Ganjam	51.5 (44.8-58.7)	35.1 (30.3-40.8)	27.2 (20.5-35.1)	-3.75 (-5.10 to -2.31)	-3.65 (-7.17 to -0.34)	-3.71 (-5.15 to -2.26)
Jagatsinghapur	36.2 (31.9-40.9)	21.2 (18.4-24.4)	15.6 (11.8-20.1)	-5.20 (-6.47 to -3.86)	-4.41 (-7.88 to -1.05)	-4.88 (-6.34 to -3.42)
Jajapur	47.1 (42.2-53.1)	29.4 (25.8-33.1)	22.0 (17.1-28.0)	-4.63 (-5.76 to -3.43)	-4.10 (-7.37 to -0.86)	-4.42 (-5.83 to -3.08)
Jharsuguda	41.3 (36.5-46.6)	34.6 (30.6-38.9)	24.4 (19.1-31.0)	-1.76 (-2.87 to -0.60)	-4.89 (-8.00 to -1.74)	-3.07 (-4.41 to -1.69)
Kalahandi	55.2 (49.0-61.7)	45.6 (40.3-51.7)	37.4 (29.0-46.9)	-1.87 (-3.02 to -0.67)	-2.86 (-6.02 to 0.36)	-2.29 (-3.62 to -0.86)
Kandhamal	54.7 (48.5-61.5)	41.4 (36.2-47.1)	32.6 (25.2-41.2)	-2.74 (-3.88 to -1.67)	-3.41 (-6.70 to -0.07)	-3.02 (-4.36 to -1.64)
Kendrapara	40.7 (35.4-46.6)	24.6 (21.1-28.4)	18.6 (14.0-24.0)	-4.91 (-6.26 to -3.51)	-4.01 (-7.56 to -0.49)	-4.55 (-6.08 to -3.03)
Kendujhar	49.1 (43.8-54.8)	39.0 (34.6-43.8)	28.4 (22.2-35.7)	-2.30 (-3.43 to -1.16)	-4.49 (-7.58 to -1.39)	-3.21 (-4.58 to -1.93)
Khordha	35.8 (31.2-40.6)	21.9 (18.8-24.8)	15.5 (11.7-19.8)	-4.81 (-6.07 to -3.49)	-4.83 (-8.27 to -1.51)	-4.82 (-6.30 to -3.42)
Koraput	51.5 (45.7-58.3)	42.8 (37.5-48.9)	37.1 (28.5-47.3)	-1.85 (-3.14 to -0.62)	-2.07 (-5.35 to 1.31)	-1.94 (-3.27 to -0.52)
Malkangiri	46.4 (40.4-53.6)	38.6 (33.6-44.6)	35.7 (26.8-46.1)	-1.85 (-3.18 to -0.49)	-1.14 (-4.53 to 2.39)	-1.56 (-3.05 to -0.04)
Mayurbhanj	38.8 (34.5-43.3)	31.3 (27.8-35.2)	23.1 (18.0-29.3)	-2.16 (-3.32 to -0.99)	-4.36 (-7.48 to -1.15)	-3.07 (-4.38 to -1.74)
Nabarangpur	49.9 (44.3-55.4)	42.2 (37.5-47.5)	36.4 (28.3-45.9)	-1.65 (-2.78 to -0.51)	-2.16 (-5.31 to 1.00)	-1.86 (-3.19 to -0.50)
Nayagarh	44.0 (39.2-49.3)	28.6 (25.0-32.3)	22.1 (17.2-27.8)	-4.20 (-5.33 to -3.04)	-3.67 (-6.98 to -0.49)	-3.99 (-5.32 to -2.63)
Nuapada	46.7 (41.6-51.6)	39.8 (35.2-44.5)	31.8 (24.7-40.1)	-1.57 (-2.67 to -0.42)	-3.19 (-6.42 to 0.08)	-2.24 (-3.58 to -0.81)
Puri	33.2 (29.0-38.0)	20.5 (17.6-23.7)	14.8 (11.1-19.0)	-4.71 (-6.01 to -3.39)	-4.59 (-8.13 to -1.17)	-4.67 (-6.15 to -3.25)
Rayagada	58.4 (51.8-65.3)	47.4 (41.8-53.5)	39.6 (30.4-50.1)	-2.06 (-3.25 to -0.91)	-2.57 (-5.81 to 0.60)	-2.27 (-3.66 to -0.90)
Sambalpur	44.8 (40.1-50.0)	36.9 (32.8-41.1)	26.4 (20.7-33.8)	-1.92 (-2.96 to -0.85)	-4.73 (-7.74 to -1.47)	-3.09 (-4.49 to -1.74)
Sonepur	48.9 (43.3-54.5)	38.4 (34.2-43.2)	28.7 (22.1-37.0)	-2.39 (-3.48 to -1.24)	-4.13 (-7.23 to -0.92)	-3.12 (-4.49 to -1.61)
Sundargarh	47.3 (42.2-53.0)	39.8 (35.5-44.5)	28.0 (22.4-35.1)	-1.71 (-2.78 to -0.65)	-4.95 (-7.95 to -1.89)	-3.06 (-4.36 to -1.79)
**Assam**	**43.5 (38.1-49.3)**	**36.4 (30.6-42.5)**	**29.5 (25.1-35.0)**	**-1.77 (-2.54 to -1.00)**	**-3.00 (-5.87 to -0.38)**	**-2.28 (-3.40 to -1.13)**
Baksa	44.3 (39.5-49.1)	31.7 (28.2-35.7)	22.9 (18.0-28.8)	-3.29 (-4.28 to -2.25)	-4.60 (-7.68 to -1.66)	-3.83 (-5.11 to -2.58)

Barpeta	39.6 (34.8-44.5)	26.7 (23.4-30.4)	20.7 (16.1-26.1)	-3.89 (-4.94 to -2.66)	-3.62 (-6.88 to -0.31)	-3.79 (-5.16 to -2.37)
Biswanath	35.6 (31.1-40.1)	32.1 (28.5-36.3)	25.5 (20.1-32.0)	-1.03 (-2.16 to 0.13)	-3.29 (-6.33 to -0.37)	-1.97 (-3.37 to -0.66)
Bongaigaon	40.9 (36.5-45.5)	26.5 (23.3-29.7)	21.1 (16.7-26.7)	-4.24 (-5.30 to -3.14)	-3.27 (-6.52 to -0.08)	-3.85 (-5.14 to -2.48)
Cachar	51.3 (45.4-57.3)	51.1 (45.3-57.5)	43.9 (34.5-55.5)	-0.03 (-1.13 to 1.07)	-2.20 (-5.40 to 1.06)	-0.93 (-2.31 to 0.50)
Charaideo	38.5 (34.0-43.8)	37.5 (32.9-43.1)	33.2 (25.2-42.4)	-0.25 (-1.60 to 1.14)	-1.76 (-5.43 to 1.61)	-0.88 (-2.40 to 0.62)
Chirang	40.1 (35.5-44.3)	26.2 (23.2-29.5)	20.5 (16.2-25.9)	-4.16 (-5.15 to -3.14)	-3.50 (-6.65 to -0.39)	-3.90 (-5.17 to -2.56)
Darrang	49.1 (43.8-54.9)	40.6 (35.7-45.8)	30.6 (24.3-38.2)	-1.91 (-2.88 to -0.94)	-4.04 (-7.17 to -1.26)	-2.80 (-4.06 to -1.57)
Dhemaji	35.8 (31.4-41.0)	31.1 (27.5-35.5)	25.9 (19.8-33.0)	-1.38 (-2.68 to 0.03)	-2.62 (-6.14 to 0.81)	-1.90 (-3.39 to -0.40)
Dhubri	49.0 (43.8-54.7)	31.9 (28.1-35.8)	25.4 (20.1-32.7)	-4.17 (-5.31 to -3.04)	-3.32 (-6.36 to 0.05)	-3.83 (-5.07 to -2.44)
Dibrugarh	35.9 (31.5-40.9)	33.1 (29.2-37.8)	28.6 (21.9-36.3)	-0.79 (-2.15 to 0.69)	-2.13 (-5.66 to 1.33)	-1.35 (-2.89 to 0.10)
Dima Hasao	49.3 (43.7-55.4)	47.7 (42.4-53.2)	42.9 (34.0-53.7)	-0.32 (-1.43 to 0.71)	-1.56 (-4.63 to 1.45)	-0.84 (-2.19 to 0.54)
Goalpara	41.9 (37.6-46.2)	26.7 (23.7-29.8)	21.8 (17.3-27.7)	-4.42 (-5.40 to -3.42)	-2.92 (-6.08 to 0.22)	-3.81 (-5.06 to -2.48)
Golaghat	39.9 (35.2-45.1)	37.1 (32.8-41.8)	31.7 (24.9-39.6)	-0.74 (-1.95 to 0.54)	-2.28 (-5.48 to 0.76)	-1.38 (-2.74 to -0.01)
Hailakandi	52.7 (47.0-58.7)	51.8 (46.3-57.9)	45.0 (35.3-57.1)	-0.17 (-1.22 to 0.93)	-2.05 (-5.41 to 1.35)	-0.95 (-2.34 to 0.49)
Hojai	50.9 (44.6-57.8)	49.4 (43.2-56.0)	40.8 (31.8-51.7)	-0.30 (-1.50 to 0.88)	-2.81 (-5.99 to 0.22)	-1.35 (-2.67 to 0.06)
Jorhat	43.1 (37.9-48.8)	39.8 (34.9-45.7)	32.9 (25.5-41.7)	-0.80 (-2.08 to 0.51)	-2.76 (-6.15 to 0.42)	-1.62 (-3.10 to -0.17)
Kamrup	40.4 (36.0-44.7)	28.6 (25.4-32.2)	21.6 (17.1-26.9)	-3.41 (-4.38 to -2.35)	-3.94 (-7.07 to -0.83)	-3.63 (-4.96 to -2.34)
Kamrup Metropolitan	36.6 (32.7-40.8)	27.5 (24.5-30.9)	20.3 (15.8-25.1)	-3.01 (-3.96 to -1.98)	-4.42 (-7.58 to -1.33)	-3.60 (-4.84 to -2.36)
Karbi Anglong	43.3 (38.4-48.8)	41.2 (36.4-46.4)	35.0 (27.7-43.5)	-0.47 (-1.58 to 0.65)	-2.35 (-5.35 to 0.65)	-1.25 (-2.58 to 0.01)
Karimganj	55.5 (49.4-61.4)	51.3 (45.9-57.2)	45.8 (36.1-58.0)	-0.79 (-1.81 to 0.27)	-1.70 (-4.94 to 1.63)	-1.17 (-2.54 to 0.30)
Kokrajhar	45.8 (40.8-51.2)	29.6 (26.2-33.2)	23.2 (18.6-29.7)	-4.26 (-5.35 to -3.13)	-3.47 (-6.48 to -0.23)	-3.94 (-5.23 to -2.54)
Lakhimpur	38.2 (33.7-42.8)	33.8 (29.7-38.3)	27.2 (21.1-34.1)	-1.22 (-2.42 to 0.00)	-3.15 (-6.58 to 0.04)	-2.02 (-3.45 to -0.59)
Majuli	42.8 (37.7-48.3)	39.0 (34.3-44.4)	31.9 (24.7-40.1)	-0.94 (-2.23 to 0.34)	-2.90 (-6.32 to 0.25)	-1.76 (-3.18 to -0.35)
Morigaon	49.9 (44.5-55.7)	44.0 (39.1-49.2)	34.5 (27.6-43.0)	-1.25 (-2.25 to -0.23)	-3.48 (-6.62 to -0.62)	-2.18 (-3.44 to -0.93)
Nagaon	47.1 (41.7-52.5)	44.2 (39.4-49.6)	34.9 (27.8-43.6)	-0.62 (-1.69 to 0.49)	-3.32 (-6.47 to -0.42)	-1.75 (-3.07 to -0.47)
Nalbari	40.6 (36.0-45.3)	28.2 (24.9-31.9)	20.9 (16.3-26.2)	-3.57 (-4.58 to -2.45)	-4.27 (-7.41 to -1.13)	-3.86 (-5.17 to -2.52)
Sivasagar	41.0 (36.2-46.6)	38.2 (33.7-43.5)	32.6 (25.1-41.4)	-0.70 (-2.01 to 0.63)	-2.32 (-5.97 to 0.93)	-1.37 (-2.83 to 0.09)
Sonitpur	40.4 (35.8-45.3)	35.5 (31.7-39.9)	26.9 (21.4-33.3)	-1.27 (-2.27 to -0.10)	-3.95 (-7.04 to -0.97)	-2.38 (-3.74 to -1.09)
South Salmara Mancachar	48.4 (43.5-53.5)	30.9 (27.6-34.6)	24.5 (19.4-31.3)	-4.46 (-5.52 to -3.42)	-3.20 (-6.21 to 0.29)	-3.95 (-5.22 to -2.52)
Tinsukia	30.4 (26.3-35.0)	28.0 (24.2-32.3)	24.4 (18.1-31.4)	-0.80 (-2.27 to 0.88)	-2.03 (-5.61 to 1.53)	-1.31 (-2.97 to 0.28)
Udalguri	47.4 (42.1-53.5)	38.3 (33.6-43.4)	28.1 (22.2-35.2)	-2.08 (-3.15 to -1.01)	-4.35 (-7.48 to -1.48)	-3.03 (-4.30 to -1.79)
West Karbi Anglong	49.1 (43.2-55.5)	46.6 (41.0-52.6)	39.3 (30.6-49.8)	-0.54 (-1.72 to 0.59)	-2.44 (-5.64 to 0.62)	-1.33 (-2.68 to 0.06)
**Andhra Pradesh**	**37.1 (31.0-43.1)**	**28.4 (22.2-35.7)**	**20.1 (14.2-28.3)**	**-2.64 (-3.84 to -1.48)**	**-4.90 (-7.86 to -1.61)**	**-3.58 (-4.91 to -2.25)**
Anantapur	45.5 (39.3-52.8)	31.9 (27.3-37.7)	23.9 (17.5-31.7)	-3.52 (-4.97 to -2.09)	-4.14 (-7.91 to -0.07)	-3.78 (-5.42 to -2.07)
Chittoor	33.7 (28.7-39.1)	21.9 (18.8-25.7)	17.3 (12.6-23.3)	-4.21 (-5.74 to -2.67)	-3.41 (-7.25 to 0.74)	-3.89 (-5.69 to -2.14)
East Godavari	30.0 (25.4-35.7)	23.2 (19.7-27.8)	17.0 (12.5-22.8)	-2.53 (-4.24 to -0.81)	-4.50 (-8.03 to -0.64)	-3.35 (-5.10 to -1.59)
Guntur	31.3 (26.4-37.6)	25.7 (21.2-31.0)	17.2 (12.3-23.2)	-1.97 (-3.79 to -0.11)	-5.66 (-9.34 to -1.91)	-3.51 (-5.21 to -1.74)
Krishna	31.8 (27.0-37.4)	24.9 (20.8-30.0)	16.5 (11.6-22.3)	-2.44 (-4.13 to -0.68)	-5.81 (-9.36 to -1.92)	-3.85 (-5.54 to -2.09)
Kurnool	48.1 (41.6-55.8)	38.6 (32.9-45.1)	26.9 (19.7-35.7)	-2.16 (-3.65 to -0.58)	-5.12 (-8.96 to -1.29)	-3.40 (-5.07 to -1.74)
Prakasam	32.7 (27.3-38.9)	28.1 (23.0-34.0)	19.8 (14.3-26.5)	-1.54 (-3.34 to 0.35)	-4.95 (-8.84 to -1.00)	-2.96 (-4.75 to -1.19)
Sri Potti Sriramulu Nellore	32.4 (26.5-38.6)	24.7 (20.2-30.3)	18.6 (13.3-25.2)	-2.66 (-4.40 to -0.77)	-4.08 (-7.88 to 0.17)	-3.25 (-5.09 to -1.53)
Srikakulam	46.7 (40.4-53.5)	33.4 (28.5-39.5)	21.9 (16.2-28.8)	-3.30 (-4.80 to -1.84)	-6.02 (-9.68 to -2.57)	-4.44 (-6.06 to -2.79)
Visakhapatnam	37.4 (31.9-43.6)	29.1 (24.5-34.5)	20.4 (15.2-26.9)	-2.48 (-4.11 to -0.95)	-5.04 (-8.52 to -1.37)	-3.55 (-5.11 to -1.93)
Vizianagaram	51.3 (44.4-59.2)	39.7 (33.7-46.7)	26.7 (19.7-35.6)	-2.53 (-4.07 to -0.97)	-5.59 (-9.25 to -2.00)	-3.81 (-5.40 to -2.24)
West Godavari	32.7 (27.5-38.8)	25.3 (20.9-31.0)	17.8 (12.9-24.3)	-2.55 (-4.29 to -0.83)	-4.98 (-8.57 to -1.03)	-3.56 (-5.24 to -1.73)
YSR	40.2 (34.1-46.6)	31.3 (26.3-37.1)	22.5 (16.4-29.7)	-2.50 (-4.05 to -0.93)	-4.70 (-8.53 to -0.94)	-3.42 (-5.21 to -1.77)
**West Bengal**	**31.9 (28.0-36.2)**	**23.8 (19.0-29.2)**	**17.8 (15.3-20.8)**	**-2.86 (-3.71 to -2.02)**	**-4.17 (-6.97 to -1.38)**	**-3.41 (-4.63 to -2.20)**
Alipurduar	32.1 (28.6-35.9)	23.7 (20.8-26.9)	16.8 (13.4-21.7)	-2.99 (-4.17 to -1.85)	-4.85 (-7.73 to -1.52)	-3.76 (-5.06 to -2.39)
Bankura	31.6 (27.9-36.0)	26.8 (23.5-30.4)	19.7 (15.4-25.2)	-1.63 (-2.90 to -0.49)	-4.36 (-7.35 to -1.44)	-2.77 (-4.11 to -1.35)
Birbhum	39.2 (35.0-43.6)	31.6 (28.1-35.4)	23.6 (18.7-29.4)	-2.12 (-3.21 to -1.13)	-4.15 (-6.97 to -1.18)	-2.97 (-4.28 to -1.69)
Cooch Behar	36.5 (32.5-40.6)	27.1 (23.9-30.4)	19.8 (15.8-25.6)	-2.92 (-3.99 to -1.83)	-4.35 (-7.26 to -1.07)	-3.52 (-4.73 to -2.10)
Dakshin Dinajpur	36.2 (31.8-41.0)	27.4 (24.1-31.3)	20.8 (16.3-27.1)	-2.73 (-3.80 to -1.65)	-3.93 (-6.88 to -0.51)	-3.23 (-4.57 to -1.82)
Darjeeling	33.5 (29.6-38.1)	26.7 (23.4-30.5)	17.6 (13.7-22.5)	-2.26 (-3.47 to -0.98)	-5.67 (-8.55 to -2.37)	-3.69 (-5.01 to -2.25)
Hooghly	27.8 (24.6-31.7)	19.0 (16.4-21.6)	14.8 (11.2-19.1)	-3.78 (-4.94 to -2.53)	-3.59 (-7.06 to -0.30)	-3.71 (-5.23 to -2.28)
Howrah	24.6 (21.1-28.3)	16.6 (14.2-19.4)	12.5 (9.3-16.6)	-3.81 (-5.15 to -2.51)	-4.09 (-7.67 to -0.54)	-3.93 (-5.52 to -2.43)
Jalpaiguri	32.7 (29.3-36.6)	26.5 (23.5-29.7)	18.1 (14.3-23.1)	-2.09 (-3.23 to -1.01)	-5.47 (-8.21 to -2.17)	-3.50 (-4.70 to -2.11)
Jhargram	31.4 (27.6-35.8)	25.0 (21.9-28.5)	18.7 (14.5-23.9)	-2.24 (-3.52 to -0.99)	-4.14 (-7.30 to -0.88)	-3.03 (-4.46 to -1.62)
Kalimpong	26.3 (23.3-29.8)	21.3 (18.5-24.3)	13.5 (10.6-17.1)	-2.11 (-3.27 to -0.92)	-6.42 (-9.30 to -3.12)	-3.91 (-5.18 to -2.51)
Kolkata	23.0 (19.8-26.5)	15.7 (13.3-18.2)	11.6 (8.6-15.4)	-3.76 (-5.12 to -2.50)	-4.21 (-7.86 to -0.69)	-3.95 (-5.53 to -2.48)
Maldah	42.5 (38.0-47.2)	34.5 (30.7-38.8)	25.3 (19.9-32.2)	-2.07 (-3.05 to -1.01)	-4.33 (-7.25 to -1.10)	-3.01 (-4.33 to -1.68)
Murshidabad	39.1 (34.6-43.9)	29.4 (26.0-33.2)	21.9 (17.1-27.9)	-2.82 (-3.90 to -1.65)	-4.16 (-7.27 to -0.98)	-3.37 (-4.71 to -1.99)
Nadia	33.2 (28.7-37.5)	23.0 (19.9-26.4)	17.5 (13.2-22.7)	-3.62 (-4.73 to -2.44)	-3.83 (-7.00 to -0.48)	-3.71 (-5.12 to -2.26)
North 24 Parganas	27.3 (24.1-30.9)	18.2 (15.7-20.8)	14.0 (10.4-18.4)	-4.00 (-5.23 to -2.74)	-3.64 (-7.29 to -0.16)	-3.86 (-5.38 to -2.34)
Paschim Burdwan	33.8 (29.7-38.6)	29.5 (25.9-33.4)	20.6 (15.9-26.1)	-1.37 (-2.60 to -0.23)	-5.06 (-8.14 to -2.12)	-2.91 (-4.30 to -1.60)
Pashchim Medinipur	30.2 (26.5-34.5)	22.1 (19.1-25.4)	17.0 (13.0-21.8)	-3.10 (-4.39 to -1.83)	-3.72 (-6.90 to -0.51)	-3.36 (-4.82 to -1.99)
Purba Burdwan	31.4 (27.3-36.2)	23.4 (20.1-27.0)	18.0 (13.7-23.2)	-2.91 (-4.11 to -1.80)	-3.76 (-6.89 to -0.57)	-3.26 (-4.65 to -1.84)
Purba Medinipur	28.9 (24.8-32.9)	19.5 (16.6-22.8)	15.0 (11.2-19.6)	-3.86 (-5.18 to -2.56)	-3.71 (-7.25 to -0.06)	-3.80 (-5.36 to -2.35)
Puruliya	37.9 (33.7-42.2)	34.8 (30.9-38.6)	24.0 (19.2-30.2)	-0.83 (-1.85 to 0.16)	-5.26 (-8.17 to -2.24)	-2.68 (-3.93 to -1.38)
South 24 Parganas	28.5 (24.6-32.8)	19.6 (16.6-22.9)	14.9 (11.0-19.4)	-3.60 (-4.98 to -2.27)	-4.07 (-7.75 to -0.43)	-3.80 (-5.39 to -2.28)
Uttar Dinajpur	39.4 (35.3-43.8)	32.9 (29.3-36.9)	24.4 (19.3-31.1)	-1.76 (-2.78 to -0.79)	-4.22 (-7.16 to -0.90)	-2.78 (-4.08 to -1.40)
**Tripura**	**29.9 (25.7-34.4)**	**23.3 (18.1-29.6)**	**18.7 (16.3-22.0)**	**-2.46 (-3.53 to -1.36)**	**-3.15 (-6.61 to 0.37)**	**-2.75 (-4.30 to -1.39)**
Dhalai	32.2 (28.2-36.3)	26.8 (23.3-30.6)	23.0 (17.7-29.8)	-1.86 (-3.11 to -0.63)	-2.20 (-5.70 to 1.51)	-2.01 (-3.53 to -0.57)
Gomati	27.5 (24.3-30.8)	21.9 (19.1-25.1)	17.4 (13.4-22.3)	-2.24 (-3.40 to -0.99)	-3.25 (-6.72 to 0.32)	-2.67 (-4.20 to -1.25)
Khowai	33.0 (29.2-36.8)	24.4 (21.5-27.6)	19.5 (15.1-25.3)	-2.97 (-4.05 to -1.87)	-3.19 (-6.76 to 0.41)	-3.07 (-4.62 to -1.70)
North Tripura	36.0 (31.9-40.2)	31.3 (27.7-35.3)	27.0 (20.9-34.7)	-1.40 (-2.53 to -0.23)	-2.17 (-5.65 to 1.37)	-1.72 (-3.20 to -0.28)
Sepahijala	26.7 (23.6-29.7)	19.8 (17.4-22.7)	15.5 (11.9-19.9)	-2.93 (-4.02 to -1.76)	-3.59 (-7.16 to 0.01)	-3.21 (-4.68 to -1.81)
South Tripura	24.7 (21.4-28.0)	19.6 (16.8-22.9)	15.8 (12.1-20.8)	-2.27 (-3.52 to -0.94)	-3.07 (-6.61 to 0.55)	-2.60 (-4.13 to -1.10)
Unakoti	33.9 (30.0-38.0)	27.8 (24.5-31.5)	23.7 (18.4-30.5)	-1.98 (-3.15 to -0.82)	-2.32 (-5.84 to 1.29)	-2.12 (-3.61 to -0.68)
West Tripura	29.4 (26.1-32.7)	21.2 (18.6-24.1)	15.6 (12.1-20.3)	-3.21 (-4.30 to -2.07)	-4.29 (-7.87 to -0.74)	-3.66 (-5.19 to -2.27)
**Arunachal Pradesh**	**28.7 (24.2-33.4)**	**19.2 (15.8-22.9)**	**14.4 (12.4-16.9)**	**-3.92 (-5.25 to -2.45)**	**-4.08 (-7.35 to -0.81)**	**-3.99 (-5.54 to -2.49)**
Anjaw	24.3 (18.8-31.1)	16.5 (12.8-20.8)	13.2 (9.0-19.0)	-3.82 (-6.01 to -1.46)	-3.18 (-7.48 to 0.92)	-3.57 (-5.66 to -1.51)
Changlang	29.7 (25.0-35.0)	21.0 (17.6-24.7)	16.2 (11.8-21.4)	-3.49 (-5.08 to -1.71)	-3.75 (-7.50 to -0.24)	-3.60 (-5.38 to -1.94)
Dibang Valley	19.7 (14.9-25.2)	11.7 (8.8-15.1)	8.7 (5.9-12.6)	-5.10 (-7.11 to -2.89)	-4.11 (-8.29 to -0.31)	-4.70 (-6.63 to -2.84)
East Kameng	31.9 (26.6-38.1)	18.3 (15.0-22.1)	12.4 (9.4-16.0)	-5.40 (-6.71 to -3.99)	-5.52 (-8.74 to -2.46)	-5.45 (-6.86 to -4.03)
East Siang	26.1 (22.2-30.6)	16.2 (13.8-19.1)	12.2 (9.0-16.0)	-4.85 (-6.34 to -3.19)	-4.26 (-7.99 to -0.76)	-4.61 (-6.23 to -2.91)
Kamle	29.9 (25.8-34.2)	17.7 (15.4-20.4)	12.2 (9.4-15.6)	-5.10 (-6.38 to -3.71)	-5.21 (-8.56 to -1.99)	-5.15 (-6.53 to -3.67)
Kra Daddi	27.4 (22.5-32.6)	15.8 (12.9-19.1)	10.9 (8.0-14.2)	-5.32 (-6.95 to -3.73)	-5.27 (-8.79 to -1.82)	-5.30 (-6.82 to -3.68)
Kurung Kumey	33.1 (27.4-39.8)	17.6 (14.5-21.3)	12.5 (9.5-16.3)	-6.10 (-7.58 to -4.64)	-4.89 (-8.30 to -1.73)	-5.61 (-7.08 to -4.14)

Lohit	25.2 (21.1-29.7)	16.8 (14.1-19.7)	12.6 (9.2-16.7)	-3.99 (-5.71 to -2.12)	-4.07 (-7.77 to -0.38)	-4.03 (-5.86 to -2.28)
Longding	33.2 (28.7-38.5)	25.0 (21.4-29.2)	19.5 (14.7-25.4)	-2.83 (-4.24 to -1.30)	-3.55 (-7.09 to -0.14)	-3.13 (-4.68 to -1.63)
Lower Dibang Valley	23.4 (19.5-27.6)	14.8 (12.2-17.7)	11.0 (8.0-14.7)	-4.49 (-6.15 to -2.57)	-4.26 (-8.05 to -0.56)	-4.41 (-6.20 to -2.62)
Lower Siang	32.4 (28.0-37.7)	21.1 (18.3-24.4)	15.3 (11.6-19.6)	-4.78 (-6.14 to -3.25)	-5.04 (-8.68 to -1.55)	-4.89 (-6.39 to -3.36)
Lower Subansiri	28.2 (24.4-32.4)	17.0 (14.7-19.6)	11.3 (8.6-14.3)	-4.96 (-6.24 to -3.59)	-5.67 (-9.03 to -2.46)	-5.26 (-6.72 to -3.80)
Namsai	27.6 (23.4-32.0)	18.6 (15.9-21.7)	13.9 (10.3-18.3)	-3.85 (-5.36 to -2.13)	-4.19 (-7.76 to -0.70)	-4.00 (-5.64 to -2.32)
Pakke Kessang	29.9 (25.9-34.5)	19.0 (16.6-22.0)	12.1 (9.4-15.2)	-4.42 (-5.61 to -3.13)	-6.31 (-9.44 to -3.27)	-5.21 (-6.64 to -3.82)
Papum Pare	32.3 (28.5-36.3)	20.6 (18.2-23.2)	13.8 (10.9-17.2)	-4.73 (-5.85 to -3.61)	-5.84 (-9.01 to -2.84)	-5.20 (-6.58 to -3.84)
Shi Yomi	26.4 (22.6-30.6)	15.5 (13.2-18.0)	10.6 (8.0-13.8)	-5.23 (-6.54 to -3.71)	-5.34 (-8.98 to -1.98)	-5.28 (-6.81 to -3.71)
Siang	20.0 (16.3-24.1)	11.0 (8.9-13.5)	7.9 (5.6-10.9)	-5.75 (-7.51 to -3.98)	-4.68 (-8.57 to -1.03)	-5.31 (-7.07 to -3.56)
Tawang	31.6 (27.5-36.3)	18.1 (15.5-21.0)	11.5 (9.0-14.4)	-5.46 (-6.68 to -4.18)	-6.35 (-9.41 to -3.41)	-5.83 (-7.18 to -4.53)
Tirap	34.4 (29.2-40.7)	25.2 (21.2-30.1)	18.8 (13.9-24.8)	-3.07 (-4.51 to -1.38)	-4.18 (-7.85 to -0.56)	-3.53 (-5.19 to -1.98)
Upper Siang	19.5 (15.3-24.1)	10.6 (8.2-13.4)	7.8 (5.4-11.1)	-5.90 (-7.81 to -3.86)	-4.35 (-8.42 to -0.66)	-5.27 (-7.11 to -3.44)
Upper Subansiri	25.5 (21.1-30.5)	14.4 (11.7-17.5)	10.2 (7.4-13.6)	-5.58 (-7.15 to -3.98)	-4.87 (-8.56 to -1.46)	-5.30 (-6.86 to -3.72)
West Kameng	31.3 (27.7-35.3)	18.2 (15.9-20.7)	11.6 (9.2-14.4)	-5.21 (-6.30 to -4.09)	-6.32 (-9.39 to -3.52)	-5.67 (-6.98 to -4.41)
West Siang	21.4 (16.8-26.6)	11.9 (9.2-14.9)	8.6 (5.9-11.8)	-5.71 (-7.48 to -3.88)	-4.56 (-8.33 to -1.05)	-5.25 (-6.96 to -3.59)
**Meghalaya**	**24.9 (20.6-29.7)**	**21.5 (17.1-26.0)**	**18.8 (16.1-22.1)**	**-1.43 (-2.28 to -0.59)**	**-1.98 (-5.01 to 1.21)**	**-1.66 (-2.88 to -0.36)**
East Garo Hills	22.1 (19.8-24.5)	17.3 (15.3-19.4)	14.3 (11.3-18.3)	-2.40 (-3.37 to -1.38)	-2.62 (-5.82 to 0.69)	-2.49 (-3.78 to -1.09)
East Jaintia Hills	32.5 (28.7-36.2)	36.0 (32.1-40.1)	35.0 (27.5-43.9)	1.07 (0.03 to 2.09)	-0.46 (-3.61 to 2.78)	0.43 (-0.96 to 1.84)
East Khasi Hills	23.8 (21.3-26.5)	20.8 (18.5-23.2)	18.0 (14.1-22.3)	-1.37 (-2.40 to -0.37)	-2.07 (-5.21 to 1.09)	-1.67 (-3.01 to -0.33)
North Garo Hills	23.4 (20.8-25.9)	18.1 (16.0-20.2)	15.4 (12.2-19.4)	-2.41 (-3.39 to -1.38)	-2.15 (-5.35 to 1.09)	-2.31 (-3.60 to -0.94)
Ri Bhoi	25.3 (22.7-27.9)	24.4 (21.9-27.0)	21.2 (16.8-25.9)	-0.31 (-1.32 to 0.68)	-2.45 (-5.64 to 0.52)	-1.20 (-2.50 to 0.09)
South Garo Hills	24.1 (21.5-26.9)	17.7 (15.6-20.1)	14.0 (11.0-18.0)	-3.06 (-4.09 to -2.00)	-3.41 (-6.64 to 0.10)	-3.21 (-4.51 to -1.80)
South West Garo Hills	29.3 (26.0-32.8)	21.0 (18.4-24.0)	17.2 (13.4-22.2)	-2.98 (-4.11 to -1.80)	-2.59 (-5.73 to 1.06)	-2.83 (-4.19 to -1.19)
South West Khasi Hills	23.6 (21.0-26.3)	19.0 (16.8-21.5)	17.1 (13.4-21.5)	-2.14 (-3.16 to -1.07)	-1.78 (-5.07 to 1.50)	-2.00 (-3.35 to -0.59)
West Garo Hills	25.4 (22.8-28.1)	19.4 (17.2-21.8)	16.2 (12.8-20.9)	-2.73 (-3.77 to -1.66)	-2.69 (-5.76 to 0.86)	-2.71 (-4.04 to -1.22)
West Jaintia Hills	27.5 (24.5-30.7)	28.2 (25.2-31.4)	26.6 (21.0-33.0)	0.29 (-0.74 to 1.29)	-0.86 (-4.00 to 2.24)	-0.18 (-1.50 to 1.14)
West Khasi Hills	22.6 (20.3-24.9)	18.7 (16.6-20.8)	16.6 (13.1-20.8)	-1.85 (-2.81 to -0.92)	-1.78 (-5.03 to 1.39)	-1.83 (-3.12 to -0.48)
**Karnataka**	**33.0 (29.4-36.9)**	**24.2 (20.5-28.3)**	**18.3 (15.8-21.5)**	**-3.05 (-4.22 to -1.94)**	**-3.96 (-7.40 to -0.31)**	**-3.43 (-4.87 to -2.06)**
Bagalkot	40.9 (35.8-46.7)	29.5 (25.1-33.8)	21.7 (16.1-28.5)	-3.21 (-4.66 to -1.71)	-4.41 (-8.61 to -0.34)	-3.71 (-5.28 to -1.92)
Ballari	45.3 (38.9-52.2)	35.4 (30.2-40.7)	26.7 (19.7-35.8)	-2.44 (-3.96 to -0.91)	-4.04 (-7.85 to 0.05)	-3.11 (-4.85 to -1.41)
Belagavi	29.7 (25.7-34.1)	21.7 (18.5-25.4)	16.3 (12.0-21.6)	-3.07 (-4.56 to -1.59)	-4.16 (-8.26 to 0.02)	-3.53 (-5.21 to -1.83)
Bengaluru Rural	33.5 (29.0-38.2)	24.0 (20.8-28.1)	19.0 (13.9-25.0)	-3.30 (-4.76 to -1.81)	-3.46 (-7.41 to 0.53)	-3.37 (-5.08 to -1.70)
Bengaluru Urban	27.7 (23.5-32.2)	20.6 (17.5-24.2)	15.4 (11.2-20.6)	-2.93 (-4.44 to -1.35)	-4.20 (-8.20 to -0.16)	-3.46 (-5.29 to -1.75)
Bidar	26.5 (22.7-30.5)	23.9 (20.3-27.6)	16.9 (12.7-21.8)	-1.02 (-2.60 to 0.54)	-4.88 (-8.42 to -1.44)	-2.63 (-4.20 to -1.07)
Chamarajanagar	33.4 (28.2-39.5)	24.2 (20.2-29.2)	18.2 (12.8-24.6)	-3.13 (-4.73 to -1.47)	-4.09 (-8.13 to -0.01)	-3.53 (-5.33 to -1.78)
Chikballapur	33.4 (28.8-38.6)	25.1 (21.6-29.6)	20.5 (14.7-27.1)	-2.84 (-4.31 to -1.36)	-2.94 (-6.85 to 1.14)	-2.89 (-4.68 to -1.25)
Chikkamagaluru	28.8 (24.4-33.9)	18.8 (15.8-22.6)	14.4 (10.3-19.5)	-4.16 (-5.85 to -2.36)	-3.84 (-8.06 to 0.48)	-4.04 (-5.87 to -2.25)
Chitradurga	38.3 (32.6-44.2)	27.1 (23.0-31.9)	21.4 (15.6-28.7)	-3.40 (-4.90 to -1.81)	-3.44 (-7.45 to 0.67)	-3.42 (-5.25 to -1.71)
Dakshina Kannada	15.3 (12.5-18.4)	10.3 (8.4-12.5)	7.5 (5.1-10.6)	-3.88 (-5.78 to -1.90)	-4.53 (-8.78 to -0.16)	-4.16 (-6.18 to -2.28)
Davanagere	40.2 (34.9-46.3)	28.6 (24.3-33.3)	21.0 (15.2-27.7)	-3.37 (-4.90 to -1.79)	-4.42 (-8.37 to -0.17)	-3.81 (-5.57 to -2.15)
Dharwad	39.2 (33.6-45.2)	27.1 (23.2-31.7)	20.5 (14.9-27.8)	-3.63 (-5.13 to -2.05)	-4.00 (-8.13 to 0.32)	-3.79 (-5.44 to -1.91)
Gadag	49.0 (42.8-56.2)	35.3 (30.0-41.1)	26.4 (19.3-35.4)	-3.24 (-4.71 to -1.70)	-4.16 (-8.41 to 0.11)	-3.63 (-5.32 to -1.69)
Hassan	31.5 (26.6-36.9)	20.3 (17.0-24.2)	15.5 (10.8-21.0)	-4.28 (-5.96 to -2.57)	-3.90 (-8.01 to 0.25)	-4.13 (-6.01 to -2.50)
Haveri	40.9 (34.7-47.7)	29.0 (24.3-34.1)	21.8 (15.8-29.5)	-3.37 (-4.90 to -1.70)	-4.11 (-8.21 to 0.15)	-3.68 (-5.43 to -1.84)
Kalaburgi	30.4 (25.7-35.7)	25.7 (21.4-29.9)	19.5 (14.5-25.5)	-1.67 (-3.24 to -0.05)	-3.90 (-7.51 to -0.28)	-2.60 (-4.30 to -0.92)
Kodagu	20.9 (17.6-24.4)	13.8 (11.4-16.7)	10.6 (7.3-14.3)	-4.07 (-5.80 to -2.22)	-3.74 (-7.94 to 0.60)	-3.95 (-5.87 to -2.07)
Kolar	30.5 (25.9-35.4)	22.2 (18.8-26.3)	17.7 (12.8-23.8)	-3.08 (-4.62 to -1.53)	-3.29 (-7.17 to 0.94)	-3.18 (-5.03 to -1.40)
Koppal	50.0 (43.9-56.6)	37.9 (32.6-43.4)	27.9 (20.7-37.0)	-2.75 (-4.18 to -1.25)	-4.35 (-8.34 to -0.19)	-3.42 (-5.06 to -1.62)
Mandya	35.3 (30.3-40.6)	24.1 (20.5-28.4)	18.3 (13.1-24.4)	-3.77 (-5.31 to -2.18)	-3.98 (-8.02 to 0.13)	-3.86 (-5.66 to -2.14)
Mysuru	31.6 (27.0-36.5)	21.9 (18.4-25.9)	16.0 (11.4-21.5)	-3.58 (-5.27 to -1.88)	-4.50 (-8.63 to -0.48)	-3.97 (-5.84 to -2.26)
Raichur	42.9 (37.2-49.5)	34.4 (29.0-40.0)	26.5 (19.8-34.7)	-2.20 (-3.69 to -0.73)	-3.74 (-7.38 to 0.25)	-2.85 (-4.41 to -1.12)
Ramanagara	34.7 (29.5-40.3)	24.3 (20.7-28.5)	18.8 (13.6-25.1)	-3.50 (-4.97 to -1.95)	-3.69 (-7.81 to 0.44)	-3.59 (-5.38 to -1.80)
Shivamogga	28.5 (24.3-33.3)	19.7 (16.6-23.3)	14.7 (10.5-19.9)	-3.63 (-5.24 to -1.91)	-4.16 (-8.37 to 0.14)	-3.85 (-5.63 to -2.15)
Tumakuru	36.1 (31.4-41.4)	25.2 (21.8-29.6)	20.2 (14.7-26.5)	-3.55 (-5.04 to -1.96)	-3.16 (-7.13 to 1.00)	-3.39 (-5.13 to -1.75)
Udupi	17.8 (14.6-21.6)	12.0 (9.5-15.1)	9.1 (6.3-12.8)	-3.85 (-5.67 to -1.86)	-3.95 (-8.15 to 0.31)	-3.90 (-5.94 to -2.01)
Uttara Kannada	26.9 (22.4-31.8)	18.7 (15.5-22.6)	15.0 (10.7-20.5)	-3.57 (-5.23 to -1.71)	-3.20 (-7.62 to 1.07)	-3.43 (-5.18 to -1.63)
Vijaypura	33.1 (28.6-38.2)	25.4 (21.4-29.4)	19.2 (14.3-25.2)	-2.64 (-4.14 to -1.20)	-3.98 (-7.62 to -0.01)	-3.20 (-4.86 to -1.53)
Yadgir	37.5 (32.3-43.5)	30.0 (25.4-35.0)	23.9 (17.7-31.3)	-2.22 (-3.72 to -0.64)	-3.28 (-6.96 to 0.52)	-2.66 (-4.32 to -0.99)
**Telangana**	**34.4 (29.0-39.8)**	**25.2 (19.1-32.5)**	**17.6 (12.5-24.9)**	**-3.07 (-4.40 to -1.73)**	**-5.07 (-8.40 to -1.55)**	**-3.90 (-5.32 to -2.39)**
Adilabad	30.4 (24.9-36.0)	27.3 (22.3-32.7)	19.7 (14.4-26.4)	-1.09 (-2.73 to 0.69)	-4.61 (-8.05 to -0.91)	-2.56 (-4.13 to -0.97)
Bhadradri Kothagudem	38.9 (31.9-47.5)	29.3 (23.6-35.3)	22.1 (15.9-29.1)	-2.75 (-4.24 to -1.17)	-4.00 (-7.31 to -0.30)	-3.27 (-4.81 to -1.60)
Hyderabad	29.3 (24.7-34.2)	20.6 (17.1-24.6)	13.7 (10.1-18.3)	-3.43 (-5.02 to -1.76)	-5.70 (-9.57 to -1.93)	-4.38 (-6.00 to -2.69)
Jagitial	31.9 (26.9-37.5)	25.7 (21.6-30.6)	17.6 (13.0-23.2)	-2.14 (-3.72 to -0.55)	-5.31 (-8.65 to -1.75)	-3.46 (-5.02 to -1.89)
Jangoan	33.7 (28.3-39.4)	23.4 (19.4-27.7)	16.3 (12.0-21.9)	-3.59 (-5.16 to -2.11)	-5.07 (-8.60 to -1.49)	-4.20 (-5.76 to -2.63)
Jayashankar Bhupalpally	33.4 (26.9-41.2)	25.8 (20.4-32.0)	19.5 (14.1-26.1)	-2.58 (-4.30 to -0.93)	-3.99 (-7.40 to -0.19)	-3.17 (-4.84 to -1.52)
Jogulamba Gadwal	48.1 (40.6-56.3)	33.6 (28.0-39.9)	24.3 (18.0-32.3)	-3.50 (-5.02 to -1.96)	-4.64 (-8.38 to -0.80)	-3.98 (-5.63 to -2.29)
Kamareddy	34.3 (28.2-41.0)	26.2 (21.4-31.9)	17.8 (12.9-23.3)	-2.65 (-4.37 to -0.98)	-5.46 (-9.18 to -1.86)	-3.83 (-5.51 to -2.19)
Karimnagar	31.5 (26.4-37.4)	23.2 (19.3-28.0)	16.1 (11.8-21.2)	-3.00 (-4.63 to -1.45)	-5.19 (-8.45 to -1.56)	-3.92 (-5.43 to -2.33)
Khammam	34.9 (28.4-43.2)	26.3 (21.4-32.5)	18.7 (13.2-25.5)	-2.82 (-4.44 to -1.08)	-4.80 (-8.37 to -0.98)	-3.65 (-5.33 to -1.96)
Kumuram Bheem Asifabad	31.5 (25.5-38.4)	26.8 (21.5-33.3)	19.6 (14.2-26.7)	-1.62 (-3.34 to 0.15)	-4.44 (-7.83 to -0.81)	-2.79 (-4.35 to -1.11)
Mahabubnagar	43.2 (36.8-50.5)	28.8 (24.3-34.4)	21.2 (15.5-27.7)	-4.00 (-5.50 to -2.46)	-4.35 (-8.27 to -0.61)	-4.15 (-5.81 to -2.44)
Mahuabadad	33.1 (27.8-39.2)	24.6 (20.4-29.5)	17.7 (12.9-23.8)	-2.93 (-4.58 to -1.31)	-4.67 (-8.20 to -0.89)	-3.65 (-5.25 to -2.02)
Mancherial	31.8 (26.3-38.8)	26.0 (21.2-32.0)	18.4 (13.4-24.8)	-2.01 (-3.72 to -0.36)	-4.90 (-8.17 to -1.36)	-3.22 (-4.78 to -1.55)
Medak	37.0 (30.5-44.4)	26.6 (21.5-32.2)	17.9 (12.9-23.8)	-3.25 (-4.97 to -1.45)	-5.56 (-9.32 to -1.88)	-4.21 (-5.91 to -2.50)
Medchal Malkajgiri	31.0 (26.3-36.0)	21.3 (17.7-25.3)	14.1 (10.4-18.7)	-3.73 (-5.27 to -2.09)	-5.82 (-9.57 to -2.12)	-4.60 (-6.22 to -2.96)
Nagarkurnool	43.0 (35.0-52.7)	31.7 (25.1-39.6)	22.5 (16.0-30.7)	-3.01 (-4.71 to -1.30)	-4.89 (-8.78 to -0.93)	-3.79 (-5.57 to -2.10)
Nalgonda	35.6 (29.1-42.6)	26.4 (21.4-32.5)	18.2 (13.0-24.7)	-2.93 (-4.67 to -1.24)	-5.30 (-8.97 to -1.35)	-3.92 (-5.63 to -2.11)
Nirmal	30.5 (25.4-36.1)	26.9 (22.3-31.9)	18.9 (13.9-25.0)	-1.25 (-2.92 to 0.39)	-5.00 (-8.52 to -1.44)	-2.82 (-4.43 to -1.21)
Nizamabad	31.9 (26.8-37.5)	25.9 (21.6-30.3)	17.6 (13.0-23.0)	-2.08 (-3.74 to -0.44)	-5.46 (-8.99 to -1.86)	-3.49 (-5.07 to -1.93)
Peddapalli	31.7 (26.4-37.8)	24.3 (20.1-29.2)	16.8 (12.3-22.2)	-2.62 (-4.24 to -1.06)	-5.26 (-8.49 to -1.65)	-3.72 (-5.27 to -2.10)
Rajanna Sircilla	33.3 (27.8-39.5)	24.8 (20.5-30.0)	17.1 (12.6-22.6)	-2.90 (-4.54 to -1.28)	-5.24 (-8.75 to -1.50)	-3.88 (-5.45 to -2.23)
Rangareddy	35.3 (29.6-41.7)	23.7 (19.6-28.3)	16.4 (12.1-21.9)	-3.88 (-5.43 to -2.25)	-5.28 (-9.20 to -1.49)	-4.47 (-6.11 to -2.75)
Sangareddy	36.8 (31.3-42.8)	25.6 (21.5-30.1)	17.5 (13.0-22.6)	-3.56 (-5.12 to -1.97)	-5.37 (-9.03 to -1.86)	-4.31 (-5.94 to -2.75)
Siddipet	34.3 (29.0-39.8)	24.2 (20.2-28.9)	16.8 (12.4-22.0)	-3.43 (-4.99 to -1.87)	-5.16 (-8.71 to -1.39)	-4.15 (-5.73 to -2.56)
Suryapet	33.4 (27.8-39.5)	25.6 (20.9-30.9)	17.6 (12.9-23.9)	-2.62 (-4.29 to -0.93)	-5.29 (-8.87 to -1.53)	-3.73 (-5.39 to -1.95)
Vikarabad	40.3 (34.1-47.0)	26.5 (22.2-31.7)	19.3 (14.3-25.1)	-4.09 (-5.62 to -2.53)	-4.56 (-8.44 to -1.02)	-4.29 (-5.93 to -2.71)
Wanaparthy	45.4 (38.2-54.5)	32.4 (26.5-39.2)	23.1 (16.7-31.0)	-3.32 (-4.86 to -1.74)	-4.80 (-8.62 to -1.05)	-3.94 (-5.59 to -2.22)

Warangal Rural	31.8 (26.3-38.4)	23.5 (18.9-28.6)	16.6 (12.1-22.2)	-3.01 (-4.68 to -1.44)	-4.85 (-8.41 to -1.12)	-3.78 (-5.41 to -2.11)
Warangal Urban	30.1 (25.3-36.0)	21.9 (17.9-26.3)	14.9 (10.9-19.8)	-3.12 (-4.76 to -1.62)	-5.42 (-8.86 to -1.75)	-4.08 (-5.65 to -2.48)
Yadadri Bhuvanagiri	34.9 (28.8-41.0)	24.2 (19.9-29.2)	16.7 (12.2-22.6)	-3.59 (-5.16 to -2.04)	-5.24 (-8.96 to -1.48)	-4.28 (-5.86 to -2.57)
**Gujarat**	**36.5 (32.2-41.0)**	**28.0 (23.7-32.5)**	**23.1 (19.9-27.1)**	**-2.62 (-3.70 to -1.56)**	**-2.76 (-5.81 to 0.29)**	**-2.68 (-3.92 to -1.41)**
Ahmedabad	35.7 (31.4-40.9)	28.8 (24.7-33.3)	22.0 (16.8-28.2)	-2.15 (-3.61 to -0.77)	-3.86 (-7.22 to -0.60)	-2.86 (-4.34 to -1.44)
Amreli	29.1 (23.9-35.1)	20.5 (16.1-25.4)	18.7 (13.6-25.5)	-3.41 (-5.34 to -1.55)	-1.34 (-5.48 to 2.73)	-2.57 (-4.31 to -0.74)
Anand	47.2 (40.7-55.0)	37.2 (31.2-43.8)	29.3 (22.0-37.4)	-2.37 (-3.78 to -0.97)	-3.40 (-6.85 to -0.02)	-2.80 (-4.27 to -1.34)
Arvalli	47.7 (42.6-53.5)	39.3 (34.2-44.6)	28.5 (22.2-36.5)	-1.94 (-3.10 to -0.77)	-4.53 (-7.69 to -1.43)	-3.02 (-4.38 to -1.58)
Banaskantha	45.5 (39.8-52.0)	38.5 (33.5-44.0)	31.1 (24.3-39.1)	-1.67 (-2.98 to -0.33)	-3.04 (-6.26 to 0.15)	-2.24 (-3.62 to -0.87)
Bharuch	37.5 (32.4-42.9)	28.1 (23.6-32.7)	24.1 (18.2-30.9)	-2.82 (-4.26 to -1.36)	-2.22 (-5.76 to 1.36)	-2.58 (-4.08 to -1.02)
Bhavnagar	32.4 (28.0-37.7)	24.6 (20.3-29.5)	22.5 (16.9-29.6)	-2.72 (-4.34 to -1.18)	-1.35 (-5.07 to 2.39)	-2.17 (-3.71 to -0.61)
Botad	34.7 (29.4-40.4)	25.6 (21.2-30.3)	23.4 (17.5-30.1)	-3.02 (-4.60 to -1.49)	-1.36 (-5.01 to 2.19)	-2.34 (-3.81 to -0.76)
Chhotaudepur	44.1 (38.8-49.6)	34.6 (30.0-39.4)	30.5 (23.2-38.4)	-2.39 (-3.66 to -1.09)	-1.84 (-5.30 to 1.72)	-2.17 (-3.64 to -0.68)
Dahod	47.4 (41.5-54.0)	36.7 (32.1-41.9)	29.7 (22.6-37.8)	-2.52 (-3.84 to -1.27)	-2.96 (-6.29 to 0.43)	-2.71 (-4.28 to -1.26)
Dang	31.6 (27.5-36.0)	24.1 (20.8-27.9)	23.1 (17.5-29.7)	-2.66 (-4.13 to -1.23)	-0.55 (-4.11 to 3.17)	-1.80 (-3.31 to -0.30)
Devbhumi Dwarka	29.5 (22.8-37.8)	19.2 (14.4-25.0)	17.6 (12.0-25.4)	-4.18 (-6.55 to -1.80)	-1.36 (-5.41 to 2.83)	-3.04 (-4.97 to -0.97)
Gandhinagar	36.7 (31.5-42.8)	30.0 (24.9-35.5)	21.4 (15.9-28.4)	-1.98 (-3.54 to -0.50)	-4.75 (-8.15 to -1.50)	-3.14 (-4.70 to -1.66)
Gir Somnath	28.7 (23.5-35.2)	19.1 (14.9-23.8)	17.3 (12.4-24.4)	-4.01 (-6.00 to -2.00)	-1.45 (-5.74 to 2.84)	-2.97 (-4.74 to -0.93)
Jamnagar	29.5 (24.3-35.7)	19.7 (15.7-24.3)	17.4 (12.6-23.5)	-3.95 (-5.80 to -2.06)	-1.80 (-5.49 to 2.04)	-3.08 (-4.85 to -1.28)
Junagadh	28.6 (23.6-34.2)	18.1 (14.6-22.4)	15.4 (11.3-21.8)	-4.45 (-6.53 to -2.41)	-2.37 (-6.50 to 1.68)	-3.61 (-5.34 to -1.69)
Kachchh	35.7 (29.2-43.5)	25.9 (21.0-32.0)	25.0 (18.6-33.1)	-3.22 (-4.97 to -1.51)	-0.54 (-4.00 to 3.23)	-2.13 (-3.74 to -0.41)
Kheda	44.3 (39.2-50.0)	35.9 (31.0-41.0)	27.5 (21.2-35.3)	-2.07 (-3.39 to -0.77)	-3.78 (-7.16 to -0.61)	-2.79 (-4.24 to -1.38)
Mahesana	39.2 (34.7-44.7)	31.2 (26.9-36.0)	23.7 (18.2-30.4)	-2.25 (-3.62 to -0.96)	-3.92 (-7.21 to -0.86)	-2.95 (-4.42 to -1.53)
Mahisagar	46.5 (41.6-52.2)	37.1 (32.6-41.7)	27.9 (22.0-35.2)	-2.27 (-3.43 to -1.14)	-4.01 (-7.08 to -0.70)	-2.99 (-4.34 to -1.58)
Morbi	32.3 (26.1-39.8)	23.2 (18.3-28.9)	21.3 (15.4-28.5)	-3.28 (-5.15 to -1.56)	-1.25 (-4.80 to 2.45)	-2.46 (-4.11 to -0.87)
Narmada	42.7 (36.6-49.4)	32.6 (27.6-38.1)	29.0 (21.6-37.5)	-2.66 (-4.14 to -1.20)	-1.74 (-5.27 to 1.93)	-2.29 (-3.85 to -0.70)
Navsari	26.2 (22.2-30.3)	18.6 (15.6-22.0)	16.6 (12.3-21.9)	-3.36 (-4.97 to -1.81)	-1.67 (-5.48 to 2.33)	-2.68 (-4.32 to -1.08)
Panchmahal	45.7 (40.8-51.2)	36.2 (31.8-40.9)	28.1 (21.8-35.3)	-2.31 (-3.50 to -1.15)	-3.61 (-6.79 to -0.35)	-2.85 (-4.30 to -1.44)
Patan	41.8 (36.1-48.5)	33.6 (28.5-39.6)	28.2 (21.6-35.7)	-2.17 (-3.62 to -0.76)	-2.49 (-5.96 to 0.75)	-2.30 (-3.78 to -0.86)
Porbandar	28.5 (22.7-35.0)	18.4 (14.3-23.2)	15.6 (11.0-21.9)	-4.33 (-6.60 to -1.93)	-2.44 (-6.42 to 1.63)	-3.56 (-5.39 to -1.44)
Rajkot	28.0 (23.4-33.2)	18.8 (15.2-23.0)	16.3 (12.1-21.6)	-3.91 (-5.73 to -2.15)	-2.07 (-5.87 to 1.70)	-3.17 (-4.81 to -1.50)
Sabar Kantha	45.5 (40.5-51.4)	38.4 (33.5-43.7)	27.5 (21.4-35.3)	-1.68 (-2.86 to -0.49)	-4.68 (-7.84 to -1.65)	-2.94 (-4.35 to -1.53)
Surat	27.2 (22.9-32.0)	19.8 (16.4-23.8)	17.3 (12.6-23.2)	-3.17 (-4.78 to -1.58)	-1.97 (-5.79 to 2.03)	-2.68 (-4.31 to -1.01)
Surendranagar	35.3 (29.5-42.1)	26.7 (22.0-32.1)	23.6 (17.4-30.3)	-2.74 (-4.31 to -1.17)	-1.82 (-5.27 to 1.56)	-2.37 (-3.85 to -0.85)
Tapi	34.4 (30.4-38.8)	25.6 (22.2-29.3)	23.9 (18.1-30.8)	-2.91 (-4.36 to -1.53)	-1.07 (-4.58 to 2.64)	-2.16 (-3.68 to -0.62)
Vadodara	43.7 (38.0-49.8)	33.8 (28.8-38.8)	26.7 (20.3-34.1)	-2.56 (-3.86 to -1.24)	-3.34 (-6.66 to 0.05)	-2.89 (-4.40 to -1.45)
Valsad	25.4 (21.5-29.7)	18.6 (15.6-22.2)	17.2 (12.6-22.7)	-3.10 (-4.77 to -1.44)	-1.18 (-5.06 to 2.90)	-2.32 (-4.02 to -0.60)
**Manipur**	**21.9 (18.5-25.5)**	**16.5 (13.8-19.7)**	**13.8 (12.0-16.0)**	**-2.79 (-4.05 to -1.46)**	**-2.62 (-6.02 to 0.62)**	**-2.72 (-4.21 to -1.24)**
Bishnupur	20.8 (18.0-24.1)	16.0 (13.8-18.6)	13.3 (9.9-17.2)	-2.57 (-4.02 to -1.14)	-2.75 (-6.29 to 0.74)	-2.65 (-4.26 to -1.07)
Chandel	19.6 (16.8-22.8)	16.9 (14.3-19.5)	14.6 (10.9-18.9)	-1.51 (-3.01 to 0.06)	-2.19 (-5.76 to 1.48)	-1.79 (-3.41 to -0.13)
Churachandpur	22.7 (19.8-25.9)	18.2 (15.7-20.7)	15.0 (11.5-19.3)	-2.19 (-3.49 to -0.87)	-2.75 (-6.20 to 0.72)	-2.43 (-3.98 to -0.85)
Imphal East	20.2 (17.7-23.0)	15.0 (13.1-17.2)	12.4 (9.5-15.9)	-2.87 (-4.15 to -1.46)	-2.92 (-6.34 to 0.34)	-2.90 (-4.40 to -1.39)
Imphal West	19.7 (17.2-22.5)	15.0 (13.1-17.2)	11.9 (9.0-15.3)	-2.70 (-3.99 to -1.29)	-3.34 (-6.76 to -0.11)	-2.97 (-4.49 to -1.47)
Jiribam	42.5 (37.6-47.7)	31.6 (28.0-35.7)	24.8 (19.2-31.1)	-2.88 (-4.02 to -1.80)	-3.55 (-6.80 to -0.35)	-3.16 (-4.58 to -1.76)
Kakching	19.0 (16.3-22.2)	15.0 (12.8-17.5)	12.7 (9.4-16.7)	-2.39 (-3.92 to -0.84)	-2.42 (-6.10 to 1.22)	-2.41 (-4.06 to -0.73)
Kamjong	20.0 (17.0-23.7)	15.0 (12.7-17.7)	13.9 (10.4-18.2)	-2.84 (-4.24 to -1.35)	-1.19 (-4.79 to 2.40)	-2.17 (-3.71 to -0.60)
Kangpokpi	20.7 (18.1-23.5)	15.2 (13.3-17.4)	12.9 (9.9-16.5)	-3.13 (-4.38 to -1.75)	-2.27 (-5.71 to 1.03)	-2.78 (-4.28 to -1.29)
Noney	31.0 (27.5-35.2)	23.6 (20.7-26.9)	19.3 (14.8-24.6)	-2.68 (-3.86 to -1.47)	-2.94 (-6.22 to 0.29)	-2.79 (-4.28 to -1.33)
Pherzawl	30.3 (27.1-33.9)	22.8 (20.1-25.5)	18.9 (14.7-24.2)	-2.95 (-4.06 to -1.83)	-2.74 (-6.08 to 0.66)	-2.86 (-4.28 to -1.45)
Senapati	24.7 (21.9-27.9)	17.4 (15.4-19.6)	15.0 (11.7-18.8)	-3.50 (-4.61 to -2.18)	-2.15 (-5.41 to 1.02)	-2.95 (-4.38 to -1.60)
Tamenglong	32.4 (28.9-36.3)	23.2 (20.6-26.1)	19.3 (15.1-24.3)	-3.30 (-4.36 to -2.13)	-2.65 (-5.94 to 0.35)	-3.03 (-4.42 to -1.71)
Tengnoupal	20.0 (17.0-23.4)	15.7 (13.3-18.5)	14.0 (10.4-18.5)	-2.38 (-3.86 to -0.80)	-1.78 (-5.36 to 1.87)	-2.14 (-3.73 to -0.51)
Thoubal	19.2 (16.5-22.1)	14.6 (12.5-17.0)	12.2 (9.1-15.9)	-2.68 (-4.11 to -1.19)	-2.59 (-6.17 to 0.85)	-2.65 (-4.23 to -1.03)
Ukhrul	20.2 (17.6-23.2)	14.4 (12.6-16.5)	13.4 (10.3-17.1)	-3.29 (-4.67 to -1.89)	-1.09 (-4.43 to 2.33)	-2.40 (-3.87 to -0.93)
**Jammu & Kashmir**[†]	**27.8 (24.5-31.6)**	**24.0 (20.4-27.7)**	**19.7 (17.0-23.0)**	**-1.46 (-2.77 to -0.08)**	**-2.85 (-5.72 to -0.03)**	**-2.04 (-3.37 to -0.80)**
Anantnag	28.7 (25.2-33.0)	25.7 (22.3-29.4)	20.6 (16.1-25.7)	-1.10 (-2.59 to 0.39)	-3.19 (-6.24 to -0.17)	-1.97 (-3.39 to -0.70)
Badgam	29.1 (25.4-33.0)	25.3 (22.0-29.0)	19.7 (15.5-24.6)	-1.40 (-2.88 to 0.10)	-3.59 (-6.72 to -0.52)	-2.31 (-3.72 to -0.96)
Bandipore	32.7 (28.1-37.6)	28.2 (24.2-32.8)	22.4 (17.3-28.3)	-1.47 (-3.08 to 0.17)	-3.27 (-6.58 to -0.06)	-2.22 (-3.77 to -0.73)
Baramulla	34.9 (31.2-38.8)	31.3 (28.1-35.0)	24.6 (20.9-28.9)	-1.49 (-2.97 to 0.15)	-3.69 (-6.89 to -0.51)	-2.41 (-3.91 to -1.02)
Doda	23.6 (20.1-27.4)	23.1 (19.6-27.2)	19.5 (15.0-24.8)	-0.22 (-1.63 to 1.29)	-2.43 (-5.66 to 0.66)	-1.14 (-2.63 to 0.26)
Ganderbal	32.9 (28.2-37.9)	28.5 (24.6-33.0)	22.8 (17.7-28.9)	-1.40 (-2.98 to 0.25)	-3.21 (-6.49 to 0.02)	-2.15 (-3.64 to -0.71)
Jammu	27.2 (24.4-30.2)	23.6 (20.9-26.4)	20.7 (17-24.6)	-2.56 (-3.82 to -1.17)	-1.81 (-4.96 to 1.37)	-2.25 (-3.67 to -0.87)
Kargil	36.8 (31.9-42.7)	33.1 (28.6-38.2)	27.0 (21.4-33.5)	-1.18 (-2.79 to 0.43)	-2.67 (-5.96 to 0.62)	-1.80 (-3.27 to -0.40)
Kathua	26.6 (23.4-30.0)	22.2 (19.7-25.4)	18.9 (15.2-23.4)	-1.95 (-3.17 to -0.61)	-2.37 (-5.46 to 0.65)	-2.13 (-3.46 to -0.80)
Kishtwar	28.2 (24.3-32.9)	26.7 (22.8-31.0)	22.6 (17.5-28.7)	-0.56 (-2.04 to 0.89)	-2.37 (-5.48 to 0.70)	-1.31 (-2.76 to 0.02)
Kulgam	25.8 (22.8-29.3)	23.2 (20.3-26.6)	18.5 (14.6-23)	-1.02 (-2.39 to 0.37)	-3.22 (-6.17 to -0.31)	-1.94 (-3.30 to -0.66)
Kupwara	33.7 (29.6-38.2)	29.6 (26.2-33.7)	22.9 (18.7-27.7)	-1.62 (-3.17 to 0.00)	-3.44 (-6.76 to -0.22)	-2.38 (-3.96 to -1.00)
Leh	27.6 (22.0-34.5)	22.9 (18.2-29.0)	19.3 (14.1-25.9)	-1.90 (-3.77 to 0.02)	-2.40 (-5.95 to 1.02)	-2.11 (-3.75 to -0.34)
PoJK	40.7 (34.6-46.7)	40.5 (34.6-46.5)	30.1 (24.5-36.5)	-0.05 (-3.17 to 0.00)	-4.15 (-7.15 to 0.77)	-1.76 (-4.96 to 1.37)
Poonch	29.2 (26.3-32.5)	25.3 (22.7-28.1)	21.8 (18.4-25.4)	-2.16 (-3.60 to -0.74)	-1.87 (-4.99 to 1.22)	-2.05 (-3.41 to -0.74)
Pulwama	28.8 (25.2-32.9)	25.3 (21.9-29.0)	20.1 (15.8-25)	-1.28 (-2.76 to 0.25)	-3.29 (-6.35 to -0.27)	-2.11 (-3.52 to -0.80)
Rajouri	25.6 (22.6-28.7)	22.1 (19.8-24.8)	19.6 (16.4-23.4)	-2.24 (-3.61 to -0.88)	-1.24 (-4.41 to 1.78)	-1.83 (-3.19 to -0.49)
Ramban	23.2 (20.0-26.6)	21.9 (18.8-25.3)	18.4 (14.4-23.3)	-0.57 (-2.00 to 0.89)	-2.50 (-5.45 to 0.46)	-1.38 (-2.76 to -0.08)
Reasi	22.8 (20.0-26.0)	20.0 (17.6-23.0)	17.1 (13.4-21.4)	-1.28 (-2.59 to 0.01)	-2.30 (-5.36 to 0.59)	-1.71 (-3.02 to -0.42)
Samba	28.5 (25.4-31.9)	24.5 (21.9-27.4)	20.9 (17.1-25.3)	-2.22 (-3.62 to -0.72)	-2.35 (-5.54 to 0.80)	-2.28 (-3.78 to -0.84)
Shopian	27.2 (24.1-30.8)	24.1 (21.0-27.5)	19.1 (15-23.8)	-1.19 (-2.62 to 0.18)	-3.33 (-6.32 to -0.43)	-2.08 (-3.42 to -0.83)
Srinagar	29.8 (25.7-34.2)	25.4 (21.7-29.4)	19.2 (14.8-24.4)	-1.61 (-3.15 to 0.00)	-3.97 (-7.15 to -0.77)	-2.59 (-4.08 to -1.18)
Udhampur	23.5 (20.4-26.7)	20.9 (18.3-24.2)	17.5 (13.6-22)	-1.15 (-2.45 to 0.21)	-2.57 (-5.69 to 0.37)	-1.74 (-3.16 to -0.38)
**Haryana**	**30.1 (26.2-34.3)**	**25.6 (21.7-30.5)**	**20.0 (17.2-23.5)**	**-1.60 (-2.41 to -0.77)**	**-3.52 (-6.18 to -0.76)**	**-2.40 (-3.53 to -1.33)**
Ambala	26.5 (23.6-29.5)	21.7 (19.1-24.5)	16.4 (13.0-20.3)	-1.91 (-3.00 to -0.82)	-3.89 (-6.66 to -0.88)	-2.73 (-3.98 to -1.46)
Bhiwani	30.2 (26.8-33.7)	24.7 (21.8-27.5)	19.1 (14.9-23.8)	-2.00 (-3.04 to -0.94)	-3.64 (-6.38 to -0.69)	-2.68 (-3.99 to -1.42)
Charkhi Dadri	30.3 (26.6-34.7)	24.9 (21.6-28.4)	19.1 (14.7-24.2)	-1.96 (-3.15 to -0.76)	-3.77 (-6.67 to -0.64)	-2.71 (-4.11 to -1.39)
Faridabad	28.1 (25.4-30.9)	26.8 (23.9-29.6)	20.5 (16.4-25.4)	-0.44 (-1.40 to 0.56)	-3.56 (-6.43 to -0.67)	-1.74 (-2.99 to -0.58)
Fatehabad	30.9 (27.1-34.9)	24.9 (21.7-28.2)	20.5 (16.0-25.5)	-2.16 (-3.26 to -0.96)	-2.77 (-5.69 to 0.40)	-2.41 (-3.77 to -1.13)
Gurugram	28.2 (25.5-30.9)	25.9 (23.2-28.6)	20.4 (16.5-25.3)	-0.88 (-1.83 to 0.10)	-3.36 (-6.25 to -0.42)	-1.91 (-3.18 to -0.75)
Hisar	30.6 (27.1-33.9)	25.0 (22.2-27.8)	19.9 (15.7-24.6)	-2.01 (-2.98 to -0.90)	-3.18 (-6.01 to -0.15)	-2.49 (-3.74 to -1.29)
Jhajjar	30.2 (26.8-33.8)	25.1 (22.1-28.2)	20.0 (15.7-25.0)	-1.84 (-2.88 to -0.73)	-3.30 (-6.15 to -0.34)	-2.44 (-3.80 to -1.19)
Jind	33.0 (29.3-36.9)	26.9 (23.8-30.3)	20.9 (16.4-25.9)	-2.02 (-3.07 to -0.94)	-3.60 (-6.49 to -0.71)	-2.67 (-3.98 to -1.42)

Kaithal	32.5 (28.4-36.6)	25.6 (22.1-29.5)	19.7 (15.1-24.6)	-2.36 (-3.50 to -1.22)	-3.74 (-6.73 to -0.81)	-2.93 (-4.30 to -1.66)
Karnal	32.7 (29.2-36.1)	27.0 (23.9-30.6)	20.3 (16.0-25.3)	-1.90 (-2.90 to -0.87)	-4.09 (-6.96 to -1.27)	-2.81 (-4.08 to -1.63)
Kurukshetra	29.4 (26.3-32.6)	23.8 (20.8-26.9)	18.2 (14.4-22.5)	-2.03 (-3.06 to -1.00)	-3.84 (-6.68 to -0.86)	-2.78 (-4.00 to -1.55)
Mahendragarh	26.1 (23.2-29.1)	21.5 (18.9-24.0)	16.5 (12.9-20.6)	-1.91 (-2.97 to -0.81)	-3.87 (-6.77 to -1.03)	-2.73 (-4.05 to -1.47)
Nuh	34.2 (30.7-37.9)	33.9 (30.2-37.8)	28.5 (22.5-35.6)	-0.08 (-1.18 to 0.97)	-2.55 (-5.55 to 0.43)	-1.11 (-2.42 to 0.15)
Palwal	34.4 (30.8-38.2)	32.9 (29.3-36.6)	27.1 (21.6-33.7)	-0.49 (-1.57 to 0.52)	-2.93 (-5.89 to -0.01)	-1.51 (-2.78 to -0.28)
Panchkula	24.6 (21.6-28.0)	20.8 (18.0-23.9)	16.2 (12.7-20.3)	-1.58 (-2.84 to -0.32)	-3.90 (-6.78 to -0.80)	-2.54 (-3.86 to -1.25)
Panipat	31.5 (28.2-34.7)	26.2 (23.3-29.3)	19.4 (15.4-24.1)	-1.80 (-2.77 to -0.86)	-4.29 (-7.10 to -1.52)	-2.84 (-4.04 to -1.62)
Rewari	28.8 (25.9-32.1)	25.4 (22.5-28.3)	19.7 (15.5-24.6)	-1.23 (-2.22 to -0.17)	-3.70 (-6.55 to -0.77)	-2.26 (-3.57 to -1.00)
Rohtak	31.2 (27.8-34.8)	25.6 (22.6-28.7)	20.0 (15.8-25.2)	-1.96 (-3.02 to -0.88)	-3.52 (-6.40 to -0.53)	-2.61 (-3.93 to -1.40)
Sirsa	26.9 (23.5-30.3)	21.3 (18.8-24.1)	18.3 (14.2-23.0)	-2.33 (-3.46 to -1.12)	-2.18 (-5.13 to 1.04)	-2.27 (-3.59 to -0.94)
Sonipat	32.0 (28.9-35.3)	26.4 (23.7-29.3)	20.3 (16.1-24.9)	-1.92 (-2.86 to -0.93)	-3.77 (-6.59 to -0.92)	-2.69 (-3.91 to -1.53)
Yamunanagar	29.6 (26.4-33.0)	25.7 (22.6-28.9)	19.1 (15.1-23.9)	-1.40 (-2.53 to -0.27)	-4.29 (-7.12 to -1.32)	-2.61 (-3.84 to -1.33)
**Uttarakhand**	**27.0 (23.4-30.6)**	**22.0 (17.8-26.4)**	**17.1 (14.8-20.0)**	**-2.00 (-2.89 to -1.05)**	**-3.64 (-6.25 to -0.82)**	**-2.68 (-3.79 to -1.57)**
Almora	22.9 (20.5-25.3)	16.7 (14.7-18.8)	12.9 (10.3-16.1)	-3.02 (-4.11 to -1.97)	-3.64 (-6.50 to -0.61)	-3.28 (-4.51 to -2.05)
Bageshwar	23.9 (21.2-26.8)	17.8 (15.4-20.3)	13.4 (10.4-17.0)	-2.89 (-4.14 to -1.62)	-4.02 (-6.88 to -0.77)	-3.36 (-4.68 to -1.90)
Chamoli	20.6 (17.4-23.9)	16.3 (13.8-19.3)	12.4 (9.6-16.1)	-2.29 (-3.72 to -0.94)	-3.87 (-6.85 to -0.71)	-2.95 (-4.30 to -1.45)
Champawat	29.5 (26.4-32.9)	20.5 (18.0-23.0)	15.2 (12.0-19.2)	-3.58 (-4.65 to -2.49)	-4.27 (-7.23 to -1.24)	-3.87 (-5.17 to -2.57)
Dehradun	24.6 (22.0-27.5)	21.5 (18.9-24.2)	16.8 (13.5-20.7)	-1.36 (-2.45 to -0.23)	-3.48 (-6.34 to -0.46)	-2.24 (-3.50 to -0.97)
Haridwar	32.8 (29.5-36.4)	27.6 (24.4-31.0)	21.6 (17.2-26.6)	-1.74 (-2.82 to -0.73)	-3.44 (-6.22 to -0.43)	-2.45 (-3.62 to -1.29)
Nainital	24.9 (22.4-27.8)	19.5 (17.2-21.8)	15.1 (12.1-18.9)	-2.45 (-3.46 to -1.40)	-3.61 (-6.38 to -0.58)	-2.93 (-4.16 to -1.72)
Pauri Garhwal	24.3 (21.6-27.1)	20.3 (17.9-23.1)	15.7 (12.6-19.5)	-1.71 (-2.75 to -0.64)	-3.67 (-6.45 to -0.71)	-2.52 (-3.70 to -1.35)
Pithoragarh	26.0 (22.2-30.3)	18.7 (15.9-22.0)	13.6 (10.3-17.8)	-3.28 (-4.66 to -1.90)	-4.52 (-7.52 to -1.08)	-3.80 (-5.23 to -2.31)
Rudraprayag	21.1 (17.9-24.5)	18.0 (15.1-21.3)	13.8 (10.5-18.1)	-1.59 (-3.07 to -0.16)	-3.80 (-6.83 to -0.51)	-2.51 (-3.92 to -1.04)
Tehri Garhwal	23.2 (20.4-26.3)	20.2 (17.4-23.0)	15.6 (12.4-19.6)	-1.39 (-2.54 to -0.18)	-3.65 (-6.57 to -0.47)	-2.33 (-3.65 to -1.09)
Udham Singh Nagar	30.3 (27.4-33.6)	24.6 (21.8-27.4)	18.8 (15.1-23.3)	-2.17 (-3.14 to -1.17)	-3.82 (-6.53 to -0.89)	-2.86 (-4.08 to -1.69)
Uttarkashi	24.6 (21.5-28.2)	22.2 (19.0-25.7)	17.6 (13.5-22.3)	-0.97 (-2.37 to 0.41)	-3.35 (-6.46 to -0.25)	-1.96 (-3.40 to -0.56)
**Tamil Nadu**	**25.4 (22.6-28.6)**	**16.8 (13.7-20.4)**	**11.3 (9.7-13.3)**	**-4.08 (-5.41 to -2.89)**	**-5.52 (-9.37 to -1.80)**	**-4.68 (-6.33 to -2.96)**
Ariyalur	29.8 (25.4-34.4)	19.7 (16.6-23.4)	14.0 (10.0-19.0)	-4.05 (-5.64 to -2.20)	-4.91 (-9.22 to -0.72)	-4.41 (-6.33 to -2.46)
Chennai	17.9 (14.5-21.7)	11.2 (9.0-13.9)	8.2 (5.7-11.7)	-4.59 (-6.70 to -2.57)	-4.39 (-8.58 to 0.36)	-4.51 (-6.46 to -2.38)
Coimbatore	20.9 (17.7-24.5)	13.9 (11.6-16.7)	8.5 (6.1-11.8)	-4.01 (-5.65 to -2.27)	-6.84 (-10.93 to -2.37)	-5.19 (-6.98 to -3.39)
Cuddalore	28.2 (24.2-32.3)	17.9 (15.4-20.9)	12.6 (9.3-17.2)	-4.47 (-6.11 to -2.82)	-4.98 (-9.15 to -0.75)	-4.69 (-6.52 to -2.82)
Dharmapuri	30.2 (26.3-34.6)	21.0 (18.0-24.4)	13.3 (9.7-17.6)	-3.56 (-4.92 to -2.17)	-6.46 (-10.23 to -2.57)	-4.77 (-6.48 to -2.99)
Dindigul	27.0 (23.0-31.7)	18.5 (15.5-22.3)	11.9 (8.3-16.4)	-3.71 (-5.34 to -2.16)	-6.25 (-10.49 to -1.82)	-4.77 (-6.62 to -2.96)
Erode	29.2 (25.0-33.9)	20.2 (17.0-23.7)	12.4 (9.0-16.9)	-3.67 (-5.24 to -2.18)	-6.75 (-10.78 to -2.55)	-4.96 (-6.68 to -3.26)
Kanchipuram	22.6 (18.7-27.1)	13.4 (10.9-16.3)	10.3 (7.2-14.5)	-5.10 (-7.01 to -3.21)	-3.83 (-8.16 to 0.87)	-4.59 (-6.53 to -2.55)
Kanniyakumari	15.4 (11.7-20.2)	10.2 (7.6-13.2)	6.2 (3.8-9.3)	-4.05 (-6.40 to -1.61)	-6.90 (-11.51 to -2.00)	-5.24 (-7.61 to -2.91)
Karur	26.4 (22.5-30.8)	18.2 (15.2-21.4)	11.8 (8.4-16.1)	-3.69 (-5.20 to -2.11)	-6.16 (-10.37 to -1.91)	-4.72 (-6.50 to -2.92)
Krishnagiri	33.2 (28.6-38.2)	22.5 (19.2-26.4)	14.3 (10.4-18.9)	-3.84 (-5.31 to -2.43)	-6.37 (-10.17 to -2.44)	-4.89 (-6.71 to -3.19)
Madurai	28.9 (24.3-34.3)	19.6 (16.2-23.7)	12.9 (8.8-17.8)	-3.80 (-5.52 to -2.10)	-5.87 (-10.21 to -1.48)	-4.67 (-6.71 to -2.80)
Nagapattinam	25.8 (21.5-30.5)	16.3 (13.5-19.9)	11.7 (8.2-16.3)	-4.48 (-6.29 to -2.59)	-4.77 (-9.11 to -0.34)	-4.61 (-6.63 to -2.58)
Namakkal	25.7 (21.8-30.1)	17.8 (14.6-21.2)	11.2 (8.0-15.4)	-3.56 (-5.14 to -1.91)	-6.53 (-10.60 to -2.37)	-4.80 (-6.56 to -2.96)
Perambalur	27.4 (23.7-31.6)	18.4 (15.6-21.6)	12.6 (9.0-17.0)	-3.87 (-5.39 to -2.23)	-5.39 (-9.65 to -1.15)	-4.51 (-6.37 to -2.65)
Pudukkottai	28.9 (24.7-33.6)	18.7 (15.7-22.0)	12.9 (9.2-17.5)	-4.30 (-5.93 to -2.74)	-5.21 (-9.40 to -0.93)	-4.69 (-6.67 to -2.78)
Ramanathapuram	23.3 (18.2-28.9)	14.9 (11.6-18.3)	10.3 (6.8-14.7)	-4.40 (-6.45 to -2.34)	-5.23 (-9.81 to -0.65)	-4.75 (-6.89 to -2.63)
Salem	27.6 (23.7-31.7)	19.3 (16.1-22.5)	12.1 (8.8-16.2)	-3.50 (-4.96 to -2.04)	-6.56 (-10.52 to -2.57)	-4.78 (-6.48 to -3.02)
Sivaganga	27.0 (22.6-32.3)	17.1 (14.1-20.3)	11.8 (8.2-16.2)	-4.47 (-6.28 to -2.71)	-5.33 (-9.78 to -0.75)	-4.83 (-6.84 to -2.89)
Thanjavur	28.2 (24.1-33.1)	18.4 (15.4-21.9)	12.9 (9.2-17.9)	-4.18 (-5.85 to -2.37)	-5.09 (-9.38 to -0.86)	-4.56 (-6.54 to -2.54)
The Nilgiris	23.5 (20.1-27.3)	15.5 (13.0-18.5)	9.6 (6.8-13.0)	-4.12 (-5.76 to -2.34)	-6.85 (-10.86 to -2.61)	-5.26 (-7.11 to -3.51)
Theni	28.1 (23.1-34.4)	19.1 (15.3-23.3)	12.5 (8.4-17.7)	-3.80 (-5.61 to -1.99)	-6.01 (-10.60 to -1.41)	-4.72 (-6.79 to -2.76)
Thiruvallur	21.1 (17.4-25.2)	12.9 (10.5-15.8)	9.8 (6.8-14.0)	-4.80 (-6.82 to -2.87)	-4.02 (-8.19 to 0.61)	-4.49 (-6.44 to -2.45)
Thiruvarur	27.1 (22.8-32.1)	17.4 (14.5-21.2)	12.5 (8.8-17.4)	-4.33 (-6.14 to -2.38)	-4.67 (-9.02 to -0.23)	-4.48 (-6.51 to -2.42)
Thoothukudi	22.7 (18.2-27.9)	15.7 (12.3-19.4)	10.6 (6.9-15.4)	-3.63 (-5.74 to -1.50)	-5.64 (-10.26 to -0.95)	-4.47 (-6.65 to -2.38)
Tiruchirappalli	27.4 (23.6-31.6)	18.6 (15.7-21.7)	12.3 (8.9-16.7)	-3.82 (-5.38 to -2.31)	-5.76 (-10.08 to -1.73)	-4.63 (-6.45 to -2.77)
Tirunelveli	20.6 (17.1-25.1)	14.2 (11.2-17.2)	9.3 (6.1-13.3)	-3.70 (-5.78 to -1.53)	-5.96 (-10.52 to -1.25)	-4.65 (-6.85 to -2.56)
Tiruppur	25.0 (20.7-29.9)	16.9 (13.8-20.5)	10.4 (7.3-14.5)	-3.88 (-5.51 to -2.24)	-6.75 (-10.84 to -2.38)	-5.08 (-6.86 to -3.26)
Tiruvannamalai	27.1 (23.0-31.6)	17.4 (14.6-20.9)	12.6 (9.1-17.0)	-4.28 (-5.87 to -2.73)	-4.71 (-8.65 to -0.42)	-4.47 (-6.31 to -2.68)
Vellore	27.0 (23.2-31.1)	17.7 (15.1-20.8)	12.5 (8.9-16.9)	-4.13 (-5.79 to -2.61)	-4.99 (-8.84 to -0.76)	-4.49 (-6.35 to -2.67)
Viluppuram	27.9 (24.2-32.0)	17.9 (15.4-21.0)	12.8 (9.5-17.1)	-4.32 (-5.81 to -2.80)	-4.89 (-8.82 to -0.66)	-4.56 (-6.31 to -2.73)
Virudhunagar	27.7 (23.2-33.1)	19.0 (15.5-22.8)	12.8 (8.7-17.8)	-3.71 (-5.55 to -1.85)	-5.61 (-9.96 to -0.99)	-4.50 (-6.60 to -2.52)
**Mizoram**	**19.9 (17.0-23.1)**	**21.1 (17.4-24.9)**	**18.1 (15.6-21.3)**	**0.59 (-0.62 to 1.82)**	**-2.20 (-5.62 to 1.25)**	**-0.57 (-2.08 to 0.84)**
Aizawl	19.3 (17.2-21.5)	19.8 (17.3-22.2)	17.4 (13.6-22.3)	0.23 (-0.93 to 1.45)	-1.94 (-5.32 to 1.53)	-0.67 (-2.08 to 0.83)
Champhai	15.9 (13.8-18.2)	18.6 (15.9-21.4)	15.8 (12.2-20.3)	1.60 (0.24 to 3.00)	-2.38 (-5.84 to 1.17)	-0.07 (-1.62 to 1.54)
Kolasib	21.8 (19.5-24.2)	21.3 (18.9-24.1)	18.3 (14.4-23.6)	-0.20 (-1.32 to 0.98)	-2.23 (-5.64 to 1.19)	-1.05 (-2.45 to 0.40)
Lawngtlai	21.2 (18.4-24.4)	23.7 (20.5-27.5)	20.5 (15.5-26.5)	1.23 (-0.27 to 2.77)	-2.11 (-5.84 to 1.52)	-0.17 (-1.86 to 1.43)
Lunglei	20.0 (17.4-22.9)	21.7 (18.8-25.0)	18.1 (13.8-23.4)	0.83 (-0.53 to 2.30)	-2.57 (-6.23 to 1.00)	-0.59 (-2.18 to 0.87)
Mamit	24.8 (22.0-28.0)	24.1 (21.1-27.4)	20.9 (16.5-27.1)	-0.24 (-1.45 to 1.03)	-2.13 (-5.59 to 1.39)	-1.03 (-2.52 to 0.42)
Saiha	21.3 (18.0-25.2)	25.8 (21.7-30.3)	22.1 (16.6-28.8)	1.91 (0.32 to 3.63)	-2.35 (-6.09 to 1.24)	0.13 (-1.59 to 1.84)
Serchhip	18.3 (16.0-20.8)	19.6 (17.0-22.4)	16.7 (12.9-21.4)	0.70 (-0.61 to 2.07)	-2.36 (-5.93 to 1.26)	-0.58 (-2.12 to 0.92)
**Maharashtra**	**31.1 (27.5-34.8)**	**22.6 (18.6-27.0)**	**17.0 (14.7-20.0)**	**-3.14 (-4.12 to -2.10)**	**-4.00 (-7.02 to -0.98)**	**-3.50 (-4.75 to -2.27)**
Ahmednagar	29.6 (25.6-34.7)	21.4 (17.7-25.2)	16.0 (11.7-20.9)	-3.19 (-4.75 to -1.59)	-4.13 (-7.89 to -0.42)	-3.58 (-5.14 to -1.98)
Akola	33.2 (28.7-38.2)	25.8 (22.1-30.2)	18.9 (14.1-24.3)	-2.50 (-3.92 to -1.10)	-4.39 (-8.13 to -0.83)	-3.29 (-4.94 to -1.67)
Amravati	37.2 (31.7-43.5)	26.2 (22.1-31.1)	19.6 (14.5-25.5)	-3.43 (-4.85 to -2.09)	-4.18 (-7.79 to -0.85)	-3.74 (-5.34 to -2.21)
Aurangabad	31.0 (26.7-36.3)	23.8 (20.0-27.9)	17.6 (13.0-23.0)	-2.64 (-4.16 to -1.18)	-4.29 (-7.93 to -0.66)	-3.33 (-4.92 to -1.73)
Beed	29.8 (25.7-34.5)	22.2 (19.0-25.8)	17.0 (12.6-21.9)	-2.91 (-4.38 to -1.40)	-3.86 (-7.43 to -0.25)	-3.31 (-4.93 to -1.77)
Bhandara	43.8 (38.0-50.3)	28.8 (24.7-33.8)	22.6 (17.1-29.6)	-4.07 (-5.38 to -2.68)	-3.66 (-7.03 to -0.22)	-3.90 (-5.31 to -2.35)
Buldhana	34.3 (29.5-39.7)	27.4 (23.4-31.7)	19.9 (14.6-25.7)	-2.21 (-3.65 to -0.84)	-4.55 (-8.42 to -0.98)	-3.19 (-4.83 to -1.63)
Chandrapur	39.2 (33.6-45.2)	28.8 (24.1-34.2)	22.9 (17.3-29.9)	-3.04 (-4.46 to -1.46)	-3.29 (-6.61 to 0.20)	-3.15 (-4.66 to -1.51)
Dhule	35.8 (30.8-41.7)	27.3 (23.2-32.0)	19.8 (15.0-25.7)	-2.69 (-4.14 to -1.25)	-4.53 (-8.03 to -0.98)	-3.45 (-4.97 to -1.91)
Gadchiroli	44.6 (38.4-51.2)	33.3 (28.4-38.8)	27.2 (20.9-35.2)	-2.92 (-4.20 to -1.55)	-2.90 (-6.24 to 0.35)	-2.92 (-4.29 to -1.33)
Gondia	47.6 (41.8-54.0)	31.8 (27.9-36.8)	23.9 (18.3-31.1)	-3.96 (-5.31 to -2.66)	-4.06 (-7.35 to -0.59)	-4.01 (-5.39 to -2.49)
Hingoli	32.7 (28.0-37.5)	28.0 (23.9-32.6)	20.3 (15.1-26.5)	-1.54 (-3.04 to -0.08)	-4.61 (-8.38 to -0.94)	-2.82 (-4.52 to -1.21)
Jalgaon	37.8 (32.5-43.6)	28.8 (24.6-33.6)	20.4 (15.2-26.6)	-2.67 (-4.08 to -1.24)	-4.92 (-8.39 to -1.45)	-3.61 (-5.12 to -2.09)
Jalna	33.1 (28.5-38.3)	26.8 (22.8-30.9)	20.2 (14.9-26.2)	-2.09 (-3.53 to -0.73)	-4.05 (-7.67 to -0.44)	-2.91 (-4.58 to -1.34)
Kolhapur	30.0 (24.5-35.7)	17.4 (14.1-21.0)	12.5 (8.9-17.1)	-5.31 (-7.14 to -3.41)	-4.72 (-8.96 to -0.42)	-5.08 (-7.02 to -3.30)
Latur	30.5 (25.8-35.6)	21.2 (17.7-25.2)	15.6 (11.4-20.6)	-3.54 (-5.13 to -1.89)	-4.38 (-8.19 to -0.70)	-3.89 (-5.69 to -2.24)
Mumbai City	23.5 (19.2-28.3)	18.1 (14.3-22.4)	14.1 (9.6-19.4)	-2.60 (-4.69 to -0.38)	-3.58 (-7.89 to 0.84)	-3.01 (-4.89 to -1.11)
Mumbai Suburban	22.4 (18.6-26.5)	17.7 (14.3-21.7)	13.9 (9.8-19.1)	-2.34 (-4.37 to -0.13)	-3.49 (-7.76 to 0.82)	-2.82 (-4.68 to -0.93)

Nagpur	38.7 (33.5-44.1)	25.5 (22.0-29.5)	20.4 (15.4-26.7)	-4.06 (-5.46 to -2.67)	-3.27 (-6.78 to 0.11)	-3.74 (-5.27 to -2.17)
Nanded	34.3 (29.4-39.7)	27.2 (23.0-31.9)	20.0 (14.7-25.9)	-2.28 (-3.72 to -0.83)	-4.34 (-7.95 to -0.78)	-3.14 (-4.78 to -1.56)
Nandurbar	45.5 (39.8-51.2)	33.9 (29.6-38.7)	24.7 (18.7-31.5)	-2.87 (-4.19 to -1.54)	-4.58 (-7.94 to -1.12)	-3.59 (-5.07 to -2.07)
Nashik	31.2 (27.2-36.3)	23.1 (19.6-27.1)	17.2 (13.1-22.2)	-2.96 (-4.45 to -1.47)	-4.20 (-7.60 to -0.62)	-3.48 (-5.07 to -1.97)
Osmanabad	30.7 (26.5-35.2)	20.8 (17.6-24.1)	15.7 (11.4-20.6)	-3.84 (-5.41 to -2.35)	-3.98 (-7.78 to -0.38)	-3.91 (-5.71 to -2.32)
Palghar	28.4 (24.0-33.2)	21.3 (17.8-25.4)	17.3 (12.9-22.5)	-2.84 (-4.67 to -1.07)	-3.01 (-6.86 to 1.09)	-2.92 (-4.59 to -1.16)
Parbhani	34.4 (29.4-40.0)	28.8 (24.3-33.9)	20.7 (15.3-27.1)	-1.77 (-3.21 to -0.29)	-4.67 (-8.42 to -1.09)	-2.98 (-4.69 to -1.39)
Pune	26.1 (21.7-31.2)	17.9 (14.5-21.8)	13.5 (9.7-18.2)	-3.71 (-5.44 to -1.92)	-4.03 (-7.96 to 0.06)	-3.85 (-5.58 to -2.11)
Raigad	25.3 (21.2-30.0)	18.3 (15.0-22.3)	14.4 (10.2-19.3)	-3.17 (-5.09 to -1.14)	-3.53 (-7.72 to 0.88)	-3.33 (-5.17 to -1.49)
Ratnagiri	26.7 (20.9-33.2)	17.6 (13.6-22.4)	13.1 (8.9-18.8)	-4.07 (-6.09 to -1.97)	-4.24 (-8.69 to 0.13)	-4.15 (-6.03 to -2.20)
Sangli	30.9 (26.1-36.0)	18.2 (15.2-21.4)	13.2 (9.6-17.7)	-5.17 (-6.75 to -3.52)	-4.61 (-8.47 to -0.64)	-4.94 (-6.73 to -3.29)
Satara	26.4 (21.7-31.5)	17.4 (14.0-20.9)	12.7 (9.1-17.1)	-4.11 (-5.77 to -2.42)	-4.42 (-8.53 to -0.57)	-4.25 (-5.95 to -2.55)
Sindhudurg	28.1 (22.9-34.3)	16.3 (13.0-20.3)	12.8 (8.7-17.9)	-5.34 (-7.39 to -3.26)	-3.50 (-7.83 to 0.97)	-4.60 (-6.60 to -2.64)
Solapur	31.4 (26.9-36.5)	19.9 (16.8-23.5)	14.9 (10.7-19.7)	-4.46 (-5.97 to -2.94)	-4.17 (-8.06 to -0.32)	-4.35 (-6.07 to -2.72)
Thane	24.8 (21.1-29.2)	19.5 (16.1-23.7)	15.1 (11.0-20.2)	-2.38 (-4.30 to -0.38)	-3.66 (-7.69 to 0.48)	-2.92 (-4.69 to -1.15)
Wardha	37.2 (31.8-43.0)	25.7 (21.7-30.1)	20.8 (15.5-26.7)	-3.63 (-5.01 to -2.27)	-3.08 (-6.72 to 0.38)	-3.41 (-4.99 to -1.88)
Washim	31.3 (26.9-36.0)	26.1 (22.3-30.3)	19.0 (14.2-24.6)	-1.81 (-3.23 to -0.37)	-4.52 (-8.25 to -0.83)	-2.94 (-4.58 to -1.29)
Yavatmal	34.3 (29.2-39.5)	27.2 (23.0-31.9)	20.7 (15.4-26.9)	-2.30 (-3.68 to -0.86)	-3.87 (-7.37 to -0.44)	-2.95 (-4.56 to -1.38)
**Punjab**	**26.3 (23.2-29.7)**	**21.4 (18.2-25.0)**	**16.4 (14.1-19.2)**	**-2.04 (-2.99 to -1.09)**	**-3.78 (-6.50 to -0.98)**	**-2.76 (-3.95 to -1.63)**
Amritsar	25.3 (22.5-28.2)	19.5 (17.2-21.9)	16.3 (13.2-19.9)	-2.60 (-3.69 to -1.49)	-2.54 (-5.36 to 0.38)	-2.58 (-3.83 to -1.37)
Barnala	31.1 (27.7-34.6)	24.9 (22.0-28.0)	18.8 (14.9-23.5)	-2.17 (-3.27 to -1.12)	-3.99 (-6.82 to -1.05)	-2.93 (-4.21 to -1.64)
Bathinda	27.4 (24.5-30.5)	22.2 (19.7-25.0)	17.0 (13.4-21.1)	-2.08 (-3.11 to -0.99)	-3.83 (-6.67 to -0.81)	-2.81 (-4.07 to -1.55)
Faridkot	29.7 (26.5-33.4)	23.3 (20.4-26.2)	18.9 (15.0-23.6)	-2.40 (-3.50 to -1.26)	-3.01 (-6.03 to -0.02)	-2.65 (-3.96 to -1.38)
Fatehgarh Sahib	24.6 (21.8-27.6)	20.7 (18.2-23.6)	14.4 (11.4-17.8)	-1.75 (-2.86 to -0.60)	-5.15 (-7.84 to -2.21)	-3.17 (-4.42 to -1.98)
Fazilka	27.0 (24.2-30.2)	21.5 (19.1-24.0)	17.4 (13.9-21.6)	-2.20 (-3.28 to -1.17)	-3.04 (-5.94 to 0.07)	-2.55 (-3.82 to -1.23)
Ferozepur	33.0 (29.4-37.2)	26.2 (23.0-29.3)	21.3 (16.9-26.5)	-2.24 (-3.35 to -1.10)	-2.96 (-5.94 to -0.06)	-2.54 (-3.82 to -1.28)
Gurdaspur	22.9 (20.3-25.7)	18.3 (16.1-20.9)	15.2 (12.2-18.7)	-2.23 (-3.39 to -1.02)	-2.74 (-5.49 to 0.19)	-2.45 (-3.70 to -1.20)
Hoshiarpur	22.7 (20.2-25.6)	18.7 (16.3-21.4)	14.3 (11.3-17.8)	-1.90 (-3.09 to -0.66)	-3.78 (-6.61 to -0.86)	-2.68 (-3.99 to -1.47)
Jalandhar	25.0 (22.4-28.0)	20.5 (18.0-23.2)	15.9 (12.6-19.8)	-1.95 (-3.10 to -0.78)	-3.65 (-6.65 to -0.68)	-2.66 (-3.93 to -1.41)
Kapurthala	25.9 (23.2-29.1)	20.7 (18.2-23.3)	16.7 (13.2-20.7)	-2.25 (-3.40 to -1.10)	-3.08 (-6.08 to -0.08)	-2.59 (-3.89 to -1.33)
Ludhiana	25.8 (23.0-28.8)	22.4 (19.8-25.4)	15.7 (12.5-19.5)	-1.41 (-2.52 to -0.26)	-5.03 (-7.83 to -2.09)	-2.92 (-4.15 to -1.69)
Mansa	29.5 (25.9-33.1)	24.5 (21.5-27.8)	18.2 (14.3-22.5)	-1.84 (-2.93 to -0.64)	-4.23 (-7.08 to -1.12)	-2.84 (-4.15 to -1.59)
Moga	30.9 (27.0-35.2)	24.4 (21.0-28.0)	19.7 (15.4-24.9)	-2.35 (-3.63 to -1.05)	-3.07 (-6.25 to 0.04)	-2.65 (-4.07 to -1.30)
Pathankot	20.6 (18.1-23.4)	17.5 (15.2-20.1)	13.1 (10.3-16.4)	-1.52 (-2.73 to -0.21)	-4.10 (-6.97 to -1.22)	-2.59 (-3.91 to -1.31)
Patiala	25.7 (23.1-28.6)	21.3 (18.8-24.1)	14.6 (11.6-18.1)	-1.87 (-2.92 to -0.80)	-5.33 (-8.10 to -2.45)	-3.31 (-4.53 to -2.13)
Rupnagar	22.6 (20.1-25.3)	19.2 (16.7-21.8)	13.9 (11.0-17.3)	-1.57 (-2.74 to -0.28)	-4.68 (-7.58 to -1.75)	-2.86 (-4.17 to -1.61)
Sahibzada Ajit Singh Nagar	22.5 (19.6-25.5)	18.8 (16.2-21.7)	13.0 (10.1-16.3)	-1.76 (-3.01 to -0.46)	-5.23 (-8.10 to -2.21)	-3.21 (-4.53 to -1.94)
Sangrur	30.2 (27.0-33.4)	24.8 (21.9-27.9)	17.8 (14.1-22.1)	-1.97 (-3.02 to -0.95)	-4.63 (-7.43 to -1.73)	-3.08 (-4.30 to -1.85)
Shahid Bhagat Singh Nagar	24.2 (21.6-27.0)	20.5 (18.0-23.3)	15.1 (12.0-18.6)	-1.62 (-2.79 to -0.34)	-4.35 (-7.19 to -1.46)	-2.76 (-4.04 to -1.51)
Sri Mukhtar Sahib	26.3 (23.4-29.3)	20.8 (18.4-23.2)	16.7 (13.2-20.9)	-2.34 (-3.37 to -1.27)	-3.07 (-5.92 to -0.03)	-2.64 (-3.89 to -1.33)
Tarn Taran	29.6 (26.6-33.1)	22.3 (19.7-25.0)	19.5 (15.6-23.9)	-2.80 (-3.91 to -1.70)	-1.88 (-4.87 to 1.13)	-2.43 (-3.74 to -1.19)
**Sikkim**	**22.5 (18.7-26.8)**	**17.2 (13.2-21.4)**	**12.1 (10.5-14.0)**	**-2.66 (-4.00 to -1.32)**	**-4.99 (-7.98 to -1.73)**	**-3.63 (-4.99 to -2.18)**
East Sikkim	20.5 (17.7-23.6)	15.6 (13.4-18.2)	10.8 (8.3-13.8)	-2.73 (-4.08 to -1.38)	-5.13 (-8.16 to -1.72)	-3.73 (-5.16 to -2.24)
North Sikkim	26.5 (22.6-30.9)	20.1 (17.0-23.7)	14.4 (11.1-18.7)	-2.74 (-4.12 to -1.40)	-4.67 (-7.63 to -1.46)	-3.54 (-4.94 to -2.09)
South Sikkim	21.8 (18.9-24.9)	16.5 (14.3-19.2)	11.4 (8.9-14.6)	-2.72 (-4.04 to -1.32)	-5.23 (-8.31 to -1.91)	-3.76 (-5.12 to -2.31)
West Sikkim	24.9 (21.7-28.6)	19.3 (16.5-22.4)	13.7 (10.6-17.8)	-2.51 (-3.85 to -1.18)	-4.81 (-7.84 to -1.41)	-3.47 (-4.90 to -2.00)
**Nagaland**	**25.1 (20.4-30.7)**	**17.9 (14.0-22.5)**	**15.8 (13.7-18.6)**	**-3.30 (-4.51 to -2.01)**	**-1.84 (-5.13 to 1.26)**	**-2.71 (-4.09 to -1.36)**
Dimapur	29.8 (26.0-34.2)	20.4 (17.8-23.4)	17.3 (13.5-22.0)	-3.74 (-4.97 to -2.46)	-2.37 (-5.55 to 0.78)	-3.18 (-4.53 to -1.81)
Kiphire	17.0 (14.1-20.2)	12.6 (10.5-15.1)	12.8 (9.5-16.5)	-2.95 (-4.52 to -1.36)	0.18 (-3.41 to 3.75)	-1.68 (-3.25 to -0.05)
Kohima	22.1 (19.5-25.3)	15.4 (13.6-17.5)	13.6 (10.7-17.1)	-3.58 (-4.82 to -2.23)	-1.77 (-5.03 to 1.40)	-2.85 (-4.22 to -1.44)
Longleng	26.4 (23.3-29.9)	19.7 (17.3-22.4)	17.2 (13.3-21.6)	-2.87 (-4.20 to -1.59)	-1.91 (-5.27 to 1.41)	-2.48 (-3.94 to -1.09)
Mokokchung	28.1 (24.8-31.9)	19.8 (17.3-22.7)	16.5 (12.8-20.6)	-3.48 (-4.84 to -2.13)	-2.67 (-6.02 to 0.52)	-3.15 (-4.66 to -1.70)
Mon	27.0 (23.8-30.7)	20.7 (18.2-23.6)	18.3 (14.2-23.2)	-2.57 (-3.92 to -1.21)	-1.84 (-5.28 to 1.51)	-2.28 (-3.74 to -0.84)
Peren	29.4 (26.0-33.2)	21.0 (18.6-23.7)	18.2 (14.3-22.7)	-3.33 (-4.43 to -2.15)	-2.14 (-5.31 to 0.93)	-2.84 (-4.16 to -1.56)
Phek	17.4 (15.0-20.1)	12.6 (10.9-14.6)	12.2 (9.3-15.4)	-3.19 (-4.54 to -1.71)	-0.52 (-3.97 to 2.89)	-2.10 (-3.58 to -0.62)
Tuensang	19.6 (16.7-22.7)	14.8 (12.6-17.2)	14.3 (11.0-18.1)	-2.75 (-4.23 to -1.25)	-0.50 (-4.04 to 3.03)	-1.83 (-3.40 to -0.26)
Wokha	28.3 (25.0-32.3)	19.8 (17.4-22.4)	16.6 (13.0-20.9)	-3.51 (-4.83 to -2.18)	-2.59 (-5.87 to 0.62)	-3.14 (-4.62 to -1.74)
Zunheboto	20.5 (17.6-23.7)	14.7 (12.6-17.0)	13.5 (10.4-17.1)	-3.27 (-4.71 to -1.74)	-1.26 (-4.72 to 2.08)	-2.45 (-3.95 to -0.94)
**Himachal Pradesh**	**27.2 (24.0-30.6)**	**22.8 (19.0-26.9)**	**18.6 (16.1-21.7)**	**-1.78 (-3.07 to -0.50)**	**-2.92 (-5.81 to -0.12)**	**-2.25 (-3.61 to -1.03)**
Bilaspur	24.1 (21.1-27.4)	19.0 (16.3-22.0)	15.6 (12.3-19.4)	-2.34 (-3.64 to -0.95)	-2.86 (-5.95 to 0.12)	-2.56 (-3.92 to -1.22)
Chamba	27.7 (23.6-32.2)	24.6 (20.8-29.1)	20.8 (15.8-26.8)	-1.17 (-2.68 to 0.30)	-2.43 (-5.50 to 0.68)	-1.69 (-3.13 to -0.34)
Hamirpur	23.0 (19.9-26.6)	18.3 (15.4-21.7)	14.9 (11.5-18.9)	-2.25 (-3.72 to -0.72)	-2.99 (-6.16 to 0.12)	-2.56 (-3.98 to -1.19)
Kangra	26.7 (23.1-30.5)	22.4 (18.9-26.1)	18.5 (14.2-23.6)	-1.74 (-3.22 to -0.29)	-2.72 (-5.74 to 0.23)	-2.15 (-3.59 to -0.89)
Kinnaur	31.3 (26.3-37.5)	27.4 (22.8-32.8)	21.7 (16.2-28.3)	-1.33 (-3.05 to 0.32)	-3.35 (-6.73 to -0.07)	-2.17 (-3.80 to -0.59)
Kullu	31.8 (27.4-36.7)	27.9 (23.6-32.6)	22.5 (17.2-28.8)	-1.32 (-2.85 to 0.22)	-3.05 (-6.10 to -0.01)	-2.04 (-3.57 to -0.71)
Lahul and Spiti	29.4 (24.7-34.4)	25.7 (21.2-30.5)	21.3 (15.9-27.5)	-1.39 (-3.04 to 0.33)	-2.68 (-5.79 to 0.53)	-1.93 (-3.59 to -0.52)
Mandi	26.6 (23.4-30.1)	22.3 (19.2-25.7)	17.5 (13.8-21.8)	-1.75 (-3.17 to -0.34)	-3.43 (-6.50 to -0.53)	-2.45 (-3.88 to -1.17)
Shimla	31.7 (27.6-36.6)	27.3 (23.4-31.9)	21.9 (16.9-27.8)	-1.49 (-2.88 to -0.13)	-3.15 (-6.27 to -0.08)	-2.18 (-3.62 to -0.76)
Sirmaur	28.6 (25.4-32.2)	24.6 (21.6-27.8)	19.8 (15.7-24.6)	-1.61 (-2.72 to -0.51)	-3.08 (-5.88 to -0.11)	-2.22 (-3.51 to -0.94)
Solan	24.8 (21.8-27.8)	19.1 (16.6-22.0)	15.9 (12.5-20.0)	-2.56 (-3.79 to -1.27)	-2.65 (-5.55 to 0.38)	-2.60 (-3.97 to -1.33)
Una	24.3 (21.3-27.9)	18.7 (15.9-22.2)	15.8 (12.2-19.9)	-2.59 (-3.97 to -1.20)	-2.49 (-5.61 to 0.56)	-2.55 (-3.93 to -1.24)
**UTs other than Delhi**	**19.7 (16.4-23.3)**	**17.7 (14.0-21.8)**	**14.1 (12.2-16.7)**	**-1.07 (-1.99 to -0.14)**	**-3.15 (-5.28 to -0.90)**	**-1.93 (-2.89 to -0.88)**
Chandigarh	19.3 (16.7-22.2)	19.5 (16.6-22.9)	14.4 (11.2-18.2)	0.47 (-0.91 to 1.83)	-4.23 (-7.19 to -1.08)	-1.50 (-2.90 to -0.16)
Dadra & Nagar Haveli	24.7 (20.5-29.7)	24.5 (20.1-29.5)	19.3 (14.0-25.6)	0.10 (-1.65 to 1.90)	-3.39 (-7.26 to 0.67)	-1.36 (-3.17 to 0.47)
Daman	21.2 (17.8-24.8)	20.2 (16.8-24.0)	15.2 (11.2-20.1)	-0.49 (-2.24 to 1.22)	-4.05 (-7.76 to -0.10)	-1.98 (-3.70 to -0.23)
Diu	25.1 (20.0-30.9)	21.0 (16.3-26.3)	17.1 (12.2-23.9)	-0.84 (-2.89 to 1.29)	-3.68 (-7.69 to 0.52)	-2.02 (-3.81 to 0.05)
Karaikal	21.1 (17.2-25.4)	14.5 (11.7-18.0)	13.4 (9.3-18.9)	-3.51 (-5.43 to -1.49)	-0.90 (-5.62 to 3.84)	-2.45 (-4.55 to -0.34)
Lakshadweep	10.1 (3.9-22.1)	7.2 (2.8-16.4)	6.3 (2.3-14.1)	-3.26 (-6.83 to 0.06)		
Mahe	11.7 (9.6-14.1)	7.5 (6.0-9.1)	6.6 (4.5-9.3)	-3.51 (-5.49 to -1.31)	-2.16 (-6.76 to 2.44)	-2.96 (-4.99 to -0.95)
Nicobars	10.2 (4.4-21.6)	8.6 (3.6-17.6)	7.1 (2.6-15.3)	-1.64 (-6.00 to 3.11)	-2.99 (-8.74 to 3.34)	-2.21 (-6.00 to 1.66)
North & Middle Andaman	22.3 (15.6-31.1)	20.4 (14.1-29.4)	14.9 (8.7-23.8)	-0.88 (-4.36 to 2.65)	-4.63 (-10.28 to 1.01)	-2.45 (-5.59 to 0.57)
Puducherry	18.6 (15.8-21.6)	13.0 (11.0-15.5)	11.8 (8.6-16.1)	-3.49 (-5.16 to -1.80)	-1.32 (-5.79 to 3.27)	-2.61 (-4.48 to -0.59)
South Andaman	16.4 (11.3-23.8)	14.1 (9.6-20.3)	10.6 (6.1-17.1)	-1.49 (-4.91 to 2.06)	-4.20 (-10.30 to 1.60)	-2.63 (-5.77 to 0.46)
Yanam	22.6 (18.1-28.1)	16.4 (13.2-20.4)	11.8 (8.3-16.2)	-3.08 (-4.96 to -1.16)	-4.97 (-8.55 to -0.93)	-3.87 (-5.65 to -2.08)
**Kerala**	**12.9 (11.3-14.7)**	**8.7 (7.3-10.2)**	**7.1 (6.1-8.4)**	**-3.95 (-5.65 to -2.22)**	**-2.81 (-6.97 to 1.76)**	**-3.49 (-5.34 to -1.59)**
Alappuzha	11.6 (8.8-14.7)	7.9 (6.0-10.3)	6.2 (4.1-9.1)	-3.69 (-5.86 to -1.40)	-3.57 (-8.15 to 1.73)	-3.65 (-5.89 to -1.35)
Ernakulam	10.8 (8.8-13.1)	7.1 (5.6-9.0)	5.8 (3.9-8.2)	-4.14 (-6.20 to -2.19)	-3.03 (-7.34 to 1.90)	-3.69 (-5.71 to -1.53)
Idukki	17.1 (14.3-20.3)	11.6 (9.5-14.0)	9.9 (6.8-13.9)	-3.85 (-5.76 to -2.00)	-2.26 (-6.81 to 2.71)	-3.21 (-5.33 to -1.22)

Kannur	14.2 (11.7-16.8)	8.9 (7.2-10.8)	7.9 (5.4-10.8)	-4.57 (-6.46 to -2.51)	-1.84 (-6.29 to 2.83)	-3.46 (-5.45 to -1.48)
Kasaragod	13.7 (10.8-16.8)	8.8 (6.9-11.0)	7.4 (4.9-10.6)	-4.34 (-6.40 to -2.10)	-2.55 (-7.12 to 2.04)	-3.62 (-5.65 to -1.68)
Kollam	12.3 (9.5-15.3)	8.6 (6.7-10.8)	6.9 (4.5-10.0)	-3.50 (-5.70 to -1.21)	-3.23 (-7.95 to 2.06)	-3.40 (-5.68 to -1.22)
Kottayam	12.0 (9.5-14.7)	8.0 (6.2-10.1)	6.6 (4.4-9.6)	-4.01 (-6.09 to -1.87)	-2.90 (-7.42 to 2.23)	-3.57 (-5.74 to -1.39)
Kozhikode	13.5 (11.0-16.3)	8.9 (7.1-10.9)	7.6 (5.1-10.8)	-4.06 (-5.95 to -2.07)	-2.35 (-6.79 to 2.36)	-3.37 (-5.38 to -1.40)
Malappuram	14.1 (11.8-16.7)	9.5 (7.7-11.6)	7.8 (5.4-11.0)	-3.87 (-5.70 to -1.82)	-2.81 (-7.09 to 1.87)	-3.44 (-5.43 to -1.48)
Palakkad	13.9 (11.2-16.9)	9.2 (7.3-11.6)	7.6 (5.2-10.8)	-4.05 (-5.95 to -2.03)	-2.71 (-7.12 to 2.15)	-3.51 (-5.48 to -1.47)
Pathanamthitta	13.5 (10.6-16.7)	9.1 (7.0-11.4)	7.7 (5.1-11.2)	-3.95 (-6.07 to -1.78)	-2.37 (-7.08 to 3.01)	-3.31 (-5.53 to -1.07)
Thiruvananthapuram	12.3 (9.4-15.8)	8.8 (6.7-11.1)	6.8 (4.2-10.1)	-3.32 (-5.72 to -0.85)	-3.76 (-8.62 to 1.59)	-3.51 (-5.90 to -1.20)
Thrissur	10.7 (8.4-13.3)	7.0 (5.4-9.1)	5.8 (3.8-8.3)	-4.13 (-6.16 to -2.00)	-2.90 (-7.20 to 2.07)	-3.63 (-5.66 to -1.49)
Wayanad	17.9 (14.6-21.7)	11.9 (9.4-14.7)	9.7 (6.6-13.7)	-4.04 (-5.88 to -1.99)	-2.85 (-7.25 to 1.60)	-3.56 (-5.59 to -1.69)
**Delhi**	**31.1 (26.4-35.7)**	**19.5 (15.8-23.9)**	**16.1 (13.9-18.8)**	**-4.55 (-5.44 to -3.64)**	**-2.79 (-5.63 to 0.05)**	**-3.83 (-5.06 to -2.73)**
Central	32.1 (28.7-35.5)	20.2 (17.8-22.5)	16.8 (13.3-20.9)	-4.55 (-5.47 to -3.65)	-2.63 (-5.49 to 0.23)	-3.76 (-5.00 to -2.64)
East	31.2 (28.0-34.2)	19.4 (17.3-21.6)	16.1 (12.9-20.0)	-4.33 (-5.26 to -3.42)	-3.07 (-5.88 to -0.23)	-3.82 (-5.05 to -2.69)
New Delhi	31.2 (28.0-34.3)	20.2 (17.9-22.5)	16.8 (13.4-20.8)	-4.25 (-5.15 to -3.35)	-2.68 (-5.51 to 0.20)	-3.61 (-4.83 to -2.52)
North	31.4 (28.2-34.7)	19.5 (17.3-21.7)	15.9 (12.7-19.7)	-4.55 (-5.46 to -3.66)	-2.71 (-5.54 to 0.15)	-3.80 (-5.03 to -2.68)
North East	31.7 (28.5-34.9)	19.6 (17.4-21.9)	16.1 (12.9-20.1)	-4.65 (-5.59 to -3.75)	-2.84 (-5.67 to 0.00)	-3.91 (-5.14 to -2.78)
North West	31.3 (28.1-34.5)	19.2 (16.9-21.4)	15.7 (12.6-19.4)	-4.79 (-5.70 to -3.87)	-2.90 (-5.73 to -0.05)	-4.02 (-5.24 to -2.92)
Shahdara	30.7 (27.6-33.8)	20.0 (17.8-22.3)	15.8 (12.6-19.7)	-4.58 (-5.52 to -3.68)	-3.08 (-5.93 to -0.25)	-3.97 (-5.20 to -2.83)
South	31.3 (28.1-34.3)	20.0 (17.8-22.1)	16.5 (13.3-20.5)	-4.38 (-5.27 to -3.47)	-2.71 (-5.57 to 0.25)	-3.70 (-4.92 to -2.58)
South East	30.4 (27.3-33.4)	20.2 (18.0-22.4)	16.7 (13.4-20.7)	-4.45 (-5.35 to -3.54)	-2.68 (-5.54 to 0.22)	-3.73 (-4.94 to -2.61)
South West	30.8 (27.8-33.7)	19.5 (17.4-21.7)	16.2 (12.9-19.9)	-4.47 (-5.39 to -3.54)	-2.68 (-5.53 to 0.18)	-3.74 (-4.96 to -2.67)
West	30.4 (27.3-33.5)	19.1 (16.9-21.2)	15.8 (12.6-19.6)	-4.57 (-5.51 to -3.63)	-2.68 (-5.52 to 0.18)	-3.80 (-5.02 to -2.72)
**Goa**	**19.5 (16.8-22.6)**	**13.9 (11.1-17.0)**	**11.0 (7.8-15.6)**	**-3.31 (-5.34 to -1.26)**	**-3.50 (-7.90 to 0.83)**	**-3.40 (-5.43 to -1.39)**
North Goa	19.7 (16.0-24.1)	14.3 (11.4-17.9)	11.3 (7.6-16.0)	-3.16 (-5.19 to -1.07)	-3.44 (-7.87 to 0.94)	-3.28 (-5.33 to -1.25)
South Goa	19.3 (15.9-23.1)	13.6 (11.1-16.6)	10.7 (7.3-14.7)	-3.44 (-5.43 to -1.42)	-3.56 (-7.95 to 0.79)	-3.50 (-5.52 to -1.54)

SDI=Socio-demographic Index. UTs=Union territories.

*The states are listed in increasing order of Socio-demographic Index in 2017.

†The state of Jammu and Kashmir was divided into two union territories in August 2019; as we are reporting findings up to 2017, we report findings for the undivided state of Jammu and Kashmir.

11. Coefficient of variation for U5MR and NMR between districts within the states of India, 2000 and 2017

States*	U5MR						NMR					
	Rate			CV			Rate			CV		
	2000	2017	Percent change, 2000-2017	2000	2017	Percent change, 2000-2017	2000	2017	Percent change, 2000-2017	2000	2017	Percent change, 2000-2017
**India**	**83.1**	**42.4**	**-49.0**	**34.2**	**38.5**	**12.6**	**38.0**	**23.5**	**-38.2**	**30.8**	**35.6**	**15.7**
Bihar	88.8	43.8	-50.7	7.6	6.8	-10.2	36.5	23.4	-35.9	7.7	6.7	-13.0
Madhya Pradesh	110.5	50.7	-54.1	19.7	21.7	10.1	48.3	26.9	-44.3	18.5	21.2	14.8
Jharkhand	87.2	43.9	-49.7	10.0	11.7	17.0	37.5	22.9	-38.9	9.3	11.5	23.0
Uttar Pradesh	112.8	59.7	-47.1	13.0	18.0	38.7	48.2	31.7	-34.2	12.0	17.4	45.8
Rajasthan	90.5	48.3	-46.6	28.3	25.1	-11.4	40.5	26.5	-34.6	27.6	24.2	-12.5
Chhattisgarh	94.7	47.7	-49.6	8.6	11.7	36.6	48.2	29.2	-39.4	8.1	11.1	36.0
Odisha	99.1	49.1	-50.5	13.3	25.5	92.2	45.3	25.5	-43.7	13.5	26.0	91.9
Assam	87.6	54.9	-37.3	12.0	26.2	118.2	43.5	29.5	-32.2	13.5	26.2	93.6
Andhra Pradesh	81.8	36.1	-55.9	20.2	17.6	-12.8	37.1	20.1	-45.8	19.6	17.7	-9.8
West Bengal	65.2	29.2	-55.2	15.6	20.3	29.9	31.9	17.8	-44.2	15.8	21.4	35.4
Tripura	61.2	34.0	-44.4	13.0	23.1	77.7	29.9	18.7	-37.5	12.9	22.3	72.3
Arunachal Pradesh	73.0	27.3	-62.6	16.0	24.7	54.6	28.7	14.4	-49.8	16.6	24.2	45.7
Meghalaya	66.0	39.1	-40.8	11.8	34.2	189.7	24.9	18.8	-24.5	12.5	32.9	163.9
Karnataka	64.9	31.7	-51.2	24.3	25.9	6.3	33.0	18.3	-44.5	24.2	26.4	8.8
Telangana	73.0	28.6	-60.8	14.2	14.6	3.2	34.4	17.6	-48.8	13.5	13.5	0.4
Gujarat	72.7	38.1	-47.6	21.9	21.8	-0.2	36.5	23.1	-36.7	20.2	21.4	6.0
Manipur	47.3	24.9	-47.4	25.0	20.8	-16.7	21.9	13.8	-37.0	27.9	23.3	-16.6
Jammu and Kashmir†	57.9	33.4	-42.3	14.3	12.3	-13.8	27.8	19.7	-29.1	13.5	11.6	-14.4
Haryana	73.2	39.3	-46.3	9.9	15.5	57.2	30.1	20.0	-33.6	8.7	14.4	65.7
Uttarakhand	58.1	30.3	-47.8	14.4	16.4	13.8	27.0	17.1	-36.7	14.2	16.7	17.9
Tamil Nadu	51.4	19.6	-61.9	14.4	15.4	6.3	25.4	11.3	-55.5	14.8	15.6	5.7
Mizoram	44.0	36.3	-17.5	12.5	14.4	15.3	19.9	18.1	-9.0	13.0	11.7	-9.8
Maharashtra	54.0	26.1	-51.7	18.7	19.3	3.2	31.1	17.0	-45.3	18.8	21.0	11.9
Punjab	55.6	29.7	-46.6	12.8	13.5	5.5	26.3	16.4	-37.6	12.6	13.7	8.3
Sikkim	47.5	21.9	-53.9	11.4	14.2	24.4	22.5	12.1	-46.2	11.8	14.2	20.2
Nagaland	60.4	34.8	-42.4	17.3	12.3	-28.9	25.1	15.8	-37.1	20.3	14.4	-28.8
Himachal Pradesh	52.2	31.4	-39.8	12.4	15.7	26.1	27.2	18.6	-31.6	11.3	15.0	32.7
UTs other than Delhi	35.2	22.9	-34.9	32.9	28.7	-12.6	19.7	14.1	-28.4	28.9	30.1	4.2
Kerala	19.9	10.4	-47.7	15.7	16.6	5.8	12.9	7.1	-45.0	15.7	16.9	7.6
Delhi	57.5	25.1	-56.3	1.6	3.2	98.5	31.1	16.1	-48.2	1.7	2.5	50.3
Goa	35.2	18.8	-46.6	1.3	3.9	199.6	19.5	11.0	-43.6	1.4	4.1	197.7

CV=Coefficient of variation, SDI=Socio-demographic Index, UTs=Union Territories.
*The states are listed in increasing order of SDI in 2017
†The state of Jammu and Kashmir was divided into two union territories in August 2019; as we are reporting findings up to 2017, we report findings for the undivided state of Jammu and Kashmir.

12. Projected U5MR and NMR for the districts of India in 2025 and 2030 based on trends from 2000 to 2017

States*/Districts	Projected U5MR (95% uncertainty interval)		Projected NMR (95% uncertainty interval)	
	2025	2030	2025	2030
**Bihar**	**30.9 (23.1-41.4)**	**23.8 (16.7-33.5)**	**18.2 (13.5-24.3)**	**15.3 (10.6-21.5)**
Araria	32.7 (23.3-45.2)	25.4 (16.9-37.3)	18.9 (13.2-26.4)	16.0 (10.5-23.9)
Arwal	31.5 (22.4-43.2)	24.6 (16.2-35.7)	18.8 (13.4-26.1)	16.0 (10.6-23.6)
Aurangabad	33.7 (24.2-47.0)	26.4 (17.7-39.3)	20.1 (14.4-28.0)	17.2 (11.7-25.4)
Banka	33.6 (23.8-46.6)	26.3 (17.3-38.7)	19.7 (14.0-27.6)	16.8 (11.0-25.0)
Begusarai	29.4 (21.1-40.2)	22.7 (15.1-33.0)	17.3 (12.2-23.8)	14.6 (9.6-21.5)
Bhagalpur	32.4 (23.2-45.3)	25.2 (16.8-37.2)	18.9 (13.3-26.9)	16.0 (10.5-24.0)
Bhojpur	29.9 (21.5-40.7)	23.1 (15.6-33.4)	17.8 (12.7-24.1)	15.1 (10.0-21.4)
Buxar	31.0 (22.4-42.3)	24.1 (16.3-34.7)	18.5 (13.0-25.4)	15.7 (10.3-22.8)
Darbhanga	29.7 (21.2-40.9)	22.6 (15.0-32.9)	17.4 (12.3-24.1)	14.4 (9.4-21.3)
East Champaran	30.6 (21.5-42.1)	23.4 (15.1-34.0)	18.1 (12.6-25.2)	15.0 (9.8-22.1)
Gaya	32.8 (24.0-44.6)	25.8 (17.5-36.7)	19.6 (14.2-26.7)	16.8 (11.4-24.3)
Gopalganj	27.9 (19.4-38.4)	21.3 (13.8-30.9)	16.5 (11.5-22.9)	13.8 (8.8-20.2)
Jamui	32.5 (23.7-44.3)	25.5 (17.3-36.6)	19.2 (13.8-26.5)	16.4 (11.0-24.1)
Jehanabad	31.0 (22.4-42.0)	24.1 (16.3-34.7)	18.5 (13.3-25.3)	15.7 (10.5-22.7)
Kaimur	34.1 (24.2-47.0)	26.7 (17.7-38.8)	20.2 (14.3-28.1)	17.3 (11.4-25.5)
Katihar	36.4 (25.8-51.0)	28.2 (18.7-41.7)	21.2 (14.9-29.7)	17.8 (11.6-26.8)
Khagaria	30.5 (21.5-42.1)	23.7 (15.5-35.0)	17.9 (12.5-24.9)	15.2 (9.9-22.4)
Kishanganj	32.8 (23.1-45.1)	25.6 (16.8-37.4)	19.0 (13.3-26.5)	16.0 (10.4-23.9)
Lakhisarai	30.7 (21.9-42.5)	24.0 (15.8-35.2)	18.2 (12.8-25.4)	15.5 (10.1-23.2)
Madhepura	32.2 (22.7-45.1)	25.1 (16.5-37.2)	18.8 (13.1-26.4)	15.9 (10.3-23.7)
Madhubani	32.3 (23.1-45.3)	24.3 (16.0-36.2)	19.0 (13.2-26.7)	15.5 (10.0-23.2)
Munger	29.1 (20.9-40.2)	22.5 (15.1-33.0)	17.1 (12.1-23.9)	14.4 (9.5-21.4)
Muzaffarpur	28.5 (20.1-39.4)	21.7 (14.1-31.6)	16.8 (11.7-23.2)	13.9 (9.0-20.3)
Nalanda	30.5 (22.3-42.2)	23.8 (16.3-34.7)	18.2 (13.1-25.1)	15.5 (10.4-22.4)
Nawada	31.9 (23.5-43.7)	25.1 (17.3-36.2)	19.0 (13.7-26.3)	16.3 (11.0-23.8)
Patna	26.6 (19.4-36.4)	20.4 (13.9-29.2)	15.8 (11.4-21.6)	13.2 (8.9-19.0)
Purnia	36.1 (25.6-50.2)	28.3 (18.7-41.4)	21.0 (14.8-29.5)	17.9 (11.8-26.6)
Rohtas	32.1 (23.1-43.7)	24.9 (16.9-36.0)	19.1 (13.5-26.4)	16.2 (10.8-23.9)
Saharsa	30.7 (21.4-42.9)	23.9 (15.3-35.8)	17.9 (12.4-25.0)	15.2 (9.8-22.7)
Samastipur	29.4 (21.1-40.0)	22.5 (15.0-32.6)	17.3 (12.3-23.7)	14.5 (9.5-21.1)
Saran	27.3 (19.4-37.4)	20.9 (13.8-30.3)	16.2 (11.4-22.5)	13.5 (8.8-19.7)
Sheikhpura	31.6 (22.7-44.0)	24.8 (16.6-36.6)	18.8 (13.2-26.3)	16.1 (10.4-23.8)
Sheohar	32.0 (22.5-44.2)	24.3 (15.9-35.1)	18.9 (13.3-26.4)	15.6 (10.2-23.1)
Sitamarhi	34.6 (24.6-48.1)	26.1 (17.3-38.5)	20.4 (14.2-28.6)	16.8 (10.9-25.2)
Siwan	27.6 (19.4-37.4)	21.1 (13.8-30.2)	16.4 (11.4-22.5)	13.6 (8.8-19.7)
Supaul	31.0 (21.9-43.3)	23.7 (15.6-35.4)	18.0 (12.7-25.5)	15.0 (9.8-22.8)
Vaishali	27.7 (19.9-37.5)	21.1 (14.1-30.1)	16.4 (11.6-22.3)	13.6 (9.0-19.5)
West Champaran	30.1 (21.3-41.1)	23.0 (15.2-33.5)	17.7 (12.3-24.6)	14.8 (9.4-21.7)
**Madhya Pradesh**	**34.7 (25.8-45.3)**	**26.2 (18.4-36.0)**	**19.6 (14.4-25.4)**	**15.8 (10.9-21.6)**
Agar Malwa	34.4 (24.4-47.9)	25.8 (17.0-38.3)	19.5 (13.4-27.2)	15.5 (9.9-23.2)
Alirajpur	40.0 (27.2-55.8)	31.4 (19.7-46.2)	21.9 (14.8-30.8)	18.2 (11.4-27.5)
Anuppur	38.9 (27.0-53.5)	30.0 (19.2-43.9)	22.6 (15.5-31.5)	18.7 (11.8-27.5)
Ashoknagar	38.9 (27.3-52.1)	29.6 (19.4-41.9)	22.0 (15.2-29.8)	17.8 (11.4-25.8)
Balaghat	29.9 (21.1-41.6)	22.5 (14.7-33.4)	17.5 (12.1-24.8)	14.1 (9.0-21.1)
Barwani	32.8 (22.4-45.4)	25.7 (16.2-37.9)	18.2 (12.3-25.7)	15.2 (9.4-22.7)
Betul	24.2 (16.0-34.2)	18.0 (10.9-27.3)	13.9 (9.1-20.0)	11.0 (6.6-17.0)
Bhind	37.1 (26.8-50.3)	28.6 (19.3-40.6)	20.8 (14.7-28.7)	17.2 (11.3-25.1)
Bhopal	22.5 (15.2-32.1)	16.7 (10.3-25.6)	12.8 (8.5-18.5)	10.2 (6.2-15.7)
Burhanpur	31.0 (21.1-44.0)	24.1 (15.1-36.2)	17.8 (11.8-25.4)	14.7 (9.0-22.5)
Chhatarpur	45.9 (33.0-61.5)	34.5 (23.3-48.9)	25.9 (18.4-35.3)	20.8 (13.9-29.8)
Chhindwara	24.3 (16.5-34.2)	18.1 (11.3-27.1)	14.1 (9.5-19.8)	11.2 (7.0-16.8)
Damoh	46.7 (32.9-63.7)	35.1 (23.1-50.6)	26.6 (18.8-36.5)	21.4 (14.1-30.9)
Datia	35.6 (25.3-48.1)	27.0 (17.9-38.7)	20.0 (14.1-27.3)	16.2 (10.6-23.4)

Dewas	24.5 (16.7-33.9)	18.1 (11.3-26.7)	14.0 (9.4-19.2)	11.0 (6.7-16.0)
Dhar	29.4 (20.1-40.7)	22.4 (14.2-32.8)	16.3 (11.1-22.5)	13.2 (8.3-19.4)
Dindori	44.9 (31.4-62.3)	34.5 (22.7-51.3)	25.9 (17.8-36.4)	21.3 (13.6-32.0)
Guna	38.1 (27.2-53.4)	29.0 (19.3-42.8)	21.5 (14.9-30.2)	17.4 (11.2-26.0)
Gwalior	37.1 (26.3-50.3)	28.7 (19.0-41.0)	20.7 (14.7-28.5)	17.2 (11.4-25.1)
Harda	29.9 (19.9-43.4)	22.4 (13.6-34.7)	17.2 (11.1-25.3)	13.7 (8.1-21.8)
Hoshangabad	24.8 (17.0-34.6)	18.2 (11.4-26.7)	14.2 (9.6-19.8)	11.1 (6.9-16.5)
Indore	20.0 (13.7-27.9)	14.7 (9.2-21.7)	11.3 (7.7-15.8)	8.8 (5.5-13.1)
Jabalpur	35.6 (24.6-49.6)	26.3 (16.9-39.0)	20.4 (14.0-28.4)	16.1 (10.3-24.0)
Jhabua	34.7 (23.6-48.4)	26.3 (16.8-39.2)	18.9 (12.7-26.5)	15.2 (9.5-22.7)
Katni	47.5 (33.4-64.5)	35.8 (23.2-51.8)	27.0 (18.8-36.9)	21.8 (14.1-32.0)
Khandwa	32.4 (21.7-45.7)	24.6 (15.3-37.0)	18.5 (12.2-26.5)	15.0 (9.1-23.0)
Khargone	31.5 (21.4-43.9)	24.2 (15.2-35.5)	17.8 (11.9-25.0)	14.5 (9.0-21.6)
Mandla	37.6 (26.1-52.1)	28.3 (18.3-41.7)	21.7 (15.0-30.0)	17.5 (11.2-25.8)
Mandsaur	31.8 (22.7-44.0)	23.6 (15.7-34.6)	17.7 (12.4-24.5)	13.9 (9.1-20.5)
Morena	42.3 (30.2-57.1)	33.4 (22.2-47.4)	23.5 (16.6-32.2)	19.8 (13.0-28.6)
Narsinghpur	30.0 (20.8-41.6)	22.1 (14.1-32.2)	17.2 (11.8-23.9)	13.5 (8.6-20.0)
Neemuch	32.2 (23.4-44.0)	23.8 (16.2-34.3)	17.8 (12.9-24.5)	14.0 (9.5-20.3)
Niwari	36.3 (25.6-49.8)	27.1 (17.7-39.3)	20.5 (14.2-28.3)	16.4 (10.5-24.2)
Panna	51.0 (36.2-69.1)	38.4 (25.5-55.1)	28.8 (20.1-39.4)	23.2 (15.1-33.8)
Raisen	29.9 (20.1-41.0)	22.3 (13.9-32.7)	17.1 (11.4-23.7)	13.6 (8.4-19.9)
Rajgarh	34.5 (24.1-47.7)	26.0 (16.9-38.4)	19.5 (13.6-27.4)	15.7 (10.1-23.5)
Ratlam	26.5 (18.5-36.6)	19.6 (12.7-28.9)	14.6 (10.2-20.5)	11.5 (7.4-17.1)
Rewa	42.9 (29.9-59.4)	32.4 (21.0-47.2)	24.2 (16.9-33.6)	19.6 (12.7-29.2)
Sagar	40.5 (27.8-55.7)	30.4 (19.1-43.7)	23.0 (15.4-31.8)	18.5 (11.5-26.9)
Satna	43.7 (31.1-59.7)	32.6 (21.5-47.2)	24.6 (17.1-33.6)	19.7 (12.7-28.5)
Sehore	25.8 (17.6-35.8)	19.2 (12.0-28.3)	14.7 (9.9-20.6)	11.7 (7.2-17.3)
Seoni	28.3 (19.6-39.6)	21.0 (13.5-31.1)	16.3 (11.2-23.0)	13.0 (8.2-19.4)
Shahdol	46.2 (32.5-64.1)	35.7 (23.4-52.7)	26.4 (18.4-37.2)	21.9 (14.0-32.6)
Shajapur	26.9 (18.5-36.7)	20.1 (12.7-29.1)	15.3 (10.4-21.0)	12.2 (7.6-17.8)
Sheopur	52.2 (38.2-71.3)	40.9 (28.1-59.0)	28.9 (20.6-39.4)	24.2 (16.1-34.8)
Shivpuri	40.5 (29.0-54.3)	30.8 (20.8-43.7)	22.6 (16.0-30.7)	18.4 (12.2-26.6)
Sidhi	47.0 (32.9-64.4)	36.2 (23.6-52.3)	26.6 (18.3-37.1)	22.0 (13.9-32.3)
Singrauli	45.0 (31.3-62.0)	34.8 (22.5-50.4)	25.7 (17.6-35.6)	21.3 (13.6-31.4)
Tikamgarh	44.2 (31.6-59.9)	33.4 (22.3-47.5)	25.0 (17.7-33.9)	20.2 (13.3-28.8)
Ujjain	23.0 (15.9-32.2)	17.0 (11.0-25.4)	13.0 (8.9-18.2)	10.2 (6.5-15.2)
Umaria	49.7 (34.4-69.1)	38.1 (24.6-56.1)	28.3 (19.1-40.0)	23.3 (14.6-35.1)
Vidisha	38.8 (26.4-53.6)	29.7 (18.7-43.0)	22.0 (14.9-30.7)	18.0 (11.2-26.4)
**Jharkhand**	**31.3 (23.6-41.3)**	**24.2 (17.4-34.0)**	**17.4 (13.0-23.3)**	**14.4 (10.2-20.4)**
Bokaro	27.4 (20.1-37.4)	20.8 (14.4-30.2)	15.3 (11.1-21.0)	12.5 (8.5-18.0)
Chatra	34.8 (25.4-47.5)	27.5 (18.9-39.6)	19.6 (14.3-26.9)	16.6 (11.4-24.0)
Deoghar	32.0 (23.5-43.9)	25.1 (17.2-36.0)	18.0 (13.1-24.8)	15.1 (10.2-21.9)
Dhanbad	29.0 (21.1-39.8)	22.3 (15.1-32.3)	16.4 (11.8-22.5)	13.4 (9.0-19.7)
Dumka	32.3 (23.5-43.5)	25.1 (17.2-35.7)	18.1 (13.1-25.0)	15.0 (10.2-21.9)
East Singhbhum	22.3 (15.5-31.7)	16.9 (11.0-25.5)	12.0 (8.3-17.1)	9.7 (6.2-14.5)
Garhwa	37.1 (26.7-51.4)	29.4 (19.8-43.2)	20.7 (14.7-28.8)	17.7 (11.7-25.8)
Giridih	30.8 (22.3-41.9)	24.1 (16.5-34.5)	17.4 (12.5-23.8)	14.6 (9.8-21.2)
Godda	34.2 (24.4-47.0)	26.9 (17.8-39.2)	19.1 (13.5-26.6)	16.0 (10.5-23.6)
Gumla	37.2 (26.9-50.0)	28.7 (19.5-40.9)	20.5 (14.8-28.2)	17.0 (11.4-24.8)
Hazaribagh	28.9 (21.2-39.0)	22.2 (15.4-31.7)	16.3 (11.8-22.3)	13.4 (9.2-19.3)
Jamtara	34.0 (24.3-46.3)	26.6 (17.8-38.3)	19.2 (13.6-26.7)	16.0 (10.7-23.5)
Khunti	33.4 (24.0-46.4)	25.4 (17.1-37.3)	18.2 (12.9-25.3)	14.8 (9.8-21.8)
Koderma	29.4 (21.5-39.7)	23.1 (15.9-32.9)	16.7 (12.0-22.7)	14.0 (9.5-20.1)
Latehar	38.1 (27.7-51.6)	30.1 (20.7-43.0)	21.2 (15.4-28.9)	17.9 (12.2-25.8)
Lohardaga	36.3 (26.1-50.1)	28.0 (18.9-41.0)	20.1 (14.4-28.1)	16.6 (11.1-24.5)
Pakur	32.4 (23.5-43.9)	25.3 (17.2-36.1)	18.2 (13.1-25.0)	15.0 (10.2-22.0)
Palamu	36.7 (26.6-50.7)	29.1 (19.8-42.2)	20.5 (14.9-28.1)	17.4 (11.9-25.3)
Ramgarh	25.6 (18.6-35.0)	19.2 (13.1-27.8)	14.3 (10.3-19.6)	11.5 (7.8-16.8)

Ranchi	28.7 (20.8-39.2)	21.7 (14.7-31.3)	15.8 (11.4-21.8)	12.8 (8.6-18.6)
Sahibganj	33.6 (24.2-45.7)	26.2 (17.7-37.8)	18.7 (13.3-25.8)	15.5 (10.4-22.8)
Saraikela Kharsawan	26.9 (19.1-37.9)	20.4 (13.7-30.7)	14.6 (10.3-20.5)	11.8 (7.8-17.6)
Simdega	36.3 (26.4-49.3)	27.9 (19.0-40.0)	20.0 (14.5-27.3)	16.4 (11.1-23.6)
West Singhbhum	35.9 (25.7-49.2)	28.0 (18.5-40.4)	19.4 (13.8-26.9)	16.1 (10.7-23.8)
**Uttar Pradesh**	**43.6 (34.2-56.2)**	**34.4 (25.9-46.4)**	**25.0 (19.5-32.3)**	**21.2 (15.7-28.6)**
Agra	43.2 (31.4-57.3)	34.5 (23.4-48.3)	24.4 (17.6-32.5)	20.9 (14.2-29.5)
Aligarh	51.4 (38.4-68.4)	41.8 (29.5-58.4)	28.9 (21.4-38.5)	25.3 (17.7-35.4)
Allahabad	47.3 (32.8-65.4)	36.7 (23.9-53.7)	27.4 (19.1-38.5)	22.9 (14.8-34.3)
Ambedkar Nagar	31.7 (22.8-43.7)	24.2 (16.1-35.0)	18.8 (13.2-25.7)	15.4 (10.2-22.6)
Amethi	43.3 (31.2-60.9)	33.8 (22.7-50.4)	25.1 (18.0-35.4)	21.1 (14.1-31.6)
Amroha	48.2 (35.5-64.7)	38.9 (26.9-55.1)	27.2 (19.8-36.5)	23.7 (16.1-33.7)
Auraiya	39.5 (29.0-53.2)	30.6 (21.1-43.2)	22.7 (16.4-30.7)	18.9 (12.8-27.0)
Azamgarh	32.0 (22.8-43.5)	24.4 (16.2-35.0)	19.0 (13.3-25.9)	15.6 (10.2-22.6)
Baghpat	36.2 (26.7-47.7)	28.6 (19.9-39.8)	20.2 (14.8-27.3)	17.2 (11.8-24.4)
Bahraich	60.9 (45.1-82.7)	49.2 (34.3-70.2)	34.6 (25.4-46.9)	30.0 (20.5-43.1)
Ballia	29.9 (21.5-40.7)	22.9 (15.4-32.7)	17.7 (12.6-24.1)	14.6 (9.6-21.0)
Balrampur	55.1 (40.5-76.9)	43.7 (30.0-64.8)	32.1 (23.4-45.1)	27.3 (18.5-40.8)
Banda	46.0 (32.7-61.8)	35.6 (23.8-50.6)	26.0 (18.3-35.4)	21.7 (14.4-31.4)
Barabanki	48.5 (35.5-67.1)	38.6 (26.4-56.4)	28.0 (20.4-38.6)	24.0 (16.4-35.2)
Bareilly	54.1 (40.4-71.1)	43.7 (30.8-60.7)	30.7 (22.5-41.0)	26.7 (18.4-37.5)
Basti	38.0 (27.3-53.0)	29.3 (19.6-43.1)	22.4 (15.9-31.2)	18.6 (12.3-27.6)
Bhadohi	43.2 (30.9-59.4)	33.4 (22.1-48.7)	25.2 (17.8-35.2)	21.0 (13.8-31.1)
Bijnor	41.3 (31.0-54.2)	33.1 (23.4-45.5)	23.3 (17.3-30.8)	20.1 (14.1-27.9)
Budaun	70.3 (53.1-92.3)	58.7 (41.7-81.3)	40.0 (29.4-53.2)	35.9 (24.8-50.6)
Bulandshahr	48.7 (36.5-64.7)	39.3 (27.9-54.9)	27.3 (20.1-36.2)	23.7 (16.5-32.9)
Chandauli	37.0 (26.6-50.3)	28.6 (19.2-41.1)	21.8 (15.4-30.0)	18.1 (11.9-26.3)
Chitrakoot	50.2 (35.5-67.6)	39.1 (25.7-55.2)	28.5 (19.8-39.1)	23.9 (15.5-34.8)
Deoria	28.2 (20.0-38.7)	21.2 (14.0-30.6)	16.7 (11.7-22.9)	13.5 (8.8-19.6)
Etah	57.2 (42.3-77.4)	46.3 (32.1-66.3)	32.6 (23.6-44.5)	28.4 (19.1-41.2)
Etawah	42.4 (31.0-57.3)	33.0 (22.5-47.4)	24.3 (17.4-33.1)	20.3 (13.6-29.4)
Faizabad	39.0 (28.3-54.6)	30.2 (20.5-45.1)	22.8 (16.3-31.9)	19.0 (12.7-28.3)
Farrukhabad	57.1 (42.8-76.4)	46.2 (32.5-65.4)	32.6 (24.1-43.8)	28.4 (19.8-40.4)
Fatehpur	42.1 (30.3-57.0)	32.5 (22.0-47.0)	23.9 (17.0-33.0)	19.9 (13.2-29.3)
Firozabad	51.5 (37.2-69.9)	41.3 (27.7-59.2)	29.3 (20.8-40.1)	25.3 (16.7-36.7)
Gautam Buddha Nagar	30.2 (22.2-40.2)	23.8 (16.7-33.4)	17.1 (12.4-23.0)	14.5 (9.9-20.5)
Ghaziabad	32.0 (23.7-42.8)	25.1 (17.5-35.4)	18.0 (13.2-24.2)	15.2 (10.5-21.5)
Ghazipur	34.7 (24.6-47.9)	26.7 (17.6-39.1)	20.5 (14.4-28.6)	17.1 (11.1-25.5)
Gonda	52.2 (38.0-73.7)	41.5 (28.2-62.6)	30.5 (21.8-42.9)	26.0 (17.3-39.2)
Gorakhpur	29.8 (20.6-41.8)	22.5 (14.4-33.7)	17.7 (12.1-24.6)	14.4 (9.1-21.2)
Hamirpur	37.5 (27.0-50.7)	28.6 (19.3-40.6)	21.4 (15.2-29.0)	17.6 (11.7-25.4)
Hapur	42.3 (31.5-56.1)	33.6 (23.5-46.9)	23.7 (17.4-31.8)	20.3 (14.0-28.5)
Hardoi	56.6 (42.4-75.6)	45.7 (32.4-64.0)	32.3 (24.0-43.5)	28.1 (19.6-39.8)
Hathras	50.1 (36.9-66.7)	40.1 (27.8-56.3)	28.2 (20.6-37.5)	24.4 (16.6-34.1)
Jalaun	35.6 (25.4-48.7)	27.5 (18.4-40.0)	20.5 (14.3-28.7)	17.0 (11.1-25.2)
Jaunpur	38.6 (27.9-52.5)	29.8 (20.3-42.8)	22.7 (16.4-31.2)	18.8 (12.8-27.3)
Jhansi	39.5 (28.3-53.4)	30.5 (20.5-43.3)	22.6 (15.9-31.0)	18.8 (12.3-27.0)
Kannauj	47.2 (34.9-63.8)	37.4 (26.1-53.7)	27.1 (19.7-36.5)	23.1 (15.8-33.1)
Kanpur Dehat	35.8 (26.2-48.0)	27.4 (18.8-38.7)	20.5 (14.9-28.3)	16.9 (11.4-24.7)
Kanpur Nagar	31.8 (23.1-43.0)	24.0 (16.5-34.3)	18.1 (13.0-24.8)	14.7 (9.9-21.5)
Kasganj	65.4 (48.4-87.4)	54.2 (37.8-76.4)	37.3 (27.3-50.3)	33.2 (22.8-47.5)
Kaushambi	47.0 (33.9-64.3)	36.6 (24.7-53.4)	27.0 (19.1-37.6)	22.7 (15.0-33.5)
Khushinagar	31.6 (21.9-44.0)	24.1 (15.5-35.4)	18.7 (12.9-26.2)	15.3 (9.8-22.9)
Lakhimpur Kheri	53.8 (40.1-72.4)	41.9 (29.3-59.4)	30.0 (21.9-41.0)	25.2 (17.1-36.1)
Lalitpur	47.0 (33.0-63.3)	36.8 (24.2-52.3)	27.0 (18.9-36.8)	22.7 (14.7-32.5)
Lucknow	35.2 (25.7-48.5)	27.0 (18.6-39.3)	20.1 (14.4-27.9)	16.6 (11.2-24.2)
Maharajganj	35.4 (24.5-49.8)	27.0 (17.5-40.5)	20.9 (14.5-29.5)	17.1 (11.0-25.8)
Mahoba	42.8 (31.0-57.8)	32.8 (22.3-46.5)	24.5 (17.4-33.3)	20.2 (13.4-29.1)

Mainpuri	50.3 (37.1-68.0)	39.9 (27.7-57.2)	28.7 (20.8-39.3)	24.6 (16.6-35.6)
Mathura	45.7 (33.3-60.9)	36.9 (25.3-51.7)	25.5 (18.5-34.0)	22.2 (15.2-31.1)
Mau	29.8 (21.0-41.0)	22.5 (14.8-33.1)	17.6 (12.3-24.3)	14.3 (9.4-21.0)
Meerut	38.4 (28.7-50.8)	30.3 (21.5-42.1)	21.5 (15.9-28.8)	18.3 (12.7-25.9)
Mirzapur	45.4 (32.9-61.6)	35.6 (23.9-51.1)	26.5 (18.8-36.9)	22.4 (14.8-32.9)
Moradabad	49.8 (37.1-67.3)	40.4 (28.5-57.2)	28.2 (20.7-37.7)	24.7 (16.9-34.7)
Muzaffarnagar	39.8 (30.0-52.4)	31.6 (22.5-43.7)	22.4 (16.8-29.6)	19.1 (13.5-26.6)
Pilibhit	44.6 (33.3-59.1)	34.9 (24.7-48.3)	25.2 (18.6-34.2)	21.2 (14.8-30.1)
Pratapgarh	42.8 (31.0-58.6)	33.1 (22.3-48.0)	24.8 (17.8-34.4)	20.6 (14.0-30.5)
Rae Bareli	40.4 (29.6-55.0)	31.3 (21.5-44.7)	23.1 (16.6-32.2)	19.3 (13.0-28.3)
Rampur	47.8 (35.7-64.8)	38.6 (27.1-54.4)	27.2 (20.1-36.6)	23.6 (16.3-33.6)
Saharanpur	38.8 (28.0-52.0)	31.1 (21.1-43.7)	21.9 (15.9-29.8)	18.9 (12.8-27.1)
Sambhal	61.7 (45.9-82.6)	51.1 (35.7-71.5)	34.9 (25.6-47.0)	31.2 (21.5-44.1)
Sant Kabir Nagar	34.3 (24.3-48.0)	26.3 (17.3-39.0)	20.3 (14.3-28.5)	16.7 (10.9-25.0)
Shahjahanpur	58.8 (44.5-78.4)	47.1 (33.7-66.0)	33.3 (24.9-44.5)	28.7 (20.2-40.6)
Shamli	38.8 (28.5-51.5)	30.7 (21.2-42.9)	21.8 (15.9-28.9)	18.6 (12.7-25.8)
Shravasti	61.2 (45.2-84.1)	49.6 (34.2-72.2)	35.1 (25.6-48.5)	30.5 (20.8-44.8)
Siddharth Nagar	43.7 (31.7-61.1)	33.8 (23.0-50.1)	25.6 (18.4-35.8)	21.3 (14.2-31.8)
Sitapur	55.7 (41.5-74.7)	44.4 (31.2-62.9)	31.6 (23.2-42.8)	27.1 (18.7-38.6)
Sonbhadra	41.6 (29.8-56.6)	32.7 (21.8-47.2)	24.2 (17.1-33.4)	20.5 (13.4-30.3)
Sultanpur	35.8 (26.0-49.3)	27.4 (18.7-39.9)	21.0 (15.1-29.2)	17.3 (11.7-25.5)
Unnao	41.7 (31.0-56.3)	32.5 (22.8-46.0)	23.8 (17.3-32.7)	20.0 (13.7-29.4)
Varanasi	34.9 (25.1-47.5)	26.6 (17.9-38.3)	20.5 (14.7-28.6)	16.8 (11.2-24.6)
**Rajasthan**	**35.4 (27.3-44.9)**	**28.0 (20.5-37.3)**	**20.9 (15.9-26.6)**	**17.7 (12.8-23.5)**
Ajmer	32.8 (23.5-44.8)	25.3 (17.0-36.5)	19.5 (13.7-26.8)	16.1 (10.6-23.4)
Alwar	35.9 (25.8-47.9)	29.1 (19.6-41.1)	21.0 (15.0-28.1)	18.3 (12.4-26.0)
Banswara	34.3 (24.3-48.3)	26.1 (17.3-39.1)	19.7 (13.8-27.5)	16.0 (10.5-23.8)
Baran	41.0 (29.8-56.4)	31.6 (21.7-46.2)	24.3 (17.5-33.5)	20.1 (13.6-29.3)
Barmer	50.8 (36.1-69.1)	41.9 (27.6-59.9)	29.7 (20.8-40.8)	26.0 (17.0-37.7)
Bharatpur	42.6 (31.0-56.4)	34.5 (23.7-48.3)	24.8 (17.8-33.0)	21.7 (14.7-30.3)
Bhilwara	40.4 (29.4-54.8)	30.9 (21.3-44.0)	23.8 (17.4-32.2)	19.5 (13.3-27.8)
Bikaner	28.2 (19.5-39.6)	23.5 (15.0-34.9)	17.1 (11.6-24.5)	15.3 (9.5-23.3)
Bundi	41.9 (30.0-56.9)	32.3 (21.7-46.0)	24.5 (17.5-33.6)	20.3 (13.6-29.2)
Chittorgarh	43.0 (31.2-58.2)	32.7 (22.4-47.0)	25.1 (18.3-34.0)	20.4 (14.0-29.5)
Churu	22.7 (16.1-31.5)	18.3 (12.1-27.1)	13.7 (9.7-19.2)	11.9 (7.8-17.6)
Dausa	39.0 (28.1-52.0)	30.9 (20.9-43.4)	22.8 (16.1-30.8)	19.4 (12.8-27.6)
Dholpur	44.2 (31.9-59.4)	35.7 (24.1-50.7)	26.0 (18.6-35.1)	22.6 (15.2-32.3)
Dungarpur	39.3 (27.6-55.2)	30.1 (19.9-45.1)	22.6 (15.9-31.4)	18.4 (12.0-27.4)
Hanumangarh	23.4 (16.7-32.6)	18.9 (12.6-27.7)	14.2 (10.1-19.8)	12.3 (8.2-18.2)
Jaipur	25.5 (18.5-34.2)	19.9 (13.4-28.0)	15.1 (10.7-20.4)	12.6 (8.3-17.9)
Jaisalmer	41.0 (28.1-57.1)	34.8 (22.0-51.9)	24.1 (16.5-34.0)	21.8 (13.6-32.6)
Jalore	50.8 (36.1-71.0)	41.0 (27.2-60.9)	29.7 (20.8-41.0)	25.5 (16.6-37.1)
Jhalawar	39.1 (27.9-54.6)	29.9 (19.9-44.2)	23.3 (16.1-33.0)	19.1 (12.2-28.6)
Jhunjhunu	21.8 (15.7-29.7)	17.0 (11.5-24.5)	13.1 (9.3-17.9)	11.0 (7.3-15.8)
Jodhpur	30.6 (21.5-42.0)	24.9 (16.3-35.9)	18.3 (12.6-25.4)	15.9 (10.2-23.6)
Karauli	49.3 (36.1-65.1)	39.7 (27.3-55.1)	28.9 (21.0-38.6)	25.0 (17.1-35.1)
Kota	36.6 (26.6-49.6)	27.9 (19.0-39.7)	21.5 (15.5-29.3)	17.6 (11.9-25.2)
Nagaur	24.3 (17.3-33.9)	19.6 (13.0-28.8)	14.7 (10.2-20.4)	12.6 (8.2-18.7)
Pali	41.6 (30.6-55.9)	32.5 (22.1-45.9)	24.5 (17.7-33.4)	20.4 (13.7-29.7)
Pratapgarh	32.1 (22.7-44.8)	24.4 (16.1-36.2)	18.7 (13.0-26.1)	15.2 (9.8-22.5)
Rajsamand	42.6 (31.1-57.7)	32.5 (22.6-46.5)	24.8 (17.9-33.6)	20.2 (13.7-29.2)
Sawai Madhopur	52.9 (38.1-71.8)	42.1 (28.5-60.3)	30.8 (21.8-41.7)	26.4 (17.4-37.7)
Sikar	20.3 (14.5-27.9)	16.0 (10.6-23.2)	12.2 (8.6-16.9)	10.3 (6.7-15.1)
Sirohi	47.7 (34.2-66.0)	37.7 (25.1-55.2)	27.7 (19.5-38.1)	23.3 (15.2-34.5)
Sri Ganganagar	26.7 (18.8-37.9)	22.0 (14.3-32.8)	16.3 (11.4-22.9)	14.3 (9.3-21.4)
Tonk	43.6 (31.2-59.5)	34.2 (22.9-49.1)	25.6 (18.1-34.7)	21.5 (14.2-30.8)
Udaipur	46.4 (33.6-63.6)	35.8 (24.2-52.3)	26.6 (19.2-36.8)	22.0 (14.8-32.3)
**Chhattisgarh**	**34.1 (25.0-45.4)**	**26.5 (18.3-37.3)**	**22.2 (16.3-29.5)**	**18.4 (12.6-26.0)**

Balod	32.7 (22.8-45.3)	25.4 (16.4-37.4)	21.5 (14.8-30.2)	17.8 (11.5-26.6)
Baloda Bazar	32.5 (23.2-44.3)	25.1 (16.6-36.3)	21.4 (15.3-29.4)	17.6 (11.7-25.9)
Balrampur	36.9 (26.9-49.7)	28.5 (19.6-40.6)	23.5 (17.1-32.4)	19.5 (13.2-28.3)
Bastar	38.7 (27.2-53.0)	31.2 (20.6-45.2)	24.1 (16.6-33.5)	20.6 (13.1-30.7)
Bemetara	35.4 (24.2-50.2)	27.6 (17.5-41.0)	23.2 (15.8-33.3)	19.3 (12.0-29.5)
Bijapur	36.2 (25.3-51.3)	29.6 (19.0-44.6)	22.9 (15.8-32.7)	19.8 (12.6-30.1)
Bilaspur	33.9 (23.7-45.8)	26.2 (17.0-37.3)	22.2 (15.5-30.3)	18.3 (12.0-26.6)
Dantewada	36.5 (24.8-51.5)	30.1 (18.9-44.9)	22.7 (15.4-32.6)	19.8 (12.2-29.9)
Dhamtari	30.3 (21.0-42.0)	23.4 (15.1-34.4)	19.9 (13.7-27.7)	16.3 (10.3-24.3)
Durg	28.7 (19.9-40.0)	21.9 (14.1-32.5)	18.9 (13.0-26.3)	15.4 (9.8-22.9)
Gariaband	36.8 (26.1-50.2)	28.7 (19.1-41.6)	23.6 (16.4-32.6)	19.6 (12.8-28.7)
Janjgir-Champa	31.8 (22.4-43.8)	24.2 (15.9-35.8)	20.9 (14.6-29.1)	17.0 (11.1-25.2)
Jashpur	31.4 (22.4-43.4)	23.8 (15.9-34.7)	20.2 (14.4-27.9)	16.3 (10.9-23.8)
Kabirdham	39.1 (26.4-56.2)	30.5 (19.1-46.6)	25.5 (17.1-37.0)	21.2 (13.1-33.1)
Kondagaon	47.4 (34.2-65.3)	38.2 (25.8-56.0)	30.2 (21.2-41.7)	25.8 (16.9-38.1)
Korba	32.5 (22.7-44.0)	25.0 (16.1-35.7)	21.4 (14.7-29.4)	17.6 (11.2-25.7)
Korea	36.9 (25.9-50.3)	28.7 (18.9-41.5)	23.7 (16.5-33.2)	19.7 (12.7-29.4)
Mahasamund	31.3 (22.2-43.0)	24.0 (15.9-34.8)	20.5 (14.3-29.0)	16.7 (11.0-25.0)
Mungeli	36.5 (25.2-51.0)	28.5 (18.2-42.0)	23.9 (16.2-34.1)	19.9 (12.4-30.3)
Narayanpur	51.4 (35.8-73.3)	42.3 (27.7-64.3)	32.9 (22.6-47.2)	28.8 (18.5-43.8)
North Bastar Kanker	42.1 (30.1-59.1)	33.9 (22.5-50.7)	27.4 (19.1-38.5)	23.4 (15.2-35.1)
Raigarh	30.4 (22.1-41.9)	23.0 (15.7-33.9)	19.8 (14.2-27.4)	16.0 (10.8-23.5)
Raipur	28.7 (19.9-40.1)	22.0 (14.2-32.7)	18.9 (13.1-26.3)	15.4 (9.9-22.7)
Rajnandgaon	34.6 (24.2-48.6)	27.2 (17.8-40.5)	22.7 (15.5-32.0)	19.0 (12.1-28.6)
Sukma	42.2 (28.8-59.5)	34.3 (21.6-51.7)	25.7 (17.2-36.6)	22.1 (13.7-33.8)
Surajpur	36.0 (26.1-48.8)	27.7 (18.8-39.5)	23.1 (16.4-31.9)	19.0 (12.6-27.8)
Surguja	32.2 (23.4-43.7)	24.6 (16.7-35.3)	20.8 (14.8-28.4)	17.0 (11.4-24.4)
**Odisha**	**34.9 (25.8-45.6)**	**27.2 (18.9-37.2)**	**18.8 (13.8-24.8)**	**15.3 (10.6-21.1)**
Angul	37.4 (25.8-51.9)	28.6 (18.2-41.3)	20.3 (14.0-28.4)	16.2 (10.3-24.2)
Balangir	42.7 (30.6-59.8)	34.1 (22.7-50.8)	23.9 (16.7-34.1)	20.0 (12.9-30.4)
Balasore	25.6 (17.3-35.9)	19.3 (12.0-28.7)	13.5 (9.1-19.3)	10.6 (6.6-16.1)
Bargarh	36.4 (26.1-51.4)	28.7 (19.2-43.0)	20.6 (14.5-29.4)	17.0 (11.1-25.9)
Bhadrak	26.8 (18.5-37.2)	19.8 (12.7-29.2)	14.1 (9.5-19.8)	10.9 (6.8-16.2)
Boudh	40.7 (28.2-57.4)	31.7 (20.4-47.9)	22.4 (15.3-31.8)	18.3 (11.5-27.7)
Cuttack	21.0 (14.6-29.3)	15.1 (9.8-22.4)	11.1 (7.7-15.6)	8.4 (5.3-12.6)
Deogarh	41.2 (28.6-56.4)	32.1 (20.7-46.3)	22.4 (15.4-31.2)	18.3 (11.7-27.0)
Dhenkanal	30.4 (21.0-42.0)	22.5 (14.4-33.1)	16.2 (11.0-22.5)	12.6 (7.9-18.6)
Gajapati	43.6 (29.5-61.0)	34.5 (21.7-51.2)	22.7 (15.2-32.9)	18.8 (11.5-29.2)
Ganjam	37.0 (24.9-51.5)	28.2 (17.5-41.6)	19.4 (13.1-27.5)	15.5 (9.6-23.2)
Jagatsinghapur	19.2 (13.1-27.1)	13.8 (8.7-20.7)	10.1 (6.8-14.5)	7.5 (4.6-11.5)
Jajapur	27.9 (19.7-38.4)	20.4 (13.3-29.8)	14.7 (10.2-20.4)	11.3 (7.2-16.6)
Jharsuguda	32.7 (22.9-45.7)	25.5 (16.7-37.6)	18.3 (12.8-25.7)	15.0 (9.8-22.5)
Kalahandi	54.8 (38.2-75.3)	44.8 (29.2-65.3)	30.0 (20.7-41.5)	25.6 (16.5-38.0)
Kandhamal	45.7 (31.9-63.1)	36.0 (23.4-52.7)	24.7 (17.0-34.7)	20.3 (13.0-30.2)
Kendrapara	23.6 (16.2-33.0)	17.2 (10.8-25.7)	12.4 (8.2-17.8)	9.4 (5.7-14.5)
Kendujhar	39.4 (27.5-53.4)	30.6 (19.8-43.8)	21.0 (14.7-28.9)	17.2 (11.1-25.0)
Khordha	19.3 (13.2-26.7)	13.9 (8.7-20.3)	10.2 (6.8-14.5)	7.6 (4.7-11.5)
Koraput	57.5 (40.4-79.8)	47.8 (31.3-70.0)	30.3 (20.8-42.6)	26.4 (16.9-39.7)
Malkangiri	57.5 (38.9-80.8)	48.8 (30.5-72.9)	30.2 (20.3-43.8)	26.8 (16.4-41.6)
Mayurbhanj	32.4 (22.8-45.0)	25.4 (16.6-37.2)	17.2 (12.0-23.8)	14.2 (9.2-20.6)
Nabarangpur	55.1 (38.8-75.8)	45.9 (30.3-66.5)	30.1 (21.1-41.7)	26.3 (17.1-38.5)
Nayagarh	29.1 (20.4-39.9)	21.8 (14.2-31.6)	15.5 (10.8-21.4)	12.2 (7.9-17.8)
Nuapada	45.3 (31.9-62.7)	37.0 (24.5-54.2)	25.5 (17.9-35.8)	21.9 (14.2-32.9)
Puri	18.7 (12.6-26.1)	13.5 (8.4-20.2)	9.8 (6.5-13.9)	7.4 (4.5-11.2)
Rayagada	60.0 (41.4-81.9)	49.0 (31.4-70.9)	31.7 (21.9-44.3)	27.1 (17.2-39.9)
Sambalpur	35.5 (25.1-49.0)	27.7 (18.3-40.6)	19.7 (13.7-27.6)	16.2 (10.5-24.2)
Sonepur	38.5 (26.9-54.0)	30.1 (19.6-44.8)	21.5 (14.7-30.5)	17.6 (11.2-26.8)
Sundargarh	38.2 (27.4-52.2)	29.8 (20.0-43.1)	21.0 (15.1-28.7)	17.2 (11.5-24.8)

**Assam**	**43.8 (32.9-56.7)**	**36.7 (26.0-49.8)**	**23.8 (17.5-31.0)**	**20.6 (14.4-28.4)**
Baksa	29.4 (21.2-39.8)	22.3 (15.1-31.9)	16.1 (11.4-22.0)	12.7 (8.4-18.3)
Barpeta	26.3 (18.4-36.6)	20.0 (13.1-29.8)	14.6 (10.2-20.5)	11.5 (7.5-17.3)
Biswanath	39.3 (28.3-52.7)	33.0 (22.3-46.8)	20.9 (14.8-28.6)	18.1 (12.0-26.4)
Bongaigaon	26.2 (18.9-35.8)	19.9 (13.3-28.9)	14.8 (10.4-20.8)	11.6 (7.7-17.5)
Cachar	72.2 (51.6-100.8)	64.2 (42.4-95.8)	39.3 (27.8-54.6)	36.0 (23.6-53.5)
Charaideo	57.6 (39.1-80.1)	51.4 (32.1-75.7)	29.8 (20.1-41.8)	27.3 (17.3-40.9)
Chirang	25.8 (18.7-35.1)	19.5 (13.3-28.2)	14.4 (10.2-19.9)	11.3 (7.5-16.7)
Darrang	41.5 (30.1-55.4)	33.3 (22.5-46.8)	22.8 (16.4-31.0)	19.0 (12.6-27.2)
Dhemaji	40.7 (28.4-56.4)	34.5 (22.4-51.1)	21.3 (14.6-29.9)	18.6 (11.6-27.8)
Dhubri	30.5 (22.2-42.5)	23.1 (15.7-34.2)	17.8 (12.7-25.5)	14.0 (9.4-21.2)
Dibrugarh	47.8 (33.1-66.4)	41.7 (26.9-61.9)	24.8 (17.0-34.7)	22.2 (14.1-33.3)
Dima Hasao	70.3 (49.9-94.9)	62.7 (41.8-89.9)	38.3 (27.4-52.3)	35.3 (23.6-50.4)
Goalpara	27.1 (19.7-37.1)	20.6 (13.9-29.9)	15.4 (11.0-21.4)	12.2 (8.1-18.2)
Golaghat	50.8 (36.3-69.5)	44.1 (29.3-63.8)	27.1 (19.3-37.5)	24.2 (16.0-36.0)
Hailakandi	74.0 (52.4-103.6)	65.7 (43.3-98.0)	40.1 (28.2-56.2)	36.7 (24.0-55.1)
Hojai	63.9 (45.2-86.9)	55.5 (36.8-79.8)	35.1 (24.9-48.6)	31.5 (20.6-46.0)
Jorhat	51.9 (36.0-72.0)	44.6 (28.8-66.2)	27.7 (19.2-38.8)	24.5 (15.8-36.6)
Kamrup	28.0 (19.9-37.8)	21.5 (14.2-30.8)	15.5 (10.8-21.1)	12.3 (8.0-17.9)
Kamrup Metropolitan	27.0 (19.3-36.4)	20.9 (13.9-29.7)	14.9 (10.3-20.1)	12.0 (7.7-17.2)
Karbi Anglong	56.2 (40.4-76.0)	49.0 (32.9-70.0)	30.4 (21.7-41.3)	27.3 (18.2-39.2)
Karimganj	72.8 (51.4-101.9)	63.9 (41.7-95.2)	40.0 (28.2-56.1)	36.1 (23.7-54.1)
Kokrajhar	28.3 (20.5-39.1)	21.3 (14.4-31.1)	16.2 (11.7-22.7)	12.7 (8.5-18.8)
Lakhimpur	41.8 (29.3-57.5)	35.2 (22.9-51.3)	22.1 (15.5-30.8)	19.2 (12.5-28.1)
Majuli	49.8 (34.9-67.9)	42.4 (27.8-61.6)	26.5 (18.5-36.7)	23.3 (15.1-34.0)
Morigaon	50.7 (36.7-67.4)	42.1 (28.5-58.8)	27.9 (20.0-37.8)	24.0 (16.0-34.2)
Nagaon	53.3 (38.4-71.7)	45.3 (30.6-64.4)	29.1 (20.7-39.3)	25.6 (16.9-36.6)
Nalbari	26.5 (18.8-36.5)	20.1 (13.3-29.6)	14.6 (10.3-20.2)	11.5 (7.5-16.9)
Sivasagar	53.3 (36.5-73.6)	46.4 (29.6-67.7)	28.1 (19.4-39.3)	25.2 (16.0-37.6)
Sonitpur	39.3 (28.8-52.7)	32.3 (22.2-45.7)	21.3 (15.3-28.9)	18.1 (12.1-25.9)
South Salmara Mancachar	29.4 (21.2-41.5)	22.2 (15.0-33.3)	17.2 (12.2-24.4)	13.5 (8.9-20.5)
Tinsukia	41.8 (28.0-58.8)	36.6 (22.7-55.2)	21.0 (13.8-30.1)	18.9 (11.4-28.9)
Udalguri	38.9 (28.0-52.4)	30.9 (20.8-43.4)	21.2 (15.1-28.9)	17.4 (11.6-25.0)
West Karbi Anglong	61.1 (43.5-82.8)	53.1 (35.2-76.2)	33.7 (23.7-46.2)	30.3 (19.9-43.5)
**Andhra Pradesh**	**24.3 (17.3-33.3)**	**18.2 (12.1-26.6)**	**14.5 (10.2-19.9)**	**11.6 (7.5-17.0)**
Anantapur	28.7 (18.7-42.3)	21.4 (12.7-33.8)	17.0 (10.9-25.3)	13.5 (7.9-21.5)
Chittoor	20.9 (13.4-30.8)	15.5 (9.1-24.5)	12.1 (7.4-18.4)	9.6 (5.3-15.5)
East Godavari	20.8 (13.6-30.7)	15.7 (9.4-25.0)	12.2 (7.8-18.2)	9.9 (5.8-16.0)
Guntur	20.5 (13.2-31.0)	15.4 (9.0-25.2)	12.5 (7.8-19.0)	10.1 (5.7-16.6)
Krishna	19.3 (12.2-28.9)	14.2 (8.2-22.8)	11.6 (7.2-17.5)	9.2 (5.2-15.1)
Kurnool	32.6 (21.2-48.3)	24.7 (14.7-38.9)	19.7 (12.6-29.2)	16.0 (9.3-25.3)
Prakasam	24.8 (15.9-37.5)	19.2 (11.2-31.5)	15.0 (9.5-22.8)	12.5 (7.2-20.4)
Sri Potti Sriramulu Nellore	23.4 (14.8-35.2)	17.9 (10.2-28.8)	13.8 (8.5-21.1)	11.3 (6.2-18.5)
Srikakulam	24.7 (16.4-35.5)	17.6 (10.8-27.1)	14.6 (9.5-21.5)	11.3 (6.7-17.6)
Visakhapatnam	24.9 (16.5-36.3)	18.6 (11.2-29.1)	14.7 (9.6-21.8)	11.8 (7.1-18.7)
Vizianagaram	31.6 (20.5-46.1)	23.2 (13.8-36.5)	18.8 (12.0-28.0)	14.9 (8.6-23.6)
West Godavari	21.5 (13.8-32.6)	16.1 (9.5-26.4)	12.8 (8.1-19.8)	10.3 (6.0-17.2)
YSR	27.5 (17.7-40.5)	20.9 (12.2-33.3)	16.4 (10.3-24.5)	13.3 (7.5-21.4)
**West Bengal**	**19.7 (14.6-26.3)**	**14.8 (10.3-20.8)**	**13.0 (9.4-17.5)**	**10.5 (7.1-14.9)**
Alipurduar	19.2 (14.0-27.0)	14.1 (9.6-21.0)	11.9 (8.6-17.0)	9.4 (6.4-14.3)
Bankura	22.7 (16.0-31.7)	17.5 (11.4-26.0)	15.2 (10.7-21.4)	12.6 (8.2-19.0)
Birbhum	26.4 (18.9-36.2)	20.2 (13.4-29.5)	17.8 (12.7-24.6)	14.7 (9.8-21.6)
Cooch Behar	22.6 (16.4-31.9)	16.8 (11.4-25.0)	14.3 (10.3-20.4)	11.5 (7.7-17.3)
Dakshin Dinajpur	23.1 (16.6-32.7)	17.4 (11.7-26.0)	15.4 (11.0-22.4)	12.5 (8.3-19.3)
Darjeeling	19.5 (13.7-27.1)	14.3 (9.4-21.2)	12.6 (8.7-18.0)	10.0 (6.4-15.2)
Hooghly	16.1 (11.0-22.8)	11.9 (7.5-17.9)	10.6 (7.0-15.1)	8.4 (5.1-12.8)
Howrah	13.5 (8.8-19.7)	9.8 (5.9-15.3)	8.8 (5.6-12.8)	6.9 (4.0-10.7)
Jalpaiguri	20.2 (14.8-28.1)	15.0 (10.2-22.1)	12.9 (9.2-18.3)	10.4 (7.0-15.6)

Jhargram	21.8 (15.0-30.3)	16.6 (10.6-24.5)	14.0 (9.6-19.8)	11.6 (7.4-17.3)
Kalimpong	14.8 (10.6-20.4)	10.8 (7.3-15.8)	9.3 (6.6-13.0)	7.3 (4.9-11.0)
Kolkata	12.3 (8.0-17.8)	9.0 (5.4-13.9)	8.0 (5.2-11.8)	6.3 (3.7-9.9)
Maldah	28.5 (20.5-39.6)	21.7 (14.7-31.9)	19.1 (13.6-26.9)	15.7 (10.4-23.6)
Murshidabad	23.8 (16.7-33.5)	17.9 (11.7-26.9)	16.0 (11.2-22.7)	12.9 (8.4-19.6)
Nadia	18.6 (12.7-26.3)	13.8 (8.8-21.0)	12.4 (8.2-17.8)	9.8 (6.0-15.1)
North 24 Parganas	15.6 (10.4-22.2)	11.5 (7.1-17.4)	10.2 (6.6-14.6)	8.0 (4.8-12.3)
Paschim Burdwan	22.9 (16.2-31.7)	17.6 (11.6-25.9)	15.6 (10.9-21.7)	12.9 (8.4-19.1)
Pashchim Medinipur	19.2 (13.0-26.8)	14.4 (9.0-21.4)	12.5 (8.4-17.7)	10.1 (6.2-15.2)
Purba Burdwan	19.8 (13.4-27.9)	14.9 (9.4-22.4)	13.2 (9.0-18.7)	10.8 (6.7-16.2)
Purba Medinipur	16.5 (11.0-23.7)	12.2 (7.5-18.6)	10.6 (7.0-15.3)	8.4 (5.1-12.9)
Puruliya	27.8 (20.3-38.1)	21.5 (14.8-31.7)	18.5 (13.5-25.6)	15.5 (10.6-22.8)
South 24 Parganas	16.0 (10.5-23.1)	11.8 (7.1-18.2)	10.3 (6.7-15.0)	8.2 (4.8-12.7)
Uttar Dinajpur	28.3 (20.0-39.0)	21.8 (14.4-31.8)	18.8 (13.3-26.3)	15.7 (10.3-23.2)
**Tripura**	**25.4 (17.7-35.5)**	**20.3 (13.0-30.3)**	**14.6 (10.1-20.4)**	**12.2 (7.8-18.3)**
Dhalai	33.7 (22.9-47.7)	27.9 (17.6-42.4)	18.9 (12.9-27.1)	16.4 (10.3-25.2)
Gomati	24.0 (16.6-33.7)	19.2 (12.3-28.7)	13.6 (9.3-19.2)	11.4 (7.2-17.3)
Khowai	25.7 (17.6-36.1)	20.2 (12.8-30.2)	14.9 (10.2-21.2)	12.2 (7.6-18.4)
North Tripura	40.5 (28.0-57.4)	34.1 (21.9-51.8)	22.8 (15.7-32.5)	20.1 (12.7-30.7)
Sepahijala	20.2 (14.0-28.3)	15.6 (10.0-23.2)	11.9 (8.1-16.9)	9.7 (6.0-14.6)
South Tripura	22.0 (15.1-31.1)	17.6 (11.0-26.3)	12.6 (8.5-18.2)	10.5 (6.6-16.3)
Unakoti	34.1 (23.8-48.4)	28.0 (18.1-42.5)	19.6 (13.3-27.9)	16.9 (10.6-25.6)
West Tripura	19.3 (13.2-27.2)	14.6 (9.2-21.8)	11.4 (7.7-16.2)	9.0 (5.6-13.7)
**Arunachal Pradesh**	**18.6 (12.6-25.7)**	**13.7 (8.5-20.1)**	**10.8 (7.3-15.2)**	**8.6 (5.4-13.1)**
Anjaw	16.5 (9.6-25.7)	12.1 (6.3-20.5)	9.5 (5.4-15.3)	7.7 (3.9-13.3)
Changlang	20.9 (13.6-29.7)	15.5 (9.2-23.7)	12.0 (7.6-17.6)	9.8 (5.6-15.4)
Dibang Valley	9.8 (6.0-15.4)	6.8 (3.7-11.5)	5.7 (3.4-9.1)	4.4 (2.3-7.4)
East Kameng	12.2 (8.4-17.3)	8.0 (5.1-12.0)	7.5 (5.1-10.6)	5.5 (3.4-8.2)
East Siang	15.0 (9.9-21.4)	10.9 (6.6-16.8)	8.8 (5.7-12.7)	6.9 (4.1-10.8)
Kamle	18.3 (12.7-25.5)	13.7 (8.8-20.3)	10.4 (7.1-14.5)	8.2 (5.2-12.2)
Kra Daddi	11.3 (7.5-16.2)	7.5 (4.5-11.5)	6.8 (4.4-9.8)	5.0 (2.9-7.7)
Kurung Kumey	12.4 (8.4-17.6)	8.0 (5.0-12.2)	7.5 (5.0-10.7)	5.4 (3.3-8.2)
Lohit	16.4 (10.5-23.9)	12.1 (7.1-18.9)	9.4 (6.0-14.0)	7.5 (4.3-12.2)
Longding	25.1 (16.9-35.1)	18.8 (11.7-27.8)	14.7 (9.7-21.1)	12.1 (7.4-18.5)
Lower Dibang Valley	12.6 (8.0-18.6)	8.9 (5.1-14.1)	7.4 (4.7-11.3)	5.7 (3.3-9.5)
Lower Siang	24.8 (17.1-34.5)	19.5 (12.4-29.1)	13.7 (9.2-19.4)	11.3 (6.9-17.2)
Lower Subansiri	11.4 (7.9-16.0)	7.6 (4.8-11.3)	7.1 (4.8-9.8)	5.2 (3.3-7.7)
Namsai	20.0 (13.1-28.4)	15.2 (9.1-23.2)	11.2 (7.3-16.4)	9.2 (5.4-14.3)
Pakke Kessang	13.8 (9.8-18.9)	9.6 (6.4-14.0)	8.4 (5.9-11.6)	6.3 (4.1-9.3)
Papum Pare	27.6 (19.9-37.7)	22.1 (14.8-32.0)	15.2 (10.8-20.8)	12.6 (8.4-18.3)
Shi Yomi	10.8 (7.4-15.5)	7.2 (4.5-11.0)	6.6 (4.4-9.6)	4.9 (2.9-7.5)
Siang	8.4 (5.3-12.8)	5.6 (3.2-9.1)	5.0 (3.1-7.5)	3.7 (2.1-5.8)
Tawang	11.2 (8.2-15.1)	7.2 (4.9-10.3)	6.9 (4.9-9.4)	5.0 (3.3-7.1)
Tirap	23.5 (15.5-33.9)	17.3 (10.4-26.3)	13.8 (9.0-20.3)	11.2 (6.7-17.6)
Upper Siang	8.3 (5.0-13.0)	5.5 (3.0-9.3)	4.9 (2.9-7.7)	3.6 (1.9-6.1)
Upper Subansiri	10.6 (6.9-15.3)	7.0 (4.2-10.8)	6.3 (4.0-9.2)	4.6 (2.7-7.3)
West Kameng	13.6 (10.0-18.2)	9.3 (6.4-13.1)	8.5 (6.1-11.3)	6.3 (4.2-8.9)
West Siang	9.1 (5.8-13.9)	6.1 (3.5-9.9)	5.4 (3.3-8.3)	4.0 (2.3-6.6)
**Meghalaya**	**30.2 (21.9-40.7)**	**24.8 (16.9-35.3)**	**16.0 (11.5-21.7)**	**14.2 (9.6-20.4)**
East Garo Hills	21.3 (15.3-30.2)	16.6 (11.2-25.0)	11.4 (8.1-16.1)	9.6 (6.3-14.5)
East Jaintia Hills	66.6 (47.7-91.7)	60.2 (40.1-87.1)	34.8 (24.6-47.4)	33.9 (22.5-49.1)
East Khasi Hills	29.0 (20.6-39.2)	23.5 (15.7-33.8)	15.2 (10.8-20.6)	13.3 (8.8-19.4)
North Garo Hills	22.8 (16.4-31.9)	17.8 (11.9-26.6)	12.2 (8.7-17.2)	10.4 (6.8-15.4)
Ri Bhoi	34.2 (24.5-45.2)	28.5 (19.1-39.9)	17.7 (12.5-23.8)	16.0 (10.6-22.8)
South Garo Hills	18.5 (13.2-26.3)	13.8 (9.1-21.0)	10.3 (7.2-14.7)	8.3 (5.4-12.8)
South West Garo Hills	23.3 (16.4-33.4)	17.6 (11.6-27.5)	13.2 (9.3-19.2)	10.8 (7.1-17.1)
South West Khasi Hills	25.9 (18.2-35.6)	20.3 (13.1-29.8)	14.5 (10.0-20.0)	12.2 (7.9-18.0)
West Garo Hills	22.9 (16.5-32.3)	17.6 (11.9-26.5)	12.5 (8.8-17.8)	10.4 (6.9-15.9)

West Jaintia Hills	48.3 (34.4-65.6)	42.4 (28.2-61.2)	25.1 (17.9-34.2)	23.8 (15.9-34.3)
West Khasi Hills	26.3 (18.8-36.0)	21.2 (14.1-31.0)	13.8 (9.7-19.1)	12.0 (7.9-17.8)
**Karnataka**	**22.3 (15.4-31.1)**	**17.2 (11.0-25.6)**	**13.4 (9.2-18.7)**	**10.8 (6.9-16.2)**
Bagalkot	25.5 (16.3-38.1)	19.3 (11.3-31.1)	15.5 (10.0-22.9)	12.3 (7.3-19.8)
Ballari	33.1 (21.5-49.2)	25.9 (15.4-41.2)	20.0 (12.8-30.5)	16.4 (9.6-26.8)
Belagavi	19.4 (12.5-28.9)	14.8 (8.7-23.7)	11.8 (7.5-17.5)	9.4 (5.5-15.1)
Bengaluru Rural	23.4 (14.7-34.8)	18.2 (10.4-29.3)	13.8 (8.7-20.5)	11.2 (6.4-17.8)
Bengaluru Urban	19.1 (11.9-28.6)	14.7 (8.3-23.8)	11.2 (7.0-16.9)	9.0 (5.1-14.6)
Bidar	20.6 (13.9-29.3)	16.3 (10.0-24.7)	13.1 (8.6-18.7)	11.0 (6.6-16.8)
Chamarajanagar	22.4 (13.7-34.0)	17.4 (9.6-28.3)	13.1 (7.9-19.9)	10.6 (5.8-17.2)
Chikballapur	26.4 (16.8-38.0)	21.0 (12.1-32.6)	15.6 (9.7-23.2)	13.0 (7.3-20.5)
Chikkamagaluru	16.9 (10.7-25.8)	12.7 (7.3-20.7)	10.0 (6.2-15.3)	7.9 (4.4-12.8)
Chitradurga	26.1 (16.7-38.9)	20.1 (11.6-32.2)	15.6 (9.7-23.4)	12.6 (7.1-20.3)
Dakshina Kannada	8.9 (5.2-13.8)	6.6 (3.5-11.1)	5.2 (3.0-8.2)	4.0 (2.1-6.9)
Davanagere	24.8 (15.7-36.9)	18.8 (10.8-29.6)	14.8 (9.2-22.1)	11.8 (6.7-18.9)
Dharwad	24.1 (15.2-37.2)	18.3 (10.6-30.2)	14.6 (9.1-22.6)	11.6 (6.6-19.5)
Gadag	31.3 (19.8-47.1)	23.9 (13.7-39.4)	18.9 (11.9-29.1)	15.1 (8.8-25.4)
Hassan	18.0 (11.0-27.2)	13.5 (7.4-21.9)	10.6 (6.4-16.0)	8.3 (4.5-13.6)
Haveri	26.1 (16.2-39.7)	19.9 (11.3-32.7)	15.7 (9.9-24.2)	12.5 (7.1-20.9)
Kalaburgi	24.5 (16.3-35.5)	19.5 (11.8-30.4)	15.2 (9.9-22.3)	12.8 (7.7-20.0)
Kodagu	12.5 (7.4-18.9)	9.4 (5.0-15.4)	7.4 (4.3-11.2)	5.8 (3.0-9.7)
Kolar	22.5 (14.1-33.9)	17.6 (10.0-28.7)	13.1 (8.2-20.0)	10.8 (6.0-17.6)
Koppal	33.7 (21.7-50.2)	25.9 (15.3-41.8)	20.4 (13.1-31.0)	16.5 (9.8-27.0)
Mandya	21.8 (13.5-31.8)	16.6 (9.3-26.1)	12.8 (7.9-19.0)	10.2 (5.6-16.2)
Mysuru	19.0 (11.7-28.3)	14.4 (8.0-23.0)	11.2 (6.8-16.6)	8.8 (4.8-14.1)
Raichur	33.1 (21.8-48.5)	26.2 (16.0-41.2)	20.3 (13.2-29.8)	16.9 (10.0-26.9)
Ramanagara	23.2 (14.3-34.9)	17.8 (10.0-28.8)	13.6 (8.5-20.5)	10.9 (6.2-17.8)
Shivamogga	17.6 (11.0-26.1)	13.3 (7.6-21.1)	10.5 (6.4-15.7)	8.3 (4.6-13.3)
Tumakuru	24.9 (16.0-37.0)	19.3 (11.2-30.9)	14.8 (9.2-21.7)	12.0 (6.7-19.0)
Udupi	11.0 (6.5-16.9)	8.3 (4.4-13.8)	6.4 (3.8-10.1)	5.1 (2.7-8.7)
Uttara Kannada	18.4 (11.3-28.2)	14.2 (8.0-23.4)	11.0 (6.7-16.8)	8.9 (4.8-14.7)
Vijaypura	23.3 (15.4-33.5)	18.1 (11.0-27.8)	14.3 (9.2-20.6)	11.7 (6.8-18.1)
Yadgir	30.1 (19.8-43.7)	24.0 (14.5-37.2)	18.5 (11.9-27.1)	15.6 (9.2-24.6)
**Telangana**	**18.2 (12.5-25.9)**	**13.2 (8.5-20.1)**	**12.4 (8.4-17.9)**	**9.8 (6.2-15.2)**
Adilabad	22.1 (14.4-32.4)	17.0 (10.2-26.3)	15.5 (10.0-22.9)	13.1 (7.8-20.7)
Bhadradri Kothagudem	25.4 (16.4-36.8)	18.9 (11.3-29.4)	16.8 (10.7-24.5)	13.7 (8.0-21.3)
Hyderabad	13.7 (8.9-20.2)	9.6 (5.7-15.5)	9.3 (6.0-14.0)	7.1 (4.2-11.6)
Jagitial	18.3 (12.3-26.9)	13.5 (8.4-21.1)	12.8 (8.5-18.8)	10.3 (6.3-16.3)
Jangoan	16.4 (10.7-24.3)	11.6 (7.0-18.8)	11.2 (7.2-16.9)	8.7 (5.2-14.0)
Jayashankar Bhupalpally	21.1 (14.0-31.6)	15.8 (9.6-25.6)	14.5 (9.4-21.6)	11.9 (7.1-18.9)
Jogulamba Gadwal	26.0 (17.0-37.9)	18.8 (11.3-29.6)	17.0 (11.0-25.2)	13.3 (7.9-21.4)
Kamareddy	18.3 (11.8-26.2)	13.2 (7.9-20.3)	12.6 (8.0-18.3)	10.0 (5.8-15.4)
Karimnagar	16.2 (10.7-23.6)	11.7 (7.1-18.1)	11.2 (7.4-16.4)	8.8 (5.3-13.9)
Khammam	20.3 (12.9-30.2)	14.9 (8.7-23.7)	13.4 (8.3-20.2)	10.7 (6.1-17.3)
Kumuram Bheem Asifabad	21.5 (14.1-31.8)	16.4 (9.8-26.4)	15.1 (9.6-23.0)	12.6 (7.4-20.5)
Mahabubnagar	22.0 (14.5-31.9)	15.8 (9.5-24.6)	14.6 (9.5-21.1)	11.4 (6.8-17.6)
Mahuababad	18.8 (12.2-27.8)	13.7 (8.2-22.1)	12.7 (8.1-19.0)	10.1 (5.9-16.3)
Mancherial	19.4 (12.6-28.9)	14.5 (8.7-22.9)	13.6 (8.7-20.2)	11.1 (6.4-17.8)
Medak	17.8 (11.5-26.4)	12.7 (7.6-20.0)	12.2 (7.7-18.3)	9.5 (5.4-15.1)
Medchal Malkajgiri	13.7 (9.0-20.2)	9.5 (5.8-15.1)	9.3 (6.0-13.9)	7.1 (4.2-11.4)
Nagarkurnool	24.1 (15.2-36.3)	17.6 (10.2-28.4)	16.0 (9.8-24.5)	12.7 (7.2-20.7)
Nalgonda	19.1 (12.0-28.6)	13.8 (8.1-22.4)	12.8 (8.0-19.6)	10.1 (5.8-16.6)
Nirmal	20.8 (13.7-30.0)	15.8 (9.7-24.5)	14.5 (9.4-21.3)	12.1 (7.2-19.3)
Nizamabad	18.4 (12.2-26.3)	13.5 (8.3-20.5)	12.8 (8.3-18.3)	10.3 (6.1-15.7)
Peddapalli	17.2 (11.2-25.4)	12.5 (7.5-19.7)	11.9 (7.7-17.7)	9.5 (5.7-15.3)
Rajanna Sircilla	17.3 (11.4-25.3)	12.5 (7.5-19.2)	12.0 (7.8-17.5)	9.5 (5.7-14.8)
Rangareddy	15.8 (10.2-23.3)	11.1 (6.5-17.7)	10.7 (6.8-15.9)	8.2 (4.7-13.2)
Sangareddy	17.4 (11.6-25.1)	12.3 (7.6-19.0)	11.8 (7.7-17.2)	9.1 (5.5-14.1)

Siddipet	16.7 (11.0-24.7)	11.9 (7.2-18.8)	11.5 (7.5-16.9)	9.0 (5.3-14.3)
Suryapet	18.8 (12.2-27.8)	13.7 (8.1-21.9)	12.6 (7.9-18.9)	10.0 (5.7-16.0)
Vikarabad	19.6 (12.9-28.3)	13.9 (8.4-21.7)	13.1 (8.6-18.8)	10.1 (6.0-15.6)
Wanaparthy	24.5 (15.9-36.5)	17.8 (10.6-28.1)	16.1 (10.3-24.2)	12.7 (7.5-20.7)
Warangal Rural	17.2 (11.2-25.6)	12.5 (7.4-20.2)	11.8 (7.6-17.8)	9.3 (5.5-15.3)
Warangal Urban	15.0 (9.8-22.1)	10.7 (6.4-17.1)	10.3 (6.6-15.4)	8.0 (4.8-12.9)
Yadadri Bhuvanagiri	16.7 (10.8-25.2)	11.9 (7.0-19.1)	11.4 (7.2-17.5)	8.8 (5.1-14.5)
**Gujarat**	**27.8 (20.2-37.5)**	**21.9 (14.8-31.1)**	**17.9 (12.9-24.2)**	**15.0 (10.2-21.5)**
Ahmedabad	25.9 (17.9-36.6)	20.1 (12.9-30.3)	16.8 (11.3-24.1)	13.9 (8.7-21.2)
Amreli	23.1 (14.5-34.7)	18.4 (10.6-29.6)	14.8 (9.3-22.8)	12.5 (7.1-20.9)
Anand	34.7 (24.0-48.4)	27.1 (17.3-40.2)	22.5 (15.1-31.6)	18.7 (11.6-28.0)
Arvalli	33.6 (23.6-47.1)	25.8 (16.8-38.4)	21.4 (14.8-30.6)	17.6 (11.4-26.9)
Banaskantha	38.5 (27.0-53.1)	30.9 (20.1-45.3)	25.0 (17.1-34.7)	21.4 (13.5-32.0)
Bharuch	29.4 (20.0-41.7)	23.3 (14.6-35.2)	19.0 (12.6-27.0)	16.0 (9.8-24.5)
Bhavnagar	28.4 (19.2-41.3)	23.0 (14.4-35.7)	18.3 (12.2-26.6)	15.7 (9.7-24.4)
Botad	28.8 (19.6-40.7)	23.1 (14.7-35.0)	18.6 (12.4-26.8)	15.9 (9.8-24.5)
Chhotaudepur	38.6 (26.3-53.9)	31.0 (19.4-46.0)	24.6 (16.7-34.7)	21.1 (13.1-31.6)
Dahod	36.1 (24.5-50.2)	28.1 (17.7-41.9)	22.8 (15.3-32.2)	19.1 (11.8-28.8)
Dang	29.4 (19.5-41.9)	24.1 (14.7-36.6)	18.9 (12.6-27.1)	16.5 (10.1-25.3)
Devbhumi Dwarka	20.9 (12.5-33.8)	16.4 (8.7-28.7)	13.3 (7.7-21.6)	11.0 (5.8-19.3)
Gandhinagar	24.7 (16.3-35.5)	18.9 (11.5-29.0)	16.0 (10.4-23.4)	13.1 (7.9-20.6)
Gir Somnath	20.8 (12.9-32.2)	16.3 (9.1-27.4)	13.2 (8.1-20.8)	11.0 (6.1-18.8)
Jamnagar	20.5 (13.2-30.7)	15.9 (9.5-25.7)	13.2 (8.3-19.9)	10.8 (6.3-17.9)
Junagadh	17.5 (11.0-27.2)	13.3 (7.6-22.5)	11.1 (7.0-17.5)	8.9 (5.1-15.2)
Kachchh	31.3 (20.7-46.1)	25.5 (15.8-40.0)	20.2 (13.1-29.6)	17.4 (10.4-27.7)
Kheda	32.7 (22.8-45.3)	25.5 (16.6-37.8)	21.1 (14.5-30.3)	17.6 (11.2-26.9)
Mahesana	27.7 (19.2-38.8)	21.4 (13.8-32.0)	17.9 (12.3-25.5)	14.8 (9.4-22.4)
Mahisagar	33.3 (23.6-46.8)	25.6 (16.9-38.5)	21.2 (14.9-29.8)	17.5 (11.4-26.2)
Morbi	25.9 (17.0-37.7)	20.7 (12.3-32.0)	16.8 (10.7-25.0)	14.3 (8.3-22.9)
Narmada	36.2 (23.8-50.6)	28.9 (17.4-43.3)	23.1 (15.0-33.5)	19.8 (11.8-30.8)
Navsari	20.1 (13.1-29.7)	15.8 (9.5-25.0)	12.9 (8.3-18.9)	10.8 (6.4-16.8)
Panchmahal	33.6 (23.3-46.3)	26.0 (16.8-38.2)	21.5 (14.8-29.8)	17.8 (11.4-26.4)
Patan	34.6 (23.8-47.9)	27.7 (17.5-40.8)	22.5 (15.0-31.4)	19.2 (11.8-28.4)
Porbandar	17.7 (10.8-29.0)	13.5 (7.3-24.1)	11.3 (6.8-18.3)	9.1 (4.9-16.1)
Rajkot	18.9 (12.3-27.5)	14.6 (8.6-22.9)	12.2 (7.9-18.1)	10.0 (5.9-15.9)
Sabar Kantha	32.6 (23.1-45.6)	25.2 (16.5-37.6)	20.9 (14.5-29.7)	17.3 (11.1-26.0)
Surat	20.9 (13.4-31.2)	16.5 (9.7-26.6)	13.4 (8.6-20.1)	11.3 (6.5-18.1)
Surendranagar	28.9 (19.3-40.2)	23.1 (14.3-34.1)	18.7 (12.4-26.3)	16.0 (9.8-23.9)
Tapi	30.1 (20.4-42.3)	24.2 (15.1-36.4)	19.3 (12.9-27.6)	16.6 (10.1-25.4)
Vadodara	31.6 (22.0-44.4)	24.6 (15.8-36.9)	20.4 (13.8-28.5)	16.9 (10.6-25.2)
Valsad	21.6 (13.9-31.6)	17.4 (10.2-27.1)	13.8 (8.9-20.4)	11.8 (6.9-18.6)
**Manipur**	**18.3 (12.5-25.8)**	**14.4 (9.1-21.6)**	**10.7 (7.2-15.1)**	**8.9 (5.6-13.5)**
Bishnupur	17.6 (11.7-25.5)	13.9 (8.5-21.3)	10.3 (6.7-15.0)	8.7 (5.3-13.3)
Chandel	21.2 (14.1-30.9)	17.5 (10.7-27.2)	12.0 (7.9-17.2)	10.5 (6.4-16.2)
Churachandpur	20.5 (13.9-29.1)	16.4 (10.3-24.8)	11.9 (8.1-16.8)	10.2 (6.3-15.3)
Imphal East	15.3 (10.4-21.7)	11.9 (7.4-18.0)	9.0 (6.0-12.8)	7.4 (4.6-11.2)
Imphal West	15.7 (10.6-22.3)	12.2 (7.5-18.6)	9.3 (6.2-13.3)	7.7 (4.7-11.7)
Jiribam	34.7 (24.3-48.4)	27.9 (18.1-41.5)	20.6 (14.4-28.9)	17.4 (11.2-25.9)
Kakching	17.3 (11.3-25.2)	13.9 (8.4-21.7)	10.0 (6.5-14.6)	8.6 (5.1-13.4)
Kamjong	19.7 (13.2-28.5)	15.9 (9.8-24.6)	11.1 (7.4-16.1)	9.6 (5.8-14.8)
Kangpokpi	17.0 (11.6-24.0)	13.3 (8.3-20.0)	9.9 (6.7-14.0)	8.2 (5.1-12.4)
Noney	24.4 (16.8-34.2)	19.1 (12.2-28.7)	14.7 (10.1-20.7)	12.2 (7.7-18.3)
Pherzawl	25.0 (17.4-35.1)	19.8 (12.8-29.7)	14.8 (10.3-20.8)	12.4 (8.0-18.7)
Senapati	19.2 (13.5-26.3)	14.9 (9.7-21.7)	11.3 (7.9-15.6)	9.3 (6.1-13.9)
Tamenglong	24.5 (17.2-33.4)	19.1 (12.5-27.5)	14.7 (10.4-20.3)	12.2 (7.9-17.8)
Tengnoupal	19.5 (12.8-28.4)	15.8 (9.5-24.4)	11.2 (7.2-16.2)	9.6 (5.7-15.1)
Thoubal	16.5 (10.8-23.8)	13.0 (7.9-20.1)	9.5 (6.2-13.9)	8.0 (4.8-12.4)
Ukhrul	18.8 (12.8-26.1)	15.0 (9.4-22.2)	10.6 (7.2-15.0)	9.0 (5.6-13.6)

**Jammu & Kashmir†**	**25.4 (18.4-33.8)**	**20.5 (13.9-28.7)**	**16.0 (11.5-21.4)**	**13.9 (9.3-19.6)**
Anantnag	26.8 (19.1-35.7)	21.8 (14.6-30.5)	16.9 (11.9-22.8)	14.7 (9.6-20.7)
Badgam	25.1 (17.9-34.0)	20.0 (13.5-28.6)	15.8 (11.1-21.6)	13.5 (8.8-19.9)
Bandipore	29.4 (20.5-39.8)	23.5 (15.3-33.9)	18.2 (12.4-25.1)	15.6 (9.8-22.9)
Baramulla	26.5 (18.6-35.8)	21.0 (13.6-30.0)	16.5 (11.3-23.0)	14.0 (8.9-20.6)
Doda	26.6 (18.4-36.7)	22.4 (14.4-32.6)	17.1 (11.7-23.8)	15.5 (9.8-22.9)
Ganderbal	29.5 (20.7-40.4)	23.7 (15.5-34.6)	18.4 (12.7-25.6)	15.8 (10.1-23.3)
Jammu	19.2 (13.5-26.2)	15.3 (10.1-22.1)	12.4 (8.7-17.0)	10.6 (6.9-15.3)
Kargil	34.8 (24.1-47.7)	28.4 (18.5-41.3)	21.4 (14.5-29.7)	18.7 (11.7-27.5)
Kathua	22.7 (16.0-30.9)	18.2 (11.9-26.1)	14.6 (10.3-20.2)	12.6 (8.3-18.3)
Kishtwar	31.1 (22.0-42.2)	26.0 (17.2-36.8)	19.6 (13.6-27.0)	17.6 (11.4-25.6)
Kulgam	24.0 (17.5-32.0)	19.5 (13.2-27.4)	15.3 (10.9-20.5)	13.3 (8.8-19.0)
Kupwara	27.3 (18.9-37.3)	21.7 (14.1-31.4)	16.8 (11.3-23.3)	14.3 (8.8-20.9)
Leh	25.7 (16.5-38.2)	20.6 (12.1-32.7)	15.4 (9.8-23.5)	13.4 (7.8-21.7)
PoJK	33.7 (25.9-43.4)	27.3 (20.1-36.7)	22.1 (16.3-29.0)	18.2 (12.7-25.0)
Poonch	22.1 (15.9-30.1)	17.8 (12.0-25.5)	14.0 (10.1-19.1)	12.1 (8.1-17.4)
Pulwama	25.8 (18.3-34.7)	20.8 (13.8-29.4)	16.3 (11.4-22.0)	14.0 (9.1-20.0)
Rajouri	21.5 (15.4-29.2)	17.5 (11.8-25.2)	13.7 (9.6-19.0)	12.0 (7.9-17.6)
Ramban	24.7 (17.8-33.5)	20.6 (13.9-29.4)	15.9 (11.1-22.0)	14.2 (9.3-20.4)
Reasi	22.3 (16.0-30.2)	18.3 (12.2-26.5)	14.3 (10.1-19.6)	12.6 (8.3-18.3)
Samba	20.9 (14.2-29.1)	16.6 (10.6-24.6)	13.5 (9.3-19.0)	11.6 (7.4-17.0)
Shopian	24.3 (17.7-32.6)	19.6 (13.4-28.0)	15.5 (11.0-21.0)	13.3 (8.8-19.2)
Srinagar	23.7 (16.6-32.2)	18.7 (12.1-26.9)	14.9 (10.3-20.8)	12.6 (8.0-18.5)
Udhampur	22.6 (15.8-30.7)	18.5 (12.1-26.6)	14.6 (10.1-19.9)	12.8 (8.3-18.6)
**Haryana**	**28.8 (21.2-37.7)**	**22.8 (15.8-31.3)**	**15.8 (11.6-20.7)**	**13.5 (9.4-18.4)**
Ambala	23.0 (16.5-30.9)	17.9 (12.0-25.4)	12.7 (9.0-17.4)	10.6 (7.0-15.3)
Bhiwani	26.7 (19.1-36.3)	20.8 (13.8-30.0)	14.8 (10.5-20.2)	12.4 (8.2-17.9)
Charkhi Dadri	26.7 (18.7-36.9)	20.8 (13.5-30.5)	14.8 (10.3-20.4)	12.3 (8.0-18.1)
Faridabad	33.4 (24.2-44.7)	27.1 (18.5-38.3)	18.0 (13.0-24.2)	15.8 (10.6-22.3)
Fatehabad	28.9 (20.5-38.9)	22.9 (15.1-32.7)	16.1 (11.4-21.8)	13.7 (8.9-19.4)
Gurugram	30.4 (22.3-41.0)	24.5 (16.9-34.6)	16.6 (12.0-22.4)	14.4 (9.8-20.5)
Hisar	28.2 (20.1-37.5)	22.2 (14.9-31.1)	15.7 (11.2-21.0)	13.2 (8.9-18.8)
Jhajjar	28.7 (20.8-39.2)	22.6 (15.3-32.6)	15.8 (11.3-21.7)	13.3 (8.9-19.4)
Jind	29.2 (20.7-39.3)	22.8 (15.1-32.3)	16.1 (11.3-21.6)	13.5 (8.9-19.0)
Kaithal	27.0 (18.8-36.6)	20.8 (13.4-29.6)	14.9 (10.3-20.2)	12.3 (8.0-17.6)
Karnal	28.1 (20.0-37.4)	21.7 (14.5-30.4)	15.4 (10.8-20.8)	12.8 (8.5-18.2)
Kurukshetra	25.6 (18.5-34.2)	19.8 (13.5-28.0)	14.1 (10.1-19.1)	11.7 (7.8-16.8)
Mahendragarh	22.9 (16.5-31.1)	17.8 (12.0-25.5)	12.7 (9.0-17.2)	10.6 (7.0-15.2)
Nuh	45.9 (33.1-62.8)	38.6 (26.1-55.8)	24.9 (17.8-33.7)	22.5 (15.1-32.1)
Palwal	42.0 (30.5-57.1)	34.6 (23.6-49.5)	22.6 (16.2-30.6)	20.1 (13.5-28.6)
Panchkula	20.7 (14.7-28.4)	16.3 (10.8-23.6)	11.6 (8.2-15.9)	9.9 (6.6-14.2)
Panipat	27.0 (19.6-36.3)	20.9 (14.2-29.4)	14.8 (10.6-19.8)	12.2 (8.2-17.3)
Rewari	28.6 (20.9-39.2)	22.8 (15.5-32.9)	15.7 (11.3-21.5)	13.4 (9.1-19.4)
Rohtak	28.3 (20.4-38.3)	22.1 (14.8-31.4)	15.6 (11.1-21.2)	13.1 (8.7-18.7)
Sirsa	25.8 (18.4-35.4)	20.7 (13.7-29.7)	14.5 (10.3-20.0)	12.4 (8.2-18.0)
Sonipat	28.6 (20.8-38.2)	22.2 (15.1-31.1)	15.6 (11.4-20.9)	13.1 (8.8-18.4)
Yamunanagar	26.6 (19.2-35.8)	20.8 (14.1-29.7)	14.6 (10.5-20.0)	12.3 (8.2-17.8)
**Uttarakhand**	**22.2 (16.6-29.1)**	**17.4 (12.3-24.1)**	**13.3 (9.9-17.7)**	**11.1 (7.8-15.6)**
Almora	15.9 (11.6-21.3)	12.1 (8.3-16.9)	9.6 (6.9-12.9)	7.8 (5.3-11.1)
Bageshwar	16.5 (11.7-23.0)	12.5 (8.3-18.6)	9.8 (6.9-13.7)	7.9 (5.2-11.8)
Chamoli	15.9 (11.0-22.3)	12.3 (8.0-18.5)	9.4 (6.4-13.4)	7.8 (5.0-11.7)
Champawat	17.6 (12.6-24.2)	13.0 (8.7-18.8)	10.5 (7.5-14.4)	8.2 (5.5-12.0)
Dehradun	22.6 (16.3-30.0)	18.1 (12.3-25.5)	13.5 (9.7-18.2)	11.6 (7.7-16.6)
Haridwar	28.7 (21.0-38.0)	22.7 (15.7-31.7)	17.2 (12.5-23.0)	14.5 (10.0-20.6)
Nainital	18.9 (14.0-25.2)	14.6 (10.2-20.5)	11.4 (8.3-15.5)	9.4 (6.4-13.6)
Pauri Garhwal	21.0 (15.5-27.8)	16.6 (11.5-23.0)	12.6 (9.2-16.8)	10.6 (7.3-15.0)
Pithoragarh	16.4 (11.4-23.6)	12.2 (7.9-18.7)	9.6 (6.5-13.8)	7.6 (4.8-11.6)
Rudraprayag	18.1 (12.4-25.6)	14.4 (9.1-21.4)	10.8 (7.4-15.3)	9.1 (5.9-13.6)

Tehri Garhwal	20.8 (14.8-28.0)	16.6 (11.1-23.7)	12.4 (8.7-16.9)	10.6 (7.0-15.3)
Udham Singh Nagar	24.4 (18.1-33.0)	19.0 (13.2-26.9)	14.6 (10.7-19.6)	12.2 (8.3-17.2)
Uttarkashi	24.5 (17.2-33.7)	20.0 (13.1-29.4)	14.7 (10.2-20.3)	12.7 (8.2-18.7)
**Tamil Nadu**	**12.4 (8.3-18.0)**	**8.9 (5.4-14.0)**	**7.5 (4.8-11.1)**	**5.7 (3.4-9.2)**
Ariyalur	15.6 (9.6-24.1)	11.4 (6.3-18.9)	9.4 (5.7-14.6)	7.3 (4.0-12.2)
Chennai	9.1 (5.5-14.5)	6.6 (3.5-11.3)	5.6 (3.2-9.1)	4.3 (2.2-7.5)
Coimbatore	8.7 (5.4-13.5)	6.1 (3.5-10.4)	5.4 (3.3-8.3)	4.0 (2.2-6.7)
Cuddalore	13.8 (8.5-21.1)	9.9 (5.5-16.3)	8.3 (5.2-12.9)	6.3 (3.6-10.6)
Dharmapuri	14.0 (9.0-20.5)	10.0 (5.8-15.7)	8.7 (5.5-12.9)	6.5 (3.8-10.4)
Dindigul	12.9 (7.8-19.8)	9.2 (5.0-15.3)	7.8 (4.7-12.4)	5.9 (3.2-10.1)
Erode	13.0 (8.3-19.6)	9.2 (5.3-15.0)	8.0 (5.0-12.3)	6.0 (3.4-9.8)
Kanchipuram	11.3 (7.0-17.9)	8.1 (4.5-13.9)	6.9 (4.2-11.1)	5.3 (2.9-9.2)
Kanniyakumari	6.8 (3.6-11.6)	4.8 (2.2-8.9)	3.9 (2.0-6.9)	2.9 (1.3-5.5)
Karur	12.7 (8.0-19.3)	9.1 (5.2-15.0)	7.7 (4.7-12.1)	5.8 (3.2-9.9)
Krishnagiri	15.0 (9.6-21.8)	10.7 (6.2-16.6)	9.3 (5.8-13.8)	7.0 (4.0-11.2)
Madurai	14.3 (8.2-22.5)	10.3 (5.3-17.6)	8.6 (4.9-13.4)	6.5 (3.3-11.0)
Nagapattinam	13.1 (7.8-20.5)	9.5 (4.9-16.1)	7.8 (4.6-12.5)	6.0 (3.1-10.6)
Namakkal	11.9 (7.5-18.4)	8.5 (4.8-14.0)	7.3 (4.5-11.2)	5.5 (3.0-9.1)
Perambalur	13.8 (8.5-21.0)	10.0 (5.6-16.3)	8.4 (5.1-12.9)	6.4 (3.5-10.6)
Pudukkottai	14.2 (8.7-21.6)	10.2 (5.7-16.9)	8.5 (5.1-12.9)	6.4 (3.5-10.7)
Ramanathapuram	11.5 (6.6-18.3)	8.3 (4.3-14.3)	6.8 (3.8-10.9)	5.2 (2.5-9.0)
Salem	12.9 (8.2-19.3)	9.2 (5.3-14.8)	7.9 (4.9-11.8)	6.0 (3.4-9.7)
Sivaganga	12.9 (7.6-19.9)	9.2 (4.9-15.4)	7.7 (4.5-11.9)	5.8 (3.0-9.6)
Thanjavur	14.3 (8.7-22.4)	10.4 (5.6-17.7)	8.6 (5.1-13.6)	6.6 (3.5-11.2)
The Nilgiris	9.6 (6.0-14.4)	6.7 (3.8-11.0)	6.0 (3.7-9.1)	4.4 (2.4-7.2)
Theni	13.6 (7.8-21.4)	9.8 (4.9-16.8)	8.2 (4.6-13.3)	6.2 (3.1-11.0)
Thiruvallur	11.0 (6.7-17.4)	7.9 (4.3-13.4)	6.7 (4.0-10.8)	5.2 (2.8-8.9)
Thiruvarur	14.1 (8.4-22.2)	10.2 (5.5-17.7)	8.4 (5.0-13.5)	6.5 (3.4-11.4)
Thoothukudi	11.9 (6.7-19.2)	8.7 (4.3-15.4)	7.0 (3.8-11.7)	5.4 (2.6-9.8)
Tiruchirappalli	13.5 (8.3-20.4)	9.7 (5.4-15.8)	8.2 (5.0-12.5)	6.2 (3.4-10.4)
Tirunelveli	10.5 (5.8-17.0)	7.6 (3.7-13.4)	6.2 (3.3-10.1)	4.7 (2.2-8.4)
Tiruppur	10.9 (6.7-17.0)	7.6 (4.3-13.1)	6.6 (4.0-10.5)	4.9 (2.7-8.5)
Tiruvannamalai	13.7 (8.8-20.7)	9.9 (5.8-16.1)	8.4 (5.3-13.0)	6.5 (3.7-10.7)
Vellore	13.5 (8.7-20.3)	9.8 (5.6-15.7)	8.3 (5.2-12.8)	6.4 (3.6-10.7)
Viluppuram	14.0 (9.0-21.0)	10.2 (5.9-16.5)	8.5 (5.4-13.0)	6.6 (3.7-10.9)
Virudhunagar	14.4 (8.3-22.9)	10.5 (5.3-18.1)	8.6 (4.7-13.8)	6.6 (3.2-11.4)
**Mizoram**	**32.9 (22.9-45.5)**	**29.5 (19.1-43.5)**	**16.8 (11.7-23.7)**	**15.7 (10.2-23.6)**
Aizawl	30.0 (21.0-42.8)	26.7 (17.3-40.8)	15.8 (11.0-22.3)	14.6 (9.5-22.2)
Champhai	30.3 (20.9-43.0)	27.9 (17.7-42.4)	15.1 (10.5-21.7)	14.5 (9.3-22.1)
Kolasib	30.5 (21.5-43.0)	26.7 (17.4-40.1)	16.5 (11.4-23.5)	15.0 (9.7-22.7)
Lawngtlai	40.1 (27.5-56.9)	36.8 (23.2-56.1)	19.4 (12.8-28.3)	18.5 (11.1-28.9)
Lunglei	33.7 (23.0-47.9)	30.2 (19.0-45.6)	16.6 (11.3-23.7)	15.5 (9.6-23.8)
Mamit	35.0 (24.3-49.6)	30.6 (19.4-46.4)	18.8 (12.9-27.0)	17.1 (10.9-26.1)
Saiha	46.3 (31.2-66.1)	43.1 (26.8-66.4)	21.7 (14.3-31.7)	21.0 (12.6-33.3)
Serchhip	30.6 (21.2-43.0)	27.4 (17.4-41.2)	15.4 (10.6-22.1)	14.3 (9.1-21.9)
**Maharashtra**	**18.3 (13.2-24.4)**	**14.1 (9.5-19.9)**	**12.4 (8.9-16.5)**	**10.0 (6.7-14.0)**
Ahmednagar	17.3 (11.2-25.0)	13.2 (8.0-20.6)	11.5 (7.6-16.7)	9.3 (5.6-14.3)
Akola	19.9 (13.2-27.8)	15.4 (9.3-22.9)	14.0 (9.1-19.9)	11.4 (6.8-17.3)
Amravati	19.8 (13.0-28.3)	14.9 (8.9-22.6)	13.9 (9.1-20.1)	11.1 (6.6-17.1)
Aurangabad	19.1 (12.6-27.2)	14.8 (9.0-22.4)	12.9 (8.5-18.5)	10.5 (6.4-16.0)
Beed	18.5 (12.1-26.1)	14.3 (8.4-21.5)	12.5 (8.1-17.7)	10.2 (6.0-15.3)
Bhandara	22.6 (15.5-32.5)	16.8 (10.7-25.9)	16.0 (10.9-23.2)	12.6 (8.0-19.7)
Buldhana	21.3 (14.0-30.2)	16.6 (10.1-25.0)	14.8 (9.6-21.4)	12.1 (7.2-18.7)
Chandrapur	24.0 (16.1-35.0)	18.6 (11.5-28.9)	17.1 (11.3-25.1)	14.0 (8.4-22.3)
Dhule	21.6 (14.5-31.5)	16.5 (10.4-25.7)	14.4 (9.6-21.2)	11.6 (7.1-18.2)
Gadchiroli	28.9 (20.5-41.4)	22.8 (15.0-35.0)	20.5 (14.1-29.6)	17.0 (10.9-26.2)
Gondia	23.1 (16.1-33.2)	17.2 (11.0-26.1)	16.5 (11.3-23.7)	12.9 (8.3-20.0)
Hingoli	22.3 (14.5-32.3)	17.6 (10.4-27.4)	15.5 (9.9-22.9)	12.9 (7.6-20.4)

Jalgaon	21.4 (14.4-30.5)	16.3 (10.1-24.7)	14.6 (9.7-21.2)	11.7 (7.2-17.9)
Jalna	22.4 (14.6-32.2)	17.7 (10.6-27.3)	15.3 (10.0-22.3)	12.7 (7.6-19.8)
Kolhapur	12.3 (7.7-18.5)	8.8 (5.0-14.4)	7.9 (4.9-12.2)	5.9 (3.3-9.7)
Latur	16.2 (10.4-23.8)	12.2 (7.1-19.2)	11.0 (6.9-16.2)	8.7 (4.9-13.7)
Mumbai City	16.3 (9.8-25.0)	13.0 (7.1-21.6)	10.7 (6.3-17.1)	8.9 (4.7-15.5)
Mumbai Suburban	16.3 (10.0-24.5)	13.1 (7.3-21.3)	10.7 (6.5-16.8)	9.0 (4.9-15.1)
Nagpur	20.4 (13.8-29.1)	15.3 (9.6-23.6)	14.5 (9.6-21.0)	11.5 (7.1-17.8)
Nanded	21.4 (14.1-30.8)	16.7 (10.1-25.9)	15.0 (9.6-21.7)	12.3 (7.3-19.2)
Nandurbar	26.9 (18.2-37.5)	20.6 (12.9-30.7)	17.8 (11.9-25.4)	14.3 (8.8-21.9)
Nashik	18.8 (12.5-26.9)	14.4 (9.0-22.0)	12.5 (8.4-17.9)	10.1 (6.3-15.4)
Osmanabad	16.5 (10.5-23.7)	12.4 (7.2-19.2)	11.0 (6.9-15.8)	8.7 (4.9-13.4)
Palghar	20.0 (13.1-29.3)	15.9 (9.5-25.0)	13.2 (8.6-19.5)	11.0 (6.5-17.5)
Parbhani	22.7 (14.7-32.5)	17.9 (10.6-27.3)	15.7 (10.1-22.7)	13.0 (7.6-20.0)
Pune	14.4 (9.4-21.8)	10.9 (6.5-17.8)	9.5 (6.1-14.5)	7.6 (4.4-12.2)
Raigad	16.1 (10.0-23.9)	12.6 (7.1-20.1)	10.6 (6.5-16.1)	8.6 (4.8-14.2)
Ratnagiri	13.9 (8.3-22.0)	10.5 (5.7-17.8)	9.1 (5.3-14.4)	7.1 (3.7-12.2)
Sangli	13.0 (8.3-19.4)	9.3 (5.4-14.8)	8.5 (5.3-12.7)	6.3 (3.6-10.1)
Satara	13.2 (8.5-19.9)	9.9 (5.8-16.0)	8.7 (5.5-13.0)	6.7 (3.8-10.9)
Sindhudurg	13.2 (7.9-20.9)	9.7 (5.3-16.5)	8.5 (5.0-13.5)	6.5 (3.4-11.2)
Solapur	15.2 (9.5-22.4)	11.2 (6.4-17.5)	10.1 (6.2-14.9)	7.8 (4.4-12.3)
Thane	17.5 (11.2-26.0)	13.9 (8.2-22.5)	11.5 (7.1-17.4)	9.6 (5.4-15.7)
Wardha	21.4 (14.2-30.5)	16.4 (10.0-24.9)	15.2 (9.9-21.8)	12.3 (7.3-18.8)
Washim	20.6 (13.8-29.6)	16.2 (9.8-24.8)	14.4 (9.4-20.7)	12.0 (7.1-18.5)
Yavatmal	22.2 (14.7-31.7)	17.4 (10.7-26.8)	15.7 (10.3-22.6)	13.0 (7.9-20.1)
**Punjab**	**21.7 (16.0-28.8)**	**17.2 (11.8-23.9)**	**12.6 (9.2-16.7)**	**10.5 (7.2-14.7)**
Amritsar	21.6 (15.8-29.0)	17.3 (11.8-24.2)	12.6 (9.1-16.8)	10.6 (7.1-15.0)
Barnala	24.8 (17.9-34.0)	19.3 (13.0-28.1)	14.3 (10.2-19.5)	11.8 (7.8-17.1)
Bathinda	22.3 (16.1-30.2)	17.6 (11.9-25.0)	13.0 (9.2-17.4)	10.8 (7.2-15.3)
Faridkot	25.2 (18.1-34.3)	20.0 (13.5-28.6)	14.6 (10.4-20.0)	12.3 (8.1-17.5)
Fatehgarh Sahib	18.6 (13.4-24.7)	14.3 (9.6-19.9)	10.7 (7.6-14.4)	8.7 (5.8-12.4)
Fazilka	23.1 (16.7-31.4)	18.5 (12.4-26.7)	13.5 (9.6-18.5)	11.4 (7.6-16.6)
Ferozepur	28.7 (20.7-39.2)	23.0 (15.5-32.8)	16.7 (11.9-22.6)	14.1 (9.3-20.1)
Gurdaspur	20.3 (14.7-27.2)	16.3 (11.1-23.2)	11.8 (8.5-16.0)	10.0 (6.8-14.3)
Hoshiarpur	19.1 (13.6-25.6)	15.2 (10.2-21.3)	11.1 (7.9-14.9)	9.3 (6.2-13.1)
Jalandhar	21.2 (15.1-28.6)	16.8 (11.1-24.1)	12.3 (8.8-16.6)	10.3 (6.9-14.7)
Kapurthala	22.5 (16.2-30.3)	17.9 (12.1-25.6)	13.0 (9.3-17.6)	11.0 (7.3-15.6)
Ludhiana	20.7 (15.0-27.9)	16.1 (11.0-23.0)	11.9 (8.6-16.3)	9.8 (6.6-14.2)
Mansa	24.1 (17.1-32.4)	18.9 (12.5-26.8)	13.9 (9.8-18.8)	11.6 (7.6-16.5)
Moga	26.4 (18.7-36.7)	21.0 (13.8-30.8)	15.3 (10.6-21.1)	12.8 (8.2-18.8)
Pathankot	18.1 (13.0-24.4)	14.5 (9.7-20.7)	10.6 (7.5-14.6)	8.9 (5.9-12.8)
Patiala	18.7 (13.5-24.8)	14.3 (9.7-19.8)	10.7 (7.8-14.2)	8.7 (5.9-12.1)
Rupnagar	18.5 (13.3-24.8)	14.5 (9.8-20.6)	10.7 (7.6-14.4)	8.9 (6.0-12.5)
Sahibzada Ajit Singh Nagar	17.1 (12.1-23.3)	13.3 (8.8-19.1)	10.0 (7.0-13.7)	8.3 (5.4-12.0)
Sangrur	23.3 (16.8-31.2)	18.0 (12.1-25.5)	13.4 (9.6-18.1)	11.0 (7.4-15.6)
Shahid Bhagat Singh Nagar	20.1 (14.5-27.2)	15.8 (10.8-22.6)	11.6 (8.4-15.7)	9.7 (6.5-13.8)
Sri Mukhtar Sahib	22.3 (16.2-30.2)	17.7 (12.1-25.3)	13.0 (9.3-17.8)	10.9 (7.2-15.7)
Tarn Taran	26.2 (19.0-35.3)	21.1 (14.3-30.0)	15.2 (10.9-20.7)	12.9 (8.6-18.5)
**Sikkim**	**14.9 (10.5-20.6)**	**11.2 (7.4-16.5)**	**8.6 (6.0-12.1)**	**6.9 (4.5-10.3)**
East Sikkim	13.2 (9.3-18.3)	9.9 (6.6-14.6)	7.6 (5.3-10.8)	6.0 (3.9-9.1)
North Sikkim	18.1 (12.6-25.4)	13.8 (9.0-20.4)	10.4 (7.1-14.8)	8.3 (5.3-12.5)
South Sikkim	14.0 (9.8-19.4)	10.5 (6.9-15.4)	8.1 (5.6-11.5)	6.4 (4.1-9.7)
West Sikkim	17.1 (12.0-24.0)	13.0 (8.6-19.5)	10.0 (7.0-14.3)	8.0 (5.2-12.3)
**Nagaland**	**26.9 (19.2-36.4)**	**21.8 (14.6-31.4)**	**12.5 (8.9-17.1)**	**10.6 (7.0-15.3)**
Dimapur	27.3 (19.1-37.5)	21.6 (14.0-31.7)	13.2 (9.2-18.4)	10.8 (7.1-16.1)
Kiphire	24.7 (16.7-34.2)	20.9 (13.1-30.8)	10.8 (7.2-15.0)	9.5 (5.8-14.0)
Kohima	22.2 (15.7-30.2)	17.7 (11.6-25.6)	10.3 (7.3-14.2)	8.6 (5.6-12.5)
Longleng	29.7 (20.8-40.4)	24.3 (15.7-35.2)	13.8 (9.5-19.0)	11.7 (7.4-17.2)
Mokokchung	26.5 (18.4-36.5)	21.0 (13.5-30.6)	12.5 (8.7-17.3)	10.2 (6.5-15.2)

Mon	32.7 (22.6-45.5)	27.1 (17.4-39.8)	15.1 (10.4-21.3)	13.0 (8.3-19.4)
Peren	29.0 (20.6-39.3)	23.3 (15.4-33.5)	14.0 (9.9-19.1)	11.7 (7.8-16.9)
Phek	22.2 (15.3-30.2)	18.4 (11.7-26.4)	9.9 (6.7-13.6)	8.6 (5.4-12.5)
Tuensang	26.9 (18.5-36.9)	22.7 (14.4-33.0)	11.9 (8.1-16.5)	10.4 (6.4-15.5)
Wokha	28.0 (19.7-38.6)	22.4 (14.6-32.8)	13.4 (9.4-18.7)	11.1 (7.2-16.5)
Zunheboto	23.6 (16.1-32.3)	19.2 (12.2-28.1)	10.7 (7.3-14.6)	9.0 (5.7-13.3)
**Himachal Pradesh**	**24.3 (17.3-32.6)**	**19.9 (13.2-28.3)**	**14.9 (10.6-20.0)**	**12.7 (8.5-18.0)**
Bilaspur	19.7 (14.1-26.8)	15.8 (10.5-22.6)	12.1 (8.5-16.4)	10.2 (6.7-14.6)
Chamba	28.4 (19.6-39.6)	24.0 (15.4-35.2)	17.4 (11.9-24.3)	15.4 (9.6-22.6)
Hamirpur	18.8 (13.1-26.0)	15.2 (9.8-22.1)	11.6 (8.0-16.1)	9.8 (6.2-14.3)
Kangra	24.3 (16.9-33.0)	20.0 (13.0-28.8)	14.9 (10.3-20.8)	12.8 (8.2-18.8)
Kinnaur	29.7 (20.2-42.2)	24.4 (15.3-37.2)	18.0 (11.8-25.5)	15.5 (9.5-23.6)
Kullu	29.9 (20.9-41.2)	24.8 (15.9-36.3)	18.3 (12.4-25.4)	15.8 (9.9-23.3)
Lahul and Spiti	29.4 (19.6-41.2)	24.5 (15.0-36.1)	17.8 (11.8-25.3)	15.6 (9.4-23.5)
Mandi	22.4 (15.9-30.5)	18.1 (11.8-26.1)	13.7 (9.7-18.7)	11.6 (7.6-16.7)
Shimla	28.8 (20.2-39.7)	23.6 (15.4-34.7)	17.5 (12.2-24.4)	15.1 (9.7-22.4)
Sirmaur	26.2 (19.0-35.3)	21.3 (14.5-30.3)	15.9 (11.4-21.4)	13.6 (9.1-19.3)
Solan	20.0 (14.4-27.5)	16.0 (10.8-22.9)	12.2 (8.7-16.7)	10.3 (6.9-14.8)
Una	19.9 (14.0-27.2)	16.0 (10.6-23.1)	12.3 (8.6-16.9)	10.4 (6.7-15.1)
**Union territories other than Delhi**	**18.2 (14.4-23.2)**	**15.2 (11.4-20.4)**	**11.5 (9.0-14.8)**	**10.0 (7.5-13.4)**
Chandigarh	18.6 (13.1-25.7)	15.6 (10.2-22.8)	12.4 (8.7-17.3)	11.0 (7.2-16.2)
Dadra & Nagar Haveli	25.5 (16.2-38.3)	21.9 (12.4-35.2)	16.6 (10.5-25.1)	14.8 (8.4-24.3)
Daman	19.2 (12.2-28.3)	15.8 (9.2-24.8)	12.4 (7.9-18.3)	10.7 (6.3-16.9)
Diu	21.8 (13.6-33.9)	18.2 (10.3-30.6)	14.0 (8.6-22.2)	12.1 (6.8-20.8)
Karaikal	17.7 (10.2-28.2)	14.5 (7.5-25.6)	10.4 (6.0-16.5)	8.8 (4.6-15.6)
Lakshadweep‡				
Mahe	7.7 (4.5-12.5)	6.2 (3.2-10.9)	5.1 (2.8-8.4)	4.2 (2.1-7.4)
Nicobars	10.7 (3.2-25.7)	9.3 (2.4-23.9)	5.9 (1.6-14.7)	5.2 (1.2-14.4)
North & Middle Andaman	21.2 (9.7-39.2)	18.0 (7.0-37.9)	12.0 (5.3-23.1)	10.4 (3.8-22.1)
Puducherry	15.9 (10.0-24.7)	13.0 (7.3-22.0)	9.5 (5.8-14.6)	8.0 (4.4-13.5)
South Andaman	15.0 (6.9-27.5)	12.7 (5.0-25.9)	8.5 (3.8-16.4)	7.3 (2.7-15.4)
Yanam	14.4 (8.9-22.2)	10.6 (6.1-17.6)	8.4 (5.1-12.8)	6.6 (3.7-11.0)
**Kerala**	**7.6 (4.6-11.7)**	**6.0 (3.3-9.9)**	**5.2 (3.1-8.2)**	**4.2 (2.3-7.1)**
Alappuzha	6.6 (3.7-11.1)	5.2 (2.6-9.5)	4.5 (2.4-7.6)	3.6 (1.7-6.6)
Ernakulam	6.0 (3.5-9.7)	4.7 (2.4-8.3)	4.1 (2.3-6.8)	3.3 (1.6-5.8)
Idukki	10.8 (6.3-17.1)	8.6 (4.5-14.7)	7.3 (4.2-11.7)	6.0 (3.1-10.5)
Kannur	8.3 (4.9-12.8)	6.5 (3.4-10.8)	5.8 (3.3-9.1)	4.7 (2.4-8.1)
Kasaragod	7.7 (4.4-12.3)	6.0 (3.1-10.4)	5.3 (3.0-8.7)	4.3 (2.1-7.5)
Kollam	7.6 (4.3-12.7)	6.1 (3.0-11.1)	5.1 (2.7-8.4)	4.1 (2.0-7.5)
Kottayam	7.0 (4.0-11.5)	5.5 (2.9-10.0)	4.8 (2.7-7.9)	3.8 (1.9-6.9)
Kozhikode	8.0 (4.7-12.7)	6.4 (3.3-10.9)	5.6 (3.1-9.0)	4.5 (2.3-7.9)
Malappuram	8.2 (4.9-12.8)	6.5 (3.5-10.9)	5.7 (3.3-9.2)	4.6 (2.4-8.1)
Palakkad	8.0 (4.7-12.9)	6.3 (3.3-11.0)	5.5 (3.1-9.0)	4.4 (2.3-7.9)
Pathanamthitta	8.5 (4.8-14.0)	6.8 (3.4-12.4)	5.7 (3.2-9.5)	4.7 (2.3-8.5)
Thiruvananthapuram	7.5 (4.0-12.7)	6.0 (2.8-10.9)	4.9 (2.5-8.4)	4.0 (1.8-7.4)
Thrissur	6.1 (3.4-10.0)	4.8 (2.4-8.6)	4.2 (2.3-6.8)	3.3 (1.7-6.1)
Wayanad	10.1 (5.9-15.8)	7.9 (4.1-13.4)	7.0 (4.0-11.2)	5.7 (2.9-9.8)
**Delhi**	**16.9 (12.4-22.8)**	**12.6 (8.7-17.9)**	**11.4 (8.3-15.3)**	**9.0 (6.1-12.8)**
Central	17.3 (12.6-23.5)	12.9 (8.9-18.5)	11.7 (8.5-15.8)	9.2 (6.3-13.2)
East	16.8 (12.3-22.6)	12.5 (8.6-17.7)	11.3 (8.2-15.3)	8.9 (6.1-12.6)
New Delhi	17.7 (12.9-24.0)	13.3 (9.2-19.0)	12.0 (8.7-16.2)	9.5 (6.5-13.6)
North	16.7 (12.2-22.6)	12.4 (8.6-17.7)	11.3 (8.2-15.2)	8.9 (6.0-12.7)
North East	16.7 (12.2-22.4)	12.3 (8.5-17.6)	11.3 (8.2-15.1)	8.9 (6.0-12.6)
North West	16.1 (11.8-21.6)	11.8 (8.1-16.9)	10.9 (7.9-14.6)	8.5 (5.8-12.0)
Shahdara	16.9 (12.4-22.7)	12.5 (8.7-17.8)	11.3 (8.2-15.2)	8.9 (6.1-12.6)
South	17.8 (13.1-23.9)	13.4 (9.3-19.0)	11.9 (8.6-16.0)	9.5 (6.4-13.5)
South East	17.5 (12.9-23.5)	13.1 (9.1-18.6)	11.7 (8.5-15.8)	9.3 (6.3-13.2)
South West	17.3 (12.7-23.2)	13.0 (9.0-18.3)	11.6 (8.4-15.6)	9.2 (6.3-13.0)

West	16.6 (12.1-22.5)	12.4 (8.5-17.7)	11.2 (8.2-15.0)	8.9 (6.0-12.5)
**Goa**	**13.8 (8.1-21.2)**	**10.9 (5.8-18.3)**	**8.0 (4.6-12.8)**	**6.5 (3.4-11.2)**
North Goa	14.3 (8.4-22.4)	11.4 (6.0-19.4)	8.4 (4.8-13.4)	6.8 (3.5-11.7)
South Goa	13.3 (7.9-20.6)	10.5 (5.6-17.4)	7.8 (4.5-12.3)	6.3 (3.3-10.7)

*The states are listed in increasing order of Socio-demographic Index in 2017.
†The state of Jammu and Kashmir was divided into two union territories in August 2019; as we are reporting findings up to 2017, we report findings for the undivided state of Jammu and Kashmir.
‡Projections for Lakshadweep are not presented because of data limitations.

13. **Identification of priority districts in the states based on tertile groupings of the district-level rates of child mortality in 2017 against the annual rate of their reduction from 2010 to 2017**

Priority districts were identified in 18 states with 20 or more districts based on the child mortality rates against the annual rate of reduction. We use examples of three states in the low SDI group to describe how the differences in child mortality rates and rate of change over time can help identify districts that need the highest attention. For this description, we chose Uttar Pradesh as it had the highest U5MR and NMR in 2017, Assam as it had the second highest U5MR and NMR in 2017, and Bihar as it had relatively high rates but the lowest inequality between districts among the low SDI states. The maps showing the identification of priority districts in Uttar Pradesh, Assam and Bihar are on pp 136-138.

Based on the tertiles of district-level NMR in 2017 in Uttar Pradesh and the tertiles of the annual rate of reduction from 2010 to 2017, a cluster of eight districts in the north-central part (Bahraich, Balrampur, Barabanki, Gonda, Hardoi, Kheri, Shravasti, and Sitapur), a cluster of two districts in the south (Allahabad and Chitrakoot), and Lalitpur district in the south-west corner of the state had high NMR in 2017 and low annual rate of reduction from 2010 to 2017. These districts therefore would need the highest priority for NMR in Uttar Pradesh. In addition, districts with either high NMR and medium reduction rate or medium NMR and low reduction rate would also need priority attention for them not to spill over to the worst group with high NMR and low reduction rate. The former of these two categories include a cluster of nine districts in the west-central part of the state and one district in the south-east; and the later of these two categories include a cluster of seven districts in the east-centre, two in the south-west and one in the south-east part of the state. Interestingly, the majority of districts in these two categories were contiguous with the worst category of high NMR and low reduction rate. The districts with low NMR and high annual rate of reduction included a cluster of nine districts in the east, one in the centre, and three in the west periphery. The relative standing of the districts in Uttar Pradesh for U5MR in 2017 and the annual rates of reduction from 2010 to 2017 was generally similar to the trends for NMR. One exception was Banda in the south which was in the category of medium NMR and low rate of reduction, but fell in the worst category of high U5MR and low rate of reduction.

While use of the above approach based on tertiles of mortality distribution in the districts within a state allows the assessment of relative prioritization of districts in that state, it is also important to examine the trends of districts in the states with respect to the tertiles of the nationwide district-level distribution of U5MR and NMR and the rate of their reduction as this enables a complimentary understanding of the standing of district in a prticular state with respect to all districts in the country. This approach revealed that the majority of districts in Uttar Pradesh fell in the high nationwide tertile of NMR in 2017 (68 of 75, 90.7%) and only 3 (4.0%) districts fell in the high nationwide tertile for the rate of reduction of NMR from 2010 to 2017 as shown in the table below. This resulted in 36 (48%) districts in Uttar Pradesh falling in the worst category of high NMR and low annual rate of reduction within the nationwide distribution.

Table: Distribution of districts in low SDI states by tertiles of nationwide distribution of child mortality

States	Tertile	Percentage of districts			
		U5MR in 2017	Annual rate of reduction of U5MR, 2010-2017	NMR in 2017	Annual rate of reduction of NMR, 2010-2017
Uttar Pradesh	Low	0.0	26.7	0.0	48.0
	Medium	6.7	60.0	9.3	48.0
	High	93.3	13.3	90.7	4.0
Assam	Low	0.0	54.6	0.0	48.5
	Medium	33.3	33.3	33.3	39.4
	High	66.7	12.1	66.7	12.1
Bihar	Low	0.0	13.2	0.0	60.5
	Medium	71.0	86.8	81.6	39.5
	High	29.0	0.0	18.4	0.0
Chhattisgarh	Low	0.0	29.6	0.0	14.8
	Medium	18.5	25.9	0.0	40.7
	High	81.5	44.4	100	44.4
Jharkhand	Low	0.0	45.8	4.1	45.8

	Medium	50.0	29.2	58.3	29.3
	High	50.0	25.0	37.5	25.0
Madhya Pradesh	Low	0.0	3.8	1.9	5.7
	Medium	34.6	30.8	34.6	36.5
	High	65.4	65.4	63.5	57.7
Odisha	Low	10.0	23.3	13.3	20.0
	Medium	23.3	43.3	26.7	43.3
	High	66.7	33.3	60.0	36.7
Rajasthan	Low	12.1	51.5	12.1	51.5
	Medium	15.2	18.2	15.2	18.1
	High	72.7	30.3	72.7	30.3

The tertile cut-offs for India for U5MR were 30.9 and 45.2 and for its annual rate of reduction were 4.15% and 5.12%.
The tertile cut-offs for India for NMR were 17.6 and 24.6 and for its annual rate of reduction were 3.04% and 4.11%.

Based on tertiles of district-level U5MR and NMR levels in 2017 within a state and their annual rate of reduction from 2000 to 2017, in the state of Assam the worst category of high mortality rates and low reduction rate was concentrated in the southern handle of the state (Karbi Anglong, West Karbi Anglong, Dima Hasao, Cachar, Karimganj, and Hallakandi), and the best category of low mortality rates and high reduction rate was concentrated in the western part of the state (Kokrajhar, Chirang, Baksa, Nalbari, Barpeta, Kamrup, and Kamrup Metropolitan) for both U5MR and NMR. In contrast, in the state of Bihar, the worst category of districts was scattered in the north-east (Kishanganj and Purnia for both U5MR and NMR, and in addition Madhepura and Araia for U5MR) and the south-west of the state (Kaimur and Aurangabad for both U5MR and NMR and in addition Rohtas for NMR), and the best category of districts was concentrated in the west-centre of the state for both U5MR and NMR (Siwan, Muzaffarpur, Saran, Vaishali, Samastipur and Patna for both and in addition Nalanda for U5MR).

As for Uttar Pradesh, in the nationwide distribution for NMR in 2017 none of the districts in Assam or Bihar were in the low tertile, and only 12.1% of the districts in Assam and none in Bihar were in the high tertile for the rate of reduction as shown in the table above. Interestingly, in Bihar the vast majority of the districts (81.6%) were in the medium nationwide tertile of NMR and only 18.4% in the high nationwide tertile of NMR. Several of the other low SDI states had a high proportion of districts in the high nationwide tertile of NMR (Chattisgarh 100%, Rajasthan 72.7%, Madhya Pradesh 63.5%, and Odisha 60%). In contrast to the other low SDI states, the proportion of districts in the high nationwide tertile for the annual rate of reduction was relatively higher for Madhya Pradesh (57.5%) and Chhattisgarh (44.4%).

Using the approach based on tertiles of district-level mortality and rate of reduction distribution within a state, the identification of priority districts in 15 other states with 20 or more districts is shown on pp 139-153.

Uttar Pradesh

A U5MR

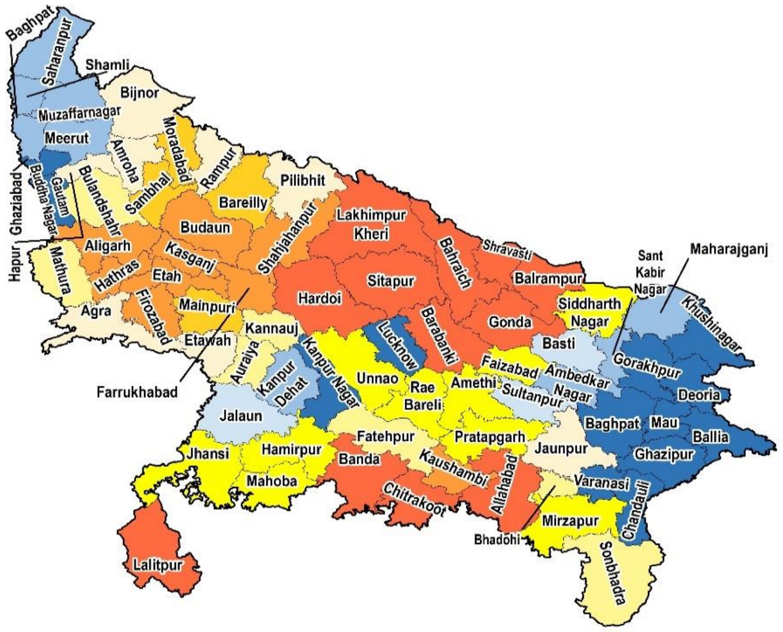

High, medium, and low groups based on tertiles of rate in 2017 and the annual rate of reduction (ARoR) from 2010 to 2017 among the districts within the state

- High rate and low ARoR
- High rate and medium ARoR
- High rate and high ARoR
- Medium rate and low ARoR
- Medium rate and medium ARoR
- Medium rate and high ARoR
- Low rate and low ARoR
- Low rate and medium ARoR
- Low rate and high ARoR

B NMR

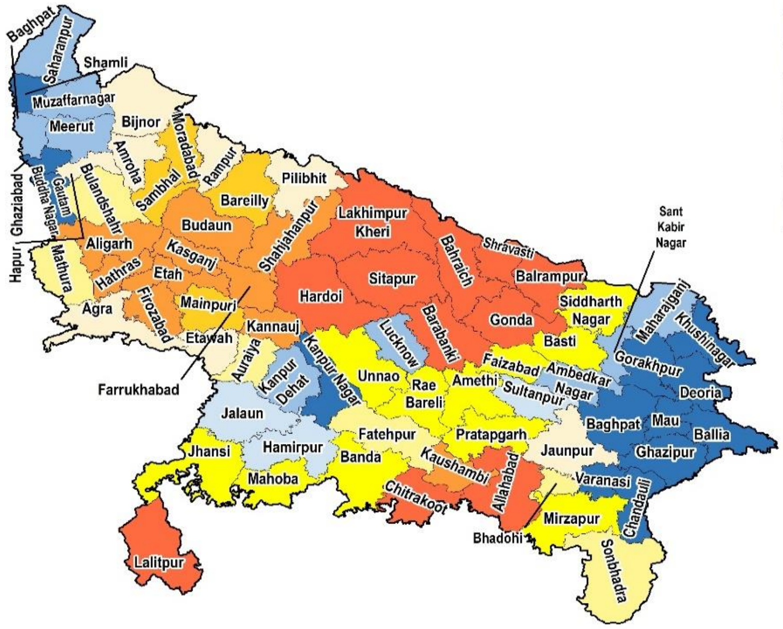

Assam

A U5MR

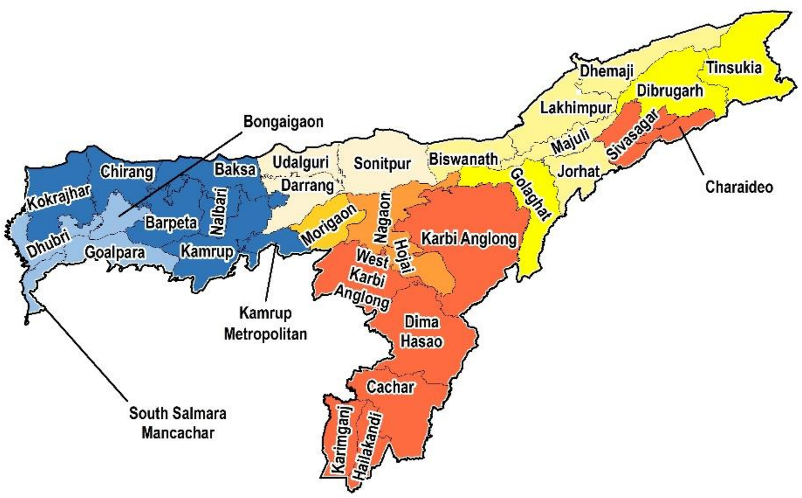

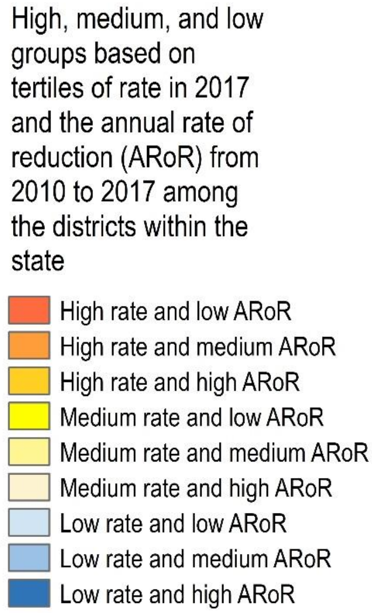

B NMR

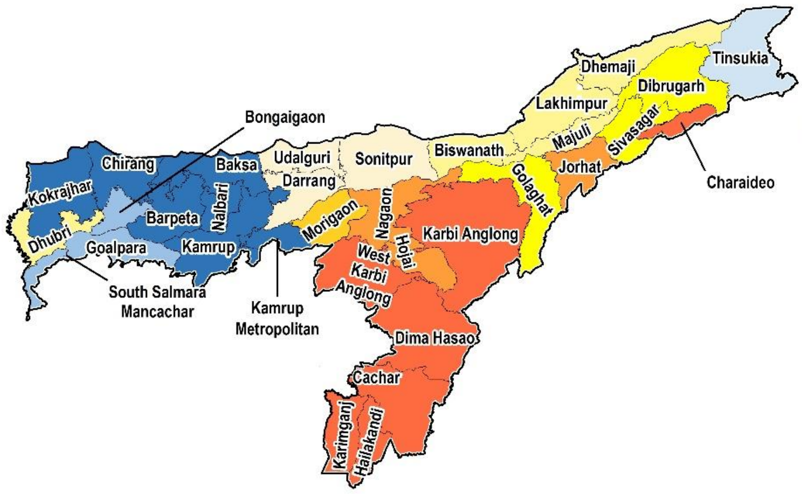

The tertile cut-offs for U5MR were 47.3 and 62.0 and its annual rate of reduction were 3.33% and 4.30%.
The tertile cut-offs for NMR were 25.1 and 32.7 and its annual rate of reduction were 2.56% and 3.37%.

Bihar

A U5MR

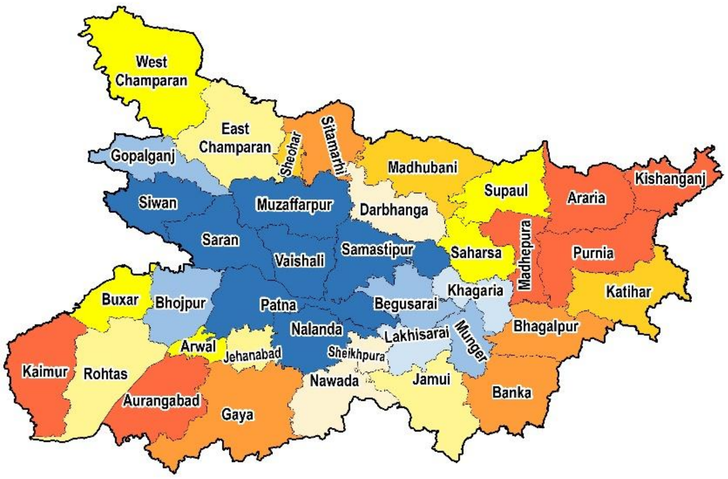

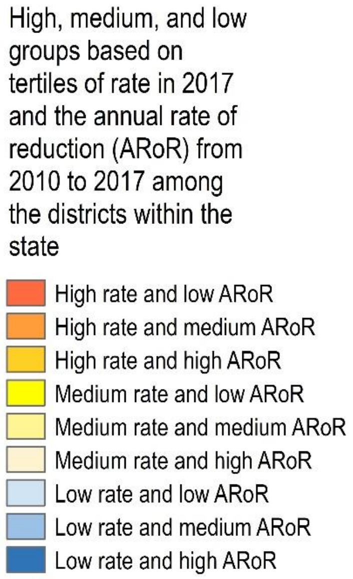

B NMR

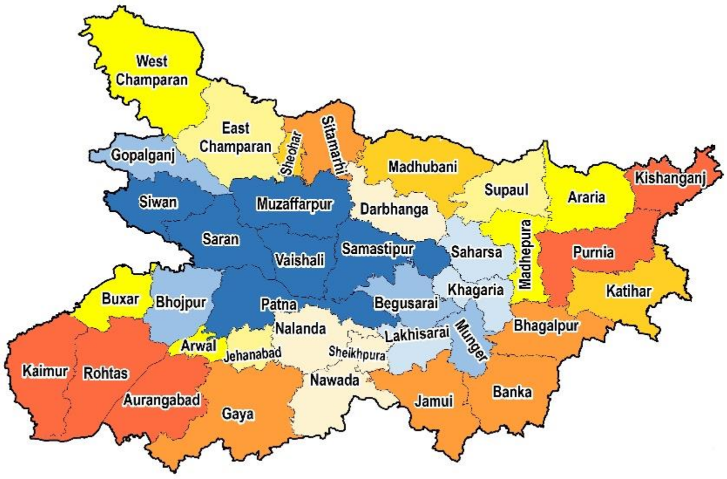

The tertile cut-offs for U5MR were 42.9 and 44.9 and its annual rate of reduction were 4.34% and 4.60%.
The tertile cut-offs for NMR were 22.9 and 24.1 and its annual rate of reduction were 2.89% and 3.13%.

Arunachal Pradesh

A U5MR

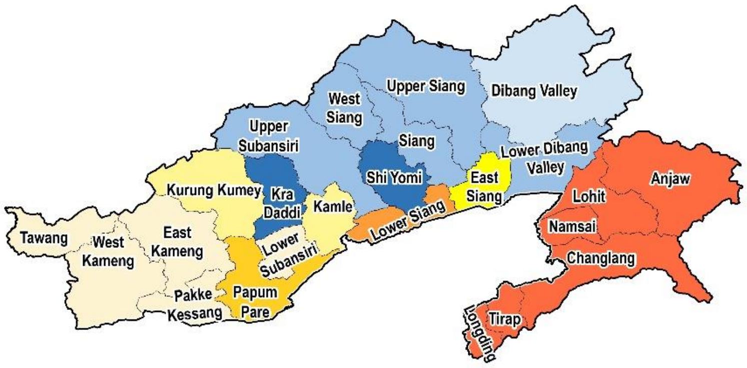

High, medium, and low groups based on tertiles of rate in 2017 and the annual rate of reduction (ARoR) from 2010 to 2017 among the districts within the state

- High rate and low ARoR
- High rate and medium ARoR
- High rate and high ARoR
- Medium rate and low ARoR
- Medium rate and medium ARoR
- Medium rate and high ARoR
- Low rate and low ARoR
- Low rate and medium ARoR
- Low rate and high ARoR

B NMR

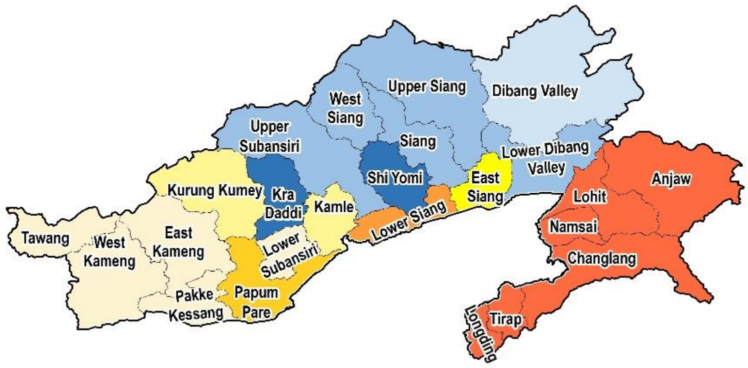

The tertile Cut-offs for U5MR were 20.7 and 23.5 and for its annual rate of reduction were 5.55% and 6.56%.
The tertile cut-offs for NMR were 11.2 and 12.6 and for its annual rate of reduction were 4.26% and 5.23%.

Chhattisgarh

A U5MR

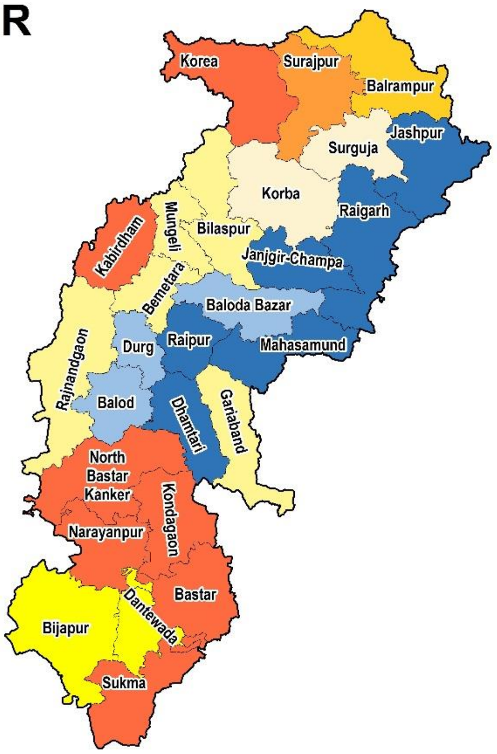

B NMR

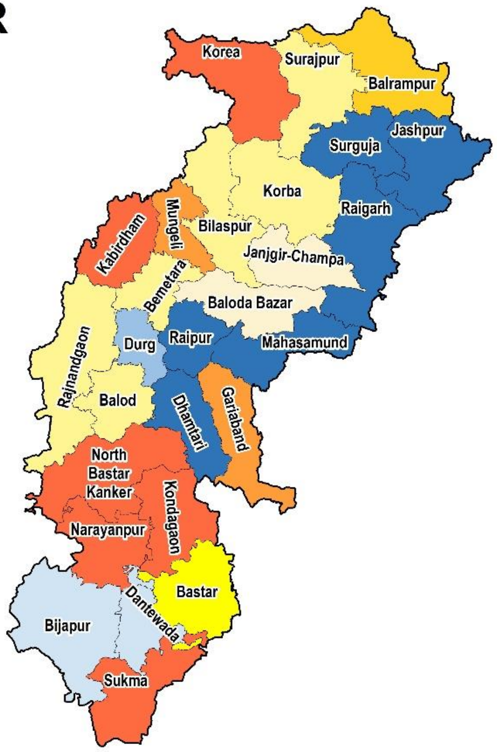

High, medium, and low groups based on tertiles of rate in 2017 and the annual rate of reduction (ARoR) from 2010 to 2017 among the districts within the state

- High rate and low ARoR
- High rate and medium ARoR
- High rate and high ARoR
- Medium rate and low ARoR
- Medium rate and medium ARoR
- Medium rate and high ARoR
- Low rate and low ARoR
- Low rate and medium ARoR
- Low rate and high ARoR

The tertile cut-offs for U5MR were 46.1 and 50.9 and for its annual rate of reduction were 4.28% and 5.52%.
The tertile cut-offs for NMR were 28.2 and 30.7 and for its annual rate of reduction were 3.43% and 4.64%.

Gujarat

A U5MR

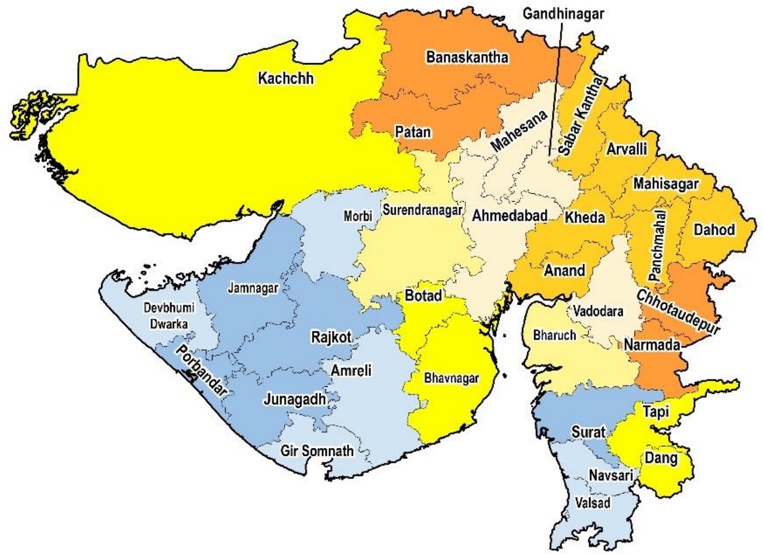

High, medium, and low groups based on tertiles of rate in 2017 and the annual rate of reduction (ARoR) from 2010 to 2017 among the districts within the state

- High rate and low ARoR
- High rate and medium ARoR
- High rate and high ARoR
- Medium rate and low ARoR
- Medium rate and medium ARoR
- Medium rate and high ARoR
- Low rate and low ARoR
- Low rate and medium ARoR
- Low rate and high ARoR

B NMR

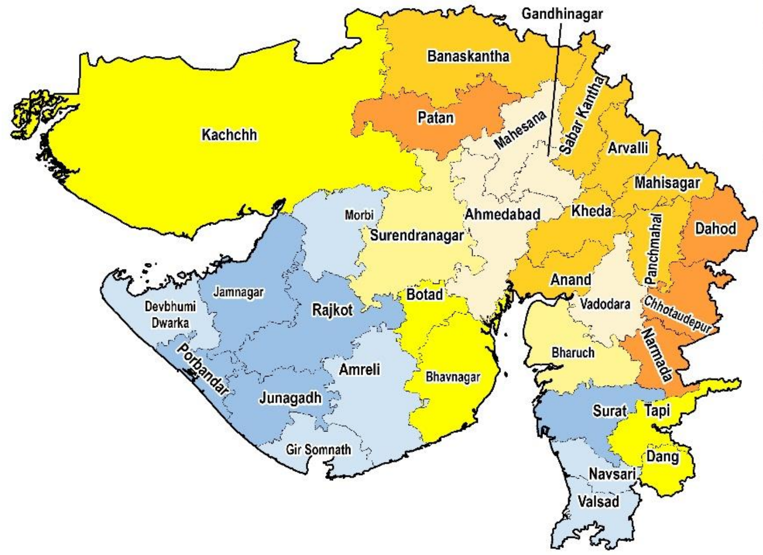

The tertile cut-offs for U5MR were 35.2 and 44.7 and for its annual rate of reduction were 2.74% and 4.12%.
The tertile cut-offs for NMR were 21.4 and 27.0 and for its annual rate of reduction were 1.72% and 2.98%.

Haryana

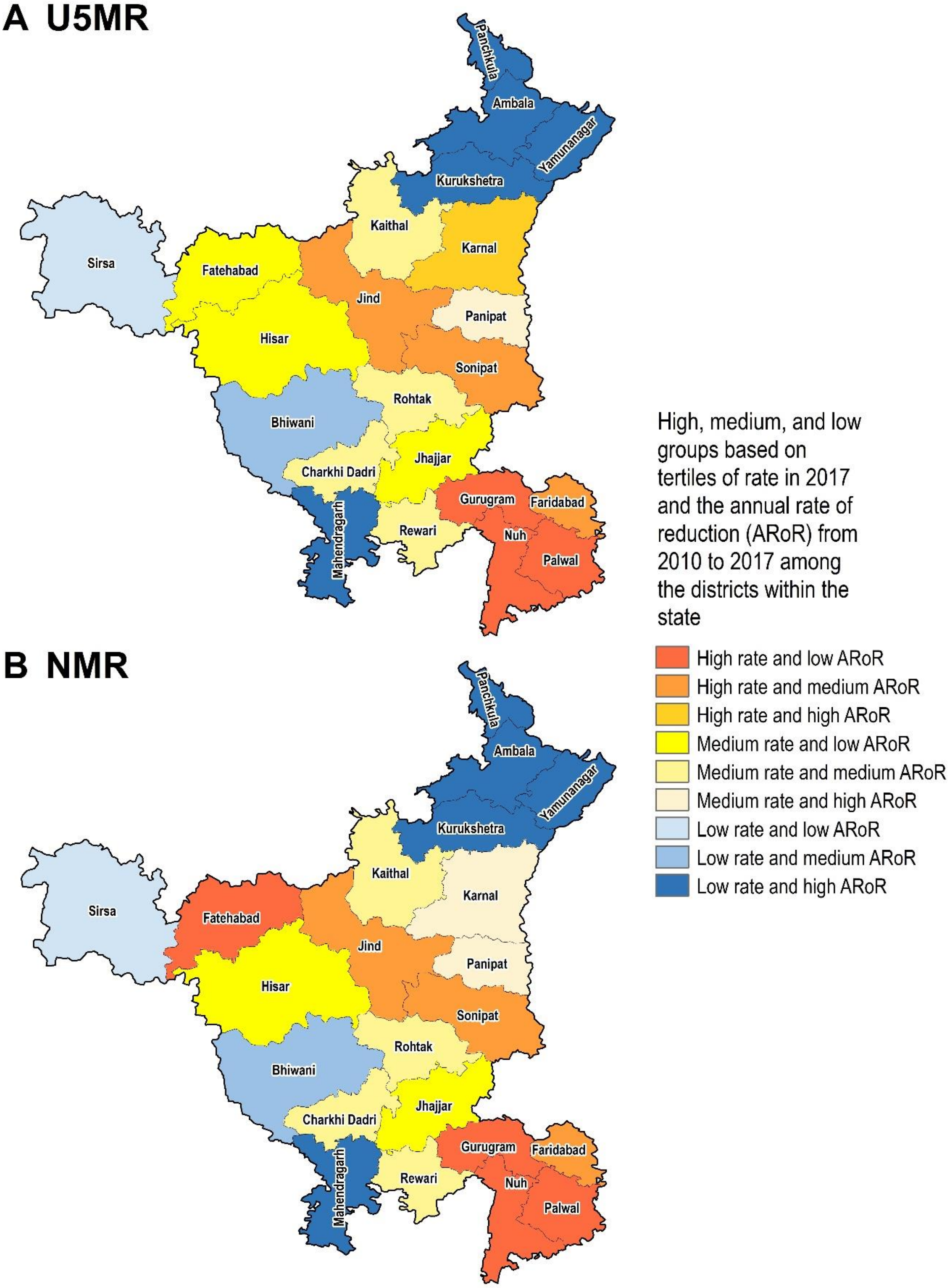

The tertile cut-offs for U5MR were 37.4 and 39.5 and for its annual rate of reduction were 4.64% and 4.91%.
The tertile cut-offs for NMR were 19.9 and 20.3 and for its annual rate of reduction were 3.52% and 3.77%.

Jammu and Kashmir

A U5MR

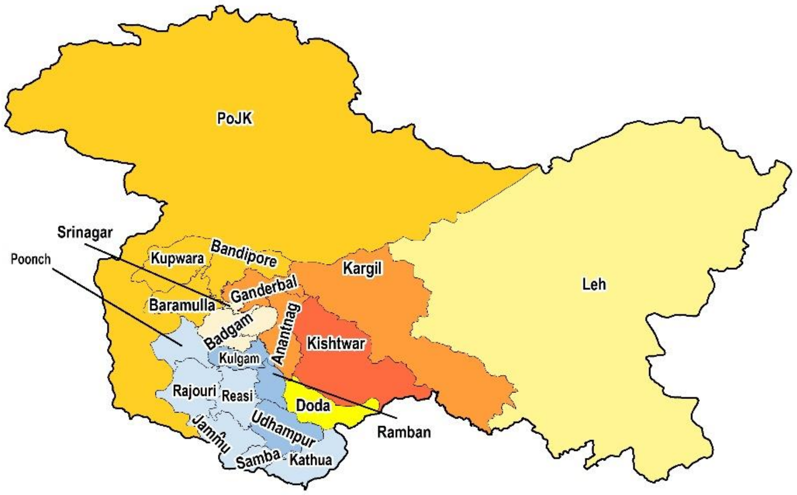

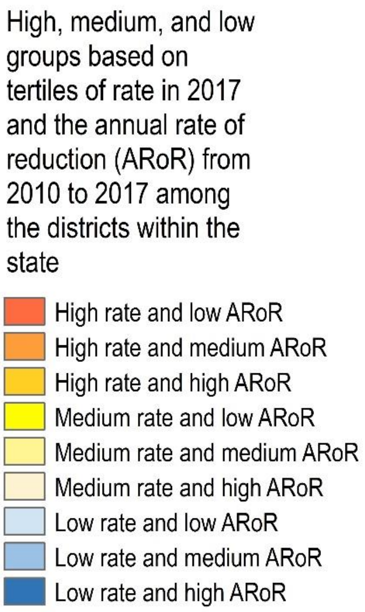

B NMR

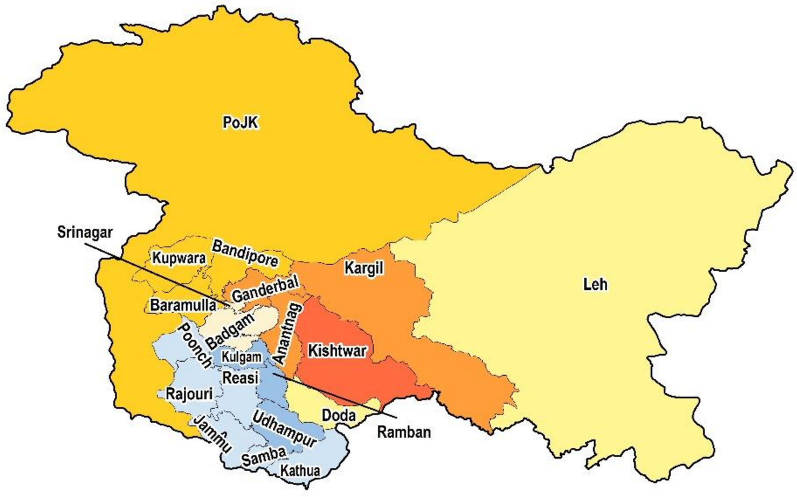

The tertile cut-offs for U5MR were 32.7 and 35.7 and for its annual rate of reduction were 3.38% and 4.10%.
The tertile cut-offs for NMR were 19.4 and 21.5 and for its annual rate of reduction were 2.40% and 3.22%.

Jharkhand

A U5MR

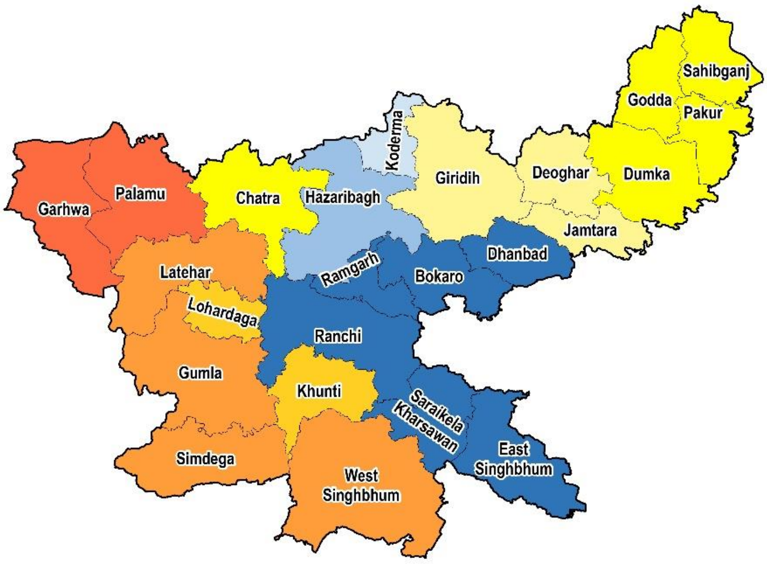

B NMR

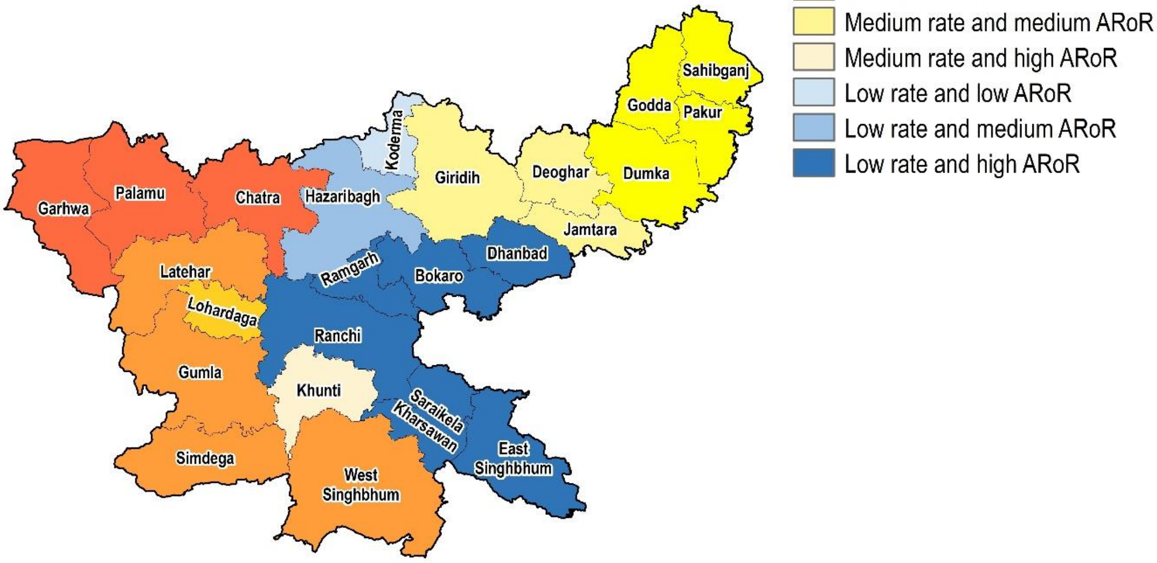

The tertile cut-offs for U5MR were 42.4 and 47.5 and for its annual rate of reduction were 3.85% and 4.87%.
The tertile cut-offs for NMR were 22.3 and 24.9 and for its annual rate of reduction were 2.71% and 3.72%.

Karnataka

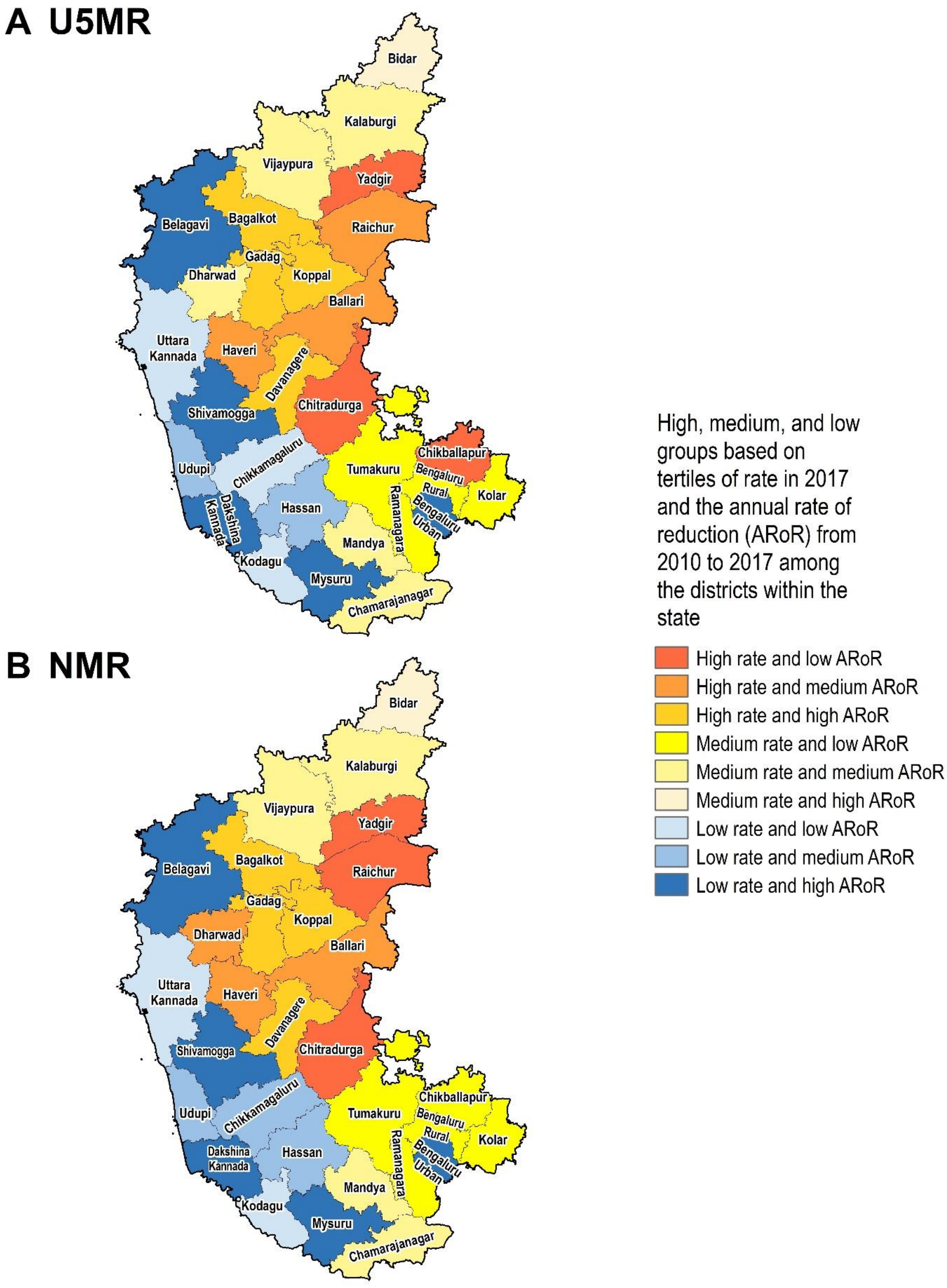

The tertile cut-offs for U5MR were 28.0 and 35.5 and for its annual rate of reduction were 4.67% and 4.97%.
The tertile cut-offs for NMR were 16.7 and 20.5 and for its annual rate of reduction were 3.81% and 4.13%.

Madhya Pradesh

A U5MR

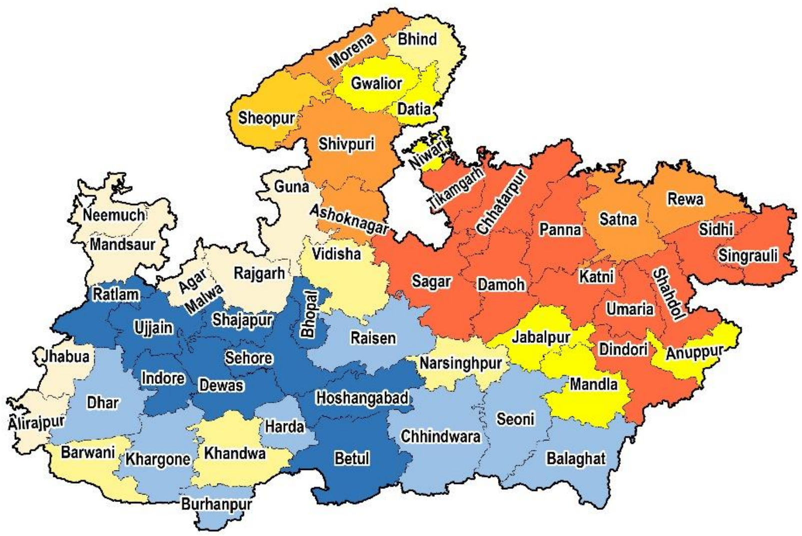

High, medium, and low groups based on tertiles of rate in 2017 and the annual rate of reduction (ARoR) from 2010 to 2017 among the districts within the state

- High rate and low ARoR
- High rate and medium ARoR
- High rate and high ARoR
- Medium rate and low ARoR
- Medium rate and medium ARoR
- Medium rate and high ARoR
- Low rate and low ARoR
- Low rate and medium ARoR
- Low rate and high ARoR

B NMR

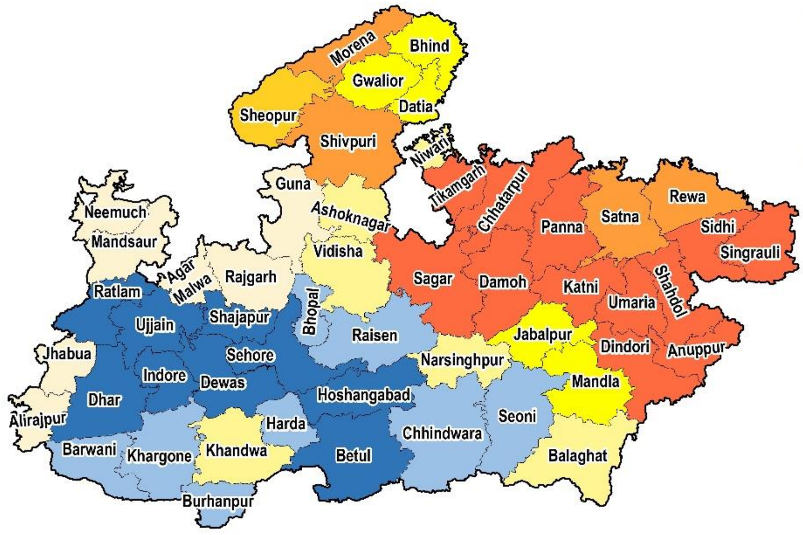

The tertile cut-offs for U5MR were 45.2 and 55.6 and for its annual rate of reduction were 5.13% and 5.94%.
The tertile cut-offs for NMR were 24.3 and 29.9 and for its annual rate of reduction were 3.91% and 4.93%.

Maharashtra

A U5MR

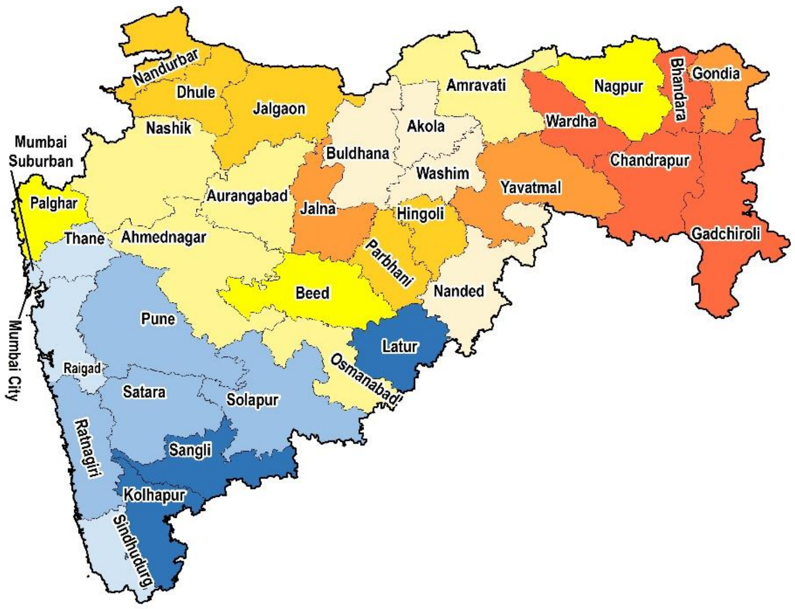

High, medium, and low groups based on tertiles of rate in 2017 and the annual rate of reduction (ARoR) from 2010 to 2017 among the districts within the state

- High rate and low ARoR
- High rate and medium ARoR
- High rate and high ARoR
- Medium rate and low ARoR
- Medium rate and medium ARoR
- Medium rate and high ARoR
- Low rate and low ARoR
- Low rate and medium ARoR
- Low rate and high ARoR

B NMR

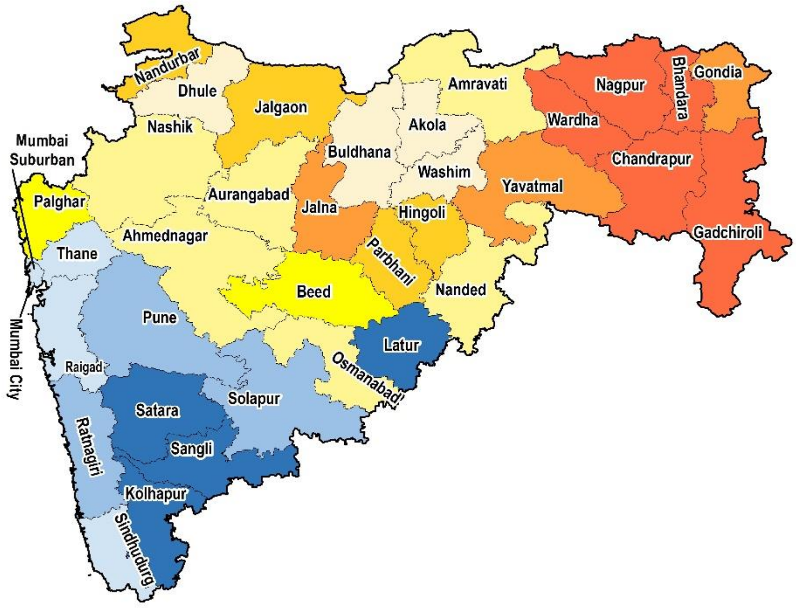

The tertile cut-offs for U5MR were 24.1 and 30.1 and for its annual rate of reduction were 4.74% and 5.16%.
The tertile cut-offs for NMR were 15.7 and 20.1 and for its annual rate of reduction were 3.86% and 4.35%.

Odisha

A U5MR

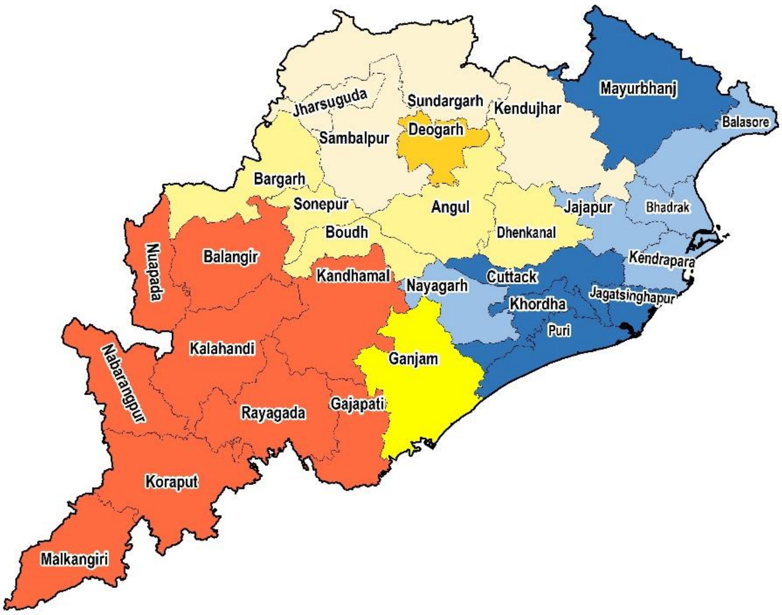

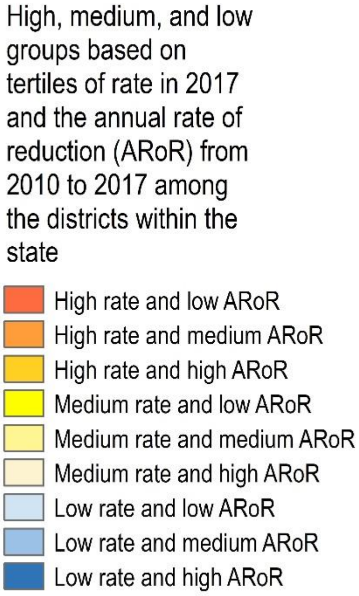

B NMR

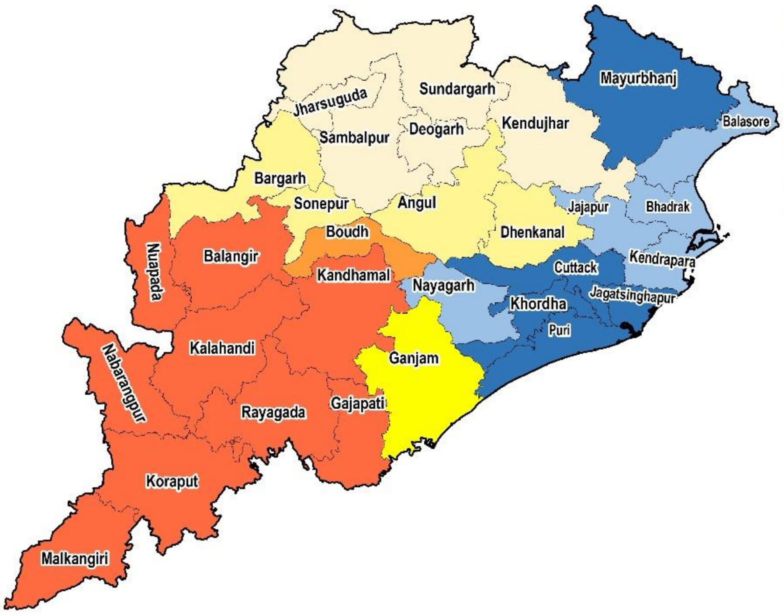

The tertile cut-offs for U5MR were 45.3 and 56.8 and for its annual rate of reduction were 4.53% and 5.16%.
The tertile cut-offs for NMR were 23.6 and 30.1 and for its annual rate of reduction were 3.66% and 4.24%.

Punjab

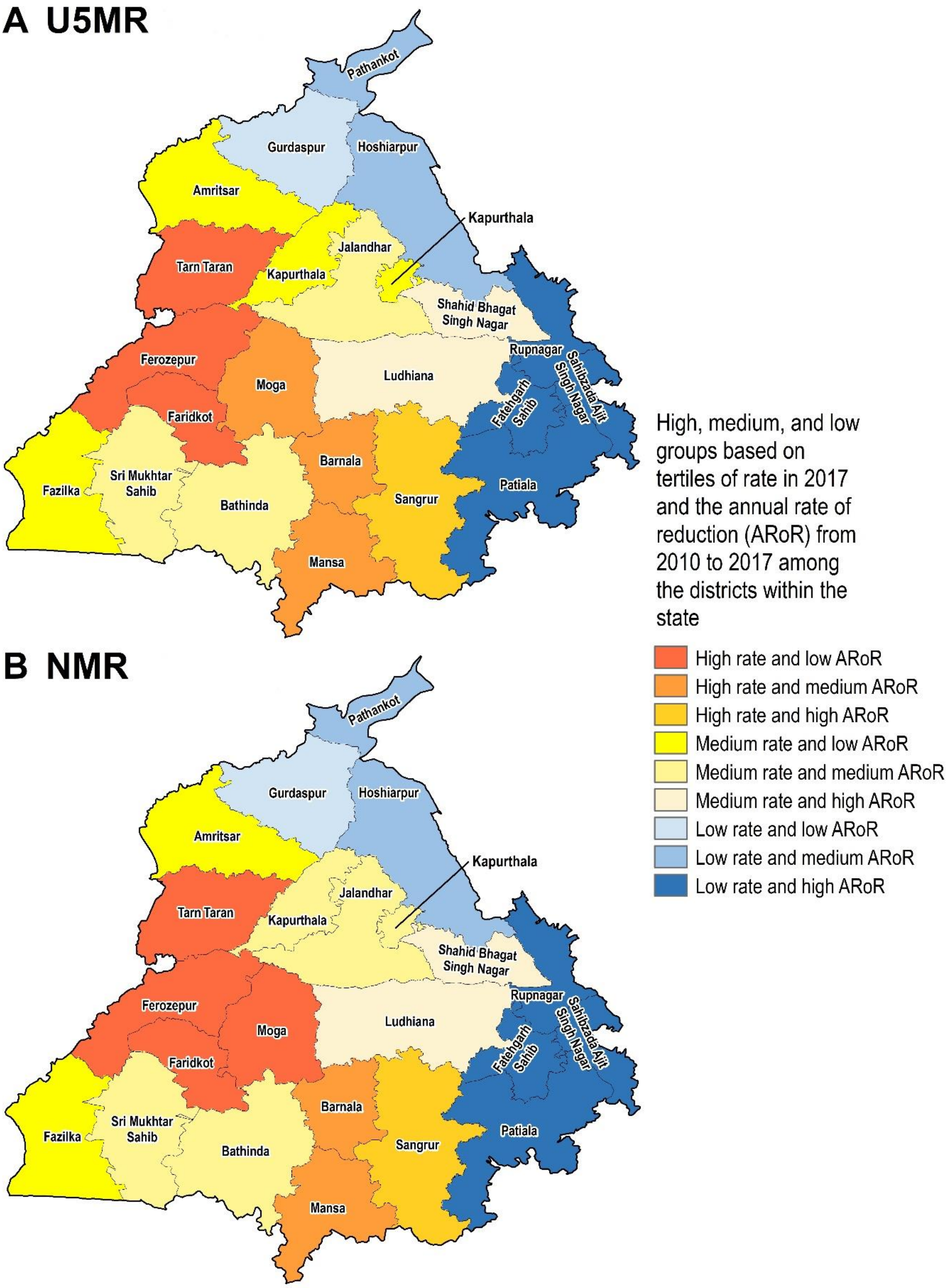

The tertile cut-offs for U5MR were 27.5 and 31.3 and for its annual rate of reduction were 4.04% and 5.28%.
The tertile cut-offs for NMR were 15.2 and 17.4 and for its annual rate of reduction were 3.07% and 4.23%.

Rajasthan

A U5MR

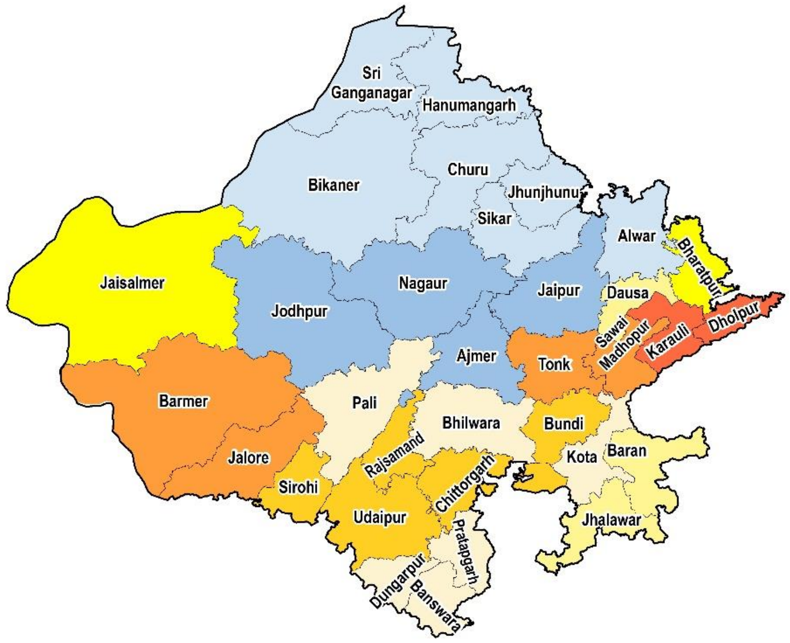

B NMR

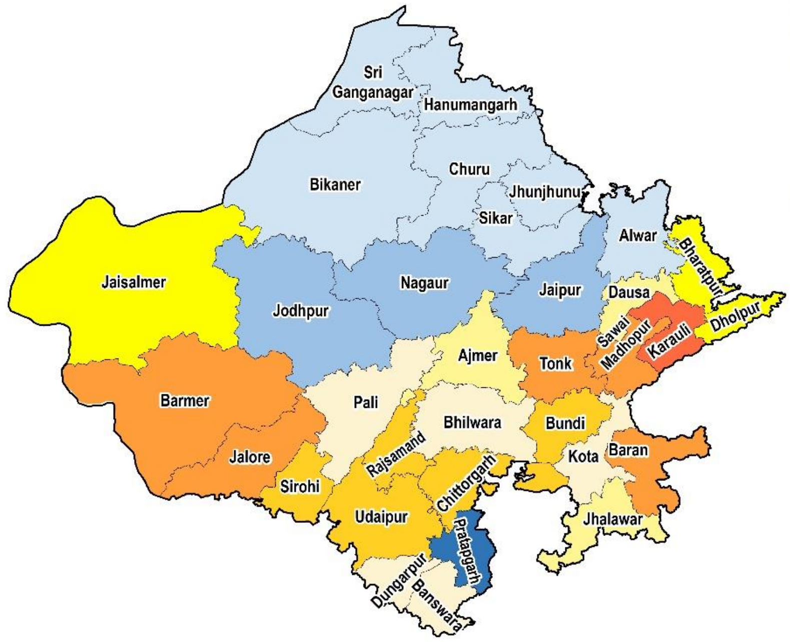

The tertile cut-offs for U5MR were 47.0 and 58.1 and for its annual rate of reduction were 3.48% and 4.96%.
The tertile cut-offs for NMR were 25.6 and 32.0 and for its annual rate of reduction were 2.49% and 4.00%.

Tamil Nadu

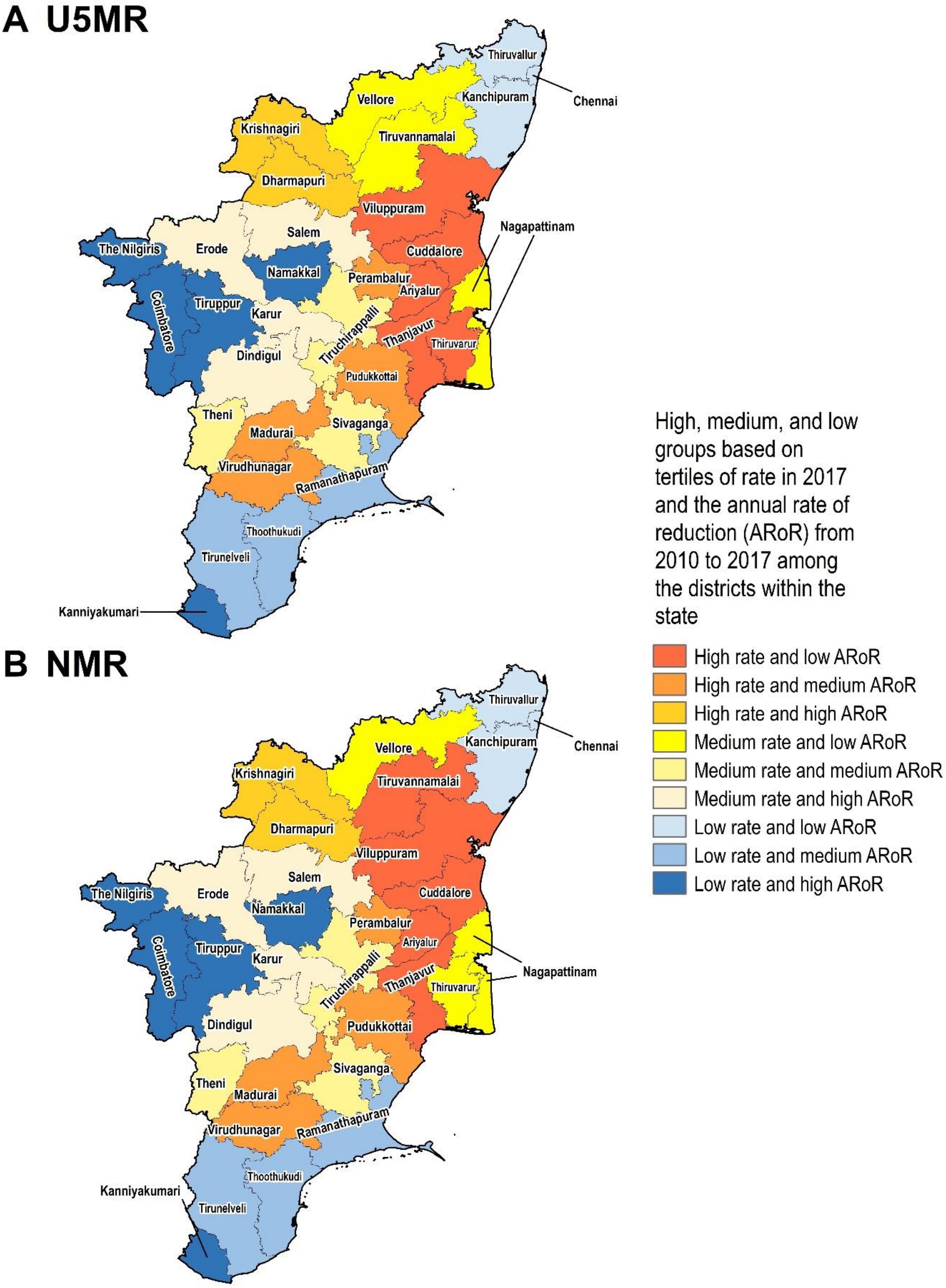

The tertile cut-offs for U5MR were 19.6 and 21.8 and for its annual rate of reduction were 5.73% and 6.69%.
The tertile cut-offs for NMR were 11.4 and 12.6 and for its annual rate of reduction were 5.13% and 6.11%.

Telangana

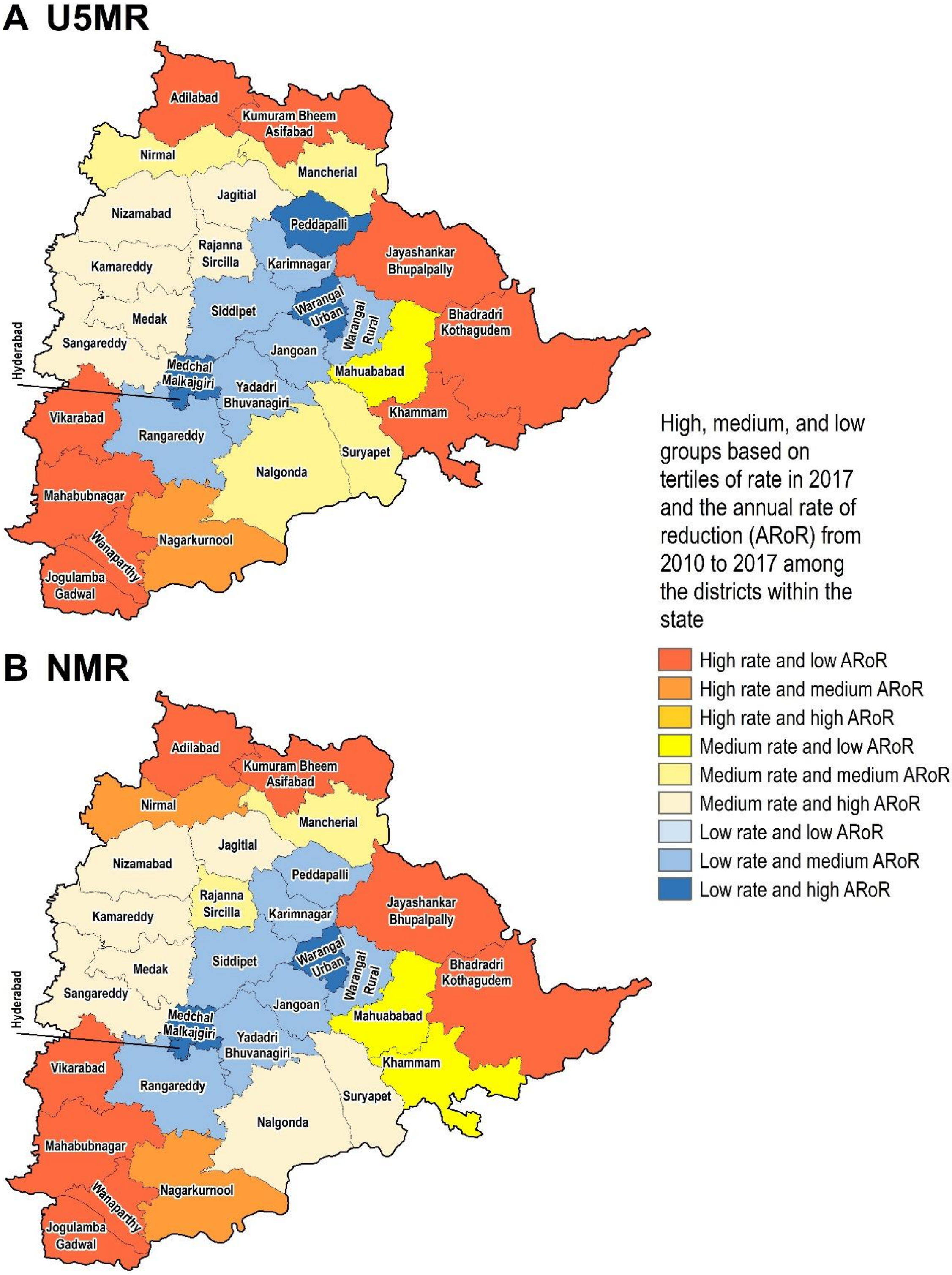

The tertile cut-offs for U5MR were 27.4 and 30.1 and for its annual rate of reduction were 6.22% and 6.65%.
The tertile cut-offs for NMR were 17.1 and 18.7 and for its annual rate of reduction were 4.85% and 5.28%.

West Bengal

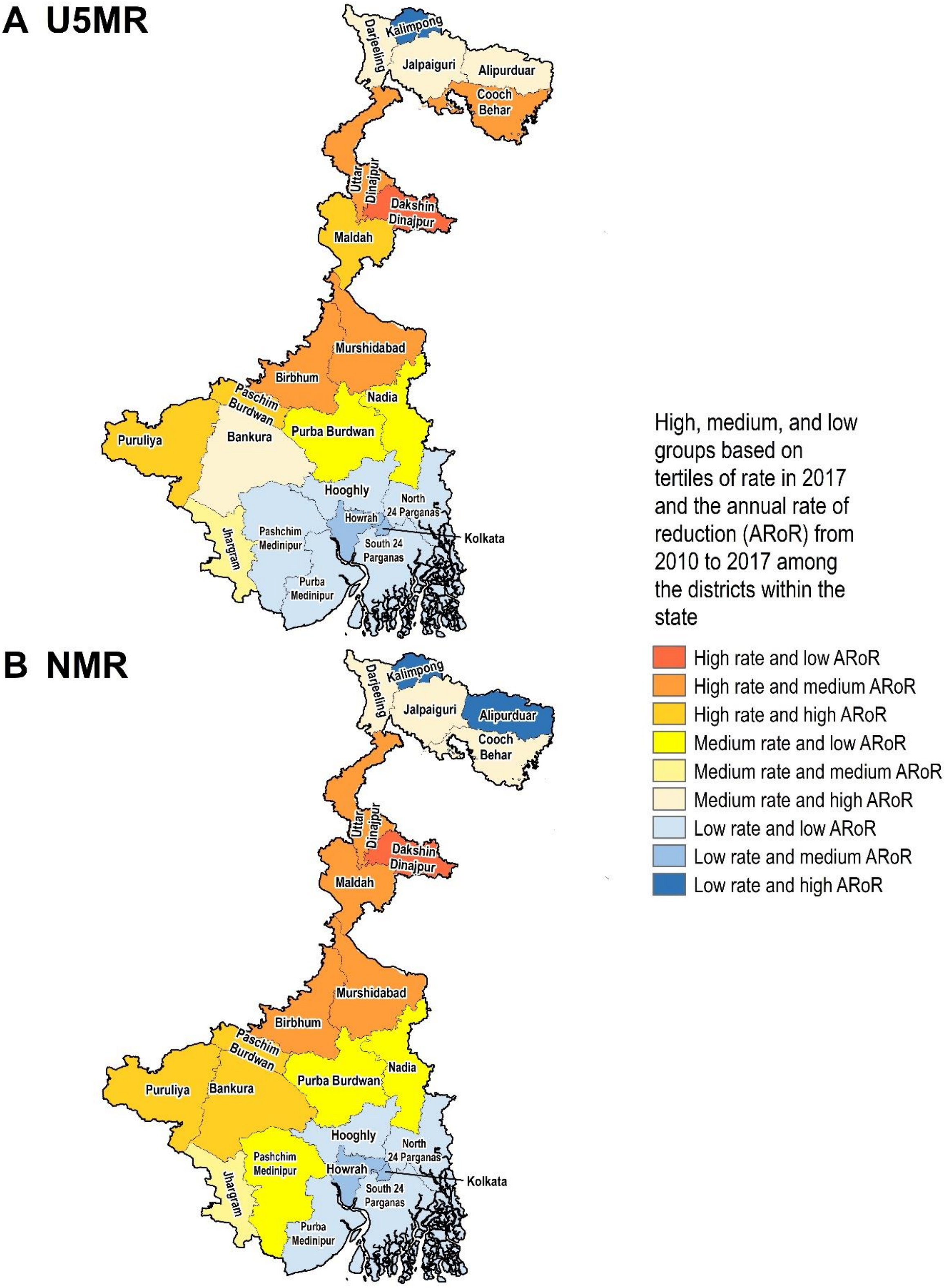

The tertile cut-offs for U5MR were 28.3 and 32.5 and for its annual rate of reduction were 5.20% and 5.54%.
The tertile cut-offs for NMR were 16.9 and 19.8 and for its annual rate of reduction were 4.08% and 4.35%.

14. Causes of under-5 death in the states of India, 2000 and 2017

State*	Percentage of under-5 deaths (95% uncertainty interval)											
	Neonatal preterm birth		Neonatal encephalopathy due to birth asphyxia and trauma		Neonatal sepsis and other neonatal infections		Haemolytic disease and neonatal jaundice and other neonatal disorders		Lower respiratory infections		Diarrhoeal diseases	
	2000	2017	2000	2017	2000	2017	2000	2017	2000	2017	2000	2017
**India**	**12.5 (11.4-14.1)**	**15.6 (13.5-17.8)**	**7.4 (6.6-8.6)**	**8.1 (7.0-11.0)**	**2.5 (1.8-3.0)**	**3.5 (2.3-4.2)**	**10.9 (9.4-11.9)**	**14.3 (11.7-16.1)**	**19.6 (18.0-21.1)**	**17.9 (16.6-19.4)**	**14.9 (12.7-17.2)**	**9.9 (8.5-11.3)**
**Low SDI**	**10.6 (9.2-13.3)**	**14.0 (11.4-17.2)**	**6.6 (5.7-7.9)**	**7.3 (6.1-10.5)**	**2.6 (1.7-3.3)**	**3.6 (2.1-4.5)**	**10.9 (8.7-12.3)**	**14.5 (11.2-16.9)**	**21.2 (19.4-22.9)**	**19.2 (17.6-21.0)**	**16.7 (14.3-19.5)**	**11.6 (10.0-13.4)**
Bihar	9.4 (7.1-12.7)	10.8 (7.9-16.5)	6.3 (4.7-8.1)	9.9 (8.0-12.5)	2.3 (1.4-3.3)	2.9 (1.8-4.1)	9.5 (7.4-11.8)	14.6 (10.8-18.8)	22.6 (19.6-25.8)	19.6 (17.2-22.1)	18.7 (14.7-22.8)	16.2 (13.5-19.3)
Madhya Pradesh	12.9 (10.6-15.1)	16.6 (13.3-19.5)	4.9 (3.5-8.0)	4.9 (3.0-10.2)	1.8 (1.2-2.5)	2.7 (1.9-3.7)	12.6 (9.6-15.1)	16.2 (11.4-20.9)	23.0 (20.4-25.4)	20.3 (18.0-22.8)	13.5 (10.4-17.2)	6.9 (5.2-9.1)
Jharkhand	10.6 (8.5-13.3)	13.2 (9.7-16.8)	6.8 (5.2-8.8)	8.6 (6.4-11.0)	2.8 (1.7-3.9)	3.6 (2.2-5.0)	10.4 (8.0-13.1)	13.0 (9.7-17.3)	19.7 (16.9-22.6)	17.9 (15.5-20.5)	16.4 (13.0-19.9)	12.3 (9.2-15.9)
Uttar Pradesh	9.5 (7.2-13.5)	14.1 (11.0-17.6)	6.7 (5.3-8.5)	6.4 (4.6-10.6)	2.9 (1.8-4.0)	4.0 (2.3-5.4)	10.4 (7.8-13.1)	14.2 (10.5-18.0)	20.3 (17.9-22.7)	17.6 (15.3-20.2)	18.0 (14.5-21.9)	13.1 (10.3-15.8)
Rajasthan	13.2 (10.9-15.9)	16.3 (13.0-19.2)	5.4 (3.8-8.7)	5.8 (4.0-11.2)	2.8 (1.6-3.7)	3.8 (2.0-5.1)	10.9 (8.0-14.0)	14.1 (10.1-18.4)	26.0 (22.8-29.2)	27.1 (24.3-30.0)	14.1 (10.6-18)	6.4 (4.8-8.2)
Chhattisgarh	14.6 (12.0-17.4)	18.9 (15.0-22.8)	8.8 (6.8-11.1)	9.1 (6.8-13.6)	1.6 (1.1-2.5)	2.5 (1.7-3.3)	13.8 (10.5-17.1)	17.6 (12.3-22.7)	19.0 (16.5-21.5)	17.7 (15.4-20.2)	11.1 (8.6-13.8)	6.3 (4.6-8.1)
Odisha	9.5 (7.4-13.6)	11.5 (8.7-16.8)	9.1 (6.9-11.7)	10.1 (8.0-12.4)	2.6 (1.8-3.6)	4.3 (2.7-5.8)	12.0 (9.4-15.1)	14.6 (10.9-18.9)	17.7 (15.3-20.1)	15.1 (12.7-17.7)	14.7 (11.2-19.3)	8.5 (6.1-10.9)
Assam	13.4 (11.3-15.9)	14.9 (12.0-18.1)	8.2 (6.5-10.1)	9.1 (7.1-11.4)	3.8 (2.3-5.1)	3.6 (2.1-4.8.0)	12.3 (9.9-15.0)	12.5 (9.4-15.8)	15.1 (12.9-17.5)	16.5 (14.3-18.7)	19.7 (15.0-23.9)	10.4 (8.0-13.1)
**Middle SDI**	**16.0 (13.7-17.6)**	**19.7 (17.3-21.7)**	**8.7 (7.8-9.8)**	**10.1 (8.9-12.3)**	**2.1 (1.7-2.9)**	**2.9 (2.3-4.1)**	**10.9 (9.7-12.3)**	**13.6 (11.8-15.4)**	**16.9 (15.1-18.3)**	**15.2 (14.1-16.5)**	**11.4 (9.6-13.3)**	**5.6 (4.7-6.5)**
Andhra Pradesh	17.1 (13.3-20.5)	22.2 (17.7-25.7)	10.0 (8.0-12.4)	12.4 (10.2-14.8)	1.5 (1.0-2.7)	2.4 (1.7-4.4)	8.9 (6.9-11.8)	11.3 (7.9-16.2)	15.6 (13.2-18.2)	12.1 (10.4-14.0)	13.6 (10.6-16.9)	8.4 (6.6-10.3)
West Bengal	15.0 (12.2-17.9)	19.0 (16.0-22.3)	6.8 (5.3-8.9)	8.1 (6.2-11.9)	2.1 (1.5-3.0)	3.1 (2.3-4.3)	12.1 (9.8-14.5)	14.9 (11.6-18.9)	18.3 (15.6-20.5)	18.1 (16.0-20.2)	9.5 (7.1-12.6)	4.3 (3.2-5.7)
Tripura	15.3 (12.7-18.4)	17.6 (14.6-21.0)	6.6 (4.7-9.6)	6.8 (4.9-11.1)	4.0 (2.8-5.3)	5.1 (3.4-6.8)	9.1 (6.9-11.9)	10.7 (8.0-14.2)	23.4 (20.0-26.8)	23.0 (20.2-26.0)	8.5 (6.1-11.2)	5.8 (4.4-7.6)
Arunachal Pradesh	10.4 (8.5-12.8)	15.8 (13.1-18.7)	6.1 (4.5-7.9)	8.1 (5.8-10.7)	3.5 (2.2-5.1)	4.6 (3.1-6.5)	9.6 (7.5-12.0)	12.9 (9.8-16.9)	19.5 (16.5-22.7)	16.5 (14.2-18.9)	15.1 (11.6-19.4)	8.7 (6.2-11.5)
Meghalaya	9.8 (7.7-12.6)	11.9 (8.7-16.5)	8.3 (5.8-10.9)	10.6 (7.9-13.5)	2.7 (1.8-3.9)	3.5 (2.3-5.0)	7.0 (5.3-9.4)	8.5 (6.3-11.3)	21.8 (17.9-26.4)	19.2 (16.2-22.6)	13.4 (9.3-18.1)	7.5 (5.4-9.6)
Karnataka	19.3 (15.2-22.4)	22.4 (17.9-25.3)	9.0 (7.3-11.2)	10.3 (8.2-12.9)	2.2 (1.6-3.3)	2.7 (2.0-4.2)	10.9 (8.7-13.5)	12.1 (9.5-15.4)	13.7 (11.5-15.6)	11.3 (9.9-12.8)	8.1 (6.2-10.1)	4.1 (3.2-5.2)
Telangana	18.8 (14.4-22.7)	24.2 (19.1-27.8)	10.1 (7.9-12.8)	12.8 (10.2-15.7)	1.6 (1.1-3.0)	2.6 (1.8-4.9)	9.3 (6.8-12.7)	11.9 (8.4-17.2)	15.3 (13.1-17.8)	10.8 (9.2-12.6)	14.3 (11.2-18.1)	7.1 (5.4-9.0)
Gujarat	15.7 (13.5-18.0)	20.2 (17.1-23.3)	10.3 (8.4-12.5)	11.5 (9.4-13.8)	2.1 (1.6-3.0)	2.5 (1.8-4.0)	11.2 (9.0-13.5)	13.5 (10.6-17.1)	16.8 (14.6-19.0)	14.7 (12.8-16.6)	10.4 (8.1-13.2)	5.1 (3.9-6.4)
Manipur	11.2 (9.1-13.8)	15.5 (12.1-20.5)	7.8 (5.9-10.1)	9.6 (7.2-12.4)	3.3 (2.0-4.4)	3.9 (2.6-5.2)	11.4 (8.8-14.6)	13.3 (9.5-18.0)	15.3 (12.6-17.7)	14.7 (12.3-17.1)	14.5 (10.8-18.6)	7.6 (5.6-9.5)
Jammu and Kashmir[†]	12.3 (10.0-14.6)	14.1 (11.1-19.0)	7.1 (5.6-8.7)	7.3 (5.3-10.9)	3.5 (2.3-4.7)	4.9 (3.0-6.6)	11.7 (9.6-14.2)	17.3 (12.8-22.0)	24.4 (21.3-27.1)	22.9 (19.8-25.8)	10.4 (7.7-13.5)	4.2 (2.9-5.7)
Haryana	10.1 (8.2-12.4)	13.5 (11.1-16.3)	6.9 (5.6-8.5)	8.1 (6.2-10.6)	2.2 (1.5-2.9)	2.9 (2.1-4.0)	12.3 (9.6-14.8)	16.5 (11.8-20.8)	19.4 (16.7-22.2)	17.9 (15.7-19.9)	16.9 (12.9-20.8)	7.7 (5.9-9.8)
**High SDI**	**17.6 (15.0-19.5)**	**19.6 (17.3-22.3)**	**9.6 (8.5-10.8)**	**10.3 (8.9-12.6)**	**2.5 (2.0-3.4)**	**3.6 (2.8-4.9)**	**10.8 (9.6-12.5)**	**13.8 (12.0-15.9)**	**15.4 (14.0-16.5)**	**13.5 (12.3-14.5)**	**10.2 (8.8-11.7)**	**4.9 (4.1-5.7)**
Uttarakhand	10.7 (8.4-13.5)	14.1 (10.8-18.1)	6.8 (4.9-9.1)	8.4 (6.4-11.7)	3.3 (2.0-4.5)	5.6 (2.9-7.6)	10.2 (7.9-12.9)	13.0 (10.0-16.7)	19.5 (17.1-22.1)	19.7 (17.2-22.1)	17.7 (13.6-21.9)	5.9 (4.4-7.6)
Tamil Nadu	17.4 (14.1-20.0)	20.7 (16.1-23.7)	11.7 (9.0-14.2)	12.3 (9.5-15.1)	1.7 (1.1-3.3)	2.3 (1.6-4.9)	7.1 (5.3-10.8)	8.5 (6.2-13.3)	14.4 (12.6-16.0)	12.1 (10.5-13.8)	9.8 (7.8-11.7)	4.8 (3.6-6.1)
Mizoram	9.8 (7.6-12.1)	12.1 (9.5-15.8)	8.0 (5.7-10.3)	8.8 (7.1-10.5)	3.3 (2.3-4.6)	3.6 (2.6-4.9)	9.8 (7.6-12.8)	12.2 (9.1-15.5)	23.2 (19.8-26.6)	24.3 (21.3-27.1)	8.6 (6.3-11.7)	3.0 (2.2-3.8)
Maharashtra	21.4 (16.2-24.9)	23.3 (18.8-27.3)	8.2 (6.3-11.7)	9.5 (7.2-13.9)	2.7 (2.1-3.8)	4.0 (2.9-5.6)	11.9 (9.5-14.4)	14.7 (11.4-18.3)	15.4 (13.6-17.0)	12.2 (10.7-13.7)	8.4 (6.7-10.2)	4.2 (3.2-5.3)
Punjab	10.7 (8.5-14.0)	13.0 (10.3-18.0)	10.1 (7.7-12.4)	10.7 (8.6-13.1)	3.1 (2.1-3.9)	3.7 (2.4-4.9)	13.0 (10.7-15.8)	15.6 (11.8-19.6)	15.8 (13.5-18.1)	15.6 (13.6-17.7)	16.8 (13.0-21.0)	7.8 (5.9-9.5)
Sikkim	10.1 (8.0-12.6)	11.5 (9.2-14.2)	4.7 (3.3-7.2)	4.9 (3.5-8.2)	3.7 (2.2-5.1)	5.7 (3.3-7.7)	11.3 (8.4-15.0)	12.8 (9.2-17.1)	25.4 (21.6-28.4)	23.9 (21.3-26.6)	14.4 (10.6-18.4)	6.5 (5.1-8.1)
Nagaland	10.7 (8.2-13.4)	12.4 (9.3-17.1)	6.6 (4.9-8.9)	7.2 (4.6-10.0)	4.3 (1.9-6.0)	5.1 (2.3-7.1)	8.9 (6.8-12.3)	11.2 (7.9-15.1)	23.3 (19.6-27.1)	25.1 (21.4-28.6)	8.2 (5.6-11.1)	3.9 (2.6-5.3)
Himachal Pradesh	12.8 (10.3-15.8)	15.4 (11.7-19.6)	12.3 (8.7-15.7)	12.1 (9.6-14.8)	1.9 (1.3-2.7)	2.8 (2.0-4.1)	12.8 (10.0-15.8)	16.0 (11.9-21.0)	19.4 (16.6-22.5)	17.1 (14.6-19.3)	14.0 (9.9-18.2)	5.0 (3.6-6.6)
UTs other than Delhi	18.6 (15.7-21.6)	20.3 (16.7-23.8)	11.6 (9.4-14)	11.7 (9.3-14.4)	2.3 (1.7-3.5)	3.2 (2.3-4.3)	10.9 (8.2-14.7)	12.7 (9.5-16.9)	15.1 (13.3-17.0)	13.7 (11.9-15.6)	9.0 (7.2-10.9)	4.4 (3.3-5.5)
Kerala	19.0 (15.1-22.6)	19.6 (15.8-23.4)	9.3 (7.0-12.3)	10.1 (7.8-13.0)	2.5 (1.7-5.1)	4.2 (3.0-7.1)	7.2 (5.1-12.9)	8.0 (5.8-14.4)	9.9 (7.9-11.9)	8.5 (7.0-9.9)	1.4 (0.9-1.8)	0.6 (0.4-0.8)
Delhi	13.8 (11.0-17.5)	16.8 (12.6-23.1)	10.5 (8.2-13.4)	11.5 (8.3-14.9)	1.9 (1.3-3.1)	2.5 (1.6-4.2)	16.7 (11.7-21.3)	20.4 (13.5-27.6)	15.3 (13.0-18.1)	13.4 (11.6-15.5)	12.5 (9.6-15.9)	6.1 (4.6-7.7)
Goa	15.9 (13.3-18.6)	14.1 (11.4-17.3)	7.8 (5.6-10.7)	7.9 (5.8-10.2)	2.3 (1.4-3.4)	3.2 (1.5-4.9)	16.6 (13.5-20.0)	18.2 (12.5-26.0)	18.6 (16.2-21.0)	16.2 (14.0-18.3)	5.0 (3.9-6.3)	3.4 (2.6-4.3)

States*	Percentage of under-5 deaths (95% uncertainty interval)									
	Measles		Congenital birth defects		Injuries		Other communicable diseases		Other non-communicable diseases	
	2000	2017	2000	2017	2000	2017	2000	2017	2000	2017
**India**	**4.1 (1.6-8.5)**	**1.6 (0.6-3.4)**	**4.4 (4.1-5.0)**	**8.0 (7.1-8.7)**	**3.5 (2.9-4.1)**	**4.1 (3.4-4.9)**	**17.3 (14.7-20.7)**	**13.8 (11.4-16.3)**	**2.9 (2.2-3.8)**	**3.4 (2.8-4.2)**
**Low SDI**	**4.0 (1.5-8.6)**	**1.5 (0.5-3.4)**	**2.9 (2.6-3.7)**	**6.9 (6.0-7.8)**	**3.5 (2.8-4.1)**	**4.1 (3.3-5.0)**	**18.3 (15.3-21.3)**	**14.0 (11.7-16.6)**	**2.8 (2.1-3.8)**	**3.2 (2.6-4.1)**
Bihar	5.4 (1.9-11.6)	1.8 (0.6-3.9)	2.5 (2.0-3.2)	5.3.0 (4.3-6.2)	3.8 (2.9-4.8)	3.9 (2.9-5.0)	16.7 (12.7-22.7)	12.0 (8.9-16.3)	2.9 (2.0-4.0)	3.1 (2.3-4.0)
Madhya Pradesh	3.7 (1.3-8)	2.1 (0.8-4.8)	3.4 (2.9-4.1)	6.7 (5.6-7.8)	3.6 (2.8-4.6)	4.7 (3.6-6.2)	17.9 (14.0-22.0)	15.4 (11.7-20.0)	2.8 (2.0-3.9)	3.5 (2.6-4.7)
Jharkhand	5 (1.9-10.7)	1.9 (0.6-4.6)	2.9 (2.3-4.8)	7.0 (5.9-8.3)	4.5 (3.4-5.9)	5.3 (3.7-7.4)	18.2 (14.1-23.1)	13.6 (9.3-19.7)	2.7 (2.0-3.7)	3.6 (2.6-4.9)
Uttar Pradesh	3.4 (1.2-7.5)	1.3 (0.4-2.9)	2.9 (2.3-4.0)	7.9 (6.5-9.4)	3.3 (2.6-4.2)	3.8 (2.8-5.0)	19.7 (16.0-24.0)	14.5 (11.3-18.7)	2.8 (2.0-4.0)	3.1 (2.3-4.3)
Rajasthan	4.0 (1.5-8.7)	1.8 (0.6-3.9)	2.2 (1.8-2.7)	5.7 (4.7-6.7)	3.0 (2.4-3.8)	3.4 (2.7-4.4)	15.9 (12.4-20.4)	12.8 (9.9-16.6)	2.5 (1.7-3.6)	2.8 (2.1-4.0)
Chhattisgarh	4.3 (1.6-9.3)	1.7 (0.6-3.9)	2.9 (2.5-3.4)	5.1 (4.2-6.2)	3.5 (2.6-4.4)	4.0 (2.9-5.4)	17.8 (13.7-22.7)	13.7 (10.3-18.5)	2.6 (1.9-3.4)	3.4 (2.5-4.4)
Odisha	3.6 (1.3-8)	0.7 (0.2-1.7)	4.1 (3.5-4.9)	6.9 (5.7-8.2)	3.3 (2.6-4.1)	5.4 (4.3-6.6)	20.2 (15.0-26.0)	18.3 (12.7-24.5)	3.2 (2.3-4.3)	4.6 (3.4-6.2)
Assam	4.2 (1.6-8.9)	1.5 (0.5-3.3)	3.9 (3.3-4.6)	9.5 (6.9-11.6)	3.2 (2.5-3.9)	4.7 (3.8-6.0)	13.7 (10.4-19.4)	13.8 (10.0-18.8)	2.6 (1.9-3.4)	3.6 (2.7-4.9)
**Middle SDI**	**5.1 (2.0-10.1)**	**1.9 (0.7-3.9)**	**6.8 (6.3-7.4)**	**10.4 (9.6-11.2)**	**3.8 (3.1-4.3)**	**4.2 (3.5-5.1)**	**15.3 (12.6-18.6)**	**12.8 (10.4-15.8)**	**3.1 (2.3-3.9)**	**3.7 (3.1-4.4)**
Andhra Pradesh	4.8 (1.9-9.5)	1.6 (0.6-3.4)	6.6 (5.8-7.6)	9.3 (7.9-10.7)	3.3 (2.5-4.1)	3.7 (2.8-4.5)	15.5 (11.9-20.9)	12.9 (9.2-18.5)	2.9 (2.0-4.2)	3.6 (2.5-5.0)
West Bengal	7.5 (3-14.7)	2.9 (1.1-5.8)	6.4 (5.5-7.3)	10.2 (8.8-11.8)	4.4 (3.3-5.5)	5.1 (3.9-6.4)	14.8 (11.1-19.1)	10.7 (8.0-14.5)	3.0 (2.1-3.9)	3.6 (2.8-4.6)
Tripura	4.8 (1.8-9.6)	1.9 (0.6-4.1)	5.7 (4.7-6.8)	10.2 (8.6-11.8)	3.8 (3.1-4.6)	3.6 (2.8-4.4)	15.9 (11.7-21.7)	12.0 (8.3-17.3)	2.7 (1.9-4.0)	3.3 (2.4-4.6)
Arunachal Pradesh	4.9 (1.7-10.5)	2.3 (0.8-5)	3.5 (2.8-5.6)	6.0 (4.8-8.3)	3.6 (2.6-5.0)	3.9 (2.9-5.2)	20.3 (13.7-27.7)	17.3 (11.2-24.9)	3.4 (2.4-4.9)	4.0 (3.0-5.4)
Meghalaya	5.3 (1.9-11.8)	1.3 (0.4-3.0)	3.3 (2.6-5.0)	8.9 (7.3-10.5)	2.3 (1.6-3.6)	2.9 (2.1-4.6)	22.8 (14.1-31.1)	21.9 (11.7-32.2)	3.3 (2.4-4.5)	3.8 (2.6-5.6)
Karnataka	5.4 (2.1-10.5)	2.0 (0.7-4.3)	9.2 (8.0-10.5)	13.9 (12.3-15.4)	3.9 (3-4.8)	4.5 (3.5-5.5)	15.0 (11.7-20.7)	12.6 (9.7-17.0)	3.4 (2.5-4.4)	4.0 (3.2-5.0)
Telangana	3.9 (1.4-7.8)	1.5 (0.5-3.3)	6.3 (5.4-7.3)	8.9 (7.6-10.3)	3.2 (2.5-4.0)	3.6 (2.6-4.5)	14.5 (11.1-19.1)	13.3 (9.3-18.6)	2.8 (1.9-4.0)	3.3 (2.4-4.4)
Gujarat	3.9 (1.5-8.3)	1.5 (0.5-3.2)	7.0 (6.1-8.0)	10.1 (8.8-11.4)	3.3 (2.6-4.0)	3.8 (3.1-4.6)	16.0 (12.2-20.2)	13.7 (10.3-18.1)	3.1 (2.3-4.1)	3.5 (2.8-4.5)
Manipur	5.1 (1.8-10.7)	1.4 (0.4-3.1)	4.8 (4.1-5.6)	9.0 (7.4-10.8)	4.3 (3.3-5.5)	5.7 (4.2-7.4)	19.5 (14.6-27.6)	15.9 (11.4-22.5)	2.8 (2.0-3.7)	3.5 (2.5-4.8)
Jammu and Kashmir[†]	3.6 (1.4-7.6)	0.9 (0.3-1.9)	6.6 (5.7-7.5)	12.3 (10.4-14.4)	5.7 (5-6.5)	3.9 (3.1-4.6)	11.9 (8.9-17.9)	9.1 (6.9-12.3)	2.8 (1.9-3.8)	3.2 (2.3-4.8)
Haryana	3.2 (1.2-6.8)	1.3 (0.5-2.9)	5.6 (4.9-6.5)	8.5 (7.4-9.7)	3.6 (3-4.4)	4.2 (3.5-4.9)	16.4 (13.2-20.2)	15.2 (11.9-19.7)	3.4 (2.5-4.8)	4.3 (3.4-5.6)
**High SDI**	**3.3 (1.3-6.7)**	**1.4 (0.5-3.0)**	**8.7 (8.0-9.5)**	**11.2 (10.1-12.2)**	**3.4 (2.8-3.9)**	**4.2 (3.4-5.1)**	**15.4 (12.8-18.8)**	**13.4 (10.6-17.1)**	**3.2 (2.6-3.9)**	**4.1 (3.4-4.7)**
Uttarakhand	3.2 (1.2-6.9)	0.9 (0.3-2.0)	2.8 (2.2-4.4)	8.7 (7.4-10.3)	3.0 (2.4-3.8)	4.3 (3.4-5.4)	20.4 (15.9-26.9)	15.6 (11.9-20.7)	2.3 (1.7-3.1)	3.8 (2.9-4.9)
Tamil Nadu	3.2 (1.2-6.7)	1.6 (0.5-3.4)	12.6 (11.2-14.1)	14.7 (13.0-16.4)	4.4 (3.5-5.3)	4.9 (3.6-6.3)	14.4 (11.3-19.3)	14.2 (10.4-19.8)	3.4 (2.5-4.5)	4.0 (3.1-4.9)
Mizoram	3.9 (1.4-8.1)	0.8 (0.3-1.7)	4.8 (4.1-5.6)	8.6 (7.5-9.9)	3.0 (2.3-3.7)	3.6 (2.8-4.6)	22.3 (16.0-29.0)	19.5 (13.2-26.5)	3.2 (2.5-4.1)	3.6 (2.6-5.0)
Maharashtra	3.2 (1.2-6.8)	1.6 (0.6-3.5)	6.7 (5.9-7.5)	8.9 (7.7-10.1)	3.0 (2.5-3.5)	4.2 (3.1-5.5)	16.1 (13.2-19.2)	13.6 (10.0-18.4)	3.0 (2.4-3.8)	3.9 (3.0-4.9)
Punjab	3.4 (1.3-7.3)	1.1 (0.4-2.4)	6.8 (5.9-7.8)	12.3 (10.6-14.2)	3.1 (2.6-3.8)	4.3 (3.4-5.3)	13.8 (10.8-18.8)	11.7 (8.9-15.5)	3.4 (2.6-4.4)	4.5 (3.5-5.7)
Sikkim	5.7 (2.2-11.8)	3.6 (1.3-7.5)	4.6 (3.9-5.5)	8.7 (7.3-10.1)	2.5 (1.8-3.3)	3.4 (2.5-4.5)	14.4 (10.1-20.1)	13.7 (9.3-20.0)	3.2 (2.3-4.2)	5.2 (3.5-7.3)
Nagaland	4.0 (1.5-8.4)	1.1 (0.4-2.5)	3.0 (2.3-4.6)	4.8 (3.6-8.1)	3.8 (2.8-4.9)	5.1 (3.8-6.5)	23.8 (18.3-31.3)	19.9 (14.3-26.5)	3.4 (2.5-4.7)	4.1 (2.9-6.1)
Himachal Pradesh	2.2 (0.8-4.6)	0.9 (0.3-2.0)	6.8 (5.6-8.1)	11.3 (9.4-13.2)	2.8 (2.2-3.4)	4.5 (3.4-5.6)	12.1 (9.3-16.3)	10.9 (7.8-15.5)	2.9 (2.1-4.0)	4.1 (3.1-5.5)
UTs other than Delhi	2.7 (1-5.6)	0.6 (0.2-1.3)	5.8 (5.0-6.7)	11.2 (9.6-13)	4.0 (3.3-4.6)	4.9 (3.9-5.9)	16.2 (12.4-21.4)	12.5 (9.1-17.2)	3.7 (2.9-4.6)	4.9 (3.8-6.3)
Kerala	6.1 (2.4-11.7)	2.4 (0.9-5.0)	24.2 (19.7-28.0)	27 (22.1-30.9)	3.3 (2.3-4.3)	3.5 (2.6-4.5)	13.9 (8.9-22.9)	12.2 (7.3-19.3)	3.1 (2.1-4.1)	3.9 (2.5-5.2)
Delhi	2.1 (0.8-4.4)	0.7 (0.2-1.5)	6.0 (5.1-7.2)	8.5 (7.1-10.3)	2.9 (2.3-3.6)	3.3 (2.5-4.3)	14.7 (11.4-19.4)	12.2 (8.9-16.8)	3.6 (2.7-4.7)	4.7 (3.2-6.4)
Goa	2.5 (0.9-5.4)	1.5 (0.5-3.3)	9.8 (8.4-11.5)	12.0 (10.4-13.7)	4.1 (3.3-5.1)	4.8 (3.7-6.1)	12.7 (9.6-17.6)	12.4 (7.7-19.6)	4.7 (3.3-6.1)	6.4 (3.9-9.7)

SDI=Socio-demographic Index, UTs=Union territories.
*The states are listed in increasing order of SDI in 2017.
[†]The state of Jammu and Kashmir was divided into two union territories in August 2019; as we are reporting findings up to 2017, we report findings for the undivided state of Jammu and Kashmir.

15. Correlation between SDI of the states and cause-specific under-5 death rates, 2017

Causes	r	r^2	p-value
Neonatal preterm birth	-0.57	0.32	0.001
Neonatal encephalopathy due to birth asphyxia and trauma	-0.42	0.17	0.020
Neonatal sepsis and other neonatal infections	-0.42	0.17	0.020
Hemolytic disease and neonatal jaundice and other neonatal disorders	-0.50	0.25	0.004
Lower respiratory infections	-0.53	0.28	0.002
Diarrhoeal diseases	-0.72	0.51	<0.0001
Congenital birth defects	-0.18	0.03	0.327
Measles	-0.76	0.58	<0.0001
Injuries	-0.54	0.29	0.002
Other communicable diseases	0.03	0.00	0.859
Other non-communicable diseases	0.12	0.01	0.521

r= Pearson correlation coefficient.

16. Cause-specific under-5 death rates in the states of India, 2000 and 2017

	Under-5 deaths rate per 100,000 (95% uncertainty interval)											
	Neonatal preterm birth			Neonatal encephalopathy due to birth asphyxia and trauma			Neonatal sepsis and other neonatal infections			Haemolytic disease and neonatal jaundice and other neonatal disorders		
States[*]	2000	2017	Percentage change, 2000-2017	2000	2017	Percentage change, 2000-2017	2000	2017	Percentage change, 2000-2017	2000	2017	Percentage change, 2000-2017
**India**	**213 (195-240)**	**125 (106-143)**	**-41.6 (-51.4 to -33.2)**	**126 (111-147)**	**65 (55-89)**	**-48.2 (-58.6 to -36.4)**	**43 (31-51)**	**28 (18-34)**	**-35.0 (-49.6 to -17.5)**	**93 (80-102)**	**57 (47-65)**	**-38.2 (-46.7 to -27.1)**
**Low SDI**	**229 (198-289)**	**143 (115-177)**	**-37.6 (-49.4 to -24.7)**	**142 (121-174)**	**75 (61-110)**	**-47.2 (-60.1 to -32.4)**	**57 (36-70)**	**36 (22-45)**	**-35.3 (-52.2 to -14.9)**	**117 (94-132)**	**74 (57-86)**	**-36.8 (-47.3 to -23.8)**
Bihar	191 (144-261)	108 (77-171)	-43.5 (-59.8 to -23.3)	128 (97-166)	99 (76-131)	-22.5 (-46.3 to 15.3)	48 (27-69)	29 (17-42)	-38.9 (-62.8 to -0.8)	97 (76-121)	73 (54-94)	-23.4 (-46.0 to 7.2)
Madhya Pradesh	284 (233-335)	141 (107-177)	-50.4 (-64.9 to -33.1)	107 (77-179)	41 (25-87)	-61.5 (-77.7 to -42.9)	39 (27-55)	23 (15-33)	-40.7 (-61.2 to -5.4)	139 (107-166)	69 (48-89)	-50.3 (-63.2 to -33.5)
Jharkhand	174 (135-222)	92 (66-121)	-47.1 (-65.3 to -25.1)	112 (84-148)	60 (43-79)	-46.5 (-66.1 to -18.7)	46 (28-66)	25 (15-36)	-45.6 (-66.0 to -13.1)	86 (66-108)	45 (34-60)	-46.3 (-62.4 to -24.1)
Uttar Pradesh	236 (179-343)	169 (123-221)	-28.4 (-49 to 0.5)	168 (129-218)	77 (52-130)	-54.5 (-72.9 to -26.1)	73 (43-102)	48 (27-67)	-34.4 (-57.3 to 0.5)	130 (97-163)	85 (63-108)	-33.8 (-52.1 to -10.4)
Rajasthan	240 (191-295)	151 (114-191)	-37.1 (-57 to -14.2)	98 (67-157)	54 (35-108)	-45.0 (-67.9 to -17.5)	50 (29-68)	35 (19-50)	-29.7 (-54.5 to 4.5)	99 (72-127)	65 (47-85)	-32.7 (-53.7 to -3.8)
Chhattisgarh	265 (214-323)	155 (120-195)	-41.2 (-59.1 to -21.1)	159 (121-206)	75 (53-114)	-52.8 (-70.6 to -27.0)	28 (20-45)	20 (14-30)	-28.6 (-54.2 to 10.8)	125 (95-155)	73 (51-93)	-41.4 (-58.1 to -17)
Odisha	200 (150-293)	108 (78-164)	-45.8 (-63.1 to -21.1)	192 (141-250)	95 (72-125)	-50.4 (-66.3 to -29.4)	55 (37-76)	41 (25-58)	-25.6 (-53.1 to 18.6)	127 (99-159)	69 (52-89)	-44.8 (-61.7 to -20.2)
Assam	221 (182-268)	161 (122-209)	-27 (-48.3 to -0.1)	136 (104-171)	98 (73-130)	-27.5 (-50.9 to 6.6)	62 (39-83)	39 (23-55)	-37.7 (-58.9 to 0.0)	102 (81-124)	68 (51-85)	-32.8 (-52.1 to -9.5)
**Middle SDI**	**213 (182-237)**	**114 (98-129)**	**-46.6 (-54.9 to -38.3)**	**116 (104-132)**	**58 (50-71)**	**-49.9 (-59.3 to -40.1)**	**28 (23-39)**	**17 (14-24)**	**-38.7 (-53 to -20.8)**	**72 (64-82)**	**39 (34-45)**	**-45.5 (-54.5 to -34.7)**
Andhra Pradesh	249 (193-302)	119 (85-165)	-52.4 (-67.3 to -31.1)	146 (115-181)	66 (47-92)	-54.7 (-69.7 to -31.5)	22 (15-41)	13 (8-25)	-42.2 (-66.7 to -3.6)	65 (50-86)	30 (21-43)	-53.0 (-69.2 to -31)
West Bengal	188 (148-228)	109 (86-138)	-42.1 (-57.4 to -22.4)	86 (65-114)	47 (33-70)	-45.5 (-62.6 to -24.4)	26 (19-38)	18 (12-26)	-31.9 (-55.5 to 7.8)	76 (61-91)	43 (33-54)	-43.1 (-58.9 to -21.4)
Tripura	196 (154-246)	157 (119-207)	-19.9 (-42.9 to 11.3)	85 (59-125)	61 (40-105)	-28.6 (-54.8 to 5.2)	51 (33-69)	45 (29-64)	-10.4 (-41.7 to 34.8)	58 (44-76)	48 (36-64)	-16.4 (-43.0 to 20.4)
Arunachal Pradesh	167 (134-209)	76 (59-96)	-54.7 (-67.3 to -37.3)	98 (73-129)	39 (27-55)	-60.4 (-74.8 to -39.1)	56 (35-81)	22 (14-32)	-60.2 (-76.5 to -35.3)	77 (60-97)	31 (24-41)	-59.3 (-71.3 to -43.3)
Meghalaya	132 (101-173)	100 (72-140)	-24.3 (-47.8 to 8.9)	112 (76-150)	89 (64-120)	-20.7 (-47.8 to 23.3)	37 (25-52)	29 (19-43)	-20.5 (-49.5 to 30.1)	47 (36-63)	36 (26-47)	-22.7 (-49.6 to 9.2)
Karnataka	242 (189-285)	119 (92-145)	-50.8 (-61.4 to -37.3)	113 (88-142)	55 (42-72)	-51.3 (-65 to -32.7)	27 (20-42)	14 (10-24)	-47.0 (-64.4 to -23.0)	69 (55-85)	32 (25-41)	-52.6 (-65.1 to -36.7)
Telangana	258 (196-332)	110 (77-149)	-57.6 (-69.7 to -38.5)	139 (104-178)	58 (40-82)	-58.4 (-73.8 to -36.2)	22 (14-41)	12 (7-22)	-47.6 (-70.1 to -10.4)	64 (47-87)	27 (19-39)	-56.7 (-72.4 to -32)
Gujarat	220 (184-260)	131 (106-162)	-40.3 (-54.8 to -23)	144 (116-178)	75 (59-93)	-48.3 (-63.1 to -29.9)	30 (22-42)	16 (11-26)	-46.3 (-66.1 to -17.4)	78 (63-95)	44 (34-56)	-43.4 (-58.7 to -23.5)
Manipur	132 (106-166)	94 (68-132)	-28.9 (-51 to 3.3)	92 (69-125)	58 (43-79)	-36.9 (-59.2 to -3.5)	38 (25-53)	24 (15-33)	-38.5 (-60.9 to -2.8)	67 (52-86)	40 (29-55)	-38.7 (-59.6 to -10.3)
Jammu and Kashmir*	139 (112-168)	90 (68-128)	-34.8 (-55 to -4.7)	80 (63-99)	47 (32-72)	-41.6 (-61.8 to -14.3)	40 (26-53)	31 (19-44)	-21.5 (-44.5 to 19.2)	67 (55-81)	55 (41-71)	-16.0 (-40.6 to 14.0)
Haryana	146 (116-183)	88 (69-111)	-39.5 (-54.1 to -20.5)	100 (78-126)	53 (38-71)	-47.5 (-65.6 to -24.2)	32 (22-42)	19 (13-27)	-39.6 (-58.9 to -9.6)	89 (69-107)	54 (38-68)	-39.2 (-54.7 to -19.1)
**High SDI**	**174 (146-195)**	**83 (71-97)**	**-52.3 (-60.7 to -42.9)**	**95 (84-108)**	**44 (37-54)**	**-53.8 (-62.4 to -43.9)**	**25 (20-33)**	**15 (12-21)**	**-37.1 (-51.6 to -16)**	**54 (47-62)**	**29 (25-34)**	**-45.3 (-54.7 to -33.5)**
Uttarakhand	124 (95-160)	77 (55-104)	-38.1 (-59.5 to -11.1)	79 (55-105)	46 (33-65)	-41.9 (-63.6 to -12.1)	38 (23-52)	31 (15-42)	-19.6 (-47.2 to 26.8)	59 (45-74)	35 (27-46)	-38.6 (-57.5 to -13.8)
Tamil Nadu	177 (142-209)	58 (45-73)	-67.2 (-74.9 to -58.1)	120 (90-148)	35 (26-45)	-71.0 (-79.2 to -61.6)	17 (12-33)	7 (4-14)	-62.0 (-74.8 to -42.3)	36 (27-55)	12 (9-19)	-66.7 (-76.8 to -53.4)
Mizoram	87 (67-110)	96 (70-130)	9.8 (-20.2 to 54.1)	71 (50-94)	70 (52-91)	-2.5 (-36.0 to 52.5)	30 (20-42)	28 (20-40)	-5.0 (-40.6 to 44.9)	44 (34-57)	48 (36-61)	11.9 (-24.2 to 57.9)
Maharashtra	225 (165-269)	107 (80-134)	-52.5 (-64.7 to -38.4)	86 (64-125)	43 (31-66)	-49.7 (-65.1 to -28.6)	29 (21-39)	19 (13-26)	-35.7 (-57.0 to -2.5)	62 (50-76)	34 (26-42)	-45.5 (-60 to -25.2)
Punjab	124 (98-166)	78 (57-111)	-37 (-55.8 to -11.8)	118 (88-148)	64 (49-82)	-45.4 (-62.0 to -22.4)	36 (24-46)	22 (14-30)	-38.3 (-57.3 to -10.5)	76 (62-92)	47 (36-59)	-37.6 (-53.8 to -18.3)
Sikkim	85 (64-110)	44 (32-59)	-48.5 (-65.6 to -24.8)	39 (26-61)	18 (12-31)	-52.6 (-69.4 to -26.9)	31 (18-43)	22 (13-32)	-29.4 (-55.0 to 8.5)	47 (35-63)	24 (17-32)	-47.8 (-65.3 to -24.8)
Nagaland	144 (111-183)	119 (83-176)	-17.3 (-47.9 to 34)	89 (66-119)	69 (42-104)	-22.9 (-58.4 to 35.7)	58 (26-82)	49 (21-77)	-15.0 (-50.1 to 40.1)	60 (46-83)	54 (38-73)	-8.6 (-43.4 to 37.6)
Himachal Pradesh	140 (108-178)	86 (61-114)	-38.8 (-58.5 to -14.5)	134 (93-176)	67 (50-90)	-49.6 (-67.2 to -27.8)	21 (15-30)	16 (11-23)	-25.4 (-51.9 to 22.1)	70 (54-86)	45 (33-58)	-35.1 (-54.9 to -9.7)
UTs other than Delhi	137 (106-171)	133 (101-172)	-3 (-33.2 to 40.3)	86 (65-111)	77 (57-102)	-10.1 (-40.2 to 34.8)	17 (11-26)	21 (14-29)	22.8 (-21.1 to 99.6)	40 (30-54)	41 (31-56)	5.4 (-28.3 to 53.0)
Kerala	81 (62-101)	39 (30-49)	-52.1 (-64.1 to -37)	39 (29-53)	20 (15-27)	-49.4 (-64.4 to -30.7)	11 (7-22)	8 (6-15)	-22.4 (-48.3 to 27.7)	15 (11-27)	8 (6-14)	-47.4 (-64.2 to -24.3)
Delhi	170 (132-219)	103 (74-148)	-39.4 (-59.3 to -12.9)	130 (97-170)	70 (48-99)	-45.9 (-66.8 to -14.9)	24 (16-38)	15 (9-26)	-36.1 (-63.1 to 7.5)	103 (72-131)	62 (41-84)	-38.6 (-58.4 to -12)
Goa	132 (109-160)	62 (42-90)	-53.2 (-69.9 to -28.1)	65 (46-90)	35 (22-53)	-46.9 (-66.6 to -10.7)	19 (11-28)	14 (6-23)	-27.6 (-58.8 to 16.0)	69 (57-83)	40 (27-57)	-41.7 (-61.8 to -16)

	Under-5 deaths rate per 100,000 (95% uncertainty interval)											
	Lower respiratory infections			Diarrhoeal diseases			Measles			Congenital birth defects		
States[*]	2000	2017	Percentage change, 2000-2017	2000	2017	Percentage change, 2000-2017	2000	2017	Percentage change, 2000-2017	2000	2017	Percentage change, 2000-2017
**India**	**335 (305-362)**	**143 (130-158)**	**-57.2 (-61.7 to -51.6)**	**254 (214-297)**	**79 (68-92)**	**-68.7 (-75.5 to -60.7)**	**70 (27-147)**	**13 (5-27)**	**-81.9 (-86.2 to -76.9)**	**75 (69-86)**	**64 (71-56)**	**-15.1 (-32.2 to -3.9)**
**Low SDI**	**457 (412-503)**	**195 (174-219)**	**-57.3 (-62.8 to -50.7)**	**361 (301-429)**	**119 (100-139)**	**-67.1 (-74.8 to -58)**	**86 (31-185)**	**16 (6-35)**	**-81.6 (-86 to -76.2)**	**63 (55-80)**	**71 (60-81)**	**12.1 (-22.9 to 34.7)**
Bihar	462 (387-540)	197 (159-240)	-57.4 (-66.7 to -45.1)	382 (294-482)	163 (125-204)	-57.3 (-69.1 to -40.6)	110 (38-239)	18 (6-42)	-83.9 (-89.4 to -76.2)	52 (41-66)	53 (66-41)	2.2 (-30.1 to 39.4)
Madhya Pradesh	508 (436-581)	173 (143-210)	-66.0 (-72.7 to -57.2)	297 (223-386)	59 (43-80)	-80.2 (-87.1 to -69.7)	82 (29-175)	18 (6-41)	-77.7 (-85.2 to -66.7)	75 (63-92)	57 (70-45)	-24.5 (-46.4 to 0.1)
Jharkhand	325 (274-386)	125 (100-154)	-61.6 (-71.3 to -49.4)	270 (211-336)	86 (61-117)	-68.2 (-79.0 to -53.2)	82 (31-176)	13 (4-31)	-83.7 (-90.3 to -73.0)	48 (37-80)	49 (61-38)	2.7 (-37.7 to 43.2)
Uttar Pradesh	508 (429-595)	211 (174-255)	-58.5 (-67.7 to -47.1)	449 (353-559)	157 (119-202)	-65.1 (-76.3 to -50.6)	85 (30-188)	15 (5-36)	-81.7 (-87.4 to -73.2)	71 (55-101)	94 (117-74)	32.3 (-21.5 to 82.7)
Rajasthan	472 (405-550)	252 (210-301)	-46.7 (-57.1 to -33.1)	257 (190-334)	59 (43-80)	-76.9 (-84.7 to -63.9)	73 (28-154)	17 (6-36)	-77.2 (-83.7 to -67.2)	39 (32-50)	53 (67-40)	34.2 (-11.6 to 88.8)
Chhattisgarh	344 (289-407)	146 (119-177)	-57.6 (-67.0 to -45.3)	201 (156-256)	52 (37-70)	-74.1 (-83.5 to -60.7)	78 (29-165)	14 (5-31)	-82.3 (-89.2 to -72.1)	53 (45-64)	42 (53-33)	-20.9 (-40.7 to 4.8)
Odisha	372 (316-439)	143 (111-182)	-61.6 (-70.8 to -49.6)	309 (233-408)	81 (55-109)	-73.9 (-83.8 to -59.6)	75 (27-168)	7 (2-16)	-90.7 (-94.3 to -85.7)	87 (72-104)	66 (82-51)	-24.3 (-44.5 to 0.4)
Assam	250 (206-297)	179 (142-221)	-28.6 (-45.5 to -7.7)	325 (238-412)	112 (81-150)	-65.4 (-77.9 to -46.5)	69 (24-147)	16 (6-35)	-77.1 (-84.5 to -66.8)	64 (52-78)	102 (134-73)	60.4 (7.8 to 121.1)
**Middle SDI**	**225 (202-247)**	**88 (80-98)**	**-60.8 (-65.2 to -55.7)**	**152 (128-178)**	**32 (27-38)**	**-78.6 (-83.2 to -72.2)**	**68 (27-135)**	**11 (4-22)**	**-84.1 (-88.3 to -79.2)**	**91 (83-100)**	**60 (54-67)**	**-33.9 (-42.3 to -24.7)**
Andhra Pradesh	227 (187-276)	65 (47-88)	-71.6 (-80 to -59.5)	198 (150-253)	45 (31-61)	-77.5 (-85.4 to -65.9)	70 (27-140)	9 (3-18)	-87.7 (-92.3 to -81.0)	96 (81-113)	49 (66-37)	-48.7 (-63.3 to -27.8)
West Bengal	229 (194-265)	104 (84-127)	-54.7 (-64.3 to -42.1)	119 (87-159)	25 (18-34)	-79.2 (-87.1 to -67.1)	94 (37-182)	16 (6-34)	-82.5 (-88.4 to -75.1)	80 (67-93)	59 (72-47)	-26.4 (-44.7 to -5.8)
Tripura	299 (242-361)	206 (161-266)	-31.1 (-47.5 to -7.8)	109 (76-147)	52 (37-72)	-52.3 (-68.8 to -22.1)	62 (23-126)	17 (6-37)	-73.3 (-84.3 to -55.7)	72 (59-89)	91 (119-71)	25.7 (-10 to 70.2)
Arunachal Pradesh	313 (259-376)	79 (63-100)	-74.7 (-80.9 to -67.1)	242 (181-319)	42 (29-59)	-82.7 (-89.2 to -72.4)	78 (27-170)	11 (4-25)	-85.9 (-91.7 to -76.8)	56 (44-89)	29 (41-22)	-48.9 (-63.1 to -30.7)
Meghalaya	292 (234-362)	161 (124-206)	-45 (-58.9 to -25.5)	180 (123-245)	63 (43-89)	-64.9 (-79.1 to -44.1)	71 (26-153)	11 (3-25)	-84.9 (-91.0 to -76.2)	44 (35-70)	75 (94-58)	68.7 (4.6 to 138.7)
Karnataka	171 (143-201)	60 (49-73)	-64.7 (-72.6 to -54.8)	101 (77-128)	22 (16-29)	-78.3 (-85.1 to -68.7)	68 (26-135)	11 (4-23)	-84.3 (-89.5 to -78.0)	115 (99-135)	74 (89-60)	-35.8 (-50.4 to -18.1)
Telangana	210 (172-256)	49 (36-65)	-76.9 (-83.3 to -67.2)	197 (151-255)	32 (22-45)	-83.7 (-89.6 to -74.7)	54 (20-112)	7 (2-16)	-87.2 (-92.6 to -78.5)	86 (71-103)	40 (53-30)	-53.6 (-67.3 to -35.4)
Gujarat	236 (199-275)	95 (77-117)	-59.5 (-68.6 to -47.6)	146 (112-186)	33 (24-43)	-77.3 (-84.4 to -66.3)	54 (20-117)	9 (3-21)	-82.6 (-88.3 to -74.8)	98 (83-115)	65 (80-54)	-33.5 (-48.2 to -15.0)
Manipur	179 (145-212)	89 (69-114)	-50.5 (-63.8 to -33.8)	171 (124-224)	46 (32-62)	-73.3 (-83.3 to -56.7)	59 (21-128)	8 (3-19)	-86.1 (-91.6 to -77.7)	56 (47-67)	54 (72-41)	-3.3 (-29.6 to 31.3)
Jammu and Kashmir*	276 (236-318)	147 (119-181)	-46.8 (-58.2 to -31.7)	117 (86-157)	27 (18-38)	-77.1 (-86.5 to -64)	41 (15-86)	6 (2-12)	-86.5 (-91.4 to -79.2)	75 (63-87)	79 (98-63)	5.8 (-20.8 to 37.8)
Haryana	281 (233-326)	117 (95-141)	-58.4 (-67.8 to -47.1)	244 (184-305)	50 (37-66)	-79.4 (-86.6 to -69.7)	46 (17-101)	8 (3-18)	-81.9 (-88.0 to -74.1)	82 (69-96)	56 (68-45)	-32.0 (-47.5 to -12.4)
**High SDI**	**152 (137-166)**	**57 (51-63)**	**-62.6 (-67 to -57.2)**	**101 (86-117)**	**21 (17-24)**	**-79.6 (-84.4 to -73.8)**	**32 (13-66)**	**6 (2-13)**	**-82 (-87.2 to -75.6)**	**87 (78-95)**	**47 (42-52)**	**-45.3 (-51.8 to -38.1)**
Uttarakhand	225 (190-264)	107 (87-131)	-52.3 (-63 to -38.5)	204 (153-259)	32 (23-43)	-84.2 (-90 to -74.9)	37 (13-78)	5 (2-11)	-86.7 (-92.0 to -78.6)	32 (25-51)	47 (60-38)	46.6 (-6.9 to 100.2)
Tamil Nadu	146 (125-168)	34 (28-41)	-76.9 (-81.7 to -70.6)	99 (78-121)	13 (10-18)	-86.5 (-90.6 to -80.2)	32 (12-69)	4 (2-10)	-86.5 (-91.3 to -79.8)	129 (111-148)	41 (50-34)	-67.8 (-74.1 to -59.4)
Mizoram	208 (173-246)	192 (154-237)	-7.6 (-30.3 to 21.4)	77 (54-106)	24 (16-32)	-69.5 (-81.6 to -49.5)	35 (13-74)	6 (2-14)	-82.4 (-88.6 to -73.4)	43 (36-50)	68 (84-54)	56.9 (20.8 to 104.2)
Maharashtra	161 (139-186)	56 (45-68)	-65.4 (-72.8 to -56.8)	88 (68-107)	19 (14-25)	-78.1 (-85.1 to -68.4)	34 (12-70)	7 (3-17)	-78.2 (-86.3 to -68.1)	70 (60-81)	41 (50-33)	-41.5 (-54.1 to -25.7)
Punjab	184 (154-213)	94 (77-114)	-49.1 (-59.6 to -35.1)	196 (150-244)	47 (35-60)	-76.1 (-84 to -64.9)	40 (14-84)	6 (2-14)	-84.1 (-89.4 to -76.2)	79 (67-94)	74 (91-60)	-7.1 (-28.4 to 21.6)
Sikkim	211 (175-246)	91 (69-116)	-57.1 (-67.5 to -43)	120 (87-157)	25 (17-34)	-79.5 (-86.9 to -67.8)	47 (18-100)	14 (5-30)	-71.3 (-82.2 to -55.6)	39 (31-47)	33 (44-25)	-14.6 (-39.7 to 18.6)
Nagaland	315 (254-383)	241 (178-319)	-23.6 (-44.6 to 6.9)	110 (74-156)	38 (23-57)	-65.7 (-81.2 to -40.5)	55 (20-118)	10 (3-24)	-80.9 (-88.0 to -70.6)	40 (31-63)	46 (81-31)	13.0 (-21.6 to 56.7)
Himachal Pradesh	212 (175-255)	95 (75-120)	-55.1 (-66.8 to -39.7)	153 (106-204)	28 (19-38)	-81.8 (-88.5 to -70.8)	24 (9-51)	5 (2-11)	-80.0 (-87.7 to -68.6)	74 (59-89)	63 (80-48)	-14.7 (-38.2 to 14.2)
UTs other than Delhi	111 (90-135)	90 (72-113)	-19.0 (-38.7 to 7.7)	66 (50-86)	29 (21-38)	-56.9 (-71.3 to -35.1)	20 (7-42)	4 (1-9)	-80.9 (-88.0 to -69.3)	43 (34-53)	74 (92-58)	72.1 (28.0 to 135.9)
Kerala	42 (32-52)	17 (13-21)	-60.1 (-69.9 to -46.5)	6 (4-8)	1 (1-2)	-79.6 (-87.5 to -65.8)	26 (10-49)	5 (2-10)	-81.3 (-88.5 to -70.8)	102 (80-125)	53 (66-41)	-48.1 (-58.8 to -35.7)
Delhi	188 (156-227)	82 (65-103)	-56.4 (-67.0 to -43.3)	154 (115-198)	38 (26-52)	-75.6 (-84.4 to -63.5)	26 (9-54)	4 (1-9)	-84.5 (-91.1 to -74.3)	74 (61-90)	52 (69-40)	-29.2 (-49.3 to -3.5)
Goa	155 (128-184)	71 (50-99)	-54.1 (-67.7 to -34.9)	42 (32-54)	15 (10-23)	-64.4 (-77.7 to -41.2)	21 (8-45)	7 (2-15)	-69.3 (-82.6 to -48.5)	82 (67-100)	53 (74-37)	-35.9 (-55.7 to -6.2)

States[*]	Under-5 deaths rate per 100,000 (95% uncertainty interval)								
	Injuries			Other communicable diseases			Other non-communicable diseases		
	2000	2017	Percentage change, 2000-2017	2000	2017	Percentage change, 2000-2017	2000	2017	Percentage change, 2000-2017
**India**	**60 (50-70)**	**33 (27-40)**	**-45.2 (-54.8 to -28.4)**	**9.2 (7.8-10.9)**	**3.4 (2.9-4.1)**	**-62.6 (-68.9 to -55.3)**	**1.2 (1-1.6)**	**0.7 (0.6-0.8)**	**-44.5 (-52.9 to -33.1)**
**Low SDI**	**75 (61-88)**	**41 (33-51)**	**-44.4 (-56.0 to -25.9)**	**12.3 (10.3-14.6)**	**4.5 (3.7-5.3)**	**-58.0 (-76.3 to -36.5)**	**1.5 (1.1-2.0)**	**0.8 (0.7-1.1)**	**-44.9 (-54.4 to -32.1)**
Bihar	78 (57-102)	39 (28-52)	-49.8 (-67.0 to -22.5)	10.7 (8.1-14.6)	3.8 (2.8-5.1)	-63.9 (-75.7 to -49)	1.5 (1.0-2.0)	0.8 (0.6-1.0)	-46.1 (-62.3 to -22.8)
Madhya Pradesh	80 (60-104)	40 (29-55)	-49.9 (-65.4 to -24.2)	12.3 (9.7-15.5)	4.1 (3.1-5.3)	-66.5 (-76.1 to -55)	1.6 (1.1-2.1)	0.7 (0.6-1.0)	-51.3 (-63.8 to -34.7)
Jharkhand	75 (56-100)	37 (24-56)	-50.1 (-69.4 to -18.2)	9.4 (7.3-12.1)	3 (2-4.3)	-68.0 (-78.9 to -51.5)	1.1 (0.8-1.5)	0.6 (0.4-0.8)	-43.3 (-61.4 to -17.0)
Uttar Pradesh	83 (64-104)	45 (32-62)	-45.6 (-61.5 to -18.6)	15.4 (12.5-18.8)	5.4 (4.2-7)	-64.5 (-73.5 to -52.7)	1.8 (1.3-2.5)	0.9 (0.7-1.3)	-47.4 (-60.5 to -30.2)
Rajasthan	55 (43-69)	31 (24-41)	-43.1 (-59.1 to -18.4)	9 (7-11.5)	3.7 (2.9-4.8)	-58.4 (-70.4 to -42.5)	1.2 (0.8-1.7)	0.7 (0.5-0.9)	-42.2 (-56.9 to -23.7)
Chhattisgarh	62 (48-80)	33 (23-46)	-47.8 (-64.1 to -21.3)	10.1 (7.8-13)	3.5 (2.7-4.8)	-64.5 (-75.6 to -50.3)	1.2 (0.9-1.5)	0.7 (0.5-0.9)	-38.8 (-55.2 to -16.6)
Odisha	70 (55-87)	51 (38-67)	-27.6 (-47.3 to 0.7)	13.3 (9.8-17.5)	5.4 (3.8-7.3)	-58.7 (-72.3 to -40.7)	1.7 (1.2-2.3)	1.1 (0.8-1.5)	-32.9 (-50.4 to -7.8)
Assam	52 (40-66)	51 (38-68)	-1.7 (-32.6 to 41.4)	7.1 (5.4-9.9)	4.7 (3.4-6.4)	-32.5 (-57.2 to -2.2)	1.1 (0.8-1.4)	1.0 (0.7-1.3)	-6.1 (-32.2 to 28.1)
**Middle SDI**	**50 (41-58)**	**25 (20-29)**	**-50.6 (-59.3 to -37.8)**	**6.4 (5.3-7.8)**	**2.3 (1.9-2.9)**	**-63.5 (-71.3 to -55.3)**	**1.0 (0.8-1.3)**	**0.5 (0.4-0.6)**	**-47.4 (-56.6 to -35.3)**
Andhra Pradesh	48 (36-60)	20 (14-27)	-59.1 (-71.8 to -38.4)	7.1 (5.4-9.3)	2.2 (1.5-3.1)	-69.1 (-79.8 to -55.3)	1.1 (0.7-1.5)	0.5 (0.3-0.7)	-54.5 (-69.1 to -33.0)
West Bengal	56 (41-70)	30 (21-38)	-46.8 (-61.4 to -25.9)	5.8 (4.4-7.6)	1.9 (1.4-2.6)	-66.3 (-77.2 to -53.6)	0.9 (0.7-1.2)	0.5 (0.4-0.7)	-43.7 (-58.4 to -24.8)
Tripura	48 (39-59)	32 (24-42)	-33.1 (-50.3 to -10.9)	6.4 (4.7-8.7)	3.4 (2.3-4.8)	-46.0 (-67 to -17.6)	0.9 (0.6-1.3)	0.7 (0.5-1.0)	-13.1 (-38.8 to 17.5)
Arunachal Pradesh	58 (40-82)	19 (13-27)	-68.0 (-78.9 to -45.0)	10.2 (6.8-13.7)	2.6 (1.7-3.7)	-74.0 (-83.5 to -60.2)	1.4 (1.0-2.0)	0.5 (0.4-0.6)	-64.3 (-76.4 to -48.8)
Meghalaya	30 (21-48)	25 (16-40)	-19.0 (-46.9 to 24.4)	9.6 (5.9-13.2)	5.8 (3.1-8.5)	-38.9 (-61.9 to -8.3)	1.1 (0.8-1.5)	0.8 (0.5-1.2)	-26.0 (-49.3 to 5.2)
Karnataka	48 (37-61)	24 (19-30)	-50.5 (-63.4 to -30.5)	5.9 (4.6-7.9)	2.1 (1.6-2.8)	-63.9 (-75.1 to -50.1)	1.1 (0.8-1.4)	0.5 (0.4-0.7)	-48.5 (-61.1 to -31.9)
Telangana	44 (33-56)	16 (11-22)	-63.6 (-76.0 to -44.1)	6.2 (4.8-8.3)	1.9 (1.3-2.6)	-69.3 (-79.8 to -56.2)	1.0 (0.6-1.4)	0.4 (0.3-0.5)	-59.9 (-74.1 to -39.1)
Gujarat	46 (37-57)	25 (19-31)	-46.9 (-61.8 to -25.7)	7 (5.4-8.9)	2.8 (2.1-3.7)	-59.8 (-71 to -46.1)	1.1 (0.8-1.4)	0.6 (0.4-0.7)	-46.7 (-60.3 to -30.6)
Manipur	51 (38-67)	34 (24-48)	-32.4 (-55.6 to 3.4)	7.2 (5.4-10)	3 (2.2-4.3)	-57.4 (-72.4 to -37.8)	0.8 (0.6-1.1)	0.5 (0.4-0.7)	-34.1 (-56 to -6.2)
Jammu and Kashmir*	65 (57-74)	25 (19-31)	-62.0 (-71.1 to -50.0)	4.2 (3.2-6.1)	1.8 (1.4-2.5)	-55.7 (-72.5 to -38)	0.8 (0.5-1.1)	0.5 (0.4-0.8)	-34.6 (-52.3 to -13.4)
Haryana	53 (43-64)	27 (22-34)	-48.4 (-60.3 to -28.9)	7.4 (6-9.3)	3.1 (2.4-4)	-57.8 (-70.1 to -43.3)	1.2 (0.9-1.7)	0.7 (0.5-0.9)	-42.8 (-57.7 to -25.1)
**High SDI**	**34 (28-38)**	**18 (14-22)**	**-46.4 (-56.4 to -32.6)**	**4.8 (4-5.8)**	**1.8 (1.4-2.3)**	**-62.6 (-70.5 to -53.5)**	**0.8 (0.6-1)**	**0.4 (0.4-0.5)**	**-44.6 (-54.9 to -31.0)**
Uttarakhand	35 (27-46)	23 (18-31)	-33.4 (-52.9 to -2.6)	7.3 (5.7-9.4)	2.7 (2-3.5)	-63.3 (-74.2 to -48.9)	0.7 (0.5-0.9)	0.5 (0.4-0.7)	-23.0 (-46.3 to 6.9)
Tamil Nadu	45 (35-55)	14 (10-18)	-69.6 (-78.3 to -56.2)	4.6 (3.6-6.2)	1.3 (0.9-1.7)	-72.4 (-81.1 to -61.9)	0.9 (0.6-1.1)	0.3 (0.2-0.3)	-67.2 (-76.4 to -55.2)
Mizoram	27 (21-34)	29 (21-39)	7.1 (-24.4 to 55.6)	6.2 (4.5-8.2)	4.8 (3.3-6.6)	-21.6 (-46.1 to 8.2)	0.7 (0.6-0.9)	0.7 (0.5-1.0)	-0.1 (-32.4 to 41.9)
Maharashtra	31 (25-38)	19 (14-26)	-38.7 (-55.1 to -15.0)	5.3 (4.3-6.5)	2 (1.4-2.6)	-62.7 (-72.6 to -49.8)	0.8 (0.6-1)	0.4 (0.3-0.6)	-43.2 (-58.3 to -23.9)
Punjab	37 (29-44)	26 (20-33)	-30.0 (-47.5 to -4.0)	5 (3.9-6.7)	2.2 (1.7-2.9)	-55.4 (-69.8 to -37.8)	1.0 (0.7-1.3)	0.7 (0.5-0.9)	-30.7 (-46.3 to -7.9)
Sikkim	21 (15-28)	13 (9-19)	-37.6 (-58.5 to -5.1)	3.7 (2.6-5.4)	1.6 (1.1-2.4)	-55.7 (-72.0 to -36.8)	0.7 (0.5-0.9)	0.5 (0.3-0.7)	-24 (-48.5 to 15.6)
Nagaland	51 (36-70)	49 (33-68)	-5.3 (-36.4 to 41.6)	10.1 (7.8-13.3)	6 (4.3-8)	-39.7 (-59.7 to -13.8)	1.2 (0.9-1.6)	1.0 (0.7-1.5)	-13.2 (-42 to 24.7)
Himachal Pradesh	30 (24-38)	25 (18-34)	-17.1 (-38.4 to 17.4)	4.1 (3.2-5.7)	1.9 (1.4-2.7)	-53.1 (-68.6 to -32.1)	0.8 (0.6-1.1)	0.6 (0.4-0.8)	-26.1 (-47.5 to 4.2)
UTs other than Delhi	29 (23-36)	32 (24-41)	9.2 (-16.3 to 44.9)	3.7 (2.9-4.8)	2.6 (1.9-3.5)	-30.2 (-49.3 to -5.3)	0.7 (0.5-0.9)	0.8 (0.6-1.0)	19.8 (-11.0 to 62.4)
Kerala	14 (9-19)	7 (5-9)	-51.8 (-65.8 to -30.1)	1.8 (1.2-2.9)	0.8 (0.4-1.2)	-63.5 (-70.1 to -55.5)	0.3 (0.2-0.4)	0.2 (0.1-0.3)	-40.0 (-59.4 to -11.4)
Delhi	36 (28-46)	20 (14-28)	-44.4 (-62.1 to -17.6)	5.7 (4.4-7.4)	2.3 (1.7-3.2)	-58.2 (-70.8 to -40.9)	1.1 (0.8-1.5)	0.7 (0.5-1.0)	-34.3 (-56.4 to -3.1)
Goa	34 (26-45)	21 (14-31)	-38.3 (-61.1 to -1.2)	3.3 (2.5-4.5)	1.7 (1.1-2.7)	-48.2 (-67.8 to -18.5)	1.0 (0.7-1.3)	0.7 (0.4-1.1)	-27.4 (-54 to 14.0)

SDI=Socio-demographic Index. UTs=Union territories.
*The states are listed in increasing order of SDI in 2017.
[†]The state of Jammu and Kashmir was divided into two union territories in August 2019; as we are reporting findings up to 2017, we report findings for the undivided state of Jammu and Kashmir.

17. Causes of neonatal death in the states of India, 2000 and 2017

	Percentage of neonatal deaths (95% uncertainty interval)									
	Neonatal preterm birth		Neonatal encephalopathy due to birth asphyxia and trauma		Neonatal sepsis and other neonatal infections		Hemolytic disease and neonatal jaundice		Other neonatal disorders	
**States***	**2000**	**2017**	**2000**	**2017**	**2000**	**2017**	**2000**	**2017**	**2000**	**2017**
**India**	**26.8 (24.6-30.4)**	**27.7 (24.1-31.7)**	**15.8 (14.1-18.5)**	**14.5 (12.4-19.7)**	**5.3 (3.8-6.5)**	**6.1 (4.0-7.5)**	**3.6 (3.2-4.2)**	**3.2 (2.7-3.8)**	**19.4 (16.6-21.6)**	**22.0 (17.4-25.2)**
**Low SDI**	**23.8 (20.9-30.2)**	**25.8 (21.1-31.5)**	**14.8 (12.7-18.0)**	**13.5 (11.1-19.5)**	**5.9 (3.7-7.3)**	**6.5 (3.9-8.1)**	**3.4 (2.9-4.1)**	**3.1 (2.5-3.7)**	**20.8 (16.1-23.9)**	**23.4 (17.2-27.9)**
Bihar	22.1 (17.2-30.0)	19.9 (14.9-30.4)	14.9 (11.4-18.7)	18.4 (15.0-22.8)	5.5 (3.1-7.9)	5.4 (3.2-7.8)	3.4 (2.4-4.7)	3.5 (2.4-4.9)	18.7 (14.2-23.8)	23.5 (16.8-30.8)
Madhya Pradesh	28.7 (24.6-33.4)	30.9 (24.9-36.1)	10.9 (7.9-17.8)	9.0 (5.6-19.0)	3.9 (2.7-5.6)	5.1 (3.4-7.3)	3.9 (2.9-5.0)	2.9 (2.0-3.9)	24.1 (17.4-29.5)	27.0 (18.0-35.6)
Jharkhand	24.3 (19.8-30.0)	25.2 (19.0-31.6)	15.5 (12.1-19.6)	16.3 (12.3-20.4)	6.3 (3.8-9.2)	6.7 (4.1-9.6)	3.0 (2.0-4.2)	2.5 (1.7-3.6)	20.8 (15.4-26.5)	22.1 (16.0-29.8)
Uttar Pradesh	21.7 (17.1-30.5)	26.2 (20.5-32.4)	15.6 (12.2-19.4)	11.8 (8.4-19.7)	6.7 (3.9-9.3)	7.3 (4.1-10.3)	3.5 (2.6-4.6)	3.2 (2.3-4.4)	20.3 (14.4-26.1)	22.9 (16.3-29.6)
Rajasthan	28.9 (24.6-33.9)	29.1 (23.2-34.1)	11.8 (8.3-18.7)	10.4 (7.2-20.2)	6.0 (3.4-8.2)	6.7 (3.6-9.5)	1.9 (1.4-2.5)	1.4 (0.9-2.0)	21.6 (15.5-28.3)	23.7 (16.6-31.1)
Chhattisgarh	28.3 (23.7-33.5)	30.6 (24.6-36.4)	17.0 (13.1-21.4)	14.7 (10.9-22.1)	3.0 (2.1-4.7)	4.0 (2.7-5.7)	3.3 (2.4-4.4)	3.0 (2.1-4.2)	23.3 (16.7-29.3)	25.3 (16.6-33.2)
Odisha	20.4 (15.8-29.1)	21.7 (16.7-31.6)	19.7 (14.8-24.9)	19.0 (15.3-23.3)	5.6 (3.7-7.7)	8.1 (5.0-11.5)	4.6 (3.3-6.2)	4.4 (3.0-6.1)	21.0 (15.5-27.5)	22.9 (16.0-30.5)
Assam	26.4 (22.5-30.8)	26.9 (22.0-32.2)	16.2 (12.8-20.0)	16.4 (12.8-20.1)	7.4 (4.6-9.9)	6.4 (3.8-9.2)	3.7 (2.8-5.1)	3.5 (2.5-4.8)	20.5 (15.7-25.7)	18.9 (13.6-24.6)
**Middle SDI**	**32.3 (27.9-35.4)**	**32.8 (29.0-36.1)**	**17.7 (15.8-20.0)**	**16.8 (14.8-20.5)**	**4.1 (3.4-5.9)**	**4.8 (3.8-6.8)**	**4.5 (3.9-5.4)**	**3.8 (3.2-4.5)**	**17.2 (15.0-19.7)**	**18.5 (16.1-21.1)**
Andhra Pradesh	35.3 (28.6-40.5)	36.9 (29.6-42.2)	20.8 (17.0-25.3)	20.7 (16.8-24.6)	3.1 (2.1-5.7)	4.0 (2.5-7.7)	6.4 (4.7-8.6)	5.2 (3.4-7.5)	11.6 (8.4-16.4)	13.3 (9.0-20.1)
West Bengal	30.5 (25.1-36.0)	30.7 (26.0-36.1)	13.9 (10.7-18.0)	13.1 (9.9-19.1)	4.2 (3.0-6.1)	5.0 (3.4-7.1)	5.6 (4.2-7.2)	5.2 (3.6-7.3)	18.8 (14.6-23.2)	18.6 (14.2-24.0)
Tripura	30.6 (25.8-35.6)	31.0 (26.1-36.8)	13.3 (9.7-18.7)	11.9 (8.6-19.6)	7.8 (5.2-10.7)	8.8 (5.5-12.6)	4.0 (2.9-5.6)	3.2 (2.1-4.8)	13.9 (10.2-18.9)	15.5 (11.4-20.9)
Arunachal Pradesh	25.5 (21.4-29.9)	28.9 (24.4-33.6)	14.9 (11.5-18.8)	14.8 (10.5-19.2)	8.4 (5.3-12.3)	8.4 (5.3-12.4)	5.5 (4.0-7.4)	4.7 (3.2-6.7)	17.7 (13.2-23.0)	18.7 (13.8-24.9)
Meghalaya	26.0 (21.5-31.5)	25.9 (19.7-34.3)	22.0 (15.9-27.8)	23.0 (17.7-28.9)	7.2 (4.8-10.3)	7.5 (4.8-11.1)	3.7 (2.6-5.2)	3.4 (2.1-5.0)	14.7 (10.7-21.0)	15.0 (10.4-20.4)
Karnataka	37.3 (30.0-43.0)	37.4 (30.1-42.4)	17.4 (14.0-21.8)	17.3 (13.8-21.7)	4.1 (3.0-6.5)	4.5 (3.2-7.2)	2.0 (1.5-2.7)	1.5 (1.1-2.0)	19.0 (14.7-23.8)	18.4 (14.3-23.7)
Telangana	36.4 (28.8-42.5)	37.7 (30.0-43.0)	19.7 (15.9-24.0)	19.9 (15.8-24.0)	3.1 (2.0-5.8)	4.0 (2.4-7.7)	6.3 (4.5-8.7)	5.0 (3.3-7.7)	11.4 (7.7-17.0)	13.3 (8.9-19.9)
Gujarat	30.7 (27.0-34.7)	33.0 (28.1-38.0)	20.2 (16.6-24.3)	18.7 (15.3-22.4)	4.1 (2.9-5.8)	4.0 (2.7-6.5)	2.6 (1.9-3.3)	2.2 (1.6-2.9)	19.2 (14.8-23.7)	19.7 (15.1-25.1)
Manipur	23.7 (19.8-28.1)	27.2 (22.1-34.7)	16.6 (12.9-21.4)	16.8 (12.9-21.2)	6.8 (4.4-9.4)	6.8 (4.4-9.4)	5.6 (4.0-8.0)	6.2 (4.0-9.1)	18.1 (13.3-24.1)	17.0 (11.3-23.8)
Jammu and Kashmir*	24.6 (20.0-29.0)	23.1 (18.3-30.6)	14.3 (11.4-17.6)	11.9 (8.7-17.8)	7.0 (4.5-9.4)	8 (4.8-11.2)	6.9 (5.0-9.2)	7.9 (5.2-10.9)	16.2 (12.5-20.6)	20.0 (14.0-26.3)
Haryana	23.9 (20.1-29.2)	26.0 (21.4-31.4)	16.6 (13.2-20.2)	15.6 (11.9-20.3)	5.2 (3.5-7.0)	5.6 (3.9-7.7)	4.1 (3.1-5.5)	3.3 (2.5-4.4)	24.8 (18.4-30.7)	27.9 (19.0-35.9)
**High SDI**	**32.4 (27.8-35.8)**	**31.4 (28.0-36.0)**	**17.7 (15.8-20.1)**	**16.5 (14.4-20.4)**	**4.5 (3.7-6.2)**	**5.7 (4.5-7.8)**	**3.4 (2.9-3.9)**	**3.2 (2.6-3.8)**	**16.4 (14.2-19.3)**	**18.7 (16.0-21.9)**
Uttarakhand	22.9 (18.1-28.4)	24.6 (19.1-31.6)	14.6 (10.4-19.1)	14.7 (11.2-20.3)	7.0 (4.2-9.6)	9.7 (4.8-13.4)	3.5 (2.5-4.9)	3.3 (2.4-4.6)	18.1 (13.6-23.6)	19.2 (14.6-25.2)
Tamil Nadu	34.5 (28.7-39.9)	34.8 (27.1-40.0)	23.5 (18.3-28.5)	20.9 (16.2-25.3)	3.3 (2.2-6.5)	3.8 (2.6-8.1)	2.9 (2.1-3.7)	2.1 (1.5-3.0)	11.1 (7.7-18.5)	11.9 (8.2-20.6)
Mizoram	21.3 (17.3-25.7)	23.6 (18.9-30.0)	17.3 (12.6-21.9)	17.1 (14.0-20.3)	7.2 (4.9-10.2)	6.9 (4.8-9.9)	2.1 (1.2-3.4)	2.4 (1.6-3.4)	19.1 (14.3-25.4)	21.1 (15.5-27.2)
Maharashtra	37.0 (28.2-42.8)	35.8 (28.9-41.7)	14.3 (10.9-20.5)	14.5 (11.2-21.5)	4.7 (3.4-6.5)	6.1 (4.3-8.7)	2.8 (2.2-3.6)	2.6 (1.9-3.5)	17.6 (13.6-22.1)	19.7 (15.3-24.9)
Punjab	22.0 (18.0-29.0)	22.9 (18.0-31.4)	21.0 (16.1-25.7)	18.8 (15.4-22.8)	6.3 (4.3-8.1)	6.4 (4.1-8.6)	5.6 (4.2-7.4)	5.0 (3.6-6.8)	20.8 (16.6-26.2)	22.0 (16.0-28.3)
Sikkim	21.2 (17.4-25.5)	20.6 (16.8-25.2)	9.8 (7.1-14.9)	8.7 (6.1-14.7)	7.6 (4.5-10.7)	10.1 (6-14.9)	4.0 (2.8-5.5)	3.1 (2.1-4.4)	19.6 (13.9-26.8)	19.6 (13.9-26.7)
Nagaland	24.7 (20.3-29.4)	25.2 (19.2-33.7)	15.4 (11.8-19.4)	14.6 (9.4-19.9)	9.8 (4.4-14)	10.3 (4.4-16.4)	8.8 (6.4-11.8)	10.7 (6.5-16.1)	11.3 (7.9-19.1)	11.6 (7.7-16.5)
Himachal Pradesh	24.0 (19.3-29.5)	25.2 (19.1-32.2)	23.1 (16.6-29.5)	19.9 (15.9-24.4)	3.6 (2.4-5.2)	4.5 (3.0-6.8)	4.8 (3.4-6.6)	4.5 (3.0-6.4)	18.7 (13.6-24.1)	21.4 (15.1-28.5)
UTs other than Delhi	32.6 (27.7-37.4)	31.8 (26.6-37.5)	20.4 (16.7-24.6)	18.4 (14.6-22.4)	4.0 (2.7-6.2)	4.9 (3.3-6.9)	6.4 (4.6-8.6)	6.7 (4.6-9.5)	12.5 (8.7-17.9)	12.9 (9.2-18.3)
Kerala	28.8 (23.3-33.4)	27.3 (22.2-32.7)	14.1 (10.6-18.4)	14.1 (10.8-18.1)	3.8 (2.5-7.7)	5.8 (4-10.3)	2.0 (1.4-2.8)	1.7 (1.2-2.3)	8.9 (5.9-18.0)	9.5 (6.6-18.5)
Delhi	25.1 (20.5-32.2)	25.5 (19.7-34.7)	19.2 (15.3-24.1)	17.4 (12.7-22.4)	3.5 (2.3-5.7)	3.7 (2.3-6.5)	4.4 (3.1-6.0)	3.8 (2.4-5.6)	25.9 (16.6-33.7)	26.9 (17.3-36.6)
Goa	26.4 (22.6-30.2)	22.9 (18.5-27.6)	13.0 (9.5-17.8)	12.7 (9.5-16.3)	3.8 (2.3-5.5)	5.0 (2.2-8.4)	9.8 (7.3-12.8)	6.4 (4.2-9.5)	17.6 (13.4-22.3)	22.8 (15.3-33.4)

	Percentage of neonatal deaths (95% uncertainty interval)									
	Lower respiratory infections		Diarrhoeal diseases		Tetanus		Congenital birth defects		Other causes	
**States***	**2000**	**2017**	**2000**	**2017**	**2000**	**2017**	**2000**	**2017**	**2000**	**2017**
**India**	**12.8 (11.6-14.0)**	**11.0 (9.7-12.5)**	**3.7 (3.2-4.3)**	**2.7 (2.2-3.2)**	**3.61 (2.67-4.75)**	**0.74 (0.41-1.40)**	**5.3 (4.8-6.1)**	**8.6 (7.5-9.6)**	**3.6 (2.2-5.7)**	**3.5 (2.2-5.4)**
**Low SDI**	**14.8 (13.1-16.5)**	**12.3 (10.6-14.1)**	**4.2 (3.5-5.0)**	**3.2 (2.6-3.9)**	**4.92 (3.51-6.65)**	**0.93 (0.48-1.85)**	**4.0 (3.5-5.0)**	**8.0 (6.7-9.4)**	**3.3 (2.1-5.1)**	**3.2 (2.1-4.8)**
Bihar	17.1 (13.5-20.7)	13.7 (11.2-16.5)	5.5 (3.8-7.4)	4.9 (3.7-6.3)	5.60 (3.09-9.02)	0.60 (0.23-1.28)	3.7 (2.9-4.8)	6.4 (5.1-7.8)	3.5 (1.9-5.7)	3.8 (2.0-5.7)
Madhya Pradesh	15.8 (13.5-18.7)	12.1 (10.2-14.5)	2.8 (2.1-3.8)	1.7 (1.2-2.3)	1.94 (1.04-3.23)	0.25 (0.10-0.57)	4.6 (3.8-5.8)	7.8 (6.3-9.5)	3.4 (2.1-5.5)	3.3 (2.1-5.2)
Jharkhand	13.4 (10.9-16.3)	12.0 (9.7-15.1)	4.6 (3.2-6.1)	3.6 (2.6-4.9)	5.12 (2.68-8.44)	0.72 (0.35-1.41)	3.5 (2.6-5.7)	7.3 (5.9-9.1)	3.6 (2.0-6.0)	3.6 (2.1-5.8)
Uttar Pradesh	13.7 (11.2-16.5)	11.0 (8.9-13.5)	4.4 (3.3-5.9)	3.5 (2.5-4.6)	6.99 (4.27-10.55)	1.56 (0.66-3.53)	4.0 (3.1-5.6)	9.3 (7.2-11.9)	3.2 (1.9-5.2)	3.1 (1.9-4.7)
Rajasthan	18.3 (15.5-21.7)	17.7 (14.5-21.4)	3.2 (2.2-4.5)	1.7 (1.2-2.3)	2.81 (1.71-4.24)	0.66 (0.23-1.30)	2.7 (2.2-3.4)	6.0 (4.7-7.5)	3.0 (1.7-4.8)	2.6 (1.6-4.2)
Chhattisgarh	13.7 (11.3-16.4)	11.8 (9.4-14.8)	2.8 (2.1-3.8)	1.7 (1.2-2.3)	1.72 (0.93-3.08)	0.37 (0.19-0.66)	3.4 (2.8-4.1)	5.2 (4.1-6.6)	3.5 (2.1-5.7)	3.3 (2.0-5.4)
Odisha	12.8 (10.7-15.2)	9.1 (7.5-11.1)	4.2 (3.0-5.6)	2.8 (2.0-3.9)	2.38 (1.22-4.15)	0.36 (0.14-0.90)	5.6 (4.7-6.7)	7.9 (6.3-9.7)	3.7 (2.4-5.6)	3.7 (2.4-5.8)
Assam	10.4 (8.5-12.7)	10.2 (8.3-12.5)	4.8 (3.5-6.4)	3.2 (2.2-4.4)	1.95 (1.10-3.14)	0.32 (0.12-0.73)	5.5 (4.5-6.6)	11.6 (8.6-14.8)	3.1 (1.7-5.3)	2.7 (1.6-4.5)
**Middle SDI**	**9.3 (8.5-10.2)**	**8.2 (7.4-9.2)**	**3.0 (2.5-3.5)**	**1.6 (1.3-1.9)**	**1.12 (0.88-1.41)**	**0.23 (0.14-0.40)**	**6.9 (6.4-7.5)**	**9.3 (8.4-10.3)**	**3.9 (2.4-6.5)**	**4.0 (2.4-6.6)**
Andhra Pradesh	7.9 (6.4-9.6)	5.4 (4.5-6.4)	4.2 (3.1-5.7)	2.5 (1.9-3.4)	1.15 (0.64-1.99)	0.18 (0.08-0.37)	6.1 (5.3-7.2)	7.7 (6.5-9.2)	3.4 (2.1-5.5)	4.2 (2.3-7.6)
West Bengal	11.9 (10-14.1)	11.7 (9.8-14.1)	2.6 (1.9-3.7)	1.5 (1.0-2.1)	1.52 (0.89-2.41)	0.33 (0.15-0.71)	7.0 (6.0-8.2)	9.8 (8.0-12.0)	4.1 (2.5-6.8)	4.1 (2.5-6.9)
Tripura	15.8 (13.2-18.7)	14.0 (11.1-17.1)	2.2 (1.5-3.0)	1.9 (1.4-2.6)	1.87 (0.97-3.36)	0.16 (0.06-0.40)	6.2 (5.1-7.8)	9.8 (7.9-12.2)	4.3 (2.6-7.0)	3.6 (2.1-5.8)
Arunachal Pradesh	13.6 (11.1-16.7)	11.0 (8.5-13.6)	5.1 (3.7-7.1)	2.6 (1.9-3.7)	0.07 (0.03-0.16)	0.02 (0.01-0.04)	4.2 (3.3-7.0)	6.3 (4.9-9.3)	4.8 (2.8-7.8)	4.6 (2.5-7.7)
Meghalaya	12.8 (10.5-15.1)	10.1 (8.0-12.5)	4.2 (3.1-5.5)	2.1 (1.5-2.9)	0.89 (0.31-1.94)	0.17 (0.06-0.36)	4.0 (3.1-6.1)	9.0 (7.1-11.3)	4.6 (2.5-7.8)	3.8 (2.1-6.7)
Karnataka	5.7 (4.7-6.9)	4.8 (4.0-5.7)	1.9 (1.4-2.6)	1.1 (0.8-1.5)	0.53 (0.28-0.92)	0.04 (0.01-0.09)	8.2 (7.1-9.7)	11.0 (9.4-12.9)	3.8 (2.2-6.4)	3.9 (2.3-6.7)
Telangana	7.9 (6.5-9.7)	5.3 (4.4-6.4)	4.2 (3.0-5.5)	2.3 (1.7-3.2)	1.02 (0.53-1.87)	0.15 (0.07-0.31)	5.9 (4.9-7.1)	7.7 (6.4-9.1)	4.1 (2.3-7.1)	4.7 (2.4-8.1)
Gujarat	9.0 (7.5-10.9)	7.8 (6.5-9.4)	2.1 (1.6-2.8)	1.4 (1.0-1.7)	0.99 (0.59-1.68)	0.25 (0.10-0.49)	7.4 (6.4-8.8)	9.3 (7.9-11.2)	3.7 (2.2-6.3)	3.7 (2.1-6.2)
Manipur	11.0 (9.2-13.0)	9.8 (7.4-12.7)	4.4 (3.2-5.8)	2.0 (1.4-2.8)	3.15 (1.60-5.51)	0.86 (0.33-1.82)	5.7 (4.7-6.9)	9.4 (7.1-12.3)	4.8 (2.7-8.4)	4.1 (2.2-7.1)
Jammu and Kashmir†	16.2 (13.7-18.7)	13.2 (10.5-16)	2.5 (1.8-3.5)	1.2 (0.8-1.7)	0.94 (0.45-1.74)	0.15 (0.06-0.30)	7.2 (6.2-8.4)	11.1 (8.8-13.7)	4.1 (2.7-6.6)	3.3 (2.0-5.3)
Haryana	10.0 (8.5-11.9)	8 (6.7-9.4)	4.0 (3.0-5.1)	1.6 (1.2-2.0)	1.46 (0.85-2.36)	0.36 (0.17-0.78)	6.0 (5.1-7.0)	7.5 (6.4-8.9)	4.0 (2.4-6.6)	4.3 (2.5-7.1)
**High SDI**	**8.6 (7.8-9.5)**	**7.4 (6.5-8.4)**	**2.5 (2.1-2.9)**	**1.3 (1.0-1.5)**	**1.31 (1.03-1.67)**	**0.35 (0.22-0.67)**	**8.9 (8.1-9.8)**	**10.7 (9.4-12.0)**	**4.3 (2.5-7.3)**	**4.7 (2.7-8.0)**
Uttarakhand	14.9 (12.2-17.7)	12.1 (9.2-15.1)	4.1 (2.9-5.8)	1.4 (1.0-1.9)	6.93 (4.31-10.93)	1.61 (0.86-3.36)	3.5 (2.7-5.6)	8.6 (6.8-10.8)	4.6 (2.4-7.9)	4.8 (2.5-8.5)
Tamil Nadu	6.5 (5.4-7.6)	5.7 (4.8-6.9)	2.4 (1.8-3.1)	1.3 (1.0-1.8)	0.17 (0.09-0.30)	0.00 (0.00-0.00)	11.1 (9.7-12.8)	13.2 (11.4-15.3)	4.6 (2.5-7.9)	6.1 (3.3-10.7)
Mizoram	18.1 (15.4-21.1)	15.3 (12.2-18.6)	2.7 (1.9-3.8)	0.9 (0.6-1.2)	1.37 (0.57-2.70)	0.11 (0.05-0.24)	5.9 (5.1-6.9)	8.8 (7.4-10.6)	5.0 (2.8-8.5)	3.9 (2.3-6.4)
Maharashtra	9.3 (7.8-10.9)	7.1 (6.0-8.5)	2.2 (1.7-2.8)	1.2 (0.9-1.5)	1.27 (0.81-1.90)	0.35 (0.18-0.68)	6.9 (6.0-8.0)	8.4 (7.0-10.0)	3.9 (2.3-6.7)	4.3 (2.4-7.4)
Punjab	7.9 (6.5-9.5)	7.6 (6.2-9.2)	4.0 (2.9-5.3)	1.6 (1.2-2.1)	1.42 (0.76-2.39)	0.35 (0.14-0.83)	7.2 (6.0-8.5)	11.5 (9.4-14.2)	3.8 (2.2-6.4)	3.9 (2.1-6.7)
Sikkim	20.9 (17.2-24.8)	19.2 (15.8-22.6)	5.4 (3.8-7.5)	2.6 (1.8-3.5)	0.11 (0.04-0.24)	0.03 (0.01-0.09)	5.7 (4.6-7.1)	9.6 (7.8-11.6)	5.8 (2.9-10)	6.5 (3.3-11.9)
Nagaland	15.9 (13.2-19.2)	16.1 (11.8-20.8)	2.0 (1.3-2.9)	1.2 (0.7-1.7)	4.29 (2.15-7.40)	0.92 (0.25-2.38)	3.5 (2.7-5.3)	4.9 (3.3-8.7)	4.2 (2.5-6.8)	4.4 (2.4-7.9)
Himachal Pradesh	10.2 (8.2-12.4)	8.6 (7.0-10.8)	3.0 (2.0-4.1)	1.3 (0.9-1.8)	1.96 (0.95-3.59)	0.51 (0.13-1.61)	6.7 (5.5-8.1)	9.9 (8.0-12.4)	3.9 (2.2-6.9)	4.2 (2.4-7.4)
UTs other than Delhi	9.1 (7.7-10.6)	8.3 (6.6-10.1)	2.7 (2.1-3.5)	1.2 (0.9-1.6)	0.20 (0.09-0.41)	0.03 (0.01-0.08)	6.2 (5.3-7.5)	10.8 (8.7-13.2)	5.9 (3.3-9.9)	4.9 (2.6-8.2)
Kerala	7.3 (5.7-9.0)	6.0 (4.6-7.4)	0.5 (0.4-0.8)	0.3 (0.2-0.4)	1.04 (0.57-1.79)	0.29 (0.10-0.70)	27.1 (20.9-31.7)	27.6 (21.4-32.4)	6.5 (3.1-12)	7.5 (3.6-13.6)
Delhi	7.3 (5.8-9.1)	7.7 (6.0-10.0)	2.8 (1.9-3.9)	1.8 (1.2-2.5)	1.42 (0.65-2.81)	0.29 (0.12-0.66)	6.5 (5.3-8.2)	8.6 (6.6-11.1)	3.9 (2.3-6.9)	4.1 (2.2-7.2)
Goa	12.4 (10.3-14.9)	11 (8.9-13.1)	1.5 (1.1-2.0)	1.1 (0.8-1.5)	0.96 (0.47-1.85)	0.47 (0.11-2.05)	10.4 (8.4-12.8)	12.0 (9.9-14.4)	4.1 (2.2-7.2)	5.6 (2.8-10.4)

SDI=Socio-demographic Index. UTs=Union territories.
*The states are listed in increasing order of SDI in 2017.
†The state of Jammu and Kashmir was divided into two union territories in August 2019; as we are reporting findings up to 2017, we report findings for the undivided state of Jammu and Kashmir.

18. Proportion of neonatal deaths in early and late neonatal periods in the states of India, 2017

States*	Percentage of neonatal deaths	
	Early neonatal	Late neonatal
**India**	**79.5**	**20.5**
Bihar	82.3	17.7
Madhya Pradesh	75.9	24.1
Jharkhand	80.9	19.1
Uttar Pradesh	78.4	21.6
Rajasthan	81.2	18.8
Chhattisgarh	83.8	16.2
Odisha	74.3	25.7
Assam	75.4	24.6
Andhra Pradesh	79.2	20.8
West Bengal	75.5	24.5
Tripura	80.0	20.0
Arunachal Pradesh	77.3	22.7
Meghalaya	82.1	18.0
Karnataka	81.8	18.2
Telangana	79.6	20.4
Gujarat	84.9	15.1
Manipur	84.2	15.8
Jammu and Kashmir†	69.9	30.1
Haryana	82.6	17.4
Uttarakhand	84.6	15.4
Tamil Nadu	77.8	22.2
Mizoram	77.5	22.5
Maharashtra	83.8	16.2
Punjab	79.0	21.0
Sikkim	69.6	30.4
Nagaland	78.8	21.2
Himachal Pradesh	80.3	19.8
UTs other than Delhi	79.9	20.1
Kerala	75.0	25.1
Delhi	79.8	20.2
Goa	74.8	25.3

UTs=Union territories.
*The states are listed in increasing order of Socio-demographic Index in 2017.
†The state of Jammu and Kashmir was divided into two union territories in August 2019; as we are reporting findings up to 2017, we report findings for the undivided state of Jammu and Kashmir.

19. Causes of early and late neonatal death in the states of India, 2017

States*	Percentage of early and late neonatal deaths (95% uncertainty interval): Neonatal preterm birth		Neonatal encephalopathy due to birth asphyxia and trauma		Neonatal sepsis and other neonatal infections		Hemolytic disease and neonatal jaundice		Other neonatal disorders	
	Early neonatal	Late neonatal	Early neonatal	Late neonatal	Early neonatal	Late neonatal	Early neonatal	Late neonatal	Early neonatal	Late neonatal
**India**	**30.6 (26.6-34.5)**	**16.7 (13.9-21.2)**	**16.6 (14.1-23.0)**	**6.1 (4.9-8.1)**	**5.2 (3.3-6.5)**	**9.5 (6.7-12.1)**	**2.8 (2.3-3.3)**	**5.0 (4.1-6.0)**	**24.0 (18.4-27.3)**	**14.2 (12.3-16.3)**
**Low SDI**	**28.7 (23.7-34.3)**	**14.8 (11.3-21.1)**	**15.6 (12.7-22.9)**	**5.6 (4.2-8.0)**	**5.6 (3.1-7.3)**	**9.8 (6.2-12.8)**	**2.7 (2.1-3.4)**	**4.4 (3.4-5.8)**	**25.7 (18.5-30.9)**	**14.5 (11.8-17.5)**
Bihar	21.6 (15.7-33.0)	11.6 (7.9-19.7)	21.1 (17.0-26.4)	6.1 (3.6-9.0)	4.9 (2.8-7.2)	7.5 (4.3-11.7)	3.3 (2.3-4.6)	4.3 (2.4-6.9)	26.0 (18.6-32.6)	11.7 (8.3-16.1)
Madhya Pradesh	34.5 (27.3-41.0)	19.7 (15.1-24.6)	10.5 (6.4-22.6)	4.1 (2.4-8.4)	4.1 (2.6-6.1)	8.2 (5.5-11.8)	2.3 (1.6-3.1)	4.8 (3.0-7.2)	29.5 (20.0-37.2)	19.1 (12.4-24.6)
Jharkhand	27.7 (20.4-34.5)	14.7 (9.9-20.6)	18.4 (13.6-23.6)	7.2 (4.5-11.0)	5.7 (3.3-8.4)	11.4 (6.5-16.6)	2.2 (1.4-3.2)	3.7 (2.1-6.2)	24.0 (18.3-30.5)	14.0 (9.7-18.4)
Uttar Pradesh	29.7 (23.0-36.0)	13.6 (9.0-22.1)	13.6 (9.3-23.0)	5.3 (3.3-8.2)	6.5 (3.3-9.5)	10.5 (6.1-15.0)	2.8 (2.0-3.9)	4.6 (2.8-7.1)	25.5 (17.5-32.1)	13.9 (10.0-18.0)
Rajasthan	31.9 (25.2-38.0)	17.2 (12.4-22.5)	11.7 (7.9-23.0)	4.5 (2.5-8.1)	5.6 (2.8-8.2)	11.5 (6.7-16.1)	1.2 (0.8-1.8)	2.0 (1.2-3.2)	25.7 (18.0-32.9)	15.1 (11.0-19.5)
Chhattisgarh	32.6 (25.9-39.6)	20.2 (16.1-25.1)	16.2 (11.7-24.7)	7.2 (4.6-10.1)	3.3 (2.1-4.8)	7.4 (4.7-11.6)	2.7 (1.8-3.8)	4.6 (2.9-6.7)	26.9 (17.3-34.2)	17.4 (13.1-22.3)
Odisha	24.5 (18.6-35.1)	13.7 (9.2-22.6)	22.9 (17.9-28.1)	7.9 (5.4-11.2)	6.7 (3.8-9.9)	12.2 (7.3-17.5)	3.6 (2.5-5.1)	6.6 (4.0-9.8)	24.8 (18.2-31.6)	17.2 (11.8-22.6)
Assam	30.4 (24.4-36.0)	16.2 (11.4-23.0)	19.4 (15.1-24.1)	7.0 (4.8-9.9)	5.4 (2.7-7.6)	9.6 (5.8-14.0)	2.9 (2.0-4.1)	5.3 (3.2-8.2)	21.0 (15.8-26.5)	12.5 (8.8-17.0)
**Middle SDI**	**35.6 (31.5-39.3)**	**21.5 (17.9-24.5)**	**19.2 (16.7-23.8)**	**7.2 (5.9-8.8)**	**3.9 (3.1-5.6)**	**8.1 (6.2-12.6)**	**3.1 (2.5-3.8)**	**6.7 (5.3-8.4)**	**19.7 (17.0-22.8)**	**13.7 (11.5-16.2)**
Andhra Pradesh	39.5 (32.2-45.7)	27.2 (18.3-33.9)	23.8 (19.1-28.7)	9.0 (6.3-12.6)	3.1 (2-6.2)	7.2 (4.5-13.2)	4.1 (3.0-5.6)	9.1 (5.8-13.4)	14.0 (10.3-19.8)	10.6 (7.3-14.6)
West Bengal	34.7 (28.9-40.9)	18.4 (13.8-23.6)	15.8 (11.8-22.9)	5.0 (3.2-8.1)	4.1 (2.8-6.2)	7.4 (4.7-11.6)	4.3 (3.1-5.8)	7.9 (4.9-11.9)	20.6 (16.4-25.5)	12.3 (8.6-16.7)
Tripura	34.6 (28.7-41.4)	16.7 (12.6-21.1)	13.6 (9.6-22.7)	5.2 (3.5-7.5)	7.7 (4.7-11.1)	13.2 (9.0-18.3)	3.0 (1.8-4.4)	4.2 (2.5-6.7)	16.9 (13.0-21.6)	10.1 (7.4-13.7)
Arunachal Pradesh	32.4 (27.2-37.9)	16.9 (12.4-22.6)	17.3 (12.1-22.8)	6.2 (3.9-9.3)	7.5 (4.6-11.3)	11.3 (6.8-16.7)	4.1 (2.8-5.8)	6.8 (4.2-10.4)	20.7 (16.0-25.5)	11.8 (8.2-17.0)
Meghalaya	28.1 (21.3-37.0)	16.1 (11.2-22.9)	25.9 (19.7-32.9)	9.6 (6.4-13.8)	6.6 (3.9-10.2)	12.0 (7.7-17.4)	3.1 (1.9-4.8)	4.5 (2.8-6.9)	16.2 (11.7-21.6)	9.7 (6.7-14.4)
Karnataka	39.6 (32.3-45.2)	27.4 (19.1-33.0)	19.3 (15.0-24.5)	8.3 (5.9-11.3)	3.5 (2.4-5.9)	8.8 (5.9-14.4)	1.3 (0.9-1.7)	2.7 (1.7-4.1)	19.1 (15.4-23.3)	15.3 (11.7-19.4)
Telangana	40.2 (32.8-46.7)	27.7 (17.9-33.9)	22.7 (17.6-27.9)	8.8 (6.1-12.4)	3.1 (2.0-6.2)	7.4 (4.4-14.8)	4.1 (2.8-5.7)	8.6 (5.1-12.4)	14.0 (10.1-19.5)	10.8 (7.4-15.7)
Gujarat	35.1 (29.6-40.6)	21.5 (17.1-26.1)	20.4 (16.2-24.7)	9.6 (6.0-13.7)	3.5 (2.3-5.4)	7.0 (4.4-13.4)	1.9 (1.4-2.5)	4.0 (2.5-6.1)	20.1 (15.7-24.9)	17.1 (13.3-21.7)
Manipur	29.6 (23.7-38.0)	14.3 (10.5-19.4)	18.6 (14.0-23.8)	7.4 (5.1-10.2)	5.8 (3.6-8.0)	12.0 (7.3-16.3)	5.5 (3.5-8.0)	9.5 (6.5-13.3)	18.4 (12.5-24.1)	9.4 (6.5-13.0)
Jammu and Kashmir*	27.3 (21.6-34.7)	13.4 (8.7-22.4)	15.2 (11.1-22.0)	4.4 (2.4-8.2)	7.0 (3.5-10.1)	10.2 (6.3-14.6)	5.9 (3.8-8.2)	12.7 (8.0-18.7)	23.6 (16.1-29.3)	11.7 (8.0-15.8)
Haryana	27.9 (22.5-34.1)	17.0 (13.5-21.2)	17.4 (13.0-23.0)	6.9 (4.8-9.5)	4.6 (3.0-6.5)	10.1 (6.9-14.5)	2.9 (2.1-3.9)	5.5 (3.6-7.9)	29.6 (20.1-35.9)	20.2 (14.4-25.4)
**High SDI**	**33.8 (30.0-38.8)**	**21.1 (17.5-24.5)**	**18.7 (16.2-23.4)**	**7.4 (6.0-9.0)**	**4.8 (3.6-6.5)**	**9.7 (7.3-14.4)**	**2.6 (2.1-3.2)**	**5.6 (4.5-6.9)**	**20.0 (17.0-23.4)**	**13.4 (11.2-15.9)**
Uttarakhand	26.6 (20.4-34.3)	13.6 (9.9-19.3)	16.3 (12.2-22.9)	5.9 (3.4-8.4)	8.7 (4.0-12.4)	15.0 (8.6-19.5)	3.2 (2.1-4.4)	4.3 (2.8-6.4)	20.7 (16.1-25.8)	11.3 (8.6-15.2)
Tamil Nadu	36.7 (29.3-42.4)	28.1 (18.3-33.7)	24.1 (18.8-29.6)	9.7 (6.3-13.1)	2.9 (1.9-6.4)	7.1 (4.5-16.1)	1.5 (1.0-2.1)	4.4 (2.7-6.8)	12.7 (8.9-21.7)	9.0 (5.8-14.6)
Mizoram	25.9 (20.6-32.9)	15.4 (10.9-22.4)	19.7 (16.0-23.7)	7.9 (5.3-11.0)	6.1 (4.1-8.7)	9.6 (6.3-14.7)	2.2 (1.4-3.0)	3.0 (1.8-4.8)	22.4 (17.8-27.5)	16.6 (10.7-21.5)
Maharashtra	38.3 (31.2-45.0)	22.8 (16.7-27.8)	16.1 (12.2-24.1)	6.4 (3.9-9.1)	5.1 (3.5-7.4)	11.3 (7.5-16.5)	2.2 (1.6-2.9)	4.8 (3.3-6.8)	20.7 (16.5-25.1)	14.7 (11.4-18.5)
Punjab	24.9 (19.2-34.3)	15.4 (11.4-21.4)	21.7 (17.3-26.6)	8.0 (5.7-10.7)	5.2 (3.2-7.2)	10.8 (7.2-14.9)	4.1 (2.8-5.7)	8.4 (5.7-11.8)	23.7 (17.4-30.0)	15.8 (11.8-19.8)
Sikkim	24.0 (18.8-30.1)	12.7 (9.6-16.6)	11.0 (7.7-18.8)	3.5 (2.0-6.0)	9.6 (4.7-14.1)	11.3 (7.6-15.6)	2.2 (1.5-3.2)	5.0 (3.2-7.7)	23.3 (17.4-29.2)	11.1 (7.9-15.2)
Nagaland	28.7 (21.6-38.0)	12.3 (7.7-20.1)	17.1 (10.9-23.6)	5.3 (2.7-8.5)	9.9 (3.6-14.9)	11.9 (6.7-16.8)	9.9 (5.7-14.6)	13.8 (9.0-19.0)	13.0 (8.6-17.8)	6.4 (4.3-9.9)
Himachal Pradesh	27.3 (20.3-34.7)	16.8 (11.9-22.5)	22.5 (17.7-28.0)	9.0 (6.0-12.6)	3.6 (2.3-5.6)	8.5 (5.2-12.7)	3.7 (2.4-5.4)	7.5 (4.4-11.9)	23.0 (16.7-30.0)	14.9 (10.4-19.8)
UTs other than Delhi	34.8 (28.8-41.0)	20.0 (15.3-25.9)	21.0 (16.3-25.9)	8.2 (5.8-11.4)	4.1 (2.8-6.0)	8.0 (5.2-12.2)	5.7 (3.9-7.9)	10.6 (7.1-14.8)	14.1 (10.6-18.5)	8.5 (6.0-12.7)
Kerala	29.7 (23.9-35.2)	20.2 (15.1-27.8)	16.9 (12.8-21.7)	5.7 (3.6-8.7)	5.1 (3.4-8.5)	8.0 (5.0-15.3)	1.3 (0.8-1.8)	2.9 (1.6-4.5)	10.5 (7.5-20.6)	6.3.0 (4.0-12.7)
Delhi	27.6 (20.9-37.3)	17.4 (11.6-25.2)	19.9 (14.1-25.8)	7.8 (5.0-11.4)	3.1 (1.9-5.4)	6.1 (3.6-11.8)	3.1 (1.9-4.6)	6.5 (4.0-10.3)	28.8 (18.5-36.7)	19.8 (13.5-26.4)
Goa	25.8 (20.7-31.1)	14.3 (10.2-19.5)	15.2 (11.2-19.7)	5.5 (3.8-7.8)	4.3 (1.8-7.4)	7.3 (3.9-11.5)	5.1 (3.4-7.3)	10.2 (7.2-13.4)	25.8 (19.9-32.5)	13.9 (10.0-18.3)

States*	Percentage of neonatal deaths (95% uncertainty interval): Lower respiratory infections		Diarrhoeal diseases		Tetanus		Congenital birth defects		Other causes	
	Early neonatal	Late neonatal	Early neonatal	Late neonatal	Early neonatal	Late neonatal	Early neonatal	Late neonatal	Early neonatal	Late neonatal
**India**	**8.1 (6.9-9.4)**	**22.4 (20.0-24.8)**	**1.4 (1.1-1.8)**	**7.7 (6.1-9.4)**	**0.45 (0.26-0.84)**	**1.84 (0.91-3.56)**	**8.2 (7.1-9.4)**	**10.1 (8.8-11.2)**	**2.8 (1.5-4.6)**	**6.5 (4.7-8.8)**
**Low SDI**	**9.1 (7.7-10.9)**	**24.3 (21.3-27.3)**	**1.7 (1.3-2.2)**	**8.9 (6.9-11.3)**	**0.60 (0.30-1.10)**	**2.30 (1.10-4.70)**	**7.8 (6.3-9.3)**	**9.1 (7.5-10.6)**	**2.5 (1.4-4.0)**	**6.2 (4.4-8.2)**
Bihar	11.0 (8.5-13.9)	26.4 (21.3-31.5)	2.8 (1.9-4.0)	14.5 (9.7-20.3)	0.39 (0.17-0.78)	1.57 (0.47-3.58)	6.1 (4.7-7.7)	7.7 (5.6-10.1)	2.7 (1.4-4.3)	8.7 (4.5-12.4)
Madhya Pradesh	8.2 (6.3-10.8)	24.5 (20.4-29.2)	0.8 (0.6-1.2)	4.5 (2.9-6.6)	0.13 (0.05-0.27)	0.63 (0.21-1.45)	7.4 (5.8-9.4)	8.8 (6.8-11)	2.6 (1.4-4.5)	5.7 (4.0-8.2)
Jharkhand	9.6 (7.0-12.9)	22.0 (17.2-26.8)	2.0 (1.3-3.0)	10.3 (6.4-15.2)	0.45 (0.19-0.95)	1.86 (0.66-4.23)	7.0 (5.4-9.2)	8.5 (6.4-10.7)	2.9 (1.4-5.1)	6.3 (4.2-9.5)
Uttar Pradesh	7.6 (5.6-10.3)	23.3 (18.6-28.3)	1.8 (1.2-2.6)	9.5 (6.3-14.0)	0.95 (0.43-2.03)	3.76 (1.45-8.49)	9.2 (6.9-12.2)	9.9 (7.4-12.5)	2.4 (1.3-3.9)	5.7 (3.9-7.9)
Rajasthan	14.5 (11.0-18.8)	31.1 (25.8-37.0)	0.9 (0.6-1.3)	5.1 (3.3-7.7)	0.43 (0.17-0.89)	1.66 (0.44-3.53)	5.8 (4.4-7.5)	6.9 (5.2-8.9)	2.1 (1.1-3.5)	5.0 (3.4-7.5)
Chhattisgarh	9.6 (6.8-12.8)	23.6 (19.2-28.5)	1.0 (0.7-1.5)	5.3 (3.4-7.8)	0.23 (0.11-0.46)	1.06 (0.42-2.27)	4.9 (3.7-6.6)	6.4 (4.9-8.1)	2.6 (1.4-4.6)	6.8 (4.5-9.9)
Odisha	5.9 (4.5-7.7)	18.2 (14.3-22.7)	1.2 (0.9-1.8)	7.4 (4.8-11.3)	0.19 (0.08-0.48)	0.82 (0.29-2.10)	7.5 (5.7-9.5)	9.2 (6.9-11.8)	2.7 (1.5-4.6)	6.7 (4.5-10.0)
Assam	6.2 (4.6-8.2)	22.4 (17.7-27.7)	1.5 (1.0-2.1)	8.3 (5.0-12.7)	0.18 (0.08-0.38)	0.77 (0.27-1.79)	11.1 (8.0-14.7)	13.1 (9.1-17.0)	2.1 (1.1-3.8)	4.7 (3.1-7.2)
**Middle SDI**	**5.7 (4.8-6.6)**	**18.4 (16.6-20.6)**	**0.8 (0.7-1.0)**	**4.8 (3.9-5.8)**	**0.10 (0.10-0.30)**	**0.60 (0.30-1.10)**	**8.7 (7.7-9.8)**	**11.8 (10.4-13.3)**	**3.2 (1.7-5.7)**	**7.1 (5.0-10.3)**
Andhra Pradesh	3.6 (2.8-4.5)	12.3 (9.3-15.3)	1.2 (0.9-1.7)	7.4 (4.8-11.2)	0.11 (0.05-0.21)	0.44 (0.15-0.97)	7.2 (5.8-8.7)	9.7 (7.5-12.4)	3.4 (1.7-6.7)	7.1 (4.3-11.4)
West Bengal	7.4 (5.8-9.5)	25.0 (20.5-30.1)	0.6 (0.4-1.0)	4.0 (2.5-6.1)	0.20 (0.10-0.39)	0.73 (0.28-1.67)	8.9 (7.1-11.2)	12.5 (9.6-16)	3.2 (1.7-5.9)	6.7 (4.4-10.3)
Tripura	10.9 (8-14.5)	26.2 (21.9-31.1)	1.0 (0.7-1.5)	5.3 (3.5-7.7)	0.11 (0.04-0.26)	0.39 (0.12-1.00)	9.3 (7.1-12.1)	11.8 (9.4-14.4)	2.8 (1.5-4.9)	7.0 (4.7-9.9)
Arunachal Pradesh	7.1 (5.2-9.6)	24.0 (18.3-29.6)	1.5 (1.0-2.3)	6.5 (4.1-10.1)	0.01 (0.00-0.03)	0.03 (0.01-0.09)	5.7 (4.2-8.5)	8.5 (6.3-12.7)	3.7 (1.8-6.6)	8.0 (5.0-11.8)
Meghalaya	7.3 (5.2-9.9)	22.5 (18.7-27.1)	1.1 (0.7-1.6)	6.6 (4.3-9.6)	0.10 (0.03-0.24)	0.48 (0.10-1.33)	8.7 (6.5-11.5)	10.5 (8.5-12.9)	2.9 (1.4-5.6)	8.0 (4.9-12.4)
Karnataka	3.2 (2.5-4.1)	11.8 (9.4-14.5)	0.6 (0.4-0.8)	3.5 (2.3-5.1)	0.02 (0.01-0.04)	0.12 (0.03-0.32)	10.2 (8.5-12.5)	14.8 (11.9-17.9)	3.2 (1.7-5.8)	7.4 (4.8-11.2)
Telangana	3.5 (2.7-4.6)	12.1 (9.4-15.3)	1.2 (0.8-1.6)	6.9 (4.4-10.3)	0.09 (0.05-0.18)	0.38 (0.16-0.82)	7.2 (5.8-8.9)	9.7 (7.4-12.4)	3.9 (1.8-7.0)	7.7 (4.6-12.2)
Gujarat	6.3 (4.8-8.2)	16.5 (13.4-19.6)	0.8 (0.5-1.1)	4.8 (3.4-6.6)	0.17 (0.07-0.34)	0.68 (0.23-1.45)	9.0 (7.4-11.3)	11.0 (8.8-13.4)	2.9 (1.5-5.3)	7.8 (5.1-11.9)
Manipur	7.8 (5.4-11.1)	20.2 (16.5-24.3)	1.1 (0.7-1.7)	6.6 (4.3-9.5)	0.61 (0.19-1.48)	2.22 (0.60-5.84)	9.2 (6.6-12.5)	10.4 (8.3-12.8)	3.3 (1.6-6.2)	8.0 (5.1-12.2)
Jammu and Kashmir*	8.1 (6.1-10.7)	25.1 (19.2-31.0)	0.5 (0.3-0.7)	2.9 (1.7-4.4)	0.08 (0.03-0.17)	0.31 (0.11-0.74)	9.6 (7.4-12.4)	14.6 (10.9-18.7)	2.7 (1.5-4.7)	4.8 (3.2-7.1)
Haryana	6.1 (4.7-7.8)	17.0 (13.9-20.3)	0.9 (0.6-1.2)	5.0 (3.5-6.8)	0.23 (0.10-0.48)	1.03 (0.44-2.24)	7.1 (5.8-8.8)	9.3 (7.4-11.3)	3.5 (1.8-6.1)	8.0 (5.2-12.3)
**High SDI**	**5.4 (4.5-6.5)**	**16.1 (14.5-17.8)**	**0.7 (0.5-0.9)**	**3.8 (3-4.6)**	**0.20 (0.10-0.40)**	**0.90 (0.50-1.80)**	**9.9 (8.5-11.3)**	**14 (12.6-15.5)**	**3.9 (2.0-7.2)**	**8.1 (5.4-12.4)**
Uttarakhand	10.3 (7.3-13.6)	22.0 (18.2-26.1)	0.8 (0.5-1.2)	4.6 (3.1-6.6)	1.06 (0.51-2.34)	4.64 (1.89-10.14)	8.4 (6.3-11.0)	9.6 (7.6-11.7)	4.0 (1.9-7.5)	9.0 (5.6-14.4)
Tamil Nadu	3.8 (2.9-4.9)	12.7 (10.3-15.4)	0.7 (0.5-1.0)	3.5 (2.4-4.9)	0.0002 (0.0001-0.0010)	0.003 (0.001-0.008)	12.3 (10.0-14.7)	16.7 (13.8-19.8)	5.4 (2.6-9.8)	8.9 (5.6-14.0)
Mizoram	11.9 (8.6-15.4)	27.0 (22.6-31.9)	0.5 (0.4-0.8)	2.0 (1.3-3.1)	0.06 (0.03-0.13)	0.29 (0.11-0.68)	8.1 (6.5-10.2)	11.1 (8.8-13.7)	3.0 (1.6-5.4)	6.9 (4.5-10.1)
Maharashtra	5.3 (4.1-6.9)	16.0 (13.0-19.2)	0.7 (0.5-0.9)	3.8 (2.6-5.3)	0.23 (0.11-0.42)	1.01 (0.44-2.11)	7.9 (6.4-9.8)	10.7 (8.8-13.1)	3.5 (1.8-6.3)	8.3 (5.4-13.5)
Punjab	5.4 (4.0-7.1)	15.9 (12.9-19.2)	0.8 (0.5-1.1)	4.6 (3.2-6.4)	0.18 (0.08-0.42)	0.98 (0.32-2.48)	10.9 (8.6-13.9)	13.7 (11.0-16.9)	3.2 (1.6-5.8)	6.5 (4.2-10.0)
Sikkim	13.2 (9.5-17.2)	33.0 (27.8-38.3)	1.5 (1.0-2.1)	5.1 (3.3-7.7)	0.020 (0.003-0.050)	0.06 (0.01-0.20)	9.0 (6.8-11.4)	11.0 (8.5-13.8)	6.2 (2.7-12.2)	7.2 (4.4-11.7)
Nagaland	12.0 (7.8-16.9)	31.3 (24.3-38.3)	0.7 (0.4-1.2)	2.9 (1.7-4.4)	0.51 (0.15-1.28)	2.45 (0.66-6.64)	4.6 (3.0-8.5)	5.9 (3.9-11.0)	3.5 (1.7-6.9)	7.7 (4.6-12.5)
Himachal Pradesh	6.2 (4.5-8.4)	18.3 (14.6-23.4)	0.6 (0.4-0.9)	4.0 (2.5-6.2)	0.25 (0.07-0.82)	1.57 (0.35-4.98)	9.3 (7.1-12.2)	12.3 (9.4-15.5)	3.5 (1.7-6.6)	7.2 (4.5-11.1)
UTs other than Delhi	5.8 (4.2-7.6)	18.2 (14.6-22.3)	0.6 (0.4-0.9)	3.6 (2.4-5.2)	0.02 (0.01-0.04)	0.08 (0.02-0.22)	10.0 (7.8-12.7)	14.0 (11.0-17.4)	3.9 (1.8-7.2)	8.8 (5.4-13.8)
Kerala	4.0 (2.8-5.2)	12.2 (9.2-15.3)	0.1 (0.1-0.2)	0.7 (0.4-1.1)	0.21 (0.07-0.51)	0.54 (0.13-1.52)	25.4 (19.1-30.8)	34.2 (26.9-40.6)	7.0 (2.9-13.3)	9.3 (5.6-14.9)
Delhi	5.1 (3.6-7.3)	18.1 (13.8-23.2)	0.9 (0.5-1.4)	5.2 (3.1-8.4)	0.19 (0.07-0.46)	0.70 (0.22-1.82)	8.0 (5.9-10.8)	11.1 (8.1-14.5)	3.4 (1.6-6.2)	7.3 (4.5-11.3)
Goa	7.1 (5.4-9.5)	22.3 (18.0-26.8)	0.5 (0.4-0.7)	2.9 (1.8-4.1)	0.29 (0.07-1.26)	1.00 (0.23-3.95)	10.9 (8.7-13.9)	15.3 (12.2-18.8)	5.0 (2.3-9.9)	7.4 (4.3-12.1)

SDI=Socio-demographic Index. UTs=Union territories.
*The states are listed in increasing order of SDI in 2017.
†The state of Jammu and Kashmir was divided into two union territories in August 2019; as we are reporting findings up to 2017, we report findings for the undivided state of Jammu and Kashmir.

20. Risk factors for under-5 death in the states of India, 2017

States[*]	Percentage of under-5 deaths attributable to each risk factor (95% uncertainty interval)									
	Child and maternal malnutrition[†]			Water, sanitation and handwashing (WaSH)			Air pollution			
	Total	Low birth weight and short gestation	Child growth failure	Total	Unsafe water	Unsafe sanitation	Total	Ambient air pollution	Household air pollution	Second-hand smoke
**India**	**68.2 (65.8-70.7)**	**45.9 (44.4-47.5)**	**21.4 (19.5-23.2)**	**10.8 (9.1-12.4)**	**8.0 (5.2-9.9)**	**4.8 (3.8-5.9)**	**8.8 (7.0-10.3)**	**4.8 (3.6-6.0)**	**4.0 (3.0-5.1)**	**1.6 (0.9-2.4)**
**Low SDI**	**69.3 (62.1-77.0)**	**44.7 (40.0-49.9)**	**23.7 (20.5-26.8)**	**12.9 (10.9-14.7)**	**9.6 (6.5-11.9)**	**5.9 (4.6-7.3)**	**9.8 (7.7-11.5)**	**5.3 (3.9-6.7)**	**4.5 (3.3-5.9)**	**1.7 (0.9-2.5)**
Bihar	72.7 (69.0-76.0)	45.4 (41.9-48.5)	26.3 (23.5-29.2)	17.7 (14.7-20.7)	13.6 (9.4-16.9)	8.7 (6.7-10.9)	10.6 (8.1-12.7)	5.9 (3.6-8.1)	4.6 (3.0-6.7)	1.3 (0.6-2.0)
Madhya Pradesh	67.8 (64.1-71.0)	44.6 (41.8-47.1)	22.5 (19.4-25.1)	8.3 (6.4-10.4)	5.7 (3.6-7.9)	3.4 (2.4-4.8)	10.1 (7.8-12.1)	4.2 (2.6-6.2)	5.9 (4.0-7.8)	1.8 (1.0-2.7)
Jharkhand	67.7 (62.7-72.2)	43.5 (37.4-48.4)	23.5 (19.8-27.3)	13.6 (10.4-17.0)	10.0 (5.8-13.5)	6.9 (4.9-9.1)	9.1 (7.0-11.1)	3.8 (2.3-5.7)	5.3 (3.4-7.0)	0.6 (0.3-1.1)
Uttar Pradesh	68.6 (64.7-72.2)	43.3 (40.6-45.9)	24.2 (21.6-26.9)	14.2 (11.4-16.9)	10.9 (7.3-14.2)	6.3 (4.5-8.2)	9.3 (7.0-11.3)	6.1 (4.1-8.1)	3.3 (2.0-4.8)	1.8 (1.0-2.7)
Rajasthan	72.2 (69.2-75.0)	47.5 (45.1-49.4)	23.9 (21.2-26.2)	7.8 (6.1-9.7)	5.2 (3.2-7.1)	3.1 (2.2-4.3)	13.6 (10.5-16.3)	6.3 (3.9-9.0)	7.3 (5.0-9.9)	2.9 (1.6-4.3)
Chhattisgarh	71.6 (67.0-75.3)	53.0 (48.4-56.6)	18.0 (15.0-20.6)	7.8 (5.9-9.6)	5.2 (3.4-7.1)	3.2 (2.2-4.4)	8.4 (6.5-10.3)	2.9 (1.9-4.2)	5.5 (4.0-7.2)	1.1 (0.5-1.8)
Odisha	62.1 (57.2-67.0)	43.1 (40.3-45.6)	18.5 (14.4-22.0)	9.6 (7.1-12.0)	6.9 (4.1-9.4)	4.6 (3.2-6.3)	6.9 (5.2-8.9)	2.5 (1.5-3.8)	4.4 (3.1-5.7)	1.0 (0.5-1.6)
Assam	65.1 (61.2-68.6)	44.1 (40.6-47.6)	20.1 (17.0-22.8)	11.3 (8.7-14.0)	7.7 (3.5-11.0)	5.5 (4.1-7.2)	7.3 (5.5-9.0)	2.5 (1.6-3.5)	4.8 (3.4-6.2)	0.9 (0.4-1.6)
**Middle SDI**	**66.1 (59.2-73.4)**	**49.2 (43.9-54.3)**	**16.3 (14.1-18.7)**	**5.9 (4.8-7.0)**	**4.1 (2.3-5.4)**	**2.2 (1.7-2.8)**	**6.5 (5.2-7.8)**	**3.4 (2.5-4.3)**	**3.1 (2.3-4.0)**	**1.5 (0.8-2.2)**
Andhra Pradesh	67.5 (64.1-70.5)	50.3 (47.7-52.5)	16.7 (14.3-19.0)	8.5 (6.5-10.4)	6.7 (4.0-8.8)	2.9 (2.0-3.9)	4.4 (3.3-5.5)	2.3 (1.7-3.1)	2.1 (1.4-2.8)	1.0 (0.5-1.5)
West Bengal	67.2 (63.7-70.3)	50.2 (47.4-52.6)	16.2 (14.0-18.5)	5.9 (4.5-7.3)	3.5 (2.1-4.8)	2.1 (1.5-2.8)	8.7 (6.8-10.5)	4.3 (3.1-5.7)	4.4 (3.1-5.9)	1.7 (0.9-2.6)
Tripura	67.6 (63.8-71.0)	46.2 (43.4-48.9)	20.7 (17.6-23.2)	7.5 (5.6-9.5)	4.2 (1.8-6.3)	3.0 (2.1-4.1)	10.1 (7.8-12.2)	4.0 (2.7-5.5)	6.0 (4.3-7.8)	1.9 (1.0-3.1)
Arunachal Pradesh	65.7 (60.2-70.6)	45.9 (40.8-50.2)	19.0 (15.4-22.5)	7.9 (5.4-10.6)	5.3 (1.5-8.5)	3.6 (2.4-5.1)	6.2 (4.7-7.9)	2.2 (1.4-3.1)	4.0 (2.9-5.4)	1.0 (0.4-1.7)
Meghalaya	59.1 (53.1-65.7)	37.6 (32.7-41.9)	20.6 (16.3-25.0)	7.9 (5.7-10.3)	5.2 (2.1-7.9)	2.8 (1.8-4.1)	8.2 (6.1-10.4)	2.8 (1.9-3.9)	5.4 (3.8-7.2)	1.8 (0.8-3.0)
Karnataka	63.1 (59.8-66.0)	48.4 (46.1-50.6)	14.2 (12.1-16.2)	4.2 (3.3-5.3)	3.0 (1.7-4.2)	1.8 (1.3-2.4)	4.1 (3.0-5.2)	1.9 (1.1-2.8)	2.2 (1.5-2.9)	0.8 (0.4-1.4)
Telangana	67.9 (64.4-71.1)	53.3 (50.2-56.0)	14.1 (11.9-16.4)	7.2 (5.5-9.1)	5.7 (3.5-7.8)	2.4 (1.6-3.4)	4.0 (3.0-5.2)	2.5 (1.7-3.4)	1.6 (1.1-2.2)	0.8 (0.4-1.3)
Gujarat	66.2 (62.8-69.4)	50.3 (47.7-52.6)	15.4 (13.1-17.7)	4.7 (3.5-6.1)	3.4 (1.7-5.0)	2.1 (1.4-2.9)	6.0 (4.6-7.6)	2.8 (1.7-4.3)	3.3 (2.2-4.4)	1.7 (1.0-2.5)
Manipur	61.8 (56.8-66.1)	46.3 (41.1-50.8)	14.5 (11.5-16.9)	8.1 (5.9-10.1)	5.5 (2.3-7.8)	3.4 (2.3-4.5)	5.8 (4.3-7.4)	2.8 (1.9-3.9)	3.0 (2.1-4.0)	0.9 (0.4-1.6)
Jammu and Kashmir[‡]	66.2 (63.2-68.8)	48.5 (46.2-50.9)	16.7 (14.3-18.7)	4.3 (2.8-5.7)	2.5 (0.8-4.1)	1.6 (1.0-2.2)	9.3 (7.1-11.9)	5.4 (3.6-7.7)	3.9 (2.6-5.6)	2.4 (1.3-3.7)
Haryana	65.4 (62.3-68.3)	42.7 (40.6-44.6)	21.7 (19.0-23.9)	6.9 (4.9-9.1)	5.2 (2.6-7.8)	2.5 (1.6-3.6)	8.6 (6.5-10.7)	6.2 (4.0-8.3)	2.5 (1.5-3.8)	2.1 (1.2-3.1)
**High SDI**	**64.3 (57.2-72.3)**	**49.8 (44.0-56.1)**	**13.9 (11.7-16.3)**	**4.5 (3.3-5.5)**	**3.3 (1.7-4.5)**	**1.4 (1.0-1.8)**	**5.3 (4.2-6.4)**	**3.6 (2.8-4.5)**	**1.7 (1.2-2.2)**	**1.0 (0.5-1.5)**
Uttarakhand	64.4 (60.4-68.2)	45.8 (42.0-49.2)	17.8 (15.3-20.1)	5.8 (4.3-7.5)	3.8 (1.6-5.9)	2.7 (1.9-3.7)	8.5 (6.4-10.6)	5.5 (3.4-7.9)	3.0 (1.9-4.4)	2.3 (1.3-3.4)
Tamil Nadu	59.8 (56.1-63.0)	45.5 (42.4-48.4)	13.8 (11.7-15.9)	4.9 (3.6-6.2)	3.5 (1.7-4.9)	1.5 (1.0-2.2)	3.5 (2.4-5.1)	2.4 (1.4-4.0)	1.1 (0.7-1.5)	0.9 (0.5-1.3)
Mizoram	60.8 (56.4-65.3)	42.0 (37.7-45.8)	18.1 (15.1-21.2)	3.6 (2.5-4.8)	1.8 (0.6-2.8)	0.8 (0.5-1.1)	8.4 (6.5-10.4)	4.4 (3.2-5.8)	4.0 (2.8-5.5)	2.8 (1.4-4.5)
Maharashtra	66.9 (63.1-70.5)	53.6 (49.5-57.0)	12.9 (10.8-15.0)	3.9 (2.8-5.0)	2.9 (1.5-4.2)	1.4 (0.9-2.0)	4.9 (3.7-6.0)	3.1 (2.1-4.0)	1.8 (1.2-2.5)	0.8 (0.5-1.3)
Punjab	63.5 (59.8-66.9)	44.8 (41.0-48.1)	17.8 (15.4-19.9)	7.1 (5.0-9.1)	5.5 (3.0-7.8)	1.9 (1.2-2.7)	6.6 (4.9-8.3)	5.0 (3.5-6.7)	1.6 (1.0-2.3)	1.0 (0.5-1.6)
Sikkim	62.7 (57.9-67.6)	43.7 (38.5-48.0)	17.3 (14.0-20.9)	4.0 (2.1-6.1)	2.6 (0.4-4.9)	0.6 (0.3-1.1)	9.3 (7.1-11.5)	5.7 (3.9-7.7)	3.6 (2.5-5.0)	1.5 (0.7-2.5)
Nagaland	61.8 (56.5-66.5)	41.5 (37.1-45.4)	19.5 (16.4-22.6)	5.1 (3.6-6.6)	2.5 (0.6-4.1)	1.6 (1.0-2.3)	10.3 (7.8-13.1)	4.4 (3.1-6.0)	5.9 (4.1-8.0)	1.7 (0.7-2.8)
Himachal Pradesh	64.8 (61.3-68.0)	49.0 (46.2-51.6)	15.1 (12.7-17.3)	4.5 (3.0-6.1)	3.2 (1.3-4.8)	1.2 (0.7-1.8)	6.5 (4.9-8.3)	3.2 (1.9-4.7)	3.4 (2.2-4.6)	1.3 (0.6-2.1)
UTs other than Delhi	63.2 (59.6-66.8)	51.0 (47.3-54.5)	11.6 (9.8-13.2)	4.0 (2.7-5.1)	2.9 (1.3-4.3)	0.9 (0.6-1.4)	4.4 (2.8-6.3)	3.5 (2.0-5.4)	0.9 (0.6-1.3)	1.5 (0.9-2.3)
Kerala	50.8 (46.0-56.6)	44.5 (40.1-50.1)	6.0 (4.5-8.1)	1.2 (0.8-1.6)	0.4 (0.1-0.6)	0.1 (0.1-0.2)	2.2 (1.6-2.9)	1.0 (0.7-1.4)	1.2 (0.8-1.7)	0.6 (0.3-1.0)
Delhi	68.5 (63.5-72.5)	54.2 (48.7-58.7)	13.8 (11.8-15.9)	4.4 (2.5-6.2)	3.5 (1.4-5.5)	0.8 (0.4-1.2)	6.8 (5.2-8.5)	6.8 (5.1-8.4)	0.0 (0.0-0.1)	1.0 (0.5-1.7)
Goa	60.8 (56.1-64.9)	47.7 (43.4-51.4)	12.6 (10.3-14.8)	2.0 (1.1-3.1)	1.3 (0.2-2.4)	0.4 (0.2-0.6)	3.8 (2.5-5.1)	3.1 (2.0-4.4)	0.6 (0.4-1.0)	0.7 (0.3-1.2)

SDI=Socio-demographic Index. UTs=Union territories.
[*]The states are listed in increasing order of SDI in 2017.
[†]Data for two major contributors are shown
[‡]The state of Jammu and Kashmir was divided into two union territories in August 2019; as we are reporting findings up to 2017, we report findings for the undivided state of Jammu and Kashmir.

21. Risk factors for neonatal death in the states of India, 2017

	Percentage of neonatal deaths attributable to each risk factor (95% uncertainty interval)								
	Child and maternal malnutrition†		Water, sanitation and handwashing (WaSH)			Air pollution			
**States of India***	**Total**	**Low birth weight and short gestation**	**Total**	**Unsafe water**	**Unsafe sanitation**	**Total**	**Ambient air pollution**	**Household air pollution**	**Second-hand smoke**
**India**	**83.0 (80.6-85.0)**	**82.8 (77.6-88.4)**	**3.5 (2.8-4.2)**	**2.2 (1.4-2.8)**	**1.3 (1.0-1.7)**	**5.4 (4.3-6.5)**	**2.9 (2.1-3.7)**	**2.5 (1.9-3.2)**	**1.0 (0.5-1.5)**
**Low SDI**	**83.4 (81.0-85.5)**	**83.1 (76.6-90.9)**	**4.3 (3.4-5.1)**	**2.7 (1.8-3.5)**	**1.6 (1.2-2.1)**	**6.3 (5.0 to 7.6)**	**3.4 (2.4-4.4)**	**2.9 (2.2-3.8)**	**1.1 (0.6-1.6)**
Bihar	85.2 (82.7-87.5)	85.0 (72.0-102.4)	6.2 (4.9-7.6)	4.1 (2.6-5.5)	2.6 (1.9-3.5)	7.4 (5.6-9.3)	4.1 (2.4-5.8)	3.3 (2.0-4.9)	0.9 (0.4-1.5)
Madhya Pradesh	84.4 (81.9-86.4)	84.2 (71.8-100.1)	2.7 (2.0-3.5)	1.4 (0.9-2.0)	0.8 (0.6-1.2)	6.0 (4.6-7.6)	2.5 (1.5-3.9)	3.5 (2.3-4.8)	1.1 (0.5-1.7)
Jharkhand	83.9 (80.8-86.5)	83.7 (69.3-101.0)	4.7 (3.4-6.1)	2.9 (1.6-4.2)	2.0 (1.4-2.9)	6.1 (4.5-8.0)	2.6 (1.5-4.0)	3.5 (2.2-5.1)	0.4 (0.2-0.8)
Uttar Pradesh	81.5 (77.9-84.5)	81.2 (68.7-96.3)	4.4 (3.2-5.6)	2.9 (1.8-4.0)	1.7 (1.1-2.3)	5.8 (4.3-7.4)	3.7 (2.4-5.2)	2.1 (1.2-3.1)	1.1 (0.7-1.7)
Rajasthan	86.2 (83.9-88.0)	86.0 (73.4-101.6)	2.8 (2.1-3.7)	1.4 (0.8-2.0)	0.8 (0.6-1.2)	8.8 (6.6-11.4)	4.1 (2.4-6.1)	4.7 (3.1-6.7)	1.9 (1.0-2.9)
Chhattisgarh	86.7 (84.0-88.8)	86.6 (73.5-102.1)	2.8 (2.0-3.7)	1.4 (0.9-2.0)	0.9 (0.6-1.2)	5.6 (4.0-7.3)	2.0 (1.2-3.0)	3.6 (2.5-5.1)	0.7 (0.3-1.2)
Odisha	83.0 (80.0-85.5)	82.8 (69.6-99.6)	3.6 (2.7-4.8)	2.3 (1.3-3.4)	1.5 (1.1-2.2)	4.1 (3.1-5.4)	1.5 (0.9-2.3)	2.6 (1.8-3.6)	0.6 (0.3-1.0)
Assam	80.8 (77.2-83.8)	80.5 (67.4-98.0)	4.0 (2.8-5.3)	2.3 (1.0-3.6)	1.7 (1.1-2.4)	4.5 (3.3-5.9)	1.5 (1.0-2.2)	2.9 (2.0-3.9)	0.6 (0.3-1.0)
**Middle SDI**	**82.9 (80.2-84.7)**	**82.8 (75.7-89.8)**	**2.0 (1.6-2.4)**	**1.2 (0.7-1.6)**	**0.7 (0.5-0.8)**	**3.6 (2.8-4.3)**	**1.8 (1.4-2.3)**	**1.7 (1.3-2.3)**	**0.8 (0.4-1.2)**
Andhra Pradesh	84.6 (81.6-86.7)	84.5 (63.6-112.6)	2.7 (2.0-3.6)	2.0 (1.2-2.9)	0.9 (0.6-1.4)	2.0 (1.5-2.5)	1.0 (0.7-1.4)	1.0 (0.7-1.3)	0.4 (0.2-0.7)
West Bengal	82.1 (78.8-84.7)	81.9 (68.5-99.0)	2.6 (1.9-3.4)	1.2 (0.7-1.8)	0.7 (0.5-1.1)	5.7 (4.4-7.2)	2.7 (1.8-3.8)	3.0 (2.0-4.1)	1.1 (0.6-1.8)
Tripura	82.6 (79.2-85.3)	82.4 (66.6-103.7)	3.0 (2.2-4.0)	1.4 (0.5-2.1)	0.9 (0.7-1.3)	6.0 (4.4-7.8)	2.5 (1.7-3.5)	3.5 (2.4-4.9)	1.2 (0.6-1.9)
Arunachal Pradesh	85.1 (81.6-87.8)	84.9 (68.8-105.6)	2.6 (1.8-3.7)	1.6 (0.4-2.6)	1.1 (0.7-1.6)	3.9 (2.8-5.2)	1.6 (1.0-2.4)	2.3 (1.5-3.2)	0.6 (0.3-1.2)
Meghalaya	83.0 (79.3-86.3)	82.8 (68.9-98.9)	2.5 (1.8-3.4)	1.4 (0.6-2.3)	0.8 (0.5-1.2)	4.2 (3.0-5.6)	1.6 (1.0-2.2)	2.7 (1.9-3.7)	0.9 (0.4-1.6)
Karnataka	81.9 (78.7-84.1)	81.8 (69.6-96.1)	1.2 (0.9-1.6)	0.8 (0.4-1.2)	0.5 (0.3-0.7)	1.7 (1.2-2.2)	0.8 (0.5-1.2)	0.9 (0.6-1.2)	0.4 (0.2-0.6)
Telangana	84.2 (81.0-86.5)	84.1 (63.4-112.7)	2.6 (1.9-3.4)	1.9 (1.2-2.8)	0.9 (0.5-1.3)	2.0 (1.5-2.7)	1.1 (0.8-1.6)	0.9 (0.6-1.2)	0.4 (0.2-0.7)
Gujarat	83.2 (80.2-85.4)	83.1 (71.3-96.6)	1.4 (1.0-1.8)	0.9 (0.4-1.3)	0.5 (0.4-0.7)	3.2 (2.3-4.1)	1.6 (0.9-2.4)	1.6 (1.1-2.3)	0.8 (0.5-1.3)
Manipur	81.8 (77.4-85.6)	81.7 (66.1-102.7)	2.6 (1.8-3.6)	1.4 (0.5-2.2)	0.9 (0.6-1.3)	3.9 (2.7-5.4)	1.9 (1.2-2.8)	2.0 (1.3-2.9)	0.6 (0.2-1.1)
Jammu and Kashmir‡	80.8 (77.6-84.0)	80.6 (68.5-96.7)	1.5 (1.0-2.1)	0.7 (0.2-1.2)	0.4 (0.3-0.7)	5.4 (3.8-7.1)	3.1 (1.9-4.7)	2.3 (1.4-3.3)	1.4 (0.7-2.2)
Haryana	83.7 (80.9-86.0)	83.6 (71.4-97.9)	1.5 (1.1-2.0)	1.0 (0.5-1.6)	0.5 (0.3-0.6)	3.8 (2.9-4.9)	2.9 (1.9-3.9)	1.0 (0.5-1.5)	0.9 (0.5-1.4)
**High SDI**	**80.7 (77.4-83.3)**	**80.6 (73.7-89.0)**	**1.4 (1.0-1.7)**	**0.9 (0.4-1.2)**	**0.4 (0.3-0.5)**	**2.9 (2.3-3.6)**	**2.0 (1.5-2.6)**	**0.9 (0.6-1.2)**	**0.6 (0.3-0.9)**
Uttarakhand	81.2 (77.0-84.9)	81.1 (67.1-97.3)	1.7 (1.2-2.3)	0.9 (0.4-1.4)	0.6 (0.4-0.9)	5.2 (3.6-7.0)	3.4 (2.0-5.1)	1.8 (1.0-2.9)	1.4 (0.8-2.2)
Tamil Nadu	77.9 (73.7-81.2)	77.8 (66.1-92.0)	1.5 (1.1-2.0)	1.0 (0.5-1.4)	0.4 (0.3-0.6)	1.7 (1.2-2.4)	1.1 (0.7-1.8)	0.6 (0.4-0.9)	0.4 (0.2-0.6)
Mizoram	82.9 (80.1-85.4)	82.8 (67.9-100.4)	1.6 (1.1-2.2)	0.5 (0.2-0.9)	0.2 (0.1-0.4)	5.3 (3.9-7.1)	2.7 (1.9-3.7)	2.6 (1.6-3.8)	1.8 (0.9-2.9)
Maharashtra	83.1 (79.9-85.5)	83.0 (70.1-98.1)	1.2 (0.9-1.6)	0.8 (0.4-1.2)	0.4 (0.2-0.6)	2.8 (2.1-3.6)	1.8 (1.3-2.5)	1.0 (0.6-1.4)	0.5 (0.3-0.8)
Punjab	80.2 (76.2-83.2)	80.0 (67.7-94.2)	1.6 (1.1-2.2)	1.1 (0.6-1.6)	0.4 (0.2-0.6)	3.2 (2.4-4.2)	2.4 (1.7-3.4)	0.8 (0.5-1.2)	0.5 (0.2-0.8)
Sikkim	79.7 (75.1-83.4)	79.4 (62.1-102.0)	1.8 (1.0-2.8)	1.0 (0.1-2.1)	0.3 (0.1-0.4)	7.4 (5.5-9.6)	4.6 (3.1-6.4)	2.8 (1.9-4.1)	1.2 (0.6-2.1)
Nagaland	85.4 (80.5-89.0)	85.2 (67.3-108.5)	2.1 (1.4-3.0)	0.7 (0.2-1.3)	0.4 (0.3-0.7)	6.6 (4.6-9.2)	2.8 (1.8-4.2)	3.8 (2.4-5.4)	1.1 (0.5-1.9)
Himachal Pradesh	81.6 (78.0-84.7)	81.5 (66.5-99.1)	1.3 (0.9-1.8)	0.8 (0.4-1.3)	0.3 (0.2-0.5)	3.3 (2.4-4.5)	1.6 (0.9-2.5)	1.7 (1.1-2.4)	0.6 (0.3-1.1)
UTs other than Delhi	81.1 (77.2-84.2)	81.0 (65.9-100.0)	1.3 (0.9-1.8)	0.8 (0.3-1.3)	0.3 (0.2-0.4)	2.7 (1.6-4.0)	2.1 (1.2-3.5)	0.5 (0.3-0.8)	0.9 (0.5-1.4)
Kerala	62.5 (56.3-70.2)	62.5 (51.8-75.3)	0.7 (0.5-1.0)	0.2 (0.0-0.3)	0.1 (0.0-0.1)	1.6 (1.1-2.1)	0.7 (0.5-1.0)	0.9 (0.6-1.3)	0.5 (0.2-0.8)
Delhi	83.2 (79.0-86.3)	83.0 (67.3-102.7)	1.4 (0.8-2.1)	1.0 (0.4-1.7)	0.2 (0.1-0.4)	3.9 (2.8-5.4)	3.9 (2.8-5.4)	0.0 (0.0-0.0)	0.6 (0.3-1.0)
Goa	78.3 (73.9-81.7)	78.2 (55.9-108.8)	0.8 (0.5-1.2)	0.4 (0.1-0.8)	0.1 (0.1-0.2)	2.5 (1.7-3.6)	2.1 (1.3-3.1)	0.4 (0.2-0.7)	0.5 (0.2 to 0.8)

SDI=Socio-demographic Index. UTs=Union territories.
*The states are listed in increasing order of SDI in 2017.
†Data for two major contributors are shown
‡The state of Jammu and Kashmir was divided into two union territories in August 2019; as we are reporting findings up to 2017, we report findings for the undivided state of Jammu and Kashmir.
